# Altered gene expression and repressed markers of autophagy in skeletal muscle of insulin resistant patients with type 2 diabetes

**DOI:** 10.1038/srep43775

**Published:** 2017-03-02

**Authors:** Andreas Buch Møller, Ulla Kampmann, Jakob Hedegaard, Kasper Thorsen, Iver Nordentoft, Mikkel Holm Vendelbo, Niels Møller, Niels Jessen

**Affiliations:** 1 Research Laboratory for Biochemical Pathology, Department of Clinical Medicine, Aarhus University, Denmark; 2 Department of Internal Medicine and Endocrinology, Aarhus University Hospital, Denmark; 3 Department of Molecular Medicine, Aarhus University Hospital, Denmark; 4 Department of Nuclear Medicine and PET Center, Aarhus University Hospital, Denmark; 5 Medical Research Laboratory, Department of Clinical Medicine, Aarhus University Hospital, Denmark; 6 Department of Clinical Pharmacology, Aarhus University Hospital, Denmark

## Abstract

This case-control study was designed to investigate the gene expression profile in skeletal muscle from severely insulin resistant patients with long-standing type 2 diabetes (T2D), and to determine associated signaling pathways. Gene expression profiles were examined by whole transcriptome, strand-specific RNA-sequencing and associated signaling was determined by western blot. We identified 117 differentially expressed gene transcripts. Ingenuity Pathway Analysis related these differences to abnormal muscle morphology and mitochondrial dysfunction. Despite a ~5-fold difference in plasma insulin, we did not observe any difference in phosphorylation of AKT or AS160, although other insulin-sensitive cascades, as mTOR/4EBP1, had retained their sensitivity. Autophagy-related gene (*ATG14, RB1CC1*/*FIP200, GABARAPL1, SQSTM1*/*p62, and WIPI1*) and protein (LC3BII, SQSTM1/p62 and ATG5) expression were decreased in skeletal muscle from the patients, and this was associated with a trend to increased phosphorylation of the insulin-sensitive regulatory transcription factor FOXO3a. These data show that gene expression is highly altered and related to mitochondrial dysfunction and abnormal morphology in skeletal muscle from severely insulin resistant patients with T2D, and that this is associated with decreased expression of autophagy-related genes and proteins. We speculate that prolonged treatment with high doses of insulin may suppress autophagy thereby generating a vicious cycle maintaining insulin resistance.

Type 2 diabetes (T2D) is a complex disease that affects millions of people worldwide and the prevalence is increasing rapidly^1^. The disease is characterized by impaired insulin action and accompanied hyperglycemia^2^. Exogenous insulin is commonly used to treat these patients, but some patients are extremely insulin resistant and represent a major clinical challenge in terms of achieving glycemic control despite treatment with high doses of insulin^3^.

Skeletal muscle is the major organ for insulin-stimulated glucose uptake in humans^4^, and insulin resistance in skeletal muscle is a major contributor to hyperglycemia in T2D^5^. Insulin resistant skeletal muscle is characterized by abnormal morphology, including lipid accumulation and dysfunctional mitochondria^6^,^7^. The molecular bases of these impairments are unknown but altered gene expression has been ascribed a critical role^8^. In accordance to this, gene expression profiles from patients in the early stage of T2D include up to 100 abnormally expressed genes and many of these have structural/contractile properties or are involved in mitochondrial function and metabolism^9^,^10^. Whether these differences in gene expression persist or are worsened in late stages of T2D are not known.

Insulin stimulates several intracellular signaling cascades in skeletal muscle, including signaling to glucose transport, protein synthesis, and autophagy^11^. Insulin treatment to patients with T2D is primarily dosed in order to obtain glycemic control, but is often complicated in the late stage of T2D due to gradually increasing insulin requirements^12^. Impaired insulin signaling to glucose transport does not necessarily translate into similar reductions in the activation of other insulin sensitive pathways. Increasing doses of insulin may therefore have unintended effects on cellular homeostasis and exaggerate the diabetic gene expression profile in these patients. Obtaining glycemic control through treatment with high doses of insulin might ultimately cause a vicious cycle where insulin resistance in skeletal muscle is worsened by the treatment. The potential consequences of treatment with high doses of insulin include excessive stimulation of growth promoting pathways and impaired cellular housekeeping through autophagy. Studies in transgenic mice have demonstrated that insufficient autophagy is associated with impaired function of insulin-sensitive tissues, including skeletal muscle^13^,^14^. Moreover, autophagy-deficient skeletal muscle displays many of the same characteristics as insulin resistant muscle, including both abnormal muscle morphology and mitochondria dysfunction^15^. Insulin has previously been shown to inhibit autophagy in human skeletal muscle^16^,^17^, and we speculate that chronic exposure to high levels of insulin may inhibit autophagy and thereby maintain insulin resistance.

The aim of the present study was to investigate global gene expression in skeletal muscle from severely insulin resistant patients with T2D treated with high doses of insulin. We hypothesized that skeletal muscle from these patients are characterized by abnormal expression of genes encoding structural and functional proteins, and that this is associated with aberrant regulation of insulin sensitive signaling cascades.

## Materials and Methods

### Study design

In the present study, we compare global gene expression in skeletal muscle from healthy human subjects and severely insulin resistant patients with long standing T2D. The study design and data of different nature from the same cohort have been presented previously^3^,^18^.

### Subjects

Seven T2D patients with severe insulin resistance (five males and two females) and seven age matched healthy human subjects (six males and one female) participated in the study after verbal and written information and consent. Severe insulin resistance was defined as insulin requirements of more than 100 U * day^−1^. The study was approved by the Ethics Committee System of Central Region Denmark and conducted in accordance with the Helsinki Declaration.

### Protocol

T2D patients had their oral antidiabetic treatments (metformin) withdrawn two day before the study and their usual insulin treatment was replaced with a continuous infusion of short acting insulin (Actrapid, Novo Nordisk, Denmark) and glucose one day before the study. The rates of insulin and glucose infusions were adjusted to reach a plasma glucose level of 8 mM. Skeletal muscle biopsies were sampled after overnight fast from m. vastus lateralis using a Bergström needle and blood samples were drawn from an antecubital vein. The biopsies were frozen in liquid nitrogen and stored at −80 °C until analyses were performed. Blood samples were handled and analyzed as previously described^3^,^18^.

### RNA sequencing

Total RNA was purified from frozen biopsies using the QiaSymphony robot in combination with the QiaSymphony RNA Mini kit (Qiagen, CA, USA) according to the Manufacturers protocol including DNase treatment. We were not able to isolate muscle RNA from one of the diabetic patients and one of the control subjects, leaving 6 patients in each group for RNA-sequencing. RNA concentration was determined using a spectrophotometer with absorbance at 260 nM (NanoDrop ND-1000) and RNA integrity was assessed using a 2100 Bioanalyzer (Agilent Technologies, Santa Clara, CA, USA). Whole transcriptome, strand-specific RNA-Seq libraries facilitating multiplexed paired-end sequencing were prepared from 500 ng total-RNA using the Ribo-Zero Magnetic Gold technology (Epicentre, an Illumina company) for depletion of rRNA followed by library preparation using the ScriptSeq v2 technology (Epicentre). The RNA-Seq libraries were combined into 2 nM pooled stocks, denatured and diluted to 10 pM with pre-chilled hybridization buffer and loaded into TruSeq PE v3 flowcells on an Illumina cBot followed by indexed paired-end sequencing (101 + 7 + 101 bp) on a Illumina HiSeq 2000 using TruSeq SBS Kit v3 chemistry (Illumina). Paired de-multiplexed fastq files were generated using CASAVA software (Illumina) and processed using tools from CLC Bio (QIAGEN). Fastq files were trimmed for stretches of adapter sequences, joined into a single read if possible followed by quality trimming using commands from the CLC Assembly Cell. Processed fastq files were then imported into the CLC Genomics Workbench (QIAGEN) and mapped against gene regions and transcripts annotated by Human NCBI REFSEQ October 30, 2012. Gene-wise matrices of “total exon reads” counts were exported from the CLC Genomics Workbench for exploration and statistical analysis in the R computing environment (version 3.0.0 for Windows) using the R package Empirical analysis of Digital Gene Expression data in R (edgeR, version 3.2.3) facilitating identification of differentially affected genes between healthy human subjects and severely insulin resistant patients with T2D^19^,^20^,^21^,^22^,^23^. The differentially regulated gene transcripts were annotated to biological function and pathways using Ingenuity Pathway Analysis software^24^. The analysis was performed in November 2015 and the results were filtered for skeletal muscle related functions in humans or mice or rats. Supervised hierarchical cluster analysis and heat map was generated using GeneSpring GX11.5 software (Agilent Technologies, CA, USA).

### Protein extraction and western blot analysis

Frozen muscle tissue were homogenized in ice-cold lysis buffer (50 mM HEPES, 137 mM NaCl, 10 mM Na_4_P_2_O_7_, 10 mM NaF, 1 mM MgCl_2_, 2 mM EDTA, 1% NP-40, 10% glycerol (vol/vol), 1 mM CaCl_2_, 2 mM Na_3_VO_4_, 100 mM AEBSF [4-(2-aminoethyl) benzenesulfonyl fluoride], hydrochloride, pH 7.4) using a Precellys homogenizer (Bertin Technologies, France). Insoluble materials were removed by centrifugation at 14,000 × *g* for 20 minutes at 4 °C. Protein concentration of the supernatant was determined using a Bradford assay (BioRad, CA, USA). Samples were adjusted to equal concentrations with milli-Q water and denatured by mixing with 4x Laemmli’s buffer and heating at 95 °C for 5 minutes. Equal amounts of protein were separated by SDS-PAGE using the BioRad Criterion system, and proteins were electroblotted onto PVDF membranes (BioRad). Control for equal loading was performed using the Stain-Free technology that allows visualization of total protein amount loaded to each lane and has been shown to be superior to beta-actin and GAPDH in human skeletal muscle^25^,^26^. Membranes were blocked for 2 hours in a 2% bovine serum albumin solution (Sigma-Aldrich, MO, USA) and incubated overnight with primary antibodies (antibodies are specified in the Electronic Supplementary Material Table S1). After incubation in primary antibodies the membranes were incubated 1 hour with HRP-conjugated secondary antibodies. Proteins were visualized by chemiluminiscence (Pierce Supersignal West Dura, Thermo Scientific, IL, USA) and quantified with ChemiDoc^TM^ MP imaging system (BioRad). Protein Plus Precision All Blue standards were used as marker of molecular weight (BioRad).

### Statistics

Normal distribution and equal variance was assumed after graphical inspection of QQ-plots and Bland-Altman plots. Comparisons between groups were performed by Student’s t-test. Data were analyzed in SigmaPlot (SigmaPlot 11.0, Sysstat Software, CA, USA) and is presented as mean ± SEM. Data based on RNA-sequencing was analyzed and corrected for multiple testing as described in the methods (RNA sequencing). Heatmap was creased in GeneSpring 13.1.1 (Agilent) using median scaled log2 transformed RNA expression data with one added to values before log2 transformation.

## Results

### Subject characteristics

Characteristics of the included subjects have been published in details previously^3^,^18^. In short, age and BMI were 59 ± 2 years and 28 ± 1.5 kg/m^2^ in the control group and 58 ± 2 years and 35.7 ± 2.1 kg/m^2^ in the diabetes group. BMI tended to be elevated in the T2D patients (p = 0.05). Fasting plasma glucose were 5.3 ± 0.2 mmol/l and 7.9 ± 0.4 mmol/l in controls and T2D patients, respectively (p < 0.001). Insulin levels were 68 ± 8 pmol/l in the controls and 350 ± 46 pmol/l in T2D patients (p < 0.001), and C-peptide were 809 ± 100 pmol/l in the controls and 641 ± 148 pmol/l in the T2D patients (p < 0.001). The mean duration of diabetes was 17.3 ± 4.1 years at the time of inclusion.

### Gene transcription profile

**Figure 1:**
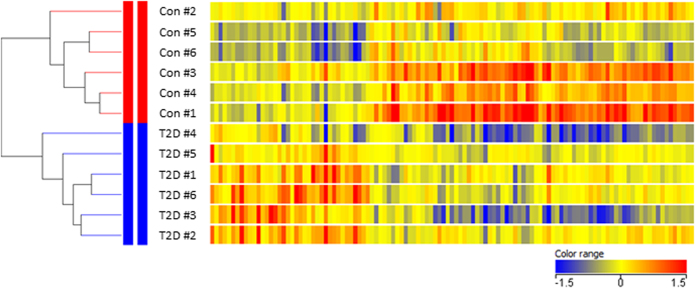
Heat map of the 117 genes differentially expressed genes between controls and type 2 diabetic subjects. Fold change in gene expression is color coded: red: expression higher than the median of all samples; blue: expression lower than the median of all samples; yellow: median expression. Supervised hierarchical clustering was performed vertically in samples and horizontally in genes. As illustrated by the dendrogram, the analysis identified two distinct clusters separating healthy subjects from patients with type 2 diabetes. The length of the lines indicates the degree of separation between the clusters.

Using whole transcriptome, strand-specific RNA-sequencing, we identified 1,732 gene transcripts that were differently expressed in the two groups with an uncorrected p-value < 0.05 and 117 genes that were differently expressed following correction for multiple testing (FDR < 0.05, Electronic Supplementary Material Table S2). Supervised hierarchical cluster analysis illustrated on the heat map in Fig. 1 separated healthy subjects and T2D patients into two distinct clusters. All differently expressed gene transcripts were further analyzed using Ingenuity Pathway Analysis software. The result of this analysis is summarized in Table 1 and shows that the most pronounced differences are associated with morphologic abnormalities, altered substrate metabolism, and mitochondrial dysfunction. Gene transcripts annotated with oxidation of fatty acids had a z-score smaller than -2. No other function achieved a z-score greater than 2 or smaller than -2. Gene transcripts encoding structural and functional genes such as myosin heavy chain isoforms (*MYH1, MYH2, MYH4*), and laminins (*LAMB3*) were highly upregulated in skeletal muscle from T2D patients, whereas genes encoding proteins involved in mitochondrial biogenesis (*PPARGC1A*) and respiration (*COX6A2*) were suppressed. Moreover, the expression of gene transcripts encoding proteins involved in insulin signal transduction, such as insulin receptor (*INSR*), insulin receptor substrate (*IRS2*), and protein kinase AKT (*AKT1 and AKT2*) were suppressed in patients with T2D. We also observed increased expression of embryonic and perinatal forms of myosin heavy chain (*MYH3* and *MYH8*) in muscle from T2D patients. The complete gene expression data can be found as a text file in the Electronic Supplementary Material (genes_exonReads_Matrix).

### Autophagy-related gene and protein expression is repressed in skeletal muscle from T2D patients

**Figure 2:**
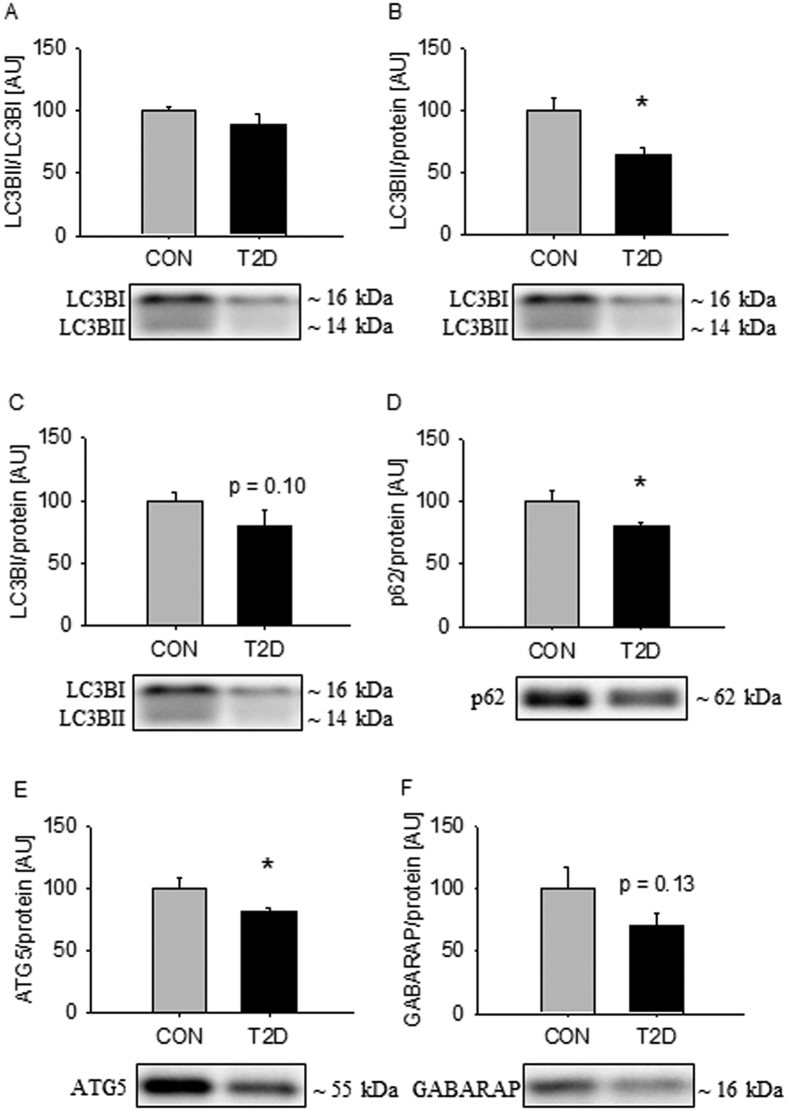
Autophagy-related protein expression is repressed in skeletal muscle from T2D patients. The ratio of LC3BII to LC3BI was equal in the two groups (**A**). Separate analysis of LC3BII and LC3BI showed that LC3BII was decreased in the diabetics (**B**), but the difference in LC3BI did not reach statistical significance (**C**). p62 and ATG5 were decreased in the patients (**D**,**E**). GABARAP was decreased in the patients, but the difference did not reach statistical significance (**F**). Values are means ± SEM. *Indicate difference in the mean values based on Student’s t-test. Representative western bots are shown below the graphs. Based on the applied molecular standards, approximated molecular weights are indicated on the right.

Autophagy is a catabolic process involved in maintenance of cellular homeostasis by delivering cytoplasmic constituents to the lysosomes for degradation^27^. As the gene expression profile revealed major differences related to mitochondrial dysfunction and altered morphology, we hypothesized that regulation of autophagy would be affected in the patients. During autophagy Microtubule Associated Protein 1 Light Chain Beta (LC3BI) and GABA(A) Receptor-Associated protein (GABARAPI) are converted to LC3BII and GABARAPII through lipidation by an ubiquitin-like system involving Autophagy-related gene (ATG) 5^27^. During this process LC3BII and GABARAPII are incorporated into the growing autophagosomal membrane where they functions as binding proteins for adapter proteins such as Sequestosome (p62) that recruits cellular components for degradation. We did not observe any difference in the ratio of LC3BII to LC3BI in the two groups (Fig. 2A). Separate analysis of LC3BI and LC3BII demonstrated that LC3BII was suppressed in T2D patients (Fig. 2B), but although LC3BI was lower in T2D patients, this did not reach statistical significance (Fig. 2C, p = 0.10). Protein expression of p62 and ATG5 were suppressed in T2D patients (Fig. 2D,E). GABARAP protein expression was lower in T2D patients, but this did not reach statistical significance (Fig. 2F, p = 0.13). In the RNA-sequencing data we identified 5 autophagy-related gene transcripts that were differently expressed (*ATG14, GABARAPL1, RB1CC1*/*FIP200, WIPI1, and SQSTM1*/*p62*) and all of them were decreased in the patients (Supplementary Material Table S3).

### Autophagic regulation through FOXO3a tends to be repressed in skeletal muscle from T2D patients

**Figure 3:**
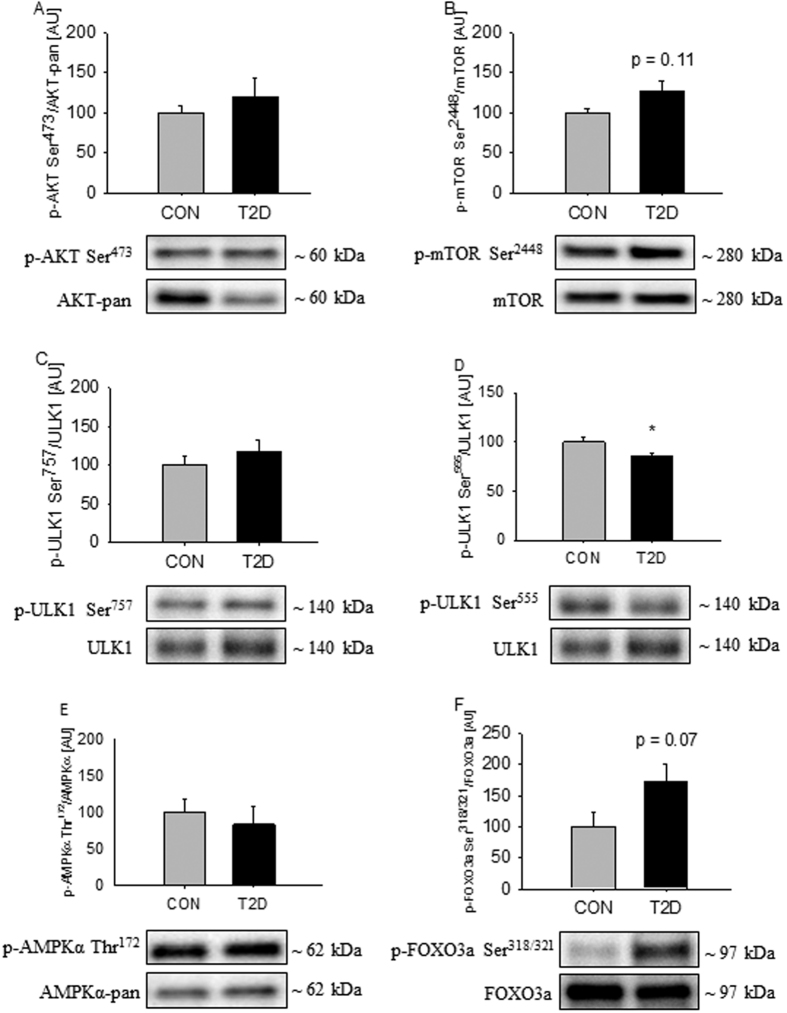
Autophagic signaling through ULK1 and Foxo3a are repressed in skeletal muscle from T2D patients. AKT phosphorylation at Ser^473^ was equal in the two groups (**A**). mTOR phosphorylation at Ser^2448^ was elevated in the patients, but the difference did not reach statistical significance (**B**). ULK1 phosphorylation at Ser^757^ was equal in the two groups (**C**), while ULK1 phosphorylation at Ser^555^ was decreased in patients with T2D (**D**). AMPKα phosphorylation at Thr^172^ was equal in the two groups (**E**). FOXO3a phosphorylation at Ser^321/318^ was increased in the patients, but the difference did not reach statistical significance (**F**). Values are means ± SEM. *Indicate difference in the mean values based on Student’s t-test. Representative western bots are shown below the graphs. Based on the applied molecular standards, approximated molecular weights are indicated on the right.

The insulin sensitive kinase AKT serves as a common upstream regulator of enzymes involved in transcriptional and non-transcriptional regulation of autophagy^27^. Forkhead box O3a (FOXO3a) plays a major role in transcriptional regulation of autophagy, while Unc-51 Like Protein Activating Kinase 1 (ULK1) plays a critical role in non-transcriptional regulation of autophagy by receiving inhibitory signals from the upstream kinase mammalian target of rapamycin complex 1 (mTORC1) and stimulatory signals from AMP activated protein kinase (AMPK)^28^,^29^. AKT-pan protein expression was decreased by ~25% in T2D patients, but this did not translate into a difference in AKT phosphorylation at Ser^473^ when expressed as a ratio of AKT-pan expression (Fig. 3A). Protein expression of mTOR was equal in the two groups, and although mTOR phosphorylation at Ser^2448^ was ~25% elevated in T2D patients, this did not reach statistical significance (Fig. 3B, p = 0.11). We did not observe any difference in ULK1 protein expression and phosphorylation at Ser^757^ (Fig. 3C), while phosphorylation of ULK1 at Ser^555^ was decreased in in T2D patients (Fig. 3D). We did not observe any difference in AMPKα protein expression or phosphorylation at Thr^172^ (Fig. 3E). FOXO3a was equally expressed in the two groups, and the phosphorylation of FOXO3a at Ser^318/321^ tended to be elevated by ~75% in T2D patients (Fig. 3F, p = 0.07).

### Signaling to protein synthesis is stimulated in skeletal muscle from T2D patients

**Figure 4:**
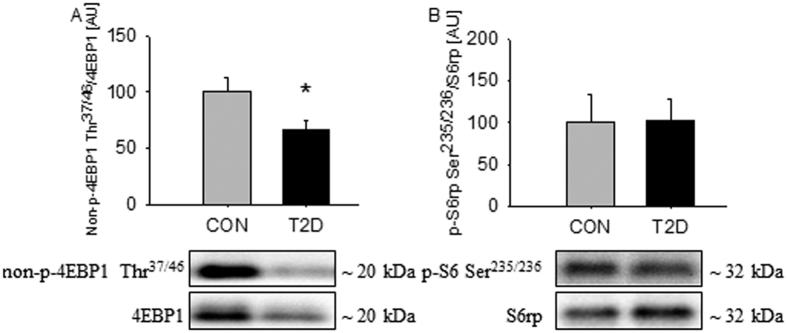
Signaling to protein synthesis is stimulated in skeletal muscle from T2D patients. 4EBP1 phosphorylation at Thr^37/46^ was elevated, in the patients as demonstrated by decreased non-p-4EBP1 (**A**). S6rp phosphorylation at Ser^235/236^ was equal in the two groups (**B**). Values are means ± SEM. *Indicate difference in the mean values based on Student’s t-test. Representative western bots are shown below the graphs. Based on the applied molecular standards, approximated molecular weights are indicated on the right.

Besides being involved in regulation of autophagy mTORC1 also plays a role in regulation of protein synthesis by regulation of downstream targets, such as Eukaryotic Translation Initiation Factor 4E Binding protein (4EBP1) and ribosomal protein S6 (S6rp)^30^. Protein expression of 4EBP1 was suppressed by ~50% in T2D patients, and phosphorylation of 4EBP1 at Thr^37/46^ was elevated, as demonstrated by ~40% decreased non-p-4EBP1 (Fig. 4A). No difference in protein expression of S6rp and phosphorylation at Ser^235/236^ was observed between the two groups (Fig. 4B).

### Expression of mitochondrial proteins are repressed in skeletal muscle from T2D patients

**Figure 5:**
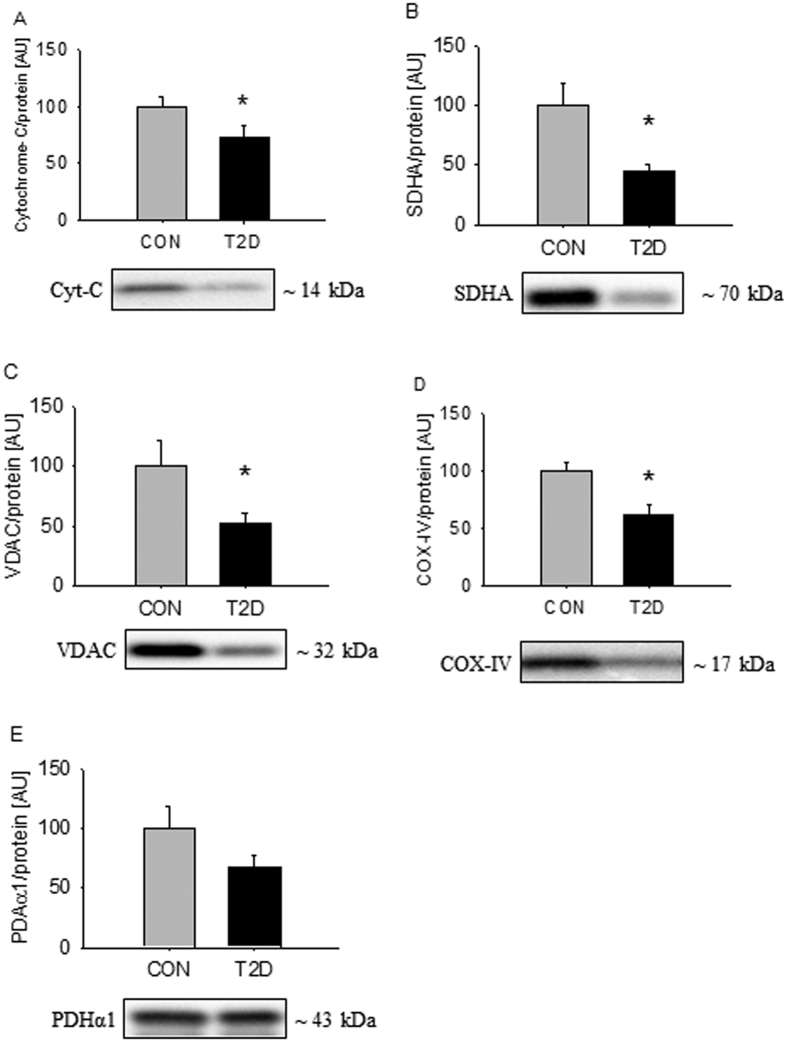
Expression of mitochondrial protein is repressed in skeletal muscle from T2D patients. Cyt-C, SDHA, VDAC, and COX-IV were suppressed in the patients (**A**–**D**), while the difference in PDHα1 did not reach statistical significance (**E**). Values are means ± SEM. *Indicate difference in the mean values based on Student’s t-test. Representative western bots are shown below the graphs. Based on the applied molecular standards, approximated molecular weights are indicated on the right.

Decreased mitochondrial content has been demonstrated in skeletal muscle from patients with T2D^7^. This is in good agreement with the mitochondrial dysfunction revealed in the present study by RNA-sequencing. To examine whether this is associated with decreased expression of mitochondrial proteins we examined the expression of Voltage Dependent Anion Channel (VDAC), Succinate Dehydrogenase alpha (SDHA), Pyrovate Dehydrogenase alpha 1 (PDHα1), Cytochrome C (Cyt-C), and Cytochrome C oxidase 4 (COX-IV). Protein expression of Cyt-C, SDHA, VDAC, and COX-IV were suppressed by 30–50% in T2D patients (Fig. 5A–D), while the difference in PDHα1 protein expression did not reach statistical significance (Fig. 5E).

## Discussion

In the present study, we used RNA-sequencing to demonstrate that skeletal muscle from severely insulin resistant T2D patients have an altered gene expression profile compared to healthy controls. 117 genes were differentially regulated and many of these genes were related to abnormal muscle morphology and mitochondrial dysfunction. These findings demonstrate that insulin resistant skeletal muscle in humans exhibit characteristics similar to those observed in autophagy-deficient skeletal muscle in mice. In accordance to this, we demonstrate that gene and protein expression of several autophagic components are suppressed in skeletal muscle from these patients. We also demonstrate that gene expression of embryonal and perinatal myosin heavy chains (*MYH3* and *MYH8*) are highly elevated in T2D. This is highly unusual and could indicate exaggerated stimulation of growth promoting pathways. The impaired muscle phenotype in T2D patients may therefore at least partly be a consequence of inadequate cellular maintenance through autophagy and excessive activity of growth promoting pathways. However, the data reported here are only associative and further studies are needed to examine the relation between autophagy, mitochondrial dysfunction, and exogenous treatment with insulin.

Comparison of gene transcription profiles obtained in previous investigations of patients with T2D^9^,^31^ with the gene transcription profile obtained in the present study reveal an overlap of less than 15%. Some of this discrepancy might be explained by the use of RNA-sequencing instead of microarrays. RNA-sequencing offers the advantage compared to microarrays that is does not require prior knowledge of transcript-specific probes, which enables detection of unknown and low abundant gene transcripts. However, much of the variation is likely explained by large differences in the investigated populations. Due to severe insulin resistance, the patients included in the present study had insulin and glucose infused at the time of biopsy sampling. Thus, we are not able to discriminate the effects of insulin stimulation, obesity, or altered substrate availability. The altered gene expression profile may therefore reflect the effects of both the disease and its treatment/complications. Nonetheless, the presented data provides new information on gene expression in an experimental setup that reflects the metabolic and hormonal environment these insulin resistant muscles are normally exposed to. Many of the differently regulated gene transcripts are in accordance with previous histological and metabolic findings. *MYH1* (myosin heavy chain IIX) and *MYH4* (myosin heavy chain IIB) were highly upregulated in the patients and *TNNT1* (slow skeletal muscle troponin T) was decreased, which corresponds well with decreased proportion of slow oxidative fibers observed in patients with T2D^31^. *PPARGC1A* and *COX6A2* were suppressed in the patients, which indicate that mitochondrial function is impaired, as the proteins encoded by these transcripts are involved in mitochondrial biogenesis and electron transport^32^. These observations are supported by the Ingenuity Pathway Analysis that demonstrated that the gene transcription profile is associated with mitochondrial dysfunction and altered muscle morphology. Impaired mitochondrial function is also supported by decreased expression of several mitochondrial proteins. Thus, RNA-sequencing enables us to generate data that reflect histological and metabolic observations in skeletal muscle from T2D patients, and reveal that many characteristics of skeletal muscle in the early stage T2D persist in the late stage of T2D.

Despite a ~5-fold difference in insulin levels at the time of biopsy sampling, we did not observe any difference in AKT phosphorylation at Ser^473^ or phosphorylation of the AKT substrate AS160 at Thr^642^. This impaired signaling to GLUT4 transport is in agreement with numerous observations in T2D patients^32^. However, the increased insulin levels were associated with increased 4EBP1 phosphorylation at Thr^37/46^ and a trend to increased mTOR phosphorylation. These data indicate that, despite severe reduction of insulin signaling to glucose uptake, other components of the insulin signaling cascade has retained some sensitivity. Thus, the growth-mediating effects of mTOR may be chronically stimulated in patients treated with high doses of insulin. This could contribute to direct muscle homeostasis into highly unusual states. In support of this, we observed that gene transcripts encoding embryonal and perinatal myosin heavy chains (*MYH3* and *MYH8*) were highly elevated in the patients. Increased expression of these genes has previously been observed in skeletal muscle from patients with spinal cord injuries, and is associated with metabolic inflexibility^33^. Reduced expression of *C10orf10* in the patients also supports that insulin has retained its ability to mediate intracellular signals, as expression of this gene is negatively regulated by insulin^34^. The mechanism behind this has been shown to depend on members of the FOXO family^35^, which is in good agreement with the inhibitory effects of insulin on these transcription factors and the observed trend to increased FOXO3a phosphorylation in the present study. Intact insulin action on growth-promoting pathways may therefore lead to unrestrained stimulation in patients treated with high doses of exogenous insulin, which probably leads to impaired muscle homeostasis and function.

Insulin is a potent inhibitor of autophagy and we speculated that the distorted gene expression profile could be worsened by insufficient cellular removal of dysfunctional mitochondria and proteins. In skeletal muscle from diabetic rats with high insulin levels the ratio of LC3BII to LC3BI is decreased, indicating that the number of autophagosomes is reduced^36^. In contrast, diabetic rats with low insulin levels have an increased ratio of LC3BII to LC3BI^36^. These findings suggest that insulin is responsible for suppressing muscular autophagy in T2D. In accordance to this, reduced ratio of LC3BII to LC3BI has been demonstrated during insulin stimulation in patients with T2D^17^. Our data showed that the expression of LC3BII was suppressed in T2D patients treated with high doses of insulin. mRNA expression of *LC3B* was equal in the two groups, and although not reaching statistical significance, protein expression of LC3BI tended to be lower in T2D patients. These findings indicate that the production of LC3B is unchanged or even suppressed in muscle from T2D patients, and autophagosome formation may therefore be impaired in skeletal muscle from these patients. *GABARAPL1* mRNA was also suppressed in the patients, which further suggests that the supply of building blocks for autophagosome formation is suppressed^27^. Our data also indicate that the recruitment of proteins to the autophagosome is inhibited, as mRNA and protein expression of p62/SQSTM1 were down-regulated in the patients. These data show that markers of autophagy is suppressed in skeletal muscle from patients with long standing T2D, which could be a consequence of prolonged exposure to highly elevated insulin levels.

ULK1 and the FOXO3a play major roles in regulation of autophagy and both are inhibited during insulin stimulation^29^,^37^. ULK1 is inhibited by mTORC1 through phosphorylation at Ser^757^ 29,38,39,40 and FOXO3a in inhibited by AKT through phosphorylation at Ser^318/321^ 41. Stimulation with insulin inhibits autophagy through mTORC1/ULK1in human skeletal muscle^16^,^42^,^43^. However, despite elevated insulin levels in T2D patients we did not observe increased ULK1 phosphorylation at Ser^757^ and mTOR phosphorylation at Ser^2448^. This could indicate insulin resistance and implies that another mechanism than mTORC1/ULK1 is responsible for suppressing autophagy. FOXO3a phosphorylation at Ser^318/321^ was elevated in skeletal muscle from T2D patients but the difference remained non-significant. However, the strong tendency could indicate that this transcription factor indeed was inactivated. To examine whether this was associated with transcriptional down-regulation of autophagy, we sampled autophagy-related gene transcripts from the RNA-sequencing dataset. Five autophagy-related gene transcripts (*ATG14, GABARAPL1, RB1CC1*/*FIP200, WIPI1, and SQSTM1*/*p62*) were down-regulated in skeletal muscle from T2D patients. The expression of *SQSTM1*/*p62* and *GABARAPL1* is known to be tightly controlled by FOXO3a, and these data therefore provide further evidence to suggest that the transcriptional activity of FOXO3a is inhibited^37^. These data indicate that insulin-induced transcriptional inhibition of FOXO3a could be involved in suppressing autophagy in skeletal muscle from severely insulin-resistant T2D patients. However, only markers of autophagy are reported in the present study, and further studies aimed at developing methods to measure autophagy flux are needed to confirm this hypothesis.

More than 30 autophagy-related genes encoding proteins of the autophagic core machinery have been discovered in mammalian cells^44^. We identified 33 autophagy-related gene transcripts by RNA sequencing in human skeletal muscle. Five of these were decreased in patients with T2D compared to healthy controls and further 3 tended to be decreased. We did not find evidence for global down-regulation of autophagy-related gene expression using the Ingenuity Pathways analysis. However, autophagy is a dynamic process that involves a high degree of non-transcriptional regulation. This limits the ability to detect regulation by bioinformatics analysis of a single muscle biopsy.

Autophagic flux cannot be determined in human skeletal muscle *in vivo* and this is a limitation for clinical studies. Consequently, the present study only contains data on autophagic markers. The observed decreased in LC3BII and p62 protein could be caused by reduced autophagosome formation and decreased flux through the autophagy-lysosomal system. Using specific lysosomal inhibitors in cultured cells demonstrates that protein levels of LC3BII do not *per se* reflect autophagy flux and that the amount of autophagy-related proteins varies among different stages of autophagy^44^,^45^,^46^. Our data does therefore not allow us to conclude that autophagic flux is decreased, but they clearly demonstrate that expression of several components of the autophagic machinery are decreased in skeletal muscle from patients with T2D.

In conclusion, skeletal muscle gene transcription profiles from severely insulin resistant patients with T2D show distinct dysregulation with major differences related to mitochondrial dysfunction and morphological abnormalities. Gene and protein expression of several autophagic markers are suppressed in skeletal muscle from patients with T2D, which may be a consequence of inhibited FOXO3a activity. We speculate that prolonged treatment with high doses of exogenous insulin in these patients could contribute to the accumulation of dysfunctional mitochondria and abnormal morphology and thereby generating a vicious cycle maintaining insulin resistance.

## Additional Information

**How to cite this article**: Møller, A. B. *et al*. Altered gene expression and repressed markers of autophagy in skeletal muscle of insulin resistant patients with type 2 diabetes. *Sci. Rep.* **7**, 43775; doi: 10.1038/srep43775 (2017).

**Publisher's note:** Springer Nature remains neutral with regard to jurisdictional claims in published maps and institutional affiliations.

## Supplementary Material

Supplementary Material

## Figures and Tables

**Table 1:** Ingenuity Pathway Analysis.

*Function annotation*	*p-value*		*Number of molecules*
Abnormal muscle morphology	1.03E-04		24
Mitochondrial quantity	4.80E-04		7
Muscle mass	8.22E-04		17
Muscle function	8.67E-04		23
Oxidation of fatty acids	2.92E-03		7
*Pathway annotation*			
Oxidative phosphorylation	2.71E-14		38
Mitochondrial dysfunction	2.71E-11		45
***Regulation***	***z-score***	***Direction***	***Number of molecules***
Oxidation of fatty acids	2.20	Down	7

*Upper part:* The top-five annotated functions sorted by p-values and number of associated genes. *Middle part:* The top-tow annotated pathways sorted by p-values and number. *Lower part:* Gene transcripts annotated with oxidation of fatty acids were significantly down-regulated in T2D patients.
