# Supplementary material for: Altered gene expression and repressed markers of autophagy in skeletal muscle of insulin resistant patients with type 2 diabetes
Source: Sci Rep. 2017 Mar 2;7:43775. doi: 10.1038/srep43775 (PMC5333153; doi:10.1038/srep43775)
Supplement: Supplementary Material [file srep43775-s1.pdf]

Supplementary Material

Title: Altered gene expression and repressed autophagy in skeletal muscle of insulin resistant patients with type 2 diabetes

Authors: Andreas Buch Møller[1], Ulla Kampmann[2], Jakob Hedegaard[3], Kasper Thorsen[3], Iver Nordentoft[3], Mikkel Holm Vendelbo[4], Niels Møller[2,5], Niels Jessen[1,3,6,*].

Table S1. Antibody specifications.

Primary antibodies		
Epitope	***Manufacturer***	***Catalog no.***
ULK1	Cell Signaling	4773
p-ULK1 Ser^{555}	Cell Signaling	5869
p-ULK1 Ser^{757}	Cell Signaling	6888
mTOR Ser^{2448}	Cell Signaling	2971
mTOR	Cell Signaling	2972
p62/SQSTM1	Abcam	ab56416
p-AKT Ser^{473}	Cell Signaling	9271
AKT-pan	Cell Signaling	3063
LC3B	Cell Signaling	3868
ATG5	Cell Signaling	12994
GABARAP	Cell Signaling	13733
p-FOXO3a Ser$^{318/321}$	Cell Signaling	9465
FOXO3a	Cell Signaling	3839
Non-p-4EBP1 Thr$^{37/46}$	Cell Signaling	4923
4EBP1	Cell Signaling	9644
p-S6rp Ser$^{235/236}$	Cell Signaling	2217
S6rp	Cell Signaling	4858
PDHα1	Cell Signaling	3205
SDHA	Cell Signaling	11998
COX-IV	Cell Signaling	4850
Cyt-C	Cell Signaling	4280
VDAC	Cell Signaling	4661
Secondary antibodies		
Epitope	***Manufacturer***	***Catalog no.***
Rabbit Ig	Santa Cruz	Sc-2054
Mouse Ig	Santa Cruz	Sc-2096
Streptavidin HRP	SouthernBiotech	7100-05

Specification of primary and secondary antibodies used for western blots, and primers used for RT-PCR.

16

Table S2. Gene transcripts differentially regulated in T2D patients versus healthy controls.

Gene	*Gene symbol*	*Log(Fold change)*	*Direction*	*p-value (FDR)*
Aminoadipate-Semialdehyde Synthase	*AASS*	0.96	Down	0.012
ATP-Binding Cassette Sub-Family A Member 5	*ABCA5*	0.77	Down	0.006
ATP-Binding Cassette Sub-Family C Member 5	*ABCC5*	0.83	Down	0.002
Acyl-CoA Synthetase Short-Chain Family Member 1	*ACSS1*	0.92	Down	0.031
Activin Receptor Type-1	*ACVR1*	0.79	Up	0.013
Aldo-Keto Reductase Family 1 Member C3	*AKR1C3*	1.54	Up	0.039
Annexin A3	*ANXA3*	1.21	Down	< *0.001*
Rho GTPase Activating Protein 28	*ARHGAP28*	1.07	Up	0.009
AT Rich Interactive Domain 5B	*ARID5B*	0.93	Down	0.033
Aryl Hydrocarbon Receptor Nuclear Translocator-Like	*ARNTL*	0.95	Up	0.026
B-Cell CLL/Lymphoma 6	*BCL6*	1.22	Down	0.005
Chromosome 10 Open Reading Frame 10	*C10orf10*	1.35	Down	< *0.001*
Calcium Binding Protein 39-Like	*CAB39L*	0.97	Up	0.030
CCAAT/Enhancer Binding Protein (C/EBP) Beta	*CEBPB*	1.29	Down	< *0.001*
CCAAT/Enhancer Binding Protein (C/EBP) Delta	*CEBPD*	2.25	Down	< *0.001*
Chromodomain Helicase DNA Binding Protein 3	*CHD3*	0.73	Up	0.012
Cholinergic Receptor Nicotinic Alpha 1	*CHRNA1*	1.26	Up	0.035
Collagen Type XIX Alpha 1	*COL19A1*	2.69	Up	0.001
Cysteine And Glycine-Rich Protein 3	*CSRP3*	1.33	Down	0.013
Cathepsin F	*CTSF*	1.07	Down	< *0.001*
Cysteine-Rich Angiogenic Inducer 61	*CYR61*	1.61	Up	0.012
Diaphanous-Related Formin 1	*DIAPH1*	0.75	Down	0.018
Disrupted In Renal Carcinoma 2	*DIRC2*	0.62	Down	0.014
Eukaryotic Translation Initiation Factor 4E Binding Protein 1	*EIF4EBP1*	1.30	Down	0.039
Elongation Factor. RNA Polymerase II. 2	*ELL2*	0.76	Down	0.027
Endothelial PAS Domain Protein 1	*EPAS1*	0.75	Down	0.042
Erb-B2 Receptor Tyrosine Kinase 3	*ERBB3*	2.51	Up	0.042
Family With Sequence Similarity 184 Member B	*FAM184B*	2.00	Up	< *0.001*
FYVE. RhoGEF And PH Domain Containing 4	*FGD4*	0.78	Down	0.019
Fat Storage-Inducing Transmembrane Protein 1	*FITM1*	0.73	Down	0.027
FK506 Binding Protein 5	*FKBP5*	2.14	Down	< *0.001*
Fibronectin Leucine Rich Transmembrane Protein 3	*FLRT3*	1.67	Down	< *0.001*
Flavin Containing Monooxygenase 2	*FMO2*	0.94	Down	0.030
Forkhead Box O1	*FOXO1*	0.86	Down	0.044
Forkhead Box O3	*FOXO3*	1.19	Down	< *0.001*
GRB2-Associated Binding Protein 1	*GAB1*	0.82	Down	0.027
GABA(A) Receptor-Associated Protein Like 1	*GABARAPL1*	0.93	Down	0.001
Glutamate-Ammonia Ligase	*GLUL*	1.44	Down	< *0.001*
Glycerol-3-Phosphate Dehydrogenase 1-Like	*GPD1L*	0.72	Down	0.008
G Protein-Coupled Receptor 116	*GPR116*	0.81	Down	0.004
General Transcription Factor IIB	*GTF2B*	0.76	Down	0.014
HMG-Box Transcription Factor 1	*HBP1*	0.62	Down	0.024

Hyperpolarization Activated Cyclic Nucleotide Gated Potassium Channel 1	*HCN1*	1.18	Up	0.018
Hexokinase 2	*HK2*	1.66	Down	0.012
Immediate Early Response 5	*IER5*	1.42	Down	0.039
Interferon. Alpha-Inducible Protein 27	*IFI27*	1.17	Down	0.010
Interleukin 17 Receptor D	*IL17RD*	1.14	Up	0.014
Insulin Receptor	*INSR*	0.70	Down	0.037
Insulin Receptor Substrate 2	*IRS2*	1.51	Down	0.001
Small Conductance Subfamily N Alpha. Member 3	*KCNN3*	1.01	Up	0.001
Kruppel-Like Factor 10	*KLF10*	1.06	Down	0.045
Kruppel-Like Factor 13	*KLF13*	0.94	Down	0.008
Kruppel-Like Factor 15	*KLF15*	2.16	Down	0.004
Kruppel-Like Factor 9	*KLF9*	1.13	Down	*< 0.001*
Kelch-Like Family Member 34	*KLHL34*	1.41	Down	0.007
Kyphoscoliosis Peptidase	*KY*	1.01	Up	0.018
Laminin Beta 3	*LAMB3*	2.06	Up	*< 0.001*
Lactate Dehydrogenase D	*LDHD*	0.94	Down	0.030
Leucine-Rich Repeat Containing G Protein-Coupled Receptor 5	*LGR5*	2.41	Down	*< 0.001*
Leiomodin 2	*LMOD2*	0.96	Down	0.004
Lipoprotein Lipase	*LPL*	1.76	Down	*< 0.001*
LIM Domain Containing Preferred Translocation Partner In Lipoma	*LPP*	0.74	Down	0.039
Leucine Rich Repeat Containing 2	*LRRC2*	0.82	Down	0.008
Muscleblind-Like Splicing Regulator 3	*MBNL3*	1.02	Up	0.046
Mitochondrial Pyruvate Carrier 1	*MPC1*	0.94	Down	0.013
Membrane Protein. Palmitoylated 7	*MPP7*	0.79	Down	0.018
Myostatin	*MSTN*	1.22	Up	0.011
Metallothionein 1E	*MT1E*	1.59	Down	0.050
Metallothionein 1X	*MT1X*	2.02	Down	*< 0.001*
Metallothionein 2A	*MT2A*	1.48	Down	0.012
Myosin Binding Protein H	*MYBPH*	2.37	Up	0.014
Myogenic Factor 6	*MYF6*	0.92	Down	0.038
Myosin Heavy Chain 1	*MYH1*	1.81	Up	0.004
Myosin Heavy Chain 3	*MYH3*	1.65	Up	0.048
Myosin Heavy Chain 4	*MYH4*	4.12	Up	*< 0.001*
Myosin Heavy Chain 8	*MYH8*	2.92	Up	0.010
Neuroblastoma Breakpoint Family Member 1	*NBPF1*	0.93	Up	0.029
Oxysterol Binding Protein-Like 9	*OSBPL9*	0.73	Down	0.042
Paternally Expressed 10	*PEG10*	1.11	Up	0.009
Period Circadian Clock 1	*PER1*	1.18	Down	0.012
6-Phosphofructo-2-Kinase/Fructose-2.6-Biphosphatase 2	*PFKFB2*	1.20	Down	0.050
Post-GPI Attachment To Proteins 1	*PGAP1*	0.60	Up	0.039
Peroxisome Proliferator-Activated Receptor Gamma Coactivator 1 Beta	*PPARGC1B*	0.81	Up	0.015
Protein Kinase. AMP-Activated Gamma 3 Non-Catalytic Subunit	*PRKAG3*	1.18	Up	0.008
Protein Tyrosine Phosphatase. Non-Receptor Type 3	*PTPN3*	0.73	Down	0.016
RNA Binding Motif Protein 20	*RBM20*	0.77	Up	0.012
Regulator Of Cell Cycle	*RGCC*	1.08	Down	0.010
Ribosomal Protein L39	*RPL39*	1.00	Down	0.049

Runt-Related Transcription Factor 1	*RUNX1*	1.48	Up	0.018
Runt-Related Transcription Factor 1	*S1PR1*	0.71	Down	0.025
Serum Deprivation Response	*SDPR*	0.81	Down	0.037
Sestrin 1	*SESN1*	1.25	Down	< *0.001*
Sphingomyelin Synthase 2	*SGMS2*	0.74	Up	0.013
Shisa Family Member 2	*SHISA2*	1.55	Up	0.006
Solute Carrier Family 16 Aromatic Amino Acid Transporter Member 10	*SLC16A10*	0.92	Up	0.014
Solute Carrier Family 19 (hiamine Transporter Member 2	*SLC19A2*	1.47	Down	0.007
Solute Carrier Family 25 Pyrimidine Nucleotide Carrier Member 33	*SLC25A33*	2.03	Down	< *0.001*
Solute Carrier Family 25 Member 34	*SLC25A34*	0.77	Up	0.034
Solute Carrier Family 38 Member 3	*SLC38A3*	1.39	Down	< *0.001*
Solute Carrier Family 43 Amino Acid System L Transporter Member 1	*SLC43A1*	1.06	Down	0.005
Sushi Nidogen And EGF-Like Domains 1	*SNED1*	1.03	Down	0.010
SplA/Ryanodine Receptor Domain And SOCS Box Containing 3	*SPSB3*	0.87	Down	0.037
Synovial Sarcoma X Breakpoint 2 Interacting Protein	*SSX2IP*	1.03	Up	0.018
TBC1 Domain Family Member 8	*TBC1D8*	1.25	Down	< *0.001*
Tet Methylcytosine Dioxygenase 1	*TET1*	0.72	Up	0.022
Transforming Growth Factor Beta 2	*TGFB2*	0.66	Up	0.022
Thrombospondin 4	*THBS4*	1.12	Up	0.008
Translocase of Inner Mitochondrial Membrane 44 Homolog	*TIMM44*	0.67	Down	0.050
Tumor Necrosis Factor Alpha-Induced Protein 3	*TNFAIP3*	0.88	Up	0.012
Troponin T Type 1	*TNNT1*	1.02	Down	0.034
TSC22 Domain Family Member 1	*TSC22D1*	0.96	Down	0.001
Tetraspanin 8	*TSPAN8*	1.18	Down	0.005
Ubiquitin-Conjugating Enzyme E2D 1	*UBE2D1*	0.79	Down	0.022
Uridine-Cytidine Kinase 1-Like 1	*UCKL1*	1.01	Down	0.001
Yippee-Like 3	*YPEL3*	0.94	Down	0.014
Zinc Finger And BTB Domain Containing 16	*ZBTB16*	1.15	Down	0.002
Zinc Finger Protein 772	*ZNF772*	0.88	Down	0.002

Genes in human skeletal muscle that are differentially expressed in patients with T2D with high insulin requirements compared with healthy human counterparts.

Table S3. Autophagy-related genetranscripts in T2D patients versus healthy controls.

Gene	*Gene symbol*	*Log(Fold change)*	*Direction*	*p-value (FDR)*	*p-value*
Autophagy-related 2A	ATG2A	0.15	Down	0.669	0.314
Autophagy-related 2B	ATG2B	0.18	Down	0.749	0.461
Autophagy-related 3	ATG3	0.12	Down	0.730	0.425
Autophagy-related 4A	ATG4A	0.03	Up	0.975	0.888
Autophagy-related 4B	ATG4B	0.14	Up	0.716	0.399
Autophagy-related 4C	ATG4C	0.22	Up	0.64 9	0.267
Autophagy-related 4D	ATG4D	0.32	Down	0.629	0.191
Autophagy-related 5	ATG5	0.05	Up	0.689	0.805
Autophagy-related 7	ATG7	0.14	Up	0.936	0.350
Autophagy-related 9A	ATG9A	0.28	Up	0.609	0.099
Autophagy-related 9B	ATG9B	1.72	Down	0.649	0.264
Autophagy-related 10	ATG10	0.07	Up	0.908	0.751
Autophagy-related 12	ATG12	0.12	Down	0.740	0.443
Autophagy-related 13	ATG13	0.31	Down	0.599	0.082
Autophagy-related 14	*ATG14*	*0.36*	*Down*	*0.497*	*0.026*
Autophagy-related 16 like-1	ATG16L1	0.18	Down	0.642	0.234
Autophagy-related 16 like-2	ATG16L2	0.30	Up	0.747	0.456
Autophagy related 101	C12orF44/ATG101	0.30	Up	0.636	0.214
Beclin1	BECN1	0.15	Down	0.736	0.436
RB1-Inducible Coiled-Coil 1	*RB1CC1/FIP200*	*0.42*	*Down*	*0.402*	*0.014*
GABA(A) Receptor-Associated Protein	GABARAP	0.22	Down	0.713	0.395
GABA(A) Receptor-Associated Protein-Like 1	*GABARAPL1*	*0.93*	*Down*	*0.001*	*< 0.001*
GABA(A) Receptor-Associated Protein-Like 2	GABARAPL2	0.17	Down	0.755	0.469
Microtubule-Associated Protein 1 Light Chain 3 Alpha	MAP1LC3A	0.46	Down	0.634	0.208
Microtubule-Associated Protein 1 Light Chain 3 Beta	MAP1LC3B	0.80	Down	0.888	0.376
Microtubule-Associated Protein 1 Light Chain 3 Beta 2	MAP1LC3B2	0.10	Up	0.704	0.710
Microtubule-Associated Protein 1 Light Chain 3 Gamma	MAP1LC3C	0.14	Up	0.992	0.926
Sequestosome 1	*SQSTM1/p62*	*1.42*	*Down*	*0.544*	*0.043*
Unc-51 Like Autophagy Activating Kinase 1	ULK1	0.36	Down	0.629	0.183
Unc-51 Like Autophagy Activating Kinase 2	ULK2	0.29	Down	0.594	0.077
Phosphoinositide-3-Kinase Regulatory Subunit 4	VSP15/PIK3R4	0.17	Down	0.651	0.274
Phosphatidylinositol 3-Kinase Catalytic Subunit Type 3	VSP34/PIK3C3	0.03	Down	0.943	0.822
WD Repeat Domain, Phosphoinositide Interacting 1	*WIPI1*	*0.62*	*Down*	*0.085*	*< 0.001*
WD Repeat Domain, Phosphoinositide Interacting 2	WIPI2	0.27	Down	0.601	0.085

Autophagy-related gene transcripts in skeletal muscle from patients with T2D versus healthy controls.

24

gene	RES-05	RES-02	RES-04	RES-07	RES-06	RES-01
	CON-17	CON-15	CON-16	CON-20	CON-18	CON-21
61E3.4	244	1014	223	631	416	570
	763	1223	311	310	629	244
A1BG	3	2	1	0	1	1
	1	4	4	0	1	0
A1CF	46	1	30	3	15	4
	1	4	26	0	24	0
A2M	481	3259	362	1651	1137	1386
	2798	3089	1595	1027	1789	568
A2ML1	59	0	24	0	9	2
	2	0	37	0	20	0
A4GALT	14	35	12	18	7	19
	16	27	22	3	14	9
A4GNT	11	0	10	0	1	0
	0	0	4	0	0	0
AAAS	38	115	24	77	57	77
	94	155	59	42	77	21
AACS	41	48	17	22	22	40
	53	52	34	9	30	14
AADAC	16	0	9	0	4	0
	0	0	2	0	3	0
AADACL2	12	0	11	0	2	0
	0	0	3	0	2	0
AADACL3	17	0	11	0	2	2
	0	1	9	0	10	0
AADACL4	8	0	1	0	1	0
	0	0	0	0	3	0
AADAT	37	34	17	33	15	36
	33	58	28	21	32	4
AAED1	11	43	9	56	20	61
	63	63	32	10	26	11
AAGAB	91	512	88	290	144	419
	269	487	199	105	202	72
AAK1	271	795	240	679	311	514
	389	658	269	168	335	148
AAMDC	190	662	170	780	366	756
	1084	1361	442	222	568	88
AAMP	52	223	52	142	79	152
	213	273	111	71	147	34
AANAT	6	0	5	0	1	1
	0	0	5	0	3	0
AAR2	31	68	16	53	40	60
	64	99	43	21	46	25
AARD	10	0	1	0	0	1
	0	0	1	0	0	0
AARS	141	656	137	413	305	495
	419	486	199	181	333	117
AARS2	52	91	36	52	34	69
	43	118	47	16	64	20
AARSD1	12	101	21	101	41	77
	101	95	61	37	65	18
AASDH	46	174	55	131	89	106
	120	206	107	54	105	28
AASDHPPT	111	592	119	502	261	418
	478	629	304	176	353	120
AASS	73	350	73	177	120	144
	271	766	244	106	363	91

AATF	47	116	39	93	61	108
	96	161	89	50	71	29
AATK	14	5	8	4	3	3
	1	7	8	0	8	4
ABAT	43	35	30	20	13	29
	9	19	25	3	42	5
ABCA1	379	738	333	591	592	1024
	976	2169	931	502	786	330
ABCA10	227	1004	233	1215	382	597
	790	1484	502	363	514	167
ABCA12	93	2	58	0	18	0
	0	0	70	1	28	0
ABCA13	133	6	89	2	31	0
	2	0	71	0	48	2
ABCA2	48	115	47	67	51	67
	76	90	51	36	54	29
ABCA3	36	64	38	56	32	80
	54	77	53	32	55	19
ABCA4	71	3	46	0	20	0
	0	1	39	0	21	0
ABCA5	651	2852	758	2211	1286	2208
	3262	6025	2333	1242	2753	905
ABCA6	219	732	226	766	415	598
	568	1129	362	287	454	180
ABCA7	39	69	40	45	16	55
	26	60	32	11	22	7
ABCA8	275	1140	387	1562	594	982
	1003	1444	579	399	731	229
ABCA9	236	744	230	854	407	658
	611	1351	408	302	579	166
ABCB1	54	27	25	10	21	11
	6	23	26	4	23	4
ABCB10	56	233	55	209	121	174
	239	262	107	82	152	35
ABCB11	48	0	31	1	14	1
	0	0	21	0	14	0
ABCB4	93	314	108	264	173	221
	236	338	218	125	302	54
ABCB5	44	2	41	16	19	1
	0	1	28	0	19	0
ABCB6	69	230	49	164	99	166
	111	297	121	54	103	30
ABCB7	96	377	97	269	172	235
	355	464	235	138	247	77
ABCB8	23	54	19	34	32	36
	48	72	24	8	32	9
ABCB9	17	5	4	2	3	4
	4	4	6	0	3	3
ABCC1	97	345	59	254	121	223
	276	397	193	129	174	85
ABCC10	37	71	27	34	23	41
	46	62	54	19	45	9
ABCC11	45	1	33	1	8	1
	1	0	17	0	8	0
ABCC12	43	7	28	3	13	5
	17	7	27	0	10	1
ABCC2	72	39	49	28	56	75
	26	65	38	13	30	10

ABCC3	29	5	15	1	6	2
	0	2	19	1	13	1
ABCC4	124	221	76	212	99	178
	190	204	179	157	245	86
ABCC5	375	900	296	664	444	702
	1105	2126	640	677	910	399
ABCC6	44	86	24	42	74	104
	45	16	42	20	29	21
ABCC8	42	36	42	16	7	31
	24	0	27	3	24	5
ABCC9	815	3873	1016	3079	1734	2876
	2713	3690	1778	1110	2359	729
ABCD1	23	61	7	45	29	39
	53	64	29	15	51	14
ABCD2	35	21	20	16	20	18
	8	9	27	3	20	10
ABCD3	121	731	138	522	371	510
	624	792	313	227	423	144
ABCD4	45	152	42	88	76	97
	119	164	81	51	88	28
ABCE1	128	503	86	358	257	331
	380	492	220	171	291	103
ABCF1	123	653	131	373	328	483
	431	646	210	230	319	117
ABCF2	146	595	108	293	303	428
	356	569	214	147	265	88
ABCF3	73	317	75	237	168	250
	267	340	171	116	189	61
ABCG1	61	112	48	100	86	78
	128	261	114	61	140	73
ABCG2	38	19	18	5	9	8
	16	11	38	3	20	3
ABCG4	19	1	13	1	1	0
	0	0	7	0	2	0
ABCG5	10	0	3	0	1	0
	0	0	10	0	4	0
ABCG8	8	0	3	0	6	0
	0	0	7	0	5	1
ABHD1	9	16	8	9	8	15
	15	24	10	10	15	3
ABHD10	89	231	70	234	154	252
	282	447	194	92	261	52
ABHD11	13	35	7	15	12	16
	30	52	18	7	13	6
ABHD12	30	43	18	26	23	33
	28	57	39	7	35	18
ABHD12B	15	1	8	1	5	0
	0	1	6	0	4	0
ABHD13	82	283	64	241	136	163
	203	327	150	89	183	59
ABHD14A	7	40	4	19	22	29
	32	52	17	11	16	10
ABHD14B	20	75	18	38	37	65
	91	80	26	16	38	21
ABHD15	19	20	12	29	12	21
	18	13	14	5	7	6
ABHD16A	52	118	30	78	53	59
	133	218	107	55	91	28

ABHD16B	0	0	0	0	0	3
	0	0	1	2	0	1
ABHD2	106	463	102	315	240	306
	250	317	182	117	181	71
ABHD3	43	128	86	86	90	139
	118	221	85	36	90	26
ABHD4	38	114	24	54	27	59
	80	98	50	21	39	18
ABHD5	81	330	83	269	161	260
	275	351	188	95	217	65
ABHD6	19	48	16	49	21	35
	58	42	34	15	25	12
ABHD8	9	12	7	5	7	9
	11	21	9	4	16	4
ABI1	79	347	105	280	183	262
	308	367	200	86	200	63
ABI2	85	290	69	236	124	178
	214	302	141	61	145	47
ABI3	7	24	9	12	5	5
	19	17	10	4	7	3
ABI3BP	78	217	79	206	106	128
	86	209	109	41	88	52
ABL1	61	184	48	139	112	132
	127	175	67	59	96	41
ABL2	141	349	110	232	142	205
	189	303	172	97	176	52
ABLIM1	302	1684	400	786	449	1023
	697	1543	639	343	516	371
ABLIM2	156	684	149	548	300	524
	445	640	287	257	354	143
ABLIM3	163	554	67	352	216	358
	434	480	256	167	245	89
ABO	18	5	2	3	7	5
	8	3	13	1	4	0
ABP1	7	0	3	0	1	2
	0	0	5	0	1	0
ABR	89	217	80	112	127	136
	137	236	64	90	118	68
ABRA	195	1432	223	1268	899	1035
	1349	1534	425	732	1348	184
ABRACL	3	8	1	4	5	7
	13	28	4	2	4	0
ABT1	24	93	22	52	45	57
	97	103	59	33	60	10
ABTB1	15	23	11	33	19	14
	30	49	21	5	25	9
ABTB2	48	113	29	83	45	57
	81	102	61	28	41	13
ACAA1	31	166	31	116	55	90
	165	146	69	24	78	15
ACAA2	137	807	105	500	274	376
	491	878	279	155	351	100
ACACA	235	508	186	369	302	411
	378	575	279	174	355	136
ACACB	376	1964	324	1165	1159	1324
	1427	1747	614	680	822	431
ACAD10	67	150	40	76	83	91
	111	144	92	55	97	25

ACAD11	69	269	77	178	123	160
	200	347	167	89	190	45
ACAD8	50	206	24	149	93	155
	174	265	126	60	147	37
ACAD9	68	352	80	227	178	252
	212	324	123	121	168	61
ACADL	34	123	30	90	63	93
	122	226	83	47	76	18
ACADM	797	3383	620	2263	2020	1498
	3576	3658	1258	1240	2300	544
ACADS	53	202	44	173	93	98
	151	297	89	41	128	42
ACADSB	257	1082	209	783	423	654
	1139	1247	458	245	667	168
ACADVL	1190	4925	675	3274	2361	2932
	3961	8065	2020	1360	2989	750
ACAN	35	2	23	3	6	0
	0	1	17	0	12	4
ACAP1	23	12	11	3	6	4
	2	11	8	2	9	2
ACAP2	86	440	123	394	221	388
	312	449	208	126	251	76
ACAP3	38	136	36	103	59	82
	60	162	64	36	75	26
ACAT1	624	3230	501	2404	1484	1884
	3443	5907	1875	991	2715	675
ACAT2	23	37	12	31	15	21
	40	49	26	10	21	9
ACBD3	109	486	110	323	212	295
	356	421	210	155	279	70
ACBD4	15	18	9	17	9	11
	9	28	22	4	19	9
ACBD5	102	568	72	379	232	344
	437	520	228	146	301	86
ACBD6	42	173	52	133	68	146
	115	183	78	61	92	28
ACBD7	30	0	7	2	5	1
	3	7	13	1	8	0
ACCS	16	41	18	32	11	28
	17	43	35	14	45	9
ACCSL	19	0	11	1	3	0
	0	1	12	0	7	0
ACD	18	11	5	10	9	7
	8	18	21	8	11	3
ACE	44	137	16	80	60	70
	120	103	55	40	74	37
ACE2	42	0	10	3	7	2
	2	8	18	2	10	1
ACER1	5	0	4	0	1	0
	0	0	1	0	0	0
ACER2	18	30	7	23	18	21
	17	25	25	8	20	5
ACER3	49	46	27	35	30	34
	30	46	47	19	32	13
ACHE	63	345	61	265	149	230
	309	371	83	101	147	53
ACIN1	155	647	171	341	384	491
	515	651	224	249	388	122

ACLY	65	201	45	146	93	143
	101	164	73	48	95	52
ACMSD	14	0	8	0	1	1
	0	0	5	0	7	0
ACN9	16	86	16	73	39	84
	82	111	40	23	59	16
ACO1	89	473	80	333	208	303
	319	473	228	116	265	75
ACO2	466	2526	301	1417	1085	1254
	2697	2451	976	845	1425	411
ACOT1	31	95	16	127	45	69
	233	157	51	11	80	2
ACOT11	116	244	62	38	129	46
	99	219	108	31	152	39
ACOT12	26	0	12	0	5	3
	0	0	11	0	7	0
ACOT13	127	525	103	324	231	371
	510	649	317	143	303	86
ACOT2	79	278	50	232	110	216
	253	300	121	81	140	49
ACOT4	4	3	4	3	4	0
	1	3	2	0	5	0
ACOT6	7	2	3	0	8	2
	2	2	6	0	2	0
ACOT7	16	6	14	2	5	4
	5	4	9	2	8	3
ACOT8	23	74	13	44	30	48
	46	109	29	19	38	10
ACOT9	85	240	60	135	136	161
	157	361	137	102	115	64
ACOX1	278	1141	195	811	632	636
	821	1440	565	359	746	261
ACOX2	30	50	12	42	35	16
	72	42	29	20	24	10
ACOX3	25	90	29	41	40	66
	79	92	46	29	35	19
ACOXL	21	1	19	0	3	0
	0	0	16	0	7	0
ACP1	171	772	150	647	287	574
	782	916	363	201	466	138
ACP2	18	34	12	41	24	46
	43	55	31	20	31	11
ACP5	8	2	1	2	3	5
	0	4	4	0	2	1
ACP6	27	70	14	51	19	67
	54	48	19	19	43	8
ACPL2	36	26	30	32	30	40
	24	28	32	9	35	8
ACPP	25	4	22	1	6	4
	5	6	15	1	7	0
ACPT	4	0	5	0	0	0
	0	0	1	0	0	0
ACR	8	1	1	4	1	0
	1	0	4	0	2	0
ACRBP	6	0	1	1	3	1
	2	1	3	0	2	0
ACRC	52	89	42	69	52	119
	49	96	46	32	21	9

ACRV1	9	0	1	0	1	0
	0	0	5	0	0	0
ACSBG1	16	2	7	0	3	0
	2	2	5	0	5	0
ACSBG2	21	0	16	0	3	0
	0	0	4	0	1	0
ACSF2	45	155	37	105	113	158
	130	177	88	55	85	23
ACSF3	20	66	18	33	30	27
	65	70	45	20	37	19
ACSL1	832	4248	495	2082	2506	2599
	3424	6177	1559	1170	2425	750
ACSL3	266	1356	287	783	752	1159
	786	1479	580	400	848	207
ACSL4	104	468	116	328	217	330
	374	384	209	119	246	84
ACSL5	39	40	27	41	20	35
	49	50	45	19	44	7
ACSL6	72	112	68	108	38	97
	68	209	85	32	115	23
ACSM1	16	2	19	4	3	8
	4	2	8	1	6	0
ACSM2A	23	0	18	0	4	0
	0	1	17	0	13	0
ACSM2B	34	1	23	0	5	1
	1	0	17	0	9	0
ACSM3	21	10	19	7	12	10
	8	30	16	3	10	3
ACSM4	18	0	13	0	4	0
	0	0	6	0	5	0
ACSM5	48	64	39	156	30	123
	170	78	151	35	125	32
ACSS1	62	109	33	78	98	58
	207	262	124	57	150	61
ACSS2	174	705	157	379	428	474
	498	922	352	204	515	134
ACSS3	91	266	67	252	100	168
	263	287	227	85	221	49
ACTA1	37683	258832	27317	164454	89754	189591
	388543	222691	98692	107314	143009	49308
ACTA2	77	425	66	245	119	113
	190	350	110	111	159	228
ACTB	336	2474	405	1777	1154	1769
	1809	2425	946	647	1106	416
ACTBL2	12	5	6	1	4	0
	4	1	2	0	3	0
ACTC1	52	267	378	63	40	148
	82	228	102	85	73	44
ACTG1	147	954	215	876	550	775
	942	1394	519	300	543	248
ACTG2	9	8	7	3	3	0
	9	6	4	1	4	39
ACTL10	3	0	2	0	4	1
	5	2	2	0	1	0
ACTL6A	49	127	43	77	62	72
	87	181	57	42	87	23
ACTL6B	12	0	2	0	0	0
	0	0	2	0	4	0

ACTL7A	9	0	2	0	2	0
	0	0	6	0	3	0
ACTL7B	3	0	2	0	0	0
	0	0	1	0	0	0
ACTL8	7	0	3	0	2	0
	0	1	9	0	4	0
ACTL9	1	0	0	0	0	0
	0	0	1	0	0	0
ACTN1	49	209	60	103	48	102
	126	121	54	39	53	54
ACTN2	3696	34972	4456	15800	12968	16242
	25229	32716	9804	10251	14574	5985
ACTN4	102	539	83	197	153	245
	345	353	144	141	207	113
ACTR10	94	427	104	321	176	223
	431	593	226	144	290	81
ACTR1A	199	1130	162	716	453	840
	732	1042	475	266	531	163
ACTR1B	59	263	44	207	120	177
	260	337	117	93	176	50
ACTR2	153	1146	201	941	465	745
	1013	904	405	223	552	163
ACTR3	104	562	115	342	258	345
	416	655	247	151	301	106
ACTR3B	24	82	23	61	24	53
	65	89	32	21	43	19
ACTR3C	14	9	9	4	6	7
	2	1	13	4	9	4
ACTR5	15	63	24	48	21	36
	49	79	44	16	33	14
ACTR6	39	148	40	97	108	120
	137	228	81	47	88	24
ACTR8	59	231	53	146	95	142
	185	231	97	84	101	48
ACTRT1	9	0	2	0	2	0
	0	0	1	0	0	0
ACTRT2	1	0	1	1	0	0
	0	0	2	0	0	0
ACTRT3	8	3	4	6	7	9
	2	7	7	0	2	0
ACVR1	82	315	78	309	190	239
	222	148	112	56	105	29
ACVR1B	66	143	30	130	88	92
	108	138	69	44	74	18
ACVR1C	52	40	45	77	85	38
	35	43	46	13	57	18
ACVR2A	116	461	137	359	197	294
	320	451	186	125	183	71
ACVR2B	244	777	192	483	464	438
	567	1375	546	215	576	157
ACVRL1	66	163	45	97	75	130
	107	229	106	58	92	27
ACY1	22	31	8	26	9	21
	51	40	21	4	22	3
ACY3	7	0	5	0	0	0
	0	0	2	0	1	0
ACYP1	44	123	38	117	76	106
	114	193	97	35	100	18

ACYP2	84	279	46	404	153	340
	464	470	207	93	227	51
ADA	22	18	13	14	21	17
	13	36	19	8	11	1
ADAD1	16	0	6	0	2	0
	0	0	16	0	11	0
ADAD2	4	0	2	0	2	0
	0	0	0	0	2	0
ADAL	74	345	61	172	173	169
	220	329	188	108	208	78
ADAM10	77	337	84	253	140	220
	277	415	179	101	178	70
ADAM11	13	2	7	3	2	3
	3	0	3	0	7	0
ADAM12	61	6	31	0	19	3
	2	1	25	0	20	7
ADAM15	27	106	22	54	37	47
	67	110	39	25	41	24
ADAM17	98	268	110	220	148	261
	217	351	146	98	172	52
ADAM18	19	0	21	0	6	0
	0	0	19	0	11	0
ADAM19	67	230	51	176	108	167
	147	195	102	58	120	29
ADAM2	27	0	12	0	8	0
	0	0	12	0	8	0
ADAM20	35	88	45	123	56	115
	83	170	67	37	68	21
ADAM21	20	2	17	0	8	9
	1	6	13	0	6	0
ADAM22	88	131	79	213	119	125
	104	445	167	104	123	27
ADAM23	89	279	136	241	116	186
	189	257	104	92	145	35
ADAM28	48	15	32	17	16	8
	16	12	37	8	14	2
ADAM29	20	0	24	1	2	0
	0	0	8	0	1	0
ADAM30	12	0	12	0	2	0
	0	0	7	0	7	0
ADAM32	29	25	28	13	24	29
	16	24	25	4	33	3
ADAM33	18	70	10	105	32	40
	35	80	31	11	33	20
ADAM7	42	1	14	0	12	0
	0	0	20	0	8	0
ADAM8	8	10	11	1	3	3
	4	6	6	1	3	0
ADAM9	124	547	128	409	303	517
	364	682	230	197	313	120
ADAMDEC1	10	0	11	0	5	0
	0	0	12	0	2	0
ADAMTS1	56	431	157	325	123	249
	193	200	127	73	72	37
ADAMTS10	28	47	15	33	23	29
	22	35	25	8	29	9
ADAMTS12	60	73	31	54	37	23
	41	48	54	18	45	7

ADAMTS13	7	29	13	12	9	7
	10	26	23	4	22	4
ADAMTS14	21	1	20	3	2	1
	3	0	12	1	6	1
ADAMTS15	22	29	21	25	19	29
	28	30	22	10	19	8
ADAMTS16	33	19	26	13	20	12
	2	3	23	1	20	1
ADAMTS17	31	36	32	19	33	40
	30	22	38	18	31	8
ADAMTS18	57	2	21	0	9	5
	1	0	26	2	16	1
ADAMTS19	84	53	42	41	69	40
	81	162	92	29	144	22
ADAMTS2	30	37	32	44	25	20
	9	32	33	1	19	12
ADAMTS20	61	0	36	0	15	1
	2	1	51	0	22	0
ADAMTS3	52	12	28	14	15	9
	4	22	35	4	20	5
ADAMTS4	20	5	10	2	4	0
	6	1	13	2	4	2
ADAMTS5	64	154	44	179	114	169
	106	339	147	61	175	99
ADAMTS6	47	18	42	24	38	19
	26	37	49	6	35	8
ADAMTS7	14	16	12	8	4	4
	9	6	6	6	7	1
ADAMTS8	16	10	8	0	13	4
	8	3	6	3	10	2
ADAMTS9	184	714	227	317	323	335
	403	649	256	188	250	83
ADAMTSL1	42	10	33	22	16	6
	15	14	17	5	15	4
ADAMTSL2	26	37	23	42	15	33
	20	63	23	10	14	10
ADAMTSL3	91	143	63	131	116	95
	158	178	117	57	107	26
ADAMTSL4	35	67	37	41	37	36
	65	191	22	32	58	35
ADAMTSL5	25	48	11	27	19	20
	43	54	24	23	32	11
ADAP1	8	3	7	2	3	0
	0	6	2	0	2	0
ADAP2	13	34	19	32	18	15
	29	27	16	8	28	10
ADAR	171	679	141	450	288	365
	469	533	273	258	295	107
ADARB1	74	281	91	157	91	185
	208	288	138	73	116	58
ADARB2	21	0	12	2	10	0
	0	0	21	1	10	0
ADAT1	45	86	25	51	26	54
	55	48	41	11	41	8
ADAT2	44	55	29	47	20	35
	38	56	37	19	37	5
ADAT3	0	0	0	0	1	0
	0	1	0	0	0	0

ADC	6	11	6	5	4	3
	4	17	12	1	15	1
ADCK1	29	44	20	24	47	54
	78	81	37	19	21	8
ADCK2	22	35	19	21	20	15
	39	42	27	6	39	7
ADCK3	1527	5530	1180	5611	3886	3958
	6407	6889	2360	1892	4534	1164
ADCK4	22	23	13	19	10	19
	21	18	20	8	17	4
ADCK5	3	6	2	4	5	5
	6	13	6	4	9	0
ADCY1	81	246	119	235	107	209
	227	152	125	62	138	36
ADCY10	34	6	27	7	13	6
	3	13	21	3	15	1
ADCY2	282	1382	334	862	783	1106
	1410	1722	790	438	911	306
ADCY3	46	63	34	51	49	61
	45	104	50	20	38	17
ADCY4	32	111	31	53	39	41
	107	89	53	27	70	18
ADCY5	34	44	31	17	25	18
	22	54	22	13	20	10
ADCY6	67	138	45	78	64	135
	97	168	74	38	73	26
ADCY7	40	76	29	62	68	80
	43	72	59	32	44	21
ADCY8	44	1	15	1	8	0
	0	0	11	0	4	0
ADCY9	204	957	150	540	574	622
	535	1009	268	307	444	165
ADCYAP1	15	2	12	0	2	4
	1	5	14	0	4	0
ADCYAP1R1	30	19	21	9	18	3
	18	22	28	7	23	5
ADD1	260	1326	284	1002	716	1012
	1080	1431	662	484	823	314
ADD2	46	17	22	7	11	4
	6	8	27	7	15	2
ADD3	85	402	85	416	223	312
	221	505	199	125	224	105
ADGB	61	0	36	1	13	3
	0	0	34	0	25	0
ADH1A	9	4	2	1	6	2
	1	3	8	1	5	0
ADH1B	164	628	174	844	544	637
	686	1492	527	274	566	365
ADH1C	50	70	20	62	42	194
	62	256	65	19	37	22
ADH4	25	1	10	1	6	0
	1	2	11	1	10	0
ADH5	153	573	112	500	287	546
	712	891	393	182	359	118
ADH6	25	0	10	1	6	1
	0	0	12	0	6	0
ADH7	18	0	12	0	8	0
	0	1	15	0	8	0

ADHFE1	72	503	66	236	162	200
	266	656	166	129	233	80
ADI1	216	1493	172	621	356	558
	1039	1094	462	202	622	209
ADIG	2	1	0	0	0	0
	2	0	0	0	2	0
ADIPOQ	27	94	23	46	146	33
	57	108	15	10	23	166
ADIPOR1	172	791	128	624	323	688
	961	929	400	246	510	151
ADIPOR2	167	500	110	416	399	446
	716	700	335	231	376	143
ADK	41	291	56	261	107	249
	284	274	117	86	155	41
ADM	15	34	24	34	23	24
	14	69	21	11	19	6
ADM2	10	2	7	0	0	0
	0	0	4	0	0	0
ADNP	145	920	181	645	394	578
	581	698	290	208	346	104
ADNP2	21	51	28	44	25	39
	40	88	55	21	38	15
ADO	69	224	56	188	135	183
	213	243	97	65	147	27
ADORA1	18	6	7	6	1	4
	2	1	4	0	6	2
ADORA2A	10	8	2	5	3	6
	4	8	6	0	1	1
ADORA2B	4	3	5	3	3	0
	5	2	3	0	4	2
ADORA3	20	1	7	2	2	0
	0	6	5	0	7	1
ADPGK	29	57	36	76	34	69
	66	157	72	31	73	15
ADPRH	30	73	46	43	46	69
	62	98	45	40	55	10
ADPRHL1	141	832	151	837	327	744
	682	514	414	181	422	90
ADPRHL2	40	115	22	93	71	99
	110	170	71	37	77	16
ADPRM	22	57	25	68	35	43
	95	80	54	25	55	18
ADRA1A	27	14	14	11	4	1
	5	3	10	0	7	5
ADRA1B	6	1	6	1	2	2
	0	4	2	0	1	2
ADRA1D	4	1	3	1	2	0
	0	2	1	1	1	0
ADRA2A	8	18	13	4	6	0
	5	8	6	3	11	2
ADRA2B	18	23	9	13	10	13
	14	23	9	7	17	2
ADRA2C	6	4	3	1	2	4
	6	7	5	0	2	0
ADRB1	7	5	7	1	2	10
	1	7	4	2	3	1
ADRB2	42	157	35	80	108	78
	90	174	103	40	70	19

ADRB3	6	0	1	0	1	0
	0	0	1	0	1	0
ADRBK1	52	267	57	140	143	211
	191	254	136	74	146	28
ADRBK2	70	135	60	53	50	79
	59	99	90	13	63	29
ADRM1	71	249	55	242	112	202
	243	364	168	90	133	50
ADSL	430	2071	376	1840	988	1788
	2015	2743	1451	666	1480	325
ADSS	46	141	24	90	85	109
	158	167	70	53	80	29
ADSSL1	571	2683	344	2419	991	2035
	2981	1982	1483	887	1657	365
ADTRP	22	1	7	5	2	1
	0	0	11	0	3	0
AEBP1	25	127	32	89	31	31
	34	52	30	13	22	43
AEBP2	61	327	84	282	135	204
	265	247	138	72	164	32
AEN	12	28	12	17	23	26
	24	34	21	8	24	2
AES	296	1628	147	766	672	1167
	1199	1207	437	413	702	153
AFAP1	51	76	30	60	28	55
	50	68	48	29	44	16
AFAP1L1	54	419	90	400	302	347
	229	264	144	59	185	85
AFAP1L2	30	39	17	26	21	20
	29	35	36	10	19	10
AFF1	577	3385	668	2993	1438	2397
	2482	2904	1448	858	1651	622
AFF2	88	1	24	10	26	1
	4	1	26	3	18	0
AFF3	54	87	58	113	61	85
	67	70	64	32	62	18
AFF4	289	1518	445	1167	781	1104
	1201	1446	672	487	904	345
AFG3L2	373	1763	320	1337	789	1326
	1648	2000	828	558	1164	277
AFM	18	0	20	0	10	0
	0	0	29	0	9	0
AFMID	22	54	9	14	21	30
	62	36	27	19	19	7
AFP	23	0	13	0	9	0
	0	0	13	0	8	2
AFTPH	90	380	108	281	186	340
	255	413	192	110	194	60
AGA	37	111	32	95	45	117
	92	146	82	44	125	28
AGAP1	119	330	62	234	149	253
	220	260	149	109	154	58
AGAP10	9	16	15	5	7	18
	23	7	5	4	14	4
AGAP11	51	121	50	89	56	92
	87	100	65	34	75	26
AGAP2	40	71	24	38	41	40
	69	79	56	40	52	20

AGAP3	41	130	24	62	68	75
	84	127	54	35	55	20
AGAP4	1	38	5	13	11	19
	38	35	26	6	3	7
AGAP5	49	103	40	53	48	82
	66	121	56	32	51	23
AGAP6	62	180	58	120	68	146
	149	292	92	57	118	46
AGAP7	18	4	7	35	4	1
	0	6	12	1	37	0
AGAP8	1	1	0	0	0	0
	0	1	1	2	1	0
AGAP9	18	125	24	9	42	88
	78	113	10	34	77	24
AGBL1	120	304	124	155	190	396
	383	499	344	133	373	89
AGBL2	40	2	15	4	7	2
	2	2	18	0	8	0
AGBL3	50	48	43	64	27	43
	20	54	45	12	47	8
AGBL4	36	0	26	1	8	0
	0	2	13	1	18	0
AGBL5	30	63	19	34	25	47
	57	55	34	16	29	5
AGER	8	5	6	10	7	5
	4	8	3	2	4	1
AGFG1	147	611	167	580	334	500
	585	835	354	201	443	137
AGFG2	26	42	12	19	15	27
	15	56	29	11	36	13
AGGF1	125	454	113	367	258	463
	366	418	217	144	320	101
AGK	38	138	39	79	62	90
	97	164	80	26	81	22
AGL	3983	29901	5281	28511	14408	21600
	21311	21962	10895	5516	15127	2938
AGMAT	110	615	154	630	211	408
	518	361	226	153	138	55
AGMO	13	5	6	3	6	1
	3	5	12	1	9	0
AGPAT1	46	166	29	116	73	109
	142	158	69	43	86	34
AGPAT2	7	25	8	17	15	19
	32	50	20	9	16	17
AGPAT3	170	750	132	607	362	636
	646	865	339	265	446	217
AGPAT4	57	53	46	38	29	37
	28	45	39	14	37	11
AGPAT5	89	373	116	199	165	214
	380	453	288	131	307	74
AGPAT6	91	303	82	270	170	318
	292	464	207	98	263	89
AGPAT9	100	364	97	285	203	299
	452	759	201	200	321	122
AGPHD1	21	29	21	19	14	23
	18	31	25	6	25	4
AGPS	147	599	129	474	335	389
	423	600	321	140	337	99

AGR2	12	0	4	1	1	0
	0	0	2	0	0	0
AGR3	5	0	3	0	3	0
	0	0	3	0	3	0
AGRN	24	33	19	18	15	22
	25	25	22	11	24	5
AGRP	1	0	0	0	0	0
	0	0	0	0	0	0
AGT	46	117	15	50	43	52
	75	102	46	34	50	20
AGTPBP1	140	638	124	721	213	350
	463	678	355	138	492	140
AGTR1	24	83	33	56	55	69
	70	192	67	36	67	22
AGTR2	15	0	1	0	1	0
	0	1	5	0	3	0
AGTRAP	3	9	4	18	10	21
	11	41	19	8	22	2
AGXT	5	0	4	0	1	0
	0	0	2	0	1	0
AGXT2	20	0	10	0	6	0
	0	0	11	0	8	0
AGXT2L1	12	12	6	9	2	2
	27	6	19	2	15	2
AGXT2L2	33	157	43	89	60	134
	167	141	69	40	85	17
AHCTF1	259	1325	334	1083	725	910
	1132	1337	685	458	793	265
AHCY	58	212	52	166	96	188
	219	367	121	87	105	36
AHCYL1	265	1526	283	1106	741	1274
	1410	1146	574	307	696	166
AHCYL2	196	691	185	589	363	483
	510	544	271	135	328	110
AHDC1	46	251	57	150	88	146
	180	86	41	55	74	33
AHI1	90	334	96	314	179	232
	247	368	165	132	188	86
AHNAK	3928	24868	6410	19483	13717	16299
	15694	23964	10141	9169	13183	7366
AHNAK2	93	146	56	247	65	80
	56	128	56	11	58	33
AHR	55	178	70	198	90	140
	142	194	126	53	107	65
AHRR	25	0	15	0	2	0
	2	4	10	0	68	1
AHSA1	128	832	124	511	456	565
	625	1054	346	198	382	120
AHSA2	95	408	109	301	204	348
	298	517	222	113	294	92
AHSG	10	0	4	0	0	0
	0	0	4	0	1	0
AHSP	3	3	1	2	0	0
	0	0	1	0	2	0
AICDA	20	1	9	1	3	0
	0	2	6	1	1	0
AIDA	80	315	69	245	164	198
	247	288	103	72	122	50

AIF1	14	24	2	20	18	17
	17	38	15	7	20	3
AIF1L	34	148	18	82	68	94
	132	143	95	37	81	40
AIFM1	105	533	61	319	181	247
	459	353	152	116	252	68
AIFM2	22	55	19	39	37	43
	35	75	25	13	33	14
AIFM3	17	5	11	4	6	0
	4	0	12	0	1	0
AIG1	32	200	42	104	55	73
	185	126	84	19	104	22
AIM1	52	71	29	65	40	54
	43	80	62	20	44	17
AIM1L	26	0	9	0	1	0
	0	0	10	0	5	0
AIM2	13	8	6	5	1	4
	1	2	7	0	7	2
AIMP1	142	724	122	485	257	580
	620	754	353	186	410	115
AIMP2	78	646	80	545	211	505
	715	624	343	158	402	68
AIP	15	47	8	38	28	58
	68	85	45	22	29	16
AIPL1	10	0	11	1	0	0
	0	0	2	1	3	0
AIRE	6	0	4	0	1	0
	0	0	4	0	1	0
AJAP1	14	2	6	14	2	1
	8	12	13	8	6	3
AJUBA	27	18	14	6	8	8
	14	6	20	4	16	3
AK1	240	2891	393	1804	753	1843
	2554	1776	867	552	878	261
AK2	271	1197	181	670	483	847
	772	1539	625	272	658	163
AK3	393	3001	153	1316	1118	761
	1918	1331	729	625	1529	378
AK4	63	33	32	25	32	32
	18	40	43	10	19	8
AK5	26	7	19	9	11	3
	2	2	20	0	6	1
AK7	28	1	16	2	6	1
	1	4	17	0	9	3
AK8	15	0	10	2	0	0
	0	0	2	0	5	0
AKAP1	396	2131	312	1128	892	1199
	1735	2097	683	467	1070	288
AKAP10	79	252	72	207	132	186
	216	329	170	94	192	69
AKAP11	150	655	218	664	278	360
	522	581	254	186	365	115
AKAP12	157	644	155	669	331	534
	350	591	247	193	296	192
AKAP13	523	2206	523	1653	1085	1641
	2105	3160	1272	991	1658	536
AKAP14	6	0	3	0	0	1
	0	3	2	0	0	0

AKAP17A (NC_000023 1710485..1721413)			12	66	7	
	48	19	36	24	63	34
	11	17	12			
AKAP17A (NC_000024 1660485..1671413)			13	38	3	
	21	15	32	30	46	27
	14	41	6			
AKAP2	1	10	0	42	13	21
	6	19	1	2	0	10
AKAP3	15	16	16	2	8	12
	6	5	15	3	13	1
AKAP4	26	0	6	1	7	2
	0	0	10	0	6	0
AKAP5	35	15	27	10	11	8
	6	19	19	6	15	3
AKAP6	618	3041	742	1988	1752	2302
	2158	2808	1258	1305	1811	775
AKAP7	54	262	43	141	75	113
	206	182	72	56	83	34
AKAP8	67	224	79	204	175	238
	270	392	128	109	213	59
AKAP8L	65	278	55	223	163	258
	241	416	188	112	201	69
AKAP9	997	6703	1479	3713	2908	4261
	4412	5461	2011	2321	3146	1137
AKD1	97	180	68	107	131	128
	139	143	93	48	105	23
AKIP1	19	99	13	75	47	49
	65	80	48	16	52	22
AKIRIN1	30	191	31	194	86	145
	157	200	97	40	97	27
AKIRIN2	41	255	37	163	109	143
	214	159	50	73	96	31
AKNA	58	63	25	43	15	33
	26	47	39	9	23	11
AKNAD1	25	1	12	6	6	2
	0	4	15	0	10	0
AKR1A1	37	123	40	92	53	96
	110	156	75	39	62	17
AKR1B1	100	658	85	412	246	476
	742	677	264	152	360	76
AKR1B10	15	5	18	10	6	10
	10	8	24	2	8	4
AKR1B15	62	21	31	18	20	18
	13	5	26	6	19	3
AKR1C1	53	323	31	141	185	115
	129	132	78	72	39	60
AKR1C2	117	799	96	298	538	187
	92	127	101	173	51	62
AKR1C3	70	295	64	175	428	111
	66	76	60	58	39	31
AKR1C4	15	1	10	0	5	0
	0	0	5	1	7	0
AKR1D1	20	0	14	0	3	0
	0	0	12	0	5	0
AKR1E2	22	21	10	15	21	24
	17	16	22	5	17	2
AKR7A2	53	157	30	130	66	99
	111	185	89	51	96	34

AKR7A3	3	6	5	6	3	4
	1	5	2	3	3	0
AKT1	57	212	62	127	106	153
	171	283	156	82	111	65
AKT1S1	33	164	19	92	78	94
	115	139	44	35	77	25
AKT2	86	444	74	299	155	331
	398	532	218	154	272	106
AKT3	113	430	88	388	189	357
	420	462	199	129	285	83
AKTIP	231	1243	230	1062	471	1010
	934	962	568	298	685	169
ALAD	46	129	30	90	75	84
	87	74	47	36	61	34
ALAS1	98	611	94	382	252	334
	426	478	212	143	337	79
ALAS2	26	67	7	10	4	21
	9	6	9	3	1	0
ALB	28	0	15	2	12	1
	0	3	9	0	12	1
ALCAM	63	45	33	48	42	29
	35	45	41	8	36	10
ALDH16A1	8	20	9	13	8	17
	12	24	14	1	8	0
ALDH18A1	33	62	24	42	30	39
	28	55	32	11	28	9
ALDH1A1	122	578	147	631	386	498
	875	1335	334	209	683	181
ALDH1A2	53	143	42	98	100	185
	125	182	149	69	123	26
ALDH1A3	57	73	54	207	79	133
	66	86	54	31	54	18
ALDH1B1	49	238	34	72	127	205
	205	172	97	40	142	36
ALDH1L1	80	425	89	284	191	230
	375	487	286	161	296	66
ALDH1L2	78	92	53	90	50	69
	50	55	56	28	39	17
ALDH2	180	440	84	328	376	314
	484	671	316	241	331	169
ALDH3A1	15	3	7	1	2	0
	0	0	5	0	5	0
ALDH3A2	139	603	171	398	342	458
	421	668	290	170	338	119
ALDH3B1	16	3	7	0	3	7
	3	9	8	5	5	1
ALDH3B2	14	0	6	0	1	0
	0	0	4	0	2	0
ALDH4A1	37	157	37	103	92	63
	199	253	80	38	120	35
ALDH5A1	170	915	192	897	531	520
	989	654	297	276	504	169
ALDH6A1	52	217	52	86	94	65
	129	403	111	49	137	48
ALDH7A1	46	118	26	49	58	43
	125	129	57	25	58	16
ALDH8A1	32	22	28	28	38	53
	8	57	36	9	24	8

ALDH9A1	194	773	172	800	362	747
	666	1059	473	214	509	167
ALDOA	5445	35774	5304	23434	12309	28516
	37430	25259	18516	10594	16782	3473
ALDOB	36	0	11	1	5	1
	0	0	12	0	8	0
ALDOC	8	9	2	7	0	7
	5	9	8	1	6	2
ALG1	66	59	18	42	24	40
	30	64	43	11	38	9
ALG10	10	52	29	56	28	37
	57	63	29	23	61	18
ALG10B	91	275	99	294	179	229
	234	298	164	94	242	72
ALG11	39	122	42	94	83	99
	90	138	65	31	98	24
ALG12	17	53	17	36	33	48
	46	77	38	10	38	4
ALG13	93	298	77	223	160	190
	216	307	131	77	186	48
ALG14	20	37	12	37	22	46
	35	96	33	10	38	15
ALG1L	5	3	4	2	1	10
	1	10	8	2	2	0
ALG1L2	2	2	7	0	2	2
	0	0	8	1	2	0
ALG2	40	101	27	74	64	96
	120	134	69	46	96	32
ALG3	15	41	14	20	38	39
	59	75	26	22	35	10
ALG5	28	79	28	84	43	89
	83	129	69	29	56	20
ALG6	33	136	41	111	57	90
	74	186	57	35	101	19
ALG8	35	54	18	38	24	39
	21	88	34	16	31	8
ALG9	93	253	73	202	113	174
	159	266	131	78	125	46
ALK	39	1	22	1	10	2
	1	3	14	0	10	2
ALKBH1	36	69	29	75	43	56
	92	111	47	29	59	13
ALKBH2	18	35	18	31	23	23
	37	49	22	12	31	5
ALKBH3	60	235	58	149	90	173
	156	296	131	69	148	34
ALKBH4	14	18	7	12	13	13
	15	22	14	8	14	4
ALKBH5	382	2317	298	1855	1036	1655
	1817	1876	843	498	1216	351
ALKBH6	6	12	7	7	6	7
	25	23	9	0	10	3
ALKBH7	45	138	7	99	61	76
	136	190	86	37	61	10
ALKBH8	65	211	62	144	113	157
	132	188	120	63	128	32
ALLC	4	0	10	1	0	1
	0	0	12	0	4	1

ALMS1	209	780	301	566	350	534
	455	668	330	284	390	149
ALOX12	22	2	10	2	4	2
	0	1	5	1	1	0
ALOX12B	17	0	15	0	5	0
	0	0	4	0	5	0
ALOX15	20	1	8	0	5	3
	0	0	6	0	6	0
ALOX15B	13	4	11	2	2	1
	2	1	4	1	3	0
ALOX5	13	15	14	14	5	9
	7	11	19	3	11	2
ALOX5AP	8	3	7	3	17	13
	10	23	14	4	4	1
ALOXE3	20	0	12	1	2	0
	0	0	10	0	4	0
ALPI	4	0	3	0	2	0
	0	0	3	0	3	0
ALPK1	68	106	59	59	49	48
	65	93	56	15	42	13
ALPK2	755	1855	493	634	1657	1196
	1344	3130	458	1143	1331	510
ALPK3	732	4307	539	2041	1715	2341
	2338	5473	1483	1681	2917	1123
ALPL	19	5	4	0	6	5
	0	1	8	7	4	2
ALPP	12	0	10	0	0	0
	0	0	2	0	1	0
ALPPL2	10	0	1	0	0	0
	0	0	6	0	1	0
ALS2	194	577	188	467	341	522
	428	733	359	229	435	99
ALS2CL	54	159	30	86	72	88
	111	202	120	61	124	30
ALS2CR11	58	11	38	15	15	14
	9	22	50	4	26	6
ALS2CR12	35	43	22	17	16	28
	35	56	26	13	32	3
ALS2CR8	75	307	74	247	137	173
	206	331	150	73	170	49
ALX1	6	2	5	0	3	1
	1	3	8	0	7	0
ALX3	2	0	2	0	0	0
	0	5	4	0	2	0
ALX4	17	6	10	31	3	28
	7	3	23	2	8	1
ALYREF	29	141	22	80	48	88
	95	137	62	36	66	23
AMACR	28	169	57	189	105	176
	159	395	121	82	165	38
AMBN	22	0	9	0	3	0
	0	0	12	0	3	0
AMBP	5	0	8	1	4	0
	0	0	7	0	6	0
AMBRA1	74	215	64	155	114	120
	153	253	122	65	138	45
AMD1	121	479	101	371	209	374
	472	619	233	172	361	102

AMDHD1	12	5	8	9	6	6
	12	8	11	4	7	2
AMDHD2	7	10	9	12	7	18
	13	10	11	7	11	4
AMELX	4	0	2	0	2	1
	0	3	4	0	1	0
AMELY	0	0	1	0	0	0
	0	0	3	0	0	0
AMFR	348	1106	200	1113	674	1074
	971	1350	687	487	815	228
AMH	1	5	4	1	2	2
	5	6	1	1	4	0
AMHR2	6	7	3	5	2	4
	10	9	7	2	6	2
AMICA1	33	20	20	21	12	18
	25	31	17	12	12	1
AMIGO1	24	93	22	59	50	134
	78	83	71	26	41	17
AMIGO2	21	8	5	15	7	2
	6	5	12	4	7	2
AMIGO3	14	20	5	12	8	9
	9	21	10	4	13	3
AMMECR1	25	49	22	61	25	38
	40	46	34	21	28	8
AMMECR1L	68	132	24	87	61	83
	100	129	83	44	90	30
AMN	1	0	1	0	0	0
	0	0	0	0	0	0
AMN1	41	167	33	117	100	135
	134	193	99	32	108	32
AMOT	343	2701	458	1245	1040	1345
	1189	1661	472	483	694	218
AMOTL1	592	4250	1152	2151	1503	2495
	1456	3334	991	708	1221	499
AMOTL2	29	199	56	175	142	178
	116	151	80	61	111	32
AMPD1	1310	10439	1706	6048	4059	5893
	6317	8389	4915	2385	6172	2067
AMPD2	12	36	15	23	7	18
	14	15	21	9	19	6
AMPD3	108	127	65	119	63	48
	84	582	145	102	131	94
AMPH	31	25	22	33	24	35
	25	41	38	8	16	10
AMT	35	76	22	58	49	67
	69	88	33	25	33	17
AMTN	11	0	8	0	2	0
	0	0	4	0	0	0
AMY1A	27	2	28	0	36	0
	0	0	35	0	24	0
AMY1B	8	1	4	0	7	0
	0	0	4	2	4	0
AMY1C	30	0	21	2	1	1
	0	0	13	0	31	0
AMY2A	31	0	18	12	16	6
	1	4	22	2	22	0
AMY2B	190	866	289	1011	481	948
	1020	1401	800	382	1287	182

AMZ1	13	0	6	1	2	1
	0	1	4	0	1	1
AMZ2	107	578	84	404	235	372
	649	692	284	188	403	86
ANAPC1	365	1103	280	770	726	645
	867	1055	594	289	886	180
ANAPC10	23	116	25	85	57	76
	124	161	77	48	82	23
ANAPC11	32	107	18	86	45	76
	170	237	82	30	80	21
ANAPC13	62	313	48	216	101	195
	321	508	159	91	159	41
ANAPC15	31	162	16	116	62	117
	155	134	65	45	67	19
ANAPC16	331	1715	273	1031	940	1487
	1612	2485	858	595	1152	309
ANAPC2	15	43	17	17	18	22
	51	36	15	13	26	1
ANAPC4	73	252	81	202	137	192
	158	423	166	76	156	34
ANAPC5	172	750	146	572	307	556
	633	888	461	205	469	142
ANAPC7	87	305	63	215	136	213
	187	365	136	92	164	65
ANG	0	3	0	2	0	4
	0	6	2	1	0	0
ANGEL1	33	91	19	73	45	58
	77	122	57	33	65	20
ANGEL2	101	336	90	298	164	335
	333	500	191	108	274	70
ANGPT1	191	1093	392	734	645	610
	903	1188	747	360	675	207
ANGPT2	48	113	60	57	52	53
	36	95	56	32	58	19
ANGPT4	8	0	4	0	1	0
	0	0	2	0	0	0
ANGPTL1	146	450	85	321	284	362
	313	932	445	182	343	131
ANGPTL2	84	274	72	206	202	312
	192	240	201	117	224	89
ANGPTL3	12	2	12	0	9	0
	0	0	18	0	5	1
ANGPTL4	3	8	21	7	17	16
	20	51	6	8	8	3
ANGPTL5	28	11	8	9	16	14
	4	12	21	3	11	2
ANGPTL6	8	0	2	0	0	0
	1	4	4	0	0	0
ANGPTL7	25	2	15	67	5	3
	23	3	9	5	6	17
ANHX	11	0	7	0	2	0
	0	0	5	0	0	0
ANK1	621	3600	599	1707	1359	2543
	3232	3527	1537	1036	1795	526
ANK2	719	3065	732	2409	1728	1900
	2653	2510	1203	1215	1956	570
ANK3	485	2176	618	1263	944	1239
	1440	1668	742	603	1098	365

ANKAR	88	240	82	205	107	142
	174	327	177	75	147	41
ANKDD1A	13	13	19	17	7	2
	7	21	20	5	2	3
ANKDD1B	26	2	15	5	7	3
	3	4	11	0	10	0
ANKFN1	22	0	17	1	4	0
	1	0	12	1	8	0
ANKFY1	130	427	168	239	222	272
	436	728	296	145	322	106
ANKH	178	951	165	703	481	561
	958	805	402	242	425	160
ANKHD1	137	1261	261	899	478	825
	816	1161	464	338	573	211
ANKHD1-EIF4EBP3		227	655	214	538	356
	583	461	750	297	244	454
	118					
ANKIB1	166	484	156	412	240	357
	406	526	273	155	305	109
ANKK1	3	3	7	2	3	2
	1	7	5	1	4	1
ANKLE1	14	1	8	2	1	2
	1	1	5	0	1	1
ANKLE2	74	368	82	282	157	270
	345	381	234	127	236	92
ANKMY1	23	45	12	11	15	25
	16	41	25	11	22	2
ANKMY2	60	386	71	198	121	209
	178	288	103	80	148	41
ANKRA2	51	100	56	106	35	99
	102	132	67	41	69	28
ANKRD1	72	2444	541	888	191	1264
	276	606	862	458	99	35
ANKRD10	209	1033	268	867	349	733
	788	1359	659	273	566	182
ANKRD11	113	627	146	342	230	367
	380	396	165	177	205	138
ANKRD12	315	1869	436	1344	898	1525
	1628	2401	869	583	972	377
ANKRD13A	89	242	57	173	119	168
	182	294	145	68	146	48
ANKRD13B	13	24	7	11	10	15
	10	11	11	1	7	5
ANKRD13C	85	453	88	336	218	289
	309	513	225	149	328	90
ANKRD13D	10	19	8	14	12	13
	23	15	12	7	10	5
ANKRD16	30	51	26	37	25	32
	41	72	39	12	30	6
ANKRD17	430	2359	533	1467	1018	1430
	1399	2043	858	690	1141	380
ANKRD18A	36	2	15	2	12	0
	0	3	20	0	10	0
ANKRD18B	50	3	19	0	16	10
	5	16	24	9	22	4
ANKRD2	225	1499	73	126	312	723
	832	2272	634	308	263	157

ANKRD20A1	72	59	29	26	37	18
	48	40	61	10	55	3
ANKRD20A19P		53	9	25	3	10
	7	7	16	32	6	28
	3					
ANKRD20A2	13	4	17	2	6	0
	4	11	26	2	8	5
ANKRD20A3	16	8	13	8	12	10
	11	8	45	4	21	3
ANKRD20A4	71	35	59	40	54	30
	45	58	71	29	34	14
ANKRD22	21	1	10	6	4	0
	0	0	15	0	10	0
ANKRD23	1750	8428	1302	5150	3961	7581
	6265	11209	4473	3726	5931	1560
ANKRD24	12	9	7	10	10	4
	5	10	9	0	6	5
ANKRD26	163	903	189	461	346	530
	797	803	403	238	496	140
ANKRD27	73	219	77	194	114	179
	161	238	112	84	139	63
ANKRD28	263	1437	320	716	563	839
	753	1270	555	450	603	193
ANKRD29	20	42	8	21	14	17
	23	29	22	9	20	7
ANKRD30A	43	1	35	0	14	1
	3	0	30	1	21	0
ANKRD30B	48	1	24	1	18	1
	3	1	47	3	28	1
ANKRD31	43	7	33	7	21	4
	2	7	32	0	15	0
ANKRD32	30	77	32	45	37	50
	95	123	46	23	72	26
ANKRD33	5	0	3	0	2	0
	0	0	2	0	3	0
ANKRD33B	97	70	56	31	60	28
	70	430	82	43	94	44
ANKRD34A	13	2	12	6	7	8
	3	19	6	2	6	2
ANKRD34B	20	0	8	0	3	0
	0	0	8	0	8	0
ANKRD34C	33	0	17	0	5	0
	0	0	18	0	4	0
ANKRD35	33	62	30	56	31	49
	46	52	32	28	34	19
ANKRD36	285	1016	304	648	477	697
	681	1071	446	380	688	192
ANKRD36B	103	174	139	125	80	106
	208	139	196	55	100	40
ANKRD36C	233	685	274	472	275	417
	351	607	368	199	480	143
ANKRD37	24	78	23	72	54	97
	88	106	52	33	51	9
ANKRD39	7	23	1	10	10	14
	23	17	13	5	13	5
ANKRD40	278	1687	252	1317	839	1225
	1369	1610	682	519	837	328

ANKRD42	28	17	21	5	14	20
	23	17	29	4	24	9
ANKRD44	71	126	80	128	121	171
	135	193	124	54	119	34
ANKRD45	11	1	10	0	2	0
	2	0	6	1	5	0
ANKRD46	117	591	123	579	342	582
	543	728	366	124	338	78
ANKRD49	21	52	17	57	36	42
	61	82	35	14	51	16
ANKRD5	50	30	23	13	27	20
	20	20	32	5	20	3
ANKRD50	80	334	96	227	201	204
	163	189	125	69	121	46
ANKRD52	125	455	114	422	184	400
	242	275	149	74	165	59
ANKRD53	4	0	10	1	1	1
	1	0	4	1	5	0
ANKRD54	21	59	5	27	28	29
	34	27	17	13	18	12
ANKRD55	18	0	13	0	3	0
	0	0	16	0	5	0
ANKRD6	71	106	52	71	44	57
	76	90	68	32	70	22
ANKRD60	6	0	4	0	1	0
	0	0	5	0	2	0
ANKRD62	64	3	27	3	17	7
	3	7	31	1	17	0
ANKRD65	4	12	6	6	7	4
	11	7	8	4	6	2
ANKRD7	2	3	5	1	5	1
	5	5	7	0	3	0
ANKRD9	68	155	29	137	74	125
	262	279	188	83	121	36
ANKS1A	114	399	86	309	170	305
	320	437	198	104	216	71
ANKS1B	119	350	108	339	108	207
	224	89	195	74	131	23
ANKS3	16	42	22	25	25	35
	51	49	36	9	20	12
ANKS4B	27	0	17	0	4	0
	0	0	15	0	12	0
ANKS6	50	83	45	51	44	71
	46	93	49	34	40	12
ANKUB1	15	0	6	0	6	0
	0	0	10	0	9	1
ANKZF1	70	307	67	185	129	208
	240	543	210	99	204	64
ANLN	48	5	34	6	14	10
	8	6	26	0	15	0
ANO1	39	115	24	51	47	112
	64	76	45	30	41	13
ANO10	38	131	27	84	72	106
	63	104	72	24	74	17
ANO2	53	86	24	38	47	60
	58	80	48	23	55	19
ANO3	42	6	33	1	18	2
	1	0	35	0	18	2

ANO4	31	0	22	7	16	3
	6	0	26	0	15	1
ANO5	733	3998	749	3853	2298	3351
	3218	5428	1434	960	1857	619
ANO6	677	3023	665	2156	1608	2217
	2670	2935	1307	976	1757	537
ANO7	20	2	8	0	9	2
	1	0	8	0	2	1
ANO8	21	53	21	18	19	28
	35	40	28	11	20	5
ANO9	4	2	4	0	2	0
	1	0	4	0	0	0
ANP32A	111	404	82	222	188	219
	267	438	177	96	201	77
ANP32B	18	92	17	45	54	62
	56	66	25	17	40	16
ANP32D	0	1	0	3	2	3
	0	1	3	0	1	0
ANP32E	118	520	87	402	291	402
	435	562	206	147	294	66
ANPEP	21	8	7	18	5	10
	6	19	11	1	7	4
ANTXR1	102	351	77	237	176	185
	231	287	104	63	136	75
ANTXR2	94	293	109	233	161	289
	189	494	169	91	211	70
ANXA1	76	323	127	310	223	246
	229	496	253	130	185	154
ANXA10	8	1	11	0	4	0
	0	2	4	0	3	0
ANXA11	132	741	166	519	239	477
	532	716	306	201	350	134
ANXA13	16	0	11	0	3	0
	0	3	12	0	2	0
ANXA2	183	857	196	746	470	745
	534	921	378	211	356	221
ANXA2R	8	24	7	19	21	21
	8	20	13	3	10	4
ANXA3	38	129	25	69	61	73
	230	270	149	75	163	30
ANXA4	35	144	40	72	65	118
	88	157	53	35	56	33
ANXA5	94	526	96	420	235	348
	408	627	325	166	276	122
ANXA6	380	1957	402	1307	896	1363
	1370	1575	879	595	1003	322
ANXA7	325	1502	317	1337	742	1218
	1510	2150	945	492	1006	324
ANXA8	3	0	2	1	2	0
	0	0	0	0	1	0
ANXA8L1	5	0	7	2	3	0
	0	0	11	0	0	0
ANXA8L2	20	1	14	0	3	0
	0	0	9	0	4	0
ANXA9	15	2	7	2	1	0
	0	1	8	0	1	0
AOAH	28	20	16	16	10	11
	22	17	19	5	20	3

AOC2	5	1	10	1	6	5
	4	7	10	0	7	1
AOC3	26	119	17	43	69	38
	57	80	32	26	39	54
AOX1	52	46	49	115	37	62
	47	155	55	12	68	35
AP1AR	60	199	49	134	85	126
	158	194	79	43	101	32
AP1B1	77	240	59	162	103	182
	150	251	98	62	149	43
AP1G1	236	1024	220	868	521	768
	870	1025	588	308	612	206
AP1G2	20	24	19	19	14	9
	13	18	15	5	11	1
AP1M1	23	87	22	64	58	68
	70	97	48	27	55	15
AP1M2	8	0	3	1	0	0
	1	0	0	0	2	0
AP1S1	5	19	1	13	10	10
	10	35	12	3	14	3
AP1S2	192	853	174	655	398	716
	929	1232	613	306	655	152
AP1S3	18	8	10	4	6	6
	5	13	9	2	8	1
AP2A1	37	220	34	124	78	161
	142	179	106	41	108	29
AP2A2	57	187	51	121	128	107
	143	173	84	65	100	36
AP2B1	150	999	192	713	417	576
	838	849	365	248	436	156
AP2M1	124	612	87	434	274	466
	469	537	283	139	310	103
AP2S1	17	69	12	52	30	65
	60	115	37	21	37	5
AP3B1	102	372	84	292	198	317
	252	376	191	125	212	75
AP3B2	30	0	24	3	7	2
	2	2	13	0	8	0
AP3D1	97	477	114	240	198	282
	256	484	178	157	201	111
AP3M1	88	297	94	193	170	206
	237	288	138	100	198	61
AP3M2	50	141	39	88	66	110
	113	113	67	26	94	19
AP3S1	48	143	40	134	102	137
	128	203	83	42	85	27
AP3S2	28	167	42	124	115	145
	170	206	81	52	109	13
AP4B1	42	159	44	115	69	123
	147	145	98	44	113	21
AP4E1	119	572	146	460	241	362
	368	512	271	135	311	101
AP4M1	21	23	6	18	18	18
	22	45	25	8	18	0
AP4S1	64	143	52	71	68	88
	76	172	60	25	93	21
AP5B1	26	65	9	35	23	35
	45	60	30	16	39	14

AP5M1	90	407	68	367	206	326
	317	487	290	140	280	68
AP5S1	19	88	12	33	36	42
	58	74	42	21	49	12
AP5Z1	30	36	13	17	16	22
	38	33	20	7	23	7
APAF1	64	220	92	140	105	156
	128	174	120	52	106	26
APBA1	44	76	48	36	58	83
	85	68	45	37	56	7
APBA2	19	0	11	0	1	1
	1	3	6	0	6	0
APBA3	9	31	8	15	8	17
	15	38	18	8	17	7
APBB1	36	149	43	93	54	130
	116	181	70	63	86	24
APBB1IP	23	23	20	22	18	17
	9	12	25	7	13	6
APBB2	223	740	167	583	369	482
	617	759	413	198	540	189
APBB3	26	45	26	40	31	49
	33	79	39	17	46	11
APC	306	1608	356	1162	773	1257
	976	1396	586	500	701	312
APC2	18	2	3	0	2	0
	0	1	6	0	4	0
APCDD1	17	46	8	27	37	37
	33	48	43	17	27	17
APCDD1L	11	1	4	6	8	3
	1	1	4	1	1	0
APCS	7	0	5	0	2	1
	0	0	2	0	0	0
APEH	97	441	67	286	163	263
	256	473	203	99	252	51
APEX1	68	316	39	187	120	209
	415	312	171	101	178	49
APEX2	12	26	11	10	6	20
	20	17	6	6	6	6
APH1A	44	113	42	114	60	110
	136	201	97	34	94	25
APH1B	63	65	45	77	59	88
	57	101	67	37	78	11
API5	148	674	126	528	336	516
	563	749	358	220	430	116
APIP	79	315	85	568	153	365
	405	376	218	104	230	64
APITD1	0	0	3	5	2	3
	10	6	7	0	1	1
APITD1-CORT		11	4	5	7	10
	5	4	3	7	1	4
	0					
APLF	60	164	78	107	97	114
	162	181	98	67	114	40
APLN	19	14	10	4	11	7
	8	5	14	5	9	1
APLNR	36	146	21	74	48	69
	81	68	87	37	48	7

APLP1	10	1	7	0	2	2
	1	2	3	0	7	0
APLP2	152	857	165	525	470	605
	647	787	397	265	481	200
APMAP	37	122	48	99	81	108
	124	184	98	41	78	40
APOA1	1	0	0	0	1	0
	0	0	2	0	2	0
APOA1BP	22	112	13	54	25	56
	69	126	37	17	32	9
APOA2	4	0	0	0	0	0
	0	0	3	0	0	0
APOA4	2	1	3	0	0	0
	0	0	1	0	1	0
APOA5	5	0	6	0	0	0
	0	0	1	0	0	0
APOB	101	11	63	5	18	16
	12	32	43	5	31	20
APOBEC1	10	0	7	0	1	0
	0	0	3	0	1	0
APOBEC2	828	3916	976	2828	1827	3163
	2581	5338	1991	1060	2478	547
APOBEC3A	12	2	5	0	2	3
	0	2	5	0	3	1
APOBEC3B	13	0	9	0	3	1
	0	1	3	1	2	1
APOBEC3C	4	5	1	3	4	0
	1	9	3	0	8	2
APOBEC3D	17	14	6	10	6	3
	2	9	8	4	13	3
APOBEC3F	26	19	9	13	10	5
	3	15	13	5	13	0
APOBEC3G	12	13	5	6	10	13
	15	15	21	2	8	5
APOBEC3H	3	0	5	0	0	0
	0	2	3	0	2	0
APOBEC4	20	0	5	0	5	0
	0	0	7	0	3	0
APOBR	17	1	16	6	9	4
	2	0	13	1	12	1
APOC1	8	14	2	3	8	8
	20	20	14	9	14	5
APOC2	5	0	3	0	4	0
	0	0	0	0	1	0
APOC3	3	0	3	0	0	0
	0	0	0	0	0	0
APOC4	0	0	3	0	0	0
	0	0	0	0	1	0
APOD	290	955	480	2962	310	678
	1527	1788	670	644	861	309
APOE	20	70	8	31	41	30
	123	118	63	26	61	16
APOF	10	0	3	0	2	0
	0	0	5	0	5	0
APOH	12	0	1	0	2	0
	0	0	4	0	1	0
APOL1	18	77	28	43	37	41
	43	81	23	24	38	12

APOL2	42	112	47	83	69	86
	145	136	82	36	77	27
APOL3	61	187	37	104	63	115
	140	214	99	67	132	34
APOL4	46	12	31	13	25	21
	16	41	26	7	16	2
APOL5	4	3	6	4	2	27
	23	9	20	6	3	2
APOL6	334	454	160	228	237	295
	256	652	304	200	290	115
APOLD1	32	143	49	169	55	92
	90	195	64	35	94	35
APOM	15	7	4	9	4	10
	10	10	19	2	12	2
APOO	23	182	12	95	65	91
	155	180	63	35	84	19
APOOL	156	731	124	529	348	418
	539	694	263	225	374	129
APOPT1	92	454	54	202	151	331
	595	597	199	148	281	87
APP	228	1082	188	800	482	806
	809	989	418	358	596	212
APPBP2	127	494	112	469	286	363
	343	584	268	144	328	98
APPL1	507	3020	572	2855	1468	2726
	3506	2858	1498	814	2197	505
APPL2	73	234	71	184	113	165
	177	300	141	75	144	56
APRT	7	39	11	35	16	19
	22	61	25	7	23	7
APTX	33	72	28	61	38	63
	64	101	42	26	55	16
AQP1	187	687	94	603	450	400
	764	1036	581	398	623	177
AQP10	9	0	0	0	4	0
	0	0	0	0	0	0
AQP11	9	11	4	4	4	12
	6	5	8	1	4	1
AQP12A	2	0	0	0	0	0
	0	0	0	0	0	0
AQP12B	0	0	0	0	0	0
	0	0	1	0	0	0
AQP2	33	0	9	0	4	0
	0	0	10	0	0	0
AQP3	20	9	4	4	4	2
	13	18	5	1	3	1
AQP4	524	2138	601	2192	2156	3097
	1935	2365	1180	570	1651	466
AQP5	6	0	2	0	0	0
	0	0	1	0	3	0
AQP6	8	0	4	3	0	0
	0	0	4	1	4	0
AQP7	60	109	38	46	74	53
	61	179	42	26	52	25
AQP8	14	0	8	0	2	4
	1	0	4	0	4	0
AQP9	13	10	12	2	1	5
	4	3	11	1	4	1

AQPEP	49	41	33	35	33	24
	25	48	22	9	25	19
AQR	133	352	128	272	179	282
	341	445	231	125	248	75
AR	255	934	161	794	587	787
	740	691	404	302	356	125
ARAF	68	363	63	251	129	253
	309	328	159	113	179	66
ARAP1	109	237	81	183	128	209
	187	307	122	72	145	60
ARAP2	67	115	68	82	57	68
	40	88	65	14	74	14
ARAP3	29	84	28	62	42	35
	97	73	39	13	37	19
ARC	2	0	5	0	1	0
	0	3	3	0	2	0
ARCN1	129	613	134	403	347	411
	460	579	240	209	329	112
AREG	3	0	1	0	1	0
	3	31	2	0	5	0
AREGB	7	0	1	0	1	0
	1	5	3	0	3	0
ARF1	140	646	117	489	312	448
	659	598	322	228	374	122
ARF3	43	90	22	73	43	58
	77	101	43	26	54	14
ARF4	61	302	68	295	146	290
	273	397	148	72	155	51
ARF5	22	84	19	58	28	44
	101	136	51	12	46	17
ARF6	58	263	53	198	117	222
	231	230	120	65	166	49
ARFGAP1	28	88	37	60	51	74
	94	148	80	42	84	19
ARFGAP2	112	420	119	287	230	350
	318	590	201	135	277	103
ARFGAP3	95	422	154	342	254	535
	381	434	210	114	268	54
ARFGEF1	228	1050	259	821	440	750
	645	934	441	299	545	195
ARFGEF2	269	1107	237	820	614	708
	825	1365	577	331	580	219
ARFIP1	43	218	49	146	95	155
	127	227	120	45	110	46
ARFIP2	55	72	22	74	36	59
	93	112	45	23	44	19
ARFRP1	23	161	32	78	40	79
	116	193	47	28	65	27
ARG1	5	2	8	0	4	3
	1	5	14	1	6	1
ARG2	18	42	22	82	64	54
	18	83	74	6	28	12
ARGFX	34	0	6	0	4	3
	0	7	14	0	11	0
ARGLU1	109	602	154	440	291	579
	512	786	358	225	415	157
ARHGAP1	24	68	17	53	26	37
	67	64	46	24	42	17

ARHGAP10	130	824	266	524	413	566
	601	849	377	251	475	203
ARHGAP11A	47	17	35	18	26	26
	12	36	32	11	26	8
ARHGAP11B	5	8	11	13	9	10
	4	2	8	0	6	1
ARHGAP12	177	719	204	594	294	528
	498	763	347	203	365	92
ARHGAP15	20	39	13	18	9	16
	24	27	18	7	17	11
ARHGAP17	72	265	69	167	122	161
	169	195	94	53	82	49
ARHGAP18	119	539	113	408	340	413
	417	463	236	148	276	87
ARHGAP19	7	86	41	71	57	73
	53	118	51	22	66	20
ARHGAP20	37	67	37	68	45	48
	22	39	31	10	29	18
ARHGAP21	395	1806	439	1460	984	1431
	1444	2129	688	629	1034	365
ARHGAP22	22	34	15	19	13	29
	8	31	17	6	17	4
ARHGAP23	31	67	22	33	32	33
	33	45	36	16	29	9
ARHGAP24	80	176	43	117	124	90
	122	217	101	60	113	58
ARHGAP25	30	34	13	24	20	27
	36	39	26	21	33	7
ARHGAP26	126	294	108	218	219	206
	247	287	146	104	151	49
ARHGAP27	24	35	22	26	13	18
	29	26	33	11	17	4
ARHGAP28	82	298	161	224	94	144
	88	131	108	28	95	32
ARHGAP29	174	920	168	693	407	643
	799	899	521	339	581	149
ARHGAP30	31	19	21	16	13	22
	2	27	14	3	18	3
ARHGAP31	102	329	61	174	113	126
	231	247	151	67	130	48
ARHGAP32	147	303	119	259	153	315
	244	370	195	135	180	49
ARHGAP33	13	6	5	4	2	3
	7	2	6	1	4	2
ARHGAP35	183	1175	171	766	561	675
	782	728	298	220	421	181
ARHGAP36	34	70	74	12	8	45
	50	16	31	2	7	0
ARHGAP39	20	18	15	1	6	6
	4	1	8	0	12	3
ARHGAP4	19	16	7	12	21	20
	19	22	17	5	9	6
ARHGAP40	14	0	8	0	2	0
	0	0	9	0	3	0
ARHGAP42	93	209	80	215	86	89
	104	204	97	47	108	39
ARHGAP44	56	23	24	19	22	22
	40	46	20	8	14	8

ARHGAP5	394	2446	548	1968	940	1790
	1641	1730	819	582	999	414
ARHGAP6	74	238	36	127	116	205
	176	162	111	65	175	31
ARHGAP9	23	10	14	11	8	4
	9	12	8	3	8	3
ARHGDIA	12	80	8	41	26	21
	51	58	27	17	25	7
ARHGDIB	32	260	27	104	78	115
	156	279	108	70	109	33
ARHGDIG	1	0	4	0	1	4
	0	0	1	0	1	0
ARHGEF1	23	60	23	50	15	26
	52	54	21	13	35	10
ARHGEF10	44	126	45	84	47	65
	95	80	72	31	67	28
ARHGEF10L	101	512	131	337	194	451
	427	473	244	140	273	81
ARHGEF11	131	401	120	302	184	309
	244	369	189	117	188	87
ARHGEF12	683	2710	805	1924	1365	1644
	1758	2997	1168	959	1586	707
ARHGEF15	28	130	27	55	35	53
	83	66	36	27	36	19
ARHGEF16	10	2	10	3	0	2
	8	4	7	1	6	0
ARHGEF17	30	132	25	66	46	43
	70	99	40	32	52	17
ARHGEF18	48	81	15	45	39	57
	50	67	60	23	55	26
ARHGEF19	10	2	8	0	5	3
	3	3	4	0	7	0
ARHGEF2	56	201	63	148	97	160
	108	166	85	52	68	28
ARHGEF25	32	110	28	44	24	31
	34	83	37	27	32	13
ARHGEF26	43	32	24	33	14	20
	30	28	47	16	27	6
ARHGEF28	76	41	46	55	43	17
	27	49	53	8	30	9
ARHGEF3	50	182	43	95	76	85
	179	180	105	41	102	31
ARHGEF33	28	4	15	13	14	15
	1	21	18	2	16	1
ARHGEF35	24	8	15	1	8	3
	2	6	15	1	9	0
ARHGEF37	93	273	49	256	138	192
	142	189	120	35	107	66
ARHGEF38	50	4	36	4	14	4
	4	3	13	1	15	1
ARHGEF39	21	4	8	3	1	1
	2	5	9	0	8	1
ARHGEF4	24	50	20	29	32	51
	31	47	26	10	19	4
ARHGEF40	50	124	48	97	78	128
	112	126	65	40	59	33
ARHGEF5	67	8	28	12	14	6
	10	11	33	4	33	4

ARHGEF6	484	1819	220	1520	970	1349
	1595	1929	882	560	1040	377
ARHGEF7	179	694	209	577	343	512
	596	770	376	211	413	141
ARHGEF9	61	122	49	103	87	73
	81	113	60	33	68	25
ARID1A	131	489	97	295	195	275
	374	349	169	148	171	102
ARID1B	180	1002	194	701	416	646
	770	779	418	359	459	189
ARID2	127	607	152	440	288	399
	407	575	320	200	302	137
ARID3A	6	13	4	5	8	8
	1	17	11	4	1	5
ARID3B	27	25	19	21	21	23
	19	30	26	9	25	5
ARID3C	3	0	5	1	1	0
	0	0	4	0	3	0
ARID4A	82	409	82	234	166	192
	255	338	123	92	178	73
ARID4B	104	505	132	350	272	392
	362	561	240	188	283	126
ARID5A	14	45	9	30	19	22
	45	101	47	10	33	13
ARID5B	257	1043	270	529	584	522
	1650	2353	637	336	1144	328
ARIH1	277	1530	279	1042	649	923
	1058	1512	540	424	752	240
ARIH2	468	1585	271	1092	887	1181
	1404	2422	703	539	1158	321
ARIH2OS	7	8	1	4	3	6
	2	19	5	0	3	0
ARL1	181	934	171	679	379	603
	723	1094	363	213	482	129
ARL10	21	88	21	62	43	54
	83	71	26	21	30	3
ARL11	23	8	10	0	5	4
	0	3	9	1	10	0
ARL13A	14	1	5	0	4	6
	0	2	2	0	2	0
ARL13B	26	59	28	41	26	35
	31	59	26	22	34	13
ARL14	11	0	4	0	3	1
	0	0	1	0	2	0
ARL14EP	75	358	63	229	194	238
	282	397	142	107	232	64
ARL14EPL	3	0	3	0	0	0
	0	0	4	0	0	0
ARL15	26	86	26	75	42	72
	61	97	50	19	52	24
ARL16	27	41	11	47	22	68
	92	69	20	16	33	16
ARL17A	81	179	77	107	94	100
	103	147	82	46	94	44
ARL17B	35	85	20	64	56	51
	38	125	47	36	40	12
ARL2	44	245	33	149	107	163
	240	383	124	59	122	41

ARL2BP	66	265	48	211	130	185
	247	301	159	64	124	35
ARL3	11	77	20	51	23	29
	52	62	33	17	32	16
ARL4A	17	59	23	46	32	37
	37	56	30	8	34	10
ARL4C	23	27	20	20	9	16
	7	21	14	0	15	5
ARL4D	9	5	8	5	4	5
	6	7	3	1	3	3
ARL5A	153	663	141	700	407	718
	624	1087	405	318	427	151
ARL5B	33	112	32	89	63	74
	93	115	58	30	83	17
ARL5C	2	0	3	0	1	0
	0	0	0	0	2	0
ARL6	9	21	7	15	15	25
	15	43	18	8	13	6
ARL6IP1	75	332	70	277	149	196
	256	494	213	85	230	79
ARL6IP4	126	675	124	532	244	642
	880	998	321	243	433	147
ARL6IP5	289	1661	266	1195	789	1138
	1252	1764	759	423	774	240
ARL6IP6	24	52	17	45	34	71
	28	65	34	17	29	9
ARL8A	29	96	31	105	47	69
	124	125	73	47	63	21
ARL8B	151	785	148	578	392	536
	599	895	298	196	403	109
ARL9	1	2	5	0	1	0
	1	1	3	0	3	1
ARMC1	66	349	83	293	190	216
	357	509	253	109	251	103
ARMC10	27	141	24	66	53	87
	87	151	71	33	72	20
ARMC12	4	0	4	0	0	0
	0	2	7	1	1	0
ARMC2	38	31	22	33	26	31
	34	48	37	11	38	3
ARMC3	21	0	7	0	5	0
	0	0	15	0	9	0
ARMC4	29	0	21	1	8	1
	1	0	15	0	12	1
ARMC5	25	51	18	31	16	43
	34	50	32	19	31	7
ARMC6	28	58	14	46	17	47
	52	66	36	14	30	5
ARMC7	10	11	5	8	14	8
	7	10	8	1	2	4
ARMC8	199	870	238	815	428	684
	730	896	442	274	577	142
ARMC9	45	16	20	7	14	20
	11	17	30	5	18	5
ARMCX1	64	342	68	219	155	263
	191	483	147	82	176	35
ARMCX2	20	72	24	51	47	50
	40	72	42	25	25	8

ARMCX3	107	502	98	315	305	325
	460	619	186	195	197	102
ARMCX4	36	72	27	54	27	28
	29	87	38	8	39	17
ARMCX5	2	3	2	6	3	1
	4	8	2	1	4	0
ARMCX5-GPRASP2		46	115	35	61	59
	67	63	109	41	30	55
	24					
ARMCX6	26	80	35	35	59	68
	66	116	34	24	31	10
ARMS2	3	0	3	0	0	0
	0	0	3	0	0	0
ARNT	179	692	160	547	365	495
	504	614	277	209	419	109
ARNT2	52	94	35	102	50	64
	86	61	57	28	73	12
ARNTL	31	115	53	78	41	89
	50	60	34	20	37	9
ARNTL2	28	11	19	6	14	9
	9	7	28	5	16	3
ARPC1A	59	245	73	165	135	161
	219	314	127	61	131	47
ARPC1B	15	29	5	21	7	26
	21	34	16	5	13	10
ARPC2	175	1339	165	1036	472	1059
	1067	1043	752	315	688	102
ARPC3	70	370	86	289	148	295
	370	528	217	120	226	57
ARPC4	21	134	24	118	44	91
	96	186	70	31	55	24
ARPC4-TTLL3		23	34	18	33	24
	22	21	46	31	7	28
	4					
ARPC5	26	131	22	110	61	76
	107	202	78	33	69	31
ARPC5L	41	177	23	104	52	136
	178	186	55	18	59	17
ARPP19	497	2067	431	1711	1099	1513
	1612	3049	976	607	1281	446
ARPP21	206	1074	290	545	463	569
	616	1406	433	375	287	352
ARR3	18	3	9	1	4	1
	1	0	8	0	6	1
ARRB1	44	135	45	74	63	94
	112	101	56	29	57	37
ARRB2	20	10	11	11	8	17
	18	32	13	9	13	7
ARRDC1	13	18	10	18	22	22
	15	42	14	8	21	6
ARRDC2	152	389	80	399	323	361
	710	454	398	150	355	126
ARRDC3	252	938	265	655	540	443
	899	1002	550	347	648	260
ARRDC4	71	137	68	154	87	237
	212	133	92	82	71	45
ARRDC5	14	3	3	1	0	0
	0	0	0	0	1	0

ARSA	15	23	3	23	13	11
	18	34	11	9	16	2
ARSB	47	83	36	47	47	80
	107	132	62	30	55	23
ARSD	36	23	12	27	26	31
	6	36	28	13	27	5
ARSE	13	0	7	2	1	0
	0	0	4	0	1	0
ARSF	24	1	11	0	7	0
	0	0	9	0	3	0
ARSG	21	22	27	16	12	21
	17	24	15	3	20	4
ARSH	25	0	5	0	2	0
	0	0	2	0	4	0
ARSI	14	3	3	4	3	5
	0	1	4	0	3	1
ARSJ	16	9	15	1	13	11
	9	12	15	4	16	2
ARSK	24	64	31	53	29	51
	34	61	33	18	29	10
ART1	22	126	28	102	55	96
	124	140	76	28	98	25
ART3	193	946	286	1087	770	1463
	1473	1651	780	431	1023	288
ART4	5	3	6	2	1	0
	1	7	4	3	3	3
ART5	4	14	12	15	8	17
	28	42	20	12	13	6
ARTN	4	1	3	1	3	0
	0	2	2	0	3	1
ARV1	34	190	28	133	73	131
	186	187	100	51	111	28
ARVCF	29	55	18	32	39	52
	30	84	38	16	32	8
ARX	26	139	14	36	32	61
	69	69	31	36	44	3
AS3MT	47	115	37	56	100	71
	128	76	90	58	118	28
ASAH1	255	1112	295	960	624	912
	1024	1870	710	319	946	213
ASAH2	36	18	23	6	16	8
	7	5	20	2	20	1
ASAH2B	7	32	16	24	23	23
	26	43	16	3	12	3
ASAP1	107	360	107	204	153	224
	259	339	147	95	172	70
ASAP2	77	191	54	157	85	106
	104	208	90	48	80	61
ASAP3	58	265	64	163	159	253
	122	219	58	89	112	48
ASB1	90	297	74	222	137	186
	256	279	135	75	171	45
ASB10	47	177	36	152	88	191
	232	177	97	76	114	38
ASB11	327	1203	209	1040	866	1155
	1041	1873	590	514	1027	317
ASB12	26	134	23	122	87	131
	140	161	72	23	73	20

ASB13	19	50	15	24	16	34
	43	48	28	7	39	7
ASB14	139	688	120	748	229	487
	562	627	358	225	472	65
ASB15	202	1235	221	909	532	831
	1290	1431	615	322	819	128
ASB16	28	104	28	81	46	64
	93	116	82	38	78	28
ASB17	13	0	5	0	0	0
	0	0	4	0	2	0
ASB18	6	38	13	27	22	47
	32	62	26	9	28	5
ASB2	436	1526	234	1517	1089	1963
	1895	1432	700	691	856	360
ASB3	6	18	5	10	13	9
	15	12	3	11	8	3
ASB4	165	638	176	477	317	493
	599	963	359	204	486	122
ASB5	611	4005	687	2972	2001	3388
	3596	3443	1737	1087	2259	470
ASB6	22	49	13	34	34	35
	24	68	25	10	22	9
ASB7	50	102	56	63	68	68
	82	139	61	42	69	23
ASB8	284	1137	204	974	723	1066
	1165	1714	723	431	934	269
ASB9	11	3	2	3	3	1
	1	5	0	4	4	1
ASCC1	74	227	89	219	127	228
	248	298	137	82	185	59
ASCC2	67	275	56	164	121	241
	183	295	104	78	140	47
ASCC3	160	727	274	461	284	473
	456	635	344	201	426	124
ASCL1	14	0	5	1	2	0
	0	0	5	0	1	0
ASCL2	1	0	2	1	1	0
	0	0	1	1	3	0
ASCL3	1	0	1	0	0	0
	0	0	3	0	1	0
ASCL4	10	0	2	0	0	0
	0	0	1	0	5	0
ASCL5	1	0	1	0	0	0
	0	0	0	1	0	0
ASF1A	74	384	68	253	145	262
	329	435	176	90	227	67
ASF1B	6	0	1	1	1	1
	0	0	5	1	1	0
ASGR1	2	0	4	0	1	1
	0	0	4	0	0	0
ASGR2	3	0	6	0	0	2
	0	0	2	0	2	0
ASH1L	586	3588	768	2528	1590	2669
	2371	3142	1340	1053	1679	648
ASH2L	64	218	51	187	112	177
	213	227	83	71	113	35
ASIC1	14	7	6	1	4	1
	0	1	8	0	7	0

ASIC2	21	0	14	1	7	0
	2	0	9	0	5	0
ASIC3	14	17	13	8	5	12
	5	10	10	6	11	1
ASIC4	10	0	5	2	2	0
	0	0	8	0	2	0
ASIC5	16	0	6	0	6	0
	0	0	3	0	3	0
ASIP	1	0	1	0	1	0
	0	0	2	0	0	0
ASL	10	29	4	15	6	20
	19	40	12	3	8	7
ASMT (NC_000023 1714347..1761974)				1	0	1
	0	1	1	1	1	2
	0	1	0			
ASMT (NC_000024 1664347..1711974)				7	0	2
	0	0	0	0	0	2
	1	0	0			
ASMTL (NC_000023 1522031..1572655)				7	22	6
	14	9	17	26	23	9
	2	4	5			
ASMTL (NC_000024 1472031..1522655)				4	14	3
	17	10	16	22	13	12
	3	8	14			
ASNA1	34	86	20	66	57	89
	89	160	68	23	80	16
ASNS	29	67	19	24	20	58
	19	59	42	13	46	3
ASNSD1	121	695	124	500	392	523
	690	949	388	229	472	105
ASPA	43	296	58	186	113	113
	258	267	112	49	123	19
ASPDH	4	1	0	0	1	0
	0	2	3	0	0	0
ASPG	4	0	9	3	0	0
	0	0	3	0	0	0
ASPH	707	4141	752	3218	2024	3112
	3061	3490	1690	1062	1902	750
ASPHD1	7	0	8	0	1	0
	0	0	3	0	3	0
ASPHD2	12	0	9	0	3	1
	0	1	6	0	3	0
ASPM	93	5	49	11	36	10
	8	14	56	4	53	3
ASPN	33	144	23	97	59	92
	117	136	79	31	75	36
ASPRV1	21	24	13	17	6	22
	7	10	5	4	9	5
ASPSCR1	21	112	26	45	40	79
	49	89	46	13	62	13
ASRGL1	23	16	7	9	8	14
	15	26	14	6	25	5
ASS1	39	271	45	101	79	76
	95	271	44	31	98	35
ASTE1	22	43	21	45	34	28
	44	56	22	18	38	8
ASTL	7	0	9	1	0	1
	0	0	4	0	3	0

ASTN1	63	1	39	0	9	1
	2	1	20	1	8	0
ASTN2	95	136	102	81	52	91
	49	73	90	11	81	12
ASUN	85	300	69	186	171	193
	219	250	151	83	169	49
ASXL1	98	365	96	268	220	275
	306	469	210	143	236	73
ASXL2	172	683	116	446	343	448
	481	473	162	171	249	114
ASXL3	50	0	45	4	19	1
	2	0	39	0	14	1
ASZ1	15	0	16	0	9	0
	0	0	15	0	6	0
ATAD1	184	963	189	690	404	655
	795	1091	453	240	571	141
ATAD2	45	48	43	35	32	26
	42	131	66	20	59	29
ATAD2B	178	571	178	390	235	433
	341	585	290	175	324	142
ATAD3A	27	115	22	65	43	79
	77	106	45	30	64	15
ATAD3B	9	38	11	25	13	25
	39	48	25	12	28	11
ATAD3C	17	1	13	0	1	3
	0	1	11	0	3	0
ATAD5	46	35	26	27	34	24
	21	39	49	17	36	10
ATAT1	36	14	12	14	11	10
	18	8	10	5	19	0
ATCAY	30	4	12	3	7	3
	2	2	16	0	11	4
ATE1	54	271	68	234	122	232
	200	341	139	82	174	70
ATF1	115	491	133	387	230	449
	396	467	217	125	314	90
ATF2	136	535	152	452	276	457
	432	632	340	200	416	120
ATF3	12	12	23	13	14	8
	30	28	28	17	51	4
ATF4	571	2744	300	1579	1540	2663
	2680	3067	1186	1104	1229	509
ATF5	23	31	8	19	9	37
	15	27	24	9	9	8
ATF6	113	450	104	323	214	285
	327	528	239	163	273	79
ATF6B	40	82	22	54	47	66
	69	114	47	30	58	30
ATF7	108	331	99	259	207	217
	267	295	104	73	144	59
ATF7IP	186	1084	260	847	414	619
	667	895	321	296	458	242
ATF7IP2	29	25	14	18	17	16
	33	23	30	14	27	4
ATG10	26	25	13	32	29	48
	37	70	25	17	26	6
ATG12	110	393	89	339	190	337
	350	461	214	132	219	99

ATG13	62	112	32	119	72	75
	119	167	90	58	111	33
ATG14	129	520	128	291	222	336
	386	750	325	180	345	90
ATG16L1	97	296	84	193	154	260
	233	421	174	94	224	69
ATG16L2	17	41	24	17	13	12
	20	46	11	9	25	5
ATG2A	43	90	25	58	30	52
	56	109	49	40	62	17
ATG2B	272	1042	305	978	509	792
	807	1273	678	348	851	207
ATG3	50	280	40	177	107	175
	192	237	111	78	138	52
ATG4A	69	217	44	186	80	163
	176	258	118	65	89	29
ATG4B	40	139	39	96	79	88
	110	166	64	21	75	20
ATG4C	47	102	44	76	52	89
	83	109	69	36	60	9
ATG4D	23	80	15	69	46	99
	95	98	71	40	54	21
ATG5	77	253	68	217	140	172
	205	324	168	95	171	43
ATG7	85	177	72	195	81	142
	131	252	130	64	180	37
ATG9A	83	329	59	250	172	301
	284	422	210	130	222	75
ATG9B	15	0	8	1	2	2
	0	1	7	0	5	0
ATHL1	10	24	4	10	7	10
	10	23	13	11	15	9
ATIC	41	212	36	94	66	59
	146	157	51	46	68	21
ATL1	39	16	16	15	12	11
	7	14	18	4	19	1
ATL2	224	949	225	980	727	778
	764	1246	554	377	539	196
ATL3	57	151	54	126	86	99
	110	162	77	54	78	51
ATM	295	1035	365	923	553	776
	759	1323	573	341	699	226
ATMIN	131	472	93	424	286	476
	430	563	239	154	286	99
ATN1	40	185	38	105	80	117
	147	121	28	53	67	46
ATOH1	3	0	0	0	0	0
	0	0	0	0	2	0
ATOH7	3	1	2	1	0	0
	0	0	1	0	0	0
ATOH8	29	64	23	36	25	42
	72	84	59	23	49	27
ATOX1	5	15	8	15	6	10
	11	16	10	7	9	4
ATP10A	21	14	13	8	9	3
	8	21	16	1	11	2
ATP10B	70	0	39	5	12	0
	1	0	35	0	21	0

ATP10D	57	110	42	82	60	57
	77	117	80	27	86	35
ATP11A	133	530	122	422	211	434
	416	372	261	123	254	90
ATP11B	166	550	191	497	355	484
	457	1008	355	230	415	146
ATP11C	91	155	69	150	97	172
	107	184	84	39	83	43
ATP12A	27	0	10	0	2	0
	0	0	11	0	9	0
ATP13A1	24	56	18	36	35	33
	53	64	33	18	36	24
ATP13A2	11	47	15	23	29	37
	31	39	19	10	23	11
ATP13A3	118	412	141	381	240	334
	395	418	228	116	302	118
ATP13A4	37	7	28	3	14	14
	0	0	33	0	19	1
ATP13A5	33	4	23	2	11	2
	0	0	21	0	13	0
ATP1A1	77	455	115	280	185	270
	265	227	179	63	169	61
ATP1A2	1941	9494	1435	7662	5366	6587
	9738	9416	4177	3370	4534	1978
ATP1A3	12	0	15	2	4	0
	0	1	10	1	5	0
ATP1A4	43	10	28	9	20	23
	4	38	25	6	19	1
ATP1B1	491	2763	356	1957	1435	1568
	3723	3124	1575	1056	2013	657
ATP1B2	18	15	15	7	9	1
	13	9	7	2	2	1
ATP1B3	27	140	20	114	59	101
	82	120	61	25	48	24
ATP1B4	539	1959	371	1097	870	931
	965	2481	507	508	993	404
ATP2A1	3928	35737	4427	32465	15398	32138
	23276	16409	15434	6925	17383	3665
ATP2A2	7434	25767	3926	11297	14212	10576
	23089	47598	5931	10603	14699	7994
ATP2A3	16	24	15	11	11	9
	16	17	12	3	9	3
ATP2B1	186	776	204	633	423	755
	508	856	358	247	405	163
ATP2B2	186	1304	201	893	500	749
	781	736	410	224	503	125
ATP2B3	33	1	9	0	6	0
	0	0	4	0	2	0
ATP2B4	256	1208	179	755	587	649
	949	1105	521	329	544	209
ATP2C1	317	1252	347	1158	735	1134
	951	1243	759	300	752	181
ATP2C2	26	0	18	1	8	1
	0	1	11	1	6	0
ATP4A	23	0	18	0	7	0
	0	0	9	0	9	0
ATP4B	7	0	0	0	1	1
	0	0	4	0	1	0

ATP5A1	1269	8924	1206	6952	3001	4640
	8725	9225	4064	1975	4834	992
ATP5B	1037	6164	851	5609	2245	3936
	7085	7712	3545	1728	4276	929
ATP5C1	429	1916	276	1499	698	1170
	2245	2810	1354	549	1325	326
ATP5D	65	480	48	332	136	319
	655	603	259	151	291	66
ATP5E	604	4281	504	2242	1417	3205
	6946	7473	1736	1198	2415	521
ATP5F1	341	1919	295	1404	654	947
	2364	2601	1033	499	1225	337
ATP5G1	75	287	22	199	116	125
	398	515	159	64	194	33
ATP5G2	260	1054	193	737	383	924
	1415	2169	711	328	683	202
ATP5G3	136	718	83	618	242	333
	789	794	386	176	422	115
ATP5H	373	2533	246	1336	851	1335
	3419	4091	1090	753	1318	336
ATP5I	186	878	99	609	263	467
	1329	1741	489	213	549	126
ATP5J	186	1264	110	762	369	694
	1438	1341	489	232	629	150
ATP5J2	171	615	78	300	269	313
	674	914	239	149	352	70
ATP5J2-PTCD1		53	134	26	86	75
	118	68	129	68	45	76
	20					
ATP5L	185	1061	134	740	376	624
	1197	1610	512	272	673	159
ATP5L2	0	0	0	1	0	0
	0	0	0	0	0	0
ATP5O	316	1849	262	1554	591	996
	2553	2969	1047	466	1240	284
ATP5S	16	79	20	80	46	51
	76	106	50	19	57	19
ATP5SL	41	142	19	77	59	67
	117	143	73	42	54	29
ATP6AP1	37	161	23	118	67	103
	103	156	59	33	86	25
ATP6AP1L	8	28	5	9	12	8
	10	23	11	5	11	0
ATP6AP2	57	176	43	207	86	165
	143	248	105	48	106	43
ATP6V0A1	165	570	141	365	236	386
	477	773	365	190	326	127
ATP6V0A2	63	186	53	176	108	157
	149	300	136	70	161	61
ATP6V0A4	24	1	17	0	6	0
	0	0	13	0	6	0
ATP6V0B	21	66	6	85	37	40
	60	145	60	19	50	16
ATP6V0C	5	62	7	33	14	33
	36	29	6	15	20	10
ATP6V0D1	47	263	47	166	100	159
	160	233	115	64	99	35

ATP6V0D2	16	0	7	0	1	0
	0	0	7	0	4	0
ATP6V0E1	72	272	47	206	138	193
	213	457	160	87	174	69
ATP6V0E2	16	21	10	27	9	22
	30	33	13	7	18	2
ATP6V1A	98	356	101	304	161	267
	271	379	167	89	222	58
ATP6V1B1	8	0	10	0	2	0
	0	2	6	0	1	1
ATP6V1B2	95	510	92	327	160	277
	422	522	209	119	298	78
ATP6V1C1	88	306	84	282	158	228
	262	405	228	95	251	71
ATP6V1C2	24	7	15	3	7	8
	9	10	10	0	11	0
ATP6V1D	61	278	42	186	115	196
	230	317	131	55	165	41
ATP6V1E1	119	726	118	500	268	546
	626	897	427	211	403	112
ATP6V1E2	12	15	12	5	12	16
	8	19	6	6	7	5
ATP6V1F	50	170	35	131	89	135
	254	355	119	66	123	32
ATP6V1G1	117	596	85	474	215	444
	591	915	308	165	409	89
ATP6V1G2	10	9	8	7	4	1
	5	5	11	3	2	1
ATP6V1G3	5	0	4	0	0	0
	0	0	4	0	3	0
ATP6V1H	76	338	66	259	162	246
	299	396	195	102	220	47
ATP7A	88	264	67	180	127	192
	178	273	129	80	163	59
ATP7B	57	97	38	83	46	89
	81	106	53	23	50	17
ATP8A1	1020	2626	501	1775	1572	2388
	2037	4467	1842	1420	2216	635
ATP8A2	50	5	32	6	11	1
	1	6	31	1	23	0
ATP8B1	67	125	53	65	70	67
	127	140	84	48	112	48
ATP8B2	66	91	39	81	39	57
	45	112	54	18	45	21
ATP8B3	18	1	14	1	2	0
	0	0	5	0	3	0
ATP8B4	59	39	58	37	37	39
	39	82	76	14	59	21
ATP9A	128	447	110	288	160	261
	290	386	232	104	255	117
ATP9B	87	345	97	297	164	260
	250	349	179	121	189	61
ATPAF1	203	1204	175	984	533	700
	1379	1122	485	263	597	148
ATPAF2	15	98	17	47	35	37
	77	93	43	17	51	13
ATPBD4	55	202	64	169	77	129
	138	217	133	54	147	35

ATPIF1	177	1090	89	589	538	685
	1197	1089	463	237	539	127
ATR	116	417	125	309	168	234
	278	484	246	144	290	85
ATRAID	64	345	50	190	140	182
	374	435	166	83	164	71
ATRIP	21	42	16	17	19	22
	25	33	29	12	28	12
ATRN	178	549	160	434	314	445
	378	594	303	177	355	84
ATRNL1	84	439	170	1676	101	446
	418	73	678	17	84	6
ATRX	462	2823	499	1932	1312	1651
	1723	2063	811	832	1110	467
ATXN1	276	1111	319	904	701	909
	840	1342	550	412	780	380
ATXN10	119	643	107	488	284	361
	494	642	266	135	311	96
ATXN1L	94	314	70	215	145	213
	169	324	126	91	193	61
ATXN2	101	483	87	269	173	208
	348	349	133	112	149	82
ATXN2L	36	200	46	98	59	67
	141	178	68	43	70	37
ATXN3	105	304	110	205	152	240
	271	432	135	101	209	84
ATXN3L	12	0	1	0	2	0
	0	0	1	0	1	0
ATXN7	113	492	146	362	278	343
	465	497	216	172	226	81
ATXN7L1	49	91	42	56	56	87
	64	86	53	27	54	7
ATXN7L2	36	129	35	109	53	100
	97	110	46	21	62	19
ATXN7L3	42	111	28	82	58	91
	54	106	44	26	43	12
ATXN7L3B	134	544	114	501	278	431
	545	452	295	143	327	93
AUH	76	251	65	227	124	195
	284	345	160	56	231	49
AUNIP	14	2	10	0	3	0
	0	3	6	0	2	0
AUP1	59	291	51	183	132	188
	276	405	159	60	132	41
AURKA	28	81	25	51	27	50
	265	82	56	18	76	14
AURKAIP1	68	410	28	224	95	197
	394	449	179	95	163	46
AURKB	6	0	1	2	1	2
	0	3	2	1	6	0
AURKC	9	10	6	11	10	5
	6	7	12	1	7	2
AUTS2	55	253	59	147	90	155
	137	177	85	43	94	32
AVEN	18	106	37	64	44	75
	80	83	46	30	62	22
AVIL	114	361	82	169	203	343
	185	295	128	71	118	40

AVL9	36	134	47	100	75	73
	94	173	84	40	81	27
AVP	3	0	2	0	0	0
	0	0	1	0	0	1
AVPI1	5	6	6	8	7	7
	7	2	2	1	5	1
AVPR1A	65	116	32	41	62	45
	76	107	56	29	62	14
AVPR1B	10	0	2	0	0	0
	0	0	4	0	1	0
AVPR2	3	5	1	1	3	5
	11	11	2	0	4	2
AWAT1	7	0	1	0	2	0
	0	1	4	0	2	0
AWAT2	11	0	5	0	0	0
	0	0	5	0	0	1
AXDND1	38	0	22	1	9	1
	0	1	25	0	10	0
AXIN1	32	104	31	59	48	69
	79	134	61	40	46	17
AXIN2	56	148	26	71	75	111
	127	155	78	48	85	22
AXL	59	136	29	137	75	110
	50	171	59	29	82	36
AZGP1	8	1	5	4	4	0
	7	0	10	2	3	2
AZI1	24	58	26	31	19	36
	25	54	25	21	46	8
AZI2	85	273	82	252	108	270
	284	372	147	106	198	43
AZIN1	259	1315	279	870	597	888
	754	1148	433	288	550	166
AZU1	4	0	0	0	0	0
	0	0	2	0	1	0
B2M	493	3322	538	2724	1342	2745
	3390	6650	2558	1370	2903	598
B3GALNT1	18	19	26	24	19	17
	14	28	30	5	23	7
B3GALNT2	228	853	234	952	485	872
	825	1445	590	392	813	228
B3GALT1	17	4	11	26	19	27
	52	133	47	32	104	8
B3GALT2	15	7	7	7	8	12
	11	6	21	5	2	3
B3GALT4	4	7	0	6	6	9
	4	13	4	1	5	1
B3GALT5	16	3	7	5	1	0
	2	4	7	1	1	0
B3GALT6	11	53	26	44	12	36
	40	60	39	12	38	5
B3GALTL	45	165	69	162	103	157
	104	127	93	31	71	26
B3GAT1	16	0	9	0	1	0
	0	0	9	0	5	0
B3GAT2	8	1	1	1	0	0
	2	3	2	0	1	0
B3GAT3	3	15	3	13	14	15
	8	17	7	3	4	0

B3GNT1	44	151	37	145	96	149
	196	218	97	52	86	20
B3GNT2	16	57	16	58	25	35
	34	54	28	16	37	19
B3GNT3	8	0	5	0	0	0
	0	0	3	0	1	0
B3GNT4	7	0	0	0	0	0
	0	0	2	0	0	0
B3GNT5	40	79	36	79	55	54
	118	140	68	49	108	28
B3GNT6	4	0	5	0	4	0
	0	0	1	0	0	1
B3GNT7	21	2	7	6	7	3
	0	10	3	3	4	1
B3GNT8	3	0	3	1	1	2
	0	2	1	1	0	0
B3GNT9	13	14	7	12	2	15
	6	13	5	0	9	3
B3GNTL1	11	15	11	11	10	4
	9	9	13	2	9	2
B4GALNT1	14	12	10	26	11	8
	15	18	18	5	19	5
B4GALNT2	23	0	5	0	3	0
	0	0	7	0	2	0
B4GALNT3	21	5	20	12	11	11
	1	17	20	3	19	3
B4GALNT4	14	0	5	1	1	0
	0	0	2	0	2	0
B4GALT1	46	252	29	133	79	140
	215	206	95	50	109	50
B4GALT2	42	117	20	78	36	83
	91	109	46	36	59	25
B4GALT3	41	56	18	53	36	46
	54	82	39	15	39	8
B4GALT4	27	63	26	64	28	74
	56	71	40	21	37	10
B4GALT5	171	610	130	303	296	364
	456	753	236	195	306	116
B4GALT6	38	64	44	93	52	57
	43	41	32	13	34	7
B4GALT7	8	28	10	22	18	26
	19	42	12	9	19	8
B9D1	18	4	4	4	5	4
	2	7	6	3	3	0
B9D2	2	0	0	0	0	0
	2	1	2	0	0	0
BAALC	30	45	16	23	29	21
	39	62	34	16	33	7
BAAT	33	10	17	12	18	31
	10	28	17	9	27	4
BABAM1	81	362	49	187	142	233
	362	446	154	113	197	54
BACE1	57	202	69	154	67	189
	157	181	129	39	109	27
BACE2	15	65	13	32	25	29
	45	57	31	14	25	20
BACH1	147	525	162	519	301	472
	659	700	383	243	477	146

BACH2	93	177	74	181	113	157
	143	137	125	66	76	33
BAD	14	18	4	35	7	37
	28	38	24	6	18	3
BAG1	129	545	80	320	171	549
	674	649	253	156	289	87
BAG2	88	376	65	225	172	247
	219	286	141	54	144	37
BAG3	253	1599	211	1152	729	953
	1314	1160	470	457	658	347
BAG4	104	321	109	269	187	262
	300	425	210	102	244	65
BAG5	72	354	77	224	161	201
	243	354	114	108	195	78
BAG6	143	616	107	420	230	362
	487	659	268	186	351	91
BAGE2	82	1	55	0	32	0
	0	5	77	1	42	1
BAHCC1	29	81	23	43	23	43
	34	65	27	16	19	14
BAHD1	31	30	27	24	20	33
	42	54	38	12	25	8
BAI1	6	1	15	1	6	0
	0	0	7	0	5	0
BAI2	22	8	16	3	10	1
	1	7	14	3	5	2
BAI3	49	19	57	22	32	17
	19	15	49	8	42	7
BAIAP2	20	13	12	15	16	7
	7	22	12	0	7	3
BAIAP2L1	25	33	7	13	12	18
	19	33	27	7	18	2
BAIAP2L2	8	3	4	1	2	5
	1	1	5	0	5	1
BAIAP3	10	14	7	1	6	7
	8	5	10	2	14	2
BAK1	10	30	10	20	11	19
	23	37	15	10	19	4
BAMBI	6	13	7	26	8	12
	6	17	12	2	19	4
BANF1	26	73	10	45	40	59
	131	71	39	23	61	14
BANF2	3	0	4	0	0	0
	0	0	1	0	2	0
BANK1	20	6	21	5	11	4
	1	2	20	2	11	3
BANP	22	84	30	47	31	41
	48	71	39	15	43	15
BAP1	64	250	52	173	111	176
	187	312	116	93	147	40
BARD1	96	210	98	110	167	360
	216	370	184	116	253	73
BARHL1	1	0	0	0	1	0
	0	0	1	0	0	0
BARHL2	7	0	5	0	2	0
	0	0	2	0	2	0
BARX1	2	0	1	0	0	0
	0	0	2	0	0	0

BARX2	14	0	7	0	3	0
	0	0	4	0	1	0
BASP1	14	12	6	7	6	15
	6	25	4	3	11	6
BATF	5	0	1	1	0	1
	2	0	1	1	0	0
BATF2	9	13	5	14	5	11
	21	22	14	5	11	2
BATF3	7	3	0	4	3	0
	3	6	3	0	1	1
BAX	7	11	2	5	3	9
	7	12	7	4	4	0
BAZ1A	73	188	75	163	126	179
	127	217	107	76	123	50
BAZ1B	195	1038	230	661	583	718
	600	1058	393	393	516	242
BAZ2A	155	514	137	332	229	309
	432	400	195	162	228	105
BAZ2B	345	1566	411	1165	670	1235
	1268	1620	716	671	1033	336
BBC3	10	10	14	12	8	11
	8	17	6	2	10	3
BBIP1	31	80	29	85	54	72
	81	126	45	41	52	20
BBOX1	18	2	3	1	3	0
	0	0	8	0	0	0
BBS1	29	68	17	41	27	60
	40	87	34	21	44	15
BBS10	57	175	55	144	89	128
	135	254	104	54	84	49
BBS12	9	7	7	13	7	7
	8	11	11	7	12	2
BBS2	94	473	110	411	219	370
	532	643	300	145	425	81
BBS4	60	155	39	168	83	124
	118	222	108	48	126	45
BBS5	34	105	25	59	36	64
	68	125	60	37	95	18
BBS7	67	145	46	73	68	104
	102	114	65	33	105	26
BBS9	75	152	59	116	77	125
	126	154	69	51	128	38
BBX	165	681	185	504	410	621
	385	682	319	178	324	167
BCAM	21	83	19	44	31	27
	43	43	37	22	31	17
BCAN	12	0	5	1	3	0
	1	0	7	0	4	0
BCAP29	142	820	149	698	368	776
	725	964	392	281	553	193
BCAP31	49	292	39	155	83	151
	179	254	80	42	105	25
BCAR1	61	181	38	118	91	115
	178	136	75	68	106	54
BCAR3	85	207	48	78	69	65
	231	190	66	58	105	29
BCAS1	36	4	13	2	11	2
	0	1	17	1	9	1

BCAS2	231	974	221	718	479	1000
	592	797	350	204	370	104
BCAS3	91	272	81	185	112	192
	172	253	166	90	201	70
BCAS4	9	5	3	1	0	0
	3	0	0	1	1	0
BCAT1	63	50	30	56	84	86
	68	173	97	28	85	25
BCAT2	12	44	14	30	24	28
	43	77	27	18	44	12
BCCIP	101	470	80	336	254	396
	371	640	279	160	330	100
BCDIN3D	26	39	17	36	26	50
	36	49	28	8	24	2
BCHE	22	66	26	61	35	34
	56	53	28	27	40	5
BCKDHA	65	294	38	189	158	171
	267	371	125	114	214	51
BCKDHB	45	155	48	77	58	60
	130	213	74	33	110	29
BCKDK	59	206	38	143	86	145
	292	206	118	91	148	40
BCL10	24	103	24	71	44	47
	59	79	40	21	54	21
BCL11A	31	9	12	1	10	0
	0	0	22	1	15	0
BCL11B	50	9	60	3	32	6
	3	11	15	1	27	1
BCL2	152	631	162	516	479	541
	732	742	374	225	536	141
BCL2A1	16	4	6	1	2	0
	1	3	7	1	3	0
BCL2L1	22	129	25	71	49	68
	97	117	44	28	69	16
BCL2L10	2	0	1	0	0	0
	0	0	0	0	0	0
BCL2L11	63	151	66	139	73	124
	156	191	85	48	90	39
BCL2L12	15	12	7	8	11	11
	13	18	12	1	9	2
BCL2L13	228	1032	168	773	469	712
	824	1041	502	342	672	200
BCL2L14	13	0	12	0	2	1
	0	2	6	0	2	0
BCL2L15	23	17	11	11	9	7
	1	16	12	4	13	3
BCL2L2	0	11	4	2	2	12
	4	10	2	1	0	2
BCL2L2-PABPN1		92	328	70	236	158
	281	282	350	161	115	172
	70					
BCL3	6	5	2	5	9	7
	13	8	4	3	9	2
BCL6	196	980	237	673	706	789
	1514	3218	989	859	1335	344
BCL6B	27	168	21	76	52	85
	102	66	70	25	44	11

BCL7A	32	84	21	55	33	49
	80	97	37	31	64	10
BCL7B	93	250	51	170	142	302
	344	302	113	112	166	67
BCL7C	11	36	5	25	20	20
	42	59	30	7	25	7
BCL9	59	144	61	116	67	97
	152	90	51	31	82	14
BCL9L	48	223	36	105	77	130
	113	85	37	37	66	19
BCLAF1	427	1760	509	1518	966	1510
	1403	2170	900	667	1134	444
BCMO1	23	1	9	1	4	0
	0	0	11	0	0	0
BCO2	96	522	40	137	233	196
	366	547	186	141	218	100
BCOR	89	418	75	264	227	271
	245	287	141	106	180	73
BCORL1	39	50	14	26	13	32
	20	20	15	12	12	9
BCR	58	56	22	30	26	17
	25	51	38	16	32	20
BCS1L	25	83	33	54	51	67
	73	121	49	26	56	12
BDH1	33	68	12	12	24	11
	48	130	46	19	52	9
BDH2	45	112	27	84	54	80
	111	162	102	20	96	11
BDKRB1	6	0	2	0	0	0
	0	0	3	0	1	0
BDKRB2	26	14	15	12	3	0
	3	14	10	0	11	1
BDNF	58	1	23	4	18	2
	2	46	31	3	11	5
BDP1	330	1246	312	828	619	1015
	867	1111	517	493	725	291
BEAN1	6	1	8	1	0	0
	0	0	10	0	0	1
BECN1	151	794	132	671	292	661
	738	830	390	226	449	131
BEGAIN	6	1	1	1	0	0
	0	3	2	0	1	2
BEND2	31	1	14	1	9	1
	2	0	8	0	5	0
BEND3	36	31	28	27	15	16
	21	20	22	7	10	5
BEND4	40	3	31	4	6	0
	1	1	25	0	12	0
BEND5	2	10	1	9	1	12
	7	8	4	3	2	7
BEND6	20	1	6	1	9	4
	1	1	5	1	9	1
BEND7	45	87	27	65	71	84
	109	110	52	36	43	27
BEST1	16	2	8	8	5	2
	0	7	8	1	10	0
BEST2	6	0	2	0	0	0
	0	0	5	0	4	0

BEST3	149	485	196	240	112	191
	350	720	346	281	423	183
BEST4	8	2	1	1	2	0
	0	1	3	0	0	0
BET1	28	99	34	76	41	59
	117	115	78	23	61	14
BET1L	13	45	12	28	13	24
	39	31	28	15	21	11
BET3L	7	0	11	2	1	1
	1	0	2	0	9	0
BEX1	9	0	2	1	1	0
	0	1	5	0	0	0
BEX2	8	9	7	3	3	18
	6	22	12	1	13	3
BEX4	10	36	7	21	20	23
	15	27	10	3	15	1
BEX5	3	1	2	0	4	4
	1	1	0	0	1	0
BFAR	45	241	54	176	134	178
	223	320	122	90	146	33
BFSP1	32	6	10	2	13	7
	9	7	12	0	5	1
BFSP2	5	0	5	0	0	0
	0	0	5	0	5	0
BGLAP	0	0	0	0	2	0
	0	0	0	0	0	0
BGN	14	22	5	19	14	16
	18	18	10	2	3	11
BHLHA15	3	0	0	0	1	2
	0	0	0	0	0	0
BHLHB9	33	52	31	66	34	42
	55	51	37	17	40	9
BHLHE22	16	9	8	1	14	0
	3	1	6	1	6	0
BHLHE40	138	935	125	492	306	542
	1038	638	416	260	272	133
BHLHE41	160	1302	106	1003	462	702
	801	872	429	222	439	124
BHMT	18	1	9	3	5	0
	0	2	11	0	7	0
BHMT2	48	141	36	144	54	73
	228	153	74	48	58	32
BICC1	57	209	62	152	127	99
	131	321	85	77	111	59
BICD1	45	113	45	71	23	65
	55	49	43	9	34	17
BICD2	96	409	85	271	252	361
	252	493	155	123	215	59
BID	11	13	10	15	11	14
	19	17	13	9	17	4
BIK	1	1	2	0	2	0
	0	1	0	0	0	0
BIN1	1408	9100	1681	7136	3763	9484
	7079	10312	4739	2781	5149	1387
BIN2	28	19	8	9	18	19
	10	10	22	3	14	3
BIN3	19	39	9	18	15	20
	22	45	16	5	24	9

BIRC2	164	788	174	606	389	613
	599	942	377	230	458	155
BIRC3	51	143	79	134	53	127
	81	103	84	32	68	19
BIRC5	5	0	4	1	0	4
	1	2	2	0	4	0
BIRC6	905	3605	953	3160	1809	2878
	2454	4238	1843	1179	2356	779
BIRC7	5	0	2	0	2	0
	0	0	2	0	1	0
BIRC8	8	0	7	0	0	0
	0	0	6	0	2	0
BIVM	105	485	83	329	243	343
	376	545	213	155	253	76
BIVM-ERCC5	120	348	103	259	238	372
	253	389	256	164	236	103
BLCAP	252	1315	314	897	458	1188
	1187	1818	762	353	655	203
BLID	2	1	3	0	1	0
	0	0	0	0	2	1
BLK	17	2	2	0	0	2
	0	1	6	1	4	1
BLM	35	7	19	4	5	3
	9	6	28	5	16	0
BLMH	41	175	37	102	76	123
	132	145	83	40	94	21
BLNK	13	11	12	4	13	11
	0	5	13	1	5	2
BLOC1S1	66	338	43	215	190	283
	265	595	179	123	220	41
BLOC1S2	44	199	43	188	83	199
	194	212	95	38	113	28
BLOC1S3	13	14	5	1	10	8
	12	17	9	3	3	3
BLOC1S4	2	10	2	13	13	6
	15	16	7	4	7	2
BLOC1S5	17	113	27	110	68	90
	93	165	57	40	64	31
BLOC1S6	141	541	98	422	275	324
	376	606	223	149	303	124
BLVRA	46	154	30	133	84	148
	169	156	89	64	86	32
BLVRB	59	286	35	141	104	128
	298	473	182	91	170	36
BLZF1	47	172	42	117	100	136
	167	185	92	50	122	40
BMF	21	13	18	21	16	30
	12	16	20	1	12	6
BMI1	120	368	89	427	252	272
	341	329	91	106	192	65
BMP1	20	57	13	32	17	38
	25	60	36	10	31	6
BMP10	17	0	7	0	2	0
	0	0	2	0	5	0
BMP15	6	0	0	0	1	0
	0	0	2	0	0	0
BMP2	9	23	15	15	17	10
	16	32	13	6	13	5

BMP2K	71	236	73	176	126	172
	104	186	106	53	112	51
BMP3	33	0	19	27	10	0
	3	1	18	0	8	1
BMP4	13	40	13	42	29	34
	24	58	22	19	18	13
BMP5	42	165	46	160	82	112
	99	192	110	50	91	22
BMP6	26	27	14	14	13	29
	36	39	35	18	36	17
BMP7	20	1	13	12	3	3
	2	5	19	0	7	0
BMP8A	23	11	9	3	0	9
	3	8	6	4	9	1
BMP8B	8	7	3	2	3	0
	1	2	5	3	8	3
BMPER	40	66	28	47	51	68
	31	118	37	21	50	18
BMPR1A	152	747	153	693	378	624
	461	670	383	195	425	144
BMPR1B	106	176	102	155	141	164
	145	198	173	55	128	37
BMPR2	261	1263	227	979	592	846
	1045	1299	531	332	639	264
BMS1	212	1156	251	702	548	715
	837	925	453	365	598	179
BMX	35	14	8	10	16	7
	13	8	11	4	11	7
BNC1	26	2	17	0	0	5
	0	4	11	0	8	2
BNC2	265	1658	210	1485	715	1445
	961	869	736	323	587	243
BNIP1	25	41	14	49	28	51
	36	92	38	19	44	8
BNIP2	124	603	129	565	260	405
	474	638	302	160	365	98
BNIP3	205	1218	136	595	458	532
	683	1671	407	192	444	162
BNIP3L	107	641	90	386	207	520
	297	577	198	106	247	98
BNIPL	12	4	13	1	5	11
	5	5	12	2	13	0
BOC	61	141	28	150	41	106
	58	96	63	34	50	18
BOD1	82	373	89	290	204	375
	406	464	221	110	235	73
BOD1L1	250	1108	297	724	561	801
	943	1024	393	381	614	240
BOD1L2	22	0	6	0	5	0
	0	1	10	0	1	0
BOK	10	17	12	7	5	17
	12	6	5	2	3	4
BOLA1	20	50	12	44	19	38
	70	65	34	18	39	9
BOLA2	5	21	9	21	17	22
	38	52	20	10	16	4
BOLA2B	20	33	2	22	4	22
	24	61	10	6	8	8

BOLA3	26	189	16	108	42	99
	253	329	85	37	87	27
BOLL	45	82	30	82	86	64
	75	90	60	41	68	11
BOP1	16	77	14	45	33	58
	68	78	40	23	49	11
BORA	17	15	10	20	15	16
	15	23	19	3	15	4
BPGM	59	316	61	216	133	308
	249	213	126	122	136	45
BPHL	25	102	24	77	42	82
	159	134	64	33	76	22
BPI	16	2	9	1	5	0
	1	0	10	0	3	0
BPIFA1	9	0	6	0	3	0
	0	0	3	0	5	0
BPIFA2	8	0	6	0	3	0
	0	0	10	1	7	0
BPIFA3	13	0	6	0	0	0
	0	0	2	0	1	0
BPIFB1	15	0	9	0	1	0
	0	0	5	0	1	0
BPIFB2	12	0	5	0	3	0
	0	0	6	0	4	0
BPIFB3	7	0	14	0	1	0
	0	0	11	0	9	0
BPIFB4	14	0	6	0	2	0
	0	0	6	0	3	0
BPIFB6	9	0	7	0	2	0
	0	0	9	0	2	0
BPIFC	12	0	9	0	1	0
	0	0	13	0	0	0
BPNT1	34	87	23	58	49	50
	57	90	55	18	45	17
BPTF	418	2167	478	1420	896	1345
	1433	1510	721	673	893	351
BPY2	1	0	7	0	0	0
	0	0	2	0	2	0
BPY2B	1	0	1	0	0	0
	0	0	4	0	1	0
BPY2C	0	0	5	1	0	0
	0	0	3	0	4	0
BRAF	67	202	62	141	93	139
	176	225	124	84	139	51
BRAP	77	322	75	184	179	246
	248	329	168	110	175	59
BRAT1	17	25	10	22	19	27
	18	39	24	18	29	6
BRCA1	80	166	73	97	98	106
	95	132	86	57	90	31
BRCA2	76	11	51	17	23	6
	6	9	55	4	35	1
BRCC3	47	276	72	171	112	183
	198	225	74	64	125	50
BRD1	54	281	64	178	96	151
	238	279	142	83	156	68
BRD2	184	945	230	541	438	669
	677	960	361	342	588	209

BRD3	61	226	47	151	132	199
	162	207	103	60	140	47
BRD4	70	269	50	149	107	149
	201	190	72	81	107	66
BRD7	131	678	122	366	307	417
	374	628	197	213	317	115
BRD8	92	310	73	171	176	219
	292	380	166	78	176	62
BRD9	54	198	51	127	107	138
	173	220	137	74	121	46
BRDT	37	1	25	0	11	0
	0	0	27	0	16	1
BRE	61	247	56	161	86	196
	233	312	113	83	155	48
BRF1	62	159	51	130	57	127
	105	200	88	42	108	20
BRF2	28	100	33	110	67	91
	94	144	72	34	95	21
BRI3	15	121	32	84	46	96
	152	152	74	29	73	15
BRI3BP	9	6	3	4	4	8
	7	9	5	3	9	1
BRICD5	4	8	3	7	5	6
	6	12	3	2	1	1
BRIP1	59	9	30	5	12	2
	3	5	35	4	18	2
BRIX1	26	81	25	69	55	73
	100	148	51	30	68	23
BRK1	72	240	53	201	88	275
	319	442	158	80	187	60
BRMS1	17	60	17	40	31	44
	39	72	29	12	26	9
BRMS1L	38	152	35	149	65	98
	109	132	57	34	84	15
BROX	69	319	84	224	130	272
	288	322	136	87	185	44
BRPF1	41	111	21	56	61	67
	84	143	43	41	68	29
BRPF3	57	154	48	132	75	115
	143	128	56	46	79	23
BRS3	10	0	6	0	2	0
	0	0	9	0	3	0
BRSK1	19	1	12	1	4	0
	1	1	9	0	2	0
BRSK2	19	1	7	0	7	1
	0	4	1	0	11	1
BRWD1	655	3372	953	2182	1578	3011
	1972	3198	1533	876	1663	564
BRWD3	132	314	76	252	186	230
	191	314	146	82	182	53
BSCL2	20	97	7	60	34	43
	57	69	35	18	23	6
BSDC1	91	329	81	275	160	278
	276	439	175	110	203	70
BSG	167	955	89	605	337	629
	905	1082	381	214	524	115
BSN	42	12	26	8	13	5
	6	13	19	2	7	0

BSND	6	0	3	0	0	0
	0	0	1	0	0	0
BSPH1	6	0	5	0	4	0
	0	0	3	0	5	0
BSPRY	15	1	4	0	1	0
	0	0	5	0	0	0
BST1	18	9	7	7	4	7
	2	10	17	1	10	2
BST2	10	91	13	51	34	64
	99	87	63	37	47	9
BSX	3	0	0	0	0	0
	0	0	0	0	0	0
BTAF1	209	825	281	754	375	723
	644	1140	562	280	665	195
BTBD1	804	4030	781	4027	1641	3392
	3367	4591	1625	1001	2798	728
BTBD10	62	241	80	200	108	221
	219	266	148	83	175	44
BTBD11	26	23	22	13	12	12
	2	5	15	0	8	1
BTBD16	37	5	9	9	11	12
	4	1	16	6	13	4
BTBD17	2	0	1	0	0	0
	0	0	0	0	1	0
BTBD18	19	1	10	1	8	3
	1	2	15	0	5	1
BTBD19	3	6	2	10	4	2
	1	8	6	0	1	1
BTBD2	22	141	18	69	47	49
	112	77	38	44	34	26
BTBD3	85	370	101	298	196	286
	259	363	193	103	180	46
BTBD6	121	535	105	473	213	475
	578	391	291	151	265	106
BTBD7	143	519	131	365	274	347
	387	441	257	157	266	110
BTBD8	16	18	13	14	18	9
	43	38	32	19	35	5
BTBD9	61	227	58	100	102	164
	172	195	131	83	159	29
BTC	11	0	4	3	3	6
	2	3	5	1	11	0
BTD	14	64	16	26	26	43
	46	63	34	38	30	16
BTF3	336	1881	329	1161	796	1547
	2172	2916	1038	641	1294	302
BTF3L4	108	498	111	407	222	539
	408	717	312	176	328	87
BTG1	52	190	54	143	91	150
	236	315	111	71	131	66
BTG2	152	376	62	264	372	251
	773	1583	202	282	888	348
BTG3	11	22	11	10	7	11
	9	22	19	0	8	3
BTG4	5	0	8	0	4	0
	0	0	1	0	2	0
BTK	32	19	14	10	11	22
	8	14	10	4	12	7

BTLA	13	4	14	2	6	2
	1	2	7	0	4	0
BTN1A1	20	1	12	0	5	1
	1	0	8	0	2	0
BTN2A1	47	157	38	94	71	138
	132	147	84	52	83	26
BTN2A2	37	44	24	28	17	29
	23	36	40	15	40	5
BTN3A1	58	179	42	109	57	99
	160	163	105	64	136	20
BTN3A2	44	138	24	79	41	89
	89	100	102	47	90	26
BTN3A3	44	108	29	51	29	56
	62	84	53	28	66	10
BTNL10	4	0	1	0	0	0
	0	0	1	0	2	0
BTNL2	9	2	6	0	5	0
	0	0	5	0	2	0
BTNL3	19	1	8	0	0	1
	0	3	8	0	3	0
BTNL8	14	11	16	0	5	1
	2	1	12	2	6	0
BTNL9	68	292	38	139	123	126
	352	245	120	65	159	48
BTRC	140	497	114	333	265	344
	408	479	199	115	261	79
BUB1	28	5	19	1	4	0
	5	2	22	3	18	0
BUB1B	33	2	24	2	12	0
	3	2	34	0	18	1
BUB3	67	181	71	215	120	199
	166	302	146	77	166	40
BUD13	48	76	28	72	56	80
	80	140	48	38	69	20
BUD31	35	116	19	53	55	111
	90	140	48	37	39	14
BVES	326	1681	294	1039	901	1138
	1003	1739	597	488	755	296
BYSL	20	30	11	17	8	17
	15	34	16	5	6	2
BZRAP1	56	93	58	35	61	70
	68	136	70	27	90	21
BZW1	130	528	158	508	249	402
	397	654	253	171	344	112
BZW2	377	1678	296	1190	908	1042
	1896	2661	1169	462	1556	160
C10orf10	36	91	19	44	39	40
	129	222	115	50	96	52
C10orf105	13	3	10	2	5	3
	1	3	4	0	4	0
C10orf107	10	0	4	1	0	0
	1	0	4	1	0	1
C10orf11	9	20	5	8	6	12
	5	14	11	1	7	4
C10orf111	9	0	6	1	2	6
	0	1	0	3	1	0
C10orf114	5	2	2	0	3	0
	0	1	5	0	0	0

C10orf116	11	64	16	39	29	20
	52	66	29	17	44	30
C10orf118	138	585	162	434	269	414
	501	650	279	178	377	127
C10orf12	67	312	96	178	167	180
	144	181	64	84	93	52
C10orf120	8	0	1	0	1	0
	0	0	4	0	1	0
C10orf128	11	6	7	7	6	4
	8	11	6	3	8	0
C10orf129	16	0	12	0	8	0
	0	1	6	0	9	0
C10orf131	18	8	7	3	3	7
	3	8	7	1	2	0
C10orf137	77	294	106	227	154	213
	207	382	153	113	237	54
C10orf2	28	78	24	49	63	76
	43	62	28	27	42	11
C10orf25	24	12	8	5	8	3
	3	6	11	3	13	0
C10orf32	40	216	49	259	103	253
	294	310	140	85	191	49
C10orf35	6	4	5	0	1	3
	1	5	5	0	3	0
C10orf47	9	5	7	3	3	3
	1	5	10	0	1	1
C10orf53	13	0	2	0	0	0
	0	0	4	0	1	0
C10orf54	17	64	28	55	32	37
	44	68	32	23	41	19
C10orf55	7	2	4	3	1	5
	2	6	9	0	5	0
C10orf57	37	46	25	41	53	38
	54	65	37	17	50	8
C10orf62	1	0	0	0	1	0
	1	1	0	0	0	0
C10orf67	20	1	7	1	7	2
	0	5	11	2	3	0
C10orf68	50	71	35	72	28	38
	80	83	42	41	67	13
C10orf71	551	4233	468	2064	1506	1869
	2998	3176	1049	1107	1930	687
C10orf76	128	451	95	317	183	312
	313	357	219	77	209	53
C10orf82	5	0	3	0	3	0
	0	0	2	0	1	1
C10orf88	34	99	36	76	60	64
	71	102	56	25	67	20
C10orf90	31	1	11	0	0	3
	1	6	9	2	4	0
C10orf91	1	0	3	0	1	0
	0	0	0	0	1	0
C10orf95	2	0	1	0	0	1
	2	0	1	0	1	0
C10orf99	4	0	1	0	1	0
	0	0	2	0	0	0
C11orf1	16	52	11	50	11	37
	65	45	33	10	33	6

C11orf10	32	97	23	99	65	96
	108	227	89	34	100	15
C11orf16	11	1	6	1	2	1
	0	1	4	0	2	0
C11orf20	3	0	3	0	0	1
	1	0	2	0	2	0
C11orf21	11	4	11	3	2	1
	1	1	3	0	6	0
C11orf24	20	51	16	39	18	46
	41	43	31	27	39	11
C11orf30	120	331	115	221	210	265
	328	452	216	110	233	77
C11orf31	20	86	14	80	39	62
	89	157	68	29	48	16
C11orf34	18	0	5	0	2	1
	0	0	4	0	3	0
C11orf35	5	0	1	0	1	0
	2	0	1	0	5	0
C11orf40	2	1	2	2	0	0
	0	0	0	1	1	0
C11orf42	6	0	1	0	1	1
	0	0	0	0	0	0
C11orf45	25	2	20	7	8	5
	3	2	10	0	9	0
C11orf48	20	65	12	32	20	25
	50	51	27	13	16	5
C11orf49	18	17	12	15	15	19
	14	10	11	4	6	4
C11orf52	5	17	2	15	8	14
	12	23	11	7	10	3
C11orf53	8	0	2	3	0	11
	4	2	5	1	5	0
C11orf54	93	367	108	286	187	251
	318	412	171	129	263	104
C11orf57	39	136	40	125	90	124
	96	191	88	59	109	42
C11orf58	272	1625	244	1059	720	1099
	1376	1807	834	475	1020	299
C11orf63	31	10	12	4	11	5
	6	8	21	7	9	2
C11orf65	9	3	2	4	9	1
	8	9	10	5	6	2
C11orf68	8	27	6	23	11	25
	34	28	15	4	13	8
C11orf70	15	2	7	4	3	4
	1	2	6	0	9	0
C11orf71	44	109	31	124	65	105
	109	179	60	29	82	25
C11orf73	71	344	80	341	149	292
	402	563	213	110	237	63
C11orf74	36	117	21	77	49	91
	128	169	64	36	43	19
C11orf75	6	12	3	12	5	7
	8	21	4	4	3	2
C11orf80	16	9	8	14	5	6
	4	8	9	2	9	3
C11orf82	26	19	12	8	5	5
	13	15	16	3	17	4

C11orf83	18	68	8	52	35	34
	93	119	36	20	47	13
C11orf84	8	14	4	5	5	9
	5	6	5	3	3	4
C11orf85	9	0	12	0	2	0
	0	0	3	0	1	0
C11orf86	1	0	2	0	0	0
	0	0	0	0	2	0
C11orf87	24	0	15	0	6	0
	0	0	12	0	8	0
C11orf88	3	0	0	0	3	1
	0	0	3	0	1	0
C11orf9	16	0	16	0	2	0
	0	1	12	2	10	1
C11orf91	4	3	1	1	1	5
	1	3	2	0	0	0
C11orf93	13	8	5	6	6	9
	1	2	9	2	4	2
C11orf94	0	0	0	0	0	1
	0	0	0	0	0	0
C11orf95	43	97	33	83	59	103
	56	112	57	30	51	20
C11orf96	3	11	0	23	8	6
	14	20	2	3	4	7
C12orf10	22	60	5	38	24	56
	57	73	31	23	24	18
C12orf23	55	272	60	272	145	206
	196	220	108	68	123	22
C12orf28	24	1	13	3	1	3
	0	0	12	0	10	1
C12orf29	32	64	41	70	52	54
	112	117	63	25	55	16
C12orf39	15	33	11	9	29	7
	14	12	8	7	10	6
C12orf4	71	301	70	192	146	144
	184	310	130	91	193	47
C12orf40	20	0	11	0	13	1
	0	0	19	0	6	1
C12orf42	10	2	3	0	1	1
	0	0	5	0	4	0
C12orf43	18	43	14	31	33	29
	35	38	26	13	34	9
C12orf44	36	140	28	102	59	110
	94	134	51	24	65	16
C12orf45	15	37	11	37	11	23
	49	43	16	7	26	6
C12orf49	27	33	16	21	24	20
	25	44	32	2	32	5
C12orf5	71	179	30	109	59	107
	80	137	70	49	62	14
C12orf50	16	2	9	0	4	0
	0	0	15	0	7	0
C12orf52	31	79	15	62	34	55
	81	60	40	22	41	9
C12orf54	9	2	10	12	7	10
	5	4	13	2	7	1
C12orf55	22	3	16	2	13	0
	1	3	24	2	7	1

C12orf56	13	0	6	0	2	0
	1	0	10	0	2	0
C12orf57	52	139	25	178	61	135
	209	239	104	33	115	17
C12orf60	10	13	16	13	20	5
	19	33	17	12	18	2
C12orf61	2	1	1	2	2	1
	1	2	1	1	2	1
C12orf63	31	0	15	1	6	1
	0	1	11	1	8	0
C12orf65	30	70	25	39	33	47
	48	62	36	16	38	16
C12orf66	27	43	15	27	32	44
	41	37	29	10	28	9
C12orf68	3	0	3	3	0	0
	1	2	2	0	3	0
C12orf69	14	1	5	1	2	0
	1	1	7	0	4	0
C12orf70	17	4	10	2	8	0
	2	1	9	0	3	1
C12orf71	10	0	4	0	1	0
	0	0	4	0	0	0
C12orf73	14	85	8	32	24	46
	93	77	21	16	49	8
C12orf74	17	0	15	0	3	0
	0	0	4	0	1	0
C12orf75	104	107	34	45	76	23
	26	244	26	17	63	45
C12orf76	21	27	9	11	9	9
	12	36	21	5	11	6
C12orf77	12	1	6	0	1	0
	0	0	7	0	1	0
C13orf35	7	0	3	0	1	0
	0	0	8	0	0	0
C13orf45	7	2	8	1	2	1
	0	4	7	0	7	0
C14orf1	19	39	10	45	22	31
	32	53	30	12	34	10
C14orf101	64	133	65	100	71	84
	97	199	107	41	111	32
C14orf105	23	2	6	0	1	3
	1	1	9	1	2	0
C14orf119	36	103	27	79	46	91
	76	174	70	33	61	11
C14orf132	38	29	24	31	21	31
	11	42	40	16	19	13
C14orf142	14	85	7	58	24	42
	79	137	32	17	30	7
C14orf159	225	946	190	685	540	618
	972	1080	460	308	685	221
C14orf164	11	0	11	1	5	0
	0	0	9	0	7	1
C14orf166	159	899	150	612	347	779
	776	1039	430	287	481	148
C14orf166B	22	0	15	0	5	0
	1	2	9	0	6	0
C14orf169	9	44	11	45	23	34
	46	52	20	14	26	9

C14orf177	11	0	5	0	1	0
	0	0	4	0	2	0
C14orf178	4	1	0	1	2	3
	1	2	2	0	2	0
C14orf180	9	14	10	1	8	27
	12	29	3	2	17	12
C14orf182	11	10	8	5	3	4
	4	2	3	4	3	0
C14orf183	8	10	9	9	5	8
	5	2	4	2	4	2
C14orf2	251	1344	142	999	556	1074
	1856	2617	735	370	909	192
C14orf28	31	123	38	99	46	90
	100	128	55	32	76	12
C14orf37	22	22	20	12	15	16
	33	36	25	8	23	2
C14orf39	58	170	75	140	57	106
	142	257	73	54	146	28
C14orf79	8	2	8	3	4	4
	1	5	6	2	6	1
C14orf80	5	22	8	8	9	8
	7	9	8	0	8	0
C14orf93	22	40	18	24	15	13
	22	45	22	17	22	7
C15orf23	27	56	26	68	42	51
	41	68	38	14	76	19
C15orf26	7	0	4	3	3	0
	0	0	9	0	3	0
C15orf27	25	116	18	136	42	128
	63	91	42	15	51	4
C15orf32	11	0	7	0	5	0
	0	0	10	0	4	0
C15orf38	5	32	6	15	19	14
	15	17	12	11	15	3
C15orf38-AP3S2		99	438	82	321	208
	339	271	317	192	90	214
	96					
C15orf39	9	15	7	11	12	5
	4	8	8	4	5	4
C15orf40	24	53	23	41	16	36
	33	55	48	17	36	8
C15orf41	61	112	35	61	51	87
	163	258	85	84	96	38
C15orf43	6	0	5	0	2	0
	0	0	6	0	1	0
C15orf48	4	0	3	2	1	0
	1	3	1	2	1	1
C15orf52	116	643	149	392	227	400
	408	519	181	174	218	69
C15orf53	3	0	4	0	1	0
	0	0	2	0	2	0
C15orf54	18	0	8	0	3	0
	0	0	10	0	1	0
C15orf55	19	0	18	1	5	1
	0	2	12	0	9	0
C15orf56	3	2	6	0	1	0
	0	0	1	0	2	0

C15orf57	24	55	17	37	29	50
	53	70	39	18	43	10
C15orf59	4	8	8	4	3	1
	6	5	0	1	1	2
C15orf60	19	1	2	1	0	1
	2	0	5	1	1	0
C15orf61	24	119	15	86	55	99
	190	195	79	25	69	23
C15orf62	8	2	0	0	2	1
	2	4	3	3	3	1
C16orf11	1	0	2	0	1	0
	0	0	1	0	0	0
C16orf13	20	56	5	49	17	42
	47	104	34	14	29	17
C16orf3	3	0	1	0	0	0
	0	0	1	0	0	0
C16orf45	83	213	50	146	81	216
	177	162	107	54	139	51
C16orf46	7	15	7	4	7	4
	9	6	10	3	11	4
C16orf5	24	58	15	54	37	67
	69	58	29	23	57	12
C16orf52	52	174	41	169	85	156
	129	250	104	60	135	25
C16orf54	13	5	4	3	4	4
	0	3	5	0	3	0
C16orf55	9	5	4	1	2	8
	1	5	8	4	1	1
C16orf58	25	76	36	46	41	54
	47	116	46	28	70	27
C16orf59	3	1	3	0	1	0
	0	1	2	0	2	0
C16orf62	91	249	59	214	120	213
	267	284	153	107	195	45
C16orf70	35	104	23	96	65	101
	98	148	59	25	72	32
C16orf71	13	0	9	0	2	2
	2	0	10	1	3	0
C16orf72	161	841	165	822	351	655
	657	811	366	219	502	135
C16orf74	4	0	1	0	0	1
	1	1	0	0	1	1
C16orf78	13	0	4	0	2	0
	0	0	3	0	2	0
C16orf80	24	79	26	61	36	57
	70	127	37	23	67	11
C16orf82	10	0	4	0	0	0
	0	0	3	0	3	0
C16orf86	8	10	2	11	5	10
	14	12	3	5	9	5
C16orf87	50	196	32	134	100	112
	169	167	55	51	93	33
C16orf88	23	127	27	68	50	55
	91	91	35	36	37	16
C16orf89	12	1	6	0	2	0
	3	1	2	1	4	0
C16orf90	0	0	3	0	0	0
	0	0	0	0	0	0

C16orf91	6	23	6	15	10	17
	29	37	16	5	15	1
C16orf92	7	0	2	0	0	0
	0	0	4	0	1	0
C16orf93	18	0	6	2	4	1
	3	6	5	0	2	0
C16orf95	3	3	4	3	2	6
	3	5	4	4	5	0
C16orf96	20	0	3	3	3	2
	1	4	10	1	4	1
C17orf100	0	2	0	6	1	3
	2	1	3	1	1	2
C17orf102	19	0	11	0	3	0
	0	0	8	0	1	0
C17orf103	56	240	21	102	102	127
	181	126	54	41	74	26
C17orf104	33	4	17	4	7	3
	1	6	9	0	10	0
C17orf105	9	0	6	1	0	0
	0	0	0	0	2	0
C17orf107	7	2	6	0	2	0
	1	1	6	0	3	0
C17orf112	4	18	9	18	14	28
	15	1	13	1	2	1
C17orf47	18	0	6	1	2	1
	0	0	10	0	7	0
C17orf49	17	38	9	10	10	15
	32	33	15	9	16	3
C17orf50	7	0	2	0	2	0
	0	0	1	0	0	0
C17orf51	63	85	42	56	44	84
	94	118	49	52	57	31
C17orf53	16	1	6	2	0	0
	0	4	2	0	3	1
C17orf58	6	26	13	26	20	22
	24	46	12	4	14	4
C17orf59	12	17	12	25	16	25
	11	41	12	3	20	3
C17orf61	18	75	2	30	21	29
	44	91	35	20	23	11
C17orf62	12	20	12	21	12	19
	22	20	32	9	9	3
C17orf64	9	0	6	0	0	0
	0	0	1	0	1	0
C17orf66	17	0	14	2	3	0
	1	0	10	0	6	2
C17orf67	11	12	7	16	6	16
	12	17	15	4	9	3
C17orf70	16	30	12	22	15	26
	20	48	27	10	27	7
C17orf72	14	3	3	4	4	1
	2	6	7	1	1	0
C17orf74	3	0	1	0	2	0
	0	0	2	0	1	0
C17orf75	62	189	37	128	87	101
	152	195	74	76	110	29
C17orf77	23	0	7	3	5	0
	1	4	8	0	8	0

C17orf78	9	0	6	1	1	0
	0	1	6	0	2	0
C17orf79	30	158	18	132	75	103
	214	276	68	43	78	30
C17orf80	81	400	86	283	167	312
	282	377	175	122	258	78
C17orf82	2	1	2	0	0	0
	1	1	0	0	0	0
C17orf85	114	344	90	227	190	242
	274	377	156	123	221	77
C17orf89	46	345	38	241	90	193
	342	430	176	71	179	39
C17orf96	9	2	5	0	1	0
	1	2	4	0	0	0
C17orf97	8	6	13	9	11	12
	12	19	8	8	3	2
C17orf98	5	0	3	0	0	0
	0	0	1	0	1	0
C17orf99	4	0	1	2	1	0
	0	0	2	0	0	0
C18orf21	36	111	33	89	60	104
	163	205	93	61	112	31
C18orf25	167	826	184	796	440	732
	820	872	443	232	478	145
C18orf32	13	44	2	20	11	4
	21	48	30	10	23	11
C18orf42	14	1	13	0	3	1
	0	1	10	0	1	0
C18orf54	33	23	29	19	26	17
	20	35	33	5	19	6
C18orf56	3	0	2	0	0	0
	0	0	0	0	0	0
C18orf62	7	0	7	0	3	0
	0	0	1	0	1	0
C18orf63	23	0	19	0	5	0
	0	0	16	0	6	0
C18orf8	34	92	21	65	57	79
	68	113	61	24	58	32
C19orf10	15	57	10	34	17	25
	34	65	27	15	14	6
C19orf12	90	366	79	325	167	298
	295	386	185	102	208	73
C19orf18	8	26	10	16	10	13
	14	18	16	11	9	2
C19orf21	4	1	7	0	0	0
	0	0	3	0	3	0
C19orf24	5	4	1	3	1	4
	6	3	2	1	4	1
C19orf25	7	13	6	15	11	6
	23	18	5	10	6	3
C19orf26	6	0	2	0	1	0
	0	1	1	0	2	0
C19orf33	3	1	3	10	3	1
	4	3	2	1	6	2
C19orf35	2	0	1	0	0	0
	0	2	3	2	0	0
C19orf38	10	22	1	9	2	16
	21	9	12	7	11	1

C19orf40	6	0	3	1	2	1
	1	0	5	0	0	0
C19orf42	21	88	9	69	33	65
	68	118	29	30	64	20
C19orf43	33	202	28	169	100	193
	243	299	144	66	100	34
C19orf44	19	30	19	9	25	23
	27	46	8	16	22	6
C19orf45	4	0	1	0	0	0
	0	0	0	0	0	0
C19orf47	59	181	33	129	92	114
	177	196	90	77	131	40
C19orf48	10	13	6	6	3	3
	5	20	12	6	11	2
C19orf52	11	48	9	31	30	25
	36	50	39	13	28	13
C19orf53	41	241	24	117	101	160
	192	468	122	91	104	29
C19orf54	15	16	7	4	10	9
	16	16	14	2	5	2
C19orf55	22	6	10	5	4	5
	3	4	6	3	4	0
C19orf57	10	1	4	1	5	4
	3	2	5	1	4	0
C19orf59	4	0	2	0	1	0
	0	0	4	0	1	0
C19orf6	45	226	43	175	100	192
	204	362	156	66	159	47
C19orf60	35	199	22	146	68	117
	128	283	93	41	79	25
C19orf66	16	19	2	14	11	26
	11	24	18	6	18	4
C19orf67	3	2	7	0	1	0
	0	0	4	0	0	0
C19orf68	7	4	3	1	0	1
	0	1	1	2	2	0
C19orf69	3	1	2	0	0	0
	0	0	1	0	3	1
C19orf70	39	159	23	149	50	127
	222	370	107	47	106	25
C19orf71	0	1	1	0	0	0
	1	1	0	0	1	0
C19orf73	3	4	4	2	1	1
	5	2	0	2	1	0
C19orf76	8	2	7	2	0	0
	0	1	3	0	1	1
C19orf77	4	0	1	0	0	1
	0	0	0	0	1	0
C19orf80	2	0	1	0	1	0
	0	0	0	0	0	2
C19orf81	3	0	2	0	0	1
	0	0	3	1	1	0
C1D	36	190	35	150	101	88
	182	232	116	31	112	22
C1GALT1	84	241	85	256	137	234
	278	395	120	80	222	53
C1GALT1C1	30	131	31	107	60	116
	114	195	75	33	63	25

C1orf100	9	1	3	0	0	0	0	0	3	0	0	0
C1orf101	38	46	27	33	19	43	29	43	39	12	40	14
C1orf105	10	1	10	0	4	0	0	3	8	1	4	0
C1orf106	12	1	1	0	3	0	0	0	8	0	2	0
C1orf109	22	44	27	50	25	50	53	75	43	13	52	11
C1orf110	11	1	6	3	3	6	5	1	4	1	4	0
C1orf111	5	0	2	0	1	0	0	0	1	0	3	0
C1orf112	38	52	28	40	30	49	39	47	45	11	44	9
C1orf114	16	3	13	2	6	4	4	2	19	0	15	0
C1orf115	25	145	23	75	47	42	109	104	74	42	65	27
C1orf116	43	8	23	1	11	1	2	13	17	1	13	1
C1orf122	6	41	5	51	13	43	52	39	23	8	17	9
C1orf123	57	265	65	143	84	194	212	394	155	101	140	33
C1orf127	30	88	20	79	21	107	99	48	57	20	46	7
C1orf129	56	1	20	0	16	0	0	0	20	0	19	1
C1orf131	37	51	14	31	44	47	47	101	37	20	35	15
C1orf141	7	0	7	0	5	0	0	0	8	0	1	0
C1orf146	3	2	5	2	5	4	1	7	6	3	6	0
C1orf151-NBL1	17	54	14	17	20	39	55	57	34	7	30	7
C1orf158	16	10	17	11	17	9	4	0	2	9	1	1
C1orf159	3	1	5	4	3	2	3	5	4	2	7	1
C1orf162	26	35	8	26	17	37	24	44	24	13	22	7
C1orf167	8	0	1	0	0	0	0	0	5	0	0	0
C1orf168	43	2	15	18	11	7	62	88	33	9	29	1
C1orf172	2	0	0	1	0	0	0	0	3	0	1	0
C1orf173	67	3	27	3	20	5	0	2	39	0	17	1
C1orf174	29	84	18	51	46	64	69	85	44	26	52	14
C1orf177	10	0	11	0	1	0	0	0	3	0	2	0

C1orf185	8	3	0	0	0	0
	0	0	3	0	1	0
C1orf186	31	5	15	7	11	13
	6	8	22	3	11	1
C1orf189	4	0	3	1	2	0
	2	0	2	0	1	1
C1orf192	14	16	9	4	9	10
	6	14	10	1	8	1
C1orf194	5	0	1	0	1	0
	0	0	1	0	1	0
C1orf198	55	196	58	171	88	102
	198	151	86	48	72	51
C1orf204	14	19	19	13	12	8
	18	40	14	11	13	11
C1orf21	618	2359	416	2176	1095	1889
	2233	3730	1573	777	1741	446
C1orf210	4	0	4	0	0	0
	0	0	2	0	0	0
C1orf212	89	234	60	193	98	204
	235	293	139	64	146	42
C1orf216	37	108	25	117	44	90
	80	148	54	37	60	29
C1orf222	15	0	5	0	0	0
	0	1	2	0	2	0
C1orf226	17	5	9	5	3	2
	4	1	7	4	6	1
C1orf227	5	1	1	0	0	0
	0	1	1	0	0	0
C1orf228	8	0	7	1	0	3
	1	2	6	1	1	0
C1orf229	4	3	8	3	4	0
	3	2	5	3	2	1
C1orf233	3	3	1	0	0	0
	0	3	0	0	1	1
C1orf27	95	387	96	279	172	294
	317	476	200	142	247	79
C1orf35	12	29	16	17	9	27
	32	42	17	7	21	4
C1orf43	246	1502	167	1252	595	1188
	1801	1528	810	358	939	185
C1orf50	11	47	9	52	26	41
	39	79	30	12	26	12
C1orf51	39	221	41	96	109	137
	310	465	167	58	234	44
C1orf52	36	142	29	125	87	103
	110	140	71	58	84	9
C1orf53	1	1	2	3	0	6
	3	3	1	1	0	3
C1orf54	13	38	10	24	22	19
	28	45	14	4	25	2
C1orf56	23	16	9	9	11	10
	17	12	11	6	13	1
C1orf61	15	0	4	0	2	0
	0	0	6	0	3	0
C1orf63	48	319	63	219	130	221
	215	464	131	82	141	56
C1orf64	0	0	0	0	1	0
	0	0	2	1	1	0

C1orf65	6	0	2	0	1	0	0	0	2	0	2	0
C1orf68	3	0	1	0	0	0	0	0	1	0	0	0
C1orf74	13	17	12	19	14	19	18	24	9	7	16	3
C1orf85	13	11	4	17	3	15	13	32	12	6	20	6
C1orf86	8	24	12	17	6	15	10	11	11	3	5	1
C1orf87	21	0	13	1	4	0	2	1	14	0	6	0
C1orf94	17	0	5	0	1	0	0	0	4	0	5	0
C1orf95	39	57	27	25	20	40	34	46	38	17	36	6
C1QA	8	19	5	12	10	10	12	36	11	3	10	7
C1QB	1	6	5	13	14	25	9	57	20	8	13	8
C1QBP	150	956	152	718	353	557	1066	1378	516	211	510	102
C1QC	7	5	4	7	9	10	8	25	4	4	13	6
C1QL1	3	0	0	0	0	0	0	0	0	0	0	0
C1QL2	2	0	1	0	1	0	0	0	1	0	0	0
C1QL3	9	1	8	2	4	1	2	4	4	0	3	0
C1QL4	6	1	2	0	0	0	0	0	0	0	1	0
C1QTNF1	18	44	11	37	26	32	26	91	28	19	36	18
C1QTNF2	20	18	8	15	11	21	9	25	7	2	10	3
C1QTNF3	37	37	27	29	22	63	32	80	53	25	31	23
C1QTNF4	0	3	1	1	2	0	0	0	0	0	1	0
C1QTNF5	8	7	8	12	4	7	7	16	14	5	6	4
C1QTNF6	5	6	4	1	4	14	1	8	5	3	2	2
C1QTNF7	23	30	16	11	17	15	16	20	32	7	15	7
C1QTNF8	7	0	2	1	2	1	0	1	1	0	4	0
C1QTNF9	26	58	6	22	41	23	60	50	39	14	20	7
C1QTNF9B	5	4	4	2	13	7	14	13	6	5	7	1
C1QTNF9B-AS1	7	0	3	2	0	0	2	2	7	0	3	0
C1R (NC_000012 7187512..7189412)	24	77	23	122	62	72	44	118	48	25	60	17

C1R (NC_000012 7241204..7245043)				25	61	32
	116	48	71	52	105	49
	23	48	23			
C1RL	26	38	17	38	17	34
	25	76	33	16	24	8
C1S	180	532	205	848	375	577
	396	972	360	201	369	127
C2	32	12	23	25	21	20
	4	15	24	1	14	2
C20orf111	113	356	77	253	204	283
	352	541	194	147	287	72
C20orf112	25	41	26	24	18	20
	34	30	17	11	21	5
C20orf118	15	2	3	6	1	1
	1	2	3	0	1	0
C20orf132	50	75	40	58	46	65
	55	103	48	20	57	15
C20orf141	0	0	2	0	0	0
	1	0	0	0	0	0
C20orf144	1	0	0	0	0	0
	0	0	0	0	0	0
C20orf151	9	0	7	0	1	0
	0	0	3	0	2	0
C20orf152	14	4	10	5	2	0
	0	2	13	1	7	0
C20orf160	12	20	8	8	10	26
	19	26	14	13	13	7
C20orf166	176	689	108	577	264	406
	728	1147	466	294	385	135
C20orf173	8	0	1	0	1	0
	0	0	4	0	2	0
C20orf194	245	642	108	508	288	706
	1068	970	603	295	764	170
C20orf195	2	0	0	1	2	0
	0	0	2	0	0	0
C20orf196	21	6	3	6	8	6
	6	14	8	5	8	4
C20orf197	14	16	14	24	12	27
	13	31	21	6	15	2
C20orf20	16	47	10	31	27	32
	32	51	25	16	23	9
C20orf201	0	0	0	0	0	0
	0	0	0	0	1	0
C20orf202	7	5	1	3	1	2
	6	1	3	0	5	0
C20orf203	34	3	13	1	5	2
	1	3	9	1	7	1
C20orf24	22	286	24	323	77	219
	247	246	135	48	130	38
C20orf26	76	162	39	33	81	143
	114	480	90	57	96	33
C20orf27	13	25	11	10	6	11
	11	30	14	11	8	2
C20orf43	140	773	140	514	347	594
	652	797	396	242	450	120
C20orf72	40	171	30	107	76	114
	158	222	90	41	126	37

C20orf78	5	0	3	0	2	0
	0	0	1	0	2	0
C20orf79	0	0	0	1	0	0
	0	0	0	0	0	0
C20orf85	2	0	3	1	1	0
	0	0	3	0	1	0
C20orf94	10	5	9	16	11	5
	11	28	20	1	2	3
C20orf96	12	5	13	8	5	6
	7	6	9	2	5	2
C21orf2	19	46	10	18	9	18
	31	38	22	12	17	3
C21orf33	80	481	61	277	173	259
	575	746	261	107	341	90
C21orf56	7	12	3	6	1	8
	13	12	7	4	5	2
C21orf58	12	4	6	4	8	5
	4	8	5	3	4	0
C21orf59	47	233	38	143	118	144
	180	294	105	91	160	39
C21orf62	41	7	15	4	13	5
	6	3	28	2	12	1
C21orf7	39	211	81	186	74	110
	176	278	95	83	108	34
C21orf91	45	146	43	104	65	100
	146	138	90	44	87	26
C22orf13	194	759	140	623	392	742
	794	966	421	290	499	158
C22orf15	7	0	0	0	1	1
	0	4	3	0	4	0
C22orf23	13	2	1	0	3	1
	1	2	5	1	6	0
C22orf24	3	0	1	0	4	0
	0	1	6	0	2	0
C22orf25	26	83	14	47	26	78
	65	90	35	18	33	23
C22orf26	13	5	1	2	2	6
	7	4	7	1	4	3
C22orf28	81	332	80	229	143	302
	240	512	188	86	243	62
C22orf29	1	0	0	1	1	0
	0	0	1	0	0	0
C22orf31	11	1	6	0	1	0
	0	2	2	1	2	0
C22orf32	26	135	8	88	42	70
	108	100	48	24	40	9
C22orf39	99	446	55	228	181	307
	294	437	195	118	225	85
C22orf42	14	0	8	0	10	0
	0	0	2	0	2	0
C22orf43	22	9	16	3	5	6
	1	5	11	1	4	0
C22orf46	41	55	32	53	44	52
	32	38	33	29	42	7
C2CD2	51	106	31	61	60	63
	82	80	61	19	54	34
C2CD2L	15	31	5	32	19	29
	30	42	20	10	37	6

C2CD3	82	151	54	110	92	83
	125	154	94	55	127	26
C2CD4A	13	0	5	0	0	0
	0	0	2	0	2	0
C2CD4B	2	1	3	2	1	0
	3	0	1	1	1	0
C2CD4C	8	4	3	1	0	0
	2	2	3	0	2	0
C2CD4D	1	0	6	0	0	0
	0	0	1	0	4	0
C2orf15	13	0	3	4	1	2
	0	1	2	1	4	0
C2orf16	39	1	28	0	8	2
	0	2	16	0	5	0
C2orf18	36	90	23	79	30	91
	74	121	53	21	73	30
C2orf27A	6	22	11	20	8	12
	7	11	17	4	18	4
C2orf27B	11	1	8	1	3	3
	1	0	6	0	7	0
C2orf29	55	192	46	150	114	128
	166	180	73	55	90	35
C2orf40	4	10	6	6	8	3
	3	10	7	1	5	2
C2orf42	15	72	17	45	29	42
	43	55	26	21	44	10
C2orf43	17	12	14	12	8	5
	10	18	29	6	18	2
C2orf44	57	129	59	94	68	130
	115	179	73	46	107	19
C2orf47	40	138	23	91	62	94
	177	173	74	51	104	24
C2orf48	7	0	3	0	1	0
	0	0	4	0	5	0
C2orf49	26	126	23	90	93	105
	177	169	69	59	109	27
C2orf50	7	0	0	0	1	0
	0	0	1	0	1	0
C2orf53	10	0	6	0	1	0
	0	0	0	0	3	0
C2orf54	17	0	7	0	4	0
	0	0	3	0	1	0
C2orf57	1	0	0	0	0	0
	0	0	0	0	0	0
C2orf61	37	6	13	5	6	8
	4	3	13	1	1	0
C2orf62	12	0	5	0	0	0
	0	0	6	0	0	0
C2orf65	16	3	17	0	7	1
	0	0	6	0	6	1
C2orf66	9	0	4	0	2	1
	0	2	4	0	3	0
C2orf68	96	429	77	321	161	234
	350	354	164	122	213	87
C2orf69	60	210	46	226	135	226
	172	280	153	86	156	54
C2orf70	4	0	4	1	4	1
	0	0	1	0	0	0

C2orf71	33	0	11	0	6	3
	0	1	6	1	9	0
C2orf72	8	1	5	0	5	0
	0	0	5	0	2	1
C2orf73	12	1	14	0	0	1
	0	1	7	1	3	0
C2orf74	17	41	8	36	12	41
	46	94	32	8	17	14
C2orf76	14	18	8	10	15	10
	18	21	19	8	19	3
C2orf78	16	0	18	0	6	0
	0	0	18	0	4	0
C2orf80	9	0	13	0	4	0
	0	0	2	0	6	0
C2orf81	4	2	0	1	4	1
	1	0	4	0	2	0
C2orf82	1	1	0	0	0	2
	0	3	0	0	0	0
C2orf83	17	0	9	0	0	0
	0	0	4	0	2	0
C2orf88	54	207	71	162	92	115
	256	194	127	69	140	46
C2orf91	38	0	18	0	7	3
	0	1	19	0	11	1
C3	131	682	316	992	478	550
	621	1114	402	257	439	186
C3AR1	9	12	7	5	9	9
	7	18	8	1	6	6
C3orf14	5	0	4	6	3	3
	4	3	5	0	6	2
C3orf17	101	451	90	358	183	376
	306	550	243	134	291	90
C3orf18	52	158	45	147	50	182
	160	180	72	53	94	21
C3orf20	27	2	21	2	11	4
	4	6	9	1	8	0
C3orf22	4	0	2	0	2	0
	0	0	3	0	2	0
C3orf24	6	0	0	0	0	0
	0	0	2	0	1	0
C3orf27	10	0	3	0	2	0
	0	0	1	0	2	0
C3orf30	5	0	5	0	2	0
	0	0	8	0	3	0
C3orf32	14	3	13	2	4	0
	0	2	7	3	8	0
C3orf33	16	79	11	48	24	73
	40	59	46	18	29	9
C3orf35	24	30	25	26	6	31
	22	29	20	14	21	3
C3orf36	10	0	5	0	0	0
	0	0	3	0	0	0
C3orf37	33	134	32	82	66	62
	142	117	49	28	72	12
C3orf38	44	157	26	140	84	130
	132	234	81	52	123	37
C3orf43	174	1096	150	565	493	591
	954	1547	397	304	981	171

C3orf45	32	65	16	63	18	60
	43	62	32	10	33	5
C3orf52	19	2	16	2	3	2
	3	3	8	2	5	2
C3orf55	13	19	19	19	14	23
	6	20	10	5	16	7
C3orf58	62	178	56	163	86	117
	100	143	86	46	50	25
C3orf62	27	32	18	22	15	31
	14	25	23	12	25	3
C3orf67	29	1	19	2	8	0
	0	2	11	0	6	0
C3orf70	31	14	11	17	16	8
	25	25	25	8	12	5
C3orf72	21	0	1	1	6	1
	1	0	9	0	1	0
C3orf79	1	0	2	0	3	0
	0	0	0	0	1	0
C3orf80	5	1	8	4	1	0
	0	0	3	0	2	0
C4A	62	19	40	32	10	13
	11	22	10	13	23	15
C4B	29	6	34	9	4	1
	11	13	18	7	22	3
C4BPA	19	0	9	0	3	0
	0	1	10	0	6	1
C4BPB	10	1	5	0	3	0
	0	3	12	0	5	0
C4orf17	11	0	10	0	5	0
	0	0	8	0	3	0
C4orf19	26	1	12	4	3	13
	8	6	3	0	7	0
C4orf21	91	125	80	164	101	83
	141	127	105	63	104	20
C4orf22	10	0	9	0	1	0
	0	0	13	0	6	0
C4orf26	24	3	8	6	4	4
	0	2	14	2	8	1
C4orf27	24	94	23	81	32	64
	62	96	40	29	59	6
C4orf29	69	153	55	115	83	96
	132	216	84	44	124	29
C4orf3	145	711	178	617	321	615
	750	887	380	208	398	136
C4orf32	23	70	17	99	36	66
	74	94	49	12	36	16
C4orf33	21	55	9	26	20	36
	30	57	24	12	25	5
C4orf34	43	106	19	86	70	64
	89	134	61	22	54	23
C4orf36	7	0	2	3	0	0
	5	3	7	0	2	0
C4orf40	15	0	16	0	6	0
	0	0	6	0	5	0
C4orf45	4	0	5	0	4	0
	0	1	3	0	2	0
C4orf46	29	51	32	47	27	46
	69	112	46	18	65	12

C4orf47	5	2	4	3	2	4
	3	3	6	2	5	0
C4orf48	1	0	1	2	0	0
	0	1	0	0	0	0
C4orf50	14	0	7	0	1	0
	0	0	2	0	1	0
C4orf51	6	0	1	0	1	0
	0	0	2	0	3	0
C4orf52	86	491	65	358	149	374
	504	497	255	93	329	46
C4orf6	5	0	4	0	1	0
	0	0	5	0	1	0
C5	58	31	44	26	28	11
	16	30	34	12	27	9
C5AR1	5	4	3	1	4	6
	8	6	4	2	2	0
C5orf15	55	295	64	256	165	237
	318	383	155	100	189	56
C5orf20	12	1	11	1	3	0
	0	1	7	0	3	0
C5orf22	90	521	76	306	168	332
	376	481	230	126	246	65
C5orf24	87	383	100	387	187	298
	419	471	189	89	262	87
C5orf25	27	46	19	33	24	32
	55	36	17	4	22	8
C5orf28	29	52	20	61	28	44
	54	62	23	15	52	7
C5orf30	25	78	33	85	63	70
	77	88	43	28	36	9
C5orf34	19	16	16	5	12	4
	14	4	16	4	20	7
C5orf38	3	0	4	0	0	0
	0	0	0	0	0	0
C5orf4	60	155	27	69	65	71
	141	281	109	38	101	38
C5orf42	247	964	316	783	423	696
	766	984	481	333	632	179
C5orf43	57	224	45	143	104	124
	169	229	99	70	101	26
C5orf44	71	240	65	212	117	195
	209	296	136	59	190	53
C5orf45	14	31	17	25	10	24
	35	51	23	11	11	9
C5orf46	4	0	1	0	1	0
	0	0	0	0	1	0
C5orf47	10	2	6	1	2	4
	0	4	3	1	2	0
C5orf48	1	1	1	0	2	5
	0	0	2	0	3	1
C5orf49	11	1	6	0	2	1
	0	0	3	0	1	0
C5orf50	3	0	3	0	3	0
	0	0	5	0	1	0
C5orf51	50	350	60	171	120	117
	194	416	133	59	191	41
C5orf52	2	0	1	0	3	1
	0	0	1	0	0	0

C5orf54	13	54	13	42	38	40
	43	69	31	15	35	21
C5orf55	1	23	5	13	4	9
	4	30	10	4	4	5
C5orf58	3	0	3	1	0	0
	0	1	0	0	0	0
C5orf60	6	0	3	0	0	0
	0	0	2	0	3	0
C5orf63	66	135	58	72	52	118
	81	162	85	33	62	19
C5orf64	12	0	7	0	7	0
	0	1	10	0	7	0
C6	34	2	27	9	10	1
	6	25	34	6	20	5
C6orf1	10	12	1	4	1	13
	19	14	11	4	6	3
C6orf10	29	0	17	0	6	1
	0	0	15	1	8	0
C6orf106	282	1759	154	1138	779	1392
	1320	1552	673	488	806	273
C6orf108	14	110	8	54	41	45
	148	124	43	13	42	10
C6orf118	12	0	5	0	0	0
	0	0	4	0	3	0
C6orf120	73	320	62	349	188	251
	279	359	167	87	195	61
C6orf130	30	127	15	60	56	62
	101	188	67	42	61	24
C6orf132	28	1	19	8	11	4
	1	7	9	2	14	2
C6orf136	13	48	12	47	30	28
	82	84	18	12	27	6
C6orf141	7	12	13	4	4	3
	9	10	6	2	6	4
C6orf15	6	0	1	0	1	0
	0	0	1	0	1	0
C6orf162	47	215	45	142	70	128
	178	278	121	57	148	43
C6orf163	16	95	25	46	21	63
	70	86	52	25	46	22
C6orf165	17	3	9	3	6	4
	1	2	11	0	2	0
C6orf170	45	71	40	53	32	38
	27	29	62	8	38	9
C6orf183	37	14	18	15	6	33
	27	108	36	9	25	3
C6orf195	12	8	7	5	7	13
	7	10	22	5	4	2
C6orf201	15	2	4	1	5	0
	0	4	8	1	3	2
C6orf203	53	216	29	114	104	120
	183	210	75	76	126	30
C6orf211	93	480	75	286	238	402
	367	617	245	148	300	84
C6orf222	21	0	9	0	7	0
	0	0	14	0	8	0
C6orf223	10	0	4	0	2	0
	0	0	4	0	3	0

C6orf225	26	52	21	34	16	29
	34	60	40	18	27	8
C6orf226	0	0	0	1	1	3
	3	1	1	0	1	0
C6orf228	72	275	59	266	128	205
	244	241	113	81	140	46
C6orf25	22	2	7	0	1	0
	0	0	2	0	5	0
C6orf47	28	88	27	53	48	75
	53	113	52	16	46	13
C6orf48	21	69	16	82	39	104
	61	139	45	25	54	11
C6orf52	4	2	0	1	1	2
	0	4	1	1	0	0
C6orf57	13	52	14	34	33	46
	41	127	28	23	34	7
C6orf58	11	0	6	0	3	0
	0	0	9	0	6	0
C6orf62	157	849	174	765	394	668
	683	1013	340	220	429	152
C6orf7	9	0	2	0	1	0
	0	0	3	0	2	0
C6orf70	63	198	76	221	139	178
	139	262	110	76	135	27
C6orf89	143	561	108	507	251	435
	454	712	325	174	387	95
C6orf99	3	0	2	0	0	0
	0	0	0	0	4	0
C7	39	28	26	82	13	16
	7	82	29	3	18	35
C7orf10	31	111	39	59	56	64
	120	133	72	44	69	17
C7orf23	5	14	2	18	3	12
	21	31	16	4	17	5
C7orf25	30	141	34	99	50	114
	93	127	67	31	68	14
C7orf26	19	39	14	24	22	31
	27	35	18	10	15	8
C7orf29	0	1	0	3	1	0
	0	2	0	0	0	0
C7orf31	25	11	16	7	13	9
	11	7	22	1	7	1
C7orf33	7	0	3	0	0	0
	0	0	5	0	2	0
C7orf34	2	0	1	1	0	0
	0	0	2	0	0	0
C7orf41	228	730	262	472	382	417
	584	779	451	253	519	201
C7orf43	13	32	9	24	15	18
	33	37	11	6	11	4
C7orf45	2	0	6	0	1	0
	0	0	5	0	2	0
C7orf49	43	132	26	111	65	154
	125	127	63	36	63	18
C7orf50	42	122	21	59	28	55
	85	164	60	22	67	17
C7orf53	184	1025	151	643	507	808
	1076	1071	527	252	658	104

C7orf55	6	50	8	72	24	32	68	111	33	7	43	7
C7orf55-LUC7L2	28	99	59	106	144	150	119	186	50	75	48	28
C7orf57	8	0	7	0	2	0	0	0	5	0	4	0
C7orf60	36	122	24	103	62	68	91	124	81	35	62	20
C7orf61	2	0	2	0	3	0	0	0	1	0	3	0
C7orf62	10	0	5	0	0	0	0	0	3	0	0	0
C7orf63	49	53	28	32	41	26	51	97	42	37	59	14
C7orf65	10	0	7	0	1	0	0	0	2	0	2	0
C7orf66	3	0	0	0	0	0	0	0	1	0	0	0
C7orf69	7	1	4	0	1	3	0	1	1	1	1	1
C7orf71	4	1	12	0	2	0	0	0	5	0	2	0
C7orf72	21	0	7	0	7	0	0	0	3	0	4	0
C7orf73	67	420	81	335	163	334	417	379	135	110	173	55
C8A	14	0	10	0	1	0	0	0	13	0	0	0
C8B	20	0	14	0	4	0	0	0	11	0	4	0
C8G	2	2	6	2	1	0	3	4	0	3	0	1
C8orf22	706	1023	443	1806	1138	1394	1905	4597	3014	658	2454	215
C8orf31	21	5	1	1	5	3	0	3	5	2	6	0
C8orf33	46	109	27	116	53	96	90	151	55	40	68	19
C8orf34	25	16	14	15	28	6	1	6	17	1	11	2
C8orf37	21	30	14	55	13	22	18	26	18	4	18	4
C8orf4	21	209	22	88	66	62	125	142	66	49	74	15
C8orf40	60	312	39	288	128	273	324	469	178	84	189	50
C8orf42	16	25	21	28	16	15	27	37	31	14	17	2
C8orf44	0	3	0	4	3	5	8	6	1	3	0	8
C8orf44-SGK3	63	104	49	82	64	81	85	160	85	41	60	23
C8orf46	36	16	17	5	15	21	15	28	17	3	23	10
C8orf47	5	0	4	0	3	0	0	2	1	2	1	0

C8orf48	11	5	9	3	4	1
	2	7	8	0	10	0
C8orf58	28	32	17	38	17	34
	34	37	24	14	20	3
C8orf59	30	190	30	171	80	166
	204	344	112	57	112	26
C8orf73	7	3	7	2	1	2
	1	5	4	0	1	0
C8orf74	2	0	1	0	0	0
	0	0	1	0	3	0
C8orf76	13	82	14	49	37	36
	59	53	35	27	37	7
C8orf82	2	7	4	0	7	7
	5	16	6	4	7	2
C8orf86	15	0	6	0	1	0
	0	1	7	0	1	1
C8orf87	3	0	4	0	1	0
	0	0	1	0	2	0
C9	18	0	21	1	4	0
	0	1	11	0	7	2
C9orf106	1	1	2	3	0	1
	1	3	3	2	1	1
C9orf114	44	93	24	34	42	54
	55	98	49	24	39	9
C9orf116	8	0	1	0	1	2
	1	0	2	0	0	0
C9orf117	4	0	8	1	0	5
	0	2	8	0	8	0
C9orf123	87	413	82	355	182	297
	486	668	228	119	289	63
C9orf129	9	5	4	10	2	1
	2	0	5	0	2	0
C9orf131	21	13	9	8	7	10
	4	14	11	3	10	2
C9orf135	9	0	7	3	10	6
	9	10	3	0	7	0
C9orf139	6	0	3	0	1	1
	2	1	5	0	1	0
C9orf142	5	17	8	10	7	5
	8	22	4	4	8	1
C9orf152	7	0	3	0	1	1
	1	0	5	0	1	0
C9orf153	5	3	5	6	1	0
	4	4	4	2	3	4
C9orf156	36	127	29	68	64	93
	87	194	51	41	103	19
C9orf16	2	6	2	3	0	3
	14	2	3	1	5	0
C9orf163	1	0	3	1	1	0
	0	1	1	0	1	0
C9orf169	1	2	0	0	0	0
	1	0	0	0	0	0
C9orf170	24	14	26	23	11	32
	7	11	15	4	15	1
C9orf171	5	0	4	0	0	0
	0	0	3	0	1	0
C9orf172	4	2	2	1	1	0
	0	1	0	0	1	2

C9orf173	3	1	3	0	0	0
	0	0	1	0	1	0
C9orf174	59	94	52	72	47	83
	62	80	67	19	76	29
C9orf24	4	2	5	4	2	1
	1	2	7	0	5	0
C9orf3	211	760	131	434	275	616
	825	1093	510	263	444	118
C9orf37	7	26	8	13	15	16
	15	25	11	9	24	6
C9orf40	20	43	13	30	23	31
	30	55	21	14	28	5
C9orf41	37	174	57	131	91	146
	108	160	86	48	120	24
C9orf43	27	2	7	5	4	6
	2	4	8	2	13	0
C9orf47	2	0	2	0	0	0
	0	0	1	0	1	0
C9orf50	5	0	2	0	0	0
	0	0	1	0	1	0
C9orf57	9	0	6	1	2	0
	0	0	2	0	0	0
C9orf62	5	0	4	0	3	0
	0	0	1	0	2	0
C9orf64	32	86	16	69	52	59
	92	82	67	17	58	21
C9orf66	8	2	6	0	0	1
	1	1	0	0	1	0
C9orf69	24	129	16	82	50	83
	84	150	72	39	60	17
C9orf72	58	167	86	213	112	148
	200	391	176	86	269	54
C9orf78	63	356	52	211	164	336
	308	411	136	109	185	61
C9orf84	34	4	35	2	13	1
	0	3	29	0	13	1
C9orf85	18	85	18	41	28	51
	58	56	37	22	33	10
C9orf89	5	17	1	19	11	11
	24	30	12	6	18	6
C9orf9	5	5	3	1	0	2
	1	3	4	0	0	1
C9orf91	39	81	17	80	55	53
	100	77	40	19	38	15
C9orf96	17	5	10	0	4	7
	2	1	13	0	7	1
CA1	19	10	11	8	3	7
	1	0	6	2	6	1
CA10	28	1	22	0	6	0
	0	0	9	0	4	0
CA11	11	8	12	4	3	3
	10	3	8	6	3	0
CA12	14	3	15	5	9	4
	1	3	17	0	3	2
CA13	37	7	9	8	14	6
	5	8	15	4	11	0
CA14	21	98	32	30	60	14
	135	85	22	40	42	5

CA15P3	7	1	2	0	3	1
	0	6	1	1	0	0
CA2	43	316	58	340	102	124
	284	305	112	62	116	26
CA3	10147	65801	15914	28024	15774	27487
	38134	84588	24184	8272	20719	8403
CA4	9	38	5	20	18	17
	49	41	17	13	18	5
CA5A	5	0	0	0	1	0
	0	1	3	0	0	0
CA5B	50	60	24	40	31	36
	30	56	35	20	34	16
CA6	6	0	7	0	1	0
	0	0	2	0	6	0
CA7	8	0	5	0	2	0
	0	0	3	0	1	0
CA8	18	33	12	11	17	11
	34	32	28	14	38	4
CA9	9	0	4	0	1	1
	0	0	3	0	2	0
CAAP1	40	212	39	144	73	164
	139	248	106	43	124	25
CAB39	680	4553	857	3964	2205	2736
	3236	3349	1469	1110	2166	611
CAB39L	60	130	77	235	97	97
	71	98	55	24	66	32
CABIN1	81	268	75	144	99	150
	129	226	117	76	155	53
CABLES1	46	21	12	11	15	15
	18	16	16	6	18	4
CABLES2	21	30	14	25	15	12
	14	37	16	11	27	7
CABP1	10	11	2	2	5	2
	2	12	6	3	8	1
CABP2	1	0	4	0	0	0
	0	0	1	0	0	0
CABP4	25	12	10	3	4	7
	4	10	19	0	11	7
CABP5	20	0	11	1	2	3
	0	0	7	0	4	0
CABP7	16	6	9	1	2	2
	0	2	2	0	1	0
CABS1	12	0	12	0	3	0
	0	0	3	0	1	0
CABYR	21	6	3	1	2	1
	1	7	7	0	12	0
CACFD1	6	15	5	6	5	11
	10	19	4	2	9	1
CACHD1	67	118	44	75	81	100
	60	86	83	39	60	20
CACNA1A	58	2	26	3	8	0
	1	0	26	1	19	1
CACNA1B	44	1	27	1	15	0
	0	0	27	0	17	0
CACNA1C	98	94	64	63	49	44
	67	64	65	21	54	12
CACNA1D	69	6	46	14	13	5
	4	6	41	0	11	2

CACNA1E	99	1	57	2	29	1
	1	2	47	0	34	4
CACNA1F	48	0	15	0	9	2
	0	0	14	0	9	0
CACNA1G	42	5	30	2	6	3
	0	2	8	0	16	0
CACNA1H	22	32	18	19	15	21
	11	25	20	8	11	3
CACNA1I	43	0	25	0	3	1
	0	0	15	1	7	0
CACNA1S	1148	7013	1282	5401	2510	4991
	3952	5700	3049	1563	3217	880
CACNA2D1	614	4606	678	3276	2018	2951
	3148	2810	1643	1030	2018	612
CACNA2D2	36	39	21	28	15	21
	6	19	17	7	14	1
CACNA2D3	110	378	76	293	163	326
	306	259	145	107	220	65
CACNA2D4	52	11	27	6	14	7
	1	3	20	6	16	1
CACNB1	327	1737	365	1282	700	1607
	1607	1244	726	490	827	259
CACNB2	52	25	30	36	23	141
	14	101	57	12	51	2
CACNB3	14	9	16	4	10	5
	9	12	7	4	4	3
CACNB4	39	13	36	14	22	18
	12	13	32	1	9	1
CACNG1	57	395	51	236	168	256
	430	437	244	174	266	105
CACNG2	19	0	9	0	1	1
	0	0	12	0	7	0
CACNG3	17	0	9	0	3	0
	0	0	4	0	4	0
CACNG4	5	0	3	0	2	0
	0	0	5	0	2	0
CACNG5	4	0	4	0	2	0
	0	0	3	0	0	0
CACNG6	64	188	44	172	96	158
	180	206	95	58	92	26
CACNG7	6	0	4	0	2	0
	0	0	2	0	2	0
CACNG8	45	28	27	9	13	5
	9	9	32	6	14	3
CACTIN	21	85	21	40	40	51
	49	94	39	28	43	21
CACUL1	194	772	221	693	419	581
	740	829	438	207	449	197
CACYBP	133	904	140	524	488	563
	713	770	320	211	350	103
CAD	82	113	52	95	59	89
	101	134	81	35	73	16
CADM1	60	138	65	120	83	103
	209	204	109	26	98	25
CADM2	66	147	32	147	179	102
	115	140	97	56	94	30
CADM3	39	8	12	14	15	7
	9	6	14	9	14	3

CADM4	8	30	6	11	12	8
	12	17	13	3	10	3
CADPS	46	5	43	23	15	5
	1	1	33	0	21	0
CADPS2	71	134	66	123	67	75
	93	185	92	52	72	30
CAGE1	22	0	20	2	5	3
	0	1	15	0	9	1
CALB1	22	2	16	1	8	5
	0	1	15	0	4	0
CALB2	15	1	8	2	7	2
	0	3	4	0	12	2
CALCA	20	0	4	1	1	2
	0	0	1	0	3	0
CALCB	9	0	3	1	0	0
	0	0	1	0	3	0
CALCOCO1	256	962	224	747	576	784
	937	1514	726	384	857	228
CALCOCO2	253	1255	250	962	629	1035
	1198	1263	516	501	699	252
CALCR	48	59	22	15	26	35
	31	39	44	18	35	6
CALCRL	66	292	71	211	145	151
	214	286	117	58	151	59
CALD1	155	885	169	585	325	424
	603	901	339	232	327	225
CALHM1	9	0	5	0	5	0
	0	0	3	0	4	0
CALHM2	7	26	11	16	10	14
	13	41	8	4	11	4
CALHM3	4	0	1	0	0	0
	0	0	3	0	0	0
CALM1	1433	8153	1873	7509	4318	6992
	6004	4824	3099	2429	2684	1168
CALM2	267	2058	291	1515	944	1488
	1402	1752	705	557	720	268
CALM3	266	1290	199	925	477	976
	1122	1305	566	389	662	196
CALML3	1	1	4	0	0	1
	0	0	1	0	2	0
CALML4	29	42	37	42	40	93
	51	51	20	17	30	9
CALML5	1	0	0	0	0	0
	0	0	0	0	0	0
CALML6	16	48	122	336	328	190
	216	144	84	83	103	25
CALN1	61	3	19	4	13	1
	1	2	33	2	14	0
CALR	128	802	129	495	342	575
	554	795	292	203	388	121
CALR3	3	0	6	0	6	0
	1	1	6	0	1	0
CALU	163	746	199	794	342	642
	719	907	443	235	485	179
CALY	5	0	2	0	0	0
	0	0	2	0	0	0
CAMK1	24	27	6	23	15	18
	10	21	16	8	16	7

CAMK1D	18	16	12	19	9	7
	9	12	14	2	11	7
CAMK1G	13	1	16	3	7	0
	0	2	13	0	5	0
CAMK2A	296	1013	180	969	525	795
	681	1125	490	418	579	208
CAMK2B	178	694	138	478	283	539
	450	828	309	269	418	135
CAMK2D	345	2011	414	1515	675	1011
	1277	2208	765	435	812	255
CAMK2G	232	1006	196	882	640	767
	841	1006	433	354	566	210
CAMK2N1	11	37	2	25	7	23
	22	50	15	9	15	5
CAMK2N2	4	0	5	0	0	0
	0	0	0	0	0	0
CAMK4	20	7	9	3	6	0
	0	2	16	1	9	1
CAMKK1	29	29	17	17	15	21
	18	20	18	6	10	9
CAMKK2	47	94	29	81	40	63
	67	105	97	42	45	16
CAMKMT	15	30	10	22	23	28
	17	41	32	10	25	7
CAMKV	19	0	7	0	7	0
	0	0	6	0	4	0
CAMLG	71	227	58	230	80	222
	240	318	164	70	144	51
CAMP	0	0	0	0	0	1
	0	0	3	0	0	0
CAMSAP1	173	844	240	612	399	681
	634	762	454	267	443	181
CAMSAP2	359	1528	606	1382	787	1623
	917	1002	533	481	746	271
CAMSAP3	9	1	1	0	2	3
	2	0	6	0	1	0
CAMTA1	113	314	93	192	183	187
	241	319	110	78	200	59
CAMTA2	64	213	47	125	96	135
	161	118	62	56	95	29
CAND1	173	654	220	668	373	535
	546	765	394	289	437	139
CAND2	294	1487	221	936	668	1178
	1094	1276	652	394	666	242
CANT1	45	93	16	73	38	78
	70	122	43	23	47	11
CANX	351	1949	333	1411	929	1542
	1308	1794	756	575	990	391
CAP1	81	319	75	218	188	247
	225	412	198	116	216	64
CAP2	796	4408	702	3693	2141	3592
	3434	4410	1939	1756	2297	711
CAPG	12	14	10	11	7	12
	8	8	9	2	8	1
CAPN1	72	223	53	166	94	184
	214	211	136	77	127	46
CAPN10	12	22	11	13	7	21
	19	29	20	6	16	7

CAPN11	18	2	11	0	1	0
	0	0	8	0	2	0
CAPN12	13	4	15	3	9	5
	0	4	6	0	5	0
CAPN13	21	1	16	0	2	1
	0	0	13	0	7	1
CAPN14	37	1	18	0	5	1
	0	0	14	1	9	1
CAPN2	152	775	148	639	356	546
	528	771	388	239	382	121
CAPN3	1067	6628	986	4888	2483	4809
	5086	5857	3084	1585	3830	800
CAPN5	23	13	15	12	6	9
	9	4	7	1	8	2
CAPN6	37	93	24	113	31	28
	75	58	43	15	33	3
CAPN7	134	614	180	592	279	521
	611	974	481	249	598	189
CAPN8	19	1	12	0	6	0
	0	2	12	0	6	1
CAPN9	28	3	17	0	4	0
	0	0	14	0	4	1
CAPNS1	96	504	111	354	219	461
	450	498	326	180	347	123
CAPNS2	0	0	1	0	0	0
	0	0	0	0	0	0
CAPRIN1	268	1491	294	1009	746	973
	1017	1343	557	403	643	258
CAPRIN2	70	297	126	189	107	187
	148	183	100	65	120	48
CAPS	9	15	4	8	4	6
	6	15	5	1	8	2
CAPS2	35	50	22	51	24	48
	45	94	46	22	63	8
CAPSL	5	0	9	1	3	0
	0	0	7	2	3	0
CAPZA1	100	482	106	396	252	362
	410	532	209	145	261	70
CAPZA2	594	3686	726	3043	1584	2705
	3295	3861	1817	1028	2316	546
CAPZA3	4	0	2	0	0	0
	0	0	0	0	0	0
CAPZB	79	652	104	422	257	368
	529	451	159	127	288	93
CARD10	20	46	14	24	23	25
	44	51	27	16	29	8
CARD11	30	7	20	1	4	3
	1	1	9	3	8	2
CARD14	17	0	10	1	4	2
	1	4	4	2	3	0
CARD16	9	29	5	18	13	15
	23	26	15	9	13	1
CARD17	6	1	4	0	2	0
	0	0	2	0	1	0
CARD18	2	0	3	0	1	0
	0	0	1	0	0	0
CARD6	40	76	20	49	42	65
	80	84	46	31	41	17

CARD8	97	441	95	269	235	274
	344	419	228	123	283	90
CARD9	7	3	5	3	5	2
	8	5	1	1	2	0
CARHSP1	17	49	11	30	13	23
	21	46	22	8	30	7
CARKD	66	246	62	132	122	175
	237	343	118	87	136	55
CARM1	67	321	45	231	167	266
	279	263	130	119	173	77
CARNS1	58	699	165	418	129	345
	438	172	216	130	155	59
CARS	60	106	29	66	73	107
	119	121	68	34	65	36
CARS2	63	276	47	185	137	200
	279	371	146	93	170	61
CARTPT	3	0	2	0	2	0
	0	0	3	0	2	0
CASC1	28	3	17	0	18	2
	12	4	23	0	14	2
CASC3	139	549	123	417	239	438
	504	556	216	164	343	108
CASC4	138	605	157	608	317	497
	515	636	241	192	343	118
CASC5	65	4	39	4	18	7
	1	1	29	2	13	0
CASD1	115	493	126	434	243	469
	392	603	297	131	327	80
CASK	88	223	63	166	127	115
	126	161	95	60	83	18
CASKIN1	8	6	9	3	10	5
	1	0	4	0	2	1
CASKIN2	33	99	28	55	40	37
	71	68	26	31	46	16
CASP1	29	59	27	33	27	34
	45	41	32	19	31	7
CASP10	103	122	59	78	53	66
	90	158	94	53	109	30
CASP12	9	5	9	10	4	3
	6	3	10	0	5	1
CASP14	18	0	14	0	5	0
	1	1	13	0	9	0
CASP16	7	1	5	1	3	0
	0	1	2	0	0	0
CASP2	33	85	26	56	57	59
	64	74	56	24	48	17
CASP3	53	168	53	156	97	170
	175	188	94	52	103	33
CASP4	25	80	26	41	33	49
	61	83	54	14	45	22
CASP5	11	0	4	1	7	0
	0	0	14	0	6	0
CASP6	14	27	9	26	13	23
	10	26	17	8	19	5
CASP7	51	87	33	83	53	97
	94	103	69	38	58	12
CASP8	26	38	10	26	24	20
	24	38	34	4	23	3

CASP8AP2	151	573	148	396	295	346
	424	549	220	196	287	113
CASP9	19	50	16	9	9	24
	25	37	22	1	20	9
CASQ1	3605	27381	4734	24209	11180	21531
	20831	17677	10351	4997	12080	2640
CASQ2	369	1912	649	706	762	1046
	1422	3515	885	439	890	426
CASR	36	0	22	1	6	0
	0	0	12	0	10	0
CASS4	21	6	13	2	5	1
	0	4	10	1	6	1
CAST	608	3965	660	2246	1488	2862
	2738	3166	1448	1310	1788	708
CASZ1	102	546	106	269	195	279
	296	470	126	135	180	112
CAT	287	992	229	661	629	588
	829	1435	513	301	669	223
CATSPER1	10	0	10	1	1	2
	0	0	5	1	4	0
CATSPER2	54	97	53	57	25	42
	54	84	48	25	66	12
CATSPER3	15	22	16	22	16	24
	22	29	13	12	19	8
CATSPER4	8	3	9	4	4	4
	0	4	9	1	2	1
CATSPERB	38	6	27	2	8	5
	10	14	21	1	13	1
CATSPERD	22	0	10	0	3	0
	0	1	9	0	3	0
CATSPERG	19	7	7	2	8	11
	5	9	15	0	6	0
CAV1	151	1054	97	677	473	488
	801	1262	449	352	460	262
CAV2	45	327	37	225	136	151
	207	372	171	90	159	89
CAV3	190	758	196	693	479	849
	777	1061	464	290	531	95
CBFA2T2	77	196	66	150	126	174
	189	174	112	73	124	22
CBFA2T3	16	56	11	59	18	56
	20	28	22	7	13	7
CBFB	78	295	84	197	172	269
	237	426	125	70	135	58
CBL	78	173	72	117	88	141
	162	178	78	50	83	50
CBLB	64	196	67	157	105	100
	152	252	89	70	113	50
CBLC	7	0	6	0	2	0
	0	0	2	0	1	0
CBLL1	114	659	122	524	346	431
	602	639	247	175	392	107
CBLN1	8	2	4	0	3	1
	2	8	1	1	4	0
CBLN2	13	0	8	0	4	0
	0	1	3	0	6	0
CBLN3	9	2	8	0	2	1
	2	1	3	3	5	0

CBLN4	12	9	10	19	21	7
	4	5	13	3	5	2
CBR1	27	146	16	82	60	82
	101	137	83	39	70	22
CBR3	10	27	8	20	6	14
	15	35	7	5	7	4
CBR4	73	421	96	303	227	372
	440	423	220	125	294	75
CBS	10	3	5	1	7	1
	2	4	5	0	5	1
CBWD1	42	381	67	234	94	184
	385	180	214	70	157	57
CBWD2	80	454	76	329	155	310
	358	406	225	96	284	61
CBWD3	35	80	29	58	41	65
	90	166	53	37	71	15
CBWD5	29	26	42	55	36	59
	86	117	75	32	73	13
CBWD6	33	92	49	94	51	68
	118	91	46	45	61	12
CBWD7	2	0	1	1	0	0
	0	2	0	0	0	0
CBX1	94	408	87	321	207	375
	323	455	168	128	224	84
CBX2	18	11	8	3	2	3
	5	3	12	3	6	0
CBX3	98	481	101	342	220	422
	571	626	277	161	292	74
CBX4	18	32	11	5	15	16
	16	30	19	6	14	2
CBX5	201	775	231	768	349	657
	450	715	304	226	364	140
CBX6	11	29	8	24	18	32
	23	44	24	11	7	9
CBX7	53	195	35	111	85	129
	194	222	86	55	109	35
CBX8	8	18	7	1	3	12
	4	12	7	2	8	1
CBY1	21	57	12	45	26	54
	48	57	34	17	24	10
CBY3	0	0	1	0	0	0
	0	0	0	0	1	0
CC2D1A	39	107	25	74	24	51
	66	84	48	29	55	16
CC2D1B	70	216	49	138	116	148
	165	249	113	85	142	40
CC2D2A	92	147	51	85	76	66
	74	109	84	35	74	36
CC2D2B	20	5	11	5	4	3
	9	8	11	0	4	3
CCAR1	147	813	184	489	375	529
	571	742	300	259	437	186
CCBE1	24	5	10	3	6	1
	0	8	25	0	16	3
CCBL1	12	23	11	13	15	16
	22	31	10	9	14	0
CCBL2	43	115	35	82	28	88
	91	115	73	28	59	16

CCBP2	31	1	6	13	2	3
	0	5	8	0	8	4
CCDC101	44	84	22	66	43	104
	111	150	56	63	77	19
CCDC102A	3	4	1	4	4	8
	8	7	9	2	6	1
CCDC102B	23	35	23	19	21	6
	17	30	25	3	19	4
CCDC103	10	0	2	1	1	0
	0	0	5	0	0	0
CCDC104	34	63	20	57	26	58
	82	81	41	25	55	14
CCDC105	4	0	2	0	0	0
	0	0	3	0	0	0
CCDC106	9	29	5	20	9	29
	23	28	10	10	18	2
CCDC107	15	43	9	45	22	46
	49	72	26	21	31	10
CCDC108	41	0	18	0	7	1
	1	0	15	0	8	0
CCDC109B	16	18	12	14	9	11
	10	31	19	6	12	4
CCDC11	24	25	16	22	15	36
	20	34	27	12	22	7
CCDC110	29	61	16	38	35	85
	50	25	38	18	25	7
CCDC111	42	147	37	85	78	86
	96	129	61	28	68	12
CCDC112	36	75	17	56	44	93
	27	80	47	34	39	13
CCDC113	57	116	35	58	42	67
	83	93	52	26	55	20
CCDC114	18	0	8	1	1	1
	0	0	2	0	3	0
CCDC115	16	53	14	33	19	45
	49	33	36	12	24	10
CCDC116	5	1	2	0	1	0
	0	1	1	0	0	0
CCDC117	67	323	84	300	188	258
	267	341	161	91	199	58
CCDC12	15	87	12	44	33	57
	94	128	25	25	42	6
CCDC120	21	4	5	5	5	3
	1	7	5	0	4	1
CCDC121	15	60	23	31	27	41
	44	49	22	21	28	13
CCDC122	5	33	16	22	7	23
	5	30	12	6	14	1
CCDC124	12	105	11	43	47	46
	67	40	24	22	42	9
CCDC125	35	65	22	32	26	25
	39	55	25	15	22	12
CCDC126	32	114	26	105	57	89
	141	176	73	37	80	28
CCDC127	52	180	53	156	113	171
	156	225	102	59	131	29
CCDC129	55	1	34	0	10	0
	0	0	25	0	15	0

CCDC13	15	13	10	8	7	11
	4	6	20	3	10	2
CCDC130	17	67	10	43	37	52
	71	114	38	21	53	22
CCDC132	104	413	113	306	212	341
	289	549	256	147	317	100
CCDC134	18	31	10	26	14	30
	17	31	12	3	22	5
CCDC135	25	0	7	0	4	0
	0	0	15	0	7	1
CCDC136	44	47	24	42	27	39
	37	68	30	22	18	10
CCDC137	20	35	10	22	17	31
	13	38	21	10	20	7
CCDC138	23	25	15	24	24	14
	28	31	30	22	22	6
CCDC14	56	265	100	199	98	231
	148	288	131	96	176	58
CCDC140	12	1	9	1	2	2
	3	2	6	1	7	1
CCDC141	224	406	95	226	287	188
	263	974	174	142	230	131
CCDC142	31	57	17	25	28	24
	31	53	20	22	24	9
CCDC144A	147	525	71	163	397	371
	386	529	108	92	290	132
CCDC144NL	95	9	69	2	32	7
	12	6	54	1	48	1
CCDC146	41	93	42	63	38	78
	75	79	53	28	28	27
CCDC147	35	39	20	30	37	56
	42	66	50	23	65	13
CCDC148	65	7	37	8	10	4
	4	9	40	2	25	1
CCDC149	34	103	34	89	51	85
	68	79	53	31	38	9
CCDC15	37	20	17	14	24	21
	20	9	27	1	26	1
CCDC150	48	12	29	15	11	4
	3	4	27	8	21	0
CCDC151	8	0	3	0	2	1
	0	0	2	1	2	0
CCDC152	34	88	38	61	71	121
	90	270	87	25	110	35
CCDC153	4	3	3	0	2	2
	3	2	4	0	3	1
CCDC154	5	1	5	4	4	5
	2	9	3	0	3	0
CCDC155	18	0	10	0	2	0
	0	0	7	0	2	0
CCDC157	9	3	6	4	2	1
	6	6	8	1	6	0
CCDC158	33	5	33	7	16	3
	1	3	21	0	20	0
CCDC159	13	12	12	11	6	8
	22	12	16	5	12	5
CCDC160	12	9	6	23	17	26
	7	4	6	4	5	0

CCDC164	19	0	10	0	6	1
	3	0	14	0	7	0
CCDC166	2	0	0	0	0	0
	0	0	1	0	0	0
CCDC167	16	21	2	20	8	13
	23	31	15	4	15	2
CCDC168	161	7	110	4	57	9
	3	8	101	3	57	2
CCDC169	1	17	3	9	5	16
	12	1	2	1	2	0
CCDC169-SOHLH2		20	27	36	3	10
	31	9	12	14	6	10
	7					
CCDC17	22	32	15	27	16	28
	23	31	22	8	23	6
CCDC170	38	8	12	16	19	4
	3	18	26	3	17	7
CCDC171	60	88	80	105	50	123
	78	80	72	38	96	21
CCDC172	14	0	3	0	7	0
	0	0	9	0	3	0
CCDC173	23	0	12	1	5	0
	2	3	11	0	2	1
CCDC174	49	166	54	101	70	107
	174	192	84	56	124	32
CCDC175	20	3	24	0	11	9
	2	3	17	2	12	1
CCDC176	24	18	5	19	16	31
	20	31	18	5	26	1
CCDC177	11	0	6	0	2	0
	0	0	0	0	5	0
CCDC178	33	0	21	0	12	0
	0	1	25	0	15	0
CCDC18	69	176	62	105	86	144
	134	261	105	56	88	50
CCDC19	11	4	7	2	3	1
	0	5	10	1	2	2
CCDC22	27	66	21	52	24	41
	47	82	29	34	49	12
CCDC23	19	119	18	41	48	98
	88	118	50	29	47	11
CCDC24	12	3	1	1	1	3
	4	6	4	0	1	0
CCDC25	82	468	91	408	210	528
	481	456	219	147	282	53
CCDC27	12	0	8	0	4	2
	1	0	7	0	1	0
CCDC28A	22	81	29	68	41	67
	118	131	37	42	59	15
CCDC28B	72	292	51	280	119	252
	327	342	165	95	164	36
CCDC3	26	71	19	90	24	156
	56	37	25	8	32	22
CCDC30	40	12	20	8	20	10
	7	9	22	2	8	1
CCDC33	28	0	20	0	4	0
	0	0	11	0	4	0

CCDC34	15	26	13	8	12	13
	8	18	10	5	14	1
CCDC36	24	39	11	28	38	13
	35	37	30	6	21	5
CCDC37	16	1	8	1	1	0
	1	0	5	0	5	0
CCDC38	31	0	14	0	3	0
	0	2	15	0	4	0
CCDC39	54	65	52	41	57	58
	71	62	35	40	41	35
CCDC40	29	7	12	9	5	4
	3	8	14	8	3	2
CCDC41	69	255	62	157	100	161
	187	224	109	88	148	34
CCDC42	8	0	7	0	0	0
	0	0	4	0	0	0
CCDC42B	2	0	3	0	2	0
	0	3	0	0	1	0
CCDC43	147	774	152	525	402	594
	606	772	365	259	494	124
CCDC47	208	1468	281	1208	547	1076
	940	1075	515	367	733	225
CCDC48	11	20	3	12	4	9
	13	11	11	4	11	4
CCDC50	223	925	224	762	532	880
	872	1353	559	359	697	225
CCDC51	14	71	24	55	45	59
	65	72	39	18	45	8
CCDC53	42	234	31	117	80	129
	215	238	114	61	155	39
CCDC54	3	0	3	0	2	0
	0	0	2	0	1	0
CCDC57	40	100	36	35	34	38
	61	70	32	21	30	9
CCDC58	46	168	24	128	62	95
	179	298	92	59	102	24
CCDC59	41	195	38	153	120	139
	270	301	98	78	141	40
CCDC6	78	441	91	431	173	349
	266	336	190	101	157	78
CCDC60	21	3	14	1	7	1
	3	2	11	0	6	0
CCDC61	3	13	1	7	6	10
	4	13	4	2	4	0
CCDC62	49	31	32	31	20	45
	13	40	31	11	25	8
CCDC63	14	9	11	2	4	0
	1	1	9	0	3	0
CCDC64	12	3	6	0	4	0
	0	1	8	0	4	0
CCDC64B	4	0	2	0	1	0
	0	0	2	0	2	0
CCDC65	17	2	9	1	5	0
	0	3	6	0	6	0
CCDC66	80	337	75	204	193	230
	235	434	151	122	205	65
CCDC67	13	0	15	2	3	0
	0	0	10	0	12	0

CCDC68	34	43	19	40	22	30
	53	79	46	23	40	12
CCDC69	317	1794	295	1973	797	1913
	1685	963	514	497	822	198
CCDC7	52	190	47	143	49	90
	221	177	66	49	110	22
CCDC70	8	0	4	0	1	0
	0	0	2	0	1	0
CCDC71	12	38	6	19	21	35
	31	60	35	21	42	7
CCDC71L	16	31	12	40	28	20
	22	40	24	8	27	4
CCDC73	34	3	13	2	12	2
	1	4	18	0	15	0
CCDC74A	8	0	10	0	0	0
	0	5	3	0	1	1
CCDC74B	5	2	1	1	1	0
	0	0	1	0	1	0
CCDC75	20	40	11	18	29	32
	45	47	20	13	21	5
CCDC77	38	49	21	37	38	42
	43	77	30	20	51	13
CCDC78	7	0	3	0	0	0
	0	0	2	0	1	0
CCDC79	22	0	5	2	5	0
	0	0	9	0	9	1
CCDC8	14	70	29	26	11	32
	9	20	15	4	14	5
CCDC80	83	658	144	550	263	258
	224	304	120	89	219	137
CCDC81	25	9	24	13	7	13
	4	16	14	5	15	5
CCDC82	66	281	76	223	105	258
	296	357	147	102	183	49
CCDC83	22	0	6	0	2	0
	0	0	10	0	8	0
CCDC84	32	88	18	84	31	96
	83	159	66	35	68	24
CCDC85A	38	105	47	50	68	43
	129	137	68	45	55	9
CCDC85B	8	68	14	40	25	36
	24	36	15	6	20	4
CCDC85C	38	84	18	73	31	40
	55	100	29	23	45	11
CCDC86	16	42	10	16	22	36
	42	43	11	11	19	7
CCDC87	10	1	3	1	0	0
	0	0	4	0	4	2
CCDC88A	199	974	216	660	415	615
	552	774	424	228	412	152
CCDC88B	16	12	19	11	3	4
	4	5	10	3	4	0
CCDC88C	52	95	45	45	23	43
	41	32	57	19	44	6
CCDC89	11	9	6	7	5	16
	3	12	5	1	5	1
CCDC9	18	78	18	26	43	41
	54	64	40	28	45	11

CCDC90A	76	293	47	216	116	262
	429	388	162	107	139	32
CCDC90B	62	261	59	202	115	190
	225	290	125	67	149	39
CCDC91	57	383	48	233	136	217
	261	304	183	92	160	65
CCDC92	50	316	35	114	75	192
	249	173	77	86	110	27
CCDC93	144	587	143	331	266	401
	389	558	266	194	362	99
CCDC94	18	36	19	45	33	41
	44	66	35	21	48	12
CCDC96	1	0	2	3	0	0
	0	0	1	0	0	0
CCDC97	39	129	21	89	73	85
	151	130	59	36	52	28
CCER1	13	0	4	0	1	0
	0	0	3	0	1	0
CCHCR1	37	122	29	122	46	86
	101	109	36	21	44	22
CCIN	13	0	6	2	1	0
	0	1	0	0	4	0
CCK	9	0	2	0	4	0
	0	0	0	0	3	0
CCKAR	9	1	3	0	0	0
	1	0	7	0	2	0
CCKBR	7	0	3	0	0	0
	0	0	3	0	1	0
CCL1	2	0	4	0	0	0
	0	0	0	0	0	0
CCL11	9	0	2	1	1	0
	0	0	5	0	0	0
CCL13	6	1	6	4	12	3
	3	14	5	0	7	1
CCL14	11	46	10	18	38	17
	58	32	13	8	20	12
CCL15	1	0	2	0	0	0
	0	0	1	0	1	0
CCL16	6	3	5	0	2	1
	0	1	3	2	2	1
CCL17	3	0	2	0	0	0
	0	0	1	0	2	0
CCL18	1	0	2	0	0	0
	0	1	1	0	0	0
CCL19	5	1	2	1	0	0
	0	0	1	0	2	0
CCL2	6	16	8	25	5	28
	14	17	9	4	9	2
CCL20	3	0	3	0	1	0
	0	0	7	0	0	0
CCL21	4	10	1	8	5	1
	3	26	0	5	0	6
CCL22	9	1	5	0	2	0
	0	0	8	0	3	0
CCL23	6	0	2	0	1	0
	0	0	1	0	3	1
CCL24	0	0	0	0	1	1
	0	0	2	1	0	0

CCL25	1	0	3	0	0	0
	0	0	0	0	1	0
CCL26	1	1	1	0	1	0
	0	0	1	0	0	0
CCL27	3	0	2	0	2	0
	0	1	1	0	0	0
CCL28	8	0	1	0	5	0
	0	5	3	0	1	1
CCL3	7	0	4	0	1	0
	0	1	0	0	2	0
CCL3L1	2	0	3	0	0	0
	0	0	1	0	0	0
CCL3L3	3	0	0	0	0	0
	0	0	1	0	0	0
CCL4	1	0	0	0	0	0
	0	0	1	0	0	0
CCL4L2	0	0	1	0	0	0
	0	0	0	0	0	0
CCL5	13	9	7	6	2	5
	3	9	16	0	8	2
CCL7	1	0	4	0	0	0
	0	0	5	0	0	0
CCL8	8	0	4	2	1	1
	0	0	2	0	2	0
CCM2	33	131	23	96	39	104
	63	93	58	26	40	12
CCNA1	6	0	16	0	5	0
	0	1	5	0	7	0
CCNA2	18	7	9	10	2	16
	15	20	19	4	14	2
CCNB1	17	22	12	8	9	19
	23	20	18	6	27	2
CCNB1IP1	57	244	43	184	96	216
	228	431	187	87	186	46
CCNB2	15	1	14	0	2	1
	0	3	5	0	6	0
CCNB3	37	9	16	1	15	6
	7	5	8	1	5	0
CCNC	55	210	49	153	108	154
	209	348	151	62	162	40
CCND1	19	74	27	53	38	44
	47	83	50	25	29	26
CCND2	75	170	39	197	107	143
	119	149	111	25	74	99
CCND3	6	44	8	19	11	15
	37	47	29	9	29	10
CCNDBP1	98	376	85	344	213	330
	357	480	245	136	241	54
CCNE1	11	5	5	2	6	1
	8	14	9	2	9	1
CCNE2	14	2	15	2	3	3
	4	17	9	5	16	3
CCNF	16	6	19	2	16	4
	8	13	19	0	14	5
CCNG1	315	1560	489	1694	884	1572
	1956	2223	1807	493	1436	364
CCNG2	101	193	56	235	100	175
	124	191	77	36	92	49

CCNH	77	276	64	212	112	204
	242	314	126	74	152	57
CCNI	183	1353	218	1056	468	917
	1121	1113	462	308	551	198
CCNI2	14	2	6	2	3	1
	0	2	10	0	4	0
CCNJ	53	79	17	65	42	48
	72	72	32	30	63	14
CCNJL	23	7	5	5	1	3
	1	0	12	0	5	0
CCNK	33	240	38	168	93	130
	173	190	48	47	87	30
CCNL1	69	336	84	309	162	271
	343	548	205	124	245	85
CCNL2	139	571	121	401	246	481
	453	847	288	203	340	108
CCNO	3	0	5	0	0	0
	0	0	2	0	1	0
CCNT1	141	619	164	469	328	490
	511	720	335	214	381	119
CCNT2	137	676	142	476	323	490
	510	900	388	224	429	149
CCNY	110	396	92	341	184	311
	358	394	181	119	213	81
CCNYL1	33	148	30	106	55	76
	93	90	75	23	57	16
CCNYL2	12	0	11	0	5	0
	0	0	10	0	5	0
CCP110	74	230	59	158	117	165
	133	209	103	66	133	44
CCPG1	253	1064	253	1041	593	940
	633	1414	575	403	650	269
CCR1	14	5	8	4	10	12
	6	21	6	4	10	3
CCR10	1	0	4	8	0	5
	0	1	4	0	4	0
CCR2	11	2	15	5	7	5
	4	6	15	3	12	0
CCR3	26	9	8	15	9	5
	24	28	15	9	28	9
CCR4	8	0	6	0	0	0
	0	0	2	1	3	0
CCR5	35	0	16	3	15	4
	5	4	17	3	13	1
CCR6	27	0	10	0	5	1
	1	6	12	1	4	0
CCR7	11	3	1	1	0	2
	0	2	5	0	2	0
CCR8	16	0	6	0	1	0
	0	0	3	0	0	0
CCR9	18	1	7	2	2	1
	0	1	3	0	1	0
CCRL1	13	4	1	13	4	17
	0	4	6	1	10	0
CCRL2	11	11	6	6	3	5
	7	6	12	2	6	0
CCRN4L	20	100	23	57	41	50
	110	81	38	47	64	8

CCS	16	40	12	26	14	31
	37	67	28	15	35	3
CCSAP	49	110	56	69	65	85
	95	100	58	20	79	24
CCT2	112	528	106	383	274	404
	394	624	281	185	376	90
CCT3	144	837	181	526	441	589
	536	994	353	244	432	144
CCT4	132	775	189	551	389	609
	547	999	386	228	486	140
CCT5	166	1070	192	633	499	743
	676	1191	423	258	514	149
CCT6A	161	1166	197	791	586	951
	824	1361	485	337	657	200
CCT6B	24	69	45	45	35	45
	54	58	40	11	60	9
CCT7	190	993	204	701	526	786
	796	1118	462	314	550	167
CCT8	210	1334	228	765	622	788
	903	1339	479	311	571	180
CCT8L2	3	0	4	0	1	1
	0	0	3	0	2	0
CCZ1	82	491	47	288	88	216
	268	312	133	65	184	65
CCZ1B	65	165	67	227	120	270
	325	328	201	60	181	54
CD101	29	6	22	4	10	3
	3	2	16	0	10	0
CD109	75	204	59	112	80	65
	123	200	137	43	105	35
CD14	8	9	2	12	7	18
	3	27	19	3	9	3
CD151	49	199	16	134	104	90
	119	147	71	46	63	33
CD160	11	4	10	4	8	11
	3	9	7	4	10	1
CD163	62	85	48	113	92	141
	59	221	119	58	110	36
CD163L1	53	27	36	19	33	32
	18	65	43	12	52	7
CD164	262	1367	289	1180	870	1086
	1206	2151	724	386	1095	285
CD164L2	5	0	3	0	1	0
	0	0	7	0	4	0
CD177	14	0	14	6	4	0
	3	0	7	1	5	0
CD180	20	2	8	2	5	3
	1	5	6	2	3	0
CD19	10	0	4	0	1	0
	0	0	1	0	2	1
CD1A	19	0	12	0	3	0
	1	0	9	0	5	0
CD1B	8	0	3	0	0	0
	0	0	5	0	3	0
CD1C	27	5	14	4	11	5
	3	7	18	5	5	0
CD1D	18	35	16	6	23	31
	24	16	23	4	7	5

CD1E	14	2	12	0	1	1
	0	0	7	2	7	0
CD2	13	8	8	4	5	3
	2	11	13	0	4	0
CD200	13	11	11	12	12	5
	8	18	19	6	14	2
CD200R1	9	7	20	11	15	6
	8	14	11	6	4	0
CD200R1L	9	0	11	0	0	0
	0	0	7	0	5	0
CD207	22	1	17	1	5	0
	0	0	9	2	4	3
CD209	33	6	13	13	34	16
	2	57	19	6	38	4
CD22	25	9	11	7	3	5
	4	8	16	2	3	3
CD226	24	43	16	24	14	31
	12	45	28	15	20	5
CD244	20	1	13	2	5	5
	0	3	11	0	6	1
CD247	11	2	15	6	7	1
	5	8	14	1	11	1
CD248	18	61	22	88	28	48
	15	45	28	10	30	11
CD27	3	2	4	0	2	3
	1	2	3	0	1	0
CD274	42	97	68	109	109	94
	75	68	51	21	47	13
CD276	19	50	20	40	29	49
	26	48	24	8	26	7
CD28	25	10	12	10	14	8
	6	6	8	0	7	1
CD2AP	91	375	108	256	196	296
	326	408	227	129	221	76
CD2BP2	66	204	51	139	93	140
	152	270	116	65	108	47
CD300A	8	5	5	1	4	3
	4	2	12	1	11	0
CD300C	7	1	1	1	1	1
	0	2	3	0	2	0
CD300E	21	10	13	4	6	4
	7	1	13	2	11	0
CD300LB	12	1	6	1	4	1
	0	1	3	0	3	0
CD300LD	2	0	4	0	1	0
	0	0	2	0	0	0
CD300LF	16	1	5	1	1	4
	3	4	2	0	2	0
CD300LG	22	47	11	16	24	21
	79	12	25	11	24	8
CD302	1	29	5	34	20	18
	47	152	19	16	31	31
CD320	8	27	5	16	9	17
	11	22	7	11	8	1
CD33	12	4	7	2	3	5
	2	8	12	4	3	0
CD34	61	439	33	243	156	142
	317	247	146	81	179	58

CD36	2033	11016	1170	4022	5508	4768
	6713	12498	3301	2909	3785	1266
CD37	15	11	8	6	5	5
	3	12	6	3	5	0
CD38	76	505	93	386	177	346
	345	269	216	77	268	57
CD3D	7	2	4	5	1	2
	0	4	10	0	4	1
CD3E	6	4	14	1	3	0
	1	3	2	0	2	1
CD3EAP	28	53	21	27	47	57
	51	23	20	17	23	7
CD3G	11	3	6	3	1	1
	0	3	7	0	3	0
CD4	18	16	8	19	15	15
	10	40	23	4	16	7
CD40	24	33	14	15	23	22
	39	51	21	4	22	10
CD40LG	19	4	8	3	8	1
	0	3	6	1	6	0
CD44	71	350	73	241	126	208
	171	433	124	80	132	86
CD46	209	1020	199	815	488	937
	796	1157	575	337	602	206
CD47	92	376	102	323	158	274
	274	606	207	131	263	93
CD48	15	8	6	3	7	6
	5	29	9	3	7	0
CD5	22	1	6	4	3	1
	2	3	7	1	6	0
CD52	5	16	3	2	5	5
	2	17	6	2	4	0
CD53	31	22	15	19	17	15
	14	50	24	3	20	5
CD55	105	372	59	400	272	360
	457	651	306	153	372	127
CD58	19	49	25	29	44	53
	51	69	43	14	29	13
CD59	830	3738	855	3093	2399	2743
	3338	4758	1816	1106	2479	726
CD5L	9	0	8	0	2	0
	0	0	4	0	2	0
CD6	17	5	2	0	3	1
	0	2	7	0	6	0
CD63	181	953	161	708	396	753
	881	1305	535	225	481	121
CD68	34	34	13	30	33	62
	22	90	34	21	46	14
CD69	10	3	6	0	0	2
	1	5	7	0	5	1
CD7	4	5	0	0	0	0
	1	0	0	0	0	0
CD70	8	0	3	1	1	0
	0	0	3	1	1	0
CD72	16	4	12	3	3	0
	0	3	8	0	8	1
CD74	138	798	100	458	275	476
	695	914	391	310	700	129

CD79A	0	0	2	0	0	2
	2	1	3	1	4	0
CD79B	6	17	4	6	3	6
	14	14	6	2	5	0
CD80	10	2	9	1	5	0
	0	2	10	1	5	0
CD81	24	134	30	128	55	73
	81	166	71	32	73	37
CD82	17	35	14	17	18	25
	24	28	16	10	21	4
CD83	17	18	12	14	17	27
	13	22	18	5	13	2
CD84	63	60	35	52	48	55
	44	60	53	7	41	17
CD86	27	9	17	4	16	9
	13	15	22	3	10	1
CD8A	24	2	10	6	7	0
	0	1	10	0	10	0
CD8B	15	3	4	1	0	1
	0	2	7	0	2	0
CD9	33	125	19	103	32	65
	69	102	47	34	64	37
CD93	130	816	95	433	353	353
	579	743	387	219	345	135
CD96	29	6	14	2	12	8
	4	13	22	1	16	0
CD97	29	131	35	106	58	101
	72	132	60	27	48	18
CD99 (NC_000023 2609227..2659350)				18	132	25
	102	44	88	95	104	50
	29	65	28			
CD99 (NC_000024 2559227..2609350)				25	148	23
	123	43	88	112	103	58
	31	46	29			
CD99L2	144	654	128	470	323	510
	579	683	308	187	347	117
CDA	5	0	5	0	1	1
	0	1	0	0	2	1
CDADC1	45	156	44	90	71	91
	113	154	71	42	89	20
CDAN1	43	59	22	36	44	43
	47	79	50	25	36	8
CDC123	70	281	61	296	122	224
	324	448	205	78	222	54
CDC14A	37	84	37	67	41	57
	46	72	53	27	53	21
CDC14B	67	48	45	67	40	56
	36	51	59	9	21	14
CDC16	78	468	96	386	167	263
	332	418	147	100	190	50
CDC20	10	2	9	0	4	0
	1	1	3	0	5	0
CDC20B	21	0	14	0	6	2
	0	0	11	0	4	0
CDC23	83	186	43	117	133	144
	189	201	133	61	155	41
CDC25A	27	3	16	4	8	5
	11	8	19	1	11	0

CDC25B	24	71	19	41	26	34
	38	40	26	13	25	15
CDC25C	18	0	11	0	3	0
	0	0	13	0	6	0
CDC26	34	162	19	144	70	127
	180	235	86	50	74	37
CDC27	1311	1029	540	667	630	656
	665	935	699	281	661	163
CDC34	61	290	35	169	133	170
	277	337	175	90	133	57
CDC37	290	1176	188	626	630	840
	945	1549	511	422	735	234
CDC37L1	132	536	113	497	223	433
	544	654	337	187	602	121
CDC40	79	354	80	254	198	263
	273	406	168	123	216	70
CDC42	259	1000	255	800	508	739
	912	1500	663	313	699	239
CDC42BPA	291	1400	357	1105	682	1034
	873	1139	587	400	647	214
CDC42BPB	132	647	138	434	296	424
	383	632	267	179	278	141
CDC42BPG	22	2	8	0	3	1
	0	0	10	1	7	1
CDC42EP1	3	14	3	10	5	9
	17	1	6	4	13	2
CDC42EP2	10	13	4	11	12	13
	3	10	12	4	9	6
CDC42EP3	100	415	204	516	242	508
	386	253	180	123	211	111
CDC42EP4	13	38	10	44	22	19
	56	62	28	12	40	15
CDC42EP5	0	7	0	4	5	2
	3	6	2	1	2	2
CDC42SE1	50	256	66	160	107	186
	159	303	93	63	145	49
CDC42SE2	74	281	57	222	154	231
	176	298	124	73	197	48
CDC45	17	0	12	0	6	2
	3	0	9	0	6	0
CDC5L	235	1076	266	667	569	782
	734	1205	484	396	743	208
CDC6	27	6	12	7	9	3
	6	5	23	2	10	0
CDC7	24	6	11	13	6	10
	5	3	12	2	18	1
CDC73	138	573	189	512	262	544
	468	574	330	213	354	103
CDCA2	30	4	13	1	6	5
	3	4	13	1	10	0
CDCA3	10	1	2	1	3	0
	1	0	1	0	1	0
CDCA4	10	20	7	9	7	12
	20	7	6	5	13	1
CDCA5	19	4	4	2	2	3
	3	4	4	0	5	0
CDCA7	31	27	23	5	37	20
	27	39	15	3	21	5

CDCA7L	64	83	52	63	61	56
	120	73	45	34	69	13
CDCA8	19	2	17	1	4	0
	1	2	4	0	12	0
CDCP1	30	2	10	4	7	2
	1	5	17	1	3	3
CDCP2	17	0	4	0	2	1
	1	0	0	0	2	0
CDH1	39	8	14	3	7	4
	0	0	21	3	6	1
CDH10	25	1	14	0	7	1
	2	0	20	0	9	1
CDH11	48	56	35	111	25	33
	55	58	21	16	31	15
CDH12	38	1	23	1	14	1
	0	0	21	0	10	0
CDH13	176	941	214	819	339	697
	781	817	487	231	598	146
CDH15	40	174	27	93	49	129
	86	170	81	45	58	22
CDH16	13	0	7	0	1	0
	0	0	4	0	4	0
CDH17	36	3	21	0	15	0
	1	2	14	1	9	0
CDH18	20	5	23	4	18	1
	4	0	18	1	10	0
CDH19	40	7	45	55	13	4
	20	15	39	6	23	10
CDH2	37	19	33	11	12	1
	5	5	32	2	26	4
CDH20	33	17	27	33	3	47
	46	13	39	2	34	11
CDH22	17	1	9	2	5	1
	1	0	3	0	6	0
CDH23	69	97	49	44	48	32
	69	66	72	29	51	16
CDH24	13	0	7	2	5	0
	6	1	4	0	1	0
CDH26	41	24	25	38	23	44
	17	36	30	8	21	5
CDH3	27	0	10	0	1	1
	0	1	17	0	3	0
CDH4	26	12	7	4	8	3
	4	8	19	0	8	1
CDH5	90	362	50	219	170	179
	366	295	251	137	196	75
CDH6	67	87	41	55	46	33
	33	82	78	20	38	12
CDH7	35	1	20	1	12	0
	0	2	15	0	7	0
CDH8	49	17	15	6	13	4
	12	9	25	3	10	1
CDH9	20	0	17	1	9	0
	0	0	18	0	11	0
CDHR1	35	0	20	0	6	0
	0	0	16	0	10	0
CDHR2	21	1	30	0	3	1
	1	0	18	0	13	0

CDHR3	46	7	35	14	8	21
	21	12	34	4	18	1
CDHR4	10	0	5	0	4	0
	0	0	4	0	6	1
CDHR5	15	0	13	0	8	3
	2	2	10	2	7	1
CDIPT	24	69	26	69	30	55
	67	129	57	23	61	16
CDK1	23	10	17	7	7	8
	6	10	15	5	9	1
CDK10	17	47	13	26	15	27
	21	56	25	16	28	4
CDK11A	36	139	23	53	61	53
	67	124	30	68	60	43
CDK11B	34	93	18	59	56	70
	76	86	47	48	46	14
CDK12	145	451	108	291	207	283
	335	472	177	161	265	110
CDK13	150	687	175	529	329	480
	496	700	301	215	393	151
CDK14	75	250	49	170	107	144
	132	166	112	64	94	27
CDK15	41	17	21	26	24	34
	23	20	25	5	26	2
CDK16	142	696	111	465	299	466
	633	692	314	235	359	109
CDK17	77	280	84	188	119	155
	226	312	154	100	184	64
CDK18	26	13	15	7	12	10
	13	2	9	6	6	2
CDK19	61	88	36	110	69	75
	53	101	65	32	72	22
CDK2	19	33	12	38	31	33
	42	58	25	24	22	5
CDK20	11	5	12	5	2	7
	2	7	6	4	4	2
CDK2AP1	79	378	59	392	162	306
	365	312	160	92	154	44
CDK2AP2	8	14	2	15	5	9
	8	19	10	7	11	5
CDK3	11	17	9	5	5	11
	7	12	13	6	7	2
CDK4	29	101	30	86	62	80
	117	153	69	22	65	21
CDK5	14	9	13	8	4	15
	14	16	15	5	8	2
CDK5R1	17	21	9	5	7	11
	11	21	11	3	15	2
CDK5R2	9	0	1	0	1	0
	0	0	1	0	1	0
CDK5RAP1	60	210	58	169	100	146
	228	263	106	68	111	47
CDK5RAP2	323	1525	358	877	626	952
	747	1032	424	487	531	222
CDK5RAP3	53	188	53	121	71	134
	121	257	109	47	122	24
CDK6	114	237	87	176	144	216
	207	326	173	101	163	60

CDK7	31	98	28	84	57	104
	120	183	81	42	65	23
CDK8	61	207	45	146	93	134
	138	234	102	72	139	35
CDK9	33	145	46	100	53	92
	125	171	58	33	78	16
CDKAL1	97	512	102	301	283	354
	419	479	231	163	305	74
CDKL1	10	14	15	13	6	7
	7	5	11	1	14	0
CDKL2	22	3	14	1	5	5
	1	8	21	3	7	2
CDKL3	30	74	35	73	28	58
	66	89	46	15	62	16
CDKL4	14	4	6	2	2	5
	1	4	12	1	2	3
CDKL5	39	67	29	39	37	48
	50	55	20	22	33	6
CDKN1A	14	89	45	46	11	60
	17	19	13	4	15	20
CDKN1B	97	527	100	354	278	429
	495	805	305	185	361	85
CDKN1C	28	84	15	57	47	89
	93	197	61	48	85	38
CDKN2A	6	0	8	0	3	0
	0	0	1	0	2	0
CDKN2AIP	40	125	34	109	68	89
	104	182	68	51	69	27
CDKN2AIPNL	74	383	67	244	161	280
	364	450	189	107	172	36
CDKN2B	34	85	44	58	64	53
	33	114	46	16	25	29
CDKN2C	24	54	12	46	20	36
	148	132	35	9	62	20
CDKN2D	8	19	8	14	8	19
	19	26	14	7	14	3
CDKN3	8	10	9	9	9	13
	9	14	12	2	10	0
CDNF	39	293	53	136	98	151
	258	154	84	74	120	29
CDO1	22	48	15	73	23	52
	36	49	36	14	23	15
CDON	85	106	45	122	50	67
	55	108	83	27	66	18
CDPF1	16	27	11	23	11	11
	14	27	18	12	13	9
CDR1	18	71	8	27	45	38
	31	65	10	5	15	36
CDR2	22	47	6	39	23	21
	28	35	28	12	19	7
CDR2L	16	16	13	11	12	12
	18	30	9	5	6	4
CDRT1	31	27	18	13	17	16
	28	25	23	5	16	2
CDRT15L2	6	0	3	0	0	0
	0	0	2	0	2	0
CDRT4	1	3	0	0	0	0
	0	0	0	0	0	0

CDS1	19	1	23	8	12	2
	0	4	12	0	12	0
CDS2	153	722	176	622	264	411
	529	747	314	181	338	111
CDSN	21	0	10	0	2	0
	0	0	5	0	5	0
CDT1	11	0	6	0	0	2
	0	0	5	0	2	0
CDV3	441	2806	492	1888	1114	2327
	2087	2800	1236	941	1442	445
CDX1	7	0	3	0	3	1
	0	0	4	0	2	0
CDX2	6	0	2	0	0	0
	0	0	1	0	1	0
CDX4	2	0	3	0	1	0
	0	0	1	0	2	0
CDY1	0	0	5	0	0	0
	0	0	7	0	5	0
CDY1B	0	0	2	0	0	0
	0	0	1	0	3	0
CDY2A	0	0	0	0	0	0
	0	0	0	0	1	0
CDY2B	0	0	5	0	0	0
	0	0	2	0	1	0
CDYL	76	322	71	194	136	217
	220	368	127	112	140	61
CDYL2	9	8	10	2	12	5
	11	14	15	5	3	2
CEACAM1	32	34	13	31	18	18
	29	26	24	16	20	2
CEACAM16	9	0	4	1	1	0
	0	1	6	0	2	1
CEACAM18	6	0	5	0	4	0
	0	0	4	0	2	0
CEACAM19	15	31	11	16	10	43
	29	17	13	12	31	2
CEACAM20	11	0	15	0	1	2
	0	0	8	0	4	0
CEACAM21	8	3	8	0	5	0
	0	1	8	0	1	0
CEACAM3	6	0	9	0	2	1
	0	0	6	0	0	0
CEACAM4	8	0	6	1	1	2
	0	0	3	0	2	0
CEACAM5	28	0	7	1	1	0
	0	0	11	0	4	0
CEACAM6	30	0	8	0	4	0
	0	1	15	0	4	0
CEACAM7	27	0	7	0	2	0
	0	0	5	2	6	0
CEACAM8	25	0	6	0	4	0
	0	0	11	0	6	0
CEBPA	10	173	26	24	143	146
	40	23	13	799	18	389
CEBPB	57	194	59	124	162	130
	434	637	245	131	215	127
CEBPD	17	45	18	27	66	28
	252	467	107	56	91	82

CEBPE	8	0	6	0	1	0
	0	1	3	0	2	0
CEBPG	150	985	127	881	446	1032
	1020	951	562	265	523	152
CEBPZ	135	614	140	381	282	448
	428	677	273	249	326	113
CECR1	27	38	18	19	24	25
	30	60	25	10	16	12
CECR2	139	592	161	287	303	391
	421	530	224	181	281	104
CECR5	21	72	21	48	41	61
	105	110	50	18	59	17
CECR6	28	20	10	20	10	7
	13	17	16	4	10	4
CEL	14	4	12	1	4	4
	2	4	6	0	6	1
CELA1	2	0	3	0	1	0
	0	0	3	0	1	0
CELA2A	12	0	4	0	0	0
	0	0	0	0	1	0
CELA2B	5	0	2	0	1	0
	0	0	1	0	1	0
CELA3A	7	0	3	0	0	0
	0	0	2	0	0	0
CELA3B	4	0	0	0	0	0
	0	0	1	0	3	0
CELF1	313	1182	280	1043	683	1000
	975	1237	554	398	726	227
CELF2	136	668	214	440	267	444
	409	679	267	138	279	99
CELF3	28	0	17	0	6	0
	0	0	14	0	4	0
CELF4	16	2	5	0	9	2
	1	2	3	1	6	0
CELF5	19	0	11	0	4	0
	0	1	9	0	4	0
CELF6	15	13	7	12	6	13
	9	18	16	4	18	4
CELSR1	47	10	27	1	7	1
	1	3	16	0	11	1
CELSR2	49	127	46	104	75	88
	85	102	47	37	48	22
CELSR3	30	4	24	2	10	4
	2	3	26	3	6	1
CEMP1	6	0	1	0	1	1
	0	2	2	0	1	1
CEND1	5	0	0	0	1	0
	0	2	0	0	1	0
CENPA	14	0	3	0	0	0
	0	1	5	0	2	0
CENPB	50	222	35	122	92	112
	125	267	89	73	109	39
CENPBD1	15	31	9	32	15	27
	20	21	21	9	16	5
CENPC1	75	307	88	221	171	169
	264	461	186	145	258	84
CENPE	79	10	37	4	40	6
	12	12	68	3	33	2

CENPF	86	23	56	11	39	14
	16	10	56	6	47	3
CENPH	15	23	14	15	13	14
	10	27	23	6	11	6
CENPI	31	8	2	13	13	1
	8	6	11	8	17	4
CENPJ	67	104	65	78	66	51
	78	97	64	23	61	21
CENPK	15	4	10	2	5	6
	3	5	18	3	14	1
CENPL	15	10	14	15	8	22
	11	35	16	8	5	5
CENPM	7	0	2	0	1	0
	0	0	3	1	4	0
CENPN	48	24	20	24	24	23
	22	31	27	7	30	4
CENPO	28	21	22	20	19	24
	38	22	18	11	21	1
CENPP	27	54	37	73	25	72
	69	97	49	24	37	10
CENPQ	29	46	15	23	21	27
	27	53	33	9	31	7
CENPT	32	108	31	60	34	90
	58	110	60	21	52	15
CENPV	8	52	7	25	25	55
	110	140	42	18	56	12
CENPW	4	1	4	0	0	3
	6	8	5	1	3	1
CEP104	93	275	60	177	143	174
	207	220	101	101	125	55
CEP112	99	436	84	188	174	183
	223	264	110	128	143	63
CEP120	70	182	67	151	109	158
	149	212	102	68	133	35
CEP128	49	52	44	37	42	50
	29	44	67	30	61	14
CEP135	49	97	50	42	39	55
	50	50	63	35	43	17
CEP152	46	44	39	31	53	36
	38	35	58	9	35	5
CEP164	60	85	43	53	38	59
	55	80	51	27	52	17
CEP170	384	1397	455	1259	866	1249
	1149	1400	667	482	944	260
CEP19	10	10	7	2	4	7
	5	2	13	0	8	1
CEP192	200	686	253	555	369	460
	576	883	374	213	497	157
CEP250	98	252	98	149	143	168
	159	265	101	95	169	63
CEP290	120	396	131	220	179	204
	204	297	189	111	224	71
CEP350	707	3339	898	2369	1742	2434
	2574	4269	1549	1296	2227	836
CEP41	63	45	35	32	19	16
	14	43	35	16	35	3
CEP44	103	252	126	243	149	227
	218	372	163	116	190	71

CEP55	19	0	11	2	5	1
	1	0	11	0	5	1
CEP57	88	369	90	226	202	253
	272	364	143	103	201	78
CEP57L1	45	123	47	81	63	108
	83	156	78	33	75	27
CEP63	149	706	164	366	318	548
	461	533	254	207	271	92
CEP68	193	664	139	506	460	577
	553	978	348	233	407	153
CEP70	82	462	93	250	168	273
	433	523	238	126	280	67
CEP72	20	10	6	10	2	3
	7	12	9	4	9	4
CEP76	40	67	25	36	33	37
	66	71	36	21	49	15
CEP78	30	48	29	44	35	35
	40	48	38	15	63	6
CEP85	369	1733	305	1254	737	1246
	1387	2157	870	539	1214	296
CEP85L	201	913	291	731	615	809
	925	810	592	331	674	217
CEP89	27	78	27	37	37	56
	36	58	29	18	52	15
CEP95	96	372	111	260	182	292
	332	498	196	182	269	103
CEP97	60	195	61	125	105	119
	134	195	91	59	142	40
CEPT1	60	216	68	156	124	190
	202	313	123	67	144	26
CER1	2	0	2	1	0	1
	0	0	1	0	1	0
CERCAM	28	29	14	23	20	19
	13	22	17	4	9	6
CERK	71	198	49	198	103	190
	157	201	93	57	87	37
CERKL	19	19	79	4	14	19
	8	2	17	0	12	0
CERS1	6	15	10	13	6	26
	9	11	10	8	12	4
CERS2	65	145	44	118	101	132
	167	217	108	52	100	35
CERS3	36	0	30	2	9	2
	0	2	15	0	12	0
CERS4	7	10	4	12	4	9
	5	8	11	5	5	4
CERS5	30	30	9	26	17	19
	27	57	27	17	21	3
CERS6	46	223	85	217	147	205
	257	260	110	75	152	41
CES1	32	29	21	17	31	16
	11	23	21	8	13	15
CES2	88	417	88	151	132	163
	249	272	82	65	98	63
CES3	134	299	66	184	149	281
	186	242	117	91	94	34
CES4A	21	11	18	3	5	8
	10	22	7	5	17	6

CES5A	19	0	15	0	6	0
	0	0	8	0	3	0
CETN1	5	0	2	0	0	0
	0	0	2	0	0	0
CETN2	19	90	23	74	36	95
	67	129	57	27	56	20
CETN3	34	243	47	117	107	153
	326	387	98	83	176	25
CETP	19	2	2	2	3	1
	2	0	3	0	3	0
CFB	31	12	18	18	12	12
	8	20	16	4	16	4
CFC1	7	0	4	0	0	0
	0	0	7	0	0	0
CFC1B	0	0	1	0	1	0
	0	0	0	0	1	0
CFD	54	220	54	227	190	232
	69	416	146	59	100	46
CFDP1	46	188	66	78	93	190
	134	179	69	55	78	20
CFH	122	487	149	461	280	314
	378	635	273	193	221	179
CFHR1	7	0	3	0	6	1
	0	1	3	0	0	1
CFHR2	8	0	3	0	1	0
	0	0	2	0	0	0
CFHR3	7	0	3	0	3	0
	0	0	1	0	0	0
CFHR4	8	0	5	0	2	0
	0	0	8	0	3	0
CFHR5	10	0	5	0	2	0
	0	0	5	0	8	0
CFI	20	84	16	59	37	41
	54	98	48	25	52	17
CFL1	58	327	67	215	129	186
	259	350	130	71	130	49
CFL2	1588	7442	1207	4496	4327	5690
	8876	12657	4345	2798	5834	1625
CFLAR	978	3603	890	2042	1744	2244
	2989	5011	1662	1554	2202	1300
CFP	4	4	4	2	1	9
	2	8	2	5	6	1
CFTR	58	0	39	0	17	1
	0	2	40	0	19	0
CGA	2	0	2	0	3	0
	0	0	4	0	3	0
CGB	0	0	3	0	0	0
	0	0	0	0	2	0
CGB1	4	0	0	1	0	0
	0	0	0	0	0	0
CGB2	1	0	0	0	0	1
	0	0	0	0	0	0
CGB7	1	1	2	1	0	3
	4	2	4	1	2	0
CGB8	0	0	0	0	0	0
	1	0	0	0	0	0
CGGBP1	187	950	204	816	571	783
	852	1025	456	315	552	158

CGN	26	0	19	1	7	0
	0	0	18	0	6	1
CGNL1	75	32	42	41	24	23
	17	41	47	12	38	10
CGREF1	20	18	9	9	9	8
	11	24	14	4	7	1
CGRRF1	26	97	24	112	49	113
	89	184	86	25	92	26
CH25H	0	0	1	1	1	0
	0	0	2	1	2	0
CHAC1	3	2	0	1	2	0
	2	1	1	2	2	2
CHAC2	10	22	9	18	9	28
	31	29	11	10	12	3
CHAD	10	7	9	8	6	5
	17	3	7	3	2	0
CHADL	7	3	4	6	0	2
	12	6	6	5	4	0
CHAF1A	20	60	25	36	31	50
	56	55	41	18	59	13
CHAF1B	42	112	36	122	89	281
	144	118	48	50	78	24
CHAMP1	31	100	18	62	51	56
	81	69	29	30	31	11
CHAT	18	0	6	0	3	0
	0	0	9	0	3	0
CHCHD1	22	107	15	88	56	76
	117	186	74	37	73	19
CHCHD10	142	1307	111	706	321	545
	1734	1019	334	233	517	124
CHCHD2	220	955	179	750	353	650
	1290	1682	598	281	603	146
CHCHD3	323	2031	259	1286	699	1597
	2325	2556	1041	762	1400	361
CHCHD4	35	121	21	82	66	92
	126	157	78	49	64	11
CHCHD5	6	6	4	1	7	12
	6	26	7	1	6	0
CHCHD6	13	17	13	14	15	15
	19	27	18	8	14	2
CHCHD7	54	260	43	163	92	209
	222	404	111	93	170	51
CHD1	148	639	167	482	306	430
	625	750	283	222	443	139
CHD1L	71	221	88	151	123	227
	282	407	152	86	230	56
CHD2	417	2321	564	1815	1052	1832
	1496	1633	916	690	1060	368
CHD3	135	223	67	216	113	182
	130	181	90	50	90	32
CHD4	216	1089	225	748	581	773
	734	765	400	327	486	201
CHD5	56	1	30	0	14	0
	3	0	26	0	3	0
CHD6	222	945	274	683	454	705
	606	919	460	238	497	173
CHD7	190	631	166	404	295	482
	408	503	246	195	287	130

CHD8	201	762	229	559	440	611
	511	710	330	215	393	168
CHD9	433	2209	541	1658	1099	1489
	1675	2047	940	711	1029	460
CHDH	11	46	18	33	34	27
	29	44	29	11	23	12
CHEK1	35	4	21	6	21	7
	5	4	33	4	15	1
CHEK2	36	12	13	9	6	8
	5	24	20	3	19	1
CHERP	25	76	12	52	31	42
	56	41	36	30	26	13
CHFR	22	57	20	23	16	27
	17	36	32	16	27	15
CHGA	11	0	4	1	3	0
	0	0	3	0	0	0
CHGB	21	1	10	1	4	1
	1	3	8	0	4	1
CHI3L1	14	6	10	0	3	0
	6	0	7	1	4	0
CHI3L2	22	1	7	0	3	0
	0	1	13	1	6	1
CHIA	17	0	15	0	9	0
	0	0	15	0	3	0
CHIC1	52	187	40	232	64	242
	210	122	112	45	126	25
CHIC2	23	59	17	75	40	32
	40	99	57	19	63	13
CHID1	22	69	35	31	32	43
	61	70	44	16	40	19
CHIT1	6	0	7	0	4	0
	0	0	6	0	2	0
CHKA	36	115	23	63	32	64
	77	162	61	43	56	17
CHKB	27	53	25	50	26	47
	35	116	45	31	28	14
CHL1	53	24	45	47	36	7
	12	41	33	3	32	13
CHL1-AS2	4	0	2	0	1	0
	0	0	2	0	0	0
CHM	100	342	108	331	219	289
	322	432	231	143	282	64
CHML	0	2	0	0	0	3
	1	1	0	2	2	1
CHMP1A	25	78	12	56	29	45
	60	96	43	23	46	17
CHMP1B	0	4	0	10	1	1
	3	4	2	3	4	3
CHMP2A	119	645	128	468	243	624
	673	1071	362	173	329	103
CHMP2B	102	464	73	441	233	411
	448	511	289	162	367	101
CHMP3	38	779	35	336	158	729
	741	919	95	198	223	119
CHMP4A	21	96	23	46	42	59
	59	89	42	31	48	20
CHMP4B	42	315	50	212	105	189
	230	215	93	57	107	43

CHMP4C	15	1	9	1	4	0
	0	3	5	1	3	0
CHMP5	68	301	76	211	126	220
	267	431	170	110	185	49
CHMP6	17	77	13	44	18	32
	44	71	28	25	38	13
CHMP7	71	305	85	204	166	204
	254	289	124	74	138	36
CHN1	26	33	26	31	25	19
	13	8	31	8	18	6
CHN2	26	16	24	28	14	10
	32	25	30	7	20	2
CHODL	11	7	10	3	7	6
	13	20	24	3	13	0
CHORDC1	93	403	95	292	221	251
	228	342	150	74	203	61
CHP1	115	641	119	441	222	458
	636	721	339	156	341	94
CHP2	23	4	16	3	7	4
	2	4	12	3	14	0
CHPF	18	16	6	7	10	10
	12	17	12	5	9	5
CHPF2	32	63	18	58	34	80
	50	91	63	24	43	11
CHPT1	53	242	28	142	84	142
	223	282	153	47	119	35
CHRAC1	31	119	24	128	84	153
	203	87	79	56	79	14
CHRD	26	24	14	17	3	21
	12	19	19	5	12	1
CHRDL1	64	162	40	209	97	129
	78	163	39	27	83	53
CHRDL2	11	12	4	4	4	9
	21	18	13	6	15	5
CHRFAM7A	14	5	2	3	3	0
	0	1	6	0	9	1
CHRM1	5	0	4	0	0	0
	0	0	3	0	0	0
CHRM2	20	0	7	0	4	1
	0	0	9	0	7	0
CHRM3	20	0	4	1	3	0
	0	0	6	1	5	0
CHRM4	4	0	1	0	0	0
	0	0	3	0	0	0
CHRM5	14	0	8	1	3	1
	0	5	15	0	5	0
CHRNA1	79	477	260	326	150	274
	185	194	74	92	65	35
CHRNA10	18	9	8	20	13	19
	12	24	11	2	13	2
CHRNA2	24	0	5	0	1	0
	0	0	8	0	1	0
CHRNA3	23	0	14	0	7	0
	4	1	7	0	4	0
CHRNA4	16	0	13	0	0	0
	0	0	6	0	5	0
CHRNA5	27	7	12	40	17	16
	33	5	24	8	26	1

CHRNA6	11	0	7	0	3	0
	0	0	4	0	4	0
CHRNA7	28	4	14	7	5	14
	8	3	17	4	11	0
CHRNA9	8	0	4	0	0	1
	0	0	7	0	3	0
CHRNB1	71	427	78	300	178	328
	379	264	146	109	220	53
CHRNB2	24	0	20	0	5	0
	0	1	8	1	8	0
CHRNB3	7	0	6	0	2	0
	0	0	5	0	0	0
CHRNB4	11	1	9	0	1	0
	0	0	5	0	0	0
CHRND	88	133	92	161	44	354
	127	57	89	38	45	11
CHRNE	20	6	22	24	2	2
	16	11	4	16	10	2
CHRNG	78	80	120	58	35	108
	106	28	28	48	47	7
CHST1	13	3	3	8	4	2
	13	6	9	3	5	1
CHST10	14	43	11	52	31	51
	38	41	37	13	38	7
CHST11	33	20	28	10	13	15
	15	17	19	8	8	5
CHST12	7	13	4	11	9	15
	20	19	7	3	11	9
CHST13	1	0	3	0	0	0
	0	0	2	0	0	0
CHST14	6	15	7	14	10	9
	14	14	4	3	7	2
CHST15	60	209	44	188	63	196
	106	156	79	30	115	28
CHST2	9	10	7	4	5	5
	9	10	19	6	9	4
CHST3	48	107	31	83	73	139
	94	224	67	42	70	43
CHST4	9	0	3	0	1	0
	0	0	3	0	2	0
CHST5	7	1	4	1	2	3
	0	0	5	1	4	0
CHST6	36	3	11	4	3	1
	0	0	13	0	9	0
CHST7	4	12	3	9	8	7
	8	16	4	6	16	9
CHST8	4	0	4	0	2	0
	0	0	3	0	2	0
CHST9	21	11	15	2	17	6
	4	1	13	2	7	4
CHSY1	33	96	35	87	48	74
	60	95	49	32	49	17
CHSY3	26	29	17	28	33	41
	45	19	29	19	32	1
CHTF18	9	15	6	14	8	9
	21	24	13	9	11	2
CHTF8	25	80	22	61	46	59
	55	66	31	18	45	11

CHTOP	107	555	77	298	254	298	386	610	161	197	297	41
CHUK	95	370	79	206	174	334	292	436	187	111	216	61
CHURC1	27	266	22	216	95	110	377	571	104	105	110	34
CHURC1-FNTB	102	323	72	173	168	207	296	468	147	77	168	44
CIAO1	109	494	92	359	204	337	379	505	229	153	278	92
CIAPIN1	32	151	26	122	51	104	193	213	103	42	103	24
CIB1	6	26	4	29	16	10	26	32	17	8	14	7
CIB2	7	33	9	22	14	12	20	23	7	1	9	2
CIB3	7	0	2	0	0	0	0	0	3	0	0	0
CIB4	5	0	4	0	0	0	0	0	2	0	3	0
CIC	35	141	34	62	47	78	99	75	42	48	76	28
CIDEA	6	0	5	0	4	0	2	2	0	0	1	5
CIDEB	20	19	10	13	17	21	17	18	14	6	17	3
CIDEC	12	11	9	2	12	2	5	11	6	0	8	18
CIITA	29	71	16	22	23	34	46	39	33	29	46	15
CILP	114	601	211	714	350	502	222	636	174	123	261	158
CILP2	12	2	9	0	1	0	0	0	6	0	4	0
CINP	28	119	18	59	52	72	88	125	34	28	64	16
CIR1	55	179	37	108	73	92	208	189	89	94	99	38
CIRBP	180	938	166	617	323	608	1031	1278	513	329	749	197
CIRH1A	43	156	35	85	72	79	101	115	60	45	75	19
CISD1	147	663	114	548	273	443	806	938	403	207	495	109
CISD2	114	106	79	89	78	96	77	110	122	25	75	11
CISD3	30	48	14	38	21	36	65	39	33	20	30	11
CISH	14	10	7	11	11	10	41	254	26	9	15	4
CIT	69	17	55	19	22	30	26	18	61	3	55	5
CITED1	2	1	1	0	0	2	0	0	3	0	1	0
CITED2	75	350	136	132	158	118	171	488	189	132	122	53

CITED4	5	32	3	25	14	30
	53	29	16	5	19	3
CIZ1	96	462	90	215	157	273
	330	376	157	173	224	98
CKAP2	45	85	36	95	55	55
	25	71	44	31	49	16
CKAP2L	24	0	17	0	6	0
	0	0	11	0	10	0
CKAP4	27	127	24	85	38	68
	65	97	42	21	42	11
CKAP5	328	1537	361	1012	824	1121
	1113	1790	717	560	961	303
CKB	19	53	18	19	19	23
	78	56	35	23	41	29
CKLF	8	20	3	11	5	5
	6	34	18	5	13	4
CKLF-CMTM1	6	5	10	2	4	2
	7	3	3	1	3	1
CKM	22353	108243	16886	79317	49280	95839
	145768	174919	74664	47357	78532	24831
CKMT1A	14	0	18	0	2	1
	0	0	8	0	7	0
CKMT1B	31	0	14	0	2	0
	0	0	10	0	2	0
CKMT2	738	4484	334	2324	1626	1961
	4273	4710	1860	986	2448	484
CKS1B	10	11	4	16	2	11
	8	15	4	5	8	2
CKS2	5	7	9	9	2	6
	7	11	5	4	6	0
CLASP1	597	3031	881	2200	1409	2151
	2660	3228	1510	880	1875	512
CLASP2	553	2009	524	1777	1141	1624
	1654	2645	1174	775	1507	423
CLASRP	40	109	25	55	38	77
	67	136	40	33	70	31
CLC	9	0	2	0	1	0
	0	0	4	0	1	0
CLCA1	32	0	16	0	5	0
	0	0	14	0	9	0
CLCA2	38	0	19	1	6	4
	0	0	21	0	14	0
CLCA4	27	0	13	1	9	1
	0	0	12	0	10	0
CLCC1	100	569	138	366	328	408
	389	677	303	204	334	107
CLCF1	5	1	2	1	1	1
	2	0	0	1	0	1
CLCN1	178	703	198	440	363	492
	629	652	365	280	444	133
CLCN2	17	5	12	7	3	3
	9	4	6	3	9	2
CLCN3	273	1237	287	860	629	844
	962	1299	624	470	755	251
CLCN4	302	1343	178	1140	654	1036
	957	1275	501	309	670	229
CLCN5	129	296	87	220	170	302
	159	316	207	88	175	52

CLCN6	85	158	45	133	71	139
	122	209	95	48	105	31
CLCN7	28	66	28	52	35	54
	56	78	49	20	45	15
CLCNKA	11	3	17	6	1	0
	2	1	5	1	6	0
CLCNKB	11	0	7	0	0	0
	0	0	8	0	5	0
CLDN1	29	3	30	75	4	10
	19	9	14	6	20	8
CLDN10	15	2	13	2	4	1
	1	1	6	1	4	0
CLDN11	13	2	7	10	4	5
	4	21	9	0	6	1
CLDN12	126	381	99	326	223	300
	428	642	217	164	333	70
CLDN14	17	2	10	0	0	5
	0	2	11	0	5	1
CLDN15	3	2	2	5	2	3
	1	3	9	1	3	0
CLDN16	18	0	3	0	6	0
	0	0	10	0	4	0
CLDN17	2	0	2	0	3	0
	0	0	2	0	0	0
CLDN18	24	0	11	0	4	0
	0	2	10	0	8	0
CLDN19	10	0	2	2	1	0
	2	0	2	0	2	0
CLDN2	19	12	9	1	7	7
	4	2	3	13	2	7
CLDN20	11	2	1	3	2	1
	2	2	3	0	1	0
CLDN22	12	0	9	0	6	1
	0	1	5	0	3	0
CLDN23	3	7	4	1	0	1
	1	5	4	0	3	1
CLDN24	4	0	1	0	1	0
	0	0	0	0	0	0
CLDN25	2	0	0	0	0	0
	0	0	0	0	0	0
CLDN3	1	0	0	0	0	0
	0	0	0	0	0	0
CLDN4	4	2	2	2	1	1
	1	0	2	0	0	0
CLDN5	7	29	7	16	3	16
	21	22	14	4	9	4
CLDN6	4	0	1	0	3	0
	0	0	4	0	1	0
CLDN7	11	1	1	1	1	3
	1	1	4	0	1	0
CLDN8	2	0	2	0	4	0
	0	0	1	1	0	0
CLDN9	4	2	3	0	1	3
	0	2	0	0	3	1
CLDND1	90	322	61	267	168	235
	277	433	207	135	203	76
CLDND2	2	0	3	0	1	1
	0	6	1	3	2	0

CLEC10A	18	11	7	14	7	9
	11	42	14	7	6	2
CLEC11A	3	3	5	5	3	5
	6	3	12	1	6	0
CLEC12A	12	5	14	0	6	2
	3	5	12	4	10	0
CLEC12B	18	1	11	0	8	0
	1	1	8	0	2	0
CLEC14A	44	228	27	124	87	140
	204	262	135	50	119	38
CLEC16A	83	271	62	197	106	166
	204	281	107	65	138	43
CLEC17A	14	0	3	0	1	0
	0	0	7	0	1	0
CLEC18A	20	1	8	1	0	0
	0	5	9	0	0	0
CLEC18B	19	1	10	1	4	2
	0	1	9	1	3	0
CLEC18C	6	0	3	1	0	0
	0	1	0	0	3	0
CLEC19A	11	3	10	0	6	0
	0	0	5	0	5	0
CLEC1A	12	18	9	14	15	9
	15	32	35	8	14	7
CLEC1B	7	2	9	0	6	1
	0	0	11	0	5	1
CLEC2A	10	0	6	0	1	0
	0	0	4	0	0	0
CLEC2B	69	202	70	125	78	76
	204	339	233	72	179	43
CLEC2D	82	151	43	72	77	81
	73	165	101	44	106	19
CLEC2L	4	2	4	2	2	0
	5	1	3	1	4	0
CLEC3A	21	1	0	0	2	1
	0	0	6	0	1	0
CLEC3B	20	129	9	88	77	89
	95	178	59	36	85	37
CLEC4A	20	15	10	18	8	25
	8	21	18	3	14	2
CLEC4C	16	0	2	0	3	0
	0	0	5	1	0	0
CLEC4D	20	2	4	0	5	0
	0	0	8	0	6	0
CLEC4E	14	3	7	1	3	0
	1	1	3	0	5	1
CLEC4F	15	5	10	1	6	1
	5	0	8	5	1	4
CLEC4G	3	1	5	1	0	0
	0	2	3	0	1	0
CLEC4M	25	0	6	0	1	0
	0	0	10	0	2	0
CLEC5A	25	0	22	1	6	0
	0	3	9	1	7	0
CLEC6A	15	0	12	0	1	0
	0	0	6	0	5	0
CLEC7A	24	7	12	18	7	25
	16	31	22	6	13	1

CLEC9A	15	1	17	0	5	0
	1	1	14	0	6	0
CLECL1	18	4	12	0	3	0
	0	2	12	0	5	1
CLGN	29	12	26	5	17	13
	11	26	26	8	22	2
CLHC1	77	59	35	49	65	40
	80	99	68	16	55	15
CLIC1	20	139	26	151	59	114
	77	178	73	30	53	20
CLIC2	36	108	27	71	37	60
	54	122	37	38	48	21
CLIC3	2	0	1	0	0	0
	0	1	0	0	1	0
CLIC4	152	798	167	609	319	508
	570	781	412	210	458	232
CLIC5	707	4514	696	3117	1980	3253
	3430	3786	1824	1314	2094	677
CLIC6	18	11	8	6	8	2
	11	5	10	7	7	2
CLINT1	169	1255	179	659	410	752
	695	727	365	297	446	151
CLIP1	988	5120	743	3321	2999	4535
	3703	5628	1902	2250	2467	1048
CLIP2	20	37	16	20	13	18
	26	28	22	10	20	7
CLIP3	15	10	9	3	5	3
	5	3	10	3	8	1
CLIP4	261	778	173	523	581	779
	978	1535	588	413	663	256
CLK1	389	1261	417	1118	696	1343
	1745	2184	852	568	1196	271
CLK2	32	167	49	91	74	124
	128	132	51	56	108	27
CLK3	67	191	48	140	103	161
	171	197	77	67	125	36
CLK4	253	712	241	531	383	752
	766	1304	440	308	742	146
CLLU1	23	1	18	1	7	0
	3	0	9	0	9	0
CLLU1OS	12	0	5	2	1	0
	0	0	3	0	10	0
CLMN	125	289	90	225	118	191
	274	319	181	81	176	56
CLMP	36	45	18	56	33	30
	18	50	19	21	29	26
CLN3	16	22	12	28	10	21
	27	36	17	10	17	10
CLN5	33	99	18	87	41	62
	69	124	50	32	58	24
CLN6	17	43	12	25	29	31
	42	55	29	17	15	4
CLN8	44	137	43	113	74	104
	87	166	90	25	83	26
CLNK	25	4	14	0	3	1
	0	0	22	0	8	0
CLNS1A	116	627	97	423	233	366
	599	730	293	165	310	101

CLOCK	207	1096	279	914	465	811
	612	842	451	221	412	125
CLP1	16	40	16	41	26	35
	59	100	32	17	29	13
CLPB	37	98	46	92	70	96
	96	129	68	40	73	23
CLPP	47	173	29	151	59	196
	251	303	117	64	123	40
CLPS	5	0	3	0	0	0
	0	0	4	0	0	0
CLPSL1	3	0	4	0	0	0
	0	2	2	0	1	0
CLPSL2	0	0	1	0	0	0
	0	0	1	0	1	0
CLPTM1	61	258	41	166	74	190
	197	194	91	89	122	45
CLPTM1L	33	105	23	65	40	59
	66	156	69	21	55	26
CLPX	150	806	141	537	392	464
	719	876	285	220	499	130
CLRN1	17	0	13	0	6	0
	0	0	11	0	5	0
CLRN2	3	0	3	0	0	0
	0	0	0	0	1	0
CLRN3	1	0	1	0	0	0
	0	0	5	0	2	0
CLSPN	65	2	36	2	15	1
	4	8	39	2	14	0
CLSTN1	73	273	67	222	160	190
	252	337	119	95	142	73
CLSTN2	51	67	43	41	69	41
	45	59	31	17	19	20
CLSTN3	19	27	16	16	20	14
	13	27	20	8	9	2
CLTA	56	242	41	123	91	136
	201	291	110	63	98	32
CLTB	92	436	64	275	143	300
	544	711	316	145	262	92
CLTC	384	1958	413	1722	967	1311
	1240	1784	812	480	992	370
CLTCL1	293	1482	381	1255	581	1106
	787	1502	597	492	894	314
CLU	43	184	43	207	78	62
	79	173	71	40	88	122
CLUAP1	72	240	46	116	127	197
	155	273	110	59	145	44
CLUL1	13	0	13	0	6	0
	0	0	18	0	4	0
CLVS1	20	1	8	3	8	3
	0	5	8	0	6	2
CLVS2	14	0	8	3	5	4
	0	0	7	1	13	0
CLYBL	64	149	40	118	55	68
	157	129	86	34	92	24
CMA1	6	0	2	2	4	7
	0	8	9	2	8	1
CMAS	35	221	39	237	96	172
	230	183	72	46	98	22

CMBL	303	1907	313	1907	948	924
	1800	958	599	513	599	304
CMC1	23	50	7	27	31	54
	57	55	36	21	25	4
CMC2	25	135	12	72	38	79
	166	212	64	30	84	16
CMC4	24	105	18	88	39	110
	135	120	63	30	54	14
CMIP	21	43	19	17	9	18
	18	28	17	11	10	2
CMKLR1	38	50	40	51	31	37
	22	120	38	19	45	14
CMPK1	177	832	140	740	309	670
	652	892	348	186	462	133
CMPK2	18	32	15	41	27	27
	24	49	37	42	40	1
CMSS1	45	90	31	83	81	69
	124	181	92	50	107	21
CMTM1	0	0	0	2	0	0
	0	0	1	0	0	0
CMTM2	3	2	4	1	0	1
	0	0	2	0	2	0
CMTM3	11	16	8	14	6	16
	12	23	12	5	17	5
CMTM4	56	138	43	107	60	97
	84	128	82	38	72	27
CMTM5	2	0	6	0	0	0
	0	0	2	0	1	0
CMTM6	24	106	26	92	46	79
	70	155	65	31	55	26
CMTM7	6	3	12	4	5	3
	6	6	8	2	0	1
CMTM8	5	15	1	4	8	8
	12	11	7	2	7	2
CMYA5	19360	144339	25463	115066	66339	103262
	91116	120243	46428	46102	67663	25850
CNBD1	19	0	12	0	1	1
	1	0	11	0	6	0
CNBP	1539	7627	1193	6038	3929	5282
	7278	8996	3822	2301	4726	1374
CNDP1	12	1	7	0	7	0
	0	0	14	0	6	0
CNDP2	53	165	46	82	63	106
	152	155	104	36	91	24
CNEP1R1	32	132	33	91	76	125
	145	170	55	44	96	25
CNFN	2	0	0	0	1	0
	0	0	0	0	0	0
CNGA1	53	65	22	51	33	26
	33	26	27	11	19	5
CNGA2	17	0	7	0	4	0
	0	0	2	0	0	0
CNGA3	18	0	10	1	5	1
	2	1	9	0	6	0
CNGA4	11	1	5	1	2	1
	0	0	4	0	4	0
CNGB1	54	0	18	0	8	0
	0	0	26	0	10	1

CNGB3	38	0	24	1	8	4
	0	0	21	0	14	0
CNIH	41	237	54	212	101	200
	225	284	124	55	153	36
CNIH2	1	0	1	0	0	0
	0	1	0	1	0	0
CNIH3	16	4	15	12	10	7
	6	9	7	2	8	0
CNIH4	20	91	24	84	60	80
	97	129	47	32	47	22
CNKSR1	55	148	57	113	96	166
	158	146	64	70	121	36
CNKSR2	76	19	25	24	24	7
	29	44	30	8	27	12
CNKSR3	25	38	17	19	24	25
	27	39	33	16	17	9
CNN1	11	26	1	15	4	2
	6	32	5	3	8	44
CNN2	10	17	8	13	12	7
	14	23	6	6	4	4
CNN3	42	274	50	230	133	185
	173	286	138	76	123	69
CNNM1	33	0	14	0	8	2
	0	2	17	0	10	0
CNNM2	22	52	25	48	15	40
	27	45	28	17	36	10
CNNM3	37	102	32	65	58	97
	61	128	57	35	66	23
CNNM4	69	330	55	213	110	192
	238	263	122	73	168	56
CNOT1	347	1897	405	1554	909	1276
	1302	1826	765	562	912	346
CNOT10	33	128	41	101	56	77
	82	149	75	47	81	20
CNOT2	124	599	158	396	310	463
	482	754	304	213	398	109
CNOT3	17	107	21	37	28	53
	85	83	31	31	43	18
CNOT4	139	652	160	565	390	495
	650	696	307	203	389	108
CNOT6	55	262	43	136	75	138
	177	219	109	39	104	32
CNOT6L	178	573	169	347	300	343
	561	752	303	194	380	123
CNOT7	297	1360	283	1304	800	1257
	1357	1547	588	418	837	266
CNOT8	57	223	39	175	105	154
	173	292	131	75	144	58
CNP	38	91	36	86	50	54
	64	94	37	31	55	19
CNPPD1	31	157	43	105	60	101
	149	248	75	39	101	23
CNPY1	23	1	13	6	5	8
	0	3	8	1	8	0
CNPY2	33	139	25	98	41	73
	113	175	66	25	62	17
CNPY3	17	28	10	24	22	24
	36	41	27	14	12	4

CNPY4	13	43	19	23	12	39
	20	33	23	8	13	7
CNR1	33	17	27	22	15	14
	17	28	31	9	21	9
CNR2	4	1	5	0	0	1
	0	0	2	0	1	0
CNRIP1	18	54	26	71	19	63
	35	43	43	13	32	9
CNST	193	616	143	500	473	478
	548	1103	290	254	466	162
CNTD1	19	19	17	17	12	15
	8	16	12	8	11	4
CNTD2	8	0	1	0	1	0
	0	0	4	0	2	0
CNTF	9	17	14	10	20	14
	13	12	12	5	15	12
CNTFR	21	74	30	93	55	80
	137	118	41	16	45	29
CNTLN	98	247	66	158	119	228
	187	297	154	75	173	46
CNTN1	65	73	59	76	36	24
	52	30	67	26	49	16
CNTN2	31	1	22	0	4	0
	3	0	17	0	10	0
CNTN3	44	7	33	7	10	6
	2	4	34	0	19	2
CNTN4	37	42	18	58	25	31
	20	30	45	1	31	12
CNTN5	54	1	52	9	20	1
	4	0	46	1	17	0
CNTN6	33	2	23	0	18	0
	0	1	21	0	16	0
CNTNAP1	49	38	38	43	24	46
	37	42	38	6	33	8
CNTNAP2	66	1	34	1	20	0
	3	1	39	0	9	0
CNTNAP3	53	20	27	23	18	13
	16	29	32	3	19	9
CNTNAP3B	97	142	55	73	92	25
	73	57	55	40	56	33
CNTNAP4	43	1	25	1	11	2
	2	3	23	0	20	0
CNTNAP5	47	0	39	0	14	0
	0	0	25	0	14	0
CNTRL	78	193	84	152	121	133
	126	214	113	71	138	46
CNTROB	57	65	36	57	36	56
	61	74	58	29	47	22
COA1	35	121	32	90	64	88
	101	198	70	45	99	23
COA3	23	128	12	95	41	83
	106	203	66	27	71	16
COA4	25	108	10	73	52	84
	107	186	71	28	61	20
COA5	61	245	41	189	104	172
	246	356	129	70	153	25
COA6	36	185	29	146	74	160
	257	401	117	80	129	37

COASY	23	61	18	44	29	34
	52	61	18	17	38	17
COBL	54	286	70	155	125	189
	284	156	104	73	134	21
COBLL1	53	239	90	149	82	106
	127	144	106	70	103	57
COBRA1	22	107	24	73	54	79
	84	121	52	36	56	14
COCH	15	9	8	12	12	3
	3	18	9	9	9	1
COG1	60	252	49	145	107	133
	138	246	86	59	131	33
COG2	89	293	76	225	182	264
	242	399	172	96	237	64
COG3	95	310	102	232	168	226
	281	403	230	108	253	65
COG4	113	455	97	299	275	318
	326	495	221	142	296	96
COG5	185	679	207	508	368	515
	502	855	338	208	428	121
COG6	101	309	111	358	153	303
	358	389	243	78	181	64
COG7	38	69	22	59	42	74
	70	89	61	24	43	21
COG8	49	145	44	121	61	132
	115	188	96	61	88	30
COIL	63	179	38	125	94	131
	126	218	85	68	111	23
COL10A1	6	8	7	6	3	5
	0	4	6	0	4	0
COL11A1	78	6	54	22	19	4
	5	3	45	0	25	3
COL11A2	51	12	58	15	28	17
	24	27	30	8	25	2
COL12A1	246	517	272	944	509	714
	333	787	341	153	478	180
COL13A1	33	34	33	15	21	28
	17	34	24	13	10	1
COL14A1	82	209	84	273	95	182
	67	424	137	29	119	120
COL15A1	317	1805	279	1205	660	877
	1184	1268	976	438	723	202
COL16A1	70	68	38	63	47	64
	39	80	38	19	50	29
COL17A1	66	0	44	1	12	2
	0	0	22	0	28	0
COL18A1	49	176	35	98	59	101
	46	117	57	36	67	21
COL19A1	86	329	443	150	32	144
	57	37	48	10	49	17
COL1A1	51	182	68	257	39	78
	91	69	83	20	32	30
COL1A2	296	1359	373	1744	557	1007
	669	976	647	234	522	295
COL20A1	29	0	11	3	1	1
	2	0	7	2	3	0
COL21A1	35	71	86	59	24	17
	53	143	47	15	57	17

COL22A1	71	1	55	6	19	5
	2	12	43	5	18	0
COL23A1	17	5	7	6	4	1
	1	4	8	1	5	0
COL24A1	85	65	62	33	104	28
	70	37	65	32	39	21
COL25A1	64	10	60	7	27	16
	9	2	47	3	26	2
COL27A1	43	89	50	44	40	66
	41	82	51	25	46	12
COL28A1	60	125	77	160	104	49
	233	311	192	104	235	78
COL2A1	45	0	27	0	12	0
	0	1	20	1	8	0
COL3A1	292	1733	496	1639	798	1004
	834	994	845	274	538	221
COL4A1	183	789	140	491	433	425
	648	936	438	259	414	210
COL4A2	160	835	155	520	367	390
	591	777	479	251	367	183
COL4A3	309	892	369	947	596	1119
	1076	883	1060	423	743	304
COL4A3BP	113	557	106	407	222	284
	338	500	210	128	268	107
COL4A4	196	477	190	399	324	482
	517	570	429	161	292	124
COL4A5	136	244	97	231	131	175
	190	291	168	87	172	42
COL4A6	82	39	42	27	25	37
	31	57	30	10	28	8
COL5A1	112	277	73	211	130	157
	129	151	101	32	114	51
COL5A2	131	373	154	358	177	284
	220	364	281	88	226	81
COL5A3	64	147	71	86	58	58
	89	156	66	35	79	33
COL6A1	138	567	133	539	270	443
	266	445	268	136	225	106
COL6A2	222	1036	198	707	393	833
	442	788	424	251	389	183
COL6A3	466	2318	483	2204	1125	1695
	953	2056	1178	569	946	449
COL6A5	87	0	52	1	32	1
	0	0	39	0	33	0
COL6A6	84	49	74	72	46	11
	48	35	61	4	41	11
COL7A1	76	87	66	43	52	54
	62	140	50	25	66	22
COL8A1	27	38	14	30	40	60
	30	42	26	10	19	10
COL8A2	11	2	9	5	6	5
	0	3	7	1	7	7
COL9A1	32	0	38	2	8	0
	3	0	32	0	13	1
COL9A2	11	4	12	0	1	0
	2	0	8	0	3	1
COL9A3	10	0	8	1	2	0
	4	0	6	3	1	0

COLEC10	14	0	7	0	2	0
	0	1	4	0	1	0
COLEC11	8	3	9	0	1	6
	0	8	6	1	6	0
COLEC12	37	131	17	67	63	74
	124	208	87	50	79	44
COLQ	56	113	28	74	50	81
	104	255	73	21	87	32
COMMD1	46	193	32	146	69	139
	205	276	112	43	109	27
COMMD10	31	125	35	107	76	108
	108	181	112	33	120	29
COMMD2	53	204	56	144	113	150
	150	260	131	62	126	25
COMMD3	104	383	77	266	150	275
	330	668	184	105	246	92
COMMD3-BMI1		79	481	119	292	246
	398	331	578	242	174	257
	72					
COMMD4	6	40	13	25	16	33
	37	102	37	16	36	3
COMMD5	13	44	8	21	17	23
	32	52	25	7	26	11
COMMD6	43	148	29	130	63	127
	151	299	100	43	93	27
COMMD7	38	144	26	74	43	95
	100	94	60	31	72	14
COMMD8	19	107	19	58	25	54
	69	85	39	30	64	11
COMMD9	67	159	54	165	90	137
	178	236	135	74	123	37
COMP	11	0	7	3	2	0
	0	0	3	0	1	8
COMT	61	203	27	113	80	119
	134	261	95	82	123	54
COMTD1	8	35	3	27	6	10
	33	73	40	6	19	6
COPA	242	906	177	622	488	634
	665	1069	502	314	535	207
COPB1	134	512	117	423	277	414
	401	607	286	157	342	96
COPB2	101	642	156	535	366	493
	441	620	356	200	383	103
COPE	22	108	26	96	57	81
	91	161	73	31	59	26
COPG1	92	478	78	278	190	247
	274	359	198	113	207	97
COPG2	39	143	54	89	63	99
	93	129	81	36	94	10
COPS2	197	1072	207	847	500	839
	849	1268	459	322	670	221
COPS3	198	954	192	887	440	788
	958	1401	568	310	636	195
COPS4	191	986	192	703	425	766
	787	1146	497	335	585	171
COPS5	125	857	129	606	372	581
	568	851	388	213	422	131

COPS6	48	237	33	187	102	128
	196	190	87	51	107	29
COPS7A	87	413	82	254	168	260
	459	451	193	103	203	62
COPS7B	25	118	22	66	41	66
	83	103	48	33	55	12
COPS8	123	608	112	459	349	453
	581	747	287	168	409	108
COPZ1	112	481	92	369	208	376
	378	589	241	116	269	73
COPZ2	61	155	28	95	55	83
	291	223	76	32	81	26
COQ10A	189	1088	122	940	406	868
	1672	1643	643	332	911	148
COQ10B	40	151	39	117	64	87
	109	208	64	41	122	36
COQ2	53	273	37	247	106	200
	356	221	144	75	171	49
COQ3	41	328	40	199	84	125
	266	342	143	62	169	41
COQ4	16	43	13	37	18	31
	44	50	31	14	42	2
COQ5	102	536	82	255	220	407
	496	704	280	209	314	67
COQ6	44	171	23	149	84	136
	194	265	104	60	124	38
COQ7	98	427	73	244	183	250
	380	468	158	117	304	59
COQ9	208	1541	140	674	449	816
	1181	1378	553	321	721	176
CORIN	34	2	18	0	12	0
	2	3	31	1	13	0
CORO1A	10	6	13	9	5	8
	7	15	9	1	8	1
CORO1B	10	25	6	12	9	21
	11	38	17	14	13	5
CORO1C	76	442	101	321	172	325
	218	278	116	100	153	80
CORO2A	34	7	16	1	5	1
	2	4	18	0	13	0
CORO2B	25	23	18	24	19	20
	27	36	27	7	14	4
CORO6	297	922	199	737	543	758
	809	1197	601	401	741	211
CORO7	2	16	6	10	2	15
	8	12	7	1	7	0
CORO7-PAM16		11	8	3	14	8
	12	12	21	20	1	16
	1					
CORT	1	0	0	0	0	0
	1	0	0	0	0	0
COTL1	18	21	7	17	15	13
	20	44	19	13	23	5
COX10	81	446	66	282	138	312
	499	501	169	132	314	72
COX11	128	725	148	655	314	542
	774	812	377	213	465	112

COX14	71	301	48	305	146	202
	324	623	204	69	200	49
COX15	159	551	130	425	275	373
	416	590	273	137	319	72
COX16	78	569	55	425	258	448
	596	932	285	124	343	71
COX17	196	1446	231	1070	714	1590
	1679	2420	708	315	856	114
COX18	42	110	37	84	56	71
	112	141	72	37	75	19
COX19	45	142	28	102	73	95
	106	136	80	46	91	32
COX20	80	479	77	287	142	322
	397	436	125	123	186	44
COX4I1	330	2240	284	1465	726	1150
	3267	2967	1094	425	1225	248
COX4I2	3	14	2	10	6	9
	12	25	7	9	7	1
COX5A	106	582	41	438	220	337
	845	765	341	118	327	67
COX5B	407	1608	181	1096	618	700
	2727	3015	846	574	1057	321
COX6A1	14	42	11	30	16	26
	39	76	26	15	23	5
COX6A2	412	1962	240	1302	903	1560
	2044	2839	1150	603	1625	328
COX6B1	284	1435	156	959	489	696
	1539	2349	692	357	857	211
COX6B2	5	2	0	0	0	1
	0	0	3	0	2	0
COX6C	675	3260	542	3409	1115	2196
	5761	6101	2307	807	2148	440
COX7A1	186	768	79	682	289	513
	813	1429	504	250	572	111
COX7A2	74	342	39	291	130	220
	504	760	270	109	314	65
COX7A2L	49	293	57	261	120	186
	382	473	188	87	189	53
COX7B	315	1591	162	1214	617	871
	1986	2725	731	437	1067	207
COX7B2	4	0	1	0	1	0
	0	0	1	0	5	0
COX7C	613	3005	457	3378	1184	2462
	5200	6201	2171	933	2418	453
COX8A	52	169	24	170	74	159
	242	360	137	63	127	32
CP	49	20	67	33	37	54
	21	39	31	17	29	13
CPA1	8	3	4	0	3	1
	0	1	1	0	3	2
CPA2	11	0	7	0	6	0
	0	0	10	0	7	0
CPA3	15	7	12	31	31	44
	21	69	24	13	22	14
CPA4	19	0	6	4	4	3
	0	0	8	0	4	0
CPA5	13	0	8	1	5	0
	0	0	10	0	2	0

CPA6	17	1	6	3	0	0
	3	2	11	0	7	0
CPAMD8	36	25	15	12	14	24
	25	27	27	7	35	4
CPB1	17	0	7	2	9	1
	0	3	6	1	7	1
CPB2	22	0	8	1	4	0
	0	0	12	0	4	0
CPD	172	626	142	414	347	413
	496	696	317	213	362	180
CPE	65	543	116	342	144	209
	252	336	190	81	319	88
CPEB1	16	13	11	20	7	29
	9	7	14	3	9	2
CPEB2	92	411	92	396	220	303
	274	323	168	82	145	68
CPEB3	304	1235	238	1040	585	780
	1227	1416	595	324	810	219
CPEB4	426	2202	462	1988	1076	1975
	2554	2157	940	777	1206	435
CPED1	599	2014	606	1307	1079	1488
	2135	3443	1449	833	1639	546
CPLX1	2	0	4	2	5	2
	1	2	3	0	1	2
CPLX2	12	0	9	0	2	0
	0	0	9	0	4	0
CPLX3	10	1	4	0	1	4
	0	2	5	0	0	0
CPLX4	13	0	6	0	2	0
	0	0	2	0	4	0
CPM	60	41	34	31	52	28
	85	89	52	24	45	35
CPN1	12	0	7	0	2	2
	0	0	11	0	3	0
CPN2	18	0	11	0	1	0
	0	0	15	1	6	0
CPNE1	83	297	67	165	155	189
	193	340	150	85	176	60
CPNE2	15	31	14	18	19	20
	14	47	22	10	14	7
CPNE3	117	637	147	488	253	359
	425	642	298	127	312	97
CPNE4	35	1	16	0	11	0
	0	1	18	0	9	0
CPNE5	16	1	10	1	4	1
	1	1	2	0	3	0
CPNE6	6	0	8	0	1	0
	0	0	11	0	2	0
CPNE7	9	0	8	0	0	0
	0	0	1	0	2	0
CPNE8	32	65	27	45	34	39
	62	54	64	18	43	14
CPNE9	12	0	7	0	5	0
	0	1	7	0	8	1
CPO	15	11	12	17	14	7
	12	15	7	2	12	4
CPOX	39	129	33	100	62	79
	113	162	61	39	84	21

CPPED1	44	91	32	86	37	92
	117	69	62	9	50	10
CPQ	40	132	33	102	94	105
	119	152	58	38	78	23
CPS1	71	25	45	45	26	18
	12	42	50	11	48	4
CPSF1	50	146	33	59	58	82
	93	133	53	40	57	13
CPSF2	192	1015	177	691	500	691
	796	920	397	291	598	171
CPSF3	46	189	54	98	92	132
	108	231	95	59	103	41
CPSF3L	68	238	51	155	120	199
	204	342	127	99	141	46
CPSF4	22	74	24	45	26	54
	61	75	42	28	43	16
CPSF4L	4	2	2	1	1	1
	0	1	2	0	2	1
CPSF6	178	874	246	706	431	755
	649	1142	480	307	656	183
CPSF7	98	463	110	240	172	289
	289	479	164	110	219	75
CPT1A	66	168	38	88	96	114
	91	147	69	52	51	21
CPT1B	231	862	167	646	410	598
	883	1500	482	235	596	156
CPT1C	23	0	13	2	7	3
	0	6	7	2	4	0
CPT2	49	192	36	143	84	141
	135	257	92	34	121	31
CPVL	28	38	47	64	39	70
	94	272	39	15	35	19
CPXCR1	3	0	0	0	4	0
	0	0	4	0	0	0
CPXM1	12	0	11	0	0	0
	10	0	8	0	2	0
CPXM2	33	56	18	96	40	37
	30	24	23	15	14	12
CPZ	12	0	6	0	1	0
	0	0	5	0	3	0
CR1	107	15	62	19	45	32
	14	40	71	7	35	4
CR1L	14	0	13	0	1	2
	0	0	12	0	2	0
CR2	52	1	22	1	16	0
	0	0	16	0	18	0
CRABP1	6	1	4	1	1	0
	0	0	3	0	0	0
CRABP2	3	29	4	8	5	21
	8	13	9	0	3	1
CRADD	21	78	17	67	20	69
	76	95	56	21	62	23
CRAMP1L	59	117	31	80	58	80
	119	151	67	27	79	34
CRAT	212	895	149	578	416	507
	672	704	247	232	401	145
CRB1	40	15	27	12	15	13
	15	21	46	6	21	2

CRB2	10	0	10	0	1	0
	0	0	7	0	2	0
CRB3	5	0	5	0	1	0
	1	1	0	0	0	0
CRBN	157	621	117	495	302	448
	458	821	367	219	430	154
CRCP	39	166	43	120	89	129
	81	164	58	41	97	32
CRCT1	0	0	1	0	0	0
	0	0	1	0	1	0
CREB1	122	465	123	319	278	340
	329	474	223	115	273	68
CREB3	27	78	23	68	35	86
	51	106	48	26	44	13
CREB3L1	11	8	11	12	9	6
	15	7	11	2	11	2
CREB3L2	84	294	60	282	140	185
	194	229	145	71	157	48
CREB3L3	27	0	3	0	1	1
	0	0	13	0	4	0
CREB3L4	18	25	12	27	19	22
	28	38	32	11	23	4
CREB5	97	166	45	157	108	142
	188	295	133	97	159	40
CREBBP	216	1110	267	757	403	647
	926	874	391	341	495	222
CREBL2	68	250	54	216	153	215
	248	354	136	99	185	58
CREBRF	148	484	118	351	257	335
	467	829	304	187	372	126
CREBZF	194	714	177	679	342	649
	642	1062	364	310	520	161
CREG1	107	391	100	288	186	443
	398	650	253	138	321	104
CREG2	21	0	10	0	1	0
	0	0	6	0	3	0
CRELD1	52	172	40	91	73	99
	117	197	84	51	74	36
CRELD2	19	48	17	47	20	32
	42	68	36	12	36	6
CREM	69	154	49	125	85	124
	143	257	119	54	107	32
CRH	1	1	2	0	1	0
	0	0	3	0	2	0
CRHBP	13	12	16	5	3	5
	4	4	13	0	7	0
CRHR1	20	13	11	12	12	10
	6	17	12	3	8	2
CRHR2	18	6	20	1	2	7
	11	9	7	2	10	1
CRIM1	249	683	250	443	456	372
	622	1323	430	322	809	337
CRIP1	2	12	5	9	6	8
	6	16	2	0	2	2
CRIP2	10	47	9	25	14	20
	15	39	21	10	16	10
CRIP3	5	2	2	0	1	0
	1	2	2	1	4	0

CRIPAK	19	70	25	58	39	39
	36	48	35	17	22	16
CRIPT	29	99	24	118	66	88
	114	206	103	44	85	26
CRISP1	16	0	6	0	1	0
	0	0	10	0	2	0
CRISP2	21	0	16	0	5	0
	0	0	12	0	6	0
CRISP3	10	0	13	0	7	0
	0	0	11	0	6	0
CRISPLD1	25	12	21	25	16	8
	11	9	31	2	17	3
CRISPLD2	52	161	23	109	63	96
	105	191	87	66	84	39
CRK	74	290	65	197	155	219
	321	382	128	114	184	83
CRKL	227	1135	238	914	579	827
	816	1218	562	309	644	208
CRLF1	13	23	3	18	7	19
	19	24	11	7	17	3
CRLF2 (NC_000023 1314868..1331529)				4	0	4
	0	1	0	0	0	1
	0	0	0			
CRLF2 (NC_000024 1264868..1281529)				4	0	2
	0	1	0	0	0	4
	0	0	0			
CRLF3	26	84	28	46	46	73
	68	84	49	25	47	19
CRLS1	73	365	54	274	188	229
	390	590	282	135	274	91
CRMP1	20	6	10	3	2	4
	0	0	12	2	2	0
CRNKL1	157	691	144	436	345	491
	516	860	298	217	434	158
CRNN	6	0	4	0	1	0
	0	0	5	0	1	0
CROCC	43	188	44	70	35	121
	82	149	77	37	61	10
CROT	75	194	65	148	102	133
	170	209	130	53	126	26
CRP	9	0	7	0	3	0
	0	0	11	0	3	0
CRTAC1	12	0	7	1	10	0
	3	1	5	1	7	0
CRTAM	30	1	10	0	2	0
	0	2	13	2	10	0
CRTAP	157	622	165	650	241	628
	420	621	321	252	351	129
CRTC1	34	46	18	33	12	27
	38	32	21	24	33	10
CRTC2	31	57	23	43	36	46
	48	67	34	24	49	17
CRTC3	67	169	41	138	90	152
	121	159	60	56	70	28
CRX	24	2	17	0	4	1
	0	2	12	1	4	0
CRY1	34	119	50	79	58	91
	126	151	67	40	97	19

CRY2	209	839	229	515	407	593
	683	859	329	293	555	167
CRYAA	0	0	0	0	2	0
	0	0	0	0	0	0
CRYAB	655	5114	563	1766	2136	2332
	4045	4233	1636	1106	2504	843
CRYBA1	11	1	3	1	5	1
	0	1	11	0	3	0
CRYBA2	3	0	2	0	0	1
	0	0	3	0	0	0
CRYBA4	8	0	6	0	3	0
	0	0	6	0	0	0
CRYBB1	10	2	2	0	1	0
	0	0	1	0	1	1
CRYBB2	8	1	2	0	3	0
	5	2	3	0	3	1
CRYBB3	7	9	6	3	2	2
	2	0	6	0	2	2
CRYBG3	316	1687	385	1239	874	1179
	1227	1950	953	532	1035	372
CRYGA	1	0	0	0	2	0
	0	0	1	0	0	0
CRYGB	4	0	4	0	0	0
	0	0	0	0	0	0
CRYGC	2	0	2	0	1	0
	0	0	2	0	2	0
CRYGD	0	0	1	0	0	0
	0	0	0	0	1	0
CRYGN	4	0	3	0	0	0
	0	0	2	0	0	0
CRYGS	3	10	3	7	9	6
	12	8	6	1	5	2
CRYL1	21	54	15	33	29	27
	48	70	22	11	35	10
CRYM	28	94	8	2	0	0
	0	3	8	2	89	49
CRYZ	80	228	74	164	143	269
	237	229	171	80	166	36
CRYZL1	39	137	24	100	63	95
	117	141	59	37	71	21
CS	362	2996	350	1933	1003	1668
	2615	2373	1017	668	1360	341
CSAD	40	76	27	50	49	38
	66	93	56	25	45	27
CSAG1	16	0	7	0	5	1
	0	0	4	0	1	0
CSAG2	8	0	0	0	3	0
	0	0	0	1	1	0
CSAG3	6	0	3	0	1	0
	0	0	3	0	4	0
CSDA	1269	10784	1227	5759	4318	8614
	15071	10446	5404	4555	5764	2295
CSDC2	3	4	5	3	2	1
	4	0	1	0	1	0
CSDE1	4882	25651	4732	20448	12139	20624
	19420	24936	11006	7875	14205	4222
CSE1L	171	1012	212	663	507	821
	720	1085	480	340	553	168

CSF1	28	54	29	66	35	48
	43	64	45	19	42	18
CSF1R	36	33	27	76	55	56
	44	121	52	25	64	21
CSF2	6	0	1	0	0	0
	0	0	2	0	0	0
CSF2RA (NC_000023 1387692..1428828)				12	7	5
	5	4	1	5	3	8
	1	4	0			
CSF2RA (NC_000024 1337692..1378828)				12	6	3
	1	2	6	5	7	5
	2	2	2			
CSF2RB	24	23	17	20	11	14
	8	22	14	8	12	3
CSF3	7	4	3	1	3	27
	8	6	7	0	6	1
CSF3R	9	21	7	7	2	9
	16	15	8	4	8	1
CSGALNACT1	50	103	27	64	42	39
	96	70	61	24	74	24
CSGALNACT2	74	259	57	240	102	172
	211	198	103	78	140	44
CSH1	6	0	1	0	0	0
	0	0	2	0	1	0
CSH2	2	0	0	0	0	0
	0	0	1	0	0	0
CSHL1	2	0	3	0	0	0
	0	0	0	0	1	0
CSK	17	57	16	50	21	48
	38	28	23	22	22	7
CSMD1	133	4	59	62	29	12
	20	3	67	12	49	4
CSMD2	97	3	57	5	25	5
	0	4	59	2	32	0
CSMD3	124	11	67	10	51	11
	3	19	78	3	56	0
CSN1S1	17	0	7	0	7	0
	0	0	9	0	5	0
CSN2	6	0	9	1	1	0
	0	0	8	0	0	0
CSN3	7	0	4	0	0	0
	0	0	4	0	1	0
CSNK1A1	353	1635	362	1282	826	1419
	1321	2009	780	576	1082	334
CSNK1A1L	10	0	2	0	1	0
	0	0	5	0	1	0
CSNK1D	33	168	37	99	85	115
	161	100	56	48	63	33
CSNK1E	49	151	32	119	60	108
	146	214	109	79	126	49
CSNK1G1	76	155	54	110	62	113
	92	151	93	47	99	29
CSNK1G2	14	112	21	65	45	79
	58	120	57	44	63	18
CSNK1G3	74	297	76	280	150	252
	328	375	222	84	198	54
CSNK2A1	223	962	189	638	437	647
	770	950	374	262	555	178

CSNK2A1P	0	0	0	1	1	0
	0	1	0	0	1	1
CSNK2A2	22	103	40	73	49	81
	143	105	42	38	50	17
CSNK2B	65	220	43	165	113	164
	225	241	107	52	138	35
CSPG4	53	108	49	90	43	79
	51	127	72	29	59	19
CSPG5	13	1	11	2	7	6
	1	0	7	0	5	0
CSPP1	87	373	116	207	175	291
	308	471	164	125	218	84
CSRNP1	14	27	10	35	36	28
	46	42	37	9	35	6
CSRNP2	58	182	58	119	72	139
	165	149	85	50	71	21
CSRNP3	72	37	52	65	34	23
	39	27	63	6	51	2
CSRP1	23	106	27	117	47	48
	71	123	39	38	62	55
CSRP2	11	55	6	32	14	26
	68	75	38	20	40	16
CSRP2BP	75	295	59	210	172	197
	211	325	161	110	166	51
CSRP3	1048	3935	708	1981	1766	3461
	5619	15678	5520	1867	4805	1126
CST1	3	0	0	0	1	0
	0	0	0	0	1	0
CST11	3	0	2	0	2	0
	0	0	2	0	4	0
CST2	2	0	3	2	0	0
	0	0	0	0	0	0
CST3	55	444	44	281	181	357
	370	462	181	121	144	73
CST4	2	0	4	0	0	0
	0	0	0	0	0	0
CST5	3	0	1	0	0	0
	0	0	2	0	0	0
CST6	1	1	0	1	1	0
	0	0	0	0	0	0
CST7	4	1	2	0	1	0
	2	2	2	0	5	1
CST8	9	0	5	0	1	0
	0	0	2	0	4	0
CST9	4	0	4	0	2	1
	0	0	6	0	2	0
CST9L	7	0	4	0	0	0
	0	0	3	0	1	0
CSTA	3	0	0	1	1	2
	0	0	2	0	3	0
CSTB	26	114	21	51	37	39
	110	91	29	22	53	8
CSTF1	34	175	37	108	69	109
	196	196	109	59	120	34
CSTF2	23	39	17	27	23	39
	40	32	29	8	18	3
CSTF2T	105	413	101	329	204	337
	364	428	159	112	267	44

CSTF3	57	183	51	103	108	160
	131	208	95	34	124	24
CSTL1	6	0	2	0	1	0
	0	0	4	0	0	0
CT45A1	36	0	1	0	7	0
	0	0	14	0	3	0
CT45A2	2	0	4	0	3	0
	0	1	0	0	0	0
CT45A3	0	0	0	0	2	0
	0	0	3	0	2	0
CT45A4	0	0	8	0	0	0
	0	0	0	0	0	0
CT45A5	26	0	5	0	4	0
	0	1	10	0	4	0
CT45A6	0	0	0	0	3	0
	0	0	0	0	1	0
CT47A10	0	0	0	0	0	0
	0	0	0	0	1	0
CT47A11	0	0	0	0	0	0
	0	0	0	0	1	0
CT47A2	0	0	0	0	1	0
	0	0	1	0	0	0
CT47A3	0	0	0	0	0	0
	0	0	1	0	0	0
CT47A5	0	0	1	0	0	0
	0	0	0	0	1	0
CT47A9	0	0	0	0	1	0
	0	0	0	0	0	0
CT47B1	8	0	1	0	3	0
	0	0	3	0	1	0
CT62	14	0	4	0	0	0
	0	0	8	0	1	0
CTAG1A	0	0	0	0	0	0
	0	0	1	0	1	0
CTAG2	0	0	1	0	0	0
	0	0	1	1	0	0
CTAGE1	25	1	22	0	3	0
	1	0	8	0	4	0
CTAGE15P	13	0	3	3	0	1
	0	0	7	0	6	0
CTAGE4	7	1	8	2	1	0
	6	1	10	0	2	0
CTAGE5	279	1612	231	1040	606	910
	1094	1435	713	369	920	159
CTAGE6P	8	2	11	1	1	0
	0	1	5	0	2	0
CTAGE9	8	0	6	2	1	2
	1	1	6	0	7	1
CTBP1	80	494	86	307	234	369
	340	514	230	147	271	78
CTBP2	128	197	69	97	123	151
	171	271	132	56	124	45
CTBS	29	59	21	74	38	43
	56	101	34	18	48	11
CTC1	59	106	60	83	61	74
	114	149	72	44	74	27
CTCF	76	493	77	367	252	297
	365	442	201	153	213	59

CTCFL	36	0	13	0	3	0
	0	1	13	0	7	0
CTDNEP1	92	946	78	437	246	365
	668	599	119	114	218	64
CTDP1	32	68	24	54	33	57
	52	70	30	16	36	10
CTDSP1	33	103	25	76	52	74
	96	142	62	29	70	14
CTDSP2	247	966	193	696	508	632
	908	986	516	306	600	209
CTDSPL	82	217	95	188	63	153
	156	174	113	47	95	20
CTDSPL2	71	310	95	278	145	225
	255	411	153	118	204	64
CTF1	18	27	15	15	19	24
	35	30	16	10	21	5
CTGF	17	91	17	156	40	69
	24	116	52	20	42	27
CTH	15	54	20	25	24	18
	47	101	35	31	59	12
CTHRC1	5	16	6	24	9	9
	12	18	12	1	6	2
CTIF	65	127	38	108	87	111
	114	129	78	47	79	38
CTLA4	9	0	3	0	3	2
	0	0	2	0	6	0
CTNNA1	176	873	214	667	460	605
	575	1001	433	266	444	182
CTNNA2	38	8	23	15	17	9
	3	4	22	0	15	0
CTNNA3	173	805	194	583	427	497
	715	683	472	260	557	130
CTNNAL1	113	341	63	392	252	236
	371	438	175	156	195	84
CTNNB1	270	1390	289	1154	651	912
	981	1641	724	405	867	255
CTNNBIP1	21	143	15	129	48	132
	204	126	101	30	86	25
CTNNBL1	47	118	37	81	84	96
	89	123	70	37	76	23
CTNND1	204	1074	197	673	473	646
	748	1067	549	315	574	190
CTNND2	40	3	26	6	9	2
	0	1	18	2	6	0
CTNS	34	38	24	28	24	23
	32	87	32	12	27	12
CTPS1	35	96	29	69	38	54
	64	82	48	29	40	16
CTPS2	69	174	43	122	106	147
	123	224	111	60	97	38
CTR9	110	347	99	309	217	261
	386	390	151	116	217	65
CTRB1	1	0	3	0	0	0
	0	0	1	0	0	0
CTRB2	1	0	0	0	0	0
	0	0	1	0	0	0
CTRC	2	0	2	0	0	0
	0	0	2	0	1	0

CTRL	4	4	4	3	4	4
	3	6	5	0	2	0
CTSA	44	176	44	129	100	114
	205	300	106	70	125	48
CTSB	131	801	121	588	439	695
	883	593	329	263	440	153
CTSC	110	178	104	167	153	204
	187	546	192	104	196	70
CTSD	79	270	65	221	142	279
	181	396	151	95	149	59
CTSE	15	0	9	0	4	0
	1	0	2	0	1	0
CTSF	96	254	35	233	155	241
	324	765	327	177	385	97
CTSG	5	4	8	12	11	19
	1	17	9	7	5	4
CTSH	35	127	14	61	33	85
	159	145	78	44	49	24
CTSK	23	67	15	104	28	68
	47	76	39	14	37	27
CTSL1	81	447	86	384	160	341
	493	673	288	140	318	78
CTSL2	23	3	14	2	10	2
	4	2	10	0	11	1
CTSO	51	152	45	122	90	110
	120	203	96	58	116	40
CTSS	43	134	26	71	43	117
	58	168	67	46	68	25
CTSW	4	8	1	1	2	3
	2	2	2	0	6	0
CTSZ	25	202	24	130	83	153
	129	185	86	42	68	35
CTTN	129	627	144	440	236	358
	415	511	249	178	300	110
CTTNBP2	54	14	26	23	22	21
	14	26	34	9	30	11
CTTNBP2NL	44	133	37	79	51	77
	91	135	73	36	77	30
CTU1	6	1	7	0	2	0
	1	3	5	0	2	2
CTU2	9	25	9	12	14	17
	20	30	18	6	13	2
CTXN1	1	0	0	0	0	0
	0	0	0	0	0	0
CTXN2	24	0	22	0	5	0
	0	0	5	0	8	0
CTXN3	26	325	32	613	100	387
	276	45	130	19	172	8
CUBN	139	71	86	39	68	64
	43	89	90	41	108	21
CUEDC1	31	94	30	92	72	121
	81	92	74	52	74	28
CUEDC2	54	277	38	192	110	178
	298	336	105	74	130	29
CUL1	211	1164	270	779	626	940
	874	1033	442	386	640	196
CUL2	172	864	159	612	391	654
	651	798	358	257	471	142

CUL3	359	2702	503	2125	1088	1732
	1882	1991	876	637	1232	387
CUL4A	467	2554	484	1646	1130	1676
	1804	2612	995	719	1358	416
CUL4B	100	349	110	304	170	246
	281	346	187	98	176	65
CUL5	499	2755	581	2638	1408	2084
	2372	3221	1290	887	1897	468
CUL7	56	211	67	136	73	114
	142	123	96	45	76	53
CUL9	90	176	64	77	80	126
	99	184	84	58	112	34
CUTA	29	155	27	153	76	155
	169	202	120	62	105	30
CUTC	266	1488	242	1288	674	1144
	1280	2458	1070	457	1158	226
CUX1	215	1050	223	668	491	715
	863	987	338	292	458	185
CUX2	24	11	17	2	2	1
	2	1	12	0	8	0
CUZD1	7	2	7	2	3	2
	0	1	5	2	8	2
CWC15	223	903	126	657	378	816
	932	1538	502	316	581	155
CWC22	73	341	69	203	176	285
	250	329	143	93	182	66
CWC25	40	104	25	71	45	96
	87	115	64	38	78	19
CWC27	36	229	34	96	88	127
	136	161	69	56	110	32
CWF19L1	34	115	33	79	74	67
	86	186	87	34	107	23
CWF19L2	71	374	99	253	207	255
	216	361	169	145	219	73
CWH43	9	0	10	0	8	0
	0	0	10	0	7	0
CX3CL1	15	35	8	20	15	23
	18	38	16	14	21	10
CX3CR1	33	54	29	64	16	50
	46	54	25	11	50	4
CXADR	26	4	15	2	10	0
	0	2	19	1	14	0
CXCL1	8	0	3	0	2	0
	1	1	3	1	0	0
CXCL10	16	13	4	6	6	4
	6	9	12	0	10	0
CXCL11	9	3	8	4	1	2
	1	2	10	0	5	1
CXCL12	177	933	143	742	414	585
	778	807	434	227	484	159
CXCL13	5	0	4	0	0	0
	0	0	7	0	4	0
CXCL14	73	295	84	310	61	115
	326	408	133	131	179	53
CXCL16	7	5	2	4	6	5
	9	4	6	3	1	2
CXCL17	3	0	3	0	0	0
	0	0	3	0	4	0

CXCL2	7	30	6	32	15	16
	13	10	7	14	7	0
CXCL3	2	0	4	2	3	2
	1	0	2	0	1	0
CXCL5	6	2	4	1	3	2
	0	0	1	0	5	1
CXCL6	4	0	3	1	1	1
	0	0	3	0	2	0
CXCL9	17	31	18	32	17	13
	13	31	20	8	30	1
CXCR1	7	5	6	1	2	6
	5	4	3	0	1	0
CXCR2	17	9	11	4	0	4
	7	5	9	2	5	2
CXCR3	7	2	0	0	2	0
	0	0	2	0	2	0
CXCR4	18	31	13	19	14	17
	11	15	13	4	10	2
CXCR5	10	3	8	0	2	0
	0	0	6	0	2	0
CXCR6	16	10	13	7	10	8
	7	27	19	3	17	2
CXCR7	36	98	11	127	82	132
	42	211	75	30	69	28
CXorf1	11	1	4	0	4	0
	0	0	3	0	2	0
CXorf21	17	6	4	3	3	3
	1	6	8	4	6	3
CXorf22	32	0	7	0	10	0
	0	0	10	0	8	0
CXorf23	121	398	90	263	164	196
	170	398	160	98	241	41
CXorf26	12	73	16	39	33	45
	54	52	24	20	32	13
CXorf27	1	0	2	0	1	0
	0	0	2	0	0	0
CXorf30	23	0	12	0	11	0
	0	2	7	0	6	0
CXorf36	88	253	25	124	97	108
	206	202	101	77	87	29
CXorf38	42	100	12	73	46	94
	63	74	50	33	40	14
CXorf40A	30	81	24	38	38	64
	62	113	42	26	40	17
CXorf40B	16	48	6	32	20	38
	48	55	21	20	27	4
CXorf48	29	0	4	0	3	1
	0	1	5	0	1	0
CXorf49	6	0	4	0	2	0
	0	0	1	0	0	0
CXorf49B	4	0	5	0	2	0
	0	0	2	0	0	0
CXorf51A	3	0	0	0	0	0
	0	0	1	0	0	0
CXorf51B	1	0	1	0	0	0
	0	0	0	0	0	0
CXorf56	43	93	40	96	88	81
	113	181	63	40	108	36

CXorf57	38	69	32	36	31	62
	32	88	44	26	47	13
CXorf58	8	2	1	0	2	1
	2	2	8	0	1	1
CXorf59	17	0	8	0	7	0
	0	0	10	0	4	0
CXorf61	1	0	0	1	2	5
	0	2	0	3	1	0
CXorf64	9	3	4	3	5	5
	2	2	3	0	1	0
CXorf65	11	1	5	0	1	0
	0	1	2	0	1	0
CXorf66	14	0	0	0	1	0
	0	0	2	0	3	0
CXorf68	10	3	1	4	5	1
	3	1	2	1	2	0
CXorf69	9	17	6	19	16	24
	13	18	9	6	14	7
CXXC1	25	66	20	37	31	49
	27	98	30	27	40	13
CXXC11	3	0	0	0	1	0
	0	0	0	0	0	0
CXXC4	27	25	18	33	16	11
	27	21	19	4	19	3
CXXC5	43	210	51	114	76	134
	90	81	63	26	49	26
CYB561	22	26	14	29	10	21
	23	33	19	10	23	6
CYB561D1	43	91	41	72	44	117
	51	34	29	26	33	11
CYB561D2	4	21	3	14	10	22
	15	32	13	7	11	5
CYB5A	25	95	17	86	30	67
	92	96	67	22	53	15
CYB5B	81	364	79	310	211	282
	359	522	191	105	223	52
CYB5D1	77	253	50	90	94	125
	207	252	121	72	116	38
CYB5D2	24	60	14	58	31	57
	76	77	35	22	44	10
CYB5R1	546	1998	273	1019	1045	985
	1603	3186	805	745	1124	464
CYB5R2	13	2	7	7	6	9
	4	6	7	0	1	2
CYB5R3	68	338	81	176	150	189
	284	252	101	92	133	86
CYB5R4	37	66	40	76	39	89
	85	99	59	32	65	16
CYB5RL	29	33	17	34	18	38
	31	31	17	17	23	4
CYBA	2	37	11	46	14	23
	18	71	18	14	13	9
CYBASC3	21	64	17	71	23	62
	28	47	39	19	24	13
CYBB	50	120	37	117	74	163
	55	256	95	36	95	36
CYBRD1	158	635	156	661	376	574
	394	1068	262	145	276	207

CYC1	267	1338	187	1019	506	800
	1638	2151	820	369	929	209
CYCS	820	5270	700	2964	1984	3485
	5400	7978	2611	1385	3433	663
CYFIP1	86	350	103	256	150	186
	267	329	187	87	171	76
CYFIP2	61	63	52	48	53	68
	66	77	55	13	38	9
CYGB	11	34	6	19	11	10
	29	34	18	11	11	6
CYHR1	45	141	31	67	62	85
	108	175	64	41	61	20
CYLC1	20	0	8	0	3	0
	0	0	5	0	2	0
CYLC2	18	0	8	0	10	0
	0	0	14	0	2	0
CYLD	225	982	296	842	459	783
	806	1114	479	278	662	188
CYorf17	0	0	5	0	1	0
	0	1	1	0	1	0
CYP11A1	13	7	8	7	3	5
	6	7	14	1	8	0
CYP11B1	13	0	11	0	1	0
	0	0	5	1	4	0
CYP11B2	6	0	10	0	0	0
	0	0	5	0	1	0
CYP17A1	9	1	7	2	0	0
	0	4	3	1	1	1
CYP19A1	42	2	23	0	10	1
	0	2	15	1	14	0
CYP1A1	16	1	5	1	3	0
	0	121	7	5	8	26
CYP1A2	15	0	3	0	2	0
	0	0	6	0	3	0
CYP1B1	20	57	28	106	34	22
	25	141	31	12	32	33
CYP20A1	117	275	109	172	189	208
	184	326	132	133	161	77
CYP21A2	9	3	5	3	2	2
	0	2	4	0	2	0
CYP24A1	21	0	12	0	1	0
	0	0	5	0	6	0
CYP26A1	8	0	6	0	1	0
	0	0	9	0	2	0
CYP26B1	18	7	32	18	11	11
	2	3	12	4	10	2
CYP26C1	5	1	0	2	0	0
	0	3	5	0	0	0
CYP27A1	43	145	18	180	75	249
	104	124	86	39	67	26
CYP27B1	22	1	7	0	6	0
	0	3	7	0	5	0
CYP27C1	27	13	12	4	6	6
	5	0	14	1	7	1
CYP2A13	16	0	6	0	2	0
	0	0	5	0	3	0
CYP2A6	18	0	9	0	4	2
	0	1	4	0	7	0

CYP2A7	14	0	15	1	9	1	0	1	10	0	10	0
CYP2B6	33	0	8	0	5	0	1	0	12	0	9	0
CYP2C18	25	0	18	0	2	1	0	0	11	0	4	0
CYP2C19	12	0	7	0	5	0	0	0	8	0	5	0
CYP2C8	30	2	19	4	7	1	1	20	17	2	10	0
CYP2C9	18	1	22	0	7	0	0	0	21	0	11	0
CYP2D6	13	0	7	3	0	0	1	2	2	0	2	1
CYP2E1	15	32	10	16	8	20	15	27	14	7	14	3
CYP2F1	18	0	7	0	1	0	0	0	10	0	5	0
CYP2J2	69	272	65	172	77	267	234	417	191	126	194	50
CYP2R1	14	47	30	56	37	50	68	76	43	29	50	11
CYP2S1	8	3	9	0	4	1	0	5	6	1	4	0
CYP2U1	41	115	32	88	53	113	58	112	47	47	62	15
CYP2W1	5	1	3	0	0	0	0	0	1	0	1	0
CYP39A1	23	30	16	8	12	13	4	52	21	5	13	9
CYP3A4	31	2	15	3	3	8	0	1	13	0	10	0
CYP3A43	16	9	14	13	10	4	4	11	8	4	8	4
CYP3A5	28	6	24	9	4	8	5	4	16	1	15	0
CYP3A7	0	2	0	0	1	0	0	0	1	1	0	1
CYP3A7-CYP3AP1	17	4	21	1	4	1	0	1	16	0	8	0
CYP46A1	7	9	13	2	10	2	4	6	10	1	9	1
CYP4A11	32	1	18	6	11	0	2	2	11	0	6	0
CYP4A22	7	0	9	1	1	1	0	1	5	0	3	1
CYP4B1	31	63	27	51	40	55	83	219	46	35	79	41
CYP4F11	21	2	3	0	2	0	2	0	9	0	7	0
CYP4F12	17	5	6	17	3	9	6	7	10	2	2	3
CYP4F2	10	0	7	1	2	0	0	0	6	0	4	0
CYP4F22	22	0	4	1	6	0	2	3	7	0	5	0

CYP4F3	22	0	6	2	7	2
	2	0	14	0	2	0
CYP4F8	12	0	6	0	2	0
	0	0	7	0	5	0
CYP4V2	86	669	47	452	139	335
	566	304	223	123	260	58
CYP4X1	39	137	59	109	51	77
	48	129	73	32	77	25
CYP4Z1	36	8	22	14	12	21
	6	12	15	4	20	3
CYP51A1	52	198	49	78	72	218
	198	262	157	23	108	27
CYP7A1	21	0	9	0	6	0
	0	0	16	0	5	0
CYP7B1	12	19	14	11	7	8
	9	12	18	2	10	3
CYP8B1	13	0	2	0	3	0
	0	0	6	0	4	0
CYR61	21	120	37	209	47	91
	38	36	24	15	20	13
CYS1	4	7	4	2	4	1
	3	0	3	1	6	2
CYSLTR1	10	7	2	4	6	9
	0	8	1	3	6	2
CYSLTR2	21	1	14	1	9	1
	4	2	6	2	2	1
CYSTM1	102	533	53	313	191	321
	419	713	263	113	265	71
CYTH1	106	326	81	193	205	247
	352	536	206	122	239	119
CYTH2	24	66	14	47	32	49
	48	79	35	16	44	13
CYTH3	73	182	37	137	119	132
	161	154	74	36	97	50
CYTH4	23	24	13	19	21	25
	19	52	27	3	18	12
CYTIP	24	21	13	17	10	4
	13	15	25	3	18	2
CYTL1	14	1	3	0	3	0
	0	1	2	0	0	0
CYYR1	57	187	39	129	57	90
	197	231	123	60	119	43
D2HGDH	20	83	9	48	38	40
	52	103	30	23	39	18
DAAM1	95	343	110	237	199	198
	209	334	174	123	146	67
DAAM2	85	223	54	145	90	245
	390	232	127	66	193	42
DAB1	44	1	22	2	12	4
	1	1	22	1	10	0
DAB2	95	299	83	320	207	248
	197	346	166	111	198	93
DAB2IP	87	406	70	244	168	289
	348	304	178	109	188	63
DACH1	36	98	19	79	40	49
	78	90	60	35	25	14
DACH2	24	1	9	0	3	0
	0	3	6	0	4	0

DACT1	26	40	28	45	15	23
	21	20	14	5	17	6
DACT2	6	3	1	3	3	3
	0	6	0	3	3	0
DACT3	11	1	4	5	1	0
	4	1	5	0	3	0
DAD1	45	263	21	177	94	117
	222	320	113	51	131	22
DAG1	252	1102	163	745	609	989
	1088	1305	528	412	632	240
DAGLA	29	32	27	19	20	25
	22	34	19	21	19	4
DAGLB	28	79	27	76	41	66
	67	72	55	20	51	12
DAK	22	47	17	17	13	28
	19	20	23	7	23	5
DALRD3	19	47	12	32	15	36
	44	46	40	13	24	3
DAND5	2	0	2	0	0	0
	0	0	0	0	0	0
DAO	12	0	4	1	0	0
	0	0	6	0	2	0
DAOA	5	0	11	0	5	0
	0	0	2	0	3	0
DAP	10	35	12	13	10	15
	19	28	22	3	12	6
DAP3	107	499	112	360	193	393
	487	610	253	183	323	82
DAPK1	49	69	47	58	42	58
	50	84	64	16	43	11
DAPK2	114	422	98	256	226	310
	302	402	207	152	226	66
DAPK3	34	94	18	55	52	75
	41	195	50	36	56	15
DAPL1	0	0	3	0	1	0
	0	0	3	0	0	0
DAPP1	39	42	22	17	14	21
	12	50	24	8	24	3
DARC	12	17	7	13	8	5
	9	20	8	4	13	4
DARS	198	941	196	612	447	634
	790	1089	476	297	616	201
DARS2	45	113	30	75	55	86
	93	159	61	30	70	24
DAXX	35	81	33	56	40	81
	73	113	42	34	66	20
DAZ1	0	1	10	0	0	0
	0	0	20	0	4	0
DAZ2	0	0	9	0	0	0
	0	0	15	0	6	0
DAZ3	0	0	1	0	0	0
	0	0	6	0	2	0
DAZ4	0	0	16	0	0	0
	0	0	15	0	7	0
DAZAP1	49	193	40	99	74	112
	158	255	57	55	86	29
DAZAP2	86	310	60	299	215	314
	304	491	240	115	240	74

DAZL	15	1	8	0	3	1
	0	0	18	0	9	1
DBC1	23	5	17	0	6	0
	3	0	6	1	7	0
DBF4	39	47	25	26	25	20
	33	55	26	16	42	13
DBF4B	24	16	18	1	4	14
	10	17	16	2	16	1
DBH	9	0	6	0	3	0
	0	0	6	0	2	0
DBI	135	543	90	505	381	550
	655	771	207	94	298	88
DBN1	29	112	37	121	61	104
	85	136	44	25	57	26
DBNDD1	20	127	24	99	43	159
	39	32	31	23	25	12
DBNDD2	49	308	27	256	170	273
	428	506	170	133	236	67
DBNL	36	122	35	88	51	82
	65	157	61	31	71	19
DBP	27	110	19	48	47	64
	95	123	67	25	48	17
DBR1	34	99	29	74	51	78
	113	175	65	48	63	23
DBT	147	603	116	307	275	319
	367	643	283	184	342	123
DBX1	2	0	3	0	0	0
	0	0	1	0	2	0
DBX2	18	5	9	1	1	1
	2	1	11	0	1	0
DCAF10	105	431	96	331	206	334
	334	532	190	127	243	75
DCAF11	186	770	165	557	347	539
	763	819	427	194	544	145
DCAF12	58	281	53	142	105	161
	181	171	80	51	106	42
DCAF12L1	7	0	3	0	1	0
	0	0	2	0	0	0
DCAF12L2	14	4	4	2	2	0
	4	3	6	0	4	0
DCAF13	46	187	50	175	76	144
	149	263	105	65	140	26
DCAF15	7	44	10	27	19	54
	32	58	33	21	43	8
DCAF16	84	320	107	301	182	299
	197	449	178	125	250	121
DCAF17	73	160	53	98	87	117
	156	218	90	51	116	39
DCAF4	39	73	26	49	31	57
	46	68	38	20	43	7
DCAF4L1	17	33	24	31	19	50
	6	48	32	6	36	9
DCAF4L2	24	0	9	0	7	0
	0	0	7	0	4	0
DCAF5	184	1011	178	750	429	685
	634	662	285	253	389	158
DCAF6	1442	9177	1709	6012	3865	5790
	8017	7647	3446	2864	4794	1583

DCAF7	77	331	81	248	148	243
	290	313	158	87	198	47
DCAF8	379	1992	382	1306	945	1481
	1526	2220	967	556	1427	382
DCAF8L1	14	0	6	0	3	0
	0	0	7	0	3	0
DCAF8L2	8	0	1	0	2	0
	0	0	0	0	0	0
DCAKD	18	76	23	49	33	42
	45	62	45	25	44	21
DCBLD1	24	84	20	45	56	88
	63	57	45	40	53	21
DCBLD2	116	373	105	219	208	258
	245	423	187	92	172	70
DCC	67	2	47	0	26	0
	0	0	50	0	26	0
DCD	3	0	6	0	2	0
	0	0	2	0	0	0
DCDC1	16	0	11	0	2	0
	0	0	10	1	3	0
DCDC2	28	4	10	4	6	6
	1	3	14	3	15	1
DCDC2B	22	2	2	0	2	2
	0	2	11	0	3	2
DCDC2C	6	1	4	2	2	0
	0	2	8	0	2	0
DCDC5	39	0	27	0	9	0
	0	2	21	0	10	1
DCHS1	58	133	26	104	64	75
	86	99	93	49	51	29
DCHS2	105	28	55	9	49	46
	9	14	50	14	33	2
DCK	48	179	57	213	82	178
	172	186	90	38	118	51
DCLK1	117	409	361	343	129	226
	103	331	128	40	115	61
DCLK2	25	32	21	21	18	23
	32	19	29	5	12	2
DCLK3	33	23	14	17	18	17
	21	24	29	8	23	4
DCLRE1A	42	70	33	43	53	53
	44	99	55	12	72	19
DCLRE1B	29	30	19	16	25	18
	20	25	17	5	23	6
DCLRE1C	38	59	17	37	27	40
	27	67	30	24	42	13
DCN	756	2578	955	4265	1763	4057
	2733	5391	1977	897	2263	718
DCP1A	93	337	67	246	206	289
	289	394	147	85	177	64
DCP1B	26	64	25	47	38	46
	28	66	25	17	45	21
DCP2	156	676	164	572	348	442
	405	718	353	157	347	118
DCPS	15	50	11	23	38	45
	27	77	27	29	32	10
DCST1	10	0	8	0	0	0
	0	0	6	0	3	0

DCST2	14	1	8	0	1	0
	1	0	3	1	5	0
DCSTAMP	11	0	5	0	2	0
	0	0	9	0	2	0
DCT	18	12	17	4	4	7
	4	8	16	4	10	1
DCTD	44	218	52	168	95	185
	258	213	113	81	113	34
DCTN1	188	880	189	527	427	627
	579	729	312	292	443	165
DCTN2	70	403	69	273	130	260
	276	350	160	87	150	54
DCTN3	39	193	32	126	93	162
	281	263	115	62	136	32
DCTN4	183	800	196	694	452	637
	626	881	466	270	532	155
DCTN5	138	301	106	273	179	277
	274	448	223	89	233	70
DCTN6	60	236	39	141	81	142
	185	297	124	68	136	36
DCTPP1	15	31	8	31	14	39
	51	67	35	18	40	8
DCUN1D1	146	772	187	801	463	608
	657	804	405	182	462	114
DCUN1D2	190	610	104	476	355	462
	454	786	264	184	446	95
DCUN1D3	24	70	23	54	49	64
	45	101	50	23	53	13
DCUN1D4	137	452	146	428	274	450
	478	591	225	153	303	92
DCUN1D5	160	558	158	543	254	462
	346	541	274	126	304	75
DCX	63	5	15	16	9	0
	5	3	11	5	6	0
DCXR	35	64	8	39	26	39
	87	125	53	22	48	13
DDA1	30	74	15	45	44	71
	79	100	37	31	40	16
DDAH1	192	972	218	371	383	286
	602	546	212	172	412	101
DDAH2	16	54	22	33	25	54
	25	47	26	16	24	8
DDB1	384	1310	351	1088	696	963
	991	1542	763	465	882	331
DDB2	23	57	19	53	31	17
	32	56	22	19	31	11
DDC	12	1	13	4	2	4
	2	6	10	1	6	2
DDHD1	144	253	101	223	129	258
	172	246	175	79	163	36
DDHD2	136	612	137	486	209	474
	511	694	328	216	378	124
DDI1	11	0	1	0	1	0
	0	2	7	0	4	0
DDI2	161	561	145	404	290	374
	398	627	328	192	343	112
DDIT3	21	72	14	66	39	49
	85	96	33	32	41	10

DDIT4	97	298	94	329	187	168
	281	595	187	99	198	114
DDIT4L	320	1686	612	2531	734	1886
	2555	1583	1454	277	950	187
DDN	50	353	23	227	89	206
	172	344	349	71	140	75
DDO	23	121	14	54	36	60
	67	97	57	28	41	19
DDOST	71	196	29	172	111	199
	156	348	160	79	148	44
DDR1	45	100	31	70	49	60
	103	176	77	40	82	16
DDR2	58	147	44	135	100	64
	70	136	77	31	71	46
DDRGK1	33	143	19	91	54	87
	104	172	64	46	61	28
DDT	19	80	13	64	21	66
	55	114	62	21	44	10
DDTL	8	7	7	2	5	2
	11	22	8	3	12	1
DDX1	285	1984	320	1458	819	1347
	1671	1671	668	579	1007	248
DDX10	72	227	64	118	140	173
	189	214	122	72	135	40
DDX11	54	265	39	120	38	142
	37	129	23	30	104	19
DDX17	1238	6491	1575	5100	2918	5369
	5370	7829	3228	2151	3926	1174
DDX18	94	385	131	307	198	308
	333	464	201	150	265	80
DDX19A	87	567	93	325	224	396
	476	521	235	154	295	90
DDX19B	62	271	60	145	108	208
	212	231	139	90	182	40
DDX20	50	176	49	160	98	116
	129	204	99	70	139	40
DDX21	113	660	136	424	340	524
	504	724	311	234	419	106
DDX23	132	463	128	341	258	436
	371	608	203	216	304	81
DDX24	215	956	253	720	509	725
	706	1212	456	338	509	179
DDX25	16	1	12	1	5	0
	4	0	11	0	8	0
DDX26B	50	89	23	70	43	63
	68	68	43	19	45	12
DDX27	45	174	38	64	68	105
	106	168	68	58	73	23
DDX28	18	43	8	33	24	18
	32	56	23	7	26	5
DDX31	49	86	39	53	46	56
	64	111	63	28	47	17
DDX39A	9	63	13	28	25	27
	28	31	22	21	18	6
DDX39B	116	582	138	441	272	428
	450	896	335	166	359	91
DDX3X	761	2755	498	2577	2418	2573
	2239	3019	1325	1245	1879	443

DDX3Y	6	836	155	737	2	802
	786	1190	487	0	678	171
DDX4	36	2	18	3	15	3
	2	2	16	1	15	0
DDX41	45	170	32	109	79	106
	99	144	60	41	68	17
DDX42	188	908	163	610	457	537
	715	837	359	288	471	167
DDX43	21	12	21	4	4	6
	12	8	25	0	8	2
DDX46	183	813	170	610	334	523
	651	839	305	244	459	162
DDX47	81	365	89	193	137	212
	285	378	152	89	211	42
DDX49	27	110	21	43	45	94
	63	104	66	34	57	11
DDX5	378	2577	471	2229	1108	2036
	2006	2690	1184	755	1312	495
DDX50	74	264	64	196	134	219
	237	353	150	105	170	39
DDX51	43	54	34	53	29	41
	24	77	23	23	41	15
DDX52	63	269	67	149	137	206
	225	267	116	103	181	49
DDX53	23	3	11	0	11	8
	0	5	6	1	3	0
DDX54	59	191	46	142	100	155
	105	233	118	70	118	34
DDX55	39	117	37	79	49	74
	42	148	50	54	53	19
DDX56	54	187	38	171	91	153
	142	209	135	51	90	24
DDX58	103	320	73	233	156	223
	253	314	192	150	192	51
DDX59	98	304	51	173	175	264
	312	412	159	85	161	66
DDX6	418	2610	568	1799	1209	1656
	1856	2141	858	525	1122	449
DDX60	71	221	68	152	85	114
	164	223	165	105	143	52
DDX60L	82	256	96	161	120	123
	184	183	156	167	149	49
DEAF1	27	42	17	53	20	52
	80	47	29	23	35	9
dec-01	15	0	20	0	6	0
	0	0	9	0	4	1
DECR1	140	877	111	637	304	465
	1233	964	432	233	487	123
DECR2	21	75	17	45	30	58
	77	87	42	16	46	13
DEDD	35	111	24	87	45	123
	94	126	63	34	57	28
DEDD2	20	44	14	27	12	32
	38	57	22	8	27	10
DEF6	18	6	13	18	6	11
	6	16	15	3	4	5
DEF8	34	137	31	76	53	96
	79	163	75	21	70	24

DEFA1	1	0	0	0	1	0
	0	0	0	0	1	0
DEFA1B	3	0	0	0	2	0
	0	0	1	0	1	0
DEFA3	6	0	3	0	0	0
	0	1	1	0	2	0
DEFA4	2	0	2	0	1	0
	0	0	3	0	0	0
DEFA5	4	0	1	0	0	0
	0	0	0	0	0	0
DEFA6	1	0	0	1	0	0
	0	0	1	0	0	0
DEFB1	2	1	0	0	1	0
	1	2	1	0	1	0
DEFB103A	3	0	1	0	0	0
	0	0	0	0	0	0
DEFB103B	1	0	1	0	0	0
	0	0	0	0	0	0
DEFB104A	2	0	3	0	3	0
	0	0	8	0	0	0
DEFB104B	1	0	0	0	0	0
	0	0	2	0	1	0
DEFB105A	2	0	1	0	0	0
	0	0	1	0	1	0
DEFB105B	1	0	0	0	0	0
	0	0	1	0	0	0
DEFB106A	2	0	1	0	0	0
	0	0	1	0	0	0
DEFB106B	4	0	0	0	0	0
	0	0	1	0	0	0
DEFB107A	0	0	0	0	1	0
	0	0	0	0	0	0
DEFB107B	4	0	3	0	0	0
	0	0	4	0	5	0
DEFB108B	1	0	1	0	2	0
	0	0	0	0	0	0
DEFB110	6	0	2	1	0	0
	0	0	6	0	2	0
DEFB112	1	0	1	0	0	0
	0	0	3	0	0	0
DEFB113	1	0	0	0	0	0
	0	0	0	0	0	0
DEFB114	3	0	0	0	0	0
	0	0	0	0	0	0
DEFB115	0	0	1	0	1	0
	0	0	0	0	0	0
DEFB116	2	0	1	0	0	0
	0	0	2	0	0	0
DEFB118	2	0	0	0	0	0
	0	0	2	0	2	0
DEFB119	6	0	2	0	0	0
	0	0	1	0	2	0
DEFB121	9	0	2	0	0	0
	0	0	2	0	1	0
DEFB123	3	0	1	0	1	0
	0	0	1	0	0	0
DEFB124	2	1	0	0	0	0
	0	0	0	0	0	0

DEFB125	2	0	3	0	0	0
	0	0	4	0	1	0
DEFB126	1	0	1	0	0	0
	0	0	0	0	0	0
DEFB127	4	0	0	0	0	0
	0	0	1	0	0	0
DEFB128	6	0	1	0	0	0
	0	0	0	0	1	0
DEFB129	9	0	2	0	0	0
	0	0	4	0	1	0
DEFB130	3	0	0	0	1	0
	0	0	1	0	0	0
DEFB131	2	0	3	0	0	0
	0	0	0	0	0	0
DEFB132	18	0	8	0	6	0
	0	0	4	0	5	0
DEFB134	1	0	0	0	1	0
	0	0	1	0	0	0
DEFB136	1	0	0	0	0	0
	0	0	0	0	1	0
DEFB4A	2	0	0	0	0	0
	0	0	1	0	0	0
DEGS1	17	104	23	107	67	76
	77	83	38	15	66	22
DEGS2	4	0	4	0	0	2
	0	0	2	0	1	0
DEK	321	1535	279	990	975	1245
	1225	2095	772	664	935	312
DEM1	9	20	11	32	17	27
	20	38	18	3	10	4
DENND1A	42	99	48	66	55	66
	88	115	60	45	74	28
DENND1B	151	545	158	532	311	391
	458	671	283	166	398	101
DENND1C	14	0	10	1	3	0
	1	1	6	0	3	1
DENND2A	21	58	21	45	31	39
	34	61	27	16	44	5
DENND2C	995	3942	923	2756	1819	3274
	1807	3077	1361	876	987	453
DENND2D	11	9	12	3	8	6
	5	17	11	1	12	1
DENND3	51	92	24	53	39	37
	82	104	56	27	62	23
DENND4A	171	638	213	566	339	516
	416	684	346	200	369	138
DENND4B	63	339	50	192	126	192
	215	298	99	112	136	53
DENND4C	197	766	206	711	412	649
	560	940	476	219	515	162
DENND5A	98	342	83	266	170	266
	230	312	154	109	202	51
DENND5B	137	636	156	369	323	373
	353	469	258	211	305	91
DENND6A	86	279	101	225	142	200
	252	354	187	80	212	57
DENND6B	5	1	5	4	3	2
	2	1	2	1	2	0

DENR	130	809	131	456	279	477
	563	653	267	177	377	115
DEPDC1	28	2	29	6	9	6
	5	5	22	0	22	2
DEPDC1B	10	0	13	2	5	0
	1	2	16	0	8	1
DEPDC4	8	2	9	7	1	8
	4	4	7	0	10	0
DEPDC5	85	114	51	90	68	83
	76	107	84	44	87	23
DEPDC7	17	49	18	25	37	52
	33	55	28	20	22	8
DEPTOR	276	1716	379	1319	987	1512
	1754	1860	1022	566	936	355
DERA	66	313	65	155	127	166
	300	297	131	71	142	36
DERL1	92	327	88	221	169	247
	309	665	203	124	264	99
DERL2	35	87	25	87	60	84
	132	162	53	29	67	19
DERL3	4	7	7	5	0	2
	2	4	4	1	4	0
DES	11970	59704	10118	37130	22979	47007
	42309	59827	24685	22797	30975	11257
DESI1	45	132	32	71	91	97
	127	175	69	50	72	31
DESI2	125	690	121	516	380	587
	603	609	289	199	357	114
DET1	36	96	29	63	34	68
	67	132	59	27	62	11
DEXI	66	333	33	177	163	199
	274	290	114	92	168	45
DFFA	35	108	37	54	41	100
	74	131	65	42	63	31
DFFB	11	40	10	18	16	36
	15	18	20	10	17	5
DFNA5	20	30	12	26	17	9
	12	34	19	11	10	5
DFNB31	16	3	9	0	2	2
	0	2	7	0	8	0
DFNB59	13	24	12	46	10	12
	20	26	21	3	18	3
DGAT1	35	62	17	46	22	49
	43	109	55	11	40	16
DGAT2	23	29	16	2	10	16
	13	18	6	1	6	24
DGAT2L6	19	0	3	0	2	0
	0	0	1	0	4	0
DGAT2L7P	5	0	2	0	0	0
	0	0	1	0	1	0
DGCR14	50	44	26	19	28	27
	24	48	32	16	36	3
DGCR2	51	196	58	115	75	108
	105	190	108	50	112	26
DGCR6	54	260	55	89	144	325
	373	468	241	138	293	48
DGCR6L	25	83	8	83	34	85
	106	84	59	25	55	14

DGCR8	82	380	91	208	175	237	237	374	131	96	159	57
DGKA	37	28	22	35	11	21	10	18	18	2	18	5
DGKB	82	7	42	15	28	13	10	12	42	3	32	1
DGKD	179	779	215	549	359	635	415	902	400	242	417	138
DGKE	48	112	27	72	48	53	82	143	77	33	70	22
DGKG	61	49	27	24	34	26	31	83	34	17	50	13
DGKH	72	175	54	107	101	57	139	155	87	30	69	26
DGKI	43	0	42	3	15	4	0	2	26	0	19	0
DGKK	50	0	9	0	4	0	0	0	15	0	4	0
DGKQ	15	19	11	25	7	11	15	32	13	4	15	1
DGKZ	88	406	81	192	188	276	312	315	143	121	177	74
DGUOK	14	55	12	32	24	25	36	73	39	12	31	7
DHCR24	83	340	46	118	174	426	368	276	139	63	238	71
DHCR7	18	21	15	15	12	33	27	27	28	8	20	10
DHDDS	41	143	38	70	47	68	122	123	76	32	66	26
DHDH	8	0	1	0	0	4	0	0	2	0	0	0
DHFR	81	261	72	226	123	155	202	228	72	57	79	28
DHFRL1	39	30	25	40	29	52	30	60	15	14	30	11
DHH	4	1	2	0	1	0	0	0	3	0	0	0
DHODH	21	55	23	24	34	34	53	65	31	20	45	11
DHPS	71	236	27	163	86	115	187	279	125	66	133	38
DHRS1	16	37	13	18	7	22	29	13	19	6	17	8
DHRS11	13	41	10	25	29	29	41	38	14	14	20	6
DHRS12	26	55	23	43	30	43	79	75	67	16	65	13
DHRS13	8	3	4	5	5	5	4	8	9	0	2	0
DHRS2	17	5	15	2	2	4	2	5	2	0	2	0
DHRS3	32	226	51	218	114	133	193	277	142	66	132	65
DHRS4	12	114	17	61	53	66	110	118	33	11	49	10
DHRS4L1	8	4	8	1	1	2	0	7	7	0	14	0

DHRS4L2	21	90	16	46	41	56
	98	91	30	22	41	17
DHRS7	298	1895	352	1599	653	1529
	1482	1784	917	410	766	169
DHRS7B	76	313	61	249	160	280
	233	395	151	106	192	41
DHRS7C	105	534	105	449	203	533
	693	905	361	166	539	73
DHRS9	35	3	18	1	12	14
	1	5	11	2	7	1
DHRSX (NC_000023 2137546..2419015)				15	43	4
	12	4	29	21	43	11
	4	18	5			
DHRSX (NC_000024 2087546..2369015)				23	31	14
	16	7	8	17	15	22
	9	19	7			
DHTKD1	79	402	60	200	145	147
	293	523	171	75	267	88
DHX15	116	609	149	421	289	437
	538	722	285	211	410	114
DHX16	63	232	59	146	105	222
	155	285	112	78	160	49
DHX29	142	705	145	511	335	569
	430	687	288	235	397	147
DHX30	66	222	65	170	105	156
	207	305	107	78	159	46
DHX32	86	295	61	210	174	166
	257	340	163	97	198	60
DHX33	46	132	32	86	65	66
	117	118	56	42	79	30
DHX34	27	53	19	29	28	39
	24	75	22	17	23	11
DHX35	71	205	57	153	111	167
	138	333	134	74	162	36
DHX36	105	541	132	429	233	330
	413	620	234	141	339	106
DHX37	42	51	20	46	28	43
	43	66	31	18	44	13
DHX38	84	331	92	251	150	243
	191	350	154	90	170	75
DHX40	85	299	74	234	149	298
	282	412	178	105	266	65
DHX57	75	216	68	191	110	168
	133	213	104	95	140	43
DHX58	15	22	9	20	11	12
	22	17	15	18	20	5
DHX8	96	263	75	190	144	181
	165	323	153	102	161	57
DHX9	185	904	201	608	418	547
	597	939	366	287	534	177
DIABLO	38	170	46	140	59	102
	189	177	73	42	100	27
DIAPH1	182	786	188	481	308	400
	862	1402	473	313	605	218
DIAPH2	189	790	157	620	504	571
	634	874	431	313	378	184
DIAPH3	41	0	31	2	10	2
	0	2	28	0	23	0

DICER1	515	2463	669	1989	1190	2086
	1937	2709	1261	926	1658	541
DIDO1	160	526	135	366	303	396
	357	548	270	184	311	124
DIEXF	91	127	43	97	91	86
	111	165	98	37	81	35
DIMT1	32	122	26	71	55	105
	160	184	71	56	85	28
DIO1	19	2	2	1	2	1
	0	0	3	0	1	0
DIO2	70	97	38	97	55	95
	48	116	86	20	69	32
DIO3	8	2	6	2	3	1
	4	2	3	0	4	0
DIP2A	111	410	161	393	172	353
	298	377	192	119	201	97
DIP2B	194	398	145	335	290	354
	304	519	240	153	274	103
DIP2C	166	608	173	474	358	380
	592	800	373	237	441	155
DIRAS1	14	5	9	0	3	1
	0	0	4	0	5	2
DIRAS2	17	0	18	0	4	0
	1	0	11	0	4	1
DIRAS3	7	7	10	12	8	18
	8	7	12	4	5	3
DIRC1	14	1	15	0	6	0
	0	0	13	1	3	0
DIRC2	148	450	111	371	276	302
	576	773	381	210	453	123
DIS3	119	426	108	321	230	259
	335	604	257	135	296	93
DIS3L	148	550	165	509	256	428
	395	584	253	223	419	130
DIS3L2	69	172	40	110	72	99
	128	164	73	49	86	21
DISC1	30	84	57	64	35	53
	54	100	76	28	59	20
DISP1	49	140	38	112	70	96
	70	83	60	52	76	15
DISP2	17	0	10	0	3	0
	0	0	4	0	3	0
DIXDC1	222	529	156	393	420	444
	577	752	421	139	496	101
DKC1	60	248	52	156	115	154
	177	197	86	76	144	36
DKK1	6	0	6	1	3	0
	3	1	6	1	2	0
DKK2	17	130	25	25	30	20
	13	25	15	5	13	8
DKK3	14	44	36	66	18	33
	33	39	33	9	22	15
DKK4	4	0	5	1	2	1
	0	1	3	0	3	0
DKKL1	6	0	3	0	2	0
	1	0	1	0	2	0
DLAT	245	1463	246	984	661	953
	1534	1601	670	453	942	238

DLC1	139	674	132	509	296	387
	545	589	302	212	282	104
DLD	586	3670	475	2678	1608	2161
	3711	3536	1367	939	2056	483
DLEC1	43	4	30	10	9	0
	1	1	13	1	10	0
DLEU7	9	0	4	0	0	3
	0	0	3	0	1	0
DLG1	158	714	188	388	363	481
	460	686	293	201	315	148
DLG2	98	80	69	80	67	67
	79	91	97	27	75	24
DLG3	65	49	21	35	21	33
	22	33	21	8	19	6
DLG4	38	52	22	24	23	31
	21	27	9	10	16	5
DLG5	103	236	92	203	103	235
	216	298	147	86	181	44
DLGAP1	38	5	24	3	10	5
	6	3	36	2	15	0
DLGAP2	56	1	28	0	8	0
	1	0	18	1	11	0
DLGAP3	9	0	12	1	2	2
	1	0	6	1	4	0
DLGAP4	46	367	66	220	109	210
	222	143	58	56	90	39
DLGAP5	34	1	9	0	6	2
	0	0	23	0	9	1
DLK1	22	30	5	57	35	38
	25	5	31	10	12	4
DLK2	8	1	5	0	0	0
	1	0	4	0	0	0
DLL1	15	39	17	14	6	6
	27	15	13	3	9	4
DLL3	1	0	5	0	0	0
	0	0	2	0	3	0
DLL4	10	56	9	25	18	22
	33	42	28	17	22	6
DLST	156	821	137	570	340	531
	806	970	307	253	469	169
DLX1	5	0	11	0	1	0
	0	2	3	0	3	0
DLX2	5	0	1	0	0	0
	0	0	4	0	2	0
DLX3	15	0	7	0	5	0
	1	0	6	0	1	0
DLX4	8	0	5	0	1	0
	0	0	4	0	3	0
DLX5	13	0	2	1	5	2
	0	1	4	0	3	0
DLX6	12	0	3	0	0	0
	0	0	4	0	0	1
DMAP1	25	71	19	57	51	48
	101	59	46	31	56	23
DMBT1	47	0	35	0	6	0
	0	0	41	0	14	0
DMBX1	4	0	3	0	0	0
	0	0	2	0	1	0

DMC1	28	6	14	14	10	9
	4	7	21	1	10	0
DMD	4257	22461	6146	13054	13724	14527
	13831	23262	8929	10063	12795	4716
DMGDH	29	59	27	53	24	47
	75	67	38	25	57	13
DMKN	21	1	14	0	2	0
	0	3	11	1	4	0
DMP1	21	0	15	0	0	0
	0	1	10	0	2	0
DMPK	69	293	153	251	111	260
	253	368	195	100	145	60
DMRT1	11	0	5	0	2	0
	0	0	8	0	5	0
DMRT2	21	21	11	11	10	17
	43	27	19	8	11	6
DMRT3	5	3	3	2	3	0
	4	1	6	2	3	0
DMRTA1	11	22	12	33	12	16
	9	21	23	4	18	6
DMRTA2	7	0	4	0	1	0
	0	0	0	0	3	0
DMRTB1	7	0	1	0	1	0
	0	0	2	0	1	0
DMRTC1	19	0	5	1	3	1
	0	0	2	0	2	0
DMRTC1B	4	0	1	0	0	0
	0	0	0	0	1	0
DMRTC2	9	0	6	0	1	0
	0	0	5	0	3	0
DMTF1	159	745	194	430	317	461
	487	764	344	237	390	148
DMWD	17	52	22	29	35	65
	60	93	36	18	41	13
DMXL1	278	970	328	891	535	826
	726	1272	573	323	692	210
DMXL2	120	314	143	250	154	230
	129	246	148	69	159	48
DNA2	44	72	25	49	28	58
	43	35	51	17	11	5
DNAAF1	30	0	12	0	4	0
	0	0	9	1	7	1
DNAAF2	27	68	9	54	39	48
	73	90	30	25	49	14
DNAAF3	7	0	5	0	1	0
	1	0	6	0	1	0
DNAH1	99	125	70	70	49	69
	67	118	82	31	76	19
DNAH10	124	8	63	7	22	8
	3	13	72	4	27	1
DNAH11	167	213	283	107	53	77
	64	33	108	9	85	40
DNAH12	89	5	57	4	23	4
	10	15	76	4	29	0
DNAH14	105	14	87	10	35	8
	9	7	84	2	53	5
DNAH17	85	5	36	1	11	2
	1	4	36	3	19	1

DNAH2	105	3	70	0	24	0
	5	7	51	0	22	0
DNAH3	138	23	87	36	35	9
	12	55	64	20	87	54
DNAH5	128	2	75	3	46	15
	4	12	72	2	60	1
DNAH6	120	1	107	3	35	2
	7	7	74	4	49	2
DNAH7	108	3	72	9	31	3
	5	4	71	1	47	3
DNAH8	168	4	62	8	45	3
	1	0	104	0	39	0
DNAH9	133	0	68	3	30	5
	0	1	66	0	29	1
DNAI1	24	1	15	0	5	0
	1	0	16	0	7	0
DNAI2	10	0	6	0	0	0
	0	0	7	0	1	0
DNAJA1	57	312	69	173	188	278
	184	226	103	93	164	49
DNAJA2	263	1087	199	833	621	837
	965	1491	614	414	806	246
DNAJA3	112	535	67	340	226	343
	420	631	280	138	288	94
DNAJA4	137	917	84	307	467	462
	476	859	162	243	332	129
DNAJB1	53	295	69	184	123	257
	201	260	91	71	132	46
DNAJB11	33	176	32	98	73	133
	134	187	90	45	121	44
DNAJB12	78	276	82	196	113	258
	220	278	121	107	165	71
DNAJB13	11	1	3	0	2	2
	0	0	5	0	0	0
DNAJB14	47	217	33	143	101	118
	137	193	99	48	136	34
DNAJB2	124	633	118	424	256	520
	424	734	314	127	325	89
DNAJB3	5	0	2	0	3	0
	0	0	1	0	1	0
DNAJB4	175	1265	256	838	642	882
	1077	1263	506	365	743	167
DNAJB5	88	618	77	375	301	520
	857	548	297	169	441	104
DNAJB6	330	1725	242	1009	694	1228
	1328	1891	747	483	1020	271
DNAJB7	10	2	10	2	5	0
	0	1	5	0	1	0
DNAJB8	10	0	3	0	2	0
	0	0	6	0	5	0
DNAJB9	76	310	60	278	153	256
	305	459	170	110	216	45
DNAJC1	30	97	36	94	48	111
	96	154	87	41	83	32
DNAJC10	82	465	99	430	223	409
	347	472	252	133	272	97
DNAJC11	89	470	73	302	197	230
	416	403	176	103	223	78

DNAJC12	20	65	7	34	25	57	45	94	34	27	42	15
DNAJC13	250	1182	297	824	565	770	666	979	472	326	560	202
DNAJC14	39	118	30	65	45	61	79	136	56	25	68	19
DNAJC15	99	545	90	273	202	374	450	627	233	99	186	65
DNAJC16	97	341	77	241	165	232	222	364	144	97	140	63
DNAJC17	21	84	24	44	33	49	50	85	40	31	43	12
DNAJC18	72	143	27	151	94	145	176	131	79	52	115	22
DNAJC19	153	574	107	522	261	497	757	1050	381	196	450	111
DNAJC2	45	195	31	92	63	103	123	156	63	51	99	39
DNAJC21	214	1268	171	724	534	815	871	997	373	376	583	227
DNAJC22	11	0	4	2	2	2	1	3	3	1	6	1
DNAJC24	58	264	80	223	132	233	216	281	167	96	210	34
DNAJC25	6	55	2	45	19	57	40	65	14	14	22	13
DNAJC25-GNG10	28	86	7	43	37	44	73	83	56	21	36	6
DNAJC27	61	167	43	136	69	106	170	226	106	46	128	24
DNAJC28	16	79	14	67	35	45	54	59	35	29	38	9
DNAJC3	99	534	92	373	223	417	355	513	233	172	212	98
DNAJC30	14	29	7	12	17	15	29	24	12	15	15	7
DNAJC4	18	95	8	31	36	35	56	60	26	12	27	5
DNAJC5	77	202	44	149	89	146	165	246	109	66	136	49
DNAJC5B	10	0	6	1	8	0	2	4	7	0	8	2
DNAJC5G	13	0	3	0	1	0	0	0	4	0	3	0
DNAJC6	50	21	39	14	16	15	6	14	24	6	16	4
DNAJC7	106	631	122	437	199	542	474	470	253	178	285	108
DNAJC8	62	358	59	231	160	248	282	377	157	137	163	43
DNAJC9	50	136	49	84	60	82	90	184	72	35	69	23
DNAL1	55	125	36	69	60	74	83	128	78	30	88	28
DNAL4	12	24	7	19	16	17	18	25	13	3	9	2

DNALI1	17	12	8	11	13	6
	10	16	11	3	11	5
DNASE1	19	19	13	11	7	11
	10	10	10	5	10	6
DNASE1L1	185	397	130	510	309	706
	615	939	385	269	486	95
DNASE1L2	3	2	4	0	0	0
	1	0	0	0	0	0
DNASE1L3	14	7	4	2	2	0
	0	4	6	0	1	0
DNASE2	37	88	13	71	39	90
	20	78	45	12	44	17
DNASE2B	14	1	11	2	1	2
	1	5	14	1	2	2
DND1	6	2	2	1	3	1
	2	6	2	1	0	0
DNER	14	2	7	0	5	4
	0	2	11	0	4	0
DNHD1	160	265	101	195	128	240
	168	297	169	79	216	47
DNLZ	2	7	0	8	1	23
	10	20	5	3	5	0
DNM1	21	35	20	56	26	23
	9	71	19	8	21	18
DNM1L	230	1171	176	799	404	810
	960	1193	533	287	443	222
DNM2	96	563	96	377	185	333
	400	447	190	154	245	95
DNM3	58	90	40	57	44	57
	75	58	84	22	51	11
DNMBP	103	347	111	216	218	203
	226	265	180	100	186	63
DNMT1	54	192	55	80	60	116
	80	167	62	42	74	35
DNMT3A	88	426	81	295	208	264
	249	281	171	114	156	81
DNMT3B	33	17	18	13	17	15
	8	14	16	3	24	3
DNMT3L	7	0	6	0	1	0
	0	0	3	0	1	0
DNPEP	48	118	26	83	48	99
	130	114	71	41	69	30
DNTT	15	0	13	0	6	0
	0	0	15	0	3	0
DNTTIP1	30	159	32	113	89	98
	209	216	63	37	94	30
DNTTIP2	122	639	161	452	359	543
	445	601	251	223	389	130
DOC2A	9	4	4	1	2	1
	1	0	10	0	0	0
DOC2B	6	7	5	1	6	1
	2	6	5	5	6	0
DOCK1	132	342	93	248	140	168
	213	284	176	102	169	64
DOCK10	72	71	56	72	46	38
	42	50	87	18	58	14
DOCK11	87	216	52	157	135	121
	114	201	84	53	130	105

DOCK2	96	55	71	65	45	52
	46	88	78	17	48	11
DOCK3	111	73	107	116	34	57
	74	78	77	39	97	16
DOCK4	184	712	149	352	268	428
	612	765	414	232	434	125
DOCK5	134	163	99	175	88	234
	158	144	175	79	85	45
DOCK6	54	121	34	96	45	57
	122	81	47	28	63	27
DOCK7	130	270	111	245	148	213
	153	220	133	75	149	45
DOCK8	108	102	56	71	49	68
	45	67	74	20	62	14
DOCK9	230	749	186	609	390	481
	606	970	513	319	589	160
DOHH	7	23	1	12	9	31
	5	43	16	5	5	1
DOK1	11	8	3	5	3	3
	1	10	6	2	6	2
DOK2	6	1	5	2	5	2
	1	7	1	0	0	0
DOK3	18	12	5	7	3	13
	4	7	6	1	6	2
DOK4	25	25	9	12	12	17
	9	13	14	3	10	2
DOK5	40	264	35	203	132	189
	219	237	122	66	180	45
DOK6	42	10	23	12	11	2
	5	2	20	3	20	3
DOK7	16	20	8	35	12	16
	21	35	37	12	29	8
DOLK	13	37	16	33	20	33
	31	42	25	10	29	6
DOLPP1	6	32	6	24	10	31
	27	69	34	15	26	8
DOM3Z	8	29	8	16	14	25
	27	23	19	9	15	7
DONSON	26	59	21	44	40	44
	37	96	51	20	28	16
DOPEY1	176	711	269	592	374	568
	685	912	512	323	646	136
DOPEY2	125	495	81	301	254	296
	355	354	200	136	186	72
DOT1L	26	151	26	55	48	64
	74	96	47	41	50	13
DPAGT1	15	22	14	22	17	28
	16	36	19	9	20	3
DPCD	21	48	11	20	13	16
	30	57	21	10	20	5
DPCR1	68	0	30	0	15	0
	0	0	27	0	18	0
DPEP1	3	0	7	0	1	0
	0	0	2	0	1	0
DPEP2	4	13	5	6	5	3
	12	3	12	0	4	0
DPEP3	8	0	2	1	1	0
	1	0	2	0	1	1

DPF1	6	0	8	0	0	0
	0	0	2	0	1	0
DPF2	59	125	39	68	79	99
	120	143	58	23	72	22
DPF3	88	390	84	235	188	283
	312	362	125	166	193	45
DPH1	60	211	51	135	87	161
	208	259	126	64	134	45
DPH2	28	113	16	48	32	83
	102	119	51	24	56	16
DPH3	62	362	61	271	147	327
	187	367	165	81	153	44
DPH3P1	2	0	1	0	0	0
	0	0	1	0	0	0
DPH5	43	187	36	97	75	111
	171	160	74	49	96	15
DPM1	58	461	58	312	144	262
	413	529	225	82	270	78
DPM2	12	15	5	6	7	13
	8	30	9	5	10	2
DPM3	14	53	8	43	21	41
	72	73	35	20	42	11
DPP10	55	0	28	0	11	1
	0	0	25	0	18	1
DPP3	32	66	20	40	27	40
	37	88	55	21	52	15
DPP4	47	67	61	86	56	59
	20	101	51	6	39	18
DPP6 (NC_000007 153584418..154264025)				12	9	12
	13	6	7	6	12	5
	0	5	1			
DPP6 (NC_000007 154400204..154685995)				43	19	18
	24	14	9	11	23	25
	4	24	3			
DPP7	31	85	18	60	38	66
	78	98	46	39	54	18
DPP8	239	1139	258	998	521	882
	1027	910	484	307	641	180
DPP9	85	267	46	136	112	163
	192	299	117	87	164	56
DPPA2	20	0	11	0	1	0
	0	0	8	0	3	0
DPPA3	12	0	4	0	0	0
	0	0	4	0	2	0
DPPA4	16	0	4	2	7	0
	0	0	7	0	2	0
DPPA5	3	0	3	0	0	0
	0	0	0	0	0	0
DPRX	9	2	0	2	5	2
	2	2	5	4	4	0
DPT	71	342	75	262	168	395
	123	334	152	60	128	119
DPY19L1	51	100	51	69	68	91
	77	132	72	37	56	17
DPY19L2	32	66	32	37	33	46
	39	58	62	11	51	6
DPY19L3	62	151	45	127	86	110
	114	136	73	42	100	19

DPY19L4	99	369	101	285	220	297
	214	507	222	118	242	69
DPY30	25	101	11	97	42	92
	134	192	76	29	60	23
DPYD	107	437	114	299	199	317
	395	652	268	149	331	98
DPYS	15	2	8	2	5	1
	3	1	6	0	2	2
DPYSL2	164	666	126	587	381	538
	621	1087	416	266	396	170
DPYSL3	298	708	124	438	550	443
	702	1214	255	410	530	282
DPYSL4	15	4	7	7	6	6
	5	7	10	0	4	0
DPYSL5	35	1	6	0	2	0
	2	0	10	1	7	0
DQX1	19	1	11	0	8	0
	0	0	7	0	7	0
DR1	129	566	124	455	332	446
	522	690	316	162	344	119
DRAM1	28	54	14	42	22	45
	48	56	34	11	39	12
DRAM2	51	258	36	180	115	184
	148	222	109	63	95	30
DRAP1	57	240	45	160	112	245
	283	308	132	56	138	33
DRAXIN	11	0	4	0	1	0
	0	0	5	0	3	0
DRD1	11	3	11	2	3	0
	0	0	10	2	4	0
DRD2	16	1	8	4	5	1
	0	2	8	0	13	1
DRD3	10	0	6	0	2	0
	0	0	5	0	1	0
DRD4	2	1	0	0	0	1
	3	1	2	1	0	2
DRD5	11	0	7	1	2	0
	0	0	1	0	0	0
DRG1	129	586	119	444	225	417
	563	804	360	166	424	101
DRG2	38	126	37	122	57	102
	82	160	71	45	82	32
DRGX	8	0	4	0	1	0
	0	0	6	0	2	0
DROSHA	118	487	133	300	210	304
	307	398	199	119	227	71
DRP2	53	0	14	10	16	1
	3	1	10	4	10	8
DSC1	32	0	22	0	11	0
	0	1	24	1	16	1
DSC2	33	27	25	25	29	36
	26	48	43	6	34	4
DSC3	32	1	24	2	14	2
	1	0	29	0	9	0
DSCAM	55	2	29	1	10	2
	2	0	21	0	9	1
DSCAML1	39	9	27	7	10	3
	3	8	15	3	8	2

DSCC1	24	55	12	24	24	16
	30	51	23	20	22	18
DSCR3	78	258	50	216	139	239
	211	357	173	103	192	55
DSCR4	6	0	2	0	1	0
	0	0	4	0	1	0
DSCR6	5	0	4	0	2	0
	0	0	3	0	0	0
DSE	73	339	88	231	153	266
	205	361	114	90	165	60
DSEL	48	76	57	120	48	72
	47	110	61	23	62	29
DSG1	31	0	21	2	13	0
	0	0	27	1	18	0
DSG2	50	67	40	32	36	51
	41	59	70	14	54	6
DSG3	39	0	32	0	7	0
	0	0	20	0	5	0
DSG4	29	0	29	0	15	0
	0	0	20	0	10	0
DSN1	24	32	31	29	17	22
	34	51	34	12	41	10
DSP	69	27	42	4	43	9
	6	12	50	5	32	13
DSPP	29	1	15	1	11	1
	0	1	19	2	9	0
DST	3225	19662	4009	12490	11188	12326
	11311	16964	6730	6141	10452	3619
DSTN	109	553	101	484	274	415
	554	647	245	166	305	167
DSTYK	122	254	79	250	161	250
	259	311	160	103	165	60
DTD1	24	92	19	68	32	70
	95	87	68	23	66	10
DTD2	25	68	25	44	32	33
	35	70	36	23	30	9
DTHD1	41	5	23	1	8	2
	0	3	18	1	14	0
DTL	20	3	20	3	4	2
	4	3	14	1	10	0
DTNA	568	2558	538	1870	1582	2319
	2770	3597	1523	914	2133	544
DTNB	34	23	24	21	21	30
	39	20	38	2	27	9
DTNBP1	24	79	15	56	28	45
	57	66	21	13	28	9
DTWD1	52	150	48	144	103	107
	137	247	78	63	87	38
DTWD2	123	383	87	334	213	300
	329	414	224	126	279	67
DTX1	20	9	4	11	3	5
	1	2	5	3	3	2
DTX2	27	48	17	29	22	41
	48	34	32	14	31	8
DTX3	18	49	12	16	14	23
	20	37	19	13	17	6
DTX3L	70	248	64	142	148	141
	194	297	105	147	163	59

DTX4	48	33	21	28	27	28
	16	23	22	8	23	5
DTYMK	7	29	10	21	11	12
	16	32	14	7	9	2
DUOX1	44	2	26	8	9	4
	2	5	20	1	9	4
DUOX2	37	0	17	0	5	1
	0	0	16	0	7	1
DUOXA1	9	0	3	0	1	0
	0	0	6	0	0	1
DUOXA2	5	0	7	0	1	0
	0	0	0	0	3	0
DUPD1	12	82	16	51	33	47
	44	54	38	18	32	9
DUS1L	28	138	32	87	66	103
	130	171	64	41	98	33
DUS2L	26	30	12	32	28	25
	21	42	31	10	35	4
DUS3L	12	33	6	24	24	31
	14	41	17	7	18	7
DUS4L	32	46	16	50	19	42
	48	69	30	23	45	12
DUSP1	47	202	57	202	178	186
	181	362	188	71	233	88
DUSP10	69	490	104	274	192	213
	361	410	182	130	249	49
DUSP11	31	111	37	89	80	84
	142	189	79	41	84	20
DUSP12	16	48	13	36	19	53
	40	67	39	22	30	8
DUSP13	139	377	66	417	175	440
	542	579	258	166	219	67
DUSP14	9	39	2	35	14	18
	11	20	10	3	11	2
DUSP15	9	2	8	1	0	2
	1	0	2	0	2	0
DUSP16	77	318	99	260	172	260
	182	237	111	98	150	48
DUSP18	13	11	9	7	11	9
	14	10	3	4	6	2
DUSP19	32	91	40	58	77	94
	56	85	55	23	54	8
DUSP2	4	4	6	8	2	4
	4	4	6	2	1	0
DUSP21	1	0	2	0	0	0
	0	0	3	0	0	0
DUSP22	40	200	36	135	85	137
	167	198	76	55	90	23
DUSP23	18	71	12	41	13	36
	73	108	44	12	30	10
DUSP26	399	1539	189	1298	717	1560
	1652	2473	948	568	1010	350
DUSP27	268	1203	313	831	623	1028
	906	1252	521	363	768	220
DUSP28	17	59	13	42	18	58
	63	101	45	22	36	15
DUSP3	440	2943	222	2549	1308	2795
	2735	2174	1395	847	1435	308

DUSP4	25	8	24	14	6	11
	14	14	17	6	19	23
DUSP5	15	13	9	6	6	4
	10	12	7	3	7	2
DUSP6	21	105	25	119	58	56
	76	77	27	19	62	11
DUSP7	25	102	30	82	72	87
	119	125	70	36	56	22
DUSP8	16	11	6	9	10	7
	13	36	10	6	8	4
DUSP9	8	0	1	0	3	0
	2	0	0	0	0	0
DUT	71	272	45	191	116	157
	263	296	138	69	137	24
DUX2	3	0	0	0	1	0
	0	0	2	0	0	0
DUX4	2	0	0	0	0	0
	0	0	0	0	0	0
DUX4L2	1	0	0	0	1	0
	0	0	0	0	0	0
DUX4L3	0	0	1	0	0	0
	0	0	0	0	0	0
DUX4L4	9	0	3	0	2	0
	0	0	5	0	2	0
DUX4L5	0	0	1	0	1	0
	0	0	1	0	1	0
DUX4L7	10	0	1	0	1	0
	0	0	0	0	1	0
DUXA	20	3	9	2	4	3
	1	0	12	0	7	0
DVL1	154	645	98	388	273	416
	433	826	327	239	423	141
DVL2	29	75	28	28	36	57
	45	81	44	17	54	17
DVL3	71	282	79	183	132	229
	205	206	116	87	137	79
DYDC1	11	0	2	0	1	0
	0	0	5	0	5	0
DYDC2	14	0	6	0	3	0
	0	2	4	0	8	1
DYM	93	565	91	386	251	431
	376	475	276	175	360	99
DYNAP	42	2	37	2	7	1
	1	2	16	1	15	0
DYNC1H1	1195	5701	1229	4675	2995	3611
	3434	4485	2031	1695	2575	1144
DYNC1I1	47	54	30	70	19	29
	51	47	51	5	45	9
DYNC1I2	119	539	127	360	241	382
	427	493	219	160	292	81
DYNC1LI1	107	476	89	403	248	408
	376	460	205	160	243	75
DYNC1LI2	237	1181	248	764	642	940
	850	1322	494	420	617	246
DYNC2H1	140	210	177	246	167	196
	180	307	203	87	206	58
DYNC2LI1	29	85	24	66	49	53
	80	113	66	17	76	12

DYNLL1	39	158	35	161	67	102
	117	112	56	26	64	17
DYNLL2	98	599	107	328	234	446
	423	385	168	122	245	70
DYNLRB1	91	502	79	190	227	384
	454	534	142	153	232	49
DYNLRB2	2	0	4	0	0	0
	2	1	1	0	1	0
DYNLT1	62	280	53	166	124	175
	274	351	103	75	136	29
DYNLT3	48	86	30	62	55	88
	91	163	83	38	81	24
DYRK1A	324	1712	329	1297	860	1175
	1341	1901	834	565	938	302
DYRK1B	117	603	89	398	280	363
	466	523	190	160	299	111
DYRK2	390	2051	497	1619	977	1316
	1230	1751	905	478	1063	274
DYRK3	11	15	14	9	6	16
	8	7	13	3	8	3
DYRK4	16	59	21	53	20	43
	41	91	35	22	34	7
DYSF	366	1713	372	1105	834	1055
	1045	1137	496	362	601	236
DYTN	15	0	9	0	5	0
	2	0	13	0	4	0
DYX1C1	51	22	21	6	25	16
	17	40	34	13	18	6
DZANK1	43	9	22	7	9	10
	24	4	25	3	20	3
DZIP1	70	84	38	88	42	74
	34	56	45	21	53	19
DZIP1L	29	24	31	22	17	10
	23	37	26	11	28	6
DZIP3	161	620	175	402	260	353
	428	712	298	188	408	114
E2F1	5	4	11	1	1	4
	1	1	2	2	0	1
E2F2	28	1	6	5	1	3
	0	1	7	1	6	0
E2F3	37	122	28	89	33	66
	75	46	44	16	29	8
E2F4	41	174	27	110	76	121
	119	150	89	51	81	21
E2F5	8	9	10	6	9	14
	11	4	11	0	5	3
E2F6	85	208	42	183	120	176
	162	267	97	78	146	43
E2F7	36	0	14	0	6	1
	0	0	17	0	13	0
E2F8	71	460	115	302	105	217
	272	173	165	72	119	57
E4F1	17	68	18	30	29	53
	53	76	35	23	41	20
EAF1	55	150	30	141	63	107
	146	186	88	41	92	31
EAF2	14	35	9	12	10	17
	15	27	16	13	14	5

EAPP	41	198	43	129	70	122
	151	288	121	60	149	44
EARS2	63	226	41	149	92	119
	152	177	102	74	123	33
EBAG9	87	582	82	339	240	468
	547	499	221	156	273	67
EBF1	71	287	93	308	153	222
	193	324	90	62	131	89
EBF2	64	179	77	221	125	145
	148	201	96	63	88	36
EBF3	38	212	35	212	75	143
	164	151	102	79	100	29
EBF4	8	3	8	1	2	10
	4	5	8	1	6	1
EBI3	5	1	2	0	1	0
	0	1	3	0	0	0
EBLN1	5	0	4	0	0	0
	1	0	3	0	3	1
EBLN2	0	0	0	1	0	0
	0	0	0	0	0	0
EBNA1BP2	48	197	40	113	89	150
	140	209	104	61	103	29
EBP	12	26	6	18	7	28
	40	56	11	13	23	4
EBPL	13	21	8	18	21	32
	12	31	12	10	13	4
ECD	84	299	80	191	143	202
	200	279	186	112	199	63
ECE1	66	218	60	173	99	96
	154	266	110	55	140	53
ECE2	16	3	8	2	4	4
	1	5	5	0	5	0
ECEL1	14	5	13	0	10	4
	0	2	13	6	3	0
ECH1	140	1269	137	570	454	451
	997	707	247	236	628	159
ECHDC1	92	381	70	211	125	226
	227	350	130	87	177	52
ECHDC2	36	144	25	107	67	104
	160	207	73	42	93	37
ECHDC3	22	120	19	62	41	71
	121	200	65	39	67	24
ECHS1	122	751	119	473	209	384
	782	843	270	142	365	131
ECI1	37	202	32	151	68	98
	204	267	128	75	141	34
ECI2	216	1035	138	509	500	567
	1182	1314	426	331	690	201
ECM1	23	18	15	24	13	30
	15	36	28	12	18	12
ECM2	71	191	78	125	136	128
	199	212	107	56	106	87
ECSCR (NC_000005 138784244..138784863)				6	45	4
	16	8	19	30	50	23
	15	21	11			
ECSCR (NC_000005 138837128..138842320)				5	10	1
	3	6	7	9	12	2
	3	5	2			

ECSIT	39	247	36	127	66	129
	313	326	118	70	178	39
ECT2	42	71	35	51	31	56
	30	63	46	18	45	10
ECT2L	22	4	25	6	8	5
	0	6	21	0	6	1
EDA	34	30	21	24	27	20
	25	18	25	6	16	6
EDA2R	36	104	37	41	15	49
	17	29	31	6	16	6
EDAR	23	0	10	2	3	0
	0	0	15	0	5	0
EDARADD	13	1	2	1	4	0
	0	0	9	0	2	0
EDC3	39	114	27	71	47	29
	91	95	42	30	54	14
EDC4	66	238	62	193	123	201
	162	182	104	64	109	54
EDDM3A	6	0	1	0	0	0
	0	0	0	0	2	0
EDDM3B	8	0	3	0	0	0
	0	0	9	0	2	0
EDEM1	48	131	33	107	54	85
	87	164	87	31	88	26
EDEM2	19	28	10	19	12	14
	19	64	36	10	22	4
EDEM3	158	599	168	543	343	473
	512	682	365	179	402	144
EDF1	132	708	99	302	236	419
	592	951	256	193	296	89
EDIL3	31	16	20	10	10	4
	2	5	17	4	12	9
EDN1	16	8	9	8	7	15
	10	26	24	14	10	12
EDN2	0	0	3	0	1	1
	0	0	2	0	0	0
EDN3	10	9	7	1	5	8
	6	6	5	2	6	2
EDNRA	35	79	17	53	31	41
	53	76	42	14	35	13
EDNRB	38	59	39	26	40	33
	91	101	62	26	48	14
EEA1	319	1928	399	1374	961	1481
	1385	1544	675	527	850	301
EED	52	150	52	105	75	135
	150	186	67	59	102	32
EEF1A1	1127	8208	3495	6333	2943	7507
	5822	10346	4215	2318	4216	2126
EEF1A2	1070	6470	904	3369	2278	4244
	5926	6028	2508	2374	3109	1220
EEF1B2	894	4136	822	3832	1637	4413
	7774	9414	3138	1655	3100	923
EEF1D	642	3221	609	2432	1317	3068
	3664	5330	1988	1150	2174	527
EEF1E1	8	71	12	42	24	61
	43	118	46	16	50	7
EEF1G	1205	7807	1212	7306	3041	9445
	9251	10717	5489	2830	6030	1457

EEF2	1970	10051	2678	7296	4117	9568
	8317	12469	5345	3907	6024	2112
EEF2K	241	1065	189	823	568	865
	668	1018	489	337	498	212
EEFSEC	17	34	13	22	12	14
	26	29	12	8	21	5
EEPD1	56	177	47	149	86	146
	153	283	137	48	164	45
EFCAB1	28	2	13	1	3	0
	0	0	12	1	2	1
EFCAB11	11	20	3	10	8	12
	18	17	15	9	7	2
EFCAB12	11	0	9	0	2	0
	1	0	3	0	3	0
EFCAB13	51	63	37	54	40	38
	20	63	44	12	50	14
EFCAB2	83	179	47	173	65	113
	87	150	63	46	100	34
EFCAB3	17	2	17	3	10	0
	2	2	12	5	9	1
EFCAB4A	11	10	7	4	5	4
	4	6	4	3	4	4
EFCAB4B	46	20	22	15	15	13
	3	28	29	6	13	6
EFCAB5	63	7	26	10	25	5
	4	7	45	1	18	1
EFCAB6	54	30	35	25	20	22
	14	21	52	6	35	7
EFCAB7	22	129	79	80	27	69
	62	99	37	26	59	27
EFCAB8	32	5	15	1	5	2
	0	1	25	1	13	1
EFCAB9	8	0	2	0	0	0
	0	0	3	0	0	0
EFEMP1	52	166	97	246	192	244
	131	525	71	44	151	134
EFEMP2	22	76	33	30	41	38
	61	58	20	22	22	18
EFHA1	116	688	146	442	309	448
	602	625	304	190	363	88
EFHA2	56	119	42	167	75	95
	130	132	81	45	95	21
EFHB	29	22	30	17	12	6
	6	11	16	4	17	6
EFHC1	44	62	51	66	32	48
	37	79	52	15	43	15
EFHC2	36	1	5	5	12	4
	4	5	6	1	8	1
EFHD1	33	88	10	84	50	62
	67	69	41	27	36	13
EFHD2	9	26	5	15	9	3
	19	31	14	10	12	5
EFNA1	15	38	5	12	16	12
	37	32	16	12	13	3
EFNA2	2	0	2	0	2	0
	0	1	0	1	0	1
EFNA3	5	1	4	5	3	4
	4	0	4	0	1	0

EFNA4	4	1	3	5	2	4
	3	1	1	0	0	1
EFNA5	25	15	14	16	10	14
	4	18	9	9	10	6
EFNB1	21	35	17	44	23	29
	37	38	16	5	20	10
EFNB2	53	212	36	158	91	164
	198	164	148	55	150	28
EFNB3	9	0	11	2	3	1
	0	1	7	1	4	0
EFR3A	323	1243	258	989	798	878
	973	1392	553	416	615	239
EFR3B	70	155	28	70	89	115
	143	152	88	33	121	26
EFS	7	20	11	15	6	13
	9	9	13	4	12	0
EFTUD1	83	298	84	189	129	214
	247	296	119	92	181	60
EFTUD2	127	477	98	347	222	300
	388	450	220	155	254	62
EGF	423	1895	679	1442	741	1618
	1178	1828	1150	586	827	338
EGFL6	19	15	9	2	19	2
	0	0	3	0	2	0
EGFL7	27	129	13	63	34	44
	83	86	44	25	33	14
EGFL8	16	29	11	21	9	18
	23	36	10	3	18	3
EGFLAM	164	548	113	340	335	464
	615	641	337	187	296	91
EGFR	123	397	137	387	229	329
	258	463	207	136	216	124
EGLN1	640	2683	552	2067	1197	1761
	2428	4951	1337	927	2056	677
EGLN2	25	129	20	106	57	98
	100	167	45	40	53	19
EGLN3	23	61	26	21	25	52
	60	106	51	16	47	13
EGR1	13	136	30	143	46	109
	48	75	35	12	10	10
EGR2	10	8	12	9	4	1
	0	4	11	0	7	0
EGR3	17	13	15	24	5	6
	0	1	6	1	3	1
EGR4	0	0	0	0	0	0
	0	0	2	0	3	0
EHBP1	108	375	121	345	217	376
	183	343	149	121	188	88
EHBP1L1	373	2372	471	1263	877	1456
	1344	3142	910	868	1174	396
EHD1	37	97	25	76	57	94
	60	93	53	31	46	23
EHD2	39	255	36	123	95	110
	179	157	66	52	86	40
EHD3	37	35	15	40	13	37
	28	27	25	11	18	5
EHD4	22	47	12	21	24	25
	30	35	27	20	26	5

EHF	34	3	32	5	5	2
	1	0	15	0	10	2
EHHADH	25	48	12	23	18	18
	50	46	30	15	27	7
EHMT1	78	244	61	169	121	160
	190	256	119	83	166	63
EHMT2	30	69	36	52	37	62
	56	68	36	25	34	13
EI24	164	639	135	521	257	568
	575	702	324	146	341	94
EID1	301	1752	436	1807	773	1833
	2132	2269	1197	552	1268	310
EID2	8	54	15	28	16	54
	49	56	21	11	23	5
EID2B	10	17	5	19	7	19
	10	20	18	4	8	3
EIF1	303	2704	240	1870	777	1652
	2294	2499	736	512	988	330
EIF1AD	44	200	55	133	85	114
	185	221	89	67	94	39
EIF1AX	294	1252	237	936	769	892
	970	1258	577	460	684	183
EIF1AY	2	773	128	473	0	635
	768	885	309	1	402	79
EIF1B	39	316	37	230	104	115
	294	236	85	73	98	44
EIF2A	162	897	150	581	315	858
	758	1168	465	253	515	149
EIF2AK1	78	542	107	354	213	389
	416	405	198	146	267	70
EIF2AK2	77	398	89	231	198	240
	246	341	150	164	170	65
EIF2AK3	60	121	58	91	70	84
	115	197	125	51	125	41
EIF2AK4	124	406	107	293	225	330
	274	381	170	133	211	88
EIF2B1	58	251	51	158	138	169
	193	292	128	70	151	51
EIF2B2	41	155	48	141	76	131
	140	230	126	47	118	35
EIF2B3	118	662	115	436	268	417
	500	602	317	186	369	105
EIF2B4	46	161	22	104	73	111
	131	156	86	39	74	23
EIF2B5	83	408	81	326	191	295
	338	562	186	125	215	74
EIF2C1	91	403	112	377	198	284
	339	359	198	101	226	64
EIF2C2	75	401	63	247	197	289
	259	363	148	98	181	88
EIF2C3	75	265	88	229	130	163
	171	307	123	93	167	47
EIF2C4	153	585	137	484	325	406
	440	635	325	187	398	120
EIF2D	85	280	77	185	132	203
	211	328	156	93	183	51
EIF2S1	150	742	131	605	375	576
	580	807	335	190	478	109

EIF2S2	151	964	133	650	426	744
	813	997	375	293	502	184
EIF2S3	255	1193	214	742	680	786
	942	1127	460	394	533	183
EIF3A	372	2327	428	1648	1053	1554
	1622	2160	816	739	1154	469
EIF3B	142	693	140	461	284	468
	471	716	340	230	371	135
EIF3C	308	1380	249	696	596	771
	1256	625	454	480	529	197
EIF3CL	34	425	15	165	62	146
	216	132	137	99	179	100
EIF3D	101	545	115	309	209	383
	378	650	257	173	320	107
EIF3E	439	2595	534	1815	1094	2023
	2440	3630	1334	771	1479	354
EIF3F	137	676	104	596	250	673
	625	786	410	255	437	136
EIF3G	215	1415	277	908	490	1230
	1211	1978	867	371	883	205
EIF3H	169	1178	182	704	454	782
	1184	1213	571	340	554	174
EIF3I	98	551	87	300	210	302
	462	667	311	160	285	97
EIF3J	153	917	103	615	384	580
	795	946	261	266	463	155
EIF3K	182	941	120	686	384	682
	1141	1759	682	299	634	165
EIF3L	419	2136	585	2169	951	2567
	2255	2891	1469	873	1580	460
EIF3M	107	542	90	349	187	303
	434	607	274	136	309	94
EIF4A1	123	678	141	509	275	478
	506	887	313	167	369	111
EIF4A2	1181	6069	1186	5120	2886	5317
	5493	8487	3759	1883	4325	1030
EIF4A3	48	131	24	83	52	95
	118	129	67	39	78	23
EIF4B	1385	9641	1947	6673	4236	7006
	6801	10270	4053	3171	4643	1863
EIF4E	132	661	129	480	286	483
	429	664	299	187	393	82
EIF4E1B	8	1	8	0	3	0
	1	0	7	0	10	0
EIF4E2	103	594	137	420	221	449
	461	575	241	171	304	69
EIF4E3	110	839	98	577	307	488
	716	619	245	209	344	114
EIF4EBP1	11	45	7	46	21	36
	104	208	64	13	46	14
EIF4EBP2	309	1669	325	1587	890	1376
	1806	1798	900	445	951	336
EIF4EBP3	0	8	1	3	1	2
	10	29	9	0	14	4
EIF4ENIF1	58	146	34	99	72	108
	87	134	60	36	76	35
EIF4G1	660	3681	564	2389	1849	2428
	2408	2988	1141	1234	1647	673

EIF4G2	1354	7298	1386	4690	3793	5112
	5735	7140	2715	2454	3678	1127
EIF4G3	247	1626	258	1015	690	1176
	913	1404	424	496	574	219
EIF4H	322	1538	320	1073	780	1150
	1495	1864	836	493	934	377
EIF5	524	2886	497	2737	1411	2294
	2475	2960	1418	896	1646	394
EIF5A	86	555	85	473	248	441
	523	456	297	140	313	72
EIF5A2	35	32	20	27	20	24
	41	33	33	14	29	8
EIF5AL1	153	43	67	18	30	23
	35	64	66	7	62	11
EIF5B	151	1156	172	689	404	565
	760	722	278	264	401	150
EIF6	30	61	21	64	32	64
	71	91	39	14	39	11
ELAC1	15	49	23	29	21	24
	29	23	20	9	19	14
ELAC2	102	442	119	338	184	330
	312	457	206	133	279	61
ELANE	0	0	1	0	1	0
	0	0	1	0	0	0
ELAVL1	53	223	41	141	119	114
	144	176	75	44	87	34
ELAVL2	28	12	37	2	11	9
	2	2	10	0	10	0
ELAVL3	13	0	8	0	1	0
	0	2	7	0	4	0
ELAVL4	20	1	14	0	4	2
	0	1	15	0	7	0
ELF1	107	341	92	259	160	233
	364	526	188	96	219	65
ELF2	190	1181	275	783	540	727
	990	1256	490	380	649	229
ELF3	17	4	6	0	2	0
	0	0	10	0	2	0
ELF4	21	34	8	16	24	13
	15	15	13	7	13	4
ELF5	18	0	10	0	2	0
	0	0	5	0	3	0
ELFN1	8	2	5	0	1	1
	1	2	2	1	4	1
ELFN2	19	2	15	3	6	1
	0	0	8	0	9	0
ELK1	20	71	26	56	21	62
	59	72	35	18	32	19
ELK3	18	87	11	35	29	27
	59	50	23	22	26	13
ELK4	287	1381	265	1232	718	1131
	1107	1404	631	422	837	240
ELL	27	96	33	68	58	89
	99	128	65	44	67	29
ELL2	64	147	78	157	102	121
	274	391	131	91	178	91
ELL3	8	11	5	5	8	5
	4	10	8	0	6	2

ELMO1	69	108	47	105	69	71
	81	132	70	39	83	39
ELMO2	60	216	64	249	126	195
	157	204	102	48	112	45
ELMO3	7	4	6	4	0	5
	8	3	4	1	4	0
ELMOD1	35	16	17	22	18	25
	24	34	24	4	17	3
ELMOD2	74	230	59	202	99	168
	156	277	132	67	158	50
ELMOD3	49	98	42	63	63	59
	100	165	78	46	81	30
ELMSAN1	96	330	89	216	157	233
	212	305	134	66	132	49
ELN	33	41	21	30	12	19
	18	21	21	5	13	14
ELOF1	15	47	7	38	31	34
	37	62	40	17	24	4
ELOVL1	16	31	21	37	27	31
	36	47	41	9	32	8
ELOVL2	28	23	17	12	17	12
	10	31	21	7	15	4
ELOVL3	18	2	5	1	3	0
	0	0	2	0	1	0
ELOVL4	19	11	9	6	2	5
	4	4	9	1	13	0
ELOVL5	54	135	26	103	68	72
	94	108	65	38	92	49
ELOVL6	28	8	20	8	8	9
	2	6	17	5	15	3
ELOVL7	14	17	9	6	16	14
	3	14	22	2	16	3
ELP2	134	468	130	364	238	353
	417	822	346	207	410	121
ELP3	127	583	111	334	269	355
	371	440	250	135	253	98
ELP4	34	129	50	90	71	96
	106	178	83	39	114	22
ELP5	39	161	46	115	72	141
	178	218	84	51	117	24
ELP6	40	87	19	66	37	67
	50	91	57	22	53	22
ELSPBP1	8	0	6	0	2	0
	0	0	7	0	5	0
ELTD1	53	434	70	186	120	197
	308	417	195	134	183	58
EMB	36	50	25	53	68	93
	58	159	58	32	73	20
EMC1	172	417	130	327	206	359
	325	642	206	148	292	83
EMC10	34	121	24	88	41	77
	65	120	71	36	76	20
EMC2	81	447	84	287	214	260
	406	463	178	148	208	64
EMC3	147	530	100	563	217	452
	557	1490	390	170	403	84
EMC4	118	753	103	614	305	565
	970	1013	397	251	461	149

EMC6	22	172	18	98	72	106
	241	220	80	47	82	37
EMC7	28	133	20	107	49	117
	157	130	66	24	88	17
EMC8	16	77	9	58	33	33
	63	92	45	27	33	11
EMC9	12	48	5	25	26	27
	53	94	29	21	37	7
EMCN	82	467	69	285	200	200
	438	478	310	174	258	71
EMD	23	85	17	69	25	64
	80	109	52	26	45	19
EME1	16	3	11	4	1	4
	6	8	10	0	6	2
EME2	0	10	5	4	1	10
	1	16	5	1	5	1
EMG1	25	92	17	58	40	62
	76	143	66	22	49	13
EMID1	16	5	3	1	7	0
	0	0	2	1	1	0
EMID2	6	0	5	0	1	0
	0	0	3	0	0	0
EMILIN1	18	57	11	50	26	29
	28	52	39	13	24	12
EMILIN2	24	48	13	62	36	32
	16	83	23	10	22	21
EMILIN3	13	6	5	12	3	1
	18	5	9	2	3	4
EML1	156	713	137	639	348	511
	470	486	314	199	366	113
EML2	11	27	10	8	11	5
	8	12	14	4	17	6
EML3	29	41	21	23	13	16
	31	32	29	12	26	7
EML4	68	185	64	134	84	114
	124	156	101	64	91	35
EML5	59	18	36	14	22	31
	14	14	61	13	19	3
EML6	91	20	49	42	54	30
	16	25	47	6	34	5
EMP1	62	269	66	325	186	190
	179	353	125	65	116	131
EMP2	70	252	60	144	130	148
	185	277	112	77	140	50
EMP3	10	31	13	40	19	42
	23	53	35	10	17	5
EMR1	26	1	16	0	7	1
	0	0	12	0	9	0
EMR2	57	13	28	9	16	12
	14	10	26	4	19	4
EMR3	18	0	13	3	6	4
	0	0	9	0	4	0
EMX1	3	0	3	0	0	0
	0	0	1	0	0	1
EMX2	17	17	6	2	3	8
	7	5	5	2	7	5
EN1	25	102	13	67	39	76
	43	20	23	9	29	9

EN2	12	1	1	1	2	0
	2	0	2	1	1	0
ENAH	257	1215	221	567	529	655
	548	1634	523	429	527	253
ENAM	76	270	66	86	126	96
	134	199	97	81	152	61
ENC1	57	118	35	93	82	161
	57	169	97	43	64	24
ENDOD1	410	2060	403	1366	932	1670
	1174	2243	943	529	1021	394
ENDOG	30	250	26	156	94	106
	336	330	146	70	154	30
ENDOU	15	3	11	5	8	1
	4	1	12	0	5	0
ENDOV	32	34	14	22	17	31
	31	67	25	7	32	4
ENG	37	147	23	98	58	71
	129	153	48	41	58	41
ENGASE	78	329	51	136	115	202
	226	351	121	60	100	33
ENHO	2	0	1	0	0	0
	0	0	2	0	1	1
ENKD1	4	10	10	5	4	14
	8	15	7	3	9	3
ENKUR	18	1	8	0	7	1
	0	0	12	0	9	0
ENO1	111	508	131	420	212	365
	405	591	314	143	293	96
ENO2	21	22	17	24	13	18
	10	19	18	2	11	5
ENO3	4341	27665	4223	21756	10292	22762
	33047	23000	14379	8998	15884	3665
ENO4	19	0	12	0	3	0
	0	5	10	2	6	0
ENOPH1	27	97	45	94	37	108
	135	144	56	35	82	18
ENOSF1	107	227	35	186	72	92
	183	280	184	139	230	50
ENOX1	28	42	38	22	31	43
	22	33	27	14	32	9
ENOX2	57	105	56	89	91	98
	110	156	65	37	61	29
ENPEP	95	282	95	189	147	115
	212	367	193	110	285	72
ENPP1	77	146	46	143	190	187
	102	213	106	44	104	39
ENPP2	63	122	40	64	41	64
	97	96	82	34	58	35
ENPP3	39	6	22	13	14	14
	9	22	30	4	17	2
ENPP4	203	763	162	640	368	691
	597	838	320	257	424	125
ENPP5	68	278	84	157	115	140
	311	242	63	63	127	66
ENPP6	32	3	11	15	8	5
	1	5	13	1	9	3
ENPP7	6	0	5	1	4	0
	0	0	4	0	2	0

ENSA	409	1953	314	1373	1002	1450
	1561	1857	696	626	909	295
ENTHD1	18	0	11	0	3	1
	0	0	7	0	7	0
ENTHD2	10	15	5	12	7	11
	10	20	6	3	15	3
ENTPD1	150	201	68	149	94	116
	109	190	129	48	102	31
ENTPD2	5	1	4	2	2	2
	0	2	2	0	5	0
ENTPD3	24	12	14	7	8	6
	10	10	14	4	10	2
ENTPD4	92	355	82	274	192	284
	278	430	212	74	198	90
ENTPD5	41	98	19	66	45	71
	71	73	54	28	71	19
ENTPD6	30	93	27	67	35	68
	72	141	54	33	55	16
ENTPD7	60	33	35	30	30	22
	29	34	32	14	25	8
ENTPD8	6	0	1	0	0	0
	0	0	0	0	3	0
ENY2	111	503	96	304	214	348
	475	615	230	156	331	70
EOGT	71	225	67	258	131	230
	201	405	164	95	184	87
EOMES	16	2	9	0	5	2
	1	2	9	0	5	1
EP300	214	1064	235	766	461	700
	888	839	314	299	496	209
EP400	179	559	157	383	222	350
	384	482	185	160	220	110
EPAS1	158	904	115	481	436	402
	1049	1018	456	363	652	221
EPB41	186	733	143	453	299	502
	401	501	237	175	317	88
EPB41L1	40	73	31	57	45	42
	54	68	39	17	50	10
EPB41L2	148	637	133	425	338	465
	638	650	306	267	366	145
EPB41L3	69	296	137	120	106	170
	82	159	152	47	136	55
EPB41L4A	69	134	66	113	74	76
	103	99	79	47	91	19
EPB41L4B	69	36	46	18	14	30
	43	112	47	16	39	21
EPB41L5	200	497	189	325	347	401
	444	766	306	142	317	132
EPB42	16	4	10	1	2	1
	3	3	20	0	4	1
EPB49	25	103	29	57	32	90
	91	67	42	27	58	22
EPC1	115	524	143	357	255	431
	367	592	256	198	414	109
EPC2	73	378	121	297	202	287
	362	379	181	132	229	70
EPCAM	10	2	4	2	4	4
	0	0	5	1	9	0

EPDR1	299	1418	270	1081	593	1014
	1087	927	542	327	543	169
EPG5	248	776	229	645	366	610
	571	867	461	270	576	160
EPGN	33	2	8	1	11	3
	0	5	9	0	13	2
EPHA1	19	2	6	2	1	1
	0	1	6	0	1	0
EPHA10	39	0	17	0	7	1
	0	0	12	0	7	0
EPHA2	15	38	10	26	20	14
	23	22	17	8	20	7
EPHA3	69	99	38	134	50	65
	39	82	70	16	46	30
EPHA4	77	199	82	193	134	208
	147	153	106	49	96	25
EPHA5	70	2	37	5	19	0
	0	0	45	0	15	3
EPHA6	43	2	42	0	23	4
	0	2	25	0	19	1
EPHA7	38	18	43	27	71	18
	9	14	48	6	30	6
EPHA8	12	0	9	0	2	0
	0	0	16	0	0	0
EPHB1	46	122	41	62	49	56
	65	84	54	19	70	10
EPHB2	35	10	20	19	9	4
	6	0	16	2	8	1
EPHB3	8	9	8	2	5	2
	0	1	7	0	6	1
EPHB4	20	74	11	51	19	22
	58	54	36	12	35	13
EPHB6	17	9	14	8	14	7
	5	12	9	4	12	7
EPHX1	24	141	21	78	77	49
	72	78	28	17	43	18
EPHX2	63	133	45	86	47	73
	102	230	84	28	129	17
EPHX3	6	0	0	0	1	1
	0	0	6	1	0	0
EPHX4	6	5	3	0	1	1
	1	3	5	0	4	0
EPM2A	155	876	116	494	435	560
	666	898	393	273	493	145
EPM2AIP1	158	904	204	812	418	719
	670	842	419	244	551	129
EPN1	45	240	39	121	60	150
	187	170	88	66	110	44
EPN2	63	225	63	140	118	184
	200	223	116	73	113	44
EPN3	4	0	5	5	5	0
	0	0	5	0	4	0
EPO	14	0	4	0	0	0
	0	1	2	1	0	0
EPOR	8	6	3	4	5	4
	6	6	5	3	6	2
EPPIN	0	0	0	0	0	0
	0	0	0	0	2	0

EPPIN-WFDC6	7	0	6	0	5	0	0	1	3	0	1	0
EPPK1	18	6	11	2	2	3	2	1	8	1	2	1
EPRS	314	1949	404	1250	852	1399	1271	1637	746	573	1063	383
EPS15	480	2859	471	1911	1241	1952	2132	2414	1109	773	1488	415
EPS15L1	66	213	65	119	115	162	290	196	98	117	117	47
EPS8	92	422	84	322	187	244	312	410	207	129	238	95
EPS8L1	14	2	13	1	7	2	3	1	5	0	12	0
EPS8L2	13	4	8	15	2	1	1	0	3	0	4	1
EPS8L3	12	1	11	0	3	0	0	0	12	0	4	0
EPSTI1	35	31	12	30	33	27	30	35	33	30	31	6
EPT1	108	331	130	288	152	310	278	465	196	108	226	66
EPX	14	0	2	0	0	0	0	0	4	0	1	0
EPYC	9	0	12	0	3	0	0	0	5	0	3	0
EQTN	12	3	6	2	2	0	2	1	7	0	2	0
ERAL1	31	138	17	84	58	72	151	113	60	30	67	13
ERAP1	158	610	162	413	285	581	258	512	321	195	363	126
ERAP2	89	214	93	130	173	68	163	202	143	28	264	14
ERAS	1	0	0	0	0	0	0	0	0	0	0	0
ERBB2	67	162	67	149	118	156	96	167	106	48	88	42
ERBB2IP	522	2302	683	1742	1294	1524	1689	2577	1094	711	1297	599
ERBB3	64	114	178	56	13	22	10	15	30	1	13	16
ERBB4	195	371	175	442	224	334	386	426	219	169	232	84
ERC1	163	631	139	432	273	386	399	504	227	187	263	112
ERC2	43	1	33	3	11	1	0	1	25	2	8	0
ERCC1	48	190	35	136	63	156	165	250	115	63	115	29
ERCC2	27	39	20	22	17	32	25	41	21	18	26	11
ERCC3	68	230	48	174	107	143	224	245	120	81	128	46
ERCC4	86	199	68	187	134	222	138	256	150	77	152	42

ERCC5	12	142	31	99	33	129
	132	232	58	47	111	45
ERCC6	95	352	111	245	171	252
	233	415	206	98	247	62
ERCC6L	24	1	12	0	15	3
	0	3	3	0	2	0
ERCC6L2	39	177	55	134	97	106
	146	204	113	71	117	45
ERCC8	32	94	37	54	31	62
	38	105	52	21	60	13
EREG	11	1	10	0	4	1
	0	1	15	0	7	0
ERF	9	31	4	10	11	10
	17	26	12	5	8	5
ERG	76	139	36	74	66	52
	133	157	114	56	91	29
ERGIC1	134	792	139	509	367	700
	633	831	369	184	438	160
ERGIC2	94	448	92	320	217	277
	290	454	201	115	280	63
ERGIC3	53	183	35	193	79	166
	183	229	125	44	97	36
ERH	52	255	42	173	121	126
	250	319	127	56	146	53
ERI1	37	95	29	51	44	53
	53	120	51	31	57	7
ERI2	64	173	49	112	80	138
	143	219	80	42	111	36
ERI3	41	158	36	132	72	105
	140	190	92	35	93	30
ERICH1	28	64	14	40	34	39
	40	64	51	25	28	9
ERICH2	5	2	6	0	3	0
	1	3	4	1	3	1
ERLEC1	71	368	68	285	180	304
	308	363	154	120	217	60
ERLIN1	47	120	36	78	63	76
	107	126	56	30	69	19
ERLIN2	99	334	100	202	188	240
	237	362	188	100	169	66
ERMAP	31	33	27	29	35	46
	53	68	34	15	40	6
ERMN	31	1	11	1	6	2
	0	0	14	1	6	1
ERMP1	118	521	140	411	242	400
	357	584	272	136	262	84
ERN1	33	38	34	27	36	41
	33	55	41	14	32	15
ERN2	26	1	8	0	8	0
	2	1	4	0	11	0
ERO1L	33	154	30	142	85	130
	129	128	81	40	96	39
ERO1LB	44	64	41	75	46	46
	64	100	64	23	58	20
ERP27	16	4	8	4	9	1
	7	5	13	4	9	1
ERP29	52	207	27	74	100	181
	134	264	76	57	64	29

ERP44	74	368	84	234	163	249
	260	372	141	90	182	64
ERRFI1	43	131	77	104	50	84
	106	184	68	40	107	40
ERV3-1	71	73	32	76	88	94
	56	54	41	26	64	42
ERVFRD-1	58	2	20	1	9	3
	3	2	20	2	13	0
ERVMER34-1	15	1	5	0	2	1
	0	0	4	1	2	0
ERVV-1	21	0	6	0	1	0
	0	0	7	0	2	0
ERVV-2	17	0	13	0	2	0
	0	0	5	0	4	0
ERVW-1	393	28	182	26	97	32
	8	31	141	1	76	7
ESAM	20	76	16	31	29	34
	57	44	38	14	34	7
ESCO1	169	747	216	506	414	745
	578	681	329	296	435	134
ESCO2	26	2	12	1	8	0
	0	1	9	0	8	0
ESD	88	320	46	244	177	252
	316	427	192	105	222	63
ESF1	78	518	114	227	270	284
	298	381	143	156	226	85
ESM1	5	4	7	2	4	0
	5	0	12	2	7	3
ESPL1	29	0	14	2	6	1
	0	1	23	1	4	1
ESPN	8	0	9	0	0	0
	0	0	3	0	3	0
ESPNL	6	2	4	3	2	1
	2	0	4	1	3	1
ESR1	67	241	85	119	142	210
	243	233	164	81	103	34
ESR2	14	3	10	6	4	2
	1	8	5	1	12	0
ESRP1	26	0	14	0	4	1
	0	0	16	0	7	0
ESRP2	17	0	14	2	3	5
	0	2	15	2	6	1
ESRRA	104	484	49	322	157	318
	426	588	237	156	301	71
ESRRB	27	32	28	12	16	20
	36	42	16	15	33	5
ESRRG	133	233	94	132	175	117
	232	513	95	149	203	90
ESX1	8	0	2	0	0	0
	0	0	1	0	0	0
ESYT1	140	716	100	469	212	498
	378	449	231	140	261	76
ESYT2	234	1055	282	863	529	748
	696	1333	503	339	606	269
ESYT3	39	17	25	22	17	18
	9	7	21	10	17	5
ETAA1	61	219	71	164	113	153
	192	239	145	74	155	44

ETF1	131	793	136	580	334	570
	631	709	346	196	376	158
ETFA	311	1763	213	1053	715	916
	1531	2206	664	393	880	250
ETFB	101	576	66	296	164	238
	697	818	239	114	323	67
ETFDH	334	2208	338	1180	925	1098
	1697	2371	764	512	1309	283
ETHE1	8	18	7	11	6	18
	22	38	7	10	12	5
ETNK1	142	423	140	415	224	378
	348	671	309	144	331	86
ETNK2	6	2	9	3	2	0
	2	5	4	3	6	0
ETS1	101	618	85	344	216	254
	442	418	241	134	223	74
ETS2	85	518	120	361	193	331
	311	336	131	125	177	106
ETV1	55	32	28	30	27	37
	27	16	51	9	26	6
ETV2	3	0	1	0	0	0
	0	0	1	0	0	0
ETV3	29	81	20	48	42	34
	65	64	21	12	43	10
ETV3L	9	0	2	0	1	0
	0	0	1	0	0	0
ETV4	18	0	15	5	5	0
	8	1	5	2	15	0
ETV5	51	28	32	80	27	57
	35	65	37	19	41	4
ETV6	63	143	39	97	59	106
	157	116	61	35	47	24
ETV7	12	1	6	1	1	3
	1	2	8	0	1	1
EVC	49	64	39	75	45	43
	30	57	43	24	33	23
EVC2	47	25	27	67	22	39
	28	50	32	10	27	8
EVI2A	21	9	16	12	9	33
	11	37	14	9	16	2
EVI2B	17	25	9	13	12	21
	21	29	14	9	5	4
EVI5	108	288	89	288	161	280
	188	279	171	81	152	80
EVI5L	27	96	26	46	30	48
	46	64	37	13	49	19
EVL	22	64	10	46	17	62
	68	74	29	23	42	10
EVPL	18	5	11	2	4	7
	5	7	14	0	5	1
EVPLL	16	0	11	0	2	0
	0	0	4	0	3	0
EVX1	0	0	2	0	3	0
	0	0	2	0	1	0
EVX2	4	0	0	0	0	0
	0	0	0	0	0	0
EWSR1	193	937	234	688	467	735
	916	1129	472	383	676	187

EXD1	22	0	6	0	2	0
	0	1	11	0	7	0
EXD2	84	272	87	232	148	253
	261	300	192	71	178	38
EXD3	11	29	8	17	3	27
	20	25	4	9	15	6
EXO1	25	2	11	2	6	0
	1	0	14	0	9	1
EXOC1	87	367	101	312	169	252
	324	418	249	113	251	85
EXOC2	83	250	85	181	135	199
	158	282	145	70	131	43
EXOC3	77	344	71	252	164	284
	279	338	162	114	237	61
EXOC3L1	12	11	5	10	8	2
	5	9	7	2	4	0
EXOC3L2	9	5	4	0	1	0
	3	3	7	0	2	1
EXOC3L4	3	0	3	3	2	0
	0	0	3	0	4	0
EXOC4	267	1026	297	909	618	742
	792	1186	504	428	858	218
EXOC5	168	795	189	606	407	532
	495	698	335	183	425	138
EXOC6	244	972	237	718	473	668
	829	1634	551	341	773	225
EXOC6B	92	332	101	271	158	200
	304	345	143	128	163	58
EXOC7	172	647	146	415	217	427
	438	643	309	196	392	117
EXOC8	90	326	78	276	223	177
	335	499	128	113	265	90
EXOG	33	91	32	61	45	54
	43	121	38	22	57	23
EXOSC1	19	58	8	44	18	32
	57	88	36	22	46	11
EXOSC10	144	590	159	416	258	513
	471	842	304	237	449	108
EXOSC2	47	133	32	67	57	62
	88	158	69	32	86	24
EXOSC3	25	113	25	95	64	102
	89	134	64	38	67	12
EXOSC4	3	13	1	10	5	11
	14	29	10	1	7	3
EXOSC5	8	29	7	17	11	29
	18	49	16	5	22	6
EXOSC6	28	145	16	101	53	103
	79	96	63	26	34	19
EXOSC7	45	151	31	106	62	119
	147	339	98	43	83	32
EXOSC8	53	211	40	100	79	133
	162	280	117	58	113	27
EXOSC9	56	215	79	163	106	177
	182	235	107	92	173	44
EXPH5	72	10	45	11	10	10
	8	8	44	1	22	2
EXT1	43	111	28	71	54	63
	87	119	70	28	61	24

EXT2	88	246	68	220	112	194
	191	318	146	87	164	35
EXTL1	20	16	8	17	8	28
	31	13	12	7	28	8
EXTL2	54	129	31	131	78	128
	131	193	80	42	84	34
EXTL3	85	292	38	239	148	226
	214	213	123	59	118	44
EYA1	35	142	82	169	57	97
	167	68	137	43	201	30
EYA2	27	15	19	31	8	20
	12	23	14	3	12	5
EYA3	97	350	89	204	161	217
	235	360	147	101	174	66
EYA4	225	879	278	788	620	744
	909	1062	620	419	601	207
EYS	89	8	76	12	35	7
	5	10	59	1	47	1
EZH1	175	619	148	404	289	411
	640	815	311	224	451	158
EZH2	41	47	23	24	31	27
	30	80	35	14	44	11
EZR	54	163	95	122	95	198
	157	155	85	48	91	38
F10	12	23	7	16	20	31
	12	20	6	4	5	4
F11	25	27	24	28	6	14
	27	26	37	7	11	2
F11R	44	97	21	65	49	48
	59	72	56	20	42	22
F12	7	0	1	1	0	0
	0	1	4	0	2	0
F13A1	50	118	84	129	186	256
	74	606	97	61	128	84
F13B	18	0	9	0	5	0
	0	0	12	0	2	0
F2	14	0	10	0	1	0
	0	0	7	0	5	0
F2R	26	62	9	36	19	18
	35	51	21	13	30	9
F2RL1	17	1	5	1	1	1
	3	1	5	0	6	0
F2RL2	17	0	8	0	3	0
	2	0	10	0	2	0
F2RL3	13	1	4	3	1	0
	2	5	2	0	2	2
F3	22	59	13	69	42	27
	78	126	44	16	56	38
F5	105	14	52	3	19	9
	2	15	51	2	32	4
F7	16	0	13	0	2	0
	0	0	8	1	3	0
F8	214	879	144	554	426	567
	992	1061	579	315	666	215
F8A1	11	38	14	20	29	55
	46	21	34	12	25	11
F8A2	0	1	0	1	0	0
	1	0	0	0	0	0

F8A3	0	1	0	0	0	0
	0	0	0	0	0	0
F9	20	0	5	0	7	0
	0	0	6	0	1	0
FA2H	10	0	3	0	2	0
	0	0	2	0	5	1
FAAH	12	66	26	75	29	65
	96	137	53	27	56	14
FAAH2	19	4	3	6	2	5
	5	2	6	0	3	2
FABP1	3	0	1	1	0	0
	0	0	0	0	2	0
FABP12	2	0	3	0	0	0
	0	0	1	0	0	0
FABP2	9	2	4	5	1	0
	0	0	5	0	4	0
FABP3	1129	7903	499	1972	2376	3098
	6661	9411	1598	1114	2082	556
FABP4	75	572	64	362	209	319
	633	931	324	135	247	199
FABP5	71	290	31	123	128	177
	459	437	170	109	155	70
FABP6	7	1	6	2	0	0
	1	0	3	0	1	0
FABP7	16	116	8	4	11	2
	3	18	11	2	6	1
FABP9	5	0	3	1	0	0
	0	0	4	0	1	0
FADD	13	49	9	46	21	34
	29	35	26	12	28	4
FADS1	25	46	15	49	35	47
	43	49	35	15	25	17
FADS2	27	75	22	53	33	51
	34	77	46	11	26	14
FADS3	18	75	35	43	38	65
	80	111	61	19	46	23
FADS6	10	0	5	1	2	0
	0	0	5	0	2	0
FAF1	133	857	172	586	321	584
	691	836	408	265	487	148
FAF2	96	337	104	234	178	289
	286	429	179	111	239	65
FAH	23	84	18	62	37	46
	75	99	54	22	41	21
FAHD1	41	209	31	97	57	112
	169	210	68	61	118	17
FAHD2A	50	180	32	130	85	107
	116	199	85	41	107	29
FAHD2B	41	105	23	75	47	65
	57	130	45	39	69	17
FAIM	27	61	23	33	38	29
	75	80	40	28	69	15
FAIM2	23	20	14	10	4	7
	5	3	11	0	8	0
FAIM3	27	24	16	2	9	4
	2	8	11	1	7	1
FAM100A	6	13	1	7	10	12
	16	18	4	4	9	7

FAM100B	16	100	6	41	49	56
	95	134	55	27	34	14
FAM101B	24	93	10	57	47	50
	92	109	45	38	53	14
FAM102A	12	23	10	28	9	14
	13	16	11	6	14	4
FAM102B	51	100	20	69	50	78
	55	80	55	24	32	20
FAM103A1	11	38	14	71	38	39
	57	105	51	18	39	15
FAM104A	48	197	47	128	89	100
	161	181	88	44	95	25
FAM104B	56	114	33	51	56	87
	128	181	75	25	59	10
FAM105A	44	29	21	18	25	30
	19	44	35	10	24	7
FAM105B	97	271	103	237	159	286
	261	407	153	86	195	87
FAM107A	22	69	19	55	26	21
	92	137	38	36	54	20
FAM107B	20	54	15	37	31	34
	19	25	31	3	21	24
FAM108A1	30	110	17	73	48	87
	104	133	80	32	61	15
FAM108B1	46	173	46	133	73	158
	154	219	117	59	110	22
FAM108C1	6	9	4	5	1	2
	3	2	2	1	3	2
FAM109A	10	19	10	11	7	16
	21	26	20	8	19	2
FAM109B	14	11	4	11	5	14
	9	21	15	0	8	3
FAM110A	7	6	6	2	7	2
	1	14	4	1	2	2
FAM110B	44	104	22	72	52	76
	92	74	49	29	61	17
FAM110C	15	1	8	1	2	1
	0	0	6	0	1	0
FAM110D	3	5	2	3	2	3
	8	4	11	3	6	4
FAM111A	51	110	40	108	66	115
	96	125	70	50	71	20
FAM111B	31	1	19	3	12	5
	0	3	22	4	12	0
FAM114A1	77	289	63	138	174	192
	173	285	148	105	137	57
FAM114A2	61	183	63	146	90	129
	130	173	112	56	114	34
FAM115A	184	457	196	348	291	373
	319	491	274	203	294	106
FAM115C	29	15	29	7	6	6
	4	11	17	2	11	4
FAM117A	10	41	6	15	10	18
	14	20	20	9	11	6
FAM117B	54	89	32	88	47	46
	97	120	94	28	63	26
FAM118A	64	171	38	108	81	89
	213	180	99	46	90	44

FAM118B	56	231	57	178	114	191	119	193	76	69	103	37
FAM120A	178	1004	203	566	421	668	673	879	325	296	436	196
FAM120AOS	50	146	33	109	60	88	128	133	81	44	90	24
FAM120B	55	335	84	211	153	170	228	280	108	97	172	82
FAM120C	68	118	47	153	54	111	87	102	73	41	73	23
FAM122A	0	0	0	0	0	0	0	1	0	0	0	0
FAM122B	34	110	27	96	49	50	71	115	43	25	53	19
FAM122C	30	18	16	25	16	14	7	22	27	2	17	3
FAM123A	12	0	6	0	2	1	0	1	5	0	0	0
FAM123B	61	131	28	106	97	111	127	153	71	47	75	39
FAM123C	26	0	12	0	3	0	0	0	13	0	7	0
FAM124A	26	8	13	7	7	3	8	22	20	6	22	3
FAM124B	18	16	10	15	2	11	6	14	10	2	16	2
FAM125A	8	22	3	16	7	18	14	19	12	2	9	4
FAM125B	60	304	57	191	144	203	150	194	101	56	114	40
FAM126A	262	821	279	704	499	680	484	922	412	239	597	184
FAM126B	122	452	141	339	252	277	348	484	248	146	262	104
FAM127A	32	203	40	108	84	202	176	215	97	66	135	42
FAM127B	6	6	2	10	8	14	11	5	0	3	8	2
FAM127C	8	25	5	18	10	17	16	16	4	6	10	2
FAM129A	337	2997	537	1822	1131	2363	2183	1298	1112	621	1930	411
FAM129B	26	68	16	43	33	51	57	49	57	22	32	21
FAM129C	16	6	4	2	0	0	0	1	4	0	4	0
FAM131A	13	24	12	18	12	23	29	27	16	5	16	6
FAM131B	18	10	16	6	6	7	5	16	12	1	7	1
FAM131C	3	0	7	0	2	0	0	0	2	0	0	0
FAM132A	0	0	0	0	0	0	1	0	0	1	0	0
FAM132B	1	1	3	4	1	0	2	0	1	0	2	0
FAM133A	14	1	7	0	8	0	0	0	4	0	4	1

FAM133B	77	251	61	258	135	224
	296	308	138	84	168	47
FAM134A	66	115	48	113	83	99
	109	174	82	31	72	26
FAM134B	444	2887	567	2057	812	1550
	2969	3856	1645	829	2335	425
FAM134C	103	370	93	269	184	279
	303	476	203	97	242	100
FAM135A	76	292	100	283	149	270
	259	353	172	86	230	74
FAM135B	74	11	35	12	18	3
	5	6	29	0	10	1
FAM136A	44	237	48	108	77	131
	166	200	68	38	74	27
FAM13A	148	488	184	371	301	300
	405	482	229	160	287	98
FAM13B	109	437	155	312	171	321
	354	447	208	102	261	68
FAM13C	175	642	323	496	237	377
	448	503	316	131	348	148
FAM149A	21	110	17	58	31	54
	66	39	49	20	27	5
FAM149B1	58	195	60	135	98	134
	145	203	105	59	123	25
FAM150A	3	0	7	0	3	0
	0	0	1	0	1	1
FAM150B	7	1	4	1	0	4
	1	0	2	0	0	1
FAM151A	11	0	2	0	0	0
	0	0	4	0	1	0
FAM151B	10	8	5	3	8	5
	7	7	10	1	3	0
FAM153A	8	2	12	1	4	7
	4	7	8	1	2	0
FAM153B	30	8	10	0	7	9
	4	1	15	0	12	0
FAM154A	9	1	8	0	6	1
	1	1	6	0	1	0
FAM154B	18	7	21	5	7	6
	4	6	14	1	21	0
FAM155A	33	4	9	2	2	0
	4	3	14	0	2	0
FAM155B	25	1	6	0	2	0
	0	0	0	0	1	0
FAM156A	69	180	40	131	75	101
	121	179	59	46	91	24
FAM156B	1	1	1	2	0	0
	2	2	1	0	0	3
FAM157A	35	17	19	11	17	13
	9	17	18	7	8	1
FAM157B	24	6	5	2	11	1
	4	1	19	0	10	1
FAM159A	7	0	0	1	1	1
	2	1	1	1	1	0
FAM159B	3	0	1	1	1	0
	0	0	2	0	2	0
FAM160A1	201	790	182	478	403	534
	758	1300	381	312	547	192

FAM160A2	19	46	29	33	34	42
	60	76	29	17	23	20
FAM160B1	147	452	101	453	224	365
	422	579	374	153	360	107
FAM160B2	25	74	18	48	29	51
	49	57	36	21	44	14
FAM161A	32	31	20	25	17	49
	54	36	28	25	31	10
FAM161B	36	78	27	59	47	84
	43	86	33	27	54	13
FAM162A	89	435	59	339	153	221
	554	526	216	90	305	48
FAM162B	8	23	2	10	14	8
	17	34	10	3	8	3
FAM163A	12	4	3	1	0	2
	0	0	9	0	2	1
FAM163B	4	0	2	1	0	0
	0	0	2	0	0	0
FAM165B	35	148	21	111	64	106
	163	165	78	49	74	20
FAM166A	3	0	3	0	1	0
	0	1	2	0	2	0
FAM166B	79	373	37	302	204	273
	260	368	126	91	184	49
FAM167A	12	0	8	0	0	0
	2	0	6	0	1	0
FAM167B	2	2	2	2	0	0
	1	3	2	0	0	0
FAM168A	30	82	24	63	58	60
	63	70	39	31	54	16
FAM168B	172	790	153	753	392	700
	667	695	378	192	480	142
FAM169A	33	3	25	5	14	3
	0	2	31	1	21	0
FAM169B	30	0	12	0	10	0
	0	0	12	0	10	0
FAM170A	6	1	4	2	1	0
	0	0	3	1	3	0
FAM170B	4	1	2	0	1	0
	0	0	1	0	1	1
FAM171A1	22	46	10	51	31	30
	28	16	26	7	20	16
FAM171A2	2	0	0	0	0	1
	1	0	1	0	0	0
FAM171B	20	34	13	29	27	24
	15	31	22	10	21	11
FAM172A	39	215	58	149	89	136
	128	230	107	53	111	40
FAM173A	2	16	4	21	10	9
	21	49	14	5	9	1
FAM173B	35	149	47	88	83	113
	88	158	70	35	67	20
FAM174A	27	94	18	64	43	85
	81	124	53	31	65	20
FAM174B	9	48	25	32	27	39
	28	53	41	13	16	14
FAM175A	65	208	77	172	136	190
	237	304	105	94	176	52

FAM175B	79	368	77	260	183	316
	312	339	160	121	198	73
FAM176A	15	7	10	3	2	1
	6	1	4	1	2	0
FAM176B	3	11	1	7	4	12
	2	13	5	2	4	2
FAM176C	29	21	13	20	15	18
	27	31	21	6	16	9
FAM177A1	67	244	68	252	100	201
	148	259	198	68	153	32
FAM177B	16	2	5	2	4	1
	0	1	8	0	3	0
FAM178A	283	1078	350	902	652	950
	780	1329	609	427	577	222
FAM178B	9	0	8	0	4	1
	0	0	7	0	5	0
FAM179A	23	12	12	7	10	11
	43	40	25	35	17	10
FAM179B	178	613	131	551	341	475
	542	711	319	204	511	136
FAM180A	16	4	8	4	2	4
	2	8	9	0	2	2
FAM180B	5	11	10	12	11	17
	3	15	3	0	7	3
FAM181A	8	1	3	0	1	4
	1	0	4	0	1	1
FAM181B	0	1	0	1	1	0
	0	0	0	0	1	0
FAM183A	6	0	2	0	1	0
	0	0	3	0	1	0
FAM184A	38	39	41	27	25	16
	46	52	33	15	30	8
FAM184B	44	212	112	161	47	134
	34	49	25	4	26	25
FAM185A	50	230	56	139	93	156
	138	255	113	60	121	28
FAM186A	47	1	28	2	13	1
	0	8	24	0	13	0
FAM186B	24	6	11	11	5	3
	2	14	11	2	4	1
FAM187B	3	1	1	1	0	0
	0	0	2	0	0	0
FAM188A	68	199	64	179	111	154
	176	319	121	64	129	45
FAM188B	16	32	26	51	20	17
	29	16	28	7	24	9
FAM189A1	15	1	13	1	3	2
	0	0	5	0	4	0
FAM189A2	159	645	140	455	295	566
	400	451	282	193	316	68
FAM189B	14	19	20	16	14	15
	20	12	9	4	9	4
FAM18A	18	4	6	1	3	4
	4	2	6	2	8	1
FAM18B1	44	222	45	199	115	151
	119	300	153	87	114	48
FAM18B2	2	9	1	11	9	10
	19	33	8	4	25	7

FAM18B2-CDRT4	29	32	16	32	21	40	36	36	23	9	23	8
FAM190A	66	25	33	15	48	32	4	25	36	8	26	2
FAM190B	408	2111	370	1695	890	1725	1756	2165	944	661	1081	363
FAM192A	94	605	102	356	225	373	497	584	231	131	328	107
FAM193A	91	395	102	224	151	227	236	290	127	113	181	59
FAM193B	41	156	42	127	59	114	96	119	68	42	57	24
FAM194A	10	0	12	2	5	0	1	1	11	0	6	0
FAM194B	47	0	36	0	19	0	0	0	17	0	11	0
FAM195A	30	111	16	100	61	115	140	221	75	34	86	23
FAM195B	7	32	5	19	5	19	17	41	13	6	16	4
FAM196A	34	2	21	0	7	3	1	2	19	1	9	1
FAM196B	16	0	19	0	1	0	0	0	12	0	6	0
FAM197Y1	1	0	23	0	0	0	0	0	26	0	8	0
FAM197Y3	0	0	1	0	0	0	0	0	2	0	1	0
FAM197Y4	0	0	2	0	0	0	0	0	0	0	2	0
FAM197Y6	0	0	1	0	0	0	0	0	3	0	2	0
FAM197Y7	0	0	0	0	0	0	0	0	2	0	0	0
FAM197Y8	0	0	2	0	0	0	0	0	0	0	1	0
FAM197Y9	0	0	2	0	0	0	0	0	2	0	0	0
FAM198A	13	14	13	19	6	13	8	11	21	7	12	2
FAM198B	63	469	51	335	161	251	351	314	214	114	217	68
FAM199X	168	711	166	722	377	587	602	790	343	181	377	110
FAM19A1	26	2	3	11	7	3	1	3	12	1	7	0
FAM19A2	21	5	20	2	14	0	3	7	12	2	15	1
FAM19A3	3	0	1	0	0	0	0	0	3	0	1	0
FAM19A4	11	0	9	0	1	0	0	0	9	0	5	1
FAM19A5	8	6	5	1	2	0	0	2	4	1	3	1
FAM200A	24	72	12	65	33	42	79	84	51	27	63	21

FAM200B	37	115	49	110	80	125
	134	171	98	40	87	26
FAM203A	4	48	8	10	12	13
	15	40	12	12	11	1
FAM203B	2	5	0	8	6	13
	2	7	6	5	9	3
FAM204A	116	462	118	282	232	474
	443	665	231	192	348	88
FAM205A	21	0	18	0	4	0
	0	1	3	0	2	0
FAM206A	32	77	19	40	32	39
	43	69	51	19	39	14
FAM207A	8	35	4	18	10	21
	33	37	13	9	22	3
FAM208A	143	554	154	486	296	413
	414	573	231	172	327	105
FAM208B	197	888	191	629	544	590
	626	921	420	252	571	185
FAM209A	1	2	0	0	1	0
	0	0	2	0	0	0
FAM209B	0	0	0	5	2	2
	1	3	0	1	1	0
FAM20A	41	27	33	44	22	31
	14	23	36	5	25	12
FAM20B	152	606	116	506	317	433
	466	676	274	216	327	125
FAM20C	33	111	36	58	39	70
	76	83	35	22	54	12
FAM210A	85	441	64	265	200	195
	436	426	181	114	269	53
FAM210B	121	918	156	567	250	531
	824	608	336	126	246	123
FAM211A	13	15	4	5	5	2
	0	1	4	1	4	0
FAM211B	4	3	1	4	0	3
	5	9	1	3	2	0
FAM212A	7	26	2	20	8	23
	9	13	10	4	5	3
FAM212B	63	174	52	79	77	73
	43	67	37	20	23	13
FAM213A	66	283	40	122	137	200
	243	278	89	65	119	61
FAM213B	10	14	18	13	10	12
	4	9	9	5	9	0
FAM214A	184	866	182	547	400	447
	917	1342	352	270	519	228
FAM214B	28	39	11	24	29	37
	32	33	35	9	18	3
FAM216A	10	5	5	15	5	24
	29	26	9	2	11	5
FAM216B	16	4	14	0	7	4
	0	4	10	0	8	3
FAM217A	9	0	5	0	7	0
	0	0	3	1	4	0
FAM217B	106	537	117	512	265	415
	408	497	235	159	332	70
FAM218A	23	9	16	21	9	17
	10	9	23	3	12	6

FAM219A	61	156	37	114	78	136
	132	220	91	36	101	30
FAM219B	71	275	56	231	123	236
	241	371	167	77	148	54
FAM21A	48	239	80	248	124	225
	226	253	106	92	74	89
FAM21B	38	99	3	3	54	13
	10	108	64	30	119	4
FAM21C	129	382	121	248	180	222
	264	332	176	112	219	82
FAM220A	92	472	65	366	193	381
	401	505	217	111	301	83
FAM221A	9	13	4	13	12	3
	23	51	13	6	19	6
FAM221B	31	3	9	0	7	0
	0	2	10	2	7	0
FAM222A	7	22	8	10	15	13
	16	12	5	4	9	1
FAM222B	29	95	24	47	41	56
	78	67	36	31	41	19
FAM227A	70	8	29	1	9	11
	2	3	33	3	21	1
FAM227B	30	70	35	34	32	67
	71	129	48	24	47	10
FAM228A	2	0	7	4	3	2
	3	2	5	1	4	1
FAM228B	50	153	38	101	63	126
	116	262	74	42	84	23
FAM22A	8	14	6	3	4	14
	4	7	7	0	12	2
FAM22D	80	24	61	4	24	6
	10	24	42	2	32	5
FAM22F	5	1	8	0	1	1
	0	0	0	0	1	0
FAM22G	22	3	15	1	4	0
	3	4	9	1	6	0
FAM24A	9	0	2	0	2	0
	0	0	1	0	1	0
FAM24B	7	4	4	0	1	3
	3	1	3	0	3	2
FAM25A	1	0	0	0	0	0
	0	0	2	0	0	0
FAM25B	1	0	0	0	0	0
	0	0	0	0	3	0
FAM25C	5	1	1	0	0	0
	0	0	1	0	1	0
FAM25G	1	0	0	0	0	0
	0	0	1	0	0	0
FAM26D	6	0	8	0	3	1
	0	4	5	0	2	0
FAM26E	46	60	28	51	32	28
	47	116	67	51	44	14
FAM26F	3	1	5	3	3	5
	1	3	5	0	5	1
FAM27E3	66	2	23	1	14	5
	2	6	11	0	7	2
FAM27E4P	3	0	1	0	0	0
	0	0	1	0	2	0

FAM32A	44	209	30	89	103	144
	164	249	58	57	99	40
FAM35A	73	297	59	183	144	202
	179	275	126	95	173	37
FAM3A	25	63	13	52	24	37
	45	75	30	15	37	13
FAM3B	9	0	6	1	4	1
	2	0	9	0	5	0
FAM3C	70	358	66	175	146	252
	244	283	128	101	170	62
FAM3D	4	1	3	0	2	0
	0	0	5	0	1	0
FAM40A	60	169	43	161	65	142
	145	216	115	82	115	36
FAM40B	251	698	166	429	480	540
	617	884	352	267	546	147
FAM43A	15	50	18	35	27	49
	30	42	23	13	22	4
FAM43B	6	7	3	10	4	11
	1	1	4	0	4	0
FAM45A	54	201	44	182	109	179
	139	223	103	44	119	41
FAM46A	45	182	83	186	64	158
	120	179	110	44	129	26
FAM46B	3	6	1	2	1	1
	1	7	3	1	7	2
FAM46C	127	975	110	650	378	337
	651	476	230	137	385	130
FAM46D	18	0	7	0	8	1
	0	1	7	0	2	0
FAM47A	9	1	2	2	2	0
	0	4	4	0	2	0
FAM47B	6	0	1	0	1	0
	0	0	0	0	0	0
FAM47C	11	0	4	0	2	0
	0	0	1	0	0	0
FAM47E	44	46	26	34	35	53
	36	71	39	22	38	6
FAM47E-STBD1	262	1672	362	894	735	
	1041	1131	1330	601	392	804
	182					
FAM48A	91	391	98	287	223	297
	293	411	192	131	245	66
FAM48B1	11	4	0	0	2	0
	3	0	2	0	0	0
FAM48B2	6	0	1	1	0	0
	1	0	0	0	0	0
FAM49A	48	90	27	122	72	199
	76	135	90	62	86	21
FAM49B	133	621	160	394	252	475
	373	552	251	137	262	62
FAM50A	36	202	34	85	83	143
	145	198	71	68	90	28
FAM50B	31	115	15	47	74	92
	97	135	62	40	98	25
FAM53A	8	5	6	13	2	2
	1	3	5	1	5	1

FAM53B	92	352	95	313	190	280
	227	351	148	97	185	64
FAM53C	156	638	127	623	357	624
	550	747	276	205	425	108
FAM54A	11	2	10	8	2	4
	1	3	10	3	7	1
FAM54B	111	643	68	468	225	452
	503	766	312	197	354	94
FAM57A	9	8	8	5	5	3
	7	7	7	1	9	1
FAM57B	11	4	5	17	0	17
	6	1	2	1	2	0
FAM58A	23	99	13	56	27	68
	80	116	50	21	49	13
FAM58BP	4	0	0	0	0	0
	0	0	2	0	1	0
FAM59A	90	120	58	139	74	87
	133	211	115	82	107	50
FAM59B	24	13	24	5	21	12
	24	21	13	16	23	5
FAM5B	8	0	11	0	4	0
	0	0	8	0	7	0
FAM5C	22	3	9	9	11	4
	4	3	12	1	13	0
FAM60A	46	35	26	27	21	27
	41	54	40	15	30	11
FAM63A	33	103	23	75	51	63
	72	100	68	39	61	28
FAM63B	71	229	63	184	115	128
	205	333	130	81	135	56
FAM64A	5	0	4	0	4	0
	1	1	0	0	0	0
FAM65A	62	175	46	97	70	71
	112	126	41	51	62	26
FAM65B	90	367	92	88	162	215
	291	431	209	118	193	70
FAM65C	30	60	13	24	30	35
	35	62	33	14	30	8
FAM69A	43	154	19	114	97	89
	88	118	50	32	57	9
FAM69B	9	23	4	21	10	16
	18	22	22	3	18	11
FAM69C	8	5	2	9	2	2
	9	5	3	5	7	0
FAM70A	33	10	7	5	20	10
	9	20	10	3	9	2
FAM70B	7	5	4	3	7	2
	4	6	3	2	1	5
FAM71A	9	0	10	0	2	0
	0	0	4	0	3	0
FAM71B	12	0	4	0	1	0
	0	0	4	0	5	0
FAM71C	5	1	6	0	2	1
	0	0	4	0	2	0
FAM71D	14	2	6	1	2	5
	0	3	6	1	3	2
FAM71E1	6	5	2	5	1	4
	7	5	11	4	2	2

FAM71E2	11	0	14	0	4	0
	0	0	4	0	2	0
FAM71F1	14	1	12	2	5	0
	1	2	4	0	8	1
FAM71F2	6	9	3	9	5	8
	8	25	8	6	5	3
FAM72A	14	1	7	1	3	2
	5	1	8	0	6	0
FAM72B	10	2	12	1	1	1
	0	1	9	0	6	0
FAM72D	23	1	17	0	5	0
	2	0	30	0	10	0
FAM73A	149	430	106	363	221	313
	360	562	269	153	327	97
FAM73B	30	95	26	42	24	35
	71	133	55	25	50	13
FAM76A	61	264	68	198	151	189
	263	294	154	73	219	43
FAM76B	55	131	50	116	70	130
	127	219	103	60	126	38
FAM78A	76	377	80	287	214	324
	322	300	132	93	194	51
FAM78B	3	14	2	5	7	18
	9	6	14	3	5	2
FAM81A	14	5	12	0	5	2
	0	0	11	0	8	0
FAM81B	19	0	16	0	5	0
	0	0	8	0	5	0
FAM82A1	61	71	61	66	53	51
	71	71	47	26	56	17
FAM82A2	35	70	21	55	39	49
	47	83	49	34	54	16
FAM82B	117	642	119	504	219	475
	487	715	282	199	405	103
FAM83A	10	1	4	0	1	0
	0	0	4	0	1	0
FAM83B	34	71	49	18	46	24
	50	89	15	14	45	17
FAM83C	10	1	6	0	0	0
	0	0	5	0	1	0
FAM83D	9	0	7	0	3	1
	0	0	5	0	1	0
FAM83E	1	0	0	0	0	2
	1	1	3	0	1	0
FAM83F	6	0	3	0	0	0
	0	0	3	0	0	0
FAM83G	7	2	7	1	1	0
	1	3	2	2	3	0
FAM83H	11	0	4	0	1	1
	1	0	6	0	1	0
FAM84A	28	17	14	13	12	14
	6	18	22	2	8	1
FAM84B	35	114	20	35	59	74
	103	173	71	27	66	29
FAM86A	17	25	15	18	14	19
	15	29	13	7	12	0
FAM86B1	5	4	6	7	5	0
	4	7	12	0	4	0

FAM86B2	11	1	6	0	2	1
	3	1	2	0	3	2
FAM86C1	23	10	5	1	6	4
	15	6	6	2	5	3
FAM89A	8	23	7	12	12	8
	32	31	18	8	12	5
FAM89B	17	70	3	39	23	39
	85	81	44	23	44	21
FAM8A1	82	362	73	279	225	366
	332	546	207	135	268	89
FAM90A1	9	0	7	0	5	0
	2	0	5	0	5	0
FAM91A1	68	198	81	219	111	182
	194	314	125	76	196	46
FAM92A1	27	99	19	62	48	51
	109	132	66	33	85	12
FAM92B	15	0	5	0	0	0
	0	1	5	0	1	0
FAM96A	84	356	50	295	146	222
	474	505	199	86	245	50
FAM96B	23	134	13	79	76	116
	130	200	77	44	66	21
FAM98A	118	621	72	297	273	354
	401	420	167	98	183	55
FAM98B	15	96	24	97	51	57
	108	74	33	39	63	15
FAM98C	7	4	9	4	1	7
	1	8	5	1	3	4
FAM9A	16	1	5	0	6	1
	0	1	8	0	1	0
FAM9B	5	0	2	0	2	0
	0	0	4	0	2	0
FAM9C	14	0	8	0	6	0
	1	2	3	0	2	0
FAN1	141	562	150	380	239	388
	422	665	283	155	297	102
FANCA	43	4	26	7	10	5
	8	9	20	0	16	0
FANCB	19	4	12	7	6	7
	1	13	13	5	8	4
FANCC	40	89	34	56	60	54
	61	102	66	38	42	10
FANCD2	107	17	42	17	16	13
	8	14	53	4	35	4
FANCE	20	53	19	36	27	49
	53	104	37	9	34	13
FANCF	9	33	10	23	22	22
	16	28	15	4	17	3
FANCG	26	13	17	14	11	8
	18	12	13	5	13	2
FANCI	59	23	34	25	29	13
	15	9	37	4	31	1
FANCL	34	136	59	80	70	115
	114	201	58	48	96	25
FANCM	84	212	94	175	131	161
	150	260	127	67	204	47
FANK1	17	1	10	0	5	2
	0	3	15	0	11	0

FAP	66	156	67	130	118	190
	103	209	88	42	96	45
FAR1	166	831	180	633	373	484
	539	719	377	194	438	119
FAR2	30	25	22	24	18	13
	15	31	30	3	18	5
FARP1	66	165	47	98	83	89
	168	117	129	51	84	24
FARP2	67	154	50	146	94	120
	125	180	105	54	108	38
FARS2	34	154	33	115	81	109
	216	204	82	53	108	31
FARSA	28	134	37	83	57	83
	112	155	75	38	83	33
FARSB	113	502	96	305	236	339
	424	460	185	166	287	73
FAS	24	110	24	60	39	45
	41	67	45	13	37	21
FASLG	17	1	7	3	5	0
	3	1	8	1	11	0
FASN	14	66	20	27	29	31
	27	29	23	8	16	35
FASTK	38	118	29	94	55	114
	99	158	86	46	88	26
FASTKD1	80	326	44	189	91	146
	260	307	150	74	174	32
FASTKD2	227	884	223	713	406	652
	730	1223	558	280	731	165
FASTKD3	41	87	19	59	55	69
	76	125	30	31	68	16
FASTKD5	3	17	2	6	7	8
	25	29	15	4	11	2
FAT1	156	473	191	412	189	306
	224	212	190	90	181	114
FAT2	101	15	43	7	17	11
	2	7	37	0	20	2
FAT3	167	68	100	52	48	66
	57	35	81	17	70	43
FAT4	219	1019	313	903	401	643
	516	558	555	181	484	145
FATE1	4	1	3	1	1	2
	0	3	4	0	1	0
FAU	199	803	173	717	335	689
	1188	1346	445	234	515	108
FAXC	59	4	26	4	26	3
	1	0	20	1	22	1
FBF1	16	13	18	19	14	17
	18	19	17	6	7	4
FBL	25	58	17	54	25	48
	60	99	28	22	45	10
FBLIM1	25	8	12	6	11	6
	2	8	15	3	7	3
FBLN1	81	142	52	306	81	102
	75	146	74	26	74	48
FBLN2	136	532	98	527	389	488
	217	625	223	120	263	120
FBLN5	50	188	54	162	112	149
	111	207	83	49	109	49

FBLN7	17	8	8	0	4	8
	4	2	3	2	5	1
FBN1	470	1738	553	2555	1930	1871
	802	2734	924	389	1392	701
FBN2	115	6	93	5	36	1
	2	4	73	1	50	0
FBN3	35	5	19	0	8	2
	0	1	26	1	10	0
FBP1	11	1	5	3	5	1
	2	1	6	1	3	0
FBP2	567	3161	593	2107	787	3279
	3928	4158	1803	915	2081	273
FBRS	23	72	16	27	34	46
	52	45	17	14	27	13
FBRSL1	20	103	19	47	37	39
	67	100	21	32	33	20
FBXL12	23	47	13	22	16	31
	50	54	17	13	28	9
FBXL13	32	7	18	10	6	8
	5	7	15	5	14	0
FBXL14	8	10	3	13	6	16
	13	13	15	6	11	4
FBXL15	6	10	4	14	10	7
	10	19	13	6	5	1
FBXL16	7	0	4	0	2	2
	0	0	0	0	2	0
FBXL17	204	1150	266	1290	524	975
	1230	922	519	247	654	211
FBXL18	39	52	18	31	25	24
	32	56	31	15	20	10
FBXL19	6	18	5	12	13	11
	14	11	7	5	12	0
FBXL2	23	6	5	13	6	5
	7	7	17	1	18	4
FBXL20	151	373	77	248	200	228
	290	488	207	119	229	103
FBXL21	12	0	4	0	10	0
	0	0	11	0	4	0
FBXL22	8	23	6	11	7	17
	6	2	11	1	3	3
FBXL3	133	606	148	513	364	572
	613	743	388	248	426	137
FBXL4	98	263	84	206	169	121
	244	440	209	122	170	70
FBXL5	269	1268	263	1149	634	987
	1055	1548	675	335	818	243
FBXL6	5	16	7	13	4	10
	12	34	14	4	13	3
FBXL7	18	58	11	40	15	17
	44	34	25	2	30	4
FBXL8	0	2	1	4	1	4
	3	0	6	0	2	0
FBXO10	50	138	36	115	68	154
	116	142	85	40	50	24
FBXO11	195	972	182	636	482	570
	662	892	344	275	448	215
FBXO15	24	1	7	0	1	0
	0	1	13	0	7	0

FBXO16	20	20	17	11	14	10
	8	15	13	3	5	2
FBXO17	14	8	15	7	4	6
	15	9	5	6	6	3
FBXO18	90	323	74	215	180	224
	280	419	181	99	210	69
FBXO2	3	0	5	3	1	0
	0	0	2	0	1	0
FBXO21	166	458	129	384	391	300
	406	594	205	164	347	130
FBXO22	55	223	74	235	122	185
	189	252	110	57	141	49
FBXO24	10	3	19	1	6	3
	0	2	10	2	7	0
FBXO25	48	203	57	144	81	136
	201	186	96	52	103	31
FBXO27	5	1	3	0	1	1
	1	1	6	0	2	2
FBXO28	119	469	121	405	280	408
	411	606	300	160	384	85
FBXO3	207	966	201	775	549	780
	1080	1360	571	272	645	170
FBXO30	108	463	126	491	205	399
	459	631	260	110	278	112
FBXO31	106	374	98	294	161	346
	229	472	147	94	201	84
FBXO32	2422	8688	882	7523	5346	7441
	11602	10586	4149	4240	5842	2023
FBXO33	29	97	26	65	37	63
	50	137	35	34	65	23
FBXO34	130	569	154	427	304	430
	498	442	253	187	334	107
FBXO36	11	11	13	4	9	7
	10	17	12	2	6	2
FBXO38	150	550	144	396	272	356
	417	673	262	184	364	106
FBXO39	19	3	9	1	4	0
	2	0	12	0	1	0
FBXO4	19	64	22	50	25	41
	40	103	58	19	61	18
FBXO40	1058	3885	585	3052	2335	3376
	3866	5142	1939	1670	2924	1095
FBXO41	29	9	18	4	6	0
	4	3	12	0	5	0
FBXO42	51	183	48	118	76	149
	131	158	62	42	110	27
FBXO43	10	1	9	1	3	0
	0	0	12	0	6	0
FBXO44	18	17	11	16	11	4
	23	28	18	3	15	9
FBXO45	98	352	79	316	149	285
	279	488	167	123	199	51
FBXO46	21	36	12	27	19	20
	35	30	11	8	14	4
FBXO47	28	0	10	0	4	0
	0	1	18	1	7	0
FBXO48	17	44	18	28	30	43
	45	48	30	14	17	6

FBXO5	13	7	9	18	13	18
	18	14	16	3	12	3
FBXO6	9	20	6	19	10	16
	10	29	13	4	12	2
FBXO7	87	372	69	213	123	278
	303	375	161	112	188	69
FBXO8	50	106	20	97	70	90
	100	217	69	39	99	34
FBXO9	157	508	129	448	279	515
	478	677	232	189	336	71
FBXW10	32	10	23	0	13	4
	4	4	10	5	6	1
FBXW11	134	710	137	545	320	478
	540	709	341	185	396	108
FBXW12	19	0	6	0	1	0
	1	1	7	0	9	0
FBXW2	164	659	157	554	313	453
	546	719	311	182	374	129
FBXW4	35	143	25	147	74	121
	139	174	77	47	91	25
FBXW5	101	426	62	259	163	279
	297	580	219	200	279	118
FBXW7	245	1062	246	722	476	636
	963	1530	595	437	816	183
FBXW8	54	108	39	73	42	103
	94	136	55	39	67	37
FBXW9	3	1	4	2	1	0
	3	3	3	0	3	0
FCAMR	19	0	8	0	3	0
	0	1	5	0	8	0
FCAR	13	0	4	0	5	0
	0	2	7	0	5	0
FCER1A	9	5	8	9	3	4
	2	14	13	5	3	2
FCER1G	3	16	4	15	18	26
	14	28	11	5	16	2
FCER2	15	2	11	0	7	0
	0	0	8	0	9	0
FCF1	32	96	37	59	65	94
	67	100	43	28	81	19
FCGBP	51	5	45	12	5	3
	3	0	21	0	15	0
FCGR1A	31	4	20	2	9	3
	4	1	6	2	12	1
FCGR1B	29	2	19	4	9	2
	0	2	9	1	5	0
FCGR2A	44	53	20	34	31	78
	35	111	45	21	47	21
FCGR2B	19	15	14	25	24	30
	13	54	18	10	29	6
FCGR3A	26	9	27	8	5	8
	21	15	18	3	13	4
FCGR3B	18	30	4	2	5	12
	16	8	7	8	8	4
FCGRT	32	101	37	77	77	112
	139	219	83	31	83	21
FCHO1	25	5	10	1	8	1
	1	2	12	1	4	0

FCHO2	124	419	125	365	200	289
	377	713	249	147	324	112
FCHSD1	33	28	29	30	18	26
	13	29	34	7	30	7
FCHSD2	74	307	82	155	117	125
	151	233	126	73	106	37
FCN1	4	17	5	1	2	8
	17	7	3	2	5	0
FCN2	7	1	3	0	2	0
	0	0	1	0	2	0
FCN3	4	0	3	0	0	1
	2	6	1	0	1	1
FCRL1	20	5	6	4	4	1
	0	0	15	0	8	0
FCRL2	12	0	11	0	5	0
	0	0	9	0	4	0
FCRL3	27	5	17	3	9	1
	1	0	9	0	4	4
FCRL4	28	5	21	4	4	0
	0	1	14	3	12	1
FCRL5	54	1	20	0	5	0
	0	0	8	0	7	0
FCRL6	9	3	5	0	3	1
	0	0	4	0	2	0
FCRLA	9	1	10	1	6	0
	0	1	5	0	3	0
FCRLB	6	0	3	0	1	1
	1	1	4	0	1	0
FDCSP	11	0	4	0	5	0
	0	0	3	0	2	0
FDFT1	221	838	152	836	429	721
	847	1037	504	262	802	149
FDPS	49	230	53	167	105	183
	204	314	131	61	134	36
FDX1	76	364	68	319	165	280
	339	448	176	93	261	45
FDX1L	12	50	5	36	22	48
	43	87	26	9	38	6
FDXACB1	20	15	7	15	15	10
	20	25	31	3	16	2
FDXR	12	16	10	10	9	14
	10	14	15	5	9	4
FECH	115	472	110	398	220	343
	349	459	233	149	316	80
FEM1A	759	4008	507	2886	1769	2286
	3044	3678	1638	1066	2476	667
FEM1B	64	295	98	284	173	248
	277	381	148	120	201	59
FEM1C	180	1265	301	1127	649	928
	858	1247	365	327	587	152
FEN1	16	42	20	50	24	37
	37	66	24	24	43	7
FER	58	225	64	181	114	142
	110	156	86	58	105	36
FER1L5	51	1	28	3	9	3
	0	2	19	1	10	0
FER1L6	60	2	42	0	15	5
	3	1	36	0	20	0

FERD3L	1	0	1	0	0	0
	0	0	0	0	0	0
FERMT1	25	8	14	8	18	4
	2	7	23	4	6	1
FERMT2	131	779	150	615	390	538
	556	698	305	212	362	136
FERMT3	12	15	9	13	9	7
	11	15	12	4	9	3
FES	7	24	9	26	9	20
	15	22	17	5	11	4
FETUB	13	0	9	0	3	0
	0	0	7	0	3	0
FEV	2	0	1	0	0	0
	0	0	0	0	0	0
FEZ1	25	24	19	50	16	32
	14	16	20	8	16	3
FEZ2	698	2127	966	1151	1114	1248
	1237	2074	384	423	1004	403
FEZF1	10	4	4	0	2	0
	0	0	4	0	1	0
FEZF2	8	0	10	0	2	0
	0	0	9	0	2	0
FFAR1	1	0	0	0	0	0
	0	0	0	0	1	0
FFAR2	7	2	3	0	1	3
	1	1	4	0	0	0
FFAR3	7	1	2	1	3	0
	0	0	3	0	2	0
FGA	26	0	9	0	8	0
	0	0	9	0	11	0
FGB	33	0	16	0	8	0
	0	1	24	0	7	0
FGD1	21	15	8	12	7	8
	7	12	10	7	8	0
FGD2	12	15	12	19	18	10
	15	32	19	4	16	5
FGD3	18	14	9	5	13	10
	12	15	11	6	7	3
FGD4	225	1121	331	856	517	482
	1614	2247	667	408	1064	287
FGD5	44	191	43	156	71	94
	140	188	91	51	87	42
FGD6	103	308	116	173	116	133
	152	233	126	63	114	48
FGF1	39	47	16	43	37	33
	41	43	35	19	21	24
FGF10	4	6	3	2	4	1
	2	8	3	1	2	1
FGF11	8	24	11	6	10	17
	17	5	12	8	7	2
FGF12	42	45	31	52	36	49
	27	34	32	19	35	5
FGF13	80	335	59	265	152	319
	332	353	177	109	242	67
FGF14	16	0	15	5	8	5
	1	2	16	1	7	2
FGF16	5	2	1	1	2	3
	1	0	0	0	0	0

FGF17	4	1	2	0	1	0
	0	0	1	0	1	0
FGF18	5	8	2	3	9	5
	2	4	2	1	2	0
FGF19	5	0	0	0	0	0
	0	0	0	0	0	0
FGF2	31	81	49	86	62	69
	59	162	68	35	51	31
FGF20	7	0	5	0	3	0
	0	0	6	0	4	0
FGF21	0	0	2	0	1	4
	1	0	3	0	1	0
FGF22	0	0	0	0	0	0
	0	1	0	0	0	0
FGF23	13	0	7	2	1	0
	0	0	8	1	5	0
FGF3	4	0	2	0	0	0
	0	0	0	0	1	0
FGF4	1	0	1	0	0	0
	0	0	3	0	1	0
FGF5	19	1	11	1	5	0
	1	1	8	0	4	0
FGF6	9	42	8	31	14	33
	26	28	22	16	21	10
FGF7	43	60	63	76	47	103
	102	172	46	45	96	39
FGF8	12	1	4	0	2	1
	0	0	5	0	4	0
FGF9	18	35	24	10	23	22
	20	34	11	7	14	1
FGFBP1	6	0	5	0	0	0
	0	0	0	0	1	0
FGFBP2	3	0	1	1	0	2
	0	3	2	0	2	0
FGFBP3	15	5	11	9	6	3
	5	11	8	2	5	1
FGFR1	88	321	86	314	185	185
	176	335	116	87	133	77
FGFR1OP	39	105	32	80	65	82
	86	118	61	23	63	15
FGFR1OP2	109	501	124	405	284	361
	417	663	182	186	299	113
FGFR2	42	5	24	7	13	2
	7	1	18	0	13	1
FGFR3	11	5	16	3	9	6
	0	1	11	1	2	1
FGFR4	13	25	15	10	10	27
	12	17	21	5	10	5
FGFRL1	19	59	19	28	34	37
	57	105	23	30	39	16
FGG	13	0	6	0	3	0
	0	0	14	0	4	0
FGGY	94	290	104	175	141	219
	322	496	246	100	199	50
FGL1	10	0	5	0	5	0
	0	0	9	0	7	0
FGL2	95	407	93	345	216	229
	297	370	206	130	221	95

FGR	7	10	10	7	0	4
	6	17	11	1	7	1
FH	222	1515	249	988	592	909
	1498	1733	678	421	958	195
FHAD1	34	1	29	1	7	1
	0	0	33	1	15	0
FHDC1	30	15	21	10	17	17
	4	10	19	0	9	0
FHIT	12	10	10	16	12	13
	28	23	12	2	18	2
FHL1	11332	42371	6279	31973	27861	34172
	51466	65901	28063	13896	36495	9088
FHL2	45	92	31	174	94	89
	235	103	62	41	124	25
FHL3	222	1070	195	791	551	829
	1900	1250	628	458	904	316
FHL5	31	118	28	95	44	63
	87	165	53	46	63	37
FHOD1	146	544	78	426	209	368
	433	461	227	147	339	87
FHOD3	180	848	167	375	283	627
	403	879	356	282	363	134
FIBCD1	6	0	3	0	1	0
	0	0	5	0	2	0
FIBIN	21	33	19	45	24	31
	15	104	37	20	36	25
FIBP	21	103	18	91	49	98
	97	140	51	28	72	21
FICD	11	4	1	5	1	9
	10	8	6	1	6	0
FIG4	61	145	62	119	65	143
	104	142	91	42	124	29
FIGF	46	98	34	101	70	91
	49	170	51	26	58	23
FIGLA	9	1	4	0	1	0
	0	1	1	1	1	0
FIGN	49	82	24	90	47	67
	77	62	53	15	57	19
FIGNL1	55	180	79	150	105	174
	167	234	83	56	127	38
FIGNL2	11	6	8	6	5	1
	1	3	3	0	1	0
FILIP1	430	2441	555	1537	1279	1358
	2009	2676	930	1181	1522	568
FILIP1L	944	6043	1452	4751	2852	4315
	4185	4508	2477	2128	3336	993
FIP1L1	79	354	102	210	175	243
	280	387	205	132	229	66
FIS1	88	397	61	317	158	231
	471	752	268	86	250	63
FITM1	162	488	119	515	280	440
	887	1080	491	227	511	147
FITM2	6	23	3	11	9	8
	15	20	13	3	13	2
FIZ1	7	8	3	4	3	8
	10	9	3	2	4	1
FJX1	4	2	4	11	1	1
	1	2	4	2	1	1

FKBP10	29	41	15	35	24	28
	20	46	18	10	17	5
FKBP11	6	15	5	4	6	6
	6	12	4	1	8	0
FKBP14	38	84	28	93	53	78
	65	108	49	28	60	16
FKBP15	122	308	120	200	144	227
	232	405	185	116	228	46
FKBP1A	12	300	11	174	80	163
	228	297	77	43	92	55
FKBP1B	5	2	1	2	2	3
	8	2	2	2	1	1
FKBP2	18	61	19	50	27	62
	91	111	27	17	30	12
FKBP3	365	2407	330	1803	1058	1584
	2292	2717	878	604	1257	329
FKBP4	121	517	85	279	335	337
	356	556	193	160	210	75
FKBP5	278	562	118	479	713	510
	3416	4583	569	741	2020	897
FKBP6	13	0	9	1	3	0
	0	0	6	0	4	0
FKBP7	39	42	22	57	23	41
	69	71	42	17	39	14
FKBP8	67	355	42	202	120	209
	238	351	166	91	154	73
FKBP9	151	595	117	411	276	374
	421	573	254	192	282	120
FKBPL	4	10	3	14	9	7
	7	16	7	1	13	1
FKRP	26	59	16	30	43	61
	68	98	30	24	61	19
FKTN	108	309	93	250	178	238
	222	353	188	95	211	54
FLAD1	34	84	24	58	51	72
	79	120	53	39	55	10
FLCN	68	173	39	137	133	152
	166	252	107	54	100	44
FLG	76	1	34	0	4	3
	0	0	26	0	15	1
FLG2	66	0	31	0	10	0
	0	2	29	0	12	2
FLI1	39	120	19	70	49	74
	108	114	70	46	74	17
FLII	400	2040	299	1159	853	1251
	1459	1787	888	613	1145	412
FLJ22184	10	0	1	0	1	0
	0	0	4	0	1	0
FLJ23152	13	0	7	0	1	0
	0	0	6	0	1	0
FLJ25363	4	0	5	0	2	0
	0	0	4	0	2	0
FLJ27352	6	15	4	10	6	9
	10	12	5	1	13	4
FLJ42280	7	0	8	0	9	0
	0	0	15	0	7	0
FLJ43860	30	8	13	1	2	3
	2	4	13	1	10	2

FLJ44635	10	5	3	7	7	3
	0	5	7	3	8	2
FLJ45513	7	16	5	14	14	9
	11	12	7	7	3	5
FLNA	206	1081	168	771	379	479
	350	752	363	234	383	394
FLNB	141	474	127	409	214	251
	315	497	250	149	243	116
FLNC	3086	15730	1925	9250	7231	10137
	9964	20171	7015	6191	8938	3625
FLOT1	125	578	89	390	241	463
	608	564	254	227	276	99
FLOT2	23	215	44	119	94	114
	207	184	90	68	86	43
FLRT1	11	4	4	0	2	6
	3	2	6	0	3	0
FLRT2	327	1197	296	910	458	478
	748	1207	246	404	491	192
FLRT3	41	56	24	29	53	34
	146	401	108	53	190	31
FLT1	268	1095	133	560	450	565
	983	1122	475	413	653	182
FLT3	36	5	12	3	11	1
	3	2	23	1	7	1
FLT3LG	9	6	5	1	7	10
	8	4	3	4	5	1
FLT4	25	49	20	25	32	14
	29	25	20	14	29	7
FLVCR1	30	37	20	25	27	24
	22	43	39	22	27	8
FLVCR2	35	50	24	54	32	24
	37	61	41	10	34	11
FLYWCH1	23	141	26	69	33	73
	50	95	64	32	69	25
FLYWCH2	9	30	5	28	19	35
	44	43	28	6	25	8
FMN1	91	62	65	33	36	64
	13	63	71	13	45	17
FMN2	26	0	27	9	10	17
	0	2	21	0	10	0
FMNL1	22	43	17	21	16	31
	10	44	19	7	35	6
FMNL2	127	380	146	328	198	353
	339	644	265	175	208	97
FMNL3	116	258	76	163	110	131
	191	177	156	77	128	47
FMO1	16	8	12	6	5	0
	10	0	14	0	4	0
FMO2	144	339	47	251	275	263
	413	794	243	218	454	203
FMO3	27	59	20	85	17	50
	71	38	64	5	67	6
FMO4	21	28	18	14	13	14
	24	24	40	2	29	7
FMO5	19	24	17	13	16	13
	21	22	32	0	13	4
FMOD	23	77	16	71	33	42
	43	71	33	34	28	30

FMR1	76	343	82	215	139	236
	221	369	103	116	181	51
FMR1NB	5	0	7	0	3	0
	0	0	2	0	2	0
FN1	477	1520	465	2585	851	1708
	1278	1627	1059	382	900	357
FN3K	16	31	7	25	19	29
	42	30	14	5	13	5
FN3KRP	21	49	15	41	32	44
	62	84	43	20	25	18
FNBP1	83	345	74	257	172	253
	235	224	98	101	136	71
FNBP1L	156	443	124	257	247	275
	466	614	207	166	314	87
FNBP4	253	980	208	668	555	737
	1082	1168	552	472	547	199
FNDC1	59	111	47	170	94	165
	36	76	50	4	56	32
FNDC3A	232	920	229	650	406	604
	706	1081	480	248	533	174
FNDC3B	221	932	253	676	447	732
	734	1144	405	295	508	210
FNDC4	8	9	8	11	1	3
	3	4	8	1	2	4
FNDC5	514	2174	403	1092	1013	957
	1455	1147	378	303	581	307
FNDC7	27	0	13	3	6	1
	0	0	10	0	9	0
FNDC8	7	0	2	0	0	0
	0	0	1	0	1	0
FNDC9	16	0	6	1	2	1
	0	3	12	0	2	1
FNIP1	247	1086	284	847	579	787
	1121	1567	612	432	913	283
FNIP2	93	314	72	185	140	185
	242	350	185	97	250	63
FNTA	126	606	133	387	302	464
	564	784	324	203	369	122
FNTB	3	15	4	3	6	12
	13	22	6	6	7	3
FOCAD	185	504	161	401	257	332
	349	678	370	159	341	88
FOLH1	41	7	22	2	17	3
	3	2	22	0	20	1
FOLH1B	18	0	13	0	4	0
	0	0	18	0	7	0
FOLR1	11	0	4	0	1	0
	0	0	9	0	2	0
FOLR2	10	4	4	14	11	13
	2	19	11	0	11	4
FOLR3	4	0	2	0	1	0
	0	0	3	0	0	0
FOLR4	6	0	4	0	1	0
	0	0	0	0	1	0
FOPNL	133	488	91	490	318	411
	463	604	264	174	316	108
FOS	18	152	121	326	61	147
	65	111	113	29	20	25

FOSB	18	3	23	9	4	3
	0	2	5	0	3	1
FOSL1	13	1	5	0	1	0
	3	0	1	0	1	0
FOSL2	60	167	78	104	136	139
	226	218	88	62	137	58
FOXA1	9	2	4	0	2	0
	0	0	2	0	2	0
FOXA2	10	0	5	0	1	0
	0	0	2	0	1	0
FOXA3	5	0	2	0	0	0
	0	0	1	0	0	0
FOXB1	0	1	0	0	0	0
	0	0	1	0	0	0
FOXB2	1	0	0	0	1	0
	0	0	0	4	0	0
FOXC1	18	37	16	31	13	15
	18	29	12	9	13	10
FOXC2	1	0	1	0	0	0
	1	0	2	0	0	0
FOXD1	6	0	5	12	0	0
	0	1	2	1	0	1
FOXD2	11	0	2	3	2	0
	2	2	3	0	4	1
FOXD3	5	10	3	4	2	4
	4	19	4	1	3	2
FOXD4	4	3	3	0	1	0
	0	0	4	4	0	0
FOXD4L1	6	0	6	1	1	1
	0	0	0	0	0	0
FOXD4L2	43	1	16	0	7	2
	1	3	25	1	10	0
FOXD4L3	0	0	3	0	2	0
	0	0	2	0	0	0
FOXD4L4	42	1	10	2	4	2
	2	5	14	0	13	2
FOXD4L5	130	1	19	0	21	1
	4	1	31	1	16	1
FOXD4L6	20	2	8	1	5	1
	0	2	2	0	2	1
FOXE1	2	0	4	0	1	0
	0	0	6	0	1	0
FOXE3	2	0	0	0	0	0
	0	0	0	0	1	0
FOXF1	3	0	3	0	3	1
	0	0	4	0	3	0
FOXF2	6	3	2	1	2	2
	7	2	6	0	6	2
FOXG1	6	0	0	0	4	0
	0	0	1	0	1	0
FOXH1	7	0	4	0	3	0
	0	0	7	0	0	0
FOXI1	10	0	4	0	1	0
	0	0	4	0	0	0
FOXI2	18	4	10	1	5	0
	3	0	15	1	6	1
FOXI3	10	0	4	1	1	0
	0	0	3	0	0	0

FOXJ1	3	0	4	0	0	4
	0	0	3	0	0	0
FOXJ2	78	268	54	167	140	174
	219	248	93	52	152	40
FOXJ3	207	1150	246	804	499	720
	859	980	433	286	529	165
FOXK1	107	450	90	248	157	262
	341	308	176	69	205	90
FOXK2	70	347	69	283	186	264
	284	369	129	124	153	60
FOXL1	12	10	5	4	3	2
	10	4	5	0	5	1
FOXL2	7	0	3	0	0	0
	0	0	2	0	1	0
FOXM1	19	2	11	2	6	1
	0	4	15	0	8	0
FOXN1	19	0	2	0	2	0
	0	0	11	0	4	0
FOXN2	128	387	116	412	275	322
	284	596	247	150	327	94
FOXN3	401	2172	449	1665	986	1607
	1853	2107	872	814	1337	447
FOXN4	9	0	8	0	5	1
	0	0	3	0	4	0
FOXO1	85	405	124	293	298	344
	635	829	253	214	376	249
FOXO3	279	902	194	525	617	597
	1473	2734	660	521	1113	512
FOXO4	85	312	55	251	180	260
	264	426	162	100	231	64
FOXO6	6	4	6	1	2	1
	1	1	0	1	3	0
FOXP1	112	400	101	201	188	289
	255	426	184	123	213	95
FOXP2	136	192	83	121	148	137
	162	252	171	102	229	71
FOXP3	13	1	4	0	0	0
	0	0	3	0	1	0
FOXP4	21	32	11	15	16	20
	28	22	14	18	20	18
FOXQ1	2	0	0	0	1	0
	0	0	1	0	1	0
FOXR1	7	0	4	0	0	0
	0	0	5	0	1	0
FOXR2	10	0	3	0	2	0
	0	0	7	0	1	0
FOXRED1	31	116	26	86	34	77
	125	164	62	34	75	25
FOXRED2	42	37	17	33	19	17
	17	34	26	15	31	6
FOXS1	2	6	1	2	1	5
	3	3	2	2	2	0
FPGS	31	78	19	54	26	74
	38	123	49	31	77	15
FPGT	0	21	10	23	16	16
	10	43	17	5	15	5

FPGT-TNNI3K	52	89	53	89	50	93	70	104	114	20	95	20
FPR1	5	3	1	3	0	3	2	1	6	1	0	0
FPR2	7	3	5	1	0	2	1	1	2	0	4	0
FPR3	16	17	13	15	7	26	12	34	13	11	14	6
FRA10AC1	79	298	97	194	154	261	259	362	159	107	184	64
FRAS1	194	423	303	366	230	478	353	269	282	111	290	90
FRAT1	17	19	7	13	12	11	24	31	18	6	10	5
FRAT2	27	80	12	50	41	40	90	100	43	27	38	15
FREM1	106	171	76	143	122	79	49	103	102	28	77	33
FREM2	266	1402	527	832	721	1144	684	959	386	328	584	183
FREM3	43	2	22	0	12	1	0	0	19	0	6	0
FRG1	81	352	66	168	142	242	265	404	138	104	199	41
FRG2	16	1	2	0	0	0	0	0	11	0	2	0
FRG2B	18	0	13	0	10	0	0	0	6	0	1	0
FRG2C	30	0	24	0	3	1	0	1	21	0	7	0
FRK	63	141	42	51	89	51	87	218	55	56	111	28
FRMD1	5	1	7	2	2	0	1	3	7	0	5	3
FRMD3	160	633	125	479	378	472	623	566	372	189	556	69
FRMD4A	64	99	45	87	71	66	74	111	74	37	79	43
FRMD4B	81	101	48	82	76	97	70	133	83	28	68	28
FRMD5	15	2	12	0	1	0	1	0	11	1	4	0
FRMD6	90	233	85	253	98	156	240	335	170	65	214	31
FRMD7	26	0	8	0	4	0	0	0	7	0	4	0
FRMD8	11	16	11	16	8	11	10	22	18	8	14	3
FRMPD1	49	127	22	57	41	106	61	46	38	6	38	2
FRMPD2	62	0	38	1	8	0	0	0	31	0	12	6
FRMPD3	42	0	11	3	7	0	0	1	9	0	3	1
FRMPD4	53	18	12	6	17	12	9	18	16	12	19	9

FRRS1	38	51	27	25	22	35
	30	52	39	6	28	8
FRRS1L	4	0	5	0	1	0
	0	0	3	0	0	0
FRS2	120	367	118	360	215	277
	308	453	179	105	248	74
FRS3	15	21	13	8	15	14
	17	24	18	4	13	3
FRY	358	1622	449	1179	743	1017
	1692	1310	912	389	1428	285
FRYL	313	1102	262	840	538	761
	750	1139	556	313	662	197
FRZB	94	207	37	435	88	256
	559	156	544	65	445	18
FSBP	4	16	2	20	6	14
	22	8	10	4	12	3
FSCB	15	0	10	0	3	0
	0	0	4	1	1	0
FSCN1	15	19	4	25	11	10
	20	9	13	6	9	3
FSCN2	3	3	3	0	0	0
	1	1	0	1	2	0
FSCN3	15	1	6	0	6	0
	1	1	6	0	2	0
FSD1	9	0	7	0	1	0
	0	0	0	0	1	0
FSD1L	56	396	85	208	204	173
	210	270	178	68	142	58
FSD2	274	1877	285	1034	699	1317
	1132	1506	668	441	904	178
FSHB	9	0	6	0	2	0
	0	0	6	0	0	0
FSHR	9	0	9	1	1	0
	0	0	10	0	3	0
FSIP1	27	0	19	4	14	3
	6	91	20	24	9	7
FSIP2	132	32	106	31	68	12
	34	22	130	17	86	12
FST	21	87	63	56	28	61
	19	50	15	5	25	13
FSTL1	214	938	285	1143	858	898
	436	1477	447	216	523	362
FSTL3	17	17	9	16	8	21
	12	28	11	2	16	4
FSTL4	35	0	30	1	6	1
	1	0	16	0	6	1
FSTL5	35	0	23	2	8	0
	0	1	24	0	14	0
FTCD	2	3	4	0	0	0
	3	0	2	0	2	0
FTH1	509	5155	370	2068	1525	1940
	5496	2801	832	1175	1385	508
FTHL17	1	0	0	0	0	0
	0	0	0	0	0	0
FTL	166	585	148	672	336	631
	710	1228	449	289	412	269
FTMT	4	1	0	0	0	1
	0	0	0	0	1	0

FTO	145	702	133	448	305	486
	483	581	292	198	355	113
FTSJ1	31	97	23	68	48	87
	92	125	48	35	52	12
FTSJ2	30	120	28	51	58	77
	116	148	65	32	73	9
FTSJ3	70	202	65	200	143	170
	149	213	114	86	151	40
FTSJD1	62	167	51	166	97	149
	164	200	138	62	109	35
FTSJD2	89	248	79	228	123	218
	228	314	196	105	188	70
FUBP1	168	1067	290	679	415	766
	710	1015	500	350	667	212
FUBP3	64	318	57	182	142	209
	224	358	131	85	165	57
FUCA1	38	137	54	159	80	167
	99	153	106	44	109	39
FUCA2	17	50	22	71	28	36
	37	84	47	26	38	18
FUK	15	30	16	18	17	18
	13	26	31	5	19	2
FUNDC1	36	116	20	80	43	87
	132	152	59	33	73	16
FUNDC2	370	1991	273	1364	801	1347
	2059	2883	1003	514	1158	241
FUOM	4	4	4	7	3	1
	5	11	3	0	2	0
FURIN	38	132	36	119	91	130
	171	181	84	57	120	42
FUS	63	282	67	190	139	139
	241	177	85	80	120	59
FUT1	9	27	9	11	10	22
	29	17	17	5	12	1
FUT10	43	115	38	63	50	78
	53	116	53	18	45	15
FUT11	9	53	22	38	38	35
	39	40	39	14	26	9
FUT2	20	0	2	1	1	0
	0	0	4	0	4	0
FUT3	12	1	3	0	0	0
	0	0	1	0	1	0
FUT4	37	46	17	39	23	27
	31	50	26	13	24	13
FUT5	2	2	2	0	1	0
	0	0	2	0	2	0
FUT6	11	1	2	0	0	1
	0	0	5	0	2	0
FUT7	2	1	1	1	1	0
	0	0	2	0	1	0
FUT8	64	96	64	102	60	101
	61	139	64	43	74	20
FUT9	50	2	45	7	31	11
	0	2	39	1	23	0
FUZ	26	67	4	25	19	37
	58	77	26	21	45	10
FXC1	52	272	76	147	110	232
	232	285	125	81	177	43

FXN	82	355	79	214	169	238
	251	304	128	119	176	68
FXR1	1404	8753	1743	5952	4456	7649
	5809	7620	3210	2675	5135	1404
FXR2	188	626	169	558	336	582
	459	902	383	270	486	143
FXYD1	103	408	42	196	161	282
	374	467	128	123	212	65
FXYD2	0	0	0	0	0	0
	0	1	0	0	0	0
FXYD3	7	1	4	0	3	0
	0	0	2	0	6	0
FXYD4	6	0	4	0	0	0
	0	0	2	0	2	0
FXYD5	13	42	12	13	19	16
	19	36	15	11	19	5
FXYD6	1	44	1	9	5	19
	29	51	6	9	17	3
FXYD6-FXYD2		5	6	9	8	4
	1	2	6	5	0	7
	0					
FXYD7	4	1	1	0	0	5
	1	3	2	0	6	0
FYB	50	76	36	35	34	31
	32	65	44	15	34	10
FYCO1	706	3302	851	3857	2105	2323
	3689	3945	1520	975	1939	862
FYN	58	185	55	128	89	134
	121	167	71	55	87	27
FYTTD1	350	1819	313	1291	833	1159
	1210	1831	696	519	1036	269
FZD1	30	54	25	88	41	33
	48	50	30	5	22	10
FZD10	10	4	6	6	5	4
	6	2	6	1	4	1
FZD2	14	3	7	14	2	2
	4	2	5	1	1	0
FZD3	112	214	93	178	123	203
	123	217	128	48	126	18
FZD4	259	991	171	718	614	848
	906	1388	756	254	591	325
FZD5	31	103	24	45	55	58
	82	243	73	26	81	23
FZD6	40	125	31	90	44	82
	82	114	50	35	42	13
FZD7	52	269	50	259	142	242
	342	258	166	63	223	41
FZD8	10	25	4	30	12	18
	15	33	18	5	20	1
FZD9	9	12	5	4	7	9
	8	32	11	5	9	6
FZR1	25	55	9	38	24	39
	37	67	44	21	48	11
G0S2	1179	2048	937	5652	2218	8811
	1847	483	629	633	336	391
G2E3	43	167	45	120	96	107
	121	225	98	49	101	16

G3BP1	273	1390	290	1158	845	1010
	1011	1656	650	473	803	255
G3BP2	426	2063	366	1357	1001	1189
	1334	2958	742	681	1132	357
G6PC	19	0	15	0	3	0
	0	0	11	0	5	0
G6PC2	19	0	19	0	3	0
	0	0	7	0	5	0
G6PC3	16	33	11	35	19	32
	41	36	32	12	28	11
G6PD	11	15	6	20	3	12
	8	20	2	2	9	3
GAA	32	75	33	70	42	73
	106	135	73	44	76	21
GAB1	296	824	197	588	494	470
	1349	1897	730	332	999	176
GAB2	68	91	41	84	65	109
	93	135	55	61	64	38
GAB3	42	60	15	33	52	51
	56	88	53	26	48	9
GAB4	16	0	7	0	4	0
	0	0	14	0	2	0
GABARAP	100	586	68	345	178	386
	481	649	247	122	273	76
GABARAPL1	129	350	70	267	234	258
	481	937	313	187	440	142
GABARAPL2	107	566	75	390	214	377
	563	667	206	118	275	79
GABBR1	44	89	44	65	59	53
	67	83	36	30	48	17
GABBR2	42	2	21	1	12	0
	6	4	18	4	8	2
GABPA	136	522	156	497	320	451
	484	685	302	148	384	92
GABPB1	46	101	36	69	58	62
	74	136	84	46	74	19
GABPB2	45	147	46	95	68	121
	64	178	75	60	106	30
GABRA1	31	1	28	0	9	0
	0	0	17	0	13	0
GABRA2	21	0	15	1	13	0
	0	3	14	0	8	0
GABRA3	28	1	7	1	9	0
	0	0	4	0	6	0
GABRA4	54	5	34	1	20	0
	1	1	34	0	22	1
GABRA5	21	0	7	0	3	1
	0	1	10	0	4	0
GABRA6	30	0	13	0	6	0
	0	0	11	0	6	0
GABRB1	26	0	6	0	4	0
	0	0	9	0	1	0
GABRB2	39	19	53	19	21	17
	7	29	32	3	28	2
GABRB3	41	1	32	2	10	6
	7	4	27	1	19	1
GABRD	6	0	5	0	0	0
	0	0	1	0	1	0

GABRE	21	31	12	12	10	11
	10	18	8	5	14	7
GABRG1	27	0	13	0	6	0
	0	0	16	0	9	0
GABRG2	28	0	12	0	3	0
	0	0	12	0	7	0
GABRG3	15	0	13	0	4	0
	0	1	7	0	3	0
GABRP	24	0	13	3	3	0
	0	0	15	0	1	0
GABRQ	19	0	7	0	0	0
	0	0	2	0	0	0
GABRR1	25	2	8	1	9	0
	0	0	14	0	7	0
GABRR2	20	8	6	7	9	6
	6	8	4	1	10	1
GAD1	29	0	15	0	11	0
	0	1	8	0	5	0
GAD2	28	0	12	0	4	0
	0	0	15	0	3	0
GADD45A	21	81	37	92	20	51
	64	42	49	18	26	14
GADD45B	10	28	10	43	25	27
	23	43	15	4	13	5
GADD45G	41	100	23	127	86	134
	127	159	148	23	83	27
GADD45GIP1	36	195	22	129	113	183
	265	343	123	73	125	33
GADL1	125	963	205	701	511	636
	681	598	330	96	459	28
GAGE1	19	0	2	0	2	0
	0	0	3	0	1	0
GAGE10	14	0	4	0	4	0
	0	0	6	0	0	0
GAGE12C	0	0	1	0	1	0
	0	0	2	0	1	0
GAGE12D	7	0	0	0	0	0
	0	0	2	0	0	0
GAGE12E	2	0	1	0	0	0
	0	0	4	0	0	0
GAGE12F	0	0	1	0	1	0
	0	0	2	0	0	0
GAGE12G	3	0	2	0	1	0
	0	0	0	0	0	0
GAGE12H	3	0	1	0	0	0
	0	0	3	0	0	0
GAGE12I	13	0	3	0	1	0
	0	0	8	0	0	0
GAGE12J	2	0	2	0	0	0
	0	0	0	0	0	0
GAGE13	8	0	1	0	0	0
	0	0	2	0	0	0
GAGE2A	9	0	1	0	0	0
	0	0	5	0	1	0
GAGE2B	1	0	6	0	3	0
	0	0	1	0	1	0
GAGE2C	0	0	2	0	1	0
	0	0	4	0	0	0

GAGE2D	2	0	2	0	0	0
	0	0	0	0	1	0
GAGE2E	0	0	1	0	1	0
	0	0	1	0	1	0
GAK	54	143	49	173	67	173
	138	189	84	47	73	39
GAL	5	0	1	0	0	0
	0	0	2	0	2	0
GAL3ST1	6	1	2	1	5	4
	0	6	3	0	2	3
GAL3ST2	1	1	0	5	1	1
	0	0	3	0	0	0
GAL3ST3	12	2	0	1	5	4
	3	0	8	0	2	0
GAL3ST4	14	7	11	3	5	6
	1	10	12	1	4	5
GALC	65	208	70	202	101	135
	147	195	122	63	116	39
GALE	8	19	5	6	11	2
	15	12	9	5	9	5
GALK1	5	9	3	11	6	8
	3	16	6	4	15	0
GALK2	69	229	56	131	139	152
	191	294	105	79	148	42
GALM	42	92	18	91	61	70
	94	83	58	29	65	19
GALNS	12	16	8	11	14	8
	10	21	9	10	7	7
GALNT1	187	793	186	652	501	724
	618	867	386	299	479	160
GALNT10	55	92	50	78	57	86
	65	89	61	34	72	28
GALNT11	102	384	95	274	192	368
	271	309	186	98	172	60
GALNT12	20	27	14	37	12	26
	26	34	23	4	32	2
GALNT13	52	4	33	6	13	2
	0	0	22	0	11	0
GALNT14	27	1	16	1	14	0
	1	0	19	0	5	0
GALNT2	100	507	106	487	234	342
	334	389	195	144	295	108
GALNT3	17	8	19	5	9	2
	5	3	12	2	10	3
GALNT4	0	0	0	0	0	0
	0	0	0	0	0	1
GALNT5	30	4	10	5	8	3
	0	2	19	0	10	0
GALNT6	31	1	10	1	2	2
	1	8	7	1	14	0
GALNT7	27	40	26	52	25	35
	39	37	36	11	25	3
GALNT8	23	7	23	1	4	2
	3	1	23	0	10	1
GALNT9 (NC_000012 132680916..132690573)				3	0	7
	0	2	0	0	0	1
	0	1	0			

GALNT9 (NC_000012 132824459..132905905)			1	0	1	
	0	2	0	0	0	5
	0	1	0			
GALNTL1	34	60	30	41	47	72
	25	55	26	9	37	22
GALNTL2	48	128	17	125	105	130
	76	292	100	35	123	57
GALNTL4	27	57	11	33	19	35
	37	70	42	13	44	11
GALNTL5	18	0	8	0	4	0
	0	0	6	0	3	0
GALNTL6	18	25	20	2	11	9
	0	0	20	1	11	1
GALP	5	0	4	0	2	0
	0	0	2	0	5	0
GALR1	3	1	6	2	4	0
	0	3	4	0	1	0
GALR2	5	0	2	0	0	0
	0	1	0	0	0	0
GALR3	0	0	0	0	0	0
	0	1	0	0	0	0
GALT	51	138	27	86	54	70
	93	175	77	30	70	13
GAMT	164	844	139	812	284	570
	705	842	460	191	376	100
GAN	58	147	61	95	76	134
	109	177	76	50	99	34
GANAB	77	273	59	193	141	242
	233	318	140	76	127	49
GANC	154	613	178	494	288	491
	380	616	344	166	344	109
GAP43	4	1	7	0	6	0
	0	2	5	0	5	1
GAPDH	7360	46904	5919	33566	14504	30527
	51950	24754	22561	11847	20204	4942
GAPDHS	3	1	2	0	0	0
	0	2	4	1	1	0
GAPT	18	4	16	4	12	6
	2	3	14	4	4	0
GAPVD1	182	848	194	659	435	665
	636	812	429	279	479	142
GAR1	14	13	11	9	5	8
	7	13	8	3	5	0
GARNL3	38	62	43	67	41	46
	39	101	70	19	54	18
GARS	100	370	71	274	177	235
	259	370	163	102	205	67
GART	79	267	85	147	128	223
	228	394	174	88	188	50
GAS1	28	95	27	104	77	119
	38	178	64	27	65	27
GAS2	92	221	64	154	142	202
	194	355	114	89	180	52
GAS2L1	15	11	4	7	6	8
	10	14	5	1	8	1
GAS2L2	10	26	7	7	6	7
	7	11	8	5	7	1

GAS2L3	18	11	14	9	15	13
	6	14	17	4	12	4
GAS6	59	254	48	170	105	123
	210	361	153	73	143	45
GAS7	98	226	57	225	156	177
	115	352	157	73	154	54
GAS8	44	167	38	70	80	89
	80	89	60	44	71	11
GAST	1	0	0	0	0	0
	0	0	0	0	0	0
GATA1	3	0	1	0	0	0
	0	0	0	0	0	0
GATA2	18	34	13	12	7	18
	31	26	12	6	25	9
GATA3	24	18	3	11	17	10
	11	18	9	5	9	2
GATA4	16	0	8	0	1	0
	0	1	6	0	2	0
GATA5	12	0	6	0	0	0
	0	0	4	0	0	0
GATA6	17	3	3	3	4	1
	1	0	7	0	5	2
GATAD1	88	334	109	200	130	217
	227	429	207	126	191	79
GATAD2A	37	153	25	67	60	67
	76	94	54	33	57	22
GATAD2B	99	553	115	390	217	415
	320	404	171	95	182	76
GATC	61	224	65	109	95	159
	165	254	94	80	107	37
GATM	199	486	126	477	392	357
	419	790	211	181	330	123
GATS	149	448	97	654	219	425
	505	771	374	263	554	141
GATSL1	31	102	18	83	63	76
	96	99	34	62	81	35
GATSL2	11	21	3	23	20	25
	15	12	7	5	8	5
GATSL3	8	5	5	5	5	0
	1	11	7	0	1	2
GBA	22	38	22	21	28	21
	23	34	26	18	23	8
GBA2	128	481	98	300	192	332
	495	807	220	184	348	62
GBA3	12	0	6	1	2	0
	0	1	6	0	3	0
GBAS	674	3453	559	3052	1648	2807
	4511	3978	1706	1083	2579	559
GBE1	356	1486	331	1435	738	1321
	1040	1391	760	434	1038	270
GBF1	256	1117	265	817	509	872
	967	1250	546	327	818	256
GBGT1	6	10	7	10	10	10
	10	26	8	6	11	5
GBP1	42	106	43	100	55	59
	49	93	69	53	95	17
GBP2	67	226	90	188	106	118
	184	332	171	119	211	74

GBP3	33	77	26	16	23	28
	28	47	42	7	47	12
GBP4	89	245	55	144	123	153
	168	162	183	114	228	54
GBP5	70	17	41	15	27	17
	20	38	49	17	32	4
GBP6	37	3	27	2	3	1
	0	1	24	2	14	0
GBP7	13	2	13	2	6	0
	2	1	8	0	7	0
GBX1	16	56	14	11	14	38
	36	31	5	13	13	5
GBX2	1	0	4	1	4	0
	0	1	1	0	1	0
GC	24	0	16	0	9	0
	0	0	5	0	7	0
GCA	28	80	42	83	31	55
	78	60	66	16	42	11
GCAT	19	47	9	27	20	20
	43	52	42	18	24	4
GCC1	30	105	28	81	52	77
	56	105	49	34	69	26
GCC2	113	572	120	430	303	446
	369	523	228	182	322	113
GCDH	16	83	13	35	36	29
	63	106	54	21	54	17
GCFC1	107	392	103	223	168	245
	274	435	160	118	224	60
GCFC2	124	495	154	453	263	481
	607	827	384	178	426	111
GCG	20	0	7	0	2	0
	0	0	5	0	4	0
GCGR	2	0	4	0	0	1
	0	0	4	0	5	0
GCH1	14	14	8	19	10	8
	3	14	13	3	9	1
GCHFR	3	1	0	0	0	3
	4	2	1	1	2	1
GCK	17	0	11	0	2	0
	0	0	2	0	5	0
GCKR	27	2	9	0	1	0
	1	0	8	0	7	0
GCLC	59	191	39	87	115	105
	143	243	85	63	107	42
GCLM	44	141	36	162	66	107
	165	366	141	47	126	35
GCM1	17	0	9	0	3	0
	0	0	4	0	1	0
GCM2	12	0	6	0	2	0
	0	0	4	0	3	0
GCN1L1	202	899	179	644	511	690
	626	992	414	289	523	187
GCNT1	54	32	12	31	22	30
	31	44	37	9	46	5
GCNT2	79	45	28	12	122	13
	27	116	30	31	20	35
GCNT3	14	0	5	2	4	0
	0	0	8	0	4	0

GCNT4	3	3	0	1	3	1
	0	0	1	3	2	2
GCNT7	14	2	5	0	8	1
	0	0	12	1	2	0
GCOM1	157	219	109	245	226	384
	172	148	215	150	230	57
GCSAM	16	0	16	1	5	0
	0	0	12	0	2	0
GCSAML	15	1	12	6	10	1
	4	4	10	1	8	3
GCSH	94	578	45	339	149	350
	702	637	268	118	314	78
GDA	49	99	67	78	74	33
	108	49	79	11	35	5
GDAP1	45	116	66	100	54	115
	105	161	73	43	114	37
GDAP1L1	19	0	11	0	1	0
	1	1	7	0	7	0
GDAP2	131	372	116	325	202	298
	318	474	203	119	240	91
GDE1	113	631	119	587	270	494
	640	701	310	151	355	117
GDF1	2	2	3	7	2	9
	7	4	4	5	5	0
GDF10	9	37	7	20	28	37
	5	20	7	6	13	10
GDF11	14	14	16	11	10	9
	17	21	17	3	6	0
GDF15	0	0	1	0	0	1
	0	1	1	0	1	0
GDF2	11	0	5	0	2	0
	0	0	1	0	1	0
GDF3	6	0	2	1	2	0
	0	1	1	0	2	0
GDF5	1	0	3	0	0	0
	0	0	1	0	0	0
GDF6	18	0	7	0	4	0
	0	0	5	0	5	0
GDF7	2	0	1	0	1	0
	0	0	1	0	0	0
GDF9	15	5	7	12	2	9
	2	2	4	0	7	3
GDI1	33	183	35	117	84	109
	138	194	68	50	103	47
GDI2	224	1225	223	850	521	935
	1039	1514	635	417	731	269
GDNF	41	67	38	114	84	190
	282	31	75	29	52	21
GDPD1	21	33	10	18	17	42
	27	41	38	9	23	1
GDPD2	9	2	7	0	5	0
	0	0	4	0	2	0
GDPD3	7	13	12	5	6	14
	9	4	2	2	11	1
GDPD4	33	1	12	0	8	0
	0	1	9	0	12	0
GDPD5	20	24	11	27	11	35
	23	27	22	10	17	2

GDPGP1	15	3	3	5	4	3
	4	6	9	1	6	1
GEM	6	37	14	25	14	11
	6	18	9	10	11	3
GEMIN2	19	83	16	56	32	42
	63	128	42	11	52	13
GEMIN4	28	36	9	36	37	41
	40	42	27	13	33	8
GEMIN5	105	313	98	273	222	279
	223	343	182	102	214	68
GEMIN6	9	53	14	30	20	36
	59	83	36	20	25	12
GEMIN7	17	42	10	28	26	23
	35	47	17	8	16	7
GEMIN8	19	25	6	18	20	34
	24	46	19	11	21	2
GEN1	75	144	61	141	87	125
	126	177	101	55	133	32
GET4	25	138	42	97	69	71
	86	122	68	29	79	25
GFAP	16	0	19	0	2	0
	0	0	10	0	4	0
GFER	18	64	18	39	21	44
	49	86	29	26	38	18
GFI1	10	0	15	1	2	2
	2	0	5	0	3	1
GFI1B	9	0	5	0	2	0
	2	0	4	1	1	0
GFM1	232	1364	255	1063	582	866
	1089	1692	638	450	978	192
GFM2	141	564	141	365	273	332
	460	703	296	176	410	98
GFOD1	46	151	29	95	117	119
	141	213	113	49	157	46
GFOD2	41	70	36	59	26	53
	61	68	43	19	32	8
GFPT1	173	926	209	729	481	730
	647	884	335	243	465	123
GFPT2	22	36	21	47	37	40
	19	72	26	8	43	15
GFRA1	102	222	58	149	165	231
	178	422	189	111	177	72
GFRA2	16	2	7	6	10	3
	0	15	7	2	8	2
GFRA3	8	0	4	0	1	0
	0	0	7	0	3	0
GFRA4	2	0	0	0	0	0
	0	0	2	0	0	0
GFRAL	9	0	6	0	9	0
	0	0	12	0	5	0
GGA1	55	147	48	103	52	117
	91	210	85	51	102	31
GGA2	98	362	77	281	149	286
	313	341	148	105	192	67
GGA3	43	139	38	101	73	91
	119	166	82	44	91	27
GGACT	25	33	16	17	15	22
	29	32	25	15	25	3

GGCT	45	241	35	164	79	168
	213	289	99	51	106	30
GGCX	109	241	75	165	115	184
	145	264	122	67	136	43
GGH	16	18	24	15	22	15
	15	24	20	9	15	2
GGN	4	0	3	1	1	0
	0	0	1	0	2	0
GGNBP2	77	405	82	297	168	226
	284	454	155	134	214	93
GGPS1	74	260	47	208	133	241
	186	247	121	81	145	44
GGT1	30	6	20	5	7	4
	4	11	14	5	5	1
GGT2	13	0	5	0	2	0
	0	0	7	0	2	0
GGT5	19	28	13	10	7	9
	12	33	18	11	19	8
GGT6	12	1	6	0	2	1
	0	6	7	1	1	1
GGT7	18	76	54	175	23	153
	250	113	88	20	124	37
GGTLC1	9	0	3	0	0	0
	0	0	1	0	1	0
GGTLC2	0	0	1	0	1	0
	0	0	1	0	0	1
GH1	3	1	1	1	1	0
	0	0	2	0	1	0
GH2	1	1	1	0	0	0
	0	0	2	0	1	0
GHDC	14	17	6	14	8	31
	12	27	27	6	16	3
GHITM	607	3623	542	2404	1478	2158
	4428	4411	1675	909	2151	461
GHR	310	1306	276	1075	708	1038
	1247	1632	712	481	822	294
GHRH	4	0	0	0	2	0
	0	0	1	0	1	0
GHRHR	9	1	8	0	0	0
	0	0	5	0	2	0
GHRL	3	1	2	1	4	0
	0	0	2	1	4	2
GHSR	14	0	8	0	3	0
	0	0	4	0	4	0
GID4	58	246	60	258	128	278
	187	217	126	73	116	42
GID8	49	185	50	170	81	139
	157	218	93	53	142	27
GIF	11	0	11	3	2	0
	0	0	6	0	2	0
GIGYF1	82	279	56	150	108	166
	259	380	129	98	187	77
GIGYF2	357	1580	387	1014	831	1161
	1125	1500	656	530	860	283
GIMAP1	2	11	1	13	1	4
	6	23	2	6	11	5

GIMAP1-GIMAP5	28	103	36	65	45	55	105	159	84	41	91	15
GIMAP2	10	48	6	15	8	16	32	39	18	11	29	8
GIMAP4	31	163	15	73	73	87	104	178	87	84	109	35
GIMAP5	0	16	0	13	2	4	11	22	1	6	5	2
GIMAP6	44	207	36	141	84	118	186	238	129	73	109	32
GIMAP7	19	108	15	62	36	61	70	122	48	42	67	22
GIMAP8	55	190	32	117	93	91	182	186	127	112	155	49
GIMD1	5	0	1	0	0	0	0	0	0	0	1	0
GIN1	58	262	51	174	165	176	165	329	109	84	147	43
GINM1	54	194	43	147	71	153	164	216	120	76	102	53
GINS1	15	8	17	4	14	9	5	10	16	4	14	3
GINS2	2	11	10	12	8	12	14	16	11	1	13	3
GINS3	17	12	9	8	3	12	17	28	14	6	16	2
GINS4	23	4	11	4	3	3	1	1	17	1	8	1
GIP	6	0	2	2	1	0	0	0	0	0	0	0
GIPC1	16	67	19	45	27	38	46	73	30	14	34	14
GIPC2	13	35	14	19	19	23	33	35	21	16	26	4
GIPC3	8	8	6	2	6	4	6	2	9	2	4	2
GIPR	12	0	7	0	3	1	0	0	12	0	2	0
GIT1	45	115	24	73	50	59	86	81	58	32	42	17
GIT2	118	461	88	286	202	285	300	380	202	117	223	78
GJA1	59	214	40	148	97	118	120	174	84	83	84	30
GJA10	3	0	0	1	1	0	0	0	0	0	0	0
GJA3	17	0	7	0	2	0	0	0	8	0	3	0
GJA4	21	36	3	7	11	13	19	28	13	4	5	3
GJA5	19	16	11	12	23	4	27	30	13	7	10	6
GJA8	7	0	4	0	1	0	0	0	8	0	2	0
GJA9	12	1	13	2	4	1	2	6	18	0	5	0

GJB1	3	0	1	0	0	0
	0	0	0	0	1	0
GJB2	10	1	5	3	1	1
	0	1	4	1	3	1
GJB3	4	0	4	0	0	0
	0	1	4	0	3	0
GJB4	12	0	7	0	2	0
	0	0	9	0	1	0
GJB5	5	0	4	0	1	0
	0	0	0	0	2	0
GJB6	13	1	10	0	3	0
	0	0	10	0	3	0
GJB7	22	0	8	0	6	0
	0	0	12	0	8	0
GJC1	68	140	27	99	96	80
	100	119	92	48	63	25
GJC2	4	1	2	0	1	3
	0	0	0	0	0	0
GJC3	8	2	3	1	2	0
	2	1	4	0	1	0
GJD2	6	0	6	0	0	0
	0	0	7	0	3	0
GJD3	6	3	3	5	2	1
	2	2	6	1	9	3
GJD4	0	1	5	0	1	0
	0	0	0	0	1	0
GK	62	78	32	50	33	45
	61	138	58	19	65	16
GK2	10	1	3	0	2	0
	0	0	7	0	1	0
GK5	84	330	124	286	173	304
	263	313	197	109	207	75
GKAP1	78	427	72	277	220	335
	397	461	156	145	234	50
GKN1	8	0	5	0	1	0
	0	0	7	0	1	0
GKN2	12	0	3	1	1	0
	0	0	4	0	1	0
GLA	17	34	8	37	18	29
	24	44	24	4	23	6
GLB1	44	65	23	58	34	67
	49	110	64	13	66	15
GLB1L	17	14	8	12	8	4
	6	20	19	3	11	2
GLB1L2	13	4	12	2	5	2
	1	7	4	2	7	0
GLB1L3	30	0	7	0	3	0
	0	0	9	0	7	0
GLCCI1	48	94	37	68	38	58
	99	60	54	26	31	19
GLCE	75	208	82	169	115	147
	149	260	123	70	156	44
GLDC	27	8	23	7	17	8
	16	17	30	2	16	6
GLDN	44	16	28	23	9	7
	13	14	38	14	19	14
GLE1	83	306	66	171	136	171
	212	308	135	94	178	41

GLG1	255	1433	395	1130	756	1095
	1169	1164	576	350	648	280
GLI1	24	10	8	20	2	3
	3	5	9	2	2	2
GLI2	30	24	18	17	17	9
	21	15	20	7	16	9
GLI3	37	59	34	67	31	44
	19	48	34	10	36	8
GLI4	5	6	3	4	1	6
	5	17	7	5	5	2
GLIPR1	24	51	16	56	40	66
	33	84	35	11	36	20
GLIPR1L1	7	1	3	0	4	3
	0	1	4	1	1	0
GLIPR1L2	17	7	30	4	16	5
	1	6	20	3	11	2
GLIPR2	7	34	8	30	15	32
	17	18	21	3	22	9
GLIS1	14	0	4	0	3	0
	0	0	6	0	0	0
GLIS2	13	13	5	5	5	10
	11	5	13	0	8	3
GLIS3	37	25	37	29	18	16
	33	37	27	9	25	13
GLMN	36	57	18	81	36	61
	49	74	42	21	63	14
GLO1	209	846	154	696	488	811
	892	1315	552	334	667	196
GLOD4	65	188	53	185	81	170
	234	246	118	74	139	42
GLOD5	8	0	4	0	0	0
	0	0	2	0	1	0
GLP1R	14	0	7	0	2	0
	0	0	9	0	2	0
GLP2R	14	0	6	0	4	0
	0	1	9	0	5	1
GLRA1	17	0	3	0	1	0
	0	2	7	0	3	0
GLRA2	31	0	5	0	4	0
	0	0	10	0	0	0
GLRA3	15	0	9	1	7	0
	0	1	9	0	3	0
GLRA4	17	1	8	0	3	0
	0	0	5	0	0	0
GLRB	23	32	35	30	28	37
	52	111	50	20	54	13
GLRX	87	312	61	351	171	356
	339	270	172	60	96	39
GLRX2	19	58	19	48	32	37
	67	104	45	18	35	11
GLRX3	47	247	42	146	100	163
	205	283	109	58	121	38
GLRX5	67	538	48	372	168	369
	519	717	228	84	267	77
GLS	143	776	173	537	350	497
	498	779	323	166	301	145
GLS2	18	2	12	1	5	0
	2	2	13	0	3	0

GLT1D1	32	40	11	12	10	33
	34	25	22	6	22	12
GLT25D1	32	122	26	81	44	86
	63	122	51	24	54	16
GLT25D2	118	187	61	140	119	155
	138	149	77	56	128	43
GLT6D1	12	0	9	0	0	0
	0	0	11	0	2	0
GLT8D1	43	102	31	109	68	111
	115	142	77	38	62	19
GLT8D2	16	25	16	31	20	12
	24	45	24	7	20	2
GLTP	32	117	35	100	45	84
	117	141	62	33	55	20
GLTPD1	40	149	16	69	64	67
	90	106	55	26	63	6
GLTPD2	0	0	2	0	0	0
	0	0	2	0	0	0
GLTSCR1	9	14	10	18	11	9
	9	13	5	10	11	5
GLTSCR2	79	329	83	352	133	473
	435	312	239	135	229	79
GLUD1	113	537	73	467	227	385
	484	698	333	176	338	114
GLUD2	5	7	7	3	7	4
	6	5	3	1	3	2
GLUL	531	3072	653	1549	1550	1296
	3355	11806	2912	814	4351	1436
GLYAT	11	0	5	0	7	0
	4	0	8	0	4	3
GLYATL1	26	1	17	2	4	1
	0	0	18	0	9	0
GLYATL2	10	3	6	1	4	11
	0	0	6	0	7	0
GLYATL3	11	0	4	0	4	0
	0	0	7	0	1	0
GLYCTK	17	20	8	10	4	12
	11	17	16	2	16	2
GLYR1	114	436	81	279	230	269
	308	482	212	131	250	109
GM2A	44	142	43	93	76	87
	85	108	55	41	64	21
GMCL1	20	72	24	47	24	54
	51	80	32	12	52	9
GMDS	26	23	21	12	11	11
	24	14	23	11	19	0
GMEB1	45	76	32	48	41	60
	56	59	49	26	45	13
GMEB2	27	43	15	26	34	33
	43	60	27	21	22	9
GMFB	66	232	66	172	102	178
	154	286	122	63	110	39
GMFG	12	29	6	22	10	15
	27	40	9	8	16	3
GMIP	19	6	10	6	5	8
	2	6	5	3	8	4
GML	4	0	1	0	2	0
	0	0	2	0	3	0

GMNC	33	2	12	0	5	0
	2	6	20	1	4	0
GMNN	14	33	14	19	25	19
	58	43	28	7	35	10
GMPPA	7	28	7	27	16	19
	27	37	15	8	24	3
GMPPB	8	18	8	24	10	24
	16	34	13	13	11	4
GMPR	171	944	111	692	391	524
	1085	1071	485	316	549	168
GMPR2	58	221	50	177	88	203
	228	289	137	59	140	36
GMPS	55	273	49	172	120	191
	219	253	117	91	109	39
GNA11	27	158	36	89	72	100
	103	138	60	34	73	27
GNA12	19	43	14	32	32	32
	32	40	28	19	22	15
GNA13	114	517	138	536	318	433
	388	526	208	142	263	108
GNA14	19	10	5	17	9	11
	4	14	20	5	10	1
GNA15	9	3	3	3	1	2
	1	4	4	1	2	0
GNAI1	27	140	20	89	91	91
	87	138	72	34	51	20
GNAI2	68	245	50	155	151	180
	186	267	137	118	145	61
GNAI3	86	304	83	199	145	221
	256	364	180	93	174	73
GNAL	77	221	58	224	202	260
	189	238	147	88	146	58
GNAO1	34	22	23	9	30	17
	15	16	25	9	17	4
GNAQ	35	162	31	80	66	117
	138	144	90	47	65	36
GNAS	610	3813	573	2535	1542	2136
	3317	2903	1158	1011	1754	526
GNAT1	11	0	2	1	2	0
	0	0	7	0	0	0
GNAT2	17	2	10	1	1	0
	0	0	6	0	4	0
GNAT3	11	0	4	0	2	0
	0	3	7	0	6	0
GNAZ	11	22	7	16	4	29
	18	21	16	4	12	3
GNB1	78	387	70	279	164	236
	277	345	128	99	152	101
GNB1L	1	8	5	2	1	3
	1	9	9	2	6	0
GNB2	21	66	23	66	42	45
	85	82	54	23	30	21
GNB2L1	657	3371	676	2379	1482	3069
	4117	5596	2110	1209	1965	699
GNB3	8	3	8	0	1	3
	0	0	5	0	3	0
GNB4	70	188	52	139	90	112
	102	218	89	59	104	41

GNB5	24	8	8	13	11	15
	17	20	21	6	11	5
GNE	72	197	51	140	116	125
	148	238	107	62	127	53
GNG10	4	77	4	90	17	69
	162	126	28	18	36	22
GNG11	19	137	16	91	62	52
	148	231	78	47	62	33
GNG12	76	371	83	344	214	336
	295	396	219	109	191	88
GNG13	6	0	2	0	0	1
	0	0	0	0	0	0
GNG2	42	83	34	91	53	41
	57	64	61	22	49	23
GNG3	5	0	3	0	3	0
	1	0	4	0	2	1
GNG4	25	1	5	0	5	2
	1	1	9	0	3	0
GNG5	19	133	11	84	42	44
	200	148	50	48	47	19
GNG7	16	7	5	2	4	1
	12	18	6	3	10	5
GNG8	1	0	0	0	2	0
	0	0	0	0	0	0
GNGT1	2	0	1	0	4	0
	0	0	1	0	1	0
GNGT2	5	0	2	0	0	0
	1	0	5	1	4	0
GNL1	55	141	61	148	87	117
	126	123	85	48	90	24
GNL2	81	268	73	167	135	231
	209	338	160	105	181	72
GNL3	111	564	112	321	263	387
	426	633	251	207	261	98
GNL3L	126	456	72	256	191	272
	286	357	168	127	194	56
GNLY	3	7	2	4	3	2
	4	12	8	5	7	1
GNMT	5	0	1	1	2	1
	2	1	3	1	2	1
GNPAT	249	1118	206	740	485	863
	885	1449	502	316	622	157
GNPDA1	45	107	31	100	53	115
	90	164	86	34	92	21
GNPDA2	28	122	31	102	49	106
	102	140	65	30	86	31
GNPNAT1	33	155	27	121	80	134
	147	102	66	49	71	21
GNPTAB	156	698	127	455	299	368
	583	547	287	175	257	106
GNPTG	27	104	21	65	50	75
	93	177	52	34	77	13
GNRH1	15	25	11	29	14	20
	18	49	16	11	37	11
GNRH2	5	0	0	1	0	0
	0	2	1	0	1	0
GNRHR	28	4	19	2	14	2
	1	0	13	5	6	1

GNS	183	757	161	665	352	602
	565	923	476	253	501	167
GOLGA1	110	426	95	219	177	283
	337	351	145	123	214	68
GOLGA2	166	446	104	292	284	300
	343	379	185	148	217	80
GOLGA3	209	708	154	504	308	397
	425	593	306	196	339	166
GOLGA4	809	5814	839	3967	2763	4921
	3320	4173	1793	2249	2658	1033
GOLGA5	58	291	72	230	142	249
	252	248	139	99	148	53
GOLGA6A	39	5	46	6	7	4
	4	7	15	0	19	0
GOLGA6B	15	2	19	7	8	0
	6	1	15	0	14	0
GOLGA6C	121	58	61	44	32	62
	46	33	77	3	72	6
GOLGA6D	25	16	8	15	5	1
	20	1	22	0	4	2
GOLGA6L1	85	0	28	0	7	1
	3	0	52	0	19	0
GOLGA6L10 (NC_000015 82633121..82641706)				35	30	21
	25	5	42	14	64	33
	5	21	7			
GOLGA6L10 (NC_000015 83188578..83195217)				5	5	4
	3	1	5	5	5	3
	4	3	5			
GOLGA6L2	13	0	10	0	7	0
	0	0	13	0	9	0
GOLGA6L3	43	12	11	1	14	17
	6	9	17	4	18	1
GOLGA6L4	22	57	14	35	46	22
	63	147	33	27	48	22
GOLGA6L6	44	1	32	0	9	0
	0	1	18	0	12	0
GOLGA6L9	53	115	62	135	119	174
	126	235	89	73	74	20
GOLGA7	43	164	43	135	85	104
	163	178	80	51	91	37
GOLGA7B	20	27	17	15	15	18
	15	27	25	6	30	6
GOLGA8A	93	123	92	210	210	270
	201	212	161	106	159	82
GOLGA8B	76	307	126	315	72	313
	347	511	336	99	224	114
GOLGA8H	36	12	18	4	16	2
	1	5	19	2	16	4
GOLGA8J	10	0	7	3	2	0
	0	0	6	0	1	0
GOLGA8K	10	7	20	2	2	9
	3	4	9	0	11	2
GOLGA8M	99	104	43	64	59	50
	71	97	79	29	79	23
GOLGA8O	46	158	43	64	73	41
	133	97	59	22	147	30
GOLGA8R	26	11	12	10	7	19
	8	59	36	14	7	8

GOLGB1	343	1705	437	1390	926	1347
	1203	1531	615	563	864	446
GOLIM4	141	732	123	349	422	561
	434	795	238	289	373	141
GOLM1	33	64	27	59	21	49
	35	51	40	10	23	14
GOLPH3	62	239	58	205	119	205
	305	405	184	83	179	39
GOLPH3L	51	223	49	127	117	97
	174	211	78	60	100	38
GOLT1A	6	0	3	0	2	0
	0	0	4	0	1	0
GOLT1B	47	256	58	205	126	196
	130	312	112	67	127	32
GON4L	163	643	156	323	302	374
	372	563	253	202	344	116
GOPC	119	547	153	438	291	390
	399	632	301	170	361	83
GORAB	17	39	20	38	28	30
	25	40	41	12	23	12
GORASP1	35	89	24	57	43	54
	46	141	65	34	43	12
GORASP2	55	280	37	168	122	168
	219	270	124	86	157	63
GOSR1	97	275	115	243	134	195
	251	310	155	112	190	70
GOSR2	75	265	55	172	126	152
	200	316	148	61	143	52
GOT1	363	2465	212	1702	770	1019
	2413	3012	1038	542	1546	302
GOT1L1	13	0	5	0	1	0
	0	0	13	0	3	0
GOT2	554	3563	368	3086	1342	2068
	4814	3441	1456	940	1938	448
GP1BA	8	5	4	1	2	0
	2	2	4	1	6	0
GP1BB	1	2	0	0	0	0
	1	3	1	1	0	0
GP2	19	0	7	0	4	0
	0	0	7	0	1	0
GP5	11	0	2	0	3	0
	0	1	3	1	5	0
GP6	11	3	13	0	0	1
	0	5	6	0	1	2
GP9	2	0	1	1	0	0
	0	0	0	0	0	0
GPA33	14	5	14	1	8	4
	2	15	5	5	7	2
GPAA1	16	69	6	39	6	32
	41	51	31	17	35	14
GPAM	200	806	171	361	533	435
	534	878	396	212	491	385
GPANK1	50	132	33	56	58	78
	102	165	54	49	71	35
GPAT2	28	22	22	13	14	11
	8	29	22	6	18	3
GPATCH1	51	119	42	72	62	76
	112	137	62	34	71	19

GPATCH2	44	155	42	76	62	76
	78	144	53	32	68	25
GPATCH2L	96	403	108	345	217	345
	308	445	220	108	262	68
GPATCH3	22	53	21	46	41	57
	50	79	34	25	55	9
GPATCH4	34	121	27	49	33	49
	75	84	26	39	38	11
GPATCH8	255	1200	279	712	528	909
	1166	1536	558	480	735	312
GPBAR1	3	0	2	0	2	2
	0	0	1	0	1	1
GPBP1	248	1301	301	988	705	987
	1105	1462	611	439	715	262
GPBP1L1	209	927	207	743	486	687
	882	1051	499	274	560	171
GPC1	79	370	92	252	166	204
	221	392	202	147	170	76
GPC2	8	0	7	1	7	0
	1	1	8	0	1	1
GPC3	25	28	15	57	22	5
	16	29	15	6	7	16
GPC4	52	331	62	224	134	194
	328	171	80	42	86	28
GPC5	12	1	13	1	10	2
	1	5	12	0	9	2
GPC6	50	27	35	63	33	33
	17	40	39	11	47	25
GPCPD1	100	407	100	274	154	301
	410	840	390	162	329	116
GPD1	561	4033	854	3614	1428	3973
	3182	2248	1768	697	1549	381
GPD1L	371	1569	333	867	801	1066
	1874	3019	902	523	1392	474
GPD2	301	1640	550	2077	1144	1382
	1149	1233	783	383	698	215
GPER	10	21	10	11	6	5
	12	7	8	3	3	3
GPHA2	2	0	2	0	0	0
	0	0	0	0	0	0
GPHB5	3	0	1	0	1	0
	0	0	2	0	0	0
GPHN	60	257	98	244	125	196
	250	263	151	82	180	46
GPI	253	2110	238	1859	724	1332
	2252	1262	945	563	972	253
GPIHBP1	18	82	7	20	40	27
	123	88	33	35	43	8
GPKOW	55	159	30	122	84	100
	116	203	90	48	133	45
GPLD1	73	72	70	138	58	121
	96	75	58	43	46	22
GPM6A	19	2	15	2	7	0
	1	7	20	0	14	1
GPM6B	21	20	19	26	19	18
	20	28	20	18	28	11
GPN1	64	181	57	128	97	175
	173	245	105	72	154	31

GPN2	12	39	15	27	20	21
	33	47	26	7	32	10
GPN3	44	321	60	285	110	210
	337	326	200	76	193	52
GPNMB	68	248	61	468	122	199
	174	386	167	53	233	57
GPR1	15	10	5	8	15	19
	10	13	8	2	11	7
GPR101	8	0	2	0	0	1
	0	0	0	0	0	0
GPR107	104	455	105	319	172	320
	312	393	191	145	238	73
GPR108	20	36	20	22	22	27
	33	59	16	14	27	12
GPR110	26	0	15	0	15	0
	0	0	17	0	10	1
GPR111	43	24	31	41	15	0
	29	14	11	5	33	10
GPR112	91	12	38	2	22	3
	3	18	23	1	18	0
GPR113	19	0	11	2	5	0
	4	0	17	1	6	1
GPR114	20	1	5	0	2	0
	1	1	7	0	3	0
GPR115	18	7	18	20	5	2
	11	17	12	3	18	1
GPR116	194	770	120	366	409	385
	956	1015	556	406	639	151
GPR119	9	0	2	0	0	0
	0	0	3	0	0	0
GPR12	23	7	6	2	6	3
	1	6	9	0	6	1
GPR123	12	0	5	0	1	0
	0	0	1	0	3	0
GPR124	36	129	21	98	50	54
	79	112	73	19	66	26
GPR125	194	716	194	489	361	522
	662	1146	354	243	527	127
GPR126	67	39	44	61	40	26
	37	58	73	8	35	28
GPR128	22	0	11	1	5	0
	0	1	9	0	8	0
GPR132	18	1	7	7	3	3
	3	14	13	0	7	1
GPR133	45	84	21	82	26	82
	68	83	50	26	70	15
GPR135	8	14	5	4	5	8
	11	8	8	5	7	0
GPR137	13	32	10	13	10	23
	8	11	12	5	13	3
GPR137B	14	12	7	13	14	11
	3	23	15	8	20	3
GPR137C	20	13	13	13	11	11
	12	16	17	7	14	1
GPR139	4	0	2	0	4	0
	0	0	3	0	4	0
GPR141	0	3	1	1	1	1
	2	0	0	0	0	0

GPR142	4	0	3	0	0	0
	0	0	3	0	1	0
GPR143	11	0	5	0	11	0
	0	0	1	0	2	0
GPR144	6	0	4	0	3	0
	0	0	8	0	0	0
GPR146	13	18	1	10	14	8
	16	20	8	7	16	6
GPR148	2	0	0	0	0	0
	0	0	1	0	0	0
GPR149	8	0	8	0	3	0
	0	0	9	0	2	0
GPR15	9	0	8	0	2	0
	0	4	2	0	1	0
GPR150	1	0	0	0	0	0
	0	2	0	0	1	0
GPR151	5	0	3	0	0	1
	0	1	3	0	1	0
GPR152	0	0	3	0	1	0
	0	0	3	0	0	0
GPR153	18	15	2	3	3	7
	1	3	7	1	2	1
GPR155	123	363	79	307	169	350
	234	324	197	101	205	54
GPR156	18	2	17	1	2	10
	0	0	15	0	7	0
GPR157	22	59	9	40	32	29
	76	48	38	16	46	8
GPR158	33	1	27	4	14	2
	1	1	22	0	17	0
GPR160	8	5	2	11	9	2
	11	11	7	4	6	1
GPR161	46	39	25	40	40	35
	27	65	54	20	27	10
GPR162	7	4	6	1	6	0
	1	3	6	3	3	1
GPR17	8	4	4	1	2	0
	2	2	2	2	1	0
GPR171	5	5	7	4	5	2
	0	5	6	0	3	0
GPR173	10	8	2	5	2	1
	2	0	5	1	6	2
GPR174	7	5	1	3	0	0
	1	4	1	1	1	0
GPR176	15	18	9	5	7	7
	9	14	8	3	8	0
GPR179	47	11	24	1	14	3
	10	14	19	7	12	1
GPR18	20	4	12	2	7	8
	4	4	10	0	2	0
GPR180	101	262	63	202	122	155
	103	316	133	40	151	27
GPR182	4	0	3	0	0	1
	0	0	1	0	3	0
GPR183	8	10	3	9	8	9
	8	14	13	4	5	2
GPR19	18	0	7	0	6	2
	0	0	4	0	2	0

GPR20	4	0	3	0	1	2
	1	0	2	0	0	0
GPR22	16	3	10	1	4	4
	0	4	3	0	5	0
GPR25	1	0	0	0	0	0
	0	0	0	0	0	0
GPR26	43	0	22	0	5	0
	0	1	13	0	11	0
GPR27	0	0	1	0	0	0
	1	0	1	0	0	0
GPR3	4	1	7	0	0	0
	0	1	2	0	0	0
GPR31	1	0	1	0	2	0
	0	0	1	0	0	0
GPR32	4	0	3	0	0	0
	0	0	0	0	0	0
GPR34	22	18	10	18	19	26
	25	52	19	11	17	10
GPR35	7	0	5	1	4	6
	2	7	4	0	1	2
GPR37	12	4	15	6	7	12
	9	13	14	0	6	2
GPR37L1	9	1	3	1	0	0
	2	0	3	0	0	0
GPR39	8	0	5	0	4	0
	0	0	6	0	5	0
GPR4	15	18	4	5	5	6
	6	12	5	5	11	4
GPR45	3	0	1	0	0	0
	0	0	0	0	0	0
GPR50	11	0	3	0	2	0
	0	0	0	0	3	0
GPR52	0	0	0	0	0	0
	0	0	0	0	0	1
GPR55	25	1	10	0	2	1
	0	0	8	0	6	0
GPR56	29	58	13	22	21	31
	57	78	34	25	47	11
GPR6	9	0	3	0	0	0
	0	0	1	0	1	0
GPR61	16	5	11	1	8	6
	13	9	9	5	13	0
GPR62	1	2	1	0	3	2
	1	0	3	0	2	1
GPR63	34	15	15	12	16	10
	10	15	14	1	19	2
GPR64	43	7	15	8	11	10
	6	18	17	4	6	1
GPR65	24	14	12	11	12	8
	11	6	22	6	14	1
GPR68	10	0	1	4	3	6
	3	0	4	1	1	1
GPR75	0	1	0	0	0	0
	0	1	0	0	0	0
GPR75-ASB3	44	112	51	93	94	94
	110	174	93	39	99	31
GPR77	2	0	0	0	0	1
	0	0	2	0	0	0

GPR78	5	1	6	1	2	1
	0	0	4	0	3	0
GPR82	18	1	6	1	6	3
	2	2	3	3	4	1
GPR83	18	1	6	1	4	6
	3	2	10	3	11	0
GPR84	7	0	0	0	2	0
	0	0	1	0	3	0
GPR85	33	2	16	1	8	0
	0	1	13	0	6	1
GPR87	12	0	8	1	1	2
	0	0	3	0	2	0
GPR88	7	3	4	0	1	0
	2	1	7	0	7	0
GPR89A	65	181	67	162	77	134
	187	256	145	50	165	37
GPR89B	32	93	34	51	31	70
	59	118	67	15	57	12
GPR89C	1	8	3	3	3	9
	1	7	3	4	1	5
GPR97	20	1	8	0	1	0
	0	3	6	0	4	0
GPR98	189	20	197	24	64	10
	8	1	132	5	78	1
GPRASP1	3	7	1	2	7	5
	8	5	2	3	7	0
GPRASP2	0	3	1	1	3	2
	0	2	1	1	1	0
GPRC5A	12	1	9	2	5	0
	1	5	7	1	3	1
GPRC5B	80	389	83	268	149	240
	383	283	137	112	214	66
GPRC5C	46	212	41	187	100	189
	104	150	103	32	52	21
GPRC5D	9	0	2	0	4	0
	0	0	5	0	0	0
GPRC6A	16	0	10	0	3	0
	0	0	12	0	6	0
GPRIN1	11	1	8	1	3	0
	0	0	4	0	0	0
GPRIN2	6	0	8	0	3	0
	0	0	11	0	0	0
GPRIN3	37	101	32	64	59	51
	119	111	59	42	67	22
GPS1	46	316	54	183	122	239
	244	345	182	107	185	75
GPS2	50	209	33	120	82	208
	163	242	90	97	164	43
GPSM1	11	15	9	5	12	7
	1	10	9	5	8	2
GPSM2	58	165	58	156	95	133
	105	228	131	34	85	47
GPSM3	9	18	8	4	10	7
	11	7	4	2	8	2
GPT	20	155	19	37	39	18
	79	172	61	27	70	12
GPT2	259	1006	145	483	560	465
	813	1579	351	279	601	280

GPX1	24	79	13	78	30	67
	137	152	97	32	45	14
GPX2	5	0	2	1	1	0
	0	0	0	0	1	0
GPX3	57	223	50	142	92	130
	198	284	86	64	116	44
GPX4	195	855	164	833	342	725
	1015	975	559	252	563	116
GPX5	11	0	5	0	4	0
	0	0	6	0	7	0
GPX6	9	0	7	0	1	0
	0	0	8	0	3	0
GPX7	10	34	12	55	25	29
	47	31	27	16	23	10
GPX8	38	44	22	51	26	52
	36	45	36	17	27	15
GRAMD1A	53	111	34	70	52	110
	118	128	62	38	86	22
GRAMD1B	46	27	21	25	28	11
	16	31	25	15	33	9
GRAMD1C	28	11	26	12	19	7
	31	58	43	12	38	14
GRAMD2	22	0	7	1	2	0
	0	0	8	1	3	0
GRAMD3	32	79	17	52	21	25
	45	65	41	12	36	14
GRAMD4	30	125	46	92	55	100
	69	98	54	27	37	14
GRAP	6	10	3	6	2	1
	2	5	6	0	5	2
GRAP2	7	4	7	1	3	2
	2	2	6	0	5	1
GRAPL	5	1	3	3	3	1
	3	2	3	3	3	0
GRASP	9	35	9	23	9	15
	17	9	12	4	10	4
GRB10	285	527	103	611	505	375
	674	678	362	302	421	125
GRB14	33	144	50	126	135	62
	55	121	29	29	38	11
GRB2	55	256	62	196	137	176
	211	285	164	87	149	47
GRB7	10	0	5	1	4	0
	0	0	3	0	3	0
GREB1	86	92	55	33	29	148
	81	129	83	60	50	20
GREB1L	148	370	159	270	176	330
	233	519	251	99	330	56
GREM1	21	32	13	24	39	63
	54	76	25	38	26	9
GREM2	32	120	26	130	13	73
	62	22	43	9	7	2
GRHL1	30	7	25	4	12	14
	7	10	24	6	11	2
GRHL2	35	0	26	1	8	1
	1	0	17	0	4	0
GRHL3	16	3	13	2	0	0
	2	3	18	0	6	0

GRHPR	84	361	52	265	140	255
	377	407	214	95	194	60
GRIA1	54	9	38	8	13	11
	0	6	42	3	17	4
GRIA2	39	6	33	6	10	39
	30	38	40	9	24	4
GRIA3	55	33	29	37	28	20
	13	12	28	4	18	3
GRIA4	47	4	33	2	17	8
	5	9	39	1	23	2
GRID1	35	6	32	4	10	3
	5	11	23	1	17	0
GRID2	32	6	20	11	9	0
	4	0	20	3	17	0
GRID2IP	14	1	13	0	2	0
	0	0	6	0	1	0
GRIK1	30	0	26	6	8	3
	3	4	23	0	12	1
GRIK2	36	46	58	85	41	72
	160	46	50	7	65	3
GRIK3	45	5	27	7	11	6
	1	5	21	4	18	4
GRIK4	33	2	11	3	5	3
	2	1	24	1	10	0
GRIK5	23	3	16	0	2	0
	2	1	10	1	6	0
GRIN1	21	0	6	0	1	0
	0	0	4	0	2	0
GRIN2A	76	7	50	19	20	7
	8	3	53	3	28	0
GRIN2B	37	0	23	0	12	0
	0	1	22	0	6	0
GRIN2C	13	2	12	4	1	0
	0	0	4	0	5	1
GRIN2D	15	0	7	2	0	0
	0	0	8	0	4	0
GRIN3A	49	4	36	6	9	4
	3	5	25	0	19	0
GRIN3B	2	0	2	0	0	0
	0	0	1	0	0	0
GRINA	19	96	24	65	34	59
	86	103	45	37	47	26
GRIP1	42	9	13	19	27	21
	12	12	21	6	16	4
GRIP2	155	644	124	324	308	368
	414	687	242	142	302	73
GRIPAP1	53	159	41	100	82	143
	108	123	75	57	91	26
GRK1 (NC_000013 114321593..114325974)				2	0	0
	0	0	0	0	0	2
	0	0	0			
GRK1 (NC_000013 114426045..114438637)				0	0	2
	0	0	0	0	0	0
	0	0	0			
GRK4	37	18	12	12	9	6
	10	13	16	6	15	1
GRK5	31	96	19	73	55	68
	69	138	49	30	43	22

GRK6	21	67	9	36	27	44
	37	60	24	23	37	17
GRK7	4	1	6	0	2	3
	0	2	8	0	1	0
GRM1	48	0	28	4	12	2
	1	1	26	0	6	0
GRM2	8	0	9	4	3	1
	0	1	2	1	7	2
GRM3	29	0	22	0	6	1
	0	2	8	0	8	0
GRM4	19	1	12	0	3	1
	0	0	12	0	6	0
GRM5	37	0	24	0	11	1
	1	0	16	0	12	1
GRM6	27	1	9	0	3	1
	2	1	14	0	5	0
GRM7	31	4	19	2	9	0
	0	0	16	0	5	0
GRM8	43	0	20	0	14	2
	0	0	17	0	16	0
GRN	35	73	14	83	42	60
	77	97	59	30	57	25
GRP	2	0	3	0	2	0
	0	0	2	0	2	0
GRPEL1	65	249	41	164	102	193
	274	325	164	68	176	32
GRPEL2	81	339	52	266	147	248
	222	293	146	81	183	49
GRPR	29	2	5	1	5	6
	3	2	4	2	8	2
GRSF1	539	3811	490	3000	1283	2215
	2976	2419	1506	857	1784	457
GRTP1	15	41	14	6	14	18
	36	44	14	12	25	4
GRWD1	22	65	14	34	31	53
	62	93	43	17	50	8
GRXCR1	7	0	1	0	1	0
	0	0	4	0	2	0
GRXCR2	5	0	6	0	2	0
	0	0	1	0	2	0
GSC	1	0	1	9	6	3
	1	4	2	2	7	0
GSDMA	16	2	9	0	0	1
	2	4	7	0	2	0
GSDMB	31	52	43	31	22	37
	22	63	31	23	30	13
GSDMC	45	30	14	44	36	38
	32	119	18	69	20	9
GSDMD	15	41	7	12	15	16
	25	58	25	15	19	4
GSE1	129	661	147	437	313	385
	456	492	195	144	176	81
GSG1	8	1	20	0	7	1
	0	0	15	0	5	0
GSG1L	30	0	10	0	3	1
	0	1	15	0	4	0
GSG2	11	3	3	2	1	6
	5	10	5	1	3	0

GSK3A	20	81	24	48	43	39
	62	79	24	21	36	13
GSK3B	179	1055	180	853	477	765
	681	750	414	241	500	167
GSKIP	13	54	14	44	25	33
	70	80	33	16	45	9
GSN	485	2138	601	2842	1119	1914
	1428	3393	1321	528	1151	651
GSPT1	455	2428	377	1777	1106	1830
	2045	2282	892	747	1279	381
GSPT2	26	95	20	63	48	76
	58	101	43	17	55	16
GSR	52	263	56	224	99	173
	199	211	111	55	124	32
GSS	55	138	34	67	69	99
	108	202	74	34	75	31
GSTA1	6	0	1	0	0	0
	0	0	2	0	0	0
GSTA2	13	0	13	0	3	0
	0	0	7	0	2	0
GSTA3	9	0	6	0	1	0
	0	0	5	0	1	0
GSTA4	41	153	30	183	77	150
	161	223	99	59	119	29
GSTA5	10	0	6	0	1	0
	0	0	8	0	0	0
GSTCD	46	76	34	57	33	61
	63	114	65	30	59	17
GSTK1	124	539	97	436	211	319
	760	769	366	159	382	97
GSTM1	2	2	0	2	66	114
	3	1	1	0	1	26
GSTM2	95	358	76	317	274	288
	532	439	215	120	241	103
GSTM3	103	197	110	385	70	252
	419	724	163	57	323	54
GSTM4	62	231	46	153	145	220
	245	278	132	83	122	41
GSTM5	23	22	10	35	16	11
	11	34	8	4	9	6
GSTO1	43	269	38	151	108	191
	261	419	167	55	177	42
GSTO2	7	4	4	2	2	1
	0	9	4	1	6	1
GSTP1	72	231	51	230	143	242
	315	657	235	95	189	55
GSTT1	19	117	17	89	51	110
	0	63	99	20	45	23
GSTT2	7	20	5	7	3	2
	33	13	2	3	7	2
GSTT2B	1	7	1	3	12	6
	10	7	0	1	0	0
GSTZ1	22	86	26	94	38	70
	95	89	60	24	52	18
GSX2	0	0	2	0	1	0
	0	0	1	0	0	0
GTDC1	49	245	74	216	87	211
	161	214	132	50	130	34

GTDC2	27	45	22	79	30	49
	51	91	30	19	31	13
GTF2A1	145	804	151	664	404	577
	554	767	335	208	398	141
GTF2A2	47	158	34	132	78	130
	168	268	98	53	99	24
GTF2B	53	164	26	123	81	87
	192	307	127	70	143	47
GTF2E1	41	153	33	91	99	83
	104	98	49	33	54	18
GTF2E2	21	57	22	51	40	59
	39	70	34	17	43	13
GTF2F1	20	103	18	57	49	56
	65	89	31	39	53	21
GTF2F2	102	354	95	296	140	205
	382	428	190	112	244	79
GTF2H1	67	290	92	210	187	231
	263	388	187	122	220	61
GTF2H2	57	181	59	109	83	86
	164	136	98	76	87	37
GTF2H2C	39	191	20	84	68	53
	92	223	92	60	123	35
GTF2H3	27	84	26	72	39	51
	75	116	50	28	59	12
GTF2H4	26	65	28	54	30	44
	54	102	36	15	38	14
GTF2H5	153	452	121	347	253	345
	261	592	216	125	311	77
GTF2I	684	3454	680	2460	1869	2584
	2367	3240	1668	1068	1689	566
GTF2IRD1	52	208	43	120	114	112
	151	142	97	64	87	53
GTF2IRD2	107	296	67	220	147	318
	283	324	158	91	197	75
GTF2IRD2B	95	419	108	370	215	360
	337	557	315	149	253	85
GTF3A	184	1131	170	895	367	1004
	1619	1519	703	352	657	199
GTF3C1	248	1070	229	782	531	849
	802	890	458	325	505	198
GTF3C2	77	188	56	139	102	155
	135	236	115	56	119	50
GTF3C3	103	352	96	278	207	279
	366	550	231	141	287	76
GTF3C4	51	161	42	93	79	114
	155	134	70	45	97	35
GTF3C5	42	130	28	120	75	109
	150	195	100	60	107	34
GTF3C6	131	936	103	583	330	685
	1187	861	358	223	451	132
GTPBP1	65	144	42	102	73	109
	126	184	78	61	90	33
GTPBP10	100	386	76	285	217	254
	277	479	205	89	246	78
GTPBP2	46	109	25	60	48	92
	76	151	61	35	76	21
GTPBP3	9	26	4	13	12	23
	15	21	14	8	21	2

GTPBP4	100	463	111	297	287	380
	346	522	182	142	258	85
GTPBP5	40	126	26	117	45	73
	113	106	45	19	73	20
GTPBP6 (NC_000023 221425..230887)				5	32	6
	20	8	13	20	40	11
	8	10	8			
GTPBP6 (NC_000024 171425..180887)				12	16	0
	14	6	21	13	31	16
	1	17	7			
GTPBP8	37	184	38	143	78	118
	182	244	95	32	116	27
GTSE1	13	1	5	0	2	1
	0	3	5	0	6	1
GTSF1	3	1	5	0	1	0
	0	0	5	0	3	0
GTSF1L	8	1	0	0	1	1
	0	0	0	0	1	0
GUCA1A	12	0	4	0	4	0
	0	0	4	0	0	0
GUCA1B	15	24	8	13	6	6
	6	23	9	2	19	4
GUCA1C	9	0	16	0	2	0
	0	0	6	0	8	0
GUCA2A	6	0	3	0	1	0
	0	0	2	0	3	0
GUCA2B	1	0	1	0	0	0
	0	0	0	0	0	0
GUCY1A2	204	820	112	622	377	445
	851	974	406	200	425	128
GUCY1A3	137	529	113	509	217	361
	341	527	217	154	249	106
GUCY1B3	40	126	35	98	53	77
	121	160	67	45	71	23
GUCY2C	36	6	27	5	14	5
	2	7	28	0	22	0
GUCY2D	12	0	7	0	1	0
	0	0	3	1	4	0
GUCY2F	34	0	18	1	6	0
	0	0	12	0	6	0
GUF1	170	845	174	733	368	642
	675	807	350	235	542	150
GUK1	50	266	38	215	103	200
	235	260	131	74	130	41
GULP1	56	144	41	112	86	118
	174	180	79	70	103	37
GUSB	31	76	24	47	35	55
	56	80	37	21	57	14
GXYLT1	112	205	109	151	130	163
	172	204	124	62	150	49
GXYLT2	11	18	10	19	23	23
	13	23	19	3	16	4
GYG1	208	1243	126	920	425	983
	1326	1433	465	251	624	164
GYG2	12	6	8	4	9	1
	1	4	4	0	1	4
GYLTL1B	11	0	5	0	1	0
	0	0	3	0	2	0

GYPA	19	1	17	2	5	1
	1	0	8	0	5	0
GYPB	3	0	1	0	0	1
	0	0	0	0	1	0
GYPC	93	465	88	413	173	415
	408	646	207	117	323	71
GYPE	32	3	29	1	6	3
	5	2	27	1	8	1
GYS1	263	2484	281	1065	713	1448
	1887	2203	921	625	1169	331
GYS2	27	3	20	1	13	3
	0	6	21	1	13	0
GZF1	52	200	51	122	113	141
	168	152	86	59	120	22
GZMA	12	2	3	5	5	0
	5	4	8	2	9	1
GZMB	5	1	5	1	0	0
	1	6	1	1	1	0
GZMH	9	6	4	0	3	3
	0	3	5	0	0	2
GZMK	8	2	3	0	2	0
	2	1	8	0	5	0
GZMM	2	0	2	0	0	0
	0	0	2	0	0	0
H1F0	86	630	72	381	242	350
	552	431	232	107	322	63
H1FNT	5	0	0	0	1	0
	0	0	0	1	0	0
H1FOO	1	0	1	0	0	0
	0	0	2	0	0	0
H1FX	17	103	9	70	47	70
	78	124	59	33	79	27
H2AFB2	1	0	0	0	0	0
	0	0	0	0	0	0
H2AFB3	1	0	0	0	0	0
	0	2	0	0	0	1
H2AFJ	39	138	40	195	69	117
	144	175	83	34	93	16
H2AFV	291	1658	394	1691	639	1143
	1406	1835	726	435	1089	343
H2AFX	2	8	2	6	3	10
	10	7	6	3	6	2
H2AFY	76	439	85	297	192	252
	338	442	203	122	217	91
H2AFY2	13	47	15	24	23	29
	39	47	21	13	26	6
H2AFZ	58	245	33	207	157	173
	292	424	156	81	121	38
H2BFM	8	1	2	0	0	0
	0	0	1	0	0	0
H2BFWT	2	0	1	0	0	0
	0	0	1	0	1	0
H3F3A	203	1113	232	887	529	787
	1222	1285	638	292	610	188
H3F3B	313	2158	431	1500	940	1293
	1620	3996	1133	513	1468	306
H3F3C	3	1	0	0	2	0
	0	0	5	0	0	0

H6PD	284	918	195	680	597	607
	1034	1373	700	410	773	372
HAAO	13	6	4	3	2	4
	3	7	7	2	4	2
HABP2	43	42	17	57	23	39
	27	43	35	12	28	6
HABP4	22	94	24	48	34	69
	62	105	52	21	33	13
HACE1	70	148	76	116	88	148
	129	167	113	63	117	48
HACL1	53	179	36	104	68	120
	132	266	79	62	101	27
HADH	135	977	115	605	490	534
	1046	1102	300	203	435	112
HADHA	790	4267	747	2468	2018	2129
	2710	5933	1366	1024	2205	601
HADHB	1037	4661	644	2277	2095	2046
	3859	7682	1537	966	2662	650
HAGH	125	668	81	488	262	335
	669	793	302	140	290	84
HAGHL	0	1	3	0	0	1
	1	0	1	0	0	0
HAL	31	3	24	0	7	3
	0	2	13	0	5	0
HAMP	2	0	1	0	0	0
	0	0	1	0	0	0
HAND1	11	0	7	0	1	0
	0	0	3	0	2	0
HAND2	12	0	3	3	1	1
	0	0	4	0	3	0
HAO1	8	0	9	0	3	0
	0	0	17	0	3	0
HAO2	25	0	18	0	3	0
	0	0	15	1	4	0
HAP1	20	5	4	1	1	1
	0	0	6	0	6	0
HAPLN1	26	0	11	0	6	0
	0	0	9	0	6	0
HAPLN2	2	1	3	0	2	1
	0	4	1	0	2	1
HAPLN3	3	3	4	0	2	3
	2	6	6	1	2	0
HAPLN4	9	0	8	0	0	0
	0	0	4	0	4	1
HARBI1	0	11	9	2	5	8
	17	11	9	5	5	4
HARS	66	237	67	161	81	209
	173	225	126	79	140	35
HARS2	87	315	56	236	148	251
	254	416	168	99	199	55
HAS1	4	0	1	0	2	0
	0	0	3	0	1	0
HAS2	14	25	28	63	27	37
	19	55	27	4	14	7
HAS3	23	15	17	7	6	15
	14	7	10	2	11	1
HAT1	119	664	103	363	297	441
	495	804	259	274	363	106

HAUS1	5	42	17	15	9	27
	33	55	13	14	22	12
HAUS2	50	103	20	76	47	52
	59	139	52	48	78	21
HAUS3	61	206	58	147	91	164
	163	188	105	63	121	36
HAUS4	30	74	16	52	33	62
	64	108	67	25	42	14
HAUS5	26	34	13	7	19	19
	16	38	19	5	30	3
HAUS6	97	243	90	183	138	177
	181	251	138	52	141	41
HAUS7	5	37	13	20	10	34
	32	72	26	13	21	10
HAUS8	10	12	10	5	10	6
	17	23	13	2	7	1
HAVCR1	16	4	5	0	1	3
	1	2	6	0	4	1
HAVCR2	24	14	11	14	10	14
	9	17	10	5	14	2
HAX1	180	807	135	571	311	536
	955	1192	463	248	557	136
HBA1	28	349	10	38	8	131
	95	49	7	10	69	44
HBA2	9	821	7	154	36	419
	309	93	41	70	133	28
HBB	154	2272	40	541	96	804
	513	642	122	122	398	129
HBD	2	3	2	1	2	0
	0	0	0	0	0	0
HBE1	5	0	1	0	0	0
	0	0	1	1	1	0
HBEGF	21	75	19	49	47	82
	169	202	130	30	164	20
HBG1	4	30	2	3	1	0
	16	11	2	5	1	2
HBG2	7	35	1	8	3	0
	8	29	6	2	0	2
HBM	0	1	0	0	0	0
	0	0	1	0	0	0
HBP1	228	716	168	531	368	518
	739	1372	525	313	749	184
HBS1L	304	1602	357	1196	632	1140
	1164	1509	761	478	1023	274
HCAR1	11	0	2	1	5	1
	0	1	3	0	2	2
HCAR2	10	6	3	1	3	0
	4	6	4	0	1	3
HCAR3	9	1	6	4	1	1
	0	0	4	0	3	0
HCCS	56	333	40	210	102	200
	302	221	118	57	145	49
HCFC1	91	391	53	183	136	209
	284	290	113	111	141	66
HCFC1R1	73	445	47	338	103	363
	723	385	246	153	340	55
HCFC2	164	623	167	480	313	460
	545	773	344	197	481	144

HCK	21	16	12	17	10	14
	9	27	16	0	15	4
HCLS1	31	55	25	30	41	25
	45	59	41	19	36	7
HCN1	85	302	135	254	125	70
	143	137	56	41	85	10
HCN2	5	1	3	0	1	0
	0	0	2	0	2	0
HCN3	18	5	13	4	7	5
	0	4	9	2	12	2
HCN4	39	0	20	0	4	0
	0	0	13	0	5	0
HCRT	1	0	0	0	0	0
	0	2	0	0	0	0
HCRTR1	6	1	2	1	1	1
	0	2	5	1	3	0
HCRTR2	13	0	6	2	3	0
	0	0	5	0	3	0
HCST	2	2	0	1	0	2
	0	0	1	0	2	0
HDAC1	41	138	35	86	73	97
	92	176	65	41	87	22
HDAC10	14	24	9	23	17	32
	17	27	11	10	11	3
HDAC11	34	100	22	74	51	68
	95	113	49	43	89	27
HDAC2	103	453	72	287	197	277
	305	360	154	108	177	64
HDAC3	65	199	63	199	96	206
	185	225	132	70	142	45
HDAC4	231	1069	264	527	486	673
	844	769	493	475	493	220
HDAC5	138	424	93	330	259	395
	255	551	212	173	330	101
HDAC6	90	348	66	174	157	214
	257	425	155	109	231	69
HDAC7	38	148	38	94	55	81
	135	150	75	38	56	23
HDAC8	53	159	42	97	97	112
	124	192	93	47	73	42
HDAC9	185	910	208	595	348	618
	834	896	442	146	592	122
HDC	17	3	17	20	2	11
	1	8	8	2	5	5
HDDC2	126	560	89	406	243	406
	652	606	313	158	359	85
HDDC3	4	30	7	8	10	8
	33	35	25	11	18	4
HDGF	240	1256	224	981	598	952
	1092	1246	457	400	639	183
HDGFL1	2	0	0	0	1	0
	0	0	0	0	0	0
HDGFRP2	54	240	59	120	103	199
	129	247	88	122	160	56
HDGFRP3	23	88	19	42	38	59
	46	86	49	30	35	21
HDHD1	50	231	32	118	117	146
	166	177	70	65	76	21

HDHD2	55	245	48	216	110	188
	233	302	126	93	128	39
HDHD3	9	22	7	21	8	16
	12	13	15	4	7	2
HDLBP	957	5788	1137	3498	2656	3686
	3826	4802	2043	1941	3038	1158
HDX	45	28	17	27	28	15
	27	20	24	14	18	10
HEATR1	104	404	123	275	176	314
	257	389	206	146	225	57
HEATR2	28	95	26	74	50	49
	58	98	43	23	90	21
HEATR3	54	210	65	166	103	220
	158	201	122	76	134	44
HEATR4	29	6	25	9	11	4
	8	13	18	4	13	3
HEATR5A	220	576	149	445	298	448
	566	850	402	213	417	121
HEATR5B	157	456	134	369	246	336
	395	575	265	152	337	81
HEATR6	86	209	72	142	97	189
	121	202	75	42	99	28
HEATR7A	32	51	25	35	18	35
	43	49	26	15	29	9
HEATR7B1	42	0	23	0	10	1
	0	0	17	0	10	0
HEATR7B2	73	0	38	0	21	0
	2	3	36	0	22	0
HEATR8	88	252	45	30	99	30
	77	235	117	20	185	48
HEBP1	16	61	5	55	22	25
	41	45	12	6	15	13
HEBP2	115	647	73	421	216	500
	807	706	357	181	337	102
HECA	117	536	82	410	395	465
	417	646	271	181	255	114
HECTD1	1585	7998	1791	7329	3947	5813
	5963	8525	3577	2953	4955	1567
HECTD2	77	187	111	192	101	232
	156	202	125	60	101	28
HECTD3	63	161	59	129	106	143
	169	161	84	46	108	32
HECTD4	298	1133	366	674	540	728
	847	1140	571	330	617	241
HECW1	47	24	29	63	22	23
	5	32	34	2	48	2
HECW2	68	175	46	92	101	65
	177	187	110	78	110	46
HEG1	151	655	130	542	302	421
	514	552	261	193	366	122
HELB	28	29	24	21	30	15
	25	54	44	6	33	6
HELLS	37	16	26	16	11	20
	6	22	20	6	21	3
HELQ	89	288	112	258	142	258
	265	327	141	88	207	55
HELT	3	0	4	0	1	0
	0	0	0	0	2	0

HELZ	444	2389	569	1696	1132	1500
	1527	2010	910	610	1144	394
HELZ2	19	26	18	22	10	7
	21	15	18	17	21	5
HEMGN	17	73	11	3	8	36
	18	9	8	3	12	0
HEMK1	32	94	40	50	50	56
	33	63	43	19	41	15
HENMT1	18	28	11	34	10	80
	15	22	32	11	20	6
HEPACAM	13	4	16	2	5	10
	1	0	11	0	8	9
HEPACAM2	17	0	8	0	9	0
	0	0	6	0	5	0
HEPH	44	14	18	15	20	7
	11	17	22	5	12	9
HEPHL1	57	1	35	0	20	1
	0	1	30	0	20	0
HEPN1	6	0	5	0	0	0
	0	2	2	0	3	1
HERC1	1476	7105	1476	4484	3713	4037
	3701	6646	2654	2324	3767	1260
HERC2	658	2698	589	2124	1151	2345
	1853	2445	1394	746	1531	471
HERC3	156	700	183	534	366	535
	533	774	410	213	416	109
HERC4	122	581	136	435	313	410
	386	582	275	150	277	99
HERC5	32	34	32	16	33	17
	16	37	45	14	29	6
HERC6	37	81	29	38	22	21
	42	75	56	70	91	12
HERPUD1	119	608	89	524	230	471
	669	805	317	165	409	123
HERPUD2	37	162	48	118	92	128
	165	228	120	60	118	41
HES1	48	161	24	140	83	180
	222	108	69	81	82	26
HES2	8	0	4	0	1	6
	0	0	3	0	1	0
HES3	6	0	4	0	0	1
	0	0	0	0	0	0
HES4	1	5	1	1	1	0
	11	17	6	7	8	2
HES5	2	1	1	1	0	0
	0	0	0	0	1	0
HES6	3	9	0	3	4	11
	3	9	3	6	2	1
HES7	7	1	9	0	0	0
	0	0	0	0	1	0
HESX1	5	2	4	4	3	3
	4	2	5	0	2	1
HEXA	53	174	41	107	79	97
	120	175	91	51	145	41
HEXB	74	256	64	211	151	259
	221	401	163	83	154	51
HEXDC	14	68	16	41	32	48
	51	59	40	12	19	8

HEXIM1	46	251	72	267	134	167
	277	103	51	54	64	34
HEXIM2	10	40	8	34	8	51
	39	22	11	8	18	8
HEY1	23	38	12	24	17	33
	64	72	30	12	50	9
HEY2	40	115	19	94	43	62
	173	97	62	33	75	23
HEYL	16	37	10	32	17	18
	29	22	29	6	24	2
HFE	17	20	15	19	14	20
	21	18	22	7	20	7
HFE2	562	3645	792	3427	1611	3505
	3970	4822	2289	1153	2254	563
HFM1	71	130	76	189	79	112
	157	212	138	58	226	28
HGC6.3	1	0	0	1	2	0
	0	1	2	0	2	0
HGD	23	2	11	0	3	0
	3	2	14	0	4	1
HGF	36	20	24	19	15	17
	11	15	24	5	21	8
HGFAC	7	0	0	0	1	0
	0	0	2	0	0	0
HGS	46	139	47	131	65	145
	113	206	101	63	103	41
HGSNAT	82	274	74	181	111	179
	164	279	154	69	130	53
HHAT	36	14	19	8	10	13
	3	13	30	6	22	1
HHATL	101	441	80	308	128	359
	369	638	276	100	364	78
HHEX	8	15	3	6	7	14
	9	7	9	4	9	8
HHIP	20	8	15	9	4	5
	11	15	12	6	13	3
HHIPL1	11	1	7	1	2	3
	1	9	6	0	9	1
HHIPL2	26	5	13	1	8	1
	0	0	9	0	3	0
HHLA1	33	0	18	0	7	0
	0	0	21	0	7	0
HHLA2	49	0	31	2	7	2
	2	0	19	0	5	0
HHLA3	24	7	11	4	8	5
	7	11	9	2	11	2
HIAT1	55	150	41	159	109	152
	153	276	105	76	120	30
HIATL1	46	211	51	181	76	178
	219	318	96	64	147	47
HIBADH	133	764	127	501	435	546
	840	999	354	235	616	130
HIBCH	116	442	122	437	239	413
	439	395	255	113	230	57
HIC1	11	36	5	45	23	42
	21	46	37	11	17	8
HIC2	36	17	15	10	13	20
	26	44	20	9	32	11

HID1	11	26	12	6	12	11
	17	13	12	4	10	6
HIF1A	92	432	87	277	196	225
	346	346	171	124	228	98
HIF1AN	139	831	130	574	309	389
	590	571	271	154	308	116
HIF3A	40	45	25	31	22	12
	62	53	36	30	47	26
HIGD1A	137	839	136	646	288	512
	934	1339	547	202	594	130
HIGD1B	6	27	5	9	13	7
	42	43	18	8	11	2
HIGD1C	0	0	0	0	1	0
	0	0	2	0	0	0
HIGD2A	50	178	24	110	79	132
	209	285	104	51	116	28
HILPDA	11	13	5	12	5	10
	5	15	11	1	7	1
HINFP	34	52	13	32	35	63
	67	64	38	21	47	9
HINT1	130	648	124	713	314	774
	975	1559	475	229	587	128
HINT2	16	63	7	48	16	37
	81	80	37	14	59	10
HINT3	308	1463	225	1237	527	1079
	1365	1427	727	402	879	234
HIP1	60	198	48	87	83	69
	100	114	78	28	75	53
HIP1R	24	45	22	18	16	16
	25	27	39	7	22	8
HIPK1	222	1110	260	1101	581	854
	897	1005	506	328	558	173
HIPK2 (NC_000007 139246315..139342363)				295	1272	226
	807	609	767	878	924	402
	368	552	252			
HIPK2 (NC_000007 139415729..139477693)				17	98	16
	58	44	57	82	94	31
	32	46	23			
HIPK3	683	3421	834	2924	1997	2579
	2691	3820	1548	1121	2473	654
HIPK4	7	1	6	3	1	0
	0	0	4	0	4	1
HIRA	52	121	45	87	69	101
	116	116	56	52	94	30
HIRIP3	32	89	30	46	48	63
	55	98	29	30	32	14
HIST1H1A	3	0	0	1	0	0
	1	1	3	0	0	0
HIST1H1B	1	2	1	6	0	1
	1	4	1	0	3	0
HIST1H1C	131	839	118	769	567	477
	900	1373	513	187	550	189
HIST1H1D	9	21	7	11	13	12
	14	40	5	6	6	1
HIST1H1E	35	372	63	267	160	168
	289	310	159	82	161	60
HIST1H1T	6	5	0	1	2	7
	2	2	0	3	2	2

HIST1H2AA	0	0	0	0	0	0
	0	0	0	0	1	0
HIST1H2AB	1	1	0	2	0	0
	0	0	1	0	1	0
HIST1H2AC	29	150	34	191	77	129
	196	202	130	54	105	38
HIST1H2AD	0	1	1	3	2	1
	3	4	0	1	0	0
HIST1H2AE	0	7	3	12	4	2
	6	10	6	1	4	4
HIST1H2AG	1	4	0	2	1	0
	7	2	2	0	1	0
HIST1H2AH	1	9	2	1	1	1
	4	4	0	1	1	1
HIST1H2AI	1	0	1	1	0	0
	0	1	1	2	1	0
HIST1H2AJ	0	0	1	2	0	0
	1	0	0	1	0	0
HIST1H2AK	1	2	0	0	1	0
	0	1	1	0	0	0
HIST1H2AL	3	0	0	1	1	0
	2	0	2	1	1	0
HIST1H2AM	2	2	1	3	0	0
	5	3	2	0	3	0
HIST1H2BA	1	0	0	0	0	0
	0	0	0	0	0	0
HIST1H2BB	0	4	1	5	1	1
	2	3	1	0	1	0
HIST1H2BC	11	96	34	75	45	47
	83	81	50	19	46	8
HIST1H2BD	31	124	29	83	63	61
	90	121	28	40	38	19
HIST1H2BE	1	4	0	2	1	1
	3	4	1	0	2	0
HIST1H2BF	0	1	0	0	2	1
	1	4	1	0	0	0
HIST1H2BG	4	1	2	3	2	1
	3	4	0	2	0	1
HIST1H2BH	1	2	0	0	0	0
	0	1	0	0	0	0
HIST1H2BI	2	1	0	2	1	0
	0	4	2	0	1	1
HIST1H2BJ	6	18	3	22	12	6
	24	14	8	7	17	1
HIST1H2BK	9	21	4	18	17	18
	27	16	22	4	12	2
HIST1H2BL	2	0	0	1	0	0
	1	1	0	0	0	0
HIST1H2BM	4	0	0	0	0	3
	0	1	0	0	0	0
HIST1H2BN	1	1	2	3	0	2
	5	1	1	0	1	0
HIST1H2BO	2	0	1	1	2	0
	1	0	2	0	0	0
HIST1H3A	0	4	1	1	2	1
	3	3	3	0	1	0
HIST1H3B	1	1	1	0	2	0
	2	0	0	1	0	0

HIST1H3C	2	0	0	2	3	0
	0	3	0	0	0	0
HIST1H3D	1	1	3	2	2	0
	9	4	1	1	0	0
HIST1H3E	0	2	1	1	0	0
	2	1	0	0	0	0
HIST1H3F	0	0	1	0	0	0
	0	1	0	0	0	0
HIST1H3G	0	1	0	0	0	0
	0	0	0	0	0	0
HIST1H3H	0	0	0	0	0	0
	3	3	1	0	0	1
HIST1H3I	1	3	0	0	1	0
	0	4	1	0	2	1
HIST1H3J	0	0	0	0	1	0
	0	1	0	0	0	0
HIST1H4A	0	0	0	2	2	0
	0	0	1	0	0	0
HIST1H4B	4	19	1	10	7	2
	12	21	10	3	8	1
HIST1H4C	15	54	23	47	49	8
	89	123	64	8	68	12
HIST1H4D	1	1	0	9	1	0
	0	7	1	0	0	2
HIST1H4E	8	16	2	31	8	11
	19	16	8	5	10	2
HIST1H4F	0	1	0	0	1	0
	1	0	0	0	0	2
HIST1H4H	18	49	18	57	70	29
	76	91	56	19	86	12
HIST1H4I	1	1	0	0	0	0
	1	2	2	0	1	0
HIST1H4J	2	0	1	1	1	0
	1	2	1	1	3	0
HIST1H4K	2	2	0	0	1	0
	0	4	3	0	0	1
HIST1H4L	0	3	2	0	1	2
	1	4	2	0	1	2
HIST2H2AA3	24	59	15	105	37	59
	68	68	28	13	36	6
HIST2H2AA4	16	57	17	114	37	57
	75	55	29	20	43	11
HIST2H2AB	2	22	0	9	5	8
	9	30	5	3	11	1
HIST2H2AC	62	376	60	217	175	111
	391	780	279	111	294	67
HIST2H2BE	73	387	78	358	172	259
	349	447	169	91	263	82
HIST2H2BF	35	29	15	22	19	17
	24	31	29	3	18	7
HIST2H3A	0	1	1	0	0	1
	1	1	1	0	1	0
HIST2H3C	0	1	0	0	0	1
	0	0	0	0	1	0
HIST2H3D	0	0	1	0	2	0
	1	1	1	0	2	0
HIST2H4A	4	7	1	5	2	5
	11	19	5	3	1	1

HIST2H4B	3	4	1	7	2	3
	6	17	6	2	6	0
HIST3H2A	1	2	0	4	2	0
	0	5	1	1	1	0
HIST3H2BB	0	0	0	0	0	0
	0	0	1	0	0	1
HIST4H4	8	12	1	11	5	10
	22	27	11	6	14	3
HIVEP1	85	257	80	186	144	175
	140	199	110	63	114	42
HIVEP2	314	1330	282	1115	814	866
	946	1198	542	638	701	352
HIVEP3	73	46	44	31	32	20
	37	39	36	16	28	6
HJURP	27	0	21	0	3	0
	0	0	12	0	7	0
HK1	250	1376	309	970	755	1084
	1394	672	506	384	574	163
HK2	61	124	21	21	73	35
	214	472	61	103	101	108
HK3	14	8	15	1	5	8
	7	18	11	0	6	0
HKDC1	20	0	15	2	9	0
	0	0	13	1	7	0
HKR1	60	197	63	102	67	88
	146	201	98	65	96	44
HLA-A	182	802	130	750	265	709
	714	1517	649	397	631	168
HLA-B	135	931	79	404	314	345
	572	1181	386	260	573	150
HLA-C	113	676	68	427	214	193
	437	345	313	147	232	98
HLA-DMA	18	60	14	29	24	38
	38	108	35	19	49	13
HLA-DMB	15	35	10	31	9	38
	38	59	14	27	45	12
HLA-DOA	20	18	11	5	10	9
	6	31	22	8	23	7
HLA-DOB	12	3	9	7	5	2
	1	6	9	0	9	1
HLA-DPA1	48	170	22	89	54	87
	120	264	113	52	135	24
HLA-DPB1	61	129	38	125	44	94
	188	207	144	98	136	19
HLA-DQA1	32	73	15	70	18	66
	75	87	68	25	98	13
HLA-DQA2	7	11	12	0	5	5
	0	6	2	8	5	1
HLA-DQB1	33	49	15	100	32	40
	99	124	41	9	74	15
HLA-DQB2	11	5	2	0	5	0
	1	3	2	2	8	0
HLA-DRA	68	395	40	218	106	227
	236	455	287	166	300	67
HLA-DRB1	71	284	50	263	105	198
	375	515	216	84	354	59
HLA-DRB5	32	144	9	106	76	64
	161	131	75	54	203	46

HLA-E	188	1343	180	855	493	668
	1140	1422	720	432	916	255
HLA-F	22	100	21	68	38	56
	78	128	71	36	77	9
HLA-G	4	0	3	0	2	0
	0	1	6	0	6	0
HLCS	112	365	98	300	230	346
	295	408	199	109	260	66
HLF	85	200	71	131	95	189
	202	220	139	63	158	71
HLTF	136	459	112	386	266	427
	381	649	260	200	393	95
HLX	8	58	14	25	19	35
	26	53	20	10	20	4
HM13	41	113	30	48	45	80
	67	69	33	22	38	20
HMBOX1	89	417	130	277	191	237
	228	272	163	87	144	60
HMBS	25	55	12	28	22	11
	43	21	24	8	21	6
HMCN1	308	771	217	540	418	286
	510	608	490	163	441	181
HMCN2	154	389	102	205	188	359
	252	415	173	112	231	77
HMG20A	121	412	119	349	231	325
	349	417	213	130	279	69
HMG20B	20	55	14	29	24	38
	84	73	44	20	27	22
HMGA1	45	153	37	81	73	117
	149	166	84	57	86	19
HMGA2	18	0	8	1	7	1
	1	0	18	1	9	0
HMGB1	353	1870	408	1638	870	1805
	1731	2376	985	596	1219	356
HMGB2	45	208	47	105	87	83
	157	334	93	66	150	111
HMGB3	45	53	19	15	24	39
	38	38	20	8	21	6
HMGB4	15	0	9	0	2	0
	0	0	4	0	2	0
HMGCL	55	212	29	176	91	155
	233	325	145	60	164	43
HMGCLL1	27	36	21	35	23	11
	25	52	35	8	23	9
HMGCR	62	121	41	93	55	93
	114	94	70	29	75	19
HMGCS1	98	441	123	392	178	303
	431	387	240	97	325	46
HMGCS2	39	197	32	9	34	52
	54	487	20	3	20	4
HMGN1	32	208	67	143	82	170
	215	211	102	31	63	32
HMGN2	149	867	184	512	340	427
	545	928	396	202	518	195
HMGN3	126	652	105	476	294	556
	644	925	395	221	436	101
HMGN4	52	224	33	145	79	142
	127	255	104	57	127	36

HMGN5	12	17	9	15	16	8
	16	9	9	1	12	3
HMGXB3	72	202	61	132	81	145
	167	303	124	74	160	60
HMGXB4	41	152	46	86	60	82
	145	176	69	47	108	23
HMHA1	19	22	11	20	9	12
	12	30	10	8	16	3
HMHB1	4	0	1	0	3	0
	0	0	0	0	0	0
HMMR	36	13	18	9	21	8
	12	18	20	6	23	3
HMOX1	12	16	5	18	6	6
	31	23	13	11	12	6
HMOX2	30	87	18	39	34	56
	102	109	51	28	58	15
HMP19	23	1	7	1	3	3
	4	2	10	2	2	0
HMSD	4	0	2	0	2	1
	0	0	5	0	1	0
HMX1	0	0	2	0	0	0
	0	1	1	0	0	0
HMX2	3	0	0	0	0	0
	0	0	0	0	1	0
HN1	37	303	31	128	81	162
	143	154	106	38	94	24
HN1L	46	197	31	130	95	152
	94	150	61	40	75	27
HNF1A	6	1	7	0	1	1
	0	0	9	0	1	0
HNF1B	21	0	11	0	1	0
	0	0	7	0	2	0
HNF4A	33	1	12	0	7	0
	0	0	9	1	5	1
HNF4G	30	1	10	1	15	0
	1	0	24	0	15	0
HNMT	43	74	39	70	51	73
	55	124	68	26	60	17
HNRNPA0	61	423	93	263	164	267
	396	434	133	118	224	64
HNRNPA1	187	1452	274	941	520	905
	1037	1301	629	377	701	253
HNRNPA1L2	43	103	30	74	47	62
	74	126	50	48	73	19
HNRNPA2B1	432	3529	525	2597	1557	1921
	2835	3056	1062	878	1836	518
HNRNPA3	276	1746	337	1231	757	1093
	1159	1976	648	497	904	325
HNRNPAB	46	279	60	157	104	216
	195	267	111	90	139	44
HNRNPC	332	1555	339	912	809	1137
	1288	2065	734	485	926	296
HNRNPCL1	11	1	3	0	1	0
	0	0	1	0	5	0
HNRNPD	117	872	156	582	347	442
	651	802	271	206	340	95
HNRNPF	97	505	103	339	273	371
	426	665	290	191	326	104

HNRNPH1	373	1963	431	1454	915	1366
	1846	2421	970	641	1114	405
HNRNPH2	24	706	65	466	319	593
	572	913	181	239	332	124
HNRNPH3	105	555	111	380	279	352
	511	534	209	135	259	91
HNRNPK	642	3181	812	2346	1625	2052
	3274	4141	1689	1137	2602	682
HNRNPL	134	615	146	407	273	466
	558	749	281	232	415	135
HNRNPM	151	1071	123	524	340	553
	641	821	290	227	426	127
HNRNPR	144	1047	238	587	373	617
	666	913	370	305	541	162
HNRNPU	739	5121	1177	3437	2230	3601
	2994	4580	1756	1600	2563	846
HNRNPUL1	109	589	106	382	238	327
	438	493	191	160	275	105
HNRNPUL2	83	534	86	354	213	201
	343	335	135	127	164	84
HNRPDL	605	3399	595	1814	1619	2607
	2434	4202	1354	1246	1734	586
HNRPLL	92	283	58	246	201	250
	258	408	253	117	253	72
HOGA1	22	16	4	5	6	5
	5	21	14	6	28	8
HOMER1	218	1532	317	1143	614	1155
	967	1078	477	255	676	183
HOMER2	139	690	122	420	345	778
	812	789	373	246	506	138
HOMER3	42	185	41	87	52	213
	56	74	27	25	62	25
HOMEZ	16	88	26	36	32	46
	45	77	37	22	32	8
HOOK1	36	13	24	9	28	21
	4	11	30	7	28	5
HOOK2	83	165	26	116	110	133
	140	183	124	58	88	18
HOOK3	401	1675	399	1600	1084	1258
	1190	1819	743	629	952	318
HOPX	28	46	11	41	14	27
	43	62	19	5	35	5
HORMAD1	9	5	6	0	9	1
	1	1	10	2	4	2
HORMAD2	14	0	4	0	5	0
	0	0	11	0	4	0
HOXA1	12	4	7	4	4	0
	0	0	3	0	5	0
HOXA10	68	383	80	354	168	453
	461	463	257	121	284	49
HOXA11	14	24	10	18	15	28
	18	29	20	4	6	7
HOXA13	27	50	8	23	29	26
	31	18	17	8	21	5
HOXA2	9	7	8	5	5	7
	7	5	5	1	3	0
HOXA3	15	61	12	32	26	31
	47	36	26	21	25	10

HOXA4	16	48	5	20	18	26
	36	27	13	13	14	4
HOXA5	7	55	17	32	39	45
	55	62	21	16	24	11
HOXA6	3	2	10	3	3	3
	7	9	2	3	2	1
HOXA7	19	68	19	45	15	48
	49	48	22	16	37	13
HOXA9	26	88	19	111	35	87
	91	123	37	30	27	23
HOXB1	1	0	1	0	0	0
	0	0	2	0	0	0
HOXB13	13	0	6	0	3	0
	0	0	4	0	3	0
HOXB2	9	20	1	17	16	39
	47	26	6	9	13	0
HOXB3	18	22	16	12	21	26
	29	29	18	11	31	2
HOXB4	6	21	6	5	10	13
	12	9	13	2	10	2
HOXB5	8	2	2	1	1	2
	4	0	1	0	0	0
HOXB6	14	5	7	8	5	2
	5	4	6	2	3	2
HOXB7	4	17	2	6	5	5
	11	11	5	5	4	3
HOXB8	5	0	2	0	2	0
	1	0	5	0	2	0
HOXB9	21	3	11	1	4	0
	0	0	5	0	3	0
HOXC10	38	167	16	99	54	92
	125	94	27	34	45	20
HOXC11	10	3	6	5	2	9
	3	1	5	0	2	0
HOXC12	3	0	2	0	0	0
	0	0	0	0	0	0
HOXC13	6	0	3	0	1	0
	0	0	0	0	0	0
HOXC4	13	63	15	48	27	36
	40	28	16	13	15	5
HOXC6	0	11	0	3	10	1
	7	4	0	0	3	0
HOXC8	18	84	14	41	32	59
	51	48	14	22	18	14
HOXC9	34	143	17	84	70	134
	204	152	44	54	66	18
HOXD1	6	1	4	0	0	0
	1	0	4	0	2	0
HOXD10	10	2	1	0	5	0
	0	0	6	0	4	1
HOXD11	5	1	0	0	0	0
	0	0	2	1	1	0
HOXD12	0	0	1	0	0	0
	0	0	0	0	0	0
HOXD13	12	0	7	0	5	0
	0	0	2	1	1	0
HOXD3	8	3	2	5	3	6
	9	2	2	1	4	2

HOXD4	8	6	3	3	6	5
	5	7	4	6	7	2
HOXD8	16	52	12	62	35	35
	42	51	26	12	27	7
HOXD9	5	5	4	2	2	1
	0	0	1	0	2	0
HP	16	0	8	4	1	2
	0	0	5	0	4	0
HP11026	4	1	0	0	1	0
	0	1	1	1	3	0
HP1BP3	281	1352	293	932	759	1081
	1092	1534	646	461	832	283
HPCA	2	0	1	0	1	0
	0	0	2	0	2	0
HPCAL1	22	42	8	11	11	19
	21	37	17	13	22	9
HPCAL4	20	6	10	3	16	16
	13	8	14	2	12	7
HPD	18	9	10	5	3	8
	3	1	4	2	6	2
HPDL	1	0	0	1	0	2
	6	6	6	0	4	1
HPGD	19	19	22	37	18	29
	7	49	18	3	17	6
HPGDS	17	10	7	19	25	31
	3	33	14	6	10	7
HPN	22	18	9	14	11	9
	40	80	2	3	31	22
HPR	8	0	7	1	3	0
	5	2	3	0	0	0
HPRT1	15	79	10	48	27	36
	51	72	18	14	22	7
HPS1	28	63	21	28	16	51
	37	54	33	10	37	6
HPS3	51	113	42	88	46	65
	72	108	87	34	68	20
HPS4	58	158	41	118	63	101
	96	162	95	39	92	21
HPS5	72	194	58	123	102	142
	128	255	114	53	141	44
HPS6	10	54	16	41	19	34
	54	64	18	15	29	11
HPSE	25	3	11	4	6	1
	5	6	11	3	8	0
HPSE2	35	1	31	3	7	6
	0	0	19	1	14	1
HPX	8	1	5	1	3	2
	1	3	3	0	7	0
HR	20	2	13	6	8	11
	3	8	16	3	3	3
HRAS	23	194	20	119	46	125
	157	143	86	48	67	24
HRASLS	91	324	46	272	119	237
	334	277	113	81	156	32
HRASLS2	10	0	2	0	0	1
	0	0	5	0	1	0
HRASLS5	20	1	14	2	14	5
	4	3	10	0	7	6

HRC	337	2404	300	1337	990	1467
	1264	1600	577	742	1065	338
HRCT1	1	6	1	3	3	1
	1	6	6	0	6	1
HRG	19	0	12	0	6	1
	0	0	3	0	3	0
HRH1	48	20	17	34	24	39
	11	31	26	8	22	7
HRH2	15	2	7	1	3	1
	1	1	18	0	4	0
HRH3	6	0	2	0	0	0
	0	0	0	0	0	0
HRH4	21	1	9	8	7	6
	0	5	9	1	4	1
HRK	2	0	0	0	0	0
	3	0	0	1	0	0
HRNR	66	1	34	0	15	0
	0	2	27	0	20	0
HRSP12	66	214	37	201	87	157
	247	344	139	60	138	40
HS1BP3	15	43	12	30	17	42
	19	56	29	10	19	6
HS2ST1	62	163	50	232	138	177
	151	204	138	38	135	40
HS3ST1	2	1	3	2	1	0
	0	3	3	2	3	0
HS3ST2	2	1	5	0	5	1
	0	2	1	0	2	0
HS3ST3A1	4	2	6	1	0	0
	1	0	2	0	2	0
HS3ST3B1	3	0	0	0	1	0
	1	1	1	1	1	1
HS3ST4	11	0	3	0	1	0
	0	0	6	0	1	0
HS3ST5	28	111	27	63	51	54
	83	38	38	28	44	7
HS3ST6	2	0	0	0	0	0
	0	0	0	0	0	0
HS6ST1	44	165	33	158	90	146
	110	153	66	55	81	22
HS6ST2	33	129	19	114	32	171
	108	48	68	9	73	1
HS6ST3	40	6	26	17	11	21
	17	20	32	0	6	3
HSBP1	83	602	96	333	242	364
	624	835	219	144	269	79
HSBP1L1	16	51	18	38	20	64
	48	60	37	20	36	8
HSCB	16	38	13	34	16	35
	43	61	29	9	23	5
HSD11B1	16	22	8	31	11	26
	17	59	42	11	27	11
HSD11B1L	6	15	3	6	10	10
	18	14	6	5	11	1
HSD11B2	8	1	4	2	2	1
	2	4	5	0	2	0
HSD17B1	12	7	8	6	7	13
	2	11	5	2	1	0

HSD17B10	31	111	20	87	48	84
	151	182	87	23	75	26
HSD17B11	21	84	19	67	45	68
	70	170	80	34	64	20
HSD17B12	63	364	74	270	165	220
	281	381	115	76	157	66
HSD17B13	12	1	12	12	4	2
	3	1	10	1	5	2
HSD17B14	4	1	5	1	0	4
	2	1	2	0	1	0
HSD17B2	11	0	9	0	2	1
	0	0	4	0	2	0
HSD17B3	13	3	14	0	7	0
	1	9	8	1	7	1
HSD17B4	231	893	203	576	435	618
	784	1085	482	266	587	136
HSD17B6	6	0	6	0	3	2
	0	2	6	0	2	0
HSD17B7	25	27	6	18	11	17
	17	28	27	6	14	3
HSD17B8	7	16	5	12	5	12
	14	34	11	3	17	5
HSD3B1	6	0	9	0	6	1
	0	0	2	0	0	0
HSD3B2	12	0	6	0	4	0
	1	0	7	0	3	0
HSD3B7	13	19	11	35	10	18
	19	33	9	2	9	4
HSDL1	71	290	71	261	151	224
	347	337	193	108	215	43
HSDL2	603	2876	556	1911	1400	2309
	2575	3895	1206	790	2135	493
HSF1	65	326	32	214	135	222
	335	388	153	117	180	60
HSF2	118	587	127	458	371	436
	624	809	385	189	547	119
HSF2BP	13	7	9	6	4	1
	10	4	9	2	11	4
HSF4	64	181	44	166	100	195
	179	281	110	69	96	33
HSF5	29	0	20	2	6	0
	2	8	8	1	10	0
HSFX1	4	3	4	7	3	9
	6	8	3	3	2	1
HSFX2	10	9	6	4	4	7
	12	5	2	2	8	3
HSFY1	0	2	8	0	0	2
	2	1	1	0	2	0
HSFY2	0	2	4	4	0	1
	0	1	7	0	2	0
HSH2D	8	4	3	1	3	4
	1	1	4	0	2	1
HSP90AA1	538	5461	873	3088	2990	3069
	3410	4016	1317	1236	2187	743
HSP90AB1	903	6345	960	4025	3075	4179
	4705	4984	2141	1773	2909	962
HSP90B1	213	1463	262	1254	638	1088
	1004	1278	565	361	671	269

HSPA12A	54	132	29	73	86	89
	66	65	38	23	56	24
HSPA12B	16	82	16	28	31	40
	73	64	46	32	45	14
HSPA13	42	175	29	107	72	107
	102	133	89	29	85	26
HSPA14	40	258	56	223	120	179
	199	259	106	55	165	36
HSPA1A	301	1546	321	823	872	1101
	961	2515	848	463	977	250
HSPA1B	171	710	155	410	388	528
	474	1114	319	287	438	199
HSPA1L	20	85	20	38	39	42
	83	36	23	12	37	6
HSPA2	393	1948	392	1837	873	1352
	1096	803	754	377	676	206
HSPA4	169	1167	191	598	500	660
	558	920	360	293	549	185
HSPA4L	60	92	38	63	55	53
	50	81	58	13	42	11
HSPA5	94	829	131	534	247	405
	644	475	265	145	358	115
HSPA6	10	6	3	7	1	4
	2	3	2	0	3	2
HSPA8	2165	15389	2265	9146	6986	11356
	11929	18752	6881	4026	8570	2147
HSPA9	713	4443	623	2535	1971	2882
	3436	5376	1616	1395	2636	643
HSPB1	168	757	103	351	497	522
	696	620	276	275	313	144
HSPB11	12	40	20	28	28	21
	38	46	22	16	19	9
HSPB2	20	123	30	112	68	91
	118	144	80	54	97	20
HSPB3	97	360	145	445	193	323
	495	718	288	115	260	79
HSPB6	62	478	83	166	205	109
	640	466	130	337	234	346
HSPB7	583	3040	302	2669	1315	1732
	2088	3586	891	603	1783	476
HSPB8	402	2287	434	2264	814	1514
	1764	2037	1094	525	1208	292
HSPB9	1	0	0	2	0	0
	0	1	0	0	0	0
HSPBAP1	48	181	34	88	82	131
	168	385	115	65	132	45
HSPBP1	17	58	5	23	30	24
	51	91	22	13	32	6
HSPD1	194	1130	198	695	647	712
	943	1434	375	379	625	206
HSPE1	48	362	44	166	153	195
	387	465	136	101	181	37
HSPE1-MOB4	116	309	81	247	156	261
	274	381	200	115	215	56
HSPG2	364	1726	269	1109	764	1037
	1215	1214	698	419	800	348
HSPH1	65	593	115	347	327	433
	243	417	125	82	230	70

HTATIP2	33	68	14	59	51	51
	90	126	60	25	68	10
HTATSF1	245	1676	364	1116	746	1274
	1144	1365	481	540	869	277
HTN1	0	0	1	0	0	0
	0	0	1	0	0	0
HTN3	4	0	1	1	1	0
	0	0	1	0	0	0
HTR1A	9	0	6	0	0	0
	0	0	0	0	0	0
HTR1B	2	0	3	0	1	2
	0	0	1	0	1	0
HTR1D	18	0	9	1	5	0
	0	0	12	0	7	0
HTR1E	15	0	1	1	1	0
	0	0	5	0	0	0
HTR1F	18	25	9	14	17	11
	6	25	23	9	16	6
HTR2A	22	5	15	5	3	6
	3	31	9	17	40	7
HTR2B	18	2	10	4	8	8
	4	6	9	0	6	0
HTR2C	30	0	7	0	3	0
	0	0	6	0	10	0
HTR3A	14	0	11	0	2	0
	0	0	7	0	2	0
HTR3B	16	0	11	0	3	0
	0	1	8	0	5	0
HTR3C	13	0	4	0	1	0
	0	0	9	0	3	0
HTR3D	11	0	4	0	1	0
	0	0	3	0	2	1
HTR3E	13	0	5	0	4	0
	0	0	4	0	3	0
HTR4	22	2	17	2	8	3
	4	3	12	0	19	0
HTR5A	12	0	16	0	2	0
	0	0	14	0	4	0
HTR6	2	0	1	0	0	0
	0	0	2	0	1	0
HTR7	31	62	33	65	14	38
	7	11	49	0	16	0
HTRA1	57	131	36	149	96	133
	81	185	84	37	63	35
HTRA2	36	63	15	43	30	48
	59	92	51	16	36	20
HTRA3	31	68	10	63	39	48
	56	174	87	40	57	18
HTRA4	28	6	17	2	7	3
	3	2	6	1	4	0
HTT	240	764	333	722	405	505
	951	1057	498	309	796	294
HUNK	56	4	22	13	15	5
	0	14	20	4	23	3
HUS1	42	228	52	129	93	136
	185	237	86	73	155	34
HUWE1	793	4419	879	2846	2026	2726
	2976	3273	1366	1238	2005	787

HVCN1	17	26	7	13	15	11
	35	32	13	11	15	3
HYAL1	27	100	16	61	32	51
	66	88	37	36	30	10
HYAL2	18	90	19	54	49	61
	80	119	36	16	41	19
HYAL3	6	4	4	0	0	1
	1	4	2	0	3	0
HYAL4	79	377	101	462	154	313
	97	258	72	57	240	30
HYDIN	280	19	161	22	67	15
	11	12	126	0	93	4
HYI	34	83	31	67	42	47
	59	88	36	23	42	12
HYLS1	25	39	17	26	29	39
	34	26	16	10	10	5
HYOU1	62	201	49	113	75	130
	75	130	54	33	75	32
HYPK	46	277	41	128	109	211
	379	221	109	90	160	48
IAH1	44	142	25	130	69	102
	162	255	93	41	92	29
IAPP	11	1	4	1	7	0
	1	0	10	1	2	0
IARS	283	1315	237	866	625	947
	929	1248	539	399	774	254
IARS2	260	1459	301	1030	736	979
	1245	1270	542	365	800	182
IBA57	60	141	29	77	62	82
	82	136	48	36	66	28
IBSP	10	0	2	0	3	2
	8	2	10	0	2	0
IBTK	293	1336	280	1174	554	891
	1314	1568	757	439	895	247
ICA1	33	55	26	30	22	23
	47	63	35	19	28	8
ICA1L	104	175	83	172	61	162
	135	218	107	47	96	43
ICAM1	24	27	15	24	19	20
	33	34	19	10	16	6
ICAM2	18	43	8	29	22	30
	49	71	30	8	36	3
ICAM3	13	40	5	33	20	11
	35	38	14	10	18	3
ICAM4	8	1	3	0	3	0
	0	0	1	0	2	1
ICAM5	12	2	7	0	3	0
	0	0	3	0	2	1
ICK	101	291	95	238	133	200
	210	370	177	90	175	63
ICMT	133	592	108	437	224	430
	444	639	401	130	285	130
ICOS	17	3	18	0	3	0
	0	1	9	0	5	0
ICOSLG	10	22	7	20	12	6
	1	17	13	4	12	4
ICT1	48	227	34	140	57	146
	204	327	104	60	112	39

ID1	6	78	12	64	47	34
	70	86	67	19	53	15
ID2	8	65	12	58	17	27
	79	45	16	11	22	10
ID3	2	24	5	14	14	15
	33	32	8	8	11	7
ID4	29	49	25	37	6	15
	27	32	25	6	15	17
IDE	296	1192	258	947	621	1038
	994	1614	630	490	743	242
IDH1	76	414	69	261	229	281
	288	320	189	68	180	55
IDH2	969	4523	660	4250	2329	3247
	5759	4948	2472	1560	3159	668
IDH3A	114	684	112	461	418	419
	621	635	264	183	309	72
IDH3B	115	620	104	580	178	506
	549	932	498	215	594	125
IDH3G	48	176	26	118	76	111
	162	149	88	46	94	31
IDI1	258	1416	154	711	635	1003
	1135	1700	491	339	617	170
IDI2	346	572	35	314	720	705
	884	1659	247	500	389	265
IDNK	14	18	5	13	5	13
	16	12	15	3	11	3
IDO1	24	56	9	34	24	59
	59	94	100	64	62	15
IDO2	28	1	7	1	4	0
	1	1	10	1	2	0
IDS	112	418	92	443	192	320
	402	473	200	122	223	58
IDUA	6	11	2	8	4	8
	1	8	11	1	6	0
IER2	12	51	9	23	33	24
	31	26	15	7	14	4
IER3	12	6	4	16	9	15
	23	95	9	12	8	9
IER3IP1	100	595	95	449	221	381
	562	599	269	177	300	93
IER5	23	76	22	47	66	61
	133	354	101	59	151	41
IER5L	12	8	4	18	9	7
	4	11	6	3	5	2
IFFO1	31	158	46	168	47	152
	64	136	75	27	50	20
IFFO2	26	74	35	54	23	67
	19	25	35	11	22	16
IFI16	70	257	56	188	127	155
	198	209	120	115	132	51
IFI27	18	110	12	54	35	33
	120	187	66	65	73	26
IFI27L1	8	25	6	19	6	19
	32	42	16	6	22	9
IFI27L2	1	8	2	9	6	5
	4	6	3	1	6	1
IFI30	6	15	4	7	7	8
	5	22	4	8	18	2

IFI35	10	27	3	20	8	12
	18	24	14	11	17	3
IFI44	24	136	46	134	52	69
	100	141	90	114	76	24
IFI44L	68	218	58	202	107	102
	148	223	94	299	225	34
IFI6	14	47	10	25	13	19
	43	65	19	67	31	8
IFIH1	31	76	27	36	23	41
	48	74	57	60	62	15
IFIT1	128	468	88	265	207	309
	291	317	286	229	222	65
IFIT1B	11	3	7	1	4	1
	1	3	6	0	4	0
IFIT2	21	68	16	57	26	39
	45	42	46	47	41	11
IFIT3	23	122	18	62	53	56
	51	94	60	121	95	17
IFIT5	26	106	27	70	46	80
	69	100	54	44	46	20
IFITM1	23	125	10	59	54	52
	58	141	40	71	52	16
IFITM10	13	5	10	4	7	2
	1	9	3	2	5	1
IFITM2	6	32	4	12	14	15
	5	27	11	15	9	6
IFITM3	15	67	9	40	53	62
	37	87	30	38	40	12
IFITM5	0	0	0	0	0	0
	0	0	3	0	1	0
IFLTD1	41	0	9	0	3	0
	0	0	16	0	11	0
IFNA1	5	0	2	0	0	0
	0	0	0	0	0	0
IFNA10	6	1	7	0	2	0
	0	0	2	0	0	0
IFNA13	4	0	5	0	2	0
	0	0	5	0	0	0
IFNA14	9	0	3	0	1	0
	0	0	2	0	1	0
IFNA16	6	1	2	0	1	0
	0	0	2	0	0	0
IFNA17	2	0	3	0	2	0
	0	0	3	0	1	0
IFNA2	2	0	2	0	1	0
	0	0	2	0	1	0
IFNA21	4	0	1	0	1	0
	0	0	7	0	1	0
IFNA4	6	0	4	0	5	0
	0	0	4	0	1	0
IFNA5	6	3	9	1	1	2
	0	1	3	0	1	1
IFNA6	2	0	0	0	1	0
	0	0	0	0	1	0
IFNA7	3	0	2	0	1	0
	0	0	3	0	2	0
IFNA8	5	0	3	0	2	0
	0	0	3	0	4	0

IFNAR1	101	429	122	390	183	319
	383	363	182	103	204	79
IFNAR2	47	154	21	147	56	89
	127	105	73	41	63	18
IFNB1	3	1	4	0	0	0
	0	0	2	0	0	0
IFNE	1	0	0	0	0	0
	0	0	0	0	2	0
IFNG	13	0	3	0	1	0
	0	1	2	0	3	0
IFNGR1	96	382	82	341	209	337
	444	698	302	146	307	107
IFNGR2	47	160	43	154	88	136
	182	252	90	69	104	35
IFNK	9	0	4	0	3	2
	0	4	6	0	5	0
IFNW1	4	0	1	0	1	0
	0	0	3	0	0	0
IFRD1	199	683	256	568	447	657
	934	1367	437	316	606	115
IFRD2	41	158	19	151	75	154
	180	254	104	72	103	20
IFT122	59	82	41	67	57	69
	62	84	75	31	63	21
IFT140	38	54	24	38	25	30
	28	71	32	29	36	16
IFT172	145	403	134	191	189	283
	354	586	221	161	272	78
IFT20	15	52	21	83	26	46
	48	100	38	28	25	12
IFT27	11	39	12	41	19	41
	31	73	41	11	40	8
IFT43	23	26	11	22	23	21
	36	51	17	9	16	6
IFT46	61	188	65	115	89	135
	158	246	88	65	109	37
IFT52	22	26	15	27	11	22
	22	42	21	8	20	7
IFT57	27	98	36	87	41	94
	69	127	64	21	68	13
IFT74	51	126	42	82	62	118
	121	110	66	50	86	34
IFT80	82	181	69	134	115	134
	135	208	105	84	140	33
IFT81	60	198	47	84	106	109
	161	342	82	72	124	52
IFT88	58	99	72	104	59	113
	60	204	84	58	131	34
IGBP1	106	545	136	374	249	515
	578	879	327	215	341	93
IGDCC3	17	0	11	0	1	0
	0	0	2	0	2	0
IGDCC4	65	252	84	109	114	145
	156	186	106	67	86	43
IGF1	127	420	147	442	188	323
	366	684	228	104	255	98
IGF1R	107	283	61	209	139	189
	245	414	177	143	202	73

IGF2	1	380	4	303	37	338
	1083	183	12	111	79	45
IGF2BP1	31	0	23	0	13	0
	0	1	22	0	8	0
IGF2BP2	68	34	32	12	25	35
	20	23	43	10	26	7
IGF2BP3	28	16	16	7	6	9
	3	2	21	2	14	1
IGF2R	518	2340	421	1644	1095	1636
	1565	2441	890	798	1093	602
IGFALS	2	1	1	0	0	0
	0	0	1	0	0	0
IGFBP1	9	0	4	0	0	0
	0	0	3	0	1	0
IGFBP2	3	31	4	11	16	9
	28	35	18	12	11	6
IGFBP3	40	97	27	85	37	37
	137	143	68	38	47	35
IGFBP4	31	258	36	158	88	94
	152	236	60	53	100	37
IGFBP5	1173	5783	1625	7622	3509	4583
	4593	4990	2702	1405	3127	993
IGFBP6	36	86	19	193	30	125
	31	173	50	14	44	26
IGFBP7	140	487	111	587	314	418
	635	995	516	253	387	192
IGFBPL1	2	0	2	0	4	0
	0	0	1	0	0	0
IGFL1	1	0	3	0	0	0
	0	0	1	0	3	0
IGFL2	6	1	3	1	0	0
	0	0	3	0	0	0
IGFL3	2	0	0	0	0	0
	0	0	0	0	0	0
IGFL4	2	0	0	0	1	0
	0	0	1	0	2	0
IGFLR1	1	3	3	3	0	1
	2	7	0	1	1	1
IGFN1	105	91	113	140	55	548
	872	520	414	282	237	2
IGHMBP2	24	76	15	46	28	37
	45	79	33	25	52	15
IGIP	93	364	90	299	205	344
	290	554	186	112	224	63
IGJ	7	8	15	5	10	3
	7	127	34	20	4	3
IGLL1	4	0	1	0	0	0
	0	0	1	0	0	0
IGLL5	0	1	0	0	0	1
	2	3	0	1	0	0
IGLON5	12	0	8	0	4	0
	0	1	3	0	3	0
IGSF1	47	3	21	4	18	6
	6	6	13	2	6	1
IGSF10	58	45	51	23	26	24
	27	126	48	7	50	9
IGSF11	33	58	36	33	44	36
	34	52	47	19	25	8

IGSF21	13	3	8	6	0	0
	3	6	8	3	4	0
IGSF22	37	2	31	1	6	0
	0	1	11	1	9	0
IGSF23	6	0	0	0	0	0
	0	0	3	0	1	0
IGSF3	58	67	35	38	44	41
	38	59	50	15	27	3
IGSF5	20	1	17	0	3	1
	0	2	12	0	7	0
IGSF6	17	8	5	4	1	4
	4	5	8	1	2	0
IGSF8	19	38	9	20	19	38
	31	68	22	11	24	5
IGSF9	21	0	9	0	7	0
	0	0	5	0	2	0
IGSF9B	32	57	20	26	19	28
	16	31	21	2	13	4
IHH	6	0	2	1	1	0
	0	0	1	0	0	0
IK	141	876	153	481	391	662
	564	840	336	316	465	161
IKBIP	27	34	17	20	24	48
	27	64	33	13	39	6
IKBKAP	144	450	136	336	300	332
	417	616	267	141	287	81
IKBKB	111	454	135	542	214	549
	547	786	343	125	381	119
IKBKE	22	4	21	4	4	3
	2	3	10	0	8	2
IKBKG	22	47	11	29	16	29
	35	59	23	23	37	6
IKZF1 (NC_000007 50344377..50367358)				5	0	1
	0	1	1	2	2	0
	0	2	0			
IKZF1 (NC_000007 50444230..50472799)				30	31	23
	6	13	20	8	27	15
	6	17	2			
IKZF2	133	474	123	326	333	335
	412	490	251	151	257	72
IKZF3	67	11	39	11	24	8
	8	11	48	6	11	2
IKZF4	41	91	26	73	34	63
	82	58	32	18	39	18
IKZF5	15	95	23	60	49	84
	75	74	39	28	72	12
IL10	9	1	8	0	3	0
	2	2	4	0	2	0
IL10RA	20	28	6	22	25	18
	13	58	21	3	26	5
IL10RB	23	42	13	45	25	34
	31	71	40	22	27	16
IL11	3	0	2	0	0	0
	0	0	2	0	2	0
IL11RA	22	97	14	48	43	61
	78	91	31	24	51	14
IL12A	5	0	13	0	5	1
	1	1	7	2	6	0

IL12B	16	0	9	0	2	0
	0	0	7	0	4	0
IL12RB1	27	2	13	1	2	0
	0	0	7	1	7	0
IL12RB2	43	28	27	18	43	41
	61	75	43	29	56	14
IL13	4	0	4	1	0	0
	0	0	3	0	2	0
IL13RA1	122	478	98	486	251	390
	327	558	255	176	295	124
IL13RA2	16	0	4	0	2	0
	1	3	7	0	5	0
IL15	41	63	41	52	51	41
	31	65	49	29	42	9
IL15RA	12	34	12	15	17	20
	22	32	17	9	29	6
IL16	66	99	34	76	46	77
	35	88	61	18	61	11
IL17A	13	0	6	0	3	0
	0	0	0	0	5	0
IL17B	1	1	0	0	0	0
	0	1	0	0	1	0
IL17C	1	0	0	0	0	0
	0	0	1	0	0	0
IL17D	201	390	98	140	266	221
	359	1070	115	205	365	163
IL17F	5	0	3	0	0	0
	0	0	0	0	2	0
IL17RA	51	59	25	41	17	41
	36	42	46	19	38	6
IL17RB	17	14	10	21	12	13
	19	33	21	7	13	4
IL17RC	30	65	18	37	35	50
	68	111	46	20	62	9
IL17RD	59	80	59	91	54	75
	37	58	41	12	51	9
IL17RE	10	4	12	1	2	0
	1	2	7	0	6	2
IL17REL	13	0	6	0	1	0
	0	0	1	0	2	0
IL18	12	38	4	12	19	22
	30	65	41	9	15	9
IL18BP	32	43	22	28	19	43
	26	54	17	17	22	9
IL18R1	43	20	20	17	14	9
	15	16	30	10	24	5
IL18RAP	25	3	15	4	9	1
	2	4	10	0	8	2
IL19	13	0	9	0	2	0
	0	0	6	0	4	0
IL1A	19	2	10	0	5	0
	0	0	18	0	7	0
IL1B	15	6	9	0	3	1
	1	2	8	0	2	0
IL1F10	10	0	3	0	2	0
	0	0	3	0	3	0
IL1R1	79	232	86	194	114	140
	124	258	93	50	124	72

IL1R2	7	1	8	5	0	0
	1	3	5	1	5	0
IL1RAP	36	51	45	50	46	41
	42	39	49	25	30	9
IL1RAPL1	34	12	5	6	30	7
	5	48	12	6	10	3
IL1RAPL2	27	0	4	1	5	0
	3	0	6	1	1	0
IL1RL1	35	8	19	14	20	20
	4	29	16	3	13	5
IL1RL2	17	2	5	2	7	2
	0	5	16	0	4	0
IL1RN	12	0	9	0	1	0
	0	1	10	1	4	0
IL2	9	0	2	0	3	0
	0	0	7	0	3	0
IL20	10	0	5	0	1	0
	1	0	5	0	3	0
IL20RA	16	2	10	3	6	14
	21	6	26	6	12	5
IL20RB	19	13	9	9	7	9
	6	13	14	1	13	3
IL21	2	0	3	0	0	0
	0	0	3	0	2	0
IL21R	22	2	18	0	7	2
	0	0	19	0	11	0
IL22	9	1	5	0	2	0
	0	0	1	0	3	0
IL22RA1	12	1	5	1	1	1
	0	0	14	0	3	0
IL22RA2	16	0	10	0	2	0
	0	0	6	0	5	0
IL23A	7	1	2	0	4	2
	1	4	3	1	1	1
IL23R	21	0	8	1	4	0
	0	0	16	0	4	0
IL24	7	0	4	0	2	0
	0	0	3	0	2	0
IL25	7	0	2	0	1	1
	0	0	0	0	3	0
IL26	11	0	0	0	1	0
	0	0	8	0	3	0
IL27	3	0	2	0	0	0
	0	0	1	0	2	0
IL27RA	16	13	5	5	6	5
	10	21	13	1	6	1
IL28A	3	0	3	0	0	0
	0	0	1	0	3	0
IL28B	2	0	0	0	0	0
	0	0	1	0	1	0
IL28RA	53	34	26	41	47	78
	37	42	69	21	54	4
IL29	3	0	0	0	0	0
	0	0	1	0	0	0
IL2RA	15	3	7	0	3	1
	1	10	6	2	7	0
IL2RB	11	1	8	1	4	2
	5	3	5	2	6	1

IL2RG	14	4	5	3	1	3
	6	4	5	4	2	0
IL3	8	0	2	0	1	0
	0	0	0	0	2	0
IL31	4	0	2	0	1	1
	0	0	1	0	1	0
IL31RA	31	7	27	1	5	1
	21	35	52	4	25	4
IL32	25	410	54	369	70	63
	77	137	228	15	97	31
IL33	52	138	40	96	68	107
	104	204	77	46	93	41
IL34	11	9	7	7	7	6
	6	7	10	1	11	1
IL36A	2	0	1	0	3	0
	0	0	3	0	2	0
IL36B	24	0	9	0	2	0
	0	0	6	0	4	0
IL36G	7	0	10	0	1	0
	0	0	4	0	4	0
IL36RN	15	0	9	0	1	0
	0	0	5	0	4	0
IL37	7	0	4	0	0	0
	0	0	2	0	3	0
IL3RA (NC_000023 1455508..1501582)				9	11	4
	9	4	11	12	17	5
	2	2	8			
IL3RA (NC_000024 1405508..1451582)				6	16	3
	8	1	6	7	16	5
	6	10	2			
IL4	3	0	1	1	1	0
	0	0	1	0	1	0
IL4I1	15	6	7	7	8	3
	2	6	9	2	11	1
IL4R	23	36	22	30	25	21
	14	42	13	9	17	6
IL5	3	1	1	2	3	0
	0	0	7	0	4	0
IL5RA	56	2	23	2	10	0
	0	7	12	1	13	0
IL6	8	5	6	5	1	1
	1	1	3	0	4	1
IL6R	118	239	81	265	167	268
	239	260	104	137	152	122
IL6ST	660	3037	585	2223	1616	2045
	2446	3930	1511	873	1678	799
IL7	16	2	5	4	4	8
	0	8	7	1	6	0
IL7R	43	57	22	22	11	15
	8	33	15	4	25	4
IL8	4	0	3	1	5	0
	3	1	4	1	2	1
IL9	6	0	2	0	0	0
	0	0	4	0	1	0
IL9R (NC_000023 155227245..155240482)				3	0	8
	0	3	0	1	0	2
	0	1	0			

IL9R (NC_000024 59330251..59343488)				4	0	2
	0	0	0	1	1	2
	0	0	0			
ILDR1	19	0	12	0	3	0
	0	0	9	0	7	0
ILDR2	41	22	25	12	17	28
	18	22	33	9	33	1
ILF2	111	555	103	431	288	456
	578	748	276	160	297	97
ILF3	128	820	155	502	302	547
	634	743	254	242	363	157
ILK	65	323	45	188	137	190
	198	358	161	99	164	61
ILKAP	34	192	36	157	80	91
	186	251	89	59	92	37
ILVBL	17	20	4	27	8	20
	33	28	26	9	26	2
IMMP1L	15	44	8	34	25	24
	38	67	22	11	28	8
IMMP2L	31	88	35	65	42	81
	80	142	61	28	55	13
IMMT	234	2008	251	1151	736	1118
	1703	1657	597	506	910	222
IMP3	37	174	29	129	91	146
	151	324	81	44	82	23
IMP4	20	90	30	57	48	63
	96	107	63	30	54	15
IMPA1	75	224	40	176	108	154
	253	277	116	41	128	28
IMPA2	107	752	57	379	266	367
	352	473	159	118	228	65
IMPACT	68	354	89	276	81	247
	311	285	150	75	181	37
IMPAD1	264	1484	336	1176	654	1120
	1067	1332	661	342	810	276
IMPDH1	17	33	11	14	19	19
	11	33	16	8	12	4
IMPDH2	105	501	167	371	256	548
	502	817	401	251	376	113
IMPG1	32	0	22	1	11	0
	0	0	10	0	12	0
IMPG2	60	15	30	18	28	12
	15	24	37	10	19	2
INA	18	0	6	0	0	0
	0	0	2	0	1	0
INADL	538	1962	622	1603	1718	1227
	1606	1653	929	828	1260	362
INCA1	5	21	11	15	7	15
	10	25	10	10	14	2
INCENP	25	44	26	27	25	34
	18	38	36	20	36	11
INF2	25	59	27	58	22	38
	29	55	25	18	23	14
ING1	17	73	20	22	31	22
	56	40	23	22	51	12
ING2	13	35	14	57	19	58
	95	53	27	30	46	10

ING3	44	193	58	120	98	127
	189	250	116	70	173	27
ING4	30	124	30	92	67	103
	114	182	75	41	74	22
ING5	36	77	16	48	41	42
	68	136	39	22	63	23
INHA	0	0	2	0	1	1
	0	0	1	0	0	0
INHBA	18	10	11	20	8	12
	8	7	10	4	9	3
INHBB	15	26	9	8	13	14
	15	38	8	17	4	16
INHBC	11	0	3	0	2	1
	0	0	3	0	1	0
INHBE	6	5	7	4	3	3
	1	5	5	1	2	0
INIP	22	59	25	49	30	44
	44	76	27	23	31	11
INMT	21	159	19	130	71	82
	102	183	74	54	101	35
INO80	157	573	140	427	301	414
	437	603	309	188	364	97
INO80B	16	78	14	51	30	48
	72	93	29	24	34	13
INO80C	59	33	33	28	37	30
	32	46	19	14	32	8
INO80D	321	1387	300	973	630	933
	919	1504	552	390	756	217
INO80E	19	60	13	30	27	48
	36	74	30	15	25	15
INPP1	18	80	20	55	25	63
	94	51	29	24	33	14
INPP4A	100	445	123	293	187	294
	306	373	222	92	238	60
INPP4B	143	579	124	213	229	242
	235	788	226	185	255	115
INPP5A	138	606	103	502	270	348
	482	541	251	209	278	107
INPP5B	72	168	57	108	114	155
	112	247	102	73	115	49
INPP5D (NC_000002 233925035..233995880)				19	24	4
	7	9	12	15	14	8
	2	10	3			
INPP5D (NC_000002 234054794..234116549)				46	37	15
	46	18	32	33	39	33
	3	18	10			
INPP5E	11	36	8	25	18	25
	38	55	23	10	26	11
INPP5F	75	128	65	103	93	138
	105	151	78	58	100	28
INPP5J	18	2	14	7	4	6
	7	8	11	2	9	1
INPP5K	41	101	30	78	55	82
	81	131	58	37	63	23
INPPL1	132	713	146	448	272	515
	473	621	331	166	331	107
INS	1	0	0	0	0	0
	0	0	0	0	0	0

INS-IGF2	5	0	3	0	1	0
	0	0	2	0	0	0
INSC	20	0	8	2	9	0
	0	0	10	0	6	0
INSIG1	40	140	34	131	61	164
	84	126	74	33	65	19
INSIG2	37	200	25	187	96	152
	137	198	84	51	95	28
INSL3	0	0	0	0	0	0
	0	0	0	1	0	0
INSL4	6	0	3	1	1	0
	0	0	0	0	0	0
INSL5	5	1	2	0	3	0
	0	0	1	0	2	0
INSL6	3	0	2	0	0	0
	0	0	0	0	1	0
INSM1	3	0	1	0	2	0
	0	0	2	0	2	0
INSM2	8	0	9	0	0	0
	0	0	4	0	0	0
INSR	342	1272	203	752	699	777
	1156	2674	821	463	975	384
INSRR	30	0	17	2	1	0
	0	0	12	0	3	0
INTS1	87	387	81	230	147	232
	204	331	148	113	174	74
INTS10	58	245	81	192	102	217
	215	244	135	54	174	61
INTS12	37	137	27	91	60	114
	114	163	74	49	62	14
INTS2	77	287	84	173	112	202
	171	266	145	56	145	49
INTS3	111	405	119	262	190	252
	272	387	175	125	230	91
INTS4	82	201	60	113	85	78
	123	190	90	52	82	45
INTS5	18	25	18	23	19	31
	24	37	24	7	24	3
INTS6	125	388	116	246	228	274
	294	321	180	102	204	56
INTS7	65	164	60	108	98	100
	112	159	97	46	93	27
INTS8	87	395	90	244	246	270
	253	476	174	137	218	69
INTS9	42	81	28	51	42	65
	43	61	63	15	44	12
INTU	49	83	32	48	44	76
	47	105	61	27	76	17
INVS	74	216	76	144	126	204
	137	221	121	79	139	48
IP6K1	42	90	43	96	44	54
	123	180	80	48	64	24
IP6K2	56	257	44	151	108	156
	155	306	100	71	106	30
IP6K3	236	704	180	531	474	685
	1027	922	337	275	446	255
IPCEF1	34	11	20	5	5	5
	6	17	30	0	14	1

IPMK	56	140	48	114	73	77
	129	157	84	38	74	31
IPO11	88	350	101	244	165	239
	216	386	144	87	199	58
IPO13	210	764	139	563	357	637
	575	747	398	232	404	129
IPO4	31	119	39	70	51	72
	66	112	62	39	70	24
IPO5	438	2690	541	2224	1109	1946
	2115	3143	1392	753	1624	485
IPO7	625	3573	605	2293	1831	2591
	2523	3617	1111	1215	1548	601
IPO8	142	539	174	459	276	421
	468	659	263	171	330	107
IPO9	174	599	157	476	303	562
	423	576	304	216	362	143
IPP	75	295	71	238	124	212
	161	214	139	86	176	49
IPPK	42	97	28	76	45	74
	96	89	71	25	72	15
IQCA1	40	1	17	5	9	5
	4	0	25	0	8	1
IQCB1	49	137	46	73	73	104
	106	156	70	46	116	20
IQCC	13	9	5	11	13	19
	8	7	6	10	10	2
IQCD	10	2	6	2	0	0
	2	2	3	0	4	0
IQCE	30	68	32	45	33	45
	44	55	42	19	35	12
IQCF1	9	0	2	0	1	0
	0	0	1	0	1	0
IQCF2	1	0	5	0	0	0
	0	0	1	0	1	0
IQCF3	27	0	7	1	2	0
	2	0	12	0	7	0
IQCF5	2	0	1	0	0	0
	0	0	2	0	0	0
IQCF6	0	0	0	0	0	0
	0	0	1	0	0	0
IQCG	36	38	24	29	17	33
	36	51	28	6	22	4
IQCH	31	10	35	10	9	11
	2	3	20	2	21	1
IQCJ	0	0	0	0	0	1
	0	0	0	0	0	0
IQCJ-SCHIP1		109	188	92	106	135
	122	156	200	132	70	156
	41					
IQCK	23	52	9	35	26	40
	42	61	24	12	32	6
IQGAP1	316	1388	308	807	549	914
	806	2040	650	412	643	317
IQGAP2	71	58	43	40	64	48
	34	130	64	19	55	22
IQGAP3	52	3	26	1	7	0
	1	2	18	0	15	1

IQSEC1	81	627	94	303	261	294
	387	367	171	91	182	75
IQSEC2	35	48	17	47	18	39
	69	56	29	11	35	8
IQSEC3	42	9	26	3	7	8
	25	7	17	5	13	0
IQUB	33	26	20	9	17	16
	1	20	27	3	21	6
IRAK1	41	141	35	89	50	136
	126	86	55	44	53	20
IRAK1BP1	6	11	3	11	15	16
	15	22	15	6	14	1
IRAK2	30	35	36	42	31	34
	22	68	34	18	32	5
IRAK3	79	128	73	113	76	96
	167	291	103	46	122	48
IRAK4	28	46	32	39	36	52
	38	52	32	18	24	15
IREB2	218	968	238	985	470	715
	847	789	427	230	562	150
IRF1	45	69	36	39	36	39
	40	75	43	23	49	14
IRF2	73	262	74	190	175	231
	316	398	169	123	244	50
IRF2BP1	12	59	11	48	30	39
	88	72	22	18	56	12
IRF2BP2	156	806	156	653	428	578
	877	1403	658	236	575	226
IRF2BPL	38	122	26	151	80	104
	64	93	41	41	42	10
IRF3	19	39	17	33	20	30
	14	42	18	12	24	4
IRF4	23	2	21	7	12	6
	3	23	21	1	7	1
IRF5	10	3	5	5	3	5
	2	8	8	2	4	2
IRF6	32	3	23	3	6	1
	3	0	27	1	12	1
IRF7	8	12	12	7	9	6
	6	19	16	6	8	2
IRF8	18	17	14	7	9	12
	5	26	9	4	18	3
IRF9	28	72	18	51	33	57
	62	78	51	32	63	9
IRG1	16	0	7	0	3	0
	0	0	6	0	3	0
IRGC	4	0	0	0	0	0
	0	0	1	0	1	0
IRGM	15	0	6	1	2	1
	0	2	10	0	0	1
IRGQ	121	313	80	266	189	234
	268	370	142	125	163	58
IRS1	193	958	251	814	333	818
	749	1029	500	198	487	85
IRS2	46	94	21	74	51	67
	225	461	76	60	178	70
IRS4	16	0	3	0	2	0
	0	0	0	0	1	0

IRX1	3	1	1	0	2	3
	0	0	4	0	3	1
IRX2	11	1	9	1	1	0
	1	1	6	0	3	0
IRX3	8	18	12	39	5	59
	59	21	9	2	11	4
IRX4	3	1	1	2	1	3
	3	1	2	2	5	2
IRX5	8	14	7	4	6	12
	6	20	3	4	2	5
IRX6	10	2	15	0	8	1
	3	22	2	2	6	0
ISCA1	139	709	117	567	276	485
	877	1076	451	191	509	111
ISCA2	33	90	17	60	42	51
	90	159	66	28	82	15
ISCU	317	1794	198	1273	562	1133
	1993	2598	834	416	954	301
ISG15	2	6	1	19	6	4
	15	19	7	6	3	1
ISG20	1	1	1	6	2	3
	4	0	4	2	2	1
ISG20L2	25	43	19	26	28	37
	40	48	32	21	45	17
ISL1	15	1	2	0	2	0
	0	0	3	0	8	0
ISL2	4	0	4	1	1	0
	0	0	3	0	1	0
ISLR	45	154	34	130	82	165
	65	183	48	37	98	25
ISLR2	13	11	7	2	5	3
	6	4	4	2	10	0
ISM1	12	19	14	9	6	15
	16	6	16	8	15	7
ISM2	14	1	7	0	0	0
	0	0	8	0	0	0
ISOC1	40	169	17	78	79	50
	92	97	77	36	81	16
ISOC2	27	128	12	59	49	45
	152	132	40	38	66	17
ISPD	56	91	29	42	34	85
	92	63	43	20	50	6
IST1	212	752	184	588	321	592
	705	911	428	196	562	158
ISX	22	0	6	0	4	0
	0	0	4	0	4	1
ISY1	28	143	36	109	79	152
	145	168	88	39	76	30
ISY1-RAB43	43	120	31	46	43	78
	60	123	65	24	53	20
ISYNA1	6	25	4	11	12	11
	15	11	9	3	4	4
ITCH	185	724	162	601	386	565
	620	875	393	235	397	164
ITFG1	97	639	161	486	305	465
	562	742	280	235	313	96
ITFG2	46	129	45	97	69	101
	95	178	75	51	80	25

ITFG3	37	203	35	110	75	148
	192	222	129	46	114	58
ITGA1	155	818	110	417	286	337
	675	653	430	307	408	147
ITGA10	73	17	34	10	5	19
	13	11	46	4	26	5
ITGA11	54	86	43	122	79	67
	25	73	50	13	55	22
ITGA2	45	19	29	27	27	23
	9	22	40	8	27	3
ITGA2B	20	4	9	0	9	5
	0	6	10	1	4	0
ITGA3	39	106	47	56	37	66
	39	51	34	20	54	16
ITGA4	64	48	39	40	27	23
	20	64	63	18	40	5
ITGA5	58	106	37	68	68	84
	56	107	51	26	57	32
ITGA6	219	1287	230	944	484	561
	962	1259	651	382	623	227
ITGA7	268	1222	193	589	516	795
	708	1278	457	204	398	153
ITGA8	45	109	36	65	42	18
	71	73	51	29	38	25
ITGA9	68	205	140	119	80	94
	78	142	76	30	67	29
ITGAD	30	13	22	7	7	15
	6	11	18	6	8	1
ITGAE	62	55	20	68	25	45
	87	99	54	17	61	11
ITGAL	42	18	14	5	11	4
	12	19	17	7	23	5
ITGAM	26	42	19	47	31	50
	14	64	34	12	17	11
ITGAV	303	1258	341	1136	633	861
	863	1261	613	404	620	302
ITGAX	32	15	17	10	3	4
	11	18	15	3	4	1
ITGB1	590	3471	613	2428	1590	2295
	2310	3407	1428	992	1720	780
ITGB1BP1	32	182	32	109	67	131
	181	196	118	54	87	28
ITGB1BP2	41	269	35	74	64	77
	238	262	78	48	123	31
ITGB2	13	26	15	18	12	17
	18	33	20	7	13	7
ITGB3	36	63	26	57	33	32
	39	47	25	11	25	18
ITGB3BP	32	60	25	41	22	33
	52	123	27	27	69	10
ITGB4	36	46	23	55	24	10
	15	27	25	11	29	24
ITGB5	68	186	45	156	116	159
	118	200	96	68	96	62
ITGB6	383	1461	361	1027	931	1382
	1504	1681	868	535	1072	191
ITGB7	15	6	4	4	5	1
	4	9	12	1	12	1

ITGB8	33	12	27	36	10	4
	10	7	29	10	20	19
ITGBL1	44	106	27	108	84	125
	44	69	80	28	65	35
ITIH1	30	0	13	0	3	0
	0	0	9	0	6	0
ITIH2	31	3	26	0	3	6
	0	10	19	0	12	0
ITIH3	22	8	13	3	5	0
	0	2	7	0	6	3
ITIH4	243	969	176	620	373	1034
	601	1420	614	562	633	193
ITIH5	120	405	63	331	208	326
	319	321	222	101	229	131
ITIH6	45	38	24	26	39	37
	25	48	26	12	32	8
ITK	37	15	23	9	9	3
	10	12	29	3	17	0
ITLN1	7	0	10	0	0	0
	8	0	5	0	3	1
ITLN2	6	0	7	0	2	0
	0	0	1	0	2	0
ITM2A	68	190	34	196	132	114
	118	274	125	59	124	44
ITM2B	260	1321	258	1547	714	1160
	1365	1908	852	490	986	291
ITM2C	14	49	11	28	36	42
	51	76	25	18	21	12
ITPA	17	55	6	30	29	22
	49	52	21	13	26	5
ITPK1	52	103	42	81	61	83
	78	92	77	31	59	19
ITPKA	6	1	1	1	0	1
	0	0	3	0	0	0
ITPKB	62	222	31	123	69	81
	162	129	85	48	71	37
ITPKC	18	30	11	22	16	38
	35	39	28	8	17	6
ITPR1	143	325	124	230	116	212
	192	252	172	81	143	61
ITPR2	250	891	223	516	382	525
	539	996	468	234	487	191
ITPR3	73	134	71	133	59	78
	89	119	64	43	80	15
ITPRIP	32	44	19	28	14	28
	30	30	19	13	25	6
ITPRIPL1	11	4	5	6	6	3
	3	6	9	4	10	2
ITPRIPL2	69	266	65	238	157	234
	239	296	151	124	176	81
ITSN1	173	667	160	434	386	480
	345	650	321	221	345	145
ITSN2	175	544	155	336	302	437
	426	526	261	225	329	152
IVD	72	365	50	124	88	124
	201	356	124	61	154	56
IVL	20	1	6	0	0	0
	0	1	6	0	1	0

IVNS1ABP	109	669	197	627	372	621
	450	538	284	158	409	94
IWS1	127	626	134	326	334	462
	418	624	273	261	304	125
IYD	42	1	17	0	5	0
	0	1	17	0	9	0
IZUMO1	10	2	8	2	3	2
	0	2	5	0	2	0
IZUMO2	2	0	7	0	3	0
	0	0	2	0	2	0
IZUMO3	9	0	5	0	0	0
	0	0	3	0	2	0
IZUMO4	3	2	6	0	1	1
	3	9	4	0	6	0
JAG1	103	457	98	347	137	263
	255	363	171	95	198	80
JAG2	28	159	34	105	45	115
	75	115	68	36	67	14
JAGN1	12	63	18	49	31	49
	69	93	45	20	45	11
JAK1	319	1385	286	1158	1008	1035
	1124	1791	706	533	790	322
JAK2	375	1205	331	551	721	717
	868	2115	600	545	887	327
JAK3	25	9	24	3	6	4
	2	4	20	1	9	1
JAKMIP1	23	0	13	0	7	0
	0	0	12	0	7	0
JAKMIP2	77	4	50	4	15	0
	1	3	35	2	27	1
JAKMIP3	33	47	32	52	28	26
	39	67	24	11	28	12
JAM2	56	274	42	126	84	91
	192	155	83	46	87	36
JAM3	52	117	46	107	34	75
	59	111	48	27	42	31
JARID2	120	312	71	208	151	242
	335	388	186	138	207	55
JAZF1	31	56	19	36	42	56
	34	63	29	17	30	9
JDP2	42	89	33	86	69	143
	92	107	48	27	67	26
JHDM1D	198	1031	252	752	496	736
	978	1047	566	374	617	246
JKAMP	55	249	53	205	146	224
	206	362	174	109	176	44
JMJD1C	413	1764	410	1340	919	1278
	1501	1547	752	567	882	309
JMJD4	11	43	10	42	22	24
	26	78	28	18	31	14
JMJD6	71	257	72	199	202	208
	201	334	115	86	158	57
JMJD7	12	21	10	20	17	6
	11	30	13	6	17	4
JMJD7-PLA2G4B		25	34	28	31	14
	36	21	79	26	18	39
	6					

JMJD8	16	70	19	46	26	48
	60	65	23	21	34	8
JMY	125	553	141	476	271	405
	468	698	327	209	414	142
JOSD1	31	110	44	76	66	118
	106	162	61	41	61	31
JOSD2	5	7	0	13	1	4
	7	13	8	1	5	1
JPH1	512	3955	633	3793	2011	2887
	3189	3145	1331	999	1719	545
JPH2	370	1469	289	1089	764	1138
	1067	1631	720	391	759	275
JPH3	18	2	6	0	6	0
	0	0	10	0	1	0
JPH4	7	0	4	1	2	4
	0	0	7	0	1	1
JRK	66	175	67	114	99	119
	107	185	107	44	95	32
JRKL	27	181	42	93	75	115
	95	99	39	32	71	12
JSRP1	74	387	76	338	176	449
	361	539	296	137	236	74
JTB	98	464	74	354	201	476
	543	762	312	159	251	82
JUN	242	793	245	594	601	695
	989	1137	633	365	781	259
JUNB	11	56	27	64	27	31
	18	66	37	12	21	6
JUND	17	113	9	65	60	55
	80	111	58	51	71	22
JUP	52	222	65	262	125	151
	229	169	81	63	97	45
KAAG1	3	0	1	0	0	0
	0	0	1	0	0	0
KAL1	152	476	109	504	336	579
	490	674	300	188	382	138
KALRN	304	819	262	636	359	503
	706	1253	469	392	790	179
KANK1	161	585	169	353	375	438
	435	509	204	190	280	111
KANK2	54	215	85	183	138	126
	105	213	96	53	138	65
KANK3	9	36	5	28	8	16
	27	40	24	14	25	4
KANK4	31	4	14	17	5	3
	2	3	17	2	9	6
KANSL1	177	761	191	542	248	377
	440	638	247	175	271	138
KANSL1L	79	483	129	278	226	340
	300	533	206	131	260	78
KANSL2	86	286	79	261	138	448
	277	360	248	122	200	69
KANSL3	255	1038	241	876	474	925
	1044	1196	565	370	792	196
KARS	270	1360	322	856	571	1155
	1168	1821	774	529	862	224
KAT2A	58	180	43	97	59	144
	146	205	85	52	69	24

KAT2B	291	1314	316	1000	667	955
	950	1305	581	356	671	248
KAT5	31	110	21	76	67	66
	101	150	53	51	90	31
KAT6A	219	1013	239	700	549	654
	816	968	410	343	527	191
KAT6B	167	785	171	487	399	564
	623	625	307	262	408	98
KAT7	150	630	157	406	341	494
	495	728	312	222	439	132
KAT8	54	142	38	109	87	126
	158	231	82	44	110	33
KATNA1	34	167	46	98	58	124
	135	209	92	37	117	29
KATNAL1	88	343	97	245	171	276
	237	235	143	89	193	56
KATNAL2	36	16	15	19	17	23
	7	20	23	4	16	2
KATNB1	16	40	20	21	18	42
	31	39	18	12	26	6
KATNBL1	34	160	48	152	75	127
	145	244	107	50	91	27
KAZALD1	17	44	7	35	17	18
	24	12	12	11	20	3
KAZN	48	37	27	18	12	23
	7	17	18	2	18	3
KBTBD10	10767	58481	16888	51426	28223	63415
	61595	76666	42644	26011	45402	10968
KBTBD11	30	37	17	146	16	69
	46	66	53	13	40	9
KBTBD12	232	1440	231	858	575	956
	953	1312	530	366	755	194
KBTBD13	76	329	58	245	135	262
	248	254	161	82	206	27
KBTBD2	254	1200	188	1061	640	883
	1155	1572	625	351	762	232
KBTBD3	31	89	32	62	56	58
	83	123	59	28	68	15
KBTBD4	53	155	50	136	98	172
	162	270	114	61	125	34
KBTBD5	252	791	147	412	337	490
	470	1490	471	394	642	185
KBTBD6	51	178	44	133	83	134
	151	161	71	34	77	20
KBTBD7	57	90	26	86	68	117
	109	118	38	30	53	17
KBTBD8	24	36	21	29	25	31
	47	229	73	16	38	7
KCMF1	274	1307	273	1137	596	1196
	1193	1254	679	394	770	227
KCNA1	37	2	23	0	8	1
	0	1	19	0	12	0
KCNA10	6	0	4	0	1	0
	0	0	1	0	0	0
KCNA2	13	8	15	11	9	4
	17	34	13	3	12	1
KCNA3	9	4	7	2	3	0
	1	0	8	1	7	0

KCNA4	21	0	17	1	4	0
	0	2	10	0	3	0
KCNA5	10	18	2	11	13	13
	13	18	10	9	10	2
KCNA6	18	1	13	4	8	2
	2	1	10	0	4	0
KCNA7	79	395	61	294	216	331
	301	519	155	156	201	74
KCNAB1	50	31	25	33	32	24
	23	29	44	18	31	19
KCNAB2	25	13	12	11	4	8
	9	13	13	4	14	4
KCNAB3	11	5	10	2	4	2
	1	3	10	0	3	1
KCNB1	31	120	23	38	14	87
	66	83	50	12	45	25
KCNB2	26	2	9	1	4	0
	0	4	9	0	7	1
KCNC1	39	5	27	2	3	5
	0	9	19	0	15	0
KCNC2	24	0	18	21	7	0
	4	0	21	0	8	0
KCNC3	3	4	3	0	2	0
	1	0	3	1	2	0
KCNC4	17	50	15	68	17	51
	36	23	18	13	17	8
KCND1	19	2	7	1	3	0
	0	1	2	1	1	0
KCND2	41	17	19	24	40	10
	6	30	26	5	25	7
KCND3	28	52	31	22	24	29
	29	46	38	13	40	10
KCNE1	19	0	14	0	5	0
	0	2	18	0	9	0
KCNE1L	3	0	3	32	1	0
	9	0	2	2	5	0
KCNE2	9	6	4	17	6	5
	0	4	2	0	3	0
KCNE3	14	16	9	10	10	12
	10	16	12	6	7	2
KCNE4	17	28	17	15	10	12
	12	26	13	10	11	2
KCNF1	3	0	2	0	0	0
	0	0	1	0	0	0
KCNG1	5	0	0	0	2	1
	1	0	3	0	0	0
KCNG2	0	0	1	1	0	0
	0	0	0	0	0	0
KCNG3	9	0	5	3	7	0
	0	1	6	0	1	0
KCNG4	7	0	8	0	0	1
	0	0	2	0	3	0
KCNH1	47	3	25	8	9	9
	1	13	33	3	16	1
KCNH2	14	0	12	2	3	1
	1	0	4	1	7	0
KCNH3	14	2	3	0	2	0
	0	0	3	1	2	0

KCNH4	17	0	12	1	3	0
	0	1	3	1	2	0
KCNH5	21	0	15	0	9	0
	0	0	15	0	4	1
KCNH6	13	1	8	0	1	0
	0	0	3	0	3	0
KCNH7	18	0	25	1	13	0
	0	0	14	0	5	0
KCNH8	61	3	24	2	13	6
	2	2	21	0	17	3
KCNIP1	17	0	13	0	2	0
	0	1	13	1	5	0
KCNIP2	24	59	17	22	32	32
	39	52	23	14	26	21
KCNIP3	6	3	5	4	3	4
	7	13	4	1	7	3
KCNIP4	23	1	13	2	7	2
	2	4	14	1	10	0
KCNJ1	17	0	15	1	4	0
	0	0	10	0	11	0
KCNJ10	34	0	8	0	2	0
	0	0	7	0	5	1
KCNJ11	45	226	60	179	99	156
	107	207	74	79	101	42
KCNJ12	125	756	119	504	364	457
	452	456	288	163	301	74
KCNJ13	19	3	17	3	8	3
	2	11	13	0	8	0
KCNJ14	17	8	8	6	8	1
	1	3	10	2	5	2
KCNJ15	21	11	15	4	4	0
	4	0	8	1	3	1
KCNJ16	25	30	26	7	19	6
	8	6	14	3	10	7
KCNJ2	212	1350	394	559	467	516
	686	924	436	180	450	116
KCNJ3	18	4	23	11	17	8
	41	14	15	18	15	8
KCNJ4	7	0	1	0	3	0
	1	0	2	0	0	0
KCNJ5	26	10	3	5	3	6
	3	5	11	1	12	2
KCNJ6	11	0	5	0	3	0
	0	0	4	0	2	0
KCNJ8	23	101	11	45	34	38
	52	78	59	23	38	16
KCNJ9	18	0	9	0	6	1
	0	0	7	0	3	0
KCNK1	6	0	4	3	2	0
	1	2	1	0	2	0
KCNK10	50	3	38	5	12	1
	1	0	22	0	12	0
KCNK12	0	0	0	0	0	0
	1	0	0	0	0	1
KCNK13	14	0	2	0	0	0
	0	0	1	0	2	0
KCNK15	5	1	3	0	2	0
	0	5	3	0	3	0

KCNK16	6	0	3	0	1	0
	0	0	1	0	0	0
KCNK17	9	2	1	4	0	1
	0	0	2	1	1	0
KCNK18	7	0	2	0	1	0
	0	0	4	0	0	0
KCNK2	36	19	25	61	22	25
	17	24	36	8	17	9
KCNK3	7	3	8	0	5	1
	0	1	5	0	4	0
KCNK4	2	0	3	0	0	0
	0	0	2	0	0	0
KCNK5	21	2	5	5	1	3
	1	3	7	1	3	0
KCNK6	9	6	5	5	7	3
	8	10	7	2	6	1
KCNK7	4	2	3	1	1	0
	1	2	1	0	1	0
KCNK9	7	2	1	3	0	5
	4	0	6	0	0	0
KCNMA1	320	351	280	638	467	367
	485	841	287	153	512	141
KCNMB1	7	2	4	0	1	0
	2	7	7	1	0	1
KCNMB2	24	1	3	0	8	1
	2	4	6	1	4	1
KCNMB3	27	28	25	28	19	24
	20	46	43	14	35	5
KCNMB4	27	51	17	39	24	23
	47	36	37	13	37	7
KCNN1	4	0	0	0	3	0
	0	0	4	1	3	0
KCNN2	46	100	24	60	53	36
	95	99	49	19	71	7
KCNN3	232	866	304	886	492	751
	484	423	280	166	296	65
KCNN4	8	3	10	0	3	2
	1	2	7	0	2	0
KCNQ1	15	5	4	2	5	2
	5	5	4	1	4	0
KCNQ2	17	0	0	1	1	0
	0	0	5	0	2	0
KCNQ3	58	8	42	2	13	3
	2	2	42	1	17	2
KCNQ4	18	45	17	11	19	33
	23	49	12	12	11	0
KCNQ5	181	698	310	863	286	743
	754	1339	661	265	458	165
KCNRG	9	20	6	17	11	12
	4	20	15	2	12	0
KCNS1	18	0	15	0	4	0
	0	0	9	0	6	0
KCNS2	11	2	10	0	2	0
	0	2	8	0	3	0
KCNS3	112	857	189	780	376	636
	664	583	310	131	512	77
KCNT1	44	150	46	81	58	116
	134	219	63	50	89	11

KCNT2	52	28	40	33	41	18
	12	24	46	15	32	9
KCNU1	34	0	30	0	11	0
	0	0	21	0	10	0
KCNV1	9	0	7	0	2	0
	0	0	6	0	2	1
KCNV2	10	0	16	0	9	0
	0	1	6	0	7	1
KCP	24	4	15	4	4	1
	0	0	12	0	6	0
KCTD1	21	33	16	17	11	17
	7	25	13	4	13	5
KCTD10	35	109	22	98	41	76
	118	126	62	41	68	20
KCTD11	12	14	11	24	8	22
	26	24	21	8	16	5
KCTD12	116	775	161	729	269	449
	602	891	422	214	383	160
KCTD13	8	13	2	15	5	10
	22	11	13	7	7	2
KCTD15	17	31	11	8	10	16
	25	20	25	11	17	2
KCTD16	20	1	18	9	7	6
	0	0	20	0	7	1
KCTD17	11	6	6	2	4	1
	6	7	6	0	3	1
KCTD18	42	103	20	88	50	60
	91	99	53	23	45	16
KCTD19	27	0	6	0	2	0
	0	0	18	0	8	0
KCTD2	47	200	46	128	81	112
	145	247	92	63	98	45
KCTD20	145	510	110	454	275	386
	379	541	232	158	290	110
KCTD21	28	40	20	27	19	30
	32	20	14	11	14	8
KCTD3	91	351	97	341	184	248
	278	417	219	99	234	89
KCTD4	10	0	6	1	3	0
	2	0	7	0	2	1
KCTD5	37	85	35	75	51	63
	62	81	39	28	49	14
KCTD6	20	53	15	46	37	56
	71	91	36	16	39	13
KCTD7	41	91	37	75	40	71
	83	141	55	32	80	16
KCTD8	11	19	9	21	20	24
	40	36	14	9	17	5
KCTD9	91	537	104	545	193	333
	543	599	230	108	325	74
KDELC1	15	18	11	11	14	18
	14	11	12	4	20	2
KDELC2	62	155	44	114	90	127
	116	151	84	59	80	39
KDELR1	31	148	16	85	48	86
	105	146	55	39	65	22
KDELR2	82	338	71	374	167	282
	295	404	160	107	182	63

KDELR3	12	22	12	22	13	22
	5	14	16	9	9	2
KDM1A	150	594	151	444	304	520
	484	725	343	182	402	118
KDM1B	47	168	46	75	55	83
	81	113	67	51	65	20
KDM2A	193	1002	215	643	423	629
	714	869	370	306	441	187
KDM2B	65	162	54	151	80	123
	123	197	75	60	104	25
KDM3A	143	506	147	426	310	315
	464	706	252	182	343	125
KDM3B	274	1285	230	1077	664	945
	876	1106	559	335	612	234
KDM4A	133	402	99	263	148	233
	194	357	132	108	208	66
KDM4B	53	176	58	119	85	144
	143	157	80	62	102	52
KDM4C	132	363	120	293	202	283
	310	414	220	115	220	54
KDM4D	13	6	8	11	4	6
	8	12	13	4	6	0
KDM4E	9	1	6	0	0	0
	0	0	2	0	1	0
KDM5A	247	1099	277	727	568	904
	756	1344	544	379	706	230
KDM5B	86	261	57	149	146	182
	142	263	127	67	184	50
KDM5C	175	471	128	385	349	421
	424	527	223	244	305	95
KDM5D	1	849	223	665	3	573
	714	1232	474	0	606	178
KDM6A	153	343	88	255	276	222
	261	480	211	196	212	82
KDM6B	30	112	23	40	36	52
	51	58	20	17	30	15
KDM8	13	25	11	19	5	14
	17	16	14	3	13	5
KDR	89	240	57	140	102	110
	179	218	109	68	122	49
KDSR	60	147	52	156	89	131
	156	207	76	41	79	30
KEAP1	120	422	79	381	281	400
	455	443	288	198	279	127
KEL	21	6	11	3	5	13
	10	6	25	5	12	1
KERA	8	0	6	2	6	3
	0	3	8	0	6	0
KHDC1	8	7	6	7	5	7
	2	9	6	1	7	1
KHDC1L	2	0	0	0	1	0
	0	0	0	0	1	0
KHDC3L	4	0	0	0	0	0
	0	0	3	0	0	0
KHDRBS1	68	609	86	369	230	300
	420	567	173	124	226	73
KHDRBS2	10	4	11	1	3	6
	7	6	6	0	5	0

KHDRBS3	45	184	56	119	98	114
	129	159	78	90	92	43
KHK	17	13	21	9	6	19
	8	7	14	3	9	1
KHNYN	91	258	77	228	160	221
	222	297	148	100	177	60
KHSRP	54	317	45	170	128	150
	217	252	92	80	144	46
KIAA0020	57	229	71	178	114	160
	175	255	113	84	165	53
KIAA0040	44	189	41	99	74	112
	166	238	97	56	100	38
KIAA0100	316	1724	303	1078	717	1067
	1067	1402	615	442	784	305
KIAA0101	5	0	1	1	1	2
	1	0	4	1	2	0
KIAA0141	98	231	61	220	131	147
	203	326	133	90	177	45
KIAA0146	56	141	59	97	63	85
	128	179	102	55	80	24
KIAA0195	121	450	110	295	189	315
	315	447	166	131	226	84
KIAA0196	104	314	90	262	187	213
	261	337	193	118	227	67
KIAA0226	80	187	50	151	81	180
	105	162	85	45	80	17
KIAA0226L	25	6	9	7	14	13
	4	5	17	2	9	2
KIAA0232	429	2084	388	1421	1263	1450
	2012	2539	781	829	1362	444
KIAA0240	98	504	120	427	220	331
	375	455	181	150	225	108
KIAA0247	45	164	52	131	62	99
	112	157	76	43	83	28
KIAA0284	26	28	21	17	17	31
	17	12	24	13	13	8
KIAA0317	102	320	93	259	160	285
	281	379	183	85	225	54
KIAA0319	63	0	26	2	9	0
	1	1	30	0	16	1
KIAA0319L	155	757	151	567	343	498
	593	680	356	221	428	124
KIAA0355	125	436	122	264	221	256
	280	448	230	153	256	77
KIAA0368	1182	6095	1176	4511	3289	4356
	3925	5899	2573	2086	3704	1178
KIAA0391	51	257	50	145	98	171
	186	292	113	68	140	48
KIAA0408	63	1008	45	504	357	494
	1290	1629	213	440	532	232
KIAA0430	202	847	219	666	417	547
	607	875	351	240	421	165
KIAA0494	202	1024	188	803	500	771
	837	879	382	317	437	182
KIAA0513	48	159	47	118	57	120
	97	87	63	26	63	28
KIAA0528	132	428	160	379	212	397
	508	608	341	141	348	93

KIAA0556	71	48	31	40	15	40
	37	55	49	15	31	14
KIAA0586	91	380	104	214	198	242
	252	359	206	121	219	64
KIAA0664	144	534	115	432	232	435
	526	541	280	194	376	124
KIAA0753	62	59	28	47	44	64
	52	79	39	26	52	25
KIAA0754	0	0	1	1	0	0
	1	0	0	0	0	1
KIAA0825	39	53	44	28	34	47
	25	82	61	16	77	10
KIAA0895	28	37	23	22	41	38
	31	38	35	11	20	9
KIAA0895L	17	26	14	20	12	40
	25	39	12	9	21	3
KIAA0907	78	297	96	235	163	272
	227	467	185	97	247	66
KIAA0913	106	331	95	216	176	227
	247	389	160	117	214	69
KIAA0922	269	1268	254	929	508	861
	864	1437	685	423	838	183
KIAA0930	59	217	41	240	77	302
	139	139	59	40	81	41
KIAA0947	230	914	212	694	470	595
	702	993	453	302	593	188
KIAA1009	69	126	64	100	85	57
	115	211	84	45	89	23
KIAA1024	38	5	13	2	9	0
	2	24	12	4	12	7
KIAA1024L	10	0	5	0	5	0
	0	0	1	0	5	0
KIAA1033	200	884	239	774	468	662
	887	1085	415	286	467	166
KIAA1045	25	3	12	1	3	0
	0	0	12	0	7	0
KIAA1107	41	63	33	45	52	46
	101	135	93	44	95	29
KIAA1109	747	3445	892	2757	1836	2443
	2833	4596	2224	1093	2537	671
KIAA1143	145	489	128	452	262	499
	384	586	291	173	380	104
KIAA1147	112	357	102	268	184	289
	315	542	178	145	251	91
KIAA1161	81	508	55	405	211	424
	352	196	160	161	204	54
KIAA1191	121	552	81	411	221	314
	544	554	271	152	370	91
KIAA1199	43	8	30	2	11	2
	2	2	33	0	21	1
KIAA1210	43	6	11	5	8	1
	0	0	17	0	13	0
KIAA1211	48	0	15	0	7	1
	4	5	23	0	8	0
KIAA1211L	18	25	19	18	6	13
	22	16	20	5	4	6
KIAA1217	333	1653	313	803	644	1084
	1735	2315	721	696	1093	489

KIAA1239	43	0	34	0	12	5
	0	3	30	0	7	0
KIAA1244	100	56	57	40	51	26
	57	49	76	27	50	14
KIAA1257	16	0	10	0	1	1
	3	3	9	0	3	0
KIAA1279	61	551	81	167	151	268
	237	356	159	105	182	60
KIAA1324	25	4	21	0	11	1
	0	2	19	2	14	0
KIAA1324L	86	259	81	124	99	106
	184	262	97	130	110	46
KIAA1328	51	123	55	93	63	97
	72	134	59	32	96	24
KIAA1377	44	73	41	62	47	46
	40	55	41	20	37	24
KIAA1383	37	176	40	110	67	95
	97	103	88	39	77	15
KIAA1407	39	55	31	44	35	43
	23	52	44	12	48	11
KIAA1429	154	555	147	416	297	420
	397	727	268	158	342	108
KIAA1430	72	227	58	186	146	191
	197	319	119	87	146	54
KIAA1432	161	488	173	463	259	339
	429	607	334	174	360	139
KIAA1456	57	106	84	79	82	50
	50	101	88	58	98	18
KIAA1462	129	393	54	294	170	211
	415	361	194	131	263	96
KIAA1467	40	42	19	31	19	45
	31	46	34	12	27	10
KIAA1468	109	353	120	268	159	252
	277	441	176	129	249	65
KIAA1522	14	21	11	29	10	14
	20	35	15	5	22	3
KIAA1524	38	23	27	29	24	24
	31	48	61	14	46	11
KIAA1549	88	32	54	44	30	39
	16	20	57	8	28	8
KIAA1549L	81	7	49	10	14	1
	3	10	43	7	22	3
KIAA1551	167	795	167	422	334	476
	524	600	288	263	378	141
KIAA1586	36	68	39	125	95	120
	96	156	79	42	66	23
KIAA1598	43	49	38	45	40	62
	23	48	46	11	38	13
KIAA1609	42	68	27	43	33	41
	46	78	42	10	35	6
KIAA1614	16	5	15	9	4	10
	1	6	8	0	6	3
KIAA1644	39	1	21	1	8	0
	1	3	15	0	21	1
KIAA1671	163	677	149	626	133	461
	376	380	272	130	275	115
KIAA1683	9	4	5	16	5	18
	7	3	13	10	9	4

KIAA1704	34	127	50	83	82	62
	77	141	52	49	77	28
KIAA1715	214	1226	355	1117	806	1007
	791	1000	648	433	918	200
KIAA1731	98	312	111	206	166	204
	228	328	150	117	208	69
KIAA1737	484	2108	366	1709	1173	1598
	1819	2285	1022	674	1284	379
KIAA1751	22	2	14	0	3	2
	3	3	4	0	4	0
KIAA1755	30	6	16	17	2	6
	10	5	14	1	7	0
KIAA1804	18	3	13	2	8	6
	1	3	18	0	9	0
KIAA1841	62	110	48	78	75	78
	101	197	70	71	104	43
KIAA1919	52	242	63	160	157	164
	105	333	128	65	129	37
KIAA1958	20	32	8	11	14	14
	21	24	20	7	10	7
KIAA1967	55	220	68	156	119	183
	162	251	125	71	151	37
KIAA1984	14	9	16	7	7	3
	9	11	9	6	11	1
KIAA2013	60	218	40	213	154	256
	265	268	161	107	175	47
KIAA2018	507	2391	534	2160	1353	1852
	1496	1943	773	666	1109	348
KIAA2022	52	6	19	8	20	2
	5	6	15	1	9	4
KIAA2026	226	1084	236	747	529	810
	800	913	383	385	512	182
KIDINS220	364	1555	412	984	730	1150
	1117	1880	809	594	1029	311
KIF11	24	7	22	10	14	10
	13	15	29	2	21	3
KIF12	9	0	5	0	2	0
	0	0	2	0	3	0
KIF13A	603	2648	607	1969	1687	2050
	2184	2851	1231	999	1722	622
KIF13B	116	312	124	223	128	289
	199	337	194	89	216	72
KIF14	52	4	25	4	16	4
	0	2	34	2	24	1
KIF15	56	7	27	4	22	7
	6	5	41	0	27	4
KIF16B	98	329	105	236	184	232
	232	317	211	124	182	75
KIF17	8	9	11	4	8	3
	8	12	12	6	8	3
KIF18A	34	7	32	4	21	7
	5	10	33	3	22	2
KIF18B	12	0	13	0	0	0
	0	1	13	0	8	0
KIF19	16	0	5	2	5	0
	0	0	3	0	3	0
KIF1A	41	0	24	5	6	1
	0	0	13	0	3	2

KIF1B	1954	11780	2360	10441	6751	10157
	8482	10862	5167	3389	7081	2134
KIF1C	889	5276	714	3486	2068	3450
	3460	4868	1960	1572	2650	1002
KIF20A	36	2	16	7	9	0
	1	4	18	0	8	0
KIF20B	69	59	62	69	54	77
	51	105	85	33	75	21
KIF21A	183	758	206	483	324	572
	498	532	264	262	248	84
KIF21B	40	12	30	13	15	10
	4	7	10	3	18	4
KIF22	24	54	21	55	24	53
	72	63	38	11	50	12
KIF23	34	1	22	3	8	1
	2	0	20	1	16	2
KIF24	26	16	28	11	21	12
	9	9	31	5	27	6
KIF25	9	9	6	3	5	0
	7	1	5	1	6	0
KIF26A	17	27	16	18	6	12
	18	19	17	8	11	4
KIF26B	29	10	14	7	5	1
	1	0	16	3	8	0
KIF27	89	227	64	120	107	107
	173	281	99	74	186	36
KIF2A	55	198	59	140	75	126
	115	184	72	45	71	33
KIF2B	8	0	8	0	4	0
	0	0	3	0	3	0
KIF2C	26	5	13	1	5	0
	2	5	23	2	10	1
KIF3A	71	134	45	104	70	139
	114	136	77	56	88	27
KIF3B	38	97	32	70	81	69
	55	109	72	30	58	28
KIF3C	24	21	16	12	15	18
	10	13	14	3	12	8
KIF4A	53	1	15	1	15	1
	3	0	13	1	7	0
KIF4B	43	6	25	6	8	17
	1	10	21	2	10	0
KIF5A	38	0	27	0	9	1
	0	0	13	0	11	0
KIF5B	545	3439	632	2600	1614	2553
	2124	3181	1247	1040	1729	680
KIF5C (NC_000002 149632791..149686923)				4	0	1
	0	1	0	0	0	1
	0	4	0			
KIF5C (NC_000002 149793795..149883273)				45	18	33
	12	26	14	12	16	27
	3	19	5			
KIF6	40	0	27	2	7	0
	4	2	28	1	12	1
KIF7	14	16	9	11	9	9
	8	19	17	2	6	5
KIF9	31	25	19	7	10	14
	17	17	24	3	18	7

KIFAP3	91	416	144	284	178	325
	291	417	216	135	243	72
KIFC1	10	4	11	3	5	0
	0	3	6	0	7	0
KIFC2	12	18	15	15	6	20
	9	15	7	7	10	6
KIFC3	17	33	16	15	13	25
	4	13	14	4	24	2
KIN	100	332	65	191	122	284
	178	417	169	129	251	65
KIR2DL1	22	0	8	0	0	0
	0	0	3	0	6	0
KIR2DL3	12	0	10	1	5	0
	3	0	9	0	3	0
KIR2DL4	15	0	5	0	0	1
	0	0	12	0	3	0
KIR2DS4	22	0	11	0	4	0
	0	1	7	0	4	0
KIR3DL1	16	0	10	0	2	0
	0	0	7	0	5	1
KIR3DL2	17	0	11	0	3	0
	0	1	2	0	8	0
KIR3DL3	6	0	6	0	5	0
	0	0	5	0	2	0
KIRREL	24	33	24	33	19	24
	18	29	25	13	21	15
KIRREL2	21	0	7	0	2	0
	0	0	4	0	1	0
KIRREL3	23	0	14	0	6	0
	0	0	13	0	7	0
KISS1	3	0	2	0	0	0
	0	0	0	0	0	0
KISS1R	0	0	0	0	0	0
	0	0	0	0	1	0
KIT	50	35	33	63	33	75
	37	88	47	17	31	16
KITLG	73	324	74	235	148	152
	201	279	173	66	139	37
KL	31	61	22	40	23	37
	50	36	32	12	25	8
KLB	37	16	13	7	25	4
	6	5	12	2	11	18
KLC1	192	1133	234	776	540	764
	807	1120	531	320	543	213
KLC2	19	77	19	51	37	43
	56	54	28	21	25	11
KLC3	4	0	7	0	0	2
	0	0	3	0	1	0
KLC4	50	121	24	86	85	123
	101	156	74	45	80	10
KLF1	1	2	0	0	0	7
	0	0	2	0	1	0
KLF10	135	295	173	385	354	522
	1149	859	329	508	424	294
KLF11	49	103	33	91	51	82
	128	153	55	42	77	26
KLF12	222	568	128	452	352	470
	402	783	370	169	434	145

KLF13	106	345	116	324	254	284
	602	592	303	211	416	267
KLF14	3	1	1	3	0	4
	5	3	2	0	4	0
KLF15	28	170	14	13	67	17
	339	652	105	56	253	59
KLF16	4	3	5	6	5	4
	7	6	6	0	2	0
KLF17	35	2	11	1	6	4
	7	3	15	0	13	2
KLF2	5	59	6	31	26	25
	46	46	19	6	9	12
KLF3	80	308	64	309	191	262
	254	352	151	102	169	67
KLF4	7	76	18	72	33	46
	43	62	16	9	42	9
KLF5	41	123	44	152	47	77
	172	152	53	74	34	24
KLF6	78	447	101	358	184	322
	380	414	211	101	179	60
KLF7	91	233	87	189	133	204
	170	309	127	85	141	55
KLF8	49	196	36	98	65	116
	106	90	62	34	73	17
KLF9	279	1317	196	974	981	878
	2084	3210	1047	711	1735	636
KLHDC1	50	199	63	159	141	171
	167	278	110	69	141	41
KLHDC10	167	647	156	511	304	438
	492	752	323	187	416	125
KLHDC2	154	668	178	701	309	590
	725	1148	488	235	541	174
KLHDC3	274	857	199	1124	565	937
	1364	1812	757	432	797	278
KLHDC4	21	56	28	30	27	52
	30	76	19	16	27	11
KLHDC5	40	114	28	81	48	60
	77	109	65	31	67	25
KLHDC7A	21	1	9	1	2	0
	0	0	3	0	4	0
KLHDC7B	3	0	3	0	0	0
	0	0	1	0	1	0
KLHDC8A	11	1	4	2	5	0
	0	0	4	0	1	0
KLHDC8B	38	108	30	63	70	97
	77	240	65	46	65	21
KLHDC9	10	4	5	9	12	9
	8	9	7	0	7	1
KLHL1	27	0	13	0	13	0
	0	1	13	0	9	0
KLHL10	8	1	6	0	1	3
	0	1	5	0	3	0
KLHL11	20	58	20	55	32	60
	72	83	43	16	44	13
KLHL12	78	251	90	182	138	210
	239	295	134	76	155	40
KLHL13	24	64	19	51	48	52
	58	85	51	29	50	4

KLHL14	18	0	13	1	4	1
	0	1	8	0	6	1
KLHL15	88	203	49	176	131	149
	195	237	100	68	136	56
KLHL17	4	4	1	1	4	6
	3	5	6	3	1	1
KLHL18	46	113	36	85	44	60
	61	80	51	20	50	18
KLHL2	46	125	60	58	27	56
	41	81	54	15	47	18
KLHL20	97	364	87	260	168	254
	340	389	175	118	199	76
KLHL21	91	422	61	270	193	279
	409	517	290	134	255	91
KLHL22	45	132	30	101	70	125
	101	161	75	44	95	31
KLHL23	0	7	1	0	1	5
	0	4	3	0	2	0
KLHL24	723	4248	867	3555	1907	3176
	3476	5058	2246	1107	3120	845
KLHL25	11	51	16	22	25	29
	25	32	17	7	15	6
KLHL26	11	31	8	22	12	15
	13	23	12	5	17	5
KLHL28	144	522	173	425	293	415
	436	605	245	199	367	133
KLHL29	25	63	18	29	25	38
	35	37	24	14	22	7
KLHL3	77	96	62	67	38	57
	61	120	74	24	67	28
KLHL30	94	289	45	242	110	368
	225	289	187	140	162	67
KLHL31	1282	9906	1249	6684	4518	5224
	5848	5697	2530	1682	3205	911
KLHL32	26	9	15	8	9	19
	6	7	34	2	14	2
KLHL33	66	444	110	359	177	299
	258	321	191	87	184	40
KLHL34	21	42	9	27	16	35
	94	122	45	37	72	23
KLHL35	5	3	0	1	0	1
	0	0	2	0	0	0
KLHL36	8	19	8	24	12	27
	21	31	20	5	16	8
KLHL38	97	499	59	394	241	253
	805	275	151	126	184	55
KLHL4	50	9	16	9	11	2
	4	10	17	0	11	1
KLHL5	152	414	87	305	244	267
	353	367	224	123	227	64
KLHL6	38	22	25	23	15	20
	19	17	25	2	12	0
KLHL7	64	247	65	189	113	187
	187	214	89	66	125	53
KLHL8	121	548	131	413	306	503
	367	526	246	157	312	77
KLHL9	46	237	64	181	150	206
	245	326	166	103	169	63

KLK1	2	1	0	0	0	0
	0	0	0	0	2	0
KLK10	13	0	15	0	2	0
	0	0	6	0	4	0
KLK11	2	0	7	0	0	0
	0	0	1	0	1	0
KLK12	6	0	6	0	1	0
	0	0	10	0	1	0
KLK13	27	0	18	3	15	0
	0	0	7	0	8	0
KLK14	11	0	6	1	3	0
	0	0	6	0	1	0
KLK15	7	0	6	0	0	0
	0	0	0	0	3	0
KLK2	12	0	14	1	5	0
	0	0	6	0	2	0
KLK3	5	0	4	0	1	0
	2	0	4	0	3	1
KLK4	3	0	4	1	0	0
	0	0	2	0	3	0
KLK5	9	1	3	0	0	0
	0	0	1	0	4	0
KLK6	12	0	10	0	2	0
	0	0	7	0	2	1
KLK7	6	0	4	0	1	0
	0	0	5	0	6	0
KLK8	5	0	2	1	0	0
	0	0	4	0	1	0
KLK9	1	0	1	0	0	0
	0	0	0	0	0	0
KLKB1	23	162	13	95	23	65
	76	57	32	20	43	2
KLLN	16	5	13	6	2	1
	6	2	8	0	6	0
KLRB1	11	2	6	1	2	1
	0	2	3	0	2	0
KLRC1	16	1	5	3	3	3
	5	0	13	1	3	0
KLRC2	6	2	9	3	6	3
	2	1	5	0	3	0
KLRC3	11	1	1	0	4	0
	0	1	9	2	4	0
KLRC4	0	1	1	1	0	0
	2	0	0	0	0	0
KLRC4-KLRK1		28	14	8	8	13
	12	6	13	16	7	26
	1					
KLRD1	21	3	10	0	2	2
	3	9	15	0	6	2
KLRF1	7	3	9	3	3	3
	8	1	7	0	6	0
KLRF2	4	0	2	0	2	0
	0	0	6	0	1	0
KLRG1	17	28	14	29	20	34
	18	28	27	8	30	5
KLRG2	6	0	2	1	2	0
	0	0	2	0	3	0

KLRK1	0	4	0	3	0	0
	0	0	0	2	0	1
KMO	33	6	25	2	12	5
	3	4	19	1	12	0
KNCN	15	0	3	0	1	0
	0	0	2	0	3	0
KNDC1	21	0	12	1	1	5
	0	0	5	0	3	0
KNG1	25	0	12	0	9	0
	0	0	15	0	5	0
KNTC1	92	129	75	86	51	94
	60	109	91	49	91	29
KPNA1	279	1818	253	1520	675	1303
	1270	1618	704	421	807	232
KPNA2	33	56	18	41	30	44
	56	68	27	27	24	7
KPNA3	609	4260	613	3196	2068	3313
	2672	3748	1377	946	1996	509
KPNA4	521	3975	569	3133	1658	2699
	3178	3400	1290	918	1754	543
KPNA5	87	374	72	314	162	328
	259	325	174	100	169	59
KPNA6	309	1397	280	987	607	1115
	1102	1407	583	385	782	244
KPNA7	15	0	8	0	3	0
	0	0	9	0	2	1
KPNB1	278	1550	295	1104	686	1045
	1074	1436	590	520	747	273
KPRP	12	1	7	0	6	0
	0	0	6	0	2	0
KPTN	15	5	7	4	5	6
	4	16	12	3	7	1
KRAS	58	229	57	189	136	127
	225	212	130	53	133	50
KRBA1	23	15	12	26	18	16
	20	15	12	8	20	8
KRBA2	30	126	30	52	47	74
	68	121	51	50	75	16
KRBOX1	5	11	4	8	4	8
	10	10	5	2	7	2
KRCC1	56	241	49	198	104	234
	197	260	130	84	137	44
KREMEN1	111	301	118	205	181	206
	192	388	163	88	208	83
KREMEN2	0	0	1	0	0	0
	0	1	0	0	0	0
KRI1	27	87	34	50	51	46
	84	93	31	31	62	17
KRIT1	140	617	137	450	352	473
	490	706	314	228	419	125
KRR1	193	924	195	622	448	647
	750	960	455	314	668	167
KRT1	13	3	11	2	10	0
	2	0	10	0	3	0
KRT10	19	21	20	12	17	3
	18	21	19	3	22	5
KRT12	16	0	8	0	1	0
	2	0	4	0	4	0

KRT13	11	3	8	2	2	2
	0	1	2	0	4	0
KRT14	6	2	5	1	7	0
	1	0	3	0	3	1
KRT15	10	0	8	0	3	0
	0	0	2	0	2	0
KRT16	10	0	3	2	3	0
	0	0	5	0	3	0
KRT17	15	7	6	1	4	1
	0	0	6	1	4	0
KRT18	18	4	6	4	2	1
	1	3	8	0	7	2
KRT19	11	5	7	11	5	3
	3	2	5	1	4	6
KRT2	15	0	12	0	3	0
	0	0	7	0	9	0
KRT20	18	1	14	0	5	0
	0	2	12	0	1	1
KRT222	11	0	5	2	4	2
	3	6	11	4	0	3
KRT23	14	1	6	0	2	1
	0	0	3	0	3	1
KRT24	20	0	7	0	10	0
	0	0	11	0	6	0
KRT25	8	1	9	2	1	1
	0	2	11	0	2	0
KRT26	12	0	7	0	2	0
	0	0	11	0	4	0
KRT27	5	0	4	0	0	0
	0	0	1	0	1	0
KRT28	7	0	5	0	3	0
	0	0	4	0	5	0
KRT3	26	0	9	0	6	0
	0	0	6	0	2	0
KRT31	10	12	11	13	5	15
	12	1	11	7	11	1
KRT32	15	2	4	0	2	0
	0	0	2	0	1	0
KRT33A	8	0	3	0	1	0
	0	0	1	0	1	0
KRT33B	8	0	4	0	2	0
	0	0	0	0	1	0
KRT34	8	0	6	0	0	0
	0	0	0	0	4	0
KRT35	13	0	4	0	2	0
	0	0	7	0	1	0
KRT36	6	0	0	0	0	0
	0	0	5	0	1	0
KRT37	10	0	7	0	2	0
	0	0	1	0	4	0
KRT38	10	0	6	0	1	0
	0	0	5	0	3	0
KRT39	12	0	8	0	4	0
	0	0	4	0	2	0
KRT4	12	0	9	0	4	0
	0	0	8	0	2	0
KRT40	16	0	14	0	1	1
	0	0	2	0	8	1

KRT5	20	0	9	0	4	0
	0	0	6	0	4	0
KRT6A	14	0	7	0	2	0
	0	0	2	0	1	0
KRT6B	12	0	4	0	1	0
	0	0	4	0	2	0
KRT6C	15	1	4	0	2	0
	0	0	7	0	3	0
KRT7	7	2	3	3	2	1
	0	1	7	1	0	4
KRT71	11	0	11	0	4	0
	0	0	5	0	5	0
KRT72	18	0	4	0	1	0
	1	1	12	1	5	0
KRT73	16	0	11	1	1	0
	2	0	7	0	5	0
KRT74	11	0	6	0	1	2
	0	0	8	0	3	0
KRT75	19	0	7	0	2	0
	0	0	7	0	1	0
KRT76	14	1	7	0	0	0
	0	0	9	0	6	0
KRT77	14	0	13	0	2	0
	0	0	5	0	4	0
KRT78	6	0	6	0	2	0
	0	0	0	0	3	0
KRT79	4	0	2	0	0	0
	0	0	1	0	3	0
KRT8	9	3	13	3	1	4
	1	1	5	1	2	0
KRT80	26	1	17	0	2	1
	0	0	10	0	6	0
KRT81	8	0	2	0	2	0
	0	0	5	0	3	0
KRT82	17	0	10	0	2	0
	0	0	0	0	2	0
KRT83	13	0	2	0	0	0
	0	1	5	0	3	0
KRT84	12	0	7	0	6	0
	0	0	4	0	5	0
KRT85	12	0	7	0	3	0
	0	0	5	0	2	0
KRT86	9	0	8	0	3	3
	0	2	5	0	0	0
KRT9	19	0	14	0	4	0
	0	0	13	0	2	0
KRTAP1-1	4	0	1	0	0	0
	0	0	1	0	0	0
KRTAP1-3	0	0	0	0	1	0
	0	0	5	0	4	0
KRTAP1-4	2	0	0	0	0	0
	0	0	0	0	0	0
KRTAP1-5	4	0	3	0	0	0
	0	0	0	0	1	0
KRTAP10-10	4	0	4	0	1	0
	0	0	1	0	0	0
KRTAP10-11	3	0	1	0	0	0
	0	0	0	0	0	0

KRTAP10-4	1	0	1	0	1	0
	0	0	0	0	0	0
KRTAP10-7	3	0	1	0	0	0
	0	0	3	0	0	0
KRTAP10-8	0	0	2	0	3	0
	0	0	0	0	0	0
KRTAP10-9	1	0	4	0	0	0
	0	0	2	0	0	0
KRTAP11-1	3	0	1	0	0	0
	0	0	3	0	0	0
KRTAP13-1	5	0	4	0	0	0
	0	0	1	0	2	0
KRTAP13-2	4	0	2	0	1	0
	0	0	0	0	2	0
KRTAP13-3	1	0	1	0	0	0
	0	0	2	0	1	0
KRTAP13-4	1	0	2	0	0	0
	0	0	0	0	0	0
KRTAP15-1	3	0	0	0	0	0
	0	0	0	0	0	0
KRTAP16-1	5	0	4	0	0	0
	0	0	4	0	1	0
KRTAP17-1	1	0	1	0	0	0
	0	0	0	0	1	0
KRTAP19-1	1	0	1	0	0	0
	0	0	1	0	0	0
KRTAP19-3	2	0	2	0	0	0
	0	7	1	0	3	0
KRTAP19-5	1	0	0	0	0	0
	0	0	0	0	0	0
KRTAP19-6	3	0	2	0	0	0
	0	0	0	0	1	0
KRTAP19-8	2	0	0	0	0	0
	0	0	1	0	0	0
KRTAP2-1	1	0	2	0	0	0
	0	0	1	0	0	0
KRTAP2-2	4	0	0	0	0	0
	0	0	0	0	0	0
KRTAP2-3	0	0	1	0	0	0
	0	0	1	0	2	0
KRTAP2-4	2	0	0	0	0	0
	0	0	1	0	0	0
KRTAP20-1	1	0	0	0	0	0
	0	0	1	0	0	0
KRTAP20-3	0	0	0	0	2	0
	0	0	0	0	1	0
KRTAP21-1	3	0	2	0	0	0
	0	0	0	0	0	0
KRTAP21-2	1	0	2	0	1	0
	0	0	1	0	0	0
KRTAP21-3	1	0	0	0	1	0
	0	0	1	0	0	0
KRTAP22-2	1	0	0	0	0	0
	0	0	0	0	0	0
KRTAP24-1	8	0	2	0	1	0
	0	0	1	0	0	0
KRTAP25-1	1	0	1	0	0	0
	0	0	1	0	0	0

KRTAP26-1	4	0	4	0	0	0
	0	0	4	0	0	0
KRTAP27-1	2	0	2	0	1	0
	0	0	2	0	1	0
KRTAP3-1	2	0	1	0	0	0
	0	0	1	0	0	0
KRTAP3-2	4	0	4	0	1	0
	0	0	2	0	0	0
KRTAP3-3	4	0	3	0	0	0
	0	0	0	0	1	0
KRTAP4-1	3	0	0	0	0	0
	0	0	1	0	0	0
KRTAP4-11	4	0	2	0	0	0
	0	0	2	0	1	0
KRTAP4-12	6	0	4	0	1	0
	0	0	1	0	0	0
KRTAP4-2	0	0	0	0	0	0
	0	0	1	0	0	0
KRTAP4-3	1	1	1	0	1	0
	0	0	0	0	0	0
KRTAP4-4	0	0	0	0	0	0
	0	0	0	0	2	0
KRTAP4-5	1	0	2	0	1	0
	0	0	1	0	0	0
KRTAP4-6	2	0	0	0	3	0
	0	0	0	0	0	0
KRTAP4-7	6	0	1	0	0	0
	0	0	4	0	1	0
KRTAP4-8	1	0	0	0	0	0
	0	0	1	0	2	0
KRTAP4-9	5	0	1	0	0	0
	0	0	1	0	0	0
KRTAP5-1	2	0	3	0	1	0
	0	1	0	0	0	0
KRTAP5-10	2	1	3	0	1	0
	0	0	2	0	2	0
KRTAP5-11	3	0	1	0	0	0
	0	0	0	0	0	0
KRTAP5-2	1	0	5	0	0	0
	0	0	1	0	0	0
KRTAP5-3	0	0	0	0	0	0
	0	0	2	0	0	0
KRTAP5-4	3	0	0	0	2	0
	0	0	1	0	1	0
KRTAP5-5	4	0	0	0	0	0
	0	0	2	0	0	0
KRTAP5-6	0	0	0	0	1	0
	0	0	0	0	0	0
KRTAP5-7	3	0	3	0	1	0
	1	1	2	1	2	0
KRTAP5-8	4	0	2	0	2	2
	0	0	2	0	0	0
KRTAP5-9	2	1	1	0	3	0
	1	1	1	0	1	0
KRTAP6-3	3	0	1	0	0	0
	0	0	2	0	1	0
KRTAP7-1	3	0	5	0	3	0
	0	0	2	0	0	0

KRTAP8-1	2	0	0	0	1	0
	0	0	1	0	0	0
KRTAP9-1	1	0	0	0	0	0
	0	0	0	0	0	0
KRTAP9-2	4	0	0	0	1	0
	0	0	1	0	1	0
KRTAP9-3	9	0	1	0	0	0
	0	0	0	0	0	0
KRTAP9-4	9	0	1	0	0	0
	0	0	0	0	0	0
KRTAP9-7	0	0	0	0	0	0
	0	0	1	0	0	0
KRTAP9-8	1	0	2	0	0	0
	0	0	3	0	1	0
KRTAP9-9	7	0	2	0	1	0
	0	0	0	0	1	0
KRTCAP2	20	116	16	78	51	80
	134	141	47	29	55	18
KRTCAP3	10	5	8	8	1	2
	4	3	8	2	7	1
KRTDAP	3	0	6	0	3	0
	0	0	4	0	0	0
KSR1	70	214	61	135	109	160
	211	131	97	68	108	41
KSR2	96	3	56	0	13	0
	1	0	36	0	24	0
KTI12	0	0	1	0	0	0
	0	0	0	0	0	0
KTN1	1107	8495	1185	4693	4144	5278
	5691	6602	2490	2760	3887	1298
KXD1	26	134	28	82	61	127
	128	167	81	33	88	28
KY	110	464	158	571	161	386
	116	394	139	57	132	60
KYNU	26	5	15	4	11	6
	3	9	19	2	13	0
L1CAM	34	3	7	4	11	2
	3	5	8	2	7	3
L1TD1	41	1	18	10	7	6
	2	2	25	0	11	0
L2HGDH	110	448	119	337	179	252
	356	311	178	90	249	55
L3HYPDH	9	57	15	56	25	31
	39	61	19	17	35	10
L3MBTL1	31	55	31	44	28	54
	33	51	34	15	39	20
L3MBTL2	39	139	24	73	62	110
	95	147	90	35	85	24
L3MBTL3	37	69	50	48	41	42
	26	43	61	16	40	6
L3MBTL4	39	28	33	42	29	15
	37	27	28	13	38	9
LACC1	42	53	43	35	39	37
	24	45	39	10	21	10
LACE1	34	80	24	49	32	59
	57	89	35	29	52	10
LACRT	9	0	3	0	5	0
	0	0	5	0	2	0

LACTB	78	389	49	307	148	361
	529	443	138	115	295	61
LACTB2	54	171	50	141	78	223
	256	187	94	45	195	38
LACTBL1	10	0	3	0	1	0
	0	0	0	0	3	0
LAD1	18	21	17	0	10	2
	15	21	8	8	10	6
LAG3	10	0	3	3	1	0
	0	1	5	0	2	0
LAGE3	11	9	4	9	6	9
	13	17	6	1	4	0
LAIR1	22	15	19	12	21	21
	9	24	19	8	10	8
LAIR2	2	0	2	1	1	0
	0	2	3	0	3	0
LALBA	5	0	1	0	1	0
	0	0	1	0	0	0
LAMA1	96	0	60	5	17	0
	0	1	37	1	30	1
LAMA2	504	1763	675	1975	918	1710
	1269	2413	1144	657	1231	486
LAMA3	128	179	99	80	83	66
	137	108	112	45	103	27
LAMA4	200	806	162	567	445	378
	456	510	289	225	300	224
LAMA5	27	113	26	44	25	29
	71	81	45	28	46	21
LAMB1	136	561	136	468	170	334
	374	805	379	166	319	97
LAMB2	240	1293	274	1378	503	1096
	795	836	488	295	477	202
LAMB3	46	122	58	238	33	145
	36	41	39	3	25	8
LAMB4	49	6	20	2	17	2
	0	2	35	1	16	1
LAMC1	468	1985	629	1615	1153	1724
	1433	2627	1105	708	1247	556
LAMC2	57	8	33	10	19	6
	10	35	31	8	15	7
LAMC3	22	0	7	3	3	1
	2	5	13	4	4	0
LAMP1	82	316	87	217	230	257
	292	523	195	127	226	111
LAMP2	624	2855	595	2302	1722	2298
	2539	3132	1290	965	1878	524
LAMP3	16	2	7	1	2	0
	0	1	11	3	3	0
LAMP5	16	1	9	1	1	0
	0	0	8	0	3	0
LAMTOR1	44	280	32	153	112	166
	240	318	101	68	122	46
LAMTOR2	21	65	17	62	36	72
	133	121	46	25	55	12
LAMTOR3	110	404	85	275	231	269
	342	563	224	121	290	77
LAMTOR4	32	160	34	106	70	108
	168	260	101	36	88	28

LAMTOR5	41	260	25	159	130	176
	306	305	126	76	167	22
LANCL1	151	790	172	621	355	654
	610	645	386	153	414	124
LANCL2	51	117	31	117	75	137
	150	163	86	44	105	29
LANCL3	17	12	11	1	5	2
	1	1	5	1	6	1
LAP3	78	533	105	337	236	378
	454	650	322	194	402	100
LAPTM4A	106	486	65	550	305	449
	420	739	254	145	335	88
LAPTM4B	199	947	171	873	381	954
	818	1066	706	218	768	204
LAPTM5	13	40	9	22	9	23
	22	33	12	13	15	9
LARGE	112	383	106	274	204	348
	323	407	167	141	219	67
LARP1	187	1148	181	796	492	700
	809	875	262	277	412	144
LARP1B	111	438	86	283	187	321
	328	488	189	143	240	77
LARP4	287	1560	287	1088	641	967
	963	1143	467	311	593	210
LARP4B	216	1106	224	675	442	671
	779	1062	472	321	615	199
LARP6	28	69	19	57	31	41
	29	102	35	26	32	21
LARP7	111	611	123	328	302	374
	399	512	170	210	258	77
LARS	370	1765	391	1272	858	1309
	1387	1792	846	550	934	311
LARS2	74	244	66	151	95	187
	185	218	100	55	144	32
LAS1L	42	146	47	95	54	118
	112	201	63	64	81	29
LASP1	60	193	61	169	98	165
	154	173	94	59	109	45
LAT	7	3	7	4	2	4
	6	6	3	2	6	2
LAT2	7	11	6	6	5	6
	3	13	11	2	9	3
LATS1	107	508	152	346	227	327
	380	502	182	180	261	95
LATS2	80	245	39	191	167	188
	299	200	122	106	133	66
LAX1	14	0	13	2	3	4
	4	4	12	0	4	3
LAYN	41	37	20	5	17	23
	16	40	15	5	15	2
LBH	29	86	20	64	25	41
	31	60	35	19	37	10
LBP	20	3	12	5	3	1
	6	38	27	9	5	4
LBR	56	192	41	145	126	150
	170	267	101	62	112	34
LBX1	9	56	11	26	27	24
	37	26	20	12	19	1

LBX2	4	1	4	0	2	1
	1	0	1	0	1	0
LCA5	47	70	37	61	42	58
	53	92	58	27	54	16
LCA5L	32	49	17	30	32	20
	41	53	34	24	33	9
LCAT	4	18	2	10	9	10
	9	6	9	3	9	4
LCE1A	1	0	1	0	1	0
	0	0	0	0	0	0
LCE1B	4	0	2	0	1	0
	0	0	0	0	0	0
LCE1C	1	0	1	0	1	0
	0	0	0	0	0	0
LCE1E	4	0	11	0	2	0
	0	0	5	0	4	0
LCE1F	1	0	2	0	0	0
	0	0	0	0	0	0
LCE2A	1	0	2	0	0	0
	0	0	0	0	0	0
LCE2B	3	0	1	0	0	0
	0	0	1	0	0	0
LCE2C	2	0	2	0	0	0
	0	0	0	0	3	0
LCE2D	5	0	4	0	0	0
	0	0	0	0	1	0
LCE3C	1	0	1	0	0	0
	0	0	0	0	0	0
LCE3D	2	0	1	0	0	0
	0	0	1	0	0	0
LCE3E	1	0	1	0	0	0
	0	0	2	0	0	0
LCE5A	0	0	0	0	0	0
	0	0	2	0	1	0
LCE6A	5	0	5	0	0	0
	0	0	0	0	0	0
LCK	12	5	8	1	6	3
	2	4	11	2	4	0
LCLAT1	51	151	46	133	64	93
	106	138	71	30	80	37
LCMT1	39	202	50	121	62	105
	164	152	98	41	102	29
LCMT2	11	35	17	28	33	30
	34	52	16	11	27	8
LCN1	3	0	1	0	0	0
	0	0	1	0	0	0
LCN10	4	1	2	0	2	2
	4	0	6	0	3	0
LCN12	2	0	6	0	0	1
	0	1	0	1	1	0
LCN15	0	0	1	0	1	0
	0	0	1	0	0	0
LCN2	8	0	6	0	2	0
	0	0	2	0	1	0
LCN6	1	2	8	0	2	2
	2	2	3	1	1	0
LCN8	3	0	3	0	0	0
	7	1	4	0	2	0

LCN9	7	0	3	0	1	0
	0	0	1	0	2	0
LCNL1	2	0	2	0	0	1
	0	0	0	0	0	1
LCOR	254	1476	358	1202	634	957
	743	1146	521	317	584	180
LCORL	90	266	93	258	146	244
	194	279	152	92	186	54
LCP1	51	121	29	71	46	95
	56	158	75	36	85	20
LCP2	31	50	34	22	14	42
	33	46	34	22	40	7
LCT	39	4	11	2	7	3
	1	1	15	1	11	1
LCTL	6	0	5	0	3	1
	0	0	3	0	8	1
LDB1	75	195	48	151	115	222
	171	293	129	96	155	71
LDB2	31	305	43	125	91	89
	185	212	97	60	102	43
LDB3	2140	21483	2764	13420	7395	11459
	15856	14896	5437	3602	6899	2665
LDHA	1476	14983	2313	20612	5374	7870
	9004	4318	5526	3076	2958	489
LDHAL6A	18	2	6	3	2	3
	1	1	14	1	11	0
LDHAL6B	13	3	4	0	2	1
	0	0	3	0	1	0
LDHB	186	991	119	255	496	246
	772	2662	283	377	575	344
LDHC	6	2	9	0	9	0
	16	0	6	1	2	0
LDHD	35	186	21	79	58	49
	152	307	107	53	147	34
LDLR	29	68	23	50	15	65
	55	29	36	18	15	5
LDLRAD1	7	0	2	0	2	0
	0	0	0	0	0	0
LDLRAD2	20	9	13	8	6	8
	2	13	6	1	3	1
LDLRAD3	20	21	12	18	11	30
	24	9	21	6	15	4
LDLRAD4	161	320	76	444	260	366
	325	265	204	125	246	76
LDLRAP1	26	125	18	43	49	81
	92	90	42	26	59	17
LDOC1	8	4	4	1	2	7
	6	3	0	0	0	2
LDOC1L	53	185	56	191	64	160
	132	206	73	53	118	30
LEAP2	7	17	3	7	6	19
	12	18	5	5	5	1
LECT1	11	1	5	0	0	2
	0	2	6	0	3	0
LECT2	8	0	8	0	5	0
	0	0	3	0	3	0
LEF1	25	13	17	8	12	1
	2	2	13	0	6	2

LEFTY1	2	1	4	2	2	0
	0	0	3	0	1	0
LEFTY2	5	1	1	0	2	1
	3	4	0	1	1	1
LEKR1	26	2	26	6	12	4
	2	9	29	2	14	3
LELP1	2	0	1	0	0	0
	0	0	1	0	0	0
LEMD1	8	0	2	0	2	2
	0	0	7	0	0	0
LEMD2	40	81	22	83	60	61
	46	131	52	30	67	18
LEMD3	73	287	70	221	112	179
	205	356	160	104	169	43
LENEP	1	0	0	0	1	0
	0	2	0	0	0	0
LENG1	15	37	14	11	21	37
	32	54	30	18	28	6
LENG8	96	410	74	207	165	218
	274	409	112	154	177	82
LENG9	5	8	1	2	4	4
	1	6	3	1	2	0
LEO1	45	195	51	101	95	173
	136	155	80	43	107	25
LEP	20	30	11	6	35	11
	13	12	8	0	9	28
LEPR	65	120	75	170	108	87
	70	177	85	29	83	72
LEPRE1	22	44	20	36	34	32
	26	61	33	10	36	7
LEPREL1	27	16	17	16	20	13
	10	17	28	3	18	3
LEPREL2	14	10	13	11	6	11
	5	8	10	4	7	3
LEPREL4	15	16	5	14	19	24
	12	7	8	5	10	2
LEPROT	14	132	8	95	39	130
	186	159	52	29	79	23
LEPROTL1	48	147	30	109	56	80
	106	133	62	31	69	21
LETM1	68	239	43	143	92	179
	200	294	105	66	165	43
LETM2	12	2	8	2	3	5
	0	4	10	0	5	1
LETMD1	57	243	63	150	78	135
	193	224	123	74	153	44
LEUTX	3	0	5	0	0	0
	0	0	3	0	1	0
LFNG	15	23	10	9	7	8
	12	15	9	4	5	4
LGALS1	252	593	133	530	341	499
	865	1323	231	491	418	258
LGALS12	11	2	8	3	6	0
	0	5	2	0	4	1
LGALS13	3	0	1	0	1	0
	0	0	2	0	2	0
LGALS14	20	0	3	0	0	1
	0	0	3	0	2	0

LGALS16	7	0	4	0	2	0
	0	0	2	0	0	0
LGALS2	8	1	2	2	0	0
	2	0	4	1	1	0
LGALS3	30	176	35	145	73	80
	178	265	113	48	92	50
LGALS3BP	21	92	21	80	47	91
	76	100	71	46	55	20
LGALS4	5	9	3	3	1	4
	1	2	6	2	4	2
LGALS7	1	0	3	0	1	0
	0	0	2	0	0	0
LGALS7B	3	0	2	0	0	0
	0	0	0	0	3	0
LGALS8	90	217	71	165	98	201
	187	259	148	100	135	48
LGALS9	13	9	2	7	10	5
	8	10	8	9	10	0
LGALS9B	5	0	4	0	2	0
	0	0	0	0	0	0
LGALS9C	5	0	5	0	0	0
	0	0	7	0	1	0
LGALSL	120	479	94	335	421	667
	855	726	369	147	561	75
LGI1	46	95	32	50	37	65
	53	174	25	13	39	30
LGI2	24	13	19	6	23	9
	0	25	19	2	9	6
LGI3	10	0	4	0	1	0
	0	0	8	0	1	0
LGI4	32	197	41	62	67	92
	148	345	97	59	105	23
LGMN	59	173	60	149	103	151
	121	276	102	45	108	31
LGR4	137	485	139	415	366	456
	363	477	221	145	288	84
LGR5	69	77	39	67	96	30
	268	1260	58	187	461	177
LGR6	19	0	15	0	4	0
	0	0	14	1	6	0
LGSN	29	4	12	0	4	3
	4	0	13	1	8	0
LHCGR	34	0	9	4	6	1
	0	1	13	0	4	1
LHFP	45	201	36	154	122	122
	175	299	109	73	134	53
LHFPL1	15	4	7	5	7	1
	1	4	3	3	7	0
LHFPL2	43	107	39	115	61	108
	55	118	67	11	47	25
LHFPL3	13	0	9	0	6	0
	0	0	7	0	8	0
LHFPL4	15	0	12	0	7	0
	0	1	6	0	7	0
LHFPL5	18	1	4	1	4	2
	1	1	9	0	3	0
LHPP	11	9	7	15	15	6
	21	11	12	5	9	2

LHX1	9	0	5	0	0	0
	0	0	2	0	1	0
LHX2	5	0	3	0	0	0
	0	0	1	0	2	0
LHX3	7	0	5	0	1	0
	0	0	1	0	4	0
LHX4	9	1	5	0	0	1
	3	0	2	0	6	0
LHX5	4	0	2	0	0	0
	0	0	0	0	1	0
LHX6	19	19	13	16	8	13
	21	18	14	5	14	8
LHX8	25	0	7	0	6	1
	0	0	15	0	7	0
LHX9	16	0	8	0	2	0
	0	0	4	0	5	0
LIAS	57	208	34	121	83	124
	201	241	139	96	132	36
LIF	12	1	17	3	5	3
	0	0	5	1	2	1
LIFR	472	2042	442	1811	1298	1701
	1974	3729	1261	825	1592	472
LIG1	34	33	33	22	14	20
	25	29	21	19	37	8
LIG3	76	211	46	129	109	117
	176	214	119	78	122	42
LIG4	76	238	69	238	143	220
	138	276	125	64	141	37
LILRA1	31	4	11	1	5	2
	1	1	10	0	5	1
LILRA2	11	0	4	1	3	1
	2	1	3	0	1	0
LILRA3	6	0	3	0	1	0
	0	0	1	0	0	0
LILRA4	4	0	4	0	0	0
	0	0	2	0	0	0
LILRA5	11	4	2	0	1	1
	0	1	5	0	3	0
LILRA6	25	6	23	10	4	7
	0	17	7	3	15	1
LILRB1	18	1	15	0	2	8
	6	6	7	1	9	0
LILRB2	22	13	9	6	3	8
	5	8	7	4	4	2
LILRB3	14	8	7	4	5	3
	4	11	12	1	6	1
LILRB4	7	1	5	6	5	8
	1	4	3	2	7	0
LILRB5	17	12	18	17	10	16
	14	22	10	3	16	2
LIM2	1	0	7	0	1	0
	0	0	0	0	0	0
LIMA1	126	671	175	464	319	458
	381	481	239	154	216	143
LIMCH1	993	7093	1917	5498	2786	4670
	3608	4337	2263	1653	2918	873
LIMD1	126	234	67	226	174	263
	231	531	165	86	164	94

LIMD2	17	11	7	5	2	4
	7	12	10	4	7	2
LIME1	2	11	10	2	2	5
	1	6	3	2	1	1
LIMK1	34	41	17	42	38	57
	20	21	22	6	19	16
LIMK2	39	79	47	82	42	65
	69	75	59	29	51	21
LIMS1	198	731	173	633	414	461
	677	800	382	300	476	153
LIMS2	15	60	13	36	19	25
	56	62	31	31	44	17
LIMS3	1	1	0	7	1	0
	2	5	2	0	5	0
LIMS3L	8	5	3	6	1	2
	2	4	4	1	1	1
LIN28A	71	4	31	3	17	4
	5	11	25	1	18	1
LIN28B	30	0	19	0	9	0
	0	0	22	0	6	0
LIN37	8	23	4	15	12	9
	17	28	14	2	15	6
LIN52	22	59	14	26	21	27
	23	93	38	27	44	15
LIN54	67	178	50	187	124	159
	176	229	105	76	144	40
LIN7A	12	15	4	4	9	5
	5	9	9	3	4	2
LIN7B	16	70	9	68	18	19
	115	76	33	12	32	9
LIN7C	140	540	131	464	332	404
	559	730	305	187	405	118
LIN9	36	55	30	52	56	37
	51	78	57	24	60	13
LINGO1	5	1	7	0	0	0
	3	0	3	0	5	1
LINGO2	18	1	10	0	8	0
	0	0	13	1	11	1
LINGO3	2	0	1	1	0	0
	0	0	0	0	0	0
LINGO4	16	23	10	26	11	29
	63	12	37	11	13	8
LINS	18	73	24	36	33	48
	48	116	45	15	66	12
LIPA	39	125	22	105	55	80
	68	161	95	32	82	28
LIPC	23	1	6	3	3	0
	0	0	9	0	6	0
LIPE	16	36	7	10	22	14
	22	26	15	10	14	23
LIPF	14	8	6	0	3	1
	0	0	6	0	4	0
LIPG	36	7	10	3	4	4
	0	0	11	0	5	0
LIPH	18	1	6	2	1	2
	1	1	17	0	2	0
LIPI	16	0	13	0	3	0
	0	1	11	0	7	0

LIPJ	9	18	17	9	8	6
	2	1	8	2	6	1
LIPK	13	0	8	0	3	0
	0	0	6	0	4	0
LIPM	16	1	7	0	4	0
	0	0	8	0	6	0
LIPN	13	1	12	0	4	0
	0	0	7	0	3	0
LIPT1	23	93	23	50	55	59
	92	111	49	27	67	17
LIPT2	8	41	16	47	25	35
	81	50	26	20	39	6
LITAF	50	142	27	127	46	109
	88	140	55	55	82	46
LIX1	20	0	24	1	5	0
	0	2	13	0	4	0
LIX1L	24	81	35	70	47	66
	168	103	66	27	94	24
LLGL1	19	62	14	42	30	39
	39	48	29	12	45	14
LLGL2	14	7	8	4	3	0
	3	3	10	2	10	0
LLPH	39	178	23	87	92	112
	157	162	79	33	86	26
LMAN1	285	1457	299	935	645	1042
	1018	1471	642	372	795	216
LMAN1L	9	0	6	0	1	0
	0	0	4	0	4	0
LMAN2	40	213	35	172	108	174
	204	257	119	81	140	45
LMAN2L	55	226	42	117	112	203
	113	261	121	37	91	31
LMBR1	90	542	128	421	165	280
	426	475	319	124	282	81
LMBR1L	27	80	33	63	27	50
	65	114	45	27	65	21
LMBRD1	133	433	93	369	231	289
	376	533	206	172	259	86
LMBRD2	90	350	100	268	202	253
	226	391	174	104	187	49
LMCD1	457	2024	335	1390	1128	1408
	2228	3194	1424	461	1696	362
LMF1	12	19	5	20	11	18
	8	13	11	6	15	1
LMF2	6	35	12	37	21	27
	20	37	19	10	20	6
LMLN	73	92	42	64	51	53
	49	99	52	21	58	15
LMNA	172	917	238	683	418	713
	517	932	385	220	469	155
LMNB1	17	25	21	5	21	16
	13	12	27	5	7	1
LMNB2	39	164	41	97	50	114
	91	188	67	48	78	25
LMO1	16	40	10	31	21	55
	90	68	30	23	33	7
LMO2	72	286	69	430	84	340
	300	239	110	65	161	41

LMO3	37	6	24	4	24	3	4	27	19	0	21	9
LMO4	88	250	79	305	180	306	204	302	152	109	151	48
LMO7	649	3619	1549	2243	1942	3160	3167	3686	1842	1332	1875	623
LMOD1	141	587	177	538	392	869	388	371	302	180	304	162
LMOD2	2322	7295	1662	3085	4260	5264	8806	15537	4792	4114	8665	2952
LMOD3	1254	7014	1159	3666	3418	5675	6208	6303	2892	2603	3990	1394
LMTK2	110	335	136	272	213	299	269	524	269	141	421	122
LMTK3	13	0	7	0	1	0	0	1	9	0	6	0
LMX1A	17	5	8	11	9	8	3	4	9	3	6	3
LMX1B	21	3	18	3	3	1	0	5	12	0	3	0
LNP1	28	50	20	43	29	33	28	54	27	13	30	15
LNPEP	338	1625	360	1163	680	1403	1183	1567	838	420	822	294
LNX1	28	77	18	44	42	35	55	70	58	16	47	17
LNX2	62	178	40	126	117	133	150	206	78	54	112	36
LOC100127946	1	0	3	0	0	2	0	0	0	0	0	0
LOC100127983	16	14	9	11	10	9	12	15	16	2	10	5
LOC100128071	0	3	1	8	1	7	4	3	4	3	0	0
LOC100128326	1	0	2	0	1	0	3	0	0	3	2	0
LOC100128905	6	0	0	1	1	0	0	0	0	0	0	0
LOC100129083	8	0	8	1	1	0	0	0	3	0	1	0
LOC100129098	5	1	1	0	0	0	0	0	1	0	1	0
LOC100129216	1	1	2	1	0	0	0	0	0	0	0	0
LOC100129307	2	0	2	0	0	0	0	0	0	0	0	0
LOC100129480	11	3	1	0	1	2	5	5	9	0	3	1

LOC100129520	19	0	2	0	1	0	0	0	2	0	0	0
LOC100129636	13	2	8	4	11	4	11	3	10	1	12	1
LOC100129924	6	0	4	0	2	0	0	3	7	0	1	2
LOC100130097	27	4	10	1	1	4	2	5	12	0	2	1
LOC100130301	7	1	3	2	1	2	0	0	2	0	3	0
LOC100130348	3	0	1	0	0	0	0	0	3	0	1	0
LOC100130357	15	32	18	16	11	19	9	21	26	5	15	5
LOC100130370	1	0	0	0	0	0	0	0	2	0	1	0
LOC100130451	57	3	14	3	8	1	1	6	16	0	7	1
LOC100130480	5	0	6	0	2	0	0	0	4	0	1	0
LOC100130539	5	0	9	1	0	1	0	4	3	1	4	1
LOC100130705	7	3	6	3	3	12	2	11	8	3	7	1
LOC100130880	5	0	2	1	6	1	0	0	3	0	1	0
LOC100130921	4	0	0	0	3	0	0	0	2	0	1	0
LOC100131094	3	1	7	1	0	0	1	1	2	1	5	0
LOC100131107	11	1	0	0	0	0	0	0	3	0	1	0
LOC100131381	1	0	2	0	1	0	0	0	0	0	0	0
LOC100131608	39	3	16	2	13	1	1	5	23	0	9	0
LOC100132146	15	0	4	0	1	0	1	0	6	0	2	0

LOC100132154	4	0	9	1	0	0	1	0	6	1	6	0
LOC100132174	2	0	0	0	0	0	0	0	0	0	0	0
LOC100132202	10	0	4	0	2	0	0	0	4	0	3	0
LOC100132247	45	72	16	75	44	48	95	65	33	42	38	37
LOC100132339	5	0	6	0	0	0	0	0	3	0	1	0
LOC100132581	35	3	16	0	20	0	0	3	18	0	9	1
LOC100132705	5	0	7	0	2	0	0	0	4	0	0	0
LOC100132731	10	1	5	0	4	2	0	1	8	0	4	0
LOC100132858	0	0	4	0	2	0	1	0	1	0	2	0
LOC100132859	2	0	1	0	0	0	0	0	0	0	0	0
LOC100132900	11	0	4	1	2	0	0	0	13	0	2	0
LOC100133251	0	0	2	0	1	0	0	0	1	0	1	0
LOC100133267	3	0	0	0	1	0	0	0	2	0	0	0
LOC100133301	2	0	2	0	1	0	0	0	0	0	0	0
LOC100144595	9	0	4	0	2	0	1	0	7	0	1	0
LOC100286914	10	22	8	8	14	6	2	15	8	2	6	2
LOC100287036	15	18	8	23	6	12	18	8	5	4	13	5
LOC100287177	2	0	0	0	0	0	0	0	0	0	0	0
LOC100287290	11	31	6	11	10	13	8	21	15	3	13	2

LOC100287294	1	0	1	0	0	0	0	0	1	0	0	0
LOC100287313	23	41	22	33	42	46	52	77	47	22	50	10
LOC100287368	54	1	11	0	6	2	1	0	8	1	5	0
LOC100287387	0	0	0	0	0	0	0	0	1	0	0	0
LOC100287399	16	0	14	0	12	0	0	1	20	0	14	0
LOC100287477	9	0	3	0	0	0	0	0	0	0	0	0
LOC100287482	7	0	3	0	0	1	2	1	1	0	3	0
LOC100287633	13	0	8	0	3	0	0	1	5	0	1	0
LOC100287651	18	0	9	0	3	0	0	0	8	0	1	1
LOC100287718	7	0	5	0	0	0	0	0	3	0	0	0
LOC100287837	0	0	1	3	0	0	0	4	2	1	2	0
LOC100287852	1	0	0	0	0	0	0	0	0	0	0	0
LOC100288332	127	411	137	231	151	304	364	557	153	114	212	112
LOC100288336	5	0	4	0	0	0	0	0	1	0	0	0
LOC100288524	1	0	0	0	0	0	0	0	0	0	0	0
LOC100288562	3	0	5	0	0	0	0	0	12	0	1	0
LOC100288568	14	0	7	0	4	0	0	0	6	0	0	0
LOC100288646	5	7	8	2	9	10	2	6	17	2	6	1
LOC100288807	27	0	8	0	6	2	2	5	8	1	3	0

LOC100288814	4	0	0	0	0	0	0	0	0	0	0	0
LOC100289151	12	0	5	0	1	0	0	0	7	0	1	0
LOC100289187	6	3	9	3	0	1	2	0	4	0	1	0
LOC100289375	21	1	4	0	4	0	0	2	8	0	2	0
LOC100289561	31	102	20	62	44	53	89	118	32	21	49	23
LOC100500938	2	2	3	0	3	0	0	2	2	0	1	1
LOC100505478	12	0	9	0	3	0	0	0	4	0	1	0
LOC100505549	15	9	6	10	9	6	5	13	6	2	12	3
LOC100505679	16	1	8	2	5	3	0	3	3	1	4	2
LOC100505767	15	7	6	1	4	6	6	7	13	3	13	1
LOC100505781	81	0	19	1	11	4	2	1	13	1	8	1
LOC100505836	12	8	9	13	5	4	3	5	8	2	4	0
LOC100505841	0	1	2	0	2	0	0	0	0	0	1	0
LOC100506127	7	15	6	17	6	10	13	20	13	1	10	3
LOC100506248	3	4	4	0	0	5	0	2	0	1	0	1
LOC100506310	17	32	9	16	20	15	31	18	29	20	7	1
LOC100506388	2	13	1	6	5	16	5	9	6	5	4	1
LOC100506422	33	2	17	0	9	0	1	0	15	0	8	0
LOC100506504	6	4	2	0	1	2	0	1	2	1	0	0

LOC100506533	18	1	10	0	0	0	0	0	5	2	3	0
LOC100506571	6	1	1	5	1	1	1	1	12	0	6	0
LOC100506667	1	0	1	0	2	0	0	0	0	0	2	0
LOC100506688	9	1	3	1	2	0	0	0	5	0	2	1
LOC100506859	6	0	4	0	1	1	0	0	3	0	1	0
LOC100506870	23	73	27	100	62	111	123	68	50	23	30	11
LOC100506888	0	3	9	0	2	0	0	1	9	0	0	0
LOC100506972	0	0	0	0	0	0	1	0	0	0	0	0
LOC100507003	8	0	5	0	4	0	0	0	4	0	1	0
LOC100507172	3	1	1	1	2	1	0	3	2	2	0	0
LOC100507203	14	0	10	0	2	0	0	2	7	0	8	0
LOC100507341	3	0	2	1	2	1	1	0	3	0	1	0
LOC100507369	3	0	2	0	0	0	1	0	0	1	1	0
LOC100507462	15	1	5	2	4	2	1	1	13	0	5	0
LOC100507607	11	3	5	5	6	5	1	3	20	2	7	1
LOC100507656	2	4	0	1	0	0	2	4	7	3	6	0
LOC100508736	30	1	11	0	10	0	1	0	11	0	6	0
LOC100509091	5	0	1	0	1	0	0	0	2	0	2	0
LOC100509247	16	0	1	0	1	0	0	0	6	0	1	0

LOC100509263	0	0	0	0	0	0	0	1	0	0	0	0
LOC100652732	7	3	2	2	0	1	3	1	2	0	3	0
LOC100652740	3	1	2	1	1	1	1	0	2	0	3	0
LOC100652741	8	40	13	22	27	35	39	42	19	14	23	14
LOC100652758	4	14	6	12	6	11	16	20	6	4	9	3
LOC100652766	67	493	117	309	255	365	367	496	199	138	278	96
LOC100652774	1	0	7	0	3	0	0	0	0	0	2	0
LOC100652777	28	6	11	2	1	4	1	1	10	3	8	1
LOC100652807	2	1	0	1	0	1	0	0	1	1	0	0
LOC100652824	39	1	27	0	2	0	0	0	21	0	6	0
LOC100652871	3	0	0	0	0	0	0	0	1	0	0	0
LOC100652894	7	2	1	0	1	0	1	1	1	0	1	0
LOC100652901	39	14	14	5	7	3	11	8	11	2	7	1
LOC100652943	29	5	8	3	5	1	6	5	3	2	5	1
LOC100652953	2	0	3	0	1	0	0	0	1	0	1	0
LOC100862671	13	49	12	34	25	31	20	35	14	10	10	8
LOC100996250	6	1	7	1	0	1	0	5	4	2	5	1
LOC100996253	10	5	5	6	9	5	5	8	7	0	11	1
LOC100996254	5	1	4	1	3	0	0	0	7	0	3	0

LOC100996256	5	3	3	3	4	0	3	2	10	0	2	0
LOC100996259	2	1	2	0	3	0	0	1	2	1	2	1
LOC100996261	0	0	0	0	0	0	0	0	0	0	1	0
LOC100996271	0	0	0	0	0	0	0	0	1	0	0	0
LOC100996276	10	0	3	0	1	0	0	1	1	0	3	0
LOC100996278	4	0	0	0	0	0	0	0	0	0	0	0
LOC100996279	16	0	3	0	3	0	0	0	6	0	1	0
LOC100996282	6	0	4	0	0	0	0	0	3	0	1	0
LOC100996287	10	0	5	0	1	1	0	0	3	0	1	0
LOC100996289	1	0	1	0	0	0	0	1	0	0	0	0
LOC100996290	35	6	17	1	6	1	7	6	12	3	9	2
LOC100996295	15	0	7	0	0	0	0	0	10	0	9	0
LOC100996301	3	1	3	4	0	0	0	8	0	1	2	1
LOC100996304	14	0	9	0	6	0	0	0	17	0	9	0
LOC100996312	3	0	0	0	0	0	0	0	0	0	0	0
LOC100996314	21	68	19	36	15	35	35	66	42	6	29	13
LOC100996317	0	0	0	0	0	0	0	1	0	0	1	0
LOC100996318	17	0	7	0	2	0	0	0	9	0	3	0
LOC100996325	13	0	5	0	1	0	0	1	6	0	3	0

LOC100996329	13	0	5	0	0	0	1	0	4	0	2	0
LOC100996331	12	0	13	0	5	0	0	0	12	0	15	0
LOC100996337	2	0	2	0	0	0	0	0	1	0	0	0
LOC100996346	1	0	0	0	0	0	0	0	3	0	1	0
LOC100996350	253	0	99	2	27	1	3	2	57	0	40	0
LOC100996356	325	1593	219	1080	503	1535	1029	1132	776	271	679	134
LOC100996368	2	0	0	1	0	0	0	0	0	1	0	0
LOC100996369	1	0	0	0	0	0	0	1	1	0	1	0
LOC100996372	0	0	2	0	0	0	0	0	0	0	0	0
LOC100996378	3	0	0	0	0	0	0	0	0	0	0	0
LOC100996380	1	0	0	0	0	0	0	0	0	0	0	0
LOC100996382	7	1	3	0	1	0	0	0	3	0	1	0
LOC100996392	6	0	11	0	1	0	0	0	4	0	0	0
LOC100996394	10	0	17	0	5	0	0	0	10	0	5	0
LOC100996401	1	0	0	0	0	0	0	0	0	0	1	0
LOC100996408	0	1	2	0	0	0	0	0	0	0	1	0
LOC100996413	5	0	5	0	1	0	0	0	5	0	3	0
LOC100996426	7	0	1	0	1	0	0	0	1	0	0	0
LOC100996428	2	0	1	0	0	0	0	0	2	0	1	0

LOC100996432	36	1	27	0	15	0	0	0	31	0	8	0
LOC100996433	8	9	5	0	0	7	2	0	7	0	1	0
LOC100996440	7	0	2	0	0	0	0	0	3	0	0	0
LOC100996445	9	0	3	0	0	0	0	0	0	0	2	0
LOC100996451	3	0	1	0	1	0	0	1	1	0	0	0
LOC100996459	5	0	2	0	1	0	2	1	3	2	2	0
LOC100996465	9	0	5	0	1	0	0	3	2	0	0	0
LOC100996467	328	1767	388	1139	613	1271	772	1920	559	355	772	252
LOC100996470	9	10	2	5	0	12	1	4	4	3	2	1
LOC100996473	4	1	0	0	0	1	1	0	0	2	3	1
LOC100996485	17	5	18	3	8	8	6	11	9	1	6	1
LOC100996487	0	0	0	0	0	1	0	0	1	0	1	0
LOC100996489	0	0	0	0	0	1	0	0	0	0	1	1
LOC100996492	3	0	1	0	1	0	0	0	1	0	0	0
LOC100996499	0	0	0	0	0	0	0	0	2	0	0	0
LOC100996504	6	3	4	1	2	0	2	2	6	0	1	0
LOC100996505	6	16	3	6	3	6	4	5	2	2	3	0
LOC100996515	11	17	12	8	8	9	1	11	9	2	8	4
LOC100996516	0	0	0	1	0	0	0	0	1	0	1	0

LOC100996518	6	1	2	0	4	4	0	1	7	0	4	0
LOC100996519	0	0	0	0	0	0	0	0	0	1	0	0
LOC100996525	5	0	0	0	0	0	0	0	1	0	2	0
LOC100996527	7	1	4	0	5	0	0	1	2	0	1	1
LOC100996528	3	4	3	0	2	1	0	2	2	0	1	1
LOC100996535	0	0	0	0	0	0	0	0	1	0	0	0
LOC100996541	11	0	6	0	3	0	1	1	1	0	4	0
LOC100996546	1	2	5	2	1	0	2	2	1	2	1	2
LOC100996547	2	0	0	0	0	0	0	0	1	0	0	0
LOC100996567	11	1	6	0	2	1	0	0	5	0	3	0
LOC100996571	48	6	24	2	15	1	7	14	23	0	16	1
LOC100996573	0	1	0	2	0	0	0	0	0	0	0	0
LOC100996574	4	0	1	0	1	0	0	1	0	0	1	0
LOC100996575	23	75	29	30	24	59	31	50	19	18	37	5
LOC100996577	18	26	28	8	27	24	6	30	21	5	21	0
LOC100996584	5	2	1	1	1	2	1	1	3	1	3	1
LOC100996594	6	0	2	0	2	0	0	0	0	0	2	0
LOC100996598	0	1	0	0	0	0	0	1	0	0	0	0
LOC100996599	0	0	0	0	1	0	0	0	1	0	1	0

LOC100996602	9	0	4	0	0	0	0	0	5	0	4	0
LOC100996605	2	2	1	1	0	1	3	2	1	0	3	0
LOC100996606	1	0	1	2	0	0	1	1	3	0	0	0
LOC100996610	21	6	7	0	5	1	2	6	12	4	9	2
LOC100996612	12	6	9	9	4	4	5	6	7	4	6	1
LOC100996614	13	1	5	0	2	0	0	0	6	0	3	0
LOC100996619	58	6	22	0	14	1	1	3	19	1	10	0
LOC100996625	2	0	0	0	0	0	0	0	1	0	1	0
LOC100996631	3	0	2	0	1	0	0	0	4	0	2	0
LOC100996633	7	1	1	2	0	3	3	6	0	0	3	1
LOC100996634	15	6	13	10	6	7	7	28	16	0	11	2
LOC100996641	11	10	13	11	2	7	7	24	12	1	9	0
LOC100996642	17	79	12	24	27	32	49	63	27	18	26	6
LOC100996643	17	9	9	0	9	8	8	22	21	4	16	7
LOC100996644	2	0	0	0	1	0	0	0	0	0	0	0
LOC100996646	4	0	6	0	2	0	0	1	2	0	2	0
LOC100996648	42	0	4	0	7	0	0	0	4	0	3	0
LOC100996661	0	0	1	1	0	0	0	0	0	0	0	0
LOC100996675	5	1	1	0	0	0	0	0	3	0	2	0

LOC100996677	11	2	10	1	1	4	2	3	2	0	5	0
LOC100996685	14	1	13	0	2	1	0	0	4	0	2	0
LOC100996689	99	10	46	4	15	2	3	4	43	0	22	0
LOC100996693	0	2	0	4	0	1	0	3	2	0	1	0
LOC100996701	0	0	0	0	0	0	0	0	1	0	0	0
LOC100996702	95	3	40	1	14	3	2	3	28	1	16	0
LOC100996863	0	0	0	0	1	0	0	0	0	0	0	0
LOC100996885	7	0	8	0	4	0	0	0	2	0	1	0
LOC101059906	6	0	4	0	1	0	0	0	2	0	0	0
LOC101059911	50	0	30	0	9	0	0	0	30	0	26	0
LOC101059914	3	0	0	0	1	0	0	0	2	0	0	0
LOC101059915	10	0	5	0	1	0	2	0	0	0	2	0
LOC101059918	47	60	27	28	24	36	51	45	29	23	58	8
LOC101059921	4	5	2	1	4	5	1	7	6	2	6	0
LOC101059922	43	125	27	85	57	84	96	131	61	25	76	22
LOC101059934	19	0	13	0	3	1	0	0	8	0	4	0
LOC101059935	10	5	25	5	11	4	8	9	14	9	5	1
LOC101059938	38	174	48	49	26	107	84	151	65	61	89	29
LOC101059952	9	0	9	0	4	0	0	0	3	0	3	0

LOC101059953	55	148	63	56	71	165	111	142	52	45	25	32
LOC101059957	18	0	8	0	7	1	0	0	11	0	9	0
LOC101059961	18	102	37	59	48	61	70	104	46	25	47	27
LOC101059966	0	1	0	0	1	0	1	2	0	0	0	0
LOC101059970	23	48	24	28	49	47	43	62	27	32	33	12
LOC101059976	11	19	3	15	8	13	30	18	10	1	10	0
LOC101059980	6	16	2	21	9	13	12	11	4	6	5	3
LOC101059981	10	0	10	0	3	0	0	0	0	0	3	0
LOC101059984	17	8	10	9	4	8	11	22	16	1	15	4
LOC101059991	6	0	0	0	0	1	0	1	2	0	2	0
LOC101060011	0	0	1	0	0	0	0	0	0	0	0	0
LOC101060017	34	0	28	0	10	3	1	1	8	1	9	0
LOC101060018	1	0	0	0	1	0	0	0	2	0	0	0
LOC101060026	15	0	15	1	10	3	4	2	10	2	8	1
LOC101060028	6	0	2	0	1	0	0	0	5	0	0	0
LOC101060029	12	0	9	0	1	0	0	1	6	0	3	0
LOC101060037	2	0	0	3	0	2	7	3	1	2	0	2
LOC101060041	17	13	6	4	7	3	6	5	10	2	9	2
LOC101060047	12	0	5	0	0	0	0	1	0	0	2	0

LOC101060085	5	0	2	0	5	0	0	1	2	0	2	0
LOC101060092	47	74	46	64	126	156	55	285	76	44	130	47
LOC101060101	3	0	0	0	4	0	0	0	1	1	4	0
LOC101060115	4	0	0	0	0	0	0	0	0	0	0	0
LOC101060117	18	0	12	0	9	0	0	0	14	0	17	0
LOC101060128	7	0	1	0	2	0	0	1	7	0	4	0
LOC101060133	2	0	0	0	0	0	0	0	1	0	1	0
LOC101060136	0	0	0	0	0	0	0	0	1	0	0	0
LOC101060138	3	19	4	13	6	5	17	24	20	13	4	9
LOC101060140	0	0	0	0	0	0	0	0	3	0	0	0
LOC101060155	0	0	0	0	0	1	0	0	1	0	0	0
LOC101060161	3	0	1	0	0	0	0	0	1	0	0	0
LOC101060163	37	0	34	0	7	1	0	0	31	1	17	3
LOC101060166	14	6	8	8	5	8	2	4	5	0	3	1
LOC101060167	2	0	3	0	2	0	0	0	3	0	1	0
LOC101060171	14	3	5	5	5	2	1	0	3	0	5	0
LOC101060175	42	120	24	79	65	119	81	146	73	49	70	21
LOC101060179	0	0	0	0	0	1	0	1	0	0	0	0
LOC101060181	17	55	15	34	35	28	33	60	25	20	33	14

LOC101060183	4	0	0	0	0	0	0	0	1	1	0	0
LOC101060193	0	0	3	0	2	0	0	0	0	0	2	0
LOC101060198	25	5	24	15	6	4	0	14	21	6	12	3
LOC101060200	23	44	13	33	31	20	37	49	32	28	30	19
LOC147646	7	0	3	0	0	0	0	0	1	0	1	0
LOC147670	9	3	2	1	3	2	1	3	10	0	2	0
LOC149373	2	0	1	0	0	0	0	0	3	0	0	0
LOC152586	1	0	0	0	3	0	0	0	2	0	0	0
LOC154872	4	1	3	0	1	0	0	0	1	0	1	0
LOC158434	18	0	3	1	2	0	1	1	2	0	5	0
LOC200726	5	0	5	0	1	0	0	0	5	0	3	0
LOC256021	24	0	5	1	4	1	2	0	8	1	4	0
LOC283403	7	0	7	0	2	0	0	0	2	0	3	0
LOC283710	2	0	2	0	0	0	0	0	0	0	0	0
LOC284385	21	15	18	6	9	5	7	10	3	3	11	3
LOC285423	26	5	21	7	9	17	1	8	21	2	10	0
LOC285556	708	5301	1162	5360	2116	3939	3481	2168	1940	887	1902	485
LOC286238	9	0	3	0	0	0	0	0	0	0	1	0
LOC338667	9	0	6	0	0	0	0	0	5	0	1	0
LOC339760	15	0	6	0	1	0	0	0	3	0	2	0
LOC339862	21	2	12	1	2	0	0	1	9	0	5	0
LOC377711	1	1	0	0	1	1	1	2	0	1	1	0
LOC388210	29	18	22	4	7	3	0	6	16	0	5	0
LOC388276	2	0	4	0	2	0	0	0	3	0	3	0
LOC388813	6	0	4	0	3	1	0	0	7	0	4	0
LOC388849	2	0	6	1	0	1	0	0	0	0	0	0
LOC389895	1	0	0	0	0	1	0	0	1	0	0	0

LOC390877	2	0	0	0	0	0
	0	0	1	0	0	0
LOC390956	2	0	1	0	3	0
	0	0	2	0	0	0
LOC391322	1	6	1	5	3	3
	0	2	8	1	1	3
LOC391722	4	0	3	0	3	2
	0	3	2	1	3	2
LOC392288	9	0	7	0	3	1
	0	0	2	0	3	0
LOC400499	18	24	11	6	3	5
	3	4	13	1	4	4
LOC400682	9	13	12	12	9	7
	13	4	13	2	6	1
LOC401052	16	10	6	7	5	3
	4	8	4	1	6	2
LOC401180	4	0	0	0	0	0
	0	0	0	0	1	0
LOC402160	6	1	6	0	0	0
	0	0	5	0	3	0
LOC402269	9	0	8	0	0	1
	0	1	2	0	3	0
LOC440233	35	0	7	0	11	0
	0	0	19	0	11	0
LOC440243	84	1	39	0	14	0
	0	0	34	0	25	0
LOC440292	25	7	10	3	7	1
	1	8	10	1	7	0
LOC440335	3	0	4	0	2	0
	0	0	3	0	2	0
LOC440563	22	0	7	0	0	0
	0	0	8	0	4	0
LOC440981	1	0	0	0	0	0
	0	0	3	0	1	0
LOC441098	12	1	2	1	1	0
	0	0	4	0	0	0
LOC441124	1	14	1	0	0	4
	1	9	6	3	3	2
LOC441239	15	38	9	34	20	19
	15	31	6	11	28	2
LOC554223	12	3	3	0	1	2
	0	0	4	0	3	1
LOC642131	2	0	9	0	1	0
	0	2	7	1	2	0
LOC642441	3	0	3	0	0	0
	0	0	0	0	0	0
LOC642643	28	3	13	1	6	3
	2	0	26	0	9	0
LOC642778	12	13	8	5	6	4
	9	15	4	9	10	2
LOC642799	20	30	11	17	27	23
	17	40	18	3	27	5
LOC643037	5	0	3	0	1	0
	0	0	4	0	0	0
LOC643355	12	2	8	1	1	1
	0	0	2	0	6	0
LOC643669	5	9	2	6	8	9
	3	4	3	2	1	2

LOC643802	10	4	2	0	3	0
	1	1	1	1	2	0
LOC644249	14	12	16	4	13	5
	2	6	17	2	17	0
LOC644634	3	1	0	0	0	0
	0	1	0	1	0	0
LOC645177	18	5	12	1	5	2
	2	0	7	0	9	0
LOC645202	17	1	5	0	2	2
	1	0	6	0	6	0
LOC645359	2	0	6	0	2	0
	0	0	7	0	0	0
LOC646021	56	129	32	83	63	153
	112	250	66	33	93	12
LOC646498	4	0	6	0	0	0
	0	0	0	1	0	0
LOC646543	6	8	4	7	7	3
	8	7	7	0	5	3
LOC646670	29	2	19	0	2	3
	2	2	9	0	14	0
LOC646730	7	0	6	0	3	0
	0	0	1	0	0	0
LOC649238	7	0	1	0	3	0
	0	0	2	0	0	0
LOC650157	0	0	2	0	0	1
	0	0	5	0	1	0
LOC650293	1	0	2	0	0	0
	0	0	2	0	0	0
LOC653375	14	51	2	35	19	39
	41	0	1	7	0	11
LOC728026	9	0	5	0	2	0
	0	3	5	0	1	0
LOC728065	17	0	6	0	2	0
	0	0	2	0	4	0
LOC728392	4	14	4	3	4	10
	3	13	4	2	5	5
LOC728503	9	0	4	0	1	0
	1	0	3	0	1	0
LOC728637	7	0	7	1	3	1
	0	0	7	0	2	1
LOC728671	3	1	4	0	1	0
	0	0	0	0	0	0
LOC728715	8	8	6	10	3	3
	5	3	7	1	11	0
LOC728728	10	21	7	3	8	16
	10	21	9	7	8	0
LOC728734	21	9	6	4	6	3
	2	9	3	1	6	2
LOC728741	16	4	9	2	2	1
	1	3	6	0	10	3
LOC728763	10	0	12	2	4	0
	0	0	2	0	3	0
LOC728819	5	3	2	2	2	6
	3	9	6	4	4	1
LOC728888	55	148	24	85	42	64
	68	146	61	22	73	23
LOC729020	8	0	2	0	2	0
	0	0	5	0	1	0

LOC729059	2	0	0	0	1	0
	0	0	1	0	1	0
LOC729159	1	0	2	0	0	1
	0	0	3	0	0	0
LOC729162	0	8	0	0	2	0
	2	2	0	1	1	1
LOC729175	4	1	2	0	4	2
	0	1	2	0	0	0
LOC729264	18	0	10	1	6	0
	0	4	11	2	7	0
LOC729454	3	0	4	1	0	0
	0	1	1	0	1	0
LOC729458	3	0	0	0	0	0
	0	0	1	0	1	0
LOC729461	12	1	46	0	4	0
	1	0	54	0	29	0
LOC729574	1	0	1	0	1	0
	0	0	0	0	0	0
LOC729587	2	0	0	0	0	1
	0	0	1	0	1	0
LOC729974	1	0	2	0	1	0
	0	0	2	0	0	0
LOC730058	6	0	8	0	4	0
	0	0	6	0	7	0
LOC730159	5	0	6	0	0	0
	0	0	5	0	4	0
LOC730183	2	6	0	7	1	3
	3	6	3	4	6	0
LOC730256	1	2	2	0	1	0
	1	0	0	0	0	0
LOC730268	84	144	82	95	77	136
	99	142	83	63	84	30
LOC731282	1	2	2	2	0	3
	0	2	0	0	0	0
LOC731932	37	2	13	5	8	1
	1	0	8	0	10	0
LOC732265	2	0	1	0	0	1
	1	6	4	0	0	1
LOC81691	26	23	23	16	14	10
	17	20	29	6	16	2
LOH12CR1	27	51	12	35	22	37
	39	58	26	9	40	16
LONP1	55	329	57	168	145	155
	210	282	114	77	164	53
LONP2	175	805	211	613	462	698
	599	935	411	318	485	200
LONRF1	147	624	102	615	337	369
	473	487	296	160	382	93
LONRF2	153	493	150	569	258	470
	353	773	358	185	295	111
LONRF3	51	78	47	94	60	79
	63	60	30	36	56	17
LOR	0	0	2	0	0	0
	0	0	0	0	0	0
LOX	26	24	15	25	14	13
	30	38	26	1	24	12
LOXHD1	80	15	50	15	30	7
	14	10	48	4	25	2

LOXL1	9	14	4	37	10	23
	4	15	12	5	21	13
LOXL2	24	34	22	35	18	20
	12	36	15	10	16	8
LOXL3	20	26	16	20	10	14
	3	11	24	3	15	3
LOXL4	34	42	45	45	20	31
	16	24	18	8	19	7
LPA	96	2	62	2	20	1
	4	1	51	1	38	1
LPAR1	33	83	40	107	36	73
	58	106	53	25	54	18
LPAR2	3	1	4	3	0	1
	1	5	5	1	2	0
LPAR3	8	0	5	0	0	0
	0	1	5	0	1	0
LPAR4	27	3	4	1	5	1
	0	1	7	3	2	1
LPAR5	10	2	4	1	0	2
	0	0	11	0	1	1
LPAR6	55	168	41	89	93	115
	154	212	105	91	141	23
LPCAT1	31	56	17	35	24	56
	44	44	34	15	23	12
LPCAT2	40	60	37	96	44	53
	41	126	51	10	35	13
LPCAT3	69	263	55	143	94	147
	118	303	109	58	97	37
LPCAT4	20	29	17	44	28	35
	28	33	22	6	22	6
LPGAT1	195	726	170	613	378	570
	674	845	331	220	414	185
LPHN1	20	14	21	24	15	18
	9	15	17	10	8	5
LPHN2	86	291	86	210	134	172
	179	204	120	63	125	41
LPHN3	65	110	45	50	39	23
	38	76	35	12	46	52
LPIN1	415	2045	405	1749	1210	1671
	2048	2707	1052	596	980	340
LPIN2	36	62	31	51	39	61
	53	73	40	21	39	13
LPIN3	22	22	14	25	13	16
	18	43	17	10	17	4
LPL	128	607	60	234	299	218
	556	2907	565	274	681	335
LPO	15	0	12	0	6	0
	0	0	7	0	5	0
LPP	534	2080	406	1307	800	1191
	1395	3746	1337	759	1654	760
LPPR1	16	0	6	0	8	6
	0	0	16	0	3	0
LPPR2	21	51	8	25	26	25
	45	33	24	8	24	9
LPPR3	5	0	5	0	3	0
	0	0	2	0	0	0
LPPR4	28	22	15	29	24	20
	9	16	25	10	19	6

LPPR5	14	4	10	6	10	1
	0	0	11	0	3	0
LPXN	36	55	20	61	28	34
	36	86	42	21	51	8
LRAT	25	8	14	1	9	2
	3	2	11	2	8	1
LRBA	256	878	306	730	381	764
	714	991	580	280	749	160
LRCH1	80	165	62	110	76	80
	102	171	115	54	96	26
LRCH2	34	50	9	36	36	23
	38	68	38	9	40	7
LRCH3	99	415	130	269	200	288
	322	440	181	164	250	54
LRCH4	21	63	20	38	25	61
	64	90	31	18	26	15
LRCOL1	0	0	1	0	0	0
	1	1	0	0	0	0
LRFN1	3	0	3	1	0	2
	2	0	4	0	0	0
LRFN2	8	0	6	1	0	0
	0	0	2	1	3	0
LRFN3	2	6	2	4	4	3
	1	1	2	3	2	1
LRFN4	3	7	3	2	6	7
	15	25	5	6	4	5
LRFN5	26	7	12	10	6	2
	8	20	10	4	19	1
LRG1	6	1	1	1	1	0
	0	1	2	1	1	0
LRGUK	28	3	24	7	14	6
	5	3	26	0	10	1
LRIF1	63	224	39	213	138	172
	163	198	126	73	145	42
LRIG1	187	660	123	534	415	539
	539	699	377	202	390	157
LRIG2	97	328	96	277	169	249
	292	377	182	113	209	70
LRIG3	80	188	64	168	114	131
	115	242	149	77	110	40
LRIT1	5	0	2	0	1	1
	1	0	2	0	1	0
LRIT2	13	0	8	0	1	0
	0	0	5	0	2	0
LRIT3	27	5	11	5	10	12
	2	11	18	4	7	0
LRMP	30	37	26	31	16	20
	31	27	38	13	31	3
LRP1	406	1602	484	1730	840	1018
	897	1710	618	359	771	505
LRP10	43	127	19	102	44	78
	89	113	77	42	68	26
LRP11	20	116	37	76	49	79
	49	100	42	27	42	23
LRP12	87	221	53	232	124	205
	230	189	108	46	127	33
LRP1B	262	734	353	258	404	207
	254	704	261	88	349	281

LRP2	165	1	102	2	35	1
	0	1	93	0	72	0
LRP2BP	30	87	40	76	32	19
	18	82	38	16	21	17
LRP3	12	74	13	48	34	67
	50	80	47	26	49	26
LRP4	131	285	125	140	221	124
	165	229	80	79	106	69
LRP5	37	134	26	74	47	69
	80	108	38	26	50	28
LRP5L	12	14	11	5	9	15
	14	24	2	2	14	1
LRP6	345	1844	557	1901	817	1612
	1178	1400	764	442	806	265
LRP8	36	10	34	12	10	4
	7	10	32	5	19	2
LRPAP1	114	349	106	267	183	285
	273	429	198	102	255	64
LRPPRC	556	3360	528	2486	1385	1821
	2231	3573	1308	1000	1802	535
LRR1	14	32	8	14	10	6
	13	34	12	9	26	7
LRRC1	16	20	21	9	10	13
	11	22	24	6	18	7
LRRC10	5	0	3	0	0	0
	0	0	5	0	0	0
LRRC10B	3	1	1	0	1	1
	0	0	2	0	0	0
LRRC14	18	38	22	28	15	26
	29	72	22	15	35	7
LRRC14B	28	103	24	126	54	114
	142	107	69	27	74	20
LRRC15	27	3	18	3	6	3
	0	0	24	0	5	2
LRRC16A	61	34	55	66	36	32
	20	58	32	8	39	19
LRRC16B	31	1	17	1	2	1
	1	8	15	0	7	0
LRRC17	21	47	24	56	31	57
	24	64	23	7	26	6
LRRC18	11	0	6	0	2	0
	0	1	2	0	4	0
LRRC19	21	0	4	0	10	2
	0	3	4	0	4	0
LRRC2	771	2321	384	1455	1479	1870
	2641	4287	1644	1427	2958	743
LRRC20	275	1201	180	792	484	792
	772	1105	588	332	743	210
LRRC23	27	51	13	9	27	22
	14	23	29	7	23	11
LRRC24	4	2	3	2	2	0
	1	6	2	2	3	0
LRRC25	4	8	1	10	1	3
	8	3	9	3	3	0
LRRC27	52	27	23	20	30	15
	24	18	21	8	34	3
LRRC28	59	91	41	93	47	98
	102	129	82	34	70	24

LRRC29	5	11	4	9	5	5
	6	12	5	3	6	0
LRRC3	5	8	3	2	2	2
	3	6	1	1	6	2
LRRC30	27	170	66	138	67	169
	81	63	64	43	67	16
LRRC31	16	0	6	0	4	0
	0	0	7	0	7	0
LRRC32	39	68	22	53	34	25
	40	69	48	25	49	13
LRRC33	4	7	4	8	1	5
	11	2	9	1	4	2
LRRC34	9	6	6	15	5	5
	9	12	12	1	9	2
LRRC36	27	0	9	0	5	1
	0	2	17	1	10	0
LRRC37A	55	72	16	76	48	43
	13	95	38	28	31	13
LRRC37A2	178	475	90	545	229	329
	129	399	168	148	149	37
LRRC37A3	142	213	59	159	78	97
	63	77	91	35	77	21
LRRC37B	57	106	25	74	48	53
	68	99	36	26	61	17
LRRC38	29	78	32	151	49	111
	76	64	52	13	37	21
LRRC39	1213	6777	978	3527	2649	4880
	5373	7320	2343	2271	3554	901
LRRC3B	27	55	6	44	21	10
	113	148	22	20	106	7
LRRC3C	5	0	0	0	0	0
	0	0	4	0	0	0
LRRC4	25	14	17	4	9	5
	2	4	12	0	6	1
LRRC40	62	265	68	187	141	202
	257	392	155	81	195	54
LRRC41	47	180	56	119	94	138
	128	184	83	50	93	24
LRRC42	20	70	12	27	39	41
	45	51	40	18	35	11
LRRC43	12	2	5	2	4	3
	0	0	5	0	1	1
LRRC45	12	15	8	10	8	11
	5	18	11	5	6	3
LRRC46	15	2	14	0	1	3
	1	0	8	0	3	0
LRRC47	103	421	75	296	183	296
	374	537	199	134	301	76
LRRC48	16	10	11	6	7	10
	6	13	14	4	10	1
LRRC49	26	20	21	24	22	21
	24	45	27	9	32	6
LRRC4B	1	0	0	0	0	0
	0	1	2	0	0	0
LRRC4C	16	3	14	9	3	1
	1	5	11	1	4	1
LRRC52	6	4	3	1	0	1
	3	9	1	1	2	2

LRRC55	30	5	22	9	15	2
	11	2	20	6	12	2
LRRC56	11	16	10	20	3	8
	5	1	10	2	6	6
LRRC57	31	55	26	47	39	40
	49	48	29	13	34	12
LRRC58	108	337	103	424	177	375
	308	426	209	128	261	79
LRRC59	32	116	39	93	56	112
	110	119	74	36	72	22
LRRC6	26	12	13	6	11	14
	7	9	18	1	12	1
LRRC61	5	1	1	1	1	2
	0	2	1	0	0	0
LRRC63	17	2	15	0	6	1
	1	4	7	0	2	0
LRRC66	73	204	73	151	151	243
	103	277	93	71	108	30
LRRC69	16	14	15	19	16	33
	14	30	7	9	18	4
LRRC7	51	41	58	35	62	4
	32	175	36	52	44	12
LRRC70	0	0	0	1	0	0
	0	0	0	0	0	0
LRRC71	8	0	8	1	1	1
	0	2	4	0	5	0
LRRC72	12	0	8	1	1	0
	0	0	4	0	0	0
LRRC73	15	1	4	0	3	0
	0	1	4	0	3	0
LRRC8A	36	77	20	62	58	45
	86	99	45	23	50	11
LRRC8B	87	148	57	120	94	83
	89	234	142	41	112	33
LRRC8C	77	229	46	146	102	96
	229	255	127	91	135	45
LRRC8D	36	95	22	95	47	79
	124	115	79	51	75	30
LRRC8E	17	0	9	1	2	0
	1	0	3	1	0	0
LRRCC1	35	76	38	36	35	35
	57	54	42	17	44	8
LRRD1	14	4	10	1	8	3
	1	4	15	1	12	1
LRRFIP1	424	2359	452	1396	1151	2084
	1780	2741	1021	679	1232	483
LRRFIP2	339	1859	391	1841	913	1574
	1457	1557	855	578	1206	375
LRRIQ1	40	0	30	3	21	2
	2	2	32	0	24	0
LRRIQ3	20	1	8	1	2	0
	1	0	19	0	10	0
LRRIQ4	9	0	9	0	4	3
	0	1	9	1	4	0
LRRK1	45	36	20	46	15	27
	25	29	36	11	27	18
LRRK2	203	695	382	533	302	564
	475	591	354	209	485	131

LRRN1	79	134	26	167	193	251
	114	93	61	52	85	10
LRRN2	18	9	10	6	2	1
	7	10	5	1	5	1
LRRN3	25	43	43	29	16	17
	21	27	31	2	16	6
LRRN4	12	0	3	0	3	0
	1	0	2	0	3	0
LRRN4CL	11	26	14	28	23	24
	18	31	7	3	12	8
LRRTM1	5	0	2	2	0	0
	0	0	0	0	0	0
LRRTM2	43	6	34	11	19	10
	7	17	35	2	29	2
LRRTM3	36	17	38	15	23	25
	6	26	23	9	27	1
LRRTM4	44	152	43	123	91	145
	62	87	86	20	65	23
LRSAM1	43	124	44	56	59	60
	80	78	43	37	48	18
LRTM1	6	1	4	1	1	1
	0	0	3	0	3	0
LRTM2	14	0	10	0	10	0
	0	0	3	0	4	0
LRTOMT	25	30	11	16	13	25
	13	29	18	6	14	4
LRWD1	13	23	6	10	11	10
	8	26	14	5	9	4
LSAMP	54	3	31	8	19	4
	9	6	23	0	14	2
LSG1	62	288	62	227	167	176
	207	325	147	98	181	73
LSM1	37	150	16	87	65	78
	120	198	66	26	76	19
LSM10	12	64	4	35	25	40
	89	24	28	12	33	8
LSM11	11	13	8	13	11	5
	9	13	13	7	16	4
LSM12	78	333	75	248	174	272
	353	392	168	100	211	63
LSM14A	154	841	144	579	332	677
	693	827	330	226	443	146
LSM14B	70	391	69	241	152	283
	289	402	154	101	197	55
LSM2	15	33	11	24	12	21
	36	50	20	10	24	10
LSM3	118	693	100	531	275	542
	770	1152	343	203	457	102
LSM4	63	420	37	142	111	199
	273	491	112	62	153	47
LSM5	54	254	43	250	92	196
	280	377	123	73	172	40
LSM6	22	63	19	49	35	31
	81	103	42	22	44	11
LSM7	11	25	3	17	6	13
	17	47	13	11	9	1
LSMD1	37	112	13	73	33	73
	84	163	48	27	40	14

LSP1	48	193	54	152	65	155
	85	150	87	37	60	24
LSR	6	17	4	10	3	6
	10	8	9	2	9	2
LSS	79	192	59	143	77	180
	156	282	143	59	161	47
LST1	2	3	3	7	1	5
	1	7	3	0	0	0
LTA	6	0	6	0	3	0
	1	0	4	0	1	0
LTA4H	106	490	142	359	286	381
	481	623	298	152	296	74
LTB	5	1	0	1	0	2
	0	0	1	0	0	0
LTB4R	25	22	14	19	14	25
	8	25	29	11	32	2
LTB4R2	1	4	3	0	2	2
	4	9	2	2	6	0
LTBP1	182	629	143	392	355	371
	443	606	384	183	410	135
LTBP2	166	400	150	471	271	631
	263	660	178	139	264	165
LTBP3	77	219	61	176	104	141
	188	185	111	48	105	57
LTBP4	67	220	55	268	101	146
	138	211	124	51	167	73
LTBR	28	101	19	60	34	79
	52	69	25	31	42	17
LTC4S	0	6	4	2	0	1
	0	2	4	0	2	0
LTF	26	15	17	10	11	11
	14	18	34	4	18	1
LTK	14	1	13	1	3	3
	0	3	5	0	2	0
LTN1	263	1007	283	872	632	814
	701	1287	602	303	700	216
LTV1	59	252	53	130	110	185
	139	232	82	54	101	37
LUC7L	47	187	51	109	81	129
	151	214	78	67	79	37
LUC7L2	186	882	206	507	372	713
	695	1016	424	356	669	162
LUC7L3	347	1415	337	1159	727	1177
	1086	1962	831	637	977	346
LUM	144	465	191	869	382	603
	567	801	250	194	275	156
LURAP1	11	5	6	6	3	0
	0	3	2	0	5	0
LURAP1L	7	18	11	18	11	22
	13	15	20	4	19	8
LUZP1	69	196	61	109	71	111
	107	138	94	55	72	28
LUZP2	22	1	19	1	7	0
	1	0	20	1	13	0
LUZP4	7	0	1	0	2	0
	0	0	1	0	2	1
LUZP6	97	468	86	421	188	408
	324	374	229	116	234	61

LXN	13	36	7	10	10	19
	28	27	27	10	19	5
LY6D	1	0	2	0	2	0
	0	0	0	0	0	0
LY6E	6	18	3	24	5	12
	20	23	11	18	18	5
LY6G5B	8	13	1	4	3	10
	8	15	7	2	10	2
LY6G5C	6	4	3	3	2	2
	3	0	4	0	1	0
LY6G6C	4	0	0	0	1	0
	0	0	0	0	0	0
LY6G6D	3	0	1	0	0	0
	0	0	4	0	0	0
LY6G6F	6	0	2	0	0	0
	0	0	1	0	1	0
LY6H	1	0	5	0	0	0
	0	0	1	0	2	0
LY6K	11	4	4	8	10	21
	24	8	5	8	5	3
LY75	7	17	7	8	1	4
	6	14	8	6	18	5
LY75-CD302	92	143	66	90	83	108
	62	106	87	31	66	8
LY86	4	0	3	2	1	0
	0	2	7	0	2	1
LY9	20	1	10	1	2	0
	0	2	9	0	6	0
LY96	0	7	2	7	0	5
	7	14	3	0	5	1
LYAR	45	158	30	61	59	77
	62	133	51	36	51	29
LYG1	12	15	6	7	9	14
	5	5	12	3	8	4
LYG2	9	3	7	0	2	0
	0	0	3	1	1	0
LYL1	5	7	3	2	4	5
	4	9	4	0	2	1
LYN	30	30	19	33	20	35
	31	44	31	19	33	6
LYNX1	23	60	18	13	21	28
	25	40	23	9	23	17
LYPD1	9	0	12	1	3	0
	0	3	4	0	6	0
LYPD2	3	0	0	0	0	0
	0	0	0	0	0	0
LYPD3	4	1	1	0	1	0
	0	0	1	0	1	0
LYPD4	8	0	5	0	2	1
	0	0	5	0	2	0
LYPD5	13	1	4	2	0	4
	2	1	4	1	5	0
LYPD6	19	1	9	2	6	3
	3	2	18	1	11	2
LYPD6B	7	0	6	0	4	0
	0	1	13	0	4	0
LYPD8	1	1	1	0	0	0
	0	0	0	0	0	0

LYPLA1	134	653	133	605	297	437
	782	797	368	161	378	107
LYPLA2	11	36	11	27	19	28
	46	45	21	8	26	4
LYPLAL1	79	344	71	177	127	187
	279	536	188	75	215	71
LYRM1	27	156	26	118	63	105
	159	246	74	54	89	25
LYRM2	145	538	90	309	245	393
	393	489	258	165	292	99
LYRM4	21	42	12	32	12	14
	48	46	25	14	20	5
LYRM5	89	462	64	293	170	293
	442	588	227	95	231	48
LYRM7	226	1293	201	792	524	734
	893	1189	421	358	644	200
LYRM9	16	99	22	52	18	72
	75	85	44	20	45	15
LYSMD1	32	82	19	50	32	62
	81	91	46	32	63	16
LYSMD2	19	26	18	62	18	78
	71	80	81	17	28	9
LYSMD3	59	360	62	270	185	247
	346	335	146	98	218	58
LYSMD4	19	61	17	21	17	54
	39	107	26	25	40	11
LYST	177	704	238	526	317	478
	443	628	304	187	367	152
LYVE1	23	81	20	111	145	188
	41	407	89	34	81	48
LYZ	20	120	15	58	42	86
	74	117	29	52	32	13
LYZL1	6	0	0	0	0	0
	0	0	4	0	2	0
LYZL2	7	0	1	0	1	0
	0	0	3	0	3	0
LYZL4	8	0	3	0	2	0
	0	0	1	0	1	0
LYZL6	4	0	1	0	0	0
	0	0	1	0	2	0
LZIC	33	197	42	120	91	115
	124	170	70	40	85	26
LZTFL1	25	51	20	60	46	55
	55	99	41	27	39	14
LZTR1	55	174	57	125	83	133
	96	177	80	52	69	33
LZTS1	8	6	10	4	3	3
	9	7	17	8	8	0
LZTS2	24	119	35	58	50	72
	96	99	61	29	83	20
M6PR	92	293	52	247	189	237
	241	514	214	107	192	88
MAATS1	34	4	25	15	15	9
	1	9	27	4	9	4
MAB21L1	34	65	28	52	54	58
	70	65	31	29	43	9
MAB21L2	18	0	8	0	11	0
	0	1	12	0	2	0

MAB21L3	21	0	22	1	5	1
	0	1	9	0	5	0
MACC1	28	4	24	2	20	5
	0	0	19	2	17	0
MACF1	767	4085	984	2726	1897	2614
	2323	3627	1573	1279	1934	857
MACROD1	117	662	51	312	199	338
	662	782	305	137	320	63
MACROD2	47	60	60	47	39	39
	42	23	63	11	44	13
MAD1L1	47	185	50	111	65	112
	110	154	106	45	92	33
MAD2L1	21	16	12	15	14	13
	12	26	12	2	12	7
MAD2L1BP	28	71	34	65	45	90
	84	159	47	23	66	17
MAD2L2	18	63	21	81	33	78
	88	143	47	24	40	20
MADCAM1	0	0	0	0	0	0
	0	0	2	0	1	0
MADD	135	361	110	353	190	260
	369	328	185	136	226	64
MAEA	42	146	33	110	73	132
	176	165	105	39	93	28
MAEL	10	0	12	6	6	4
	0	2	13	1	9	2
MAF	293	1761	400	1832	649	1579
	1234	1415	747	438	641	174
MAF1	175	900	93	512	360	523
	657	1057	374	303	477	138
MAFA	27	148	35	136	87	154
	97	98	84	36	47	18
MAFB	31	138	32	97	70	93
	160	241	54	32	65	39
MAFF	18	10	4	3	6	12
	6	17	7	3	4	0
MAFG	48	216	48	169	103	165
	175	208	96	60	95	54
MAFK	45	73	22	60	55	77
	73	196	63	65	89	30
MAG	12	0	9	0	0	0
	1	0	5	0	2	0
MAGEA1	7	0	1	0	4	0
	0	0	3	0	1	0
MAGEA10	0	0	0	2	0	0
	0	0	0	0	1	0
MAGEA10-MAGEA5		6	0	1	0	1
	0	0	0	2	0	0
	0					
MAGEA11	17	0	2	0	0	0
	0	3	1	0	0	0
MAGEA12	8	0	3	0	1	0
	0	0	1	0	0	0
MAGEA2	16	0	1	0	1	0
	0	0	2	0	0	0
MAGEA2B	1	0	0	0	0	0
	0	0	0	0	1	0

MAGEA3	5	0	3	0	0	0
	0	0	2	0	1	0
MAGEA4	10	0	6	0	2	0
	0	0	2	0	1	0
MAGEA6	0	0	1	0	3	0
	0	0	1	0	0	0
MAGEA8	7	1	2	0	2	0
	0	0	3	0	0	0
MAGEA9	9	0	2	0	1	0
	1	0	2	0	1	0
MAGEA9B	6	0	9	0	1	0
	0	0	1	0	0	0
MAGEB1	17	0	3	0	7	0
	0	0	1	0	1	0
MAGEB10	22	0	5	0	1	1
	0	0	5	0	2	0
MAGEB16	10	0	2	0	5	0
	0	0	0	0	0	0
MAGEB17	5	0	3	2	1	1
	1	1	2	0	2	1
MAGEB18	9	0	1	0	1	0
	0	0	1	0	1	0
MAGEB2	5	0	3	0	0	0
	0	0	1	0	0	0
MAGEB3	14	0	3	0	4	0
	0	0	5	0	4	0
MAGEB4	17	0	2	0	2	0
	1	0	3	0	2	0
MAGEB6	15	1	2	0	1	0
	0	1	1	0	2	0
MAGEC1	15	0	3	0	3	0
	0	0	5	0	0	0
MAGEC2	10	0	2	0	1	0
	0	0	1	0	1	0
MAGEC3	15	0	6	3	4	1
	0	0	5	0	4	0
MAGED1	57	302	88	225	106	158
	221	151	115	59	113	48
MAGED2	127	562	182	453	197	350
	437	641	359	132	353	87
MAGED4	10	2	4	2	2	4
	1	0	5	0	2	0
MAGED4B	20	6	5	2	2	4
	0	1	4	0	0	2
MAGEE1	11	12	7	16	7	27
	22	15	17	11	13	3
MAGEE2	10	2	5	0	4	0
	0	0	3	0	3	0
MAGEF1	40	296	48	190	98	185
	142	294	122	55	116	27
MAGEH1	10	61	7	58	23	37
	50	74	24	12	22	12
MAGEL2	10	1	4	0	1	0
	0	2	4	0	1	0
MAGI1	150	361	123	228	177	224
	324	363	171	123	308	114
MAGI2	57	77	47	54	51	66
	86	132	61	21	74	12

MAGI3	65	138	63	90	79	135
	69	139	78	32	70	26
MAGIX	14	10	4	3	6	2
	3	7	1	0	3	0
MAGOH	24	62	11	35	28	35
	77	95	46	22	49	10
MAGOHB	20	69	22	52	23	46
	40	91	31	8	24	11
MAGT1	85	397	104	336	208	300
	304	486	229	128	212	79
MAK	22	0	15	9	3	7
	1	4	15	0	7	0
MAK16	59	250	77	150	115	192
	202	325	119	101	174	36
MAL	4	7	7	4	2	2
	11	8	7	1	3	6
MAL2	11	1	2	0	5	0
	1	0	7	0	6	2
MALL	25	42	11	20	15	17
	17	55	33	16	23	3
MALSU1	58	219	28	238	139	204
	260	356	177	84	189	26
MALT1	137	394	113	230	253	306
	320	765	237	191	312	108
MAMDC2	56	147	47	151	69	137
	120	312	118	45	101	39
MAMDC4	6	15	5	4	9	9
	19	21	5	10	7	1
MAML1	60	274	71	185	134	208
	227	250	136	91	151	67
MAML2	52	147	44	90	50	64
	103	88	60	44	65	20
MAML3	41	68	41	58	26	65
	57	83	55	12	45	28
MAMLD1	21	30	25	24	22	15
	37	34	21	10	22	6
MAMSTR	22	137	20	42	41	98
	92	77	48	51	57	25
MAN1A1	81	319	82	233	171	338
	180	615	163	79	183	146
MAN1A2	177	748	153	591	388	625
	546	823	328	236	403	179
MAN1B1	30	118	26	96	43	85
	103	152	72	25	57	23
MAN1C1	24	57	17	52	33	54
	53	60	29	14	26	13
MAN2A1	141	570	181	486	357	382
	209	533	285	150	241	122
MAN2A2	254	986	165	919	399	823
	810	714	363	263	444	131
MAN2B1	19	80	23	70	33	70
	53	82	55	10	45	24
MAN2B2	26	64	22	51	30	39
	40	87	36	16	38	18
MAN2C1	67	206	55	131	97	114
	152	310	102	73	139	41
MANBA	72	126	60	87	83	120
	83	248	109	47	96	32

MANBAL	17	28	6	29	8	17
	29	21	8	5	14	3
MANEA	36	86	35	76	49	62
	46	109	59	22	60	23
MANEAL	12	12	10	10	9	13
	11	17	12	4	13	2
MANF	15	37	20	20	17	27
	31	40	17	16	29	10
MANSC1	15	14	7	9	7	9
	12	14	7	7	11	1
MANSC4	12	0	4	2	2	0
	0	1	2	0	4	2
MAOA	154	60	16	34	67	795
	88	1739	521	26	87	53
MAOB	107	546	58	255	202	205
	421	503	165	182	234	77
MAP1A	82	236	64	145	56	134
	79	157	91	45	64	41
MAP1B	173	705	201	674	284	515
	534	673	295	169	318	222
MAP1LC3A	12	55	14	26	20	27
	60	76	17	22	24	11
MAP1LC3B	124	1106	187	827	341	636
	1126	1092	348	178	471	141
MAP1LC3B2	10	4	4	5	5	0
	7	1	7	0	6	0
MAP1LC3C	5	5	2	2	0	0
	0	4	6	2	2	0
MAP1S	7	28	6	19	10	22
	26	30	11	10	17	2
MAP2	104	58	53	59	39	55
	68	94	105	33	67	36
MAP2K1	107	464	116	482	219	304
	388	456	220	145	262	81
MAP2K2	163	1011	169	579	344	738
	1116	1203	371	310	525	125
MAP2K3	53	217	37	201	64	81
	152	145	74	48	65	30
MAP2K4	126	560	111	422	282	352
	373	483	259	169	315	68
MAP2K5	36	128	31	98	56	94
	98	101	55	25	57	25
MAP2K6	147	699	111	364	324	479
	660	1103	267	218	374	153
MAP2K7	33	120	28	85	61	92
	127	133	50	56	74	34
MAP3K1	101	288	78	229	117	179
	166	257	119	73	152	60
MAP3K10	22	57	18	31	18	60
	41	44	30	15	33	12
MAP3K11	31	70	20	43	41	43
	60	49	37	19	43	20
MAP3K12	22	25	27	24	15	16
	21	38	7	7	12	10
MAP3K13	103	116	53	71	66	65
	81	64	73	33	60	12
MAP3K14	33	95	36	50	34	54
	59	45	29	17	32	15

MAP3K15	49	10	6	3	4	1
	10	6	15	2	9	7
MAP3K19	28	0	9	1	8	0
	0	0	14	0	7	0
MAP3K2	284	1155	297	942	596	857
	879	1405	594	432	757	288
MAP3K3	80	224	62	137	152	177
	176	317	120	93	155	67
MAP3K4	164	577	161	428	302	445
	403	655	275	224	339	129
MAP3K5	74	181	64	132	95	82
	149	195	117	49	117	23
MAP3K6	23	63	26	38	26	27
	38	52	26	19	25	19
MAP3K7	97	422	119	362	225	290
	342	519	249	121	271	87
MAP3K8	25	37	14	79	44	32
	46	67	40	23	26	18
MAP3K9	27	30	17	16	9	34
	13	21	29	6	15	5
MAP4	1018	6487	1041	4086	2595	4308
	3955	4608	1652	1754	2866	1225
MAP4K1	25	5	17	4	7	4
	5	6	8	1	15	0
MAP4K2	22	36	14	9	9	8
	12	30	12	6	12	7
MAP4K3	262	1098	250	805	528	804
	895	1553	676	378	865	237
MAP4K4	221	1030	197	583	382	539
	547	594	323	235	413	151
MAP4K5	105	408	95	294	199	323
	345	463	229	130	274	79
MAP6	14	7	17	8	10	9
	8	11	12	2	10	6
MAP6D1	34	169	26	137	74	99
	138	106	46	16	54	12
MAP7	32	23	32	26	13	26
	12	7	30	5	13	5
MAP7D1	170	844	149	648	392	649
	566	621	302	326	467	144
MAP7D2	27	4	7	2	14	6
	2	2	2	8	6	0
MAP7D3	72	265	61	149	94	256
	166	269	83	99	124	46
MAP9	41	27	30	35	20	15
	17	20	44	14	22	5
MAPK1	216	1150	294	882	459	843
	893	981	569	257	543	172
MAPK10	51	31	34	46	22	22
	28	11	25	9	35	4
MAPK11	6	16	5	6	11	5
	9	16	9	6	7	1
MAPK12	207	1241	202	725	510	972
	492	1055	547	252	673	148
MAPK13	45	11	15	3	7	7
	19	18	14	3	11	3
MAPK14	115	548	152	386	308	364
	387	606	273	134	349	89

MAPK15	1	0	3	0	0	0
	0	1	1	0	4	0
MAPK1IP1L	123	592	114	363	266	347
	426	496	207	144	282	84
MAPK3	23	48	24	36	38	47
	55	53	46	21	41	18
MAPK4	22	14	24	23	10	11
	9	14	16	5	9	5
MAPK6	198	996	226	1059	478	1046
	778	888	500	245	595	185
MAPK7	18	16	11	8	8	7
	8	15	15	2	13	3
MAPK8	69	260	53	195	119	168
	217	271	126	79	156	33
MAPK8IP1	17	9	11	6	6	5
	1	3	5	2	6	4
MAPK8IP2	7	2	8	0	2	2
	2	3	3	0	6	0
MAPK8IP3	55	171	54	112	57	101
	116	198	104	63	97	34
MAPK9	88	414	78	315	160	296
	372	336	170	118	226	82
MAPKAP1	158	889	148	537	363	677
	763	772	364	235	431	129
MAPKAPK2	138	843	130	624	310	514
	664	618	295	190	372	135
MAPKAPK3	110	544	130	462	248	528
	640	504	371	194	348	107
MAPKAPK5	78	248	73	185	104	236
	201	320	156	84	172	55
MAPKBP1	68	171	87	96	71	113
	110	122	82	41	81	25
MAPRE1	69	367	96	295	182	281
	328	376	149	85	186	70
MAPRE2	176	704	121	815	368	485
	546	678	342	162	316	86
MAPRE3	36	229	43	184	79	120
	188	145	75	49	75	21
MAPT	168	628	113	417	310	480
	419	512	223	249	298	132
MARC1	32	41	31	68	46	31
	57	64	71	19	37	18
MARC2	45	169	42	109	83	119
	186	187	128	51	99	36
MARCH1	29	25	17	26	27	35
	16	34	36	7	20	4
MARCH10	27	0	7	0	3	0
	0	0	12	0	7	1
MARCH11	9	0	5	1	3	1
	0	0	3	0	2	2
MARCH2	25	127	26	81	48	103
	100	136	81	27	74	20
MARCH3	19	47	15	50	26	39
	84	35	34	7	30	4
MARCH4	17	0	12	0	1	1
	0	0	10	0	0	0
MARCH5	49	245	55	221	125	188
	215	295	129	68	129	48

MARCH6	517	2703	553	2683	1376	2019
	2226	2653	1094	714	1232	465
MARCH7	307	1630	315	1174	738	1030
	1251	1502	669	385	842	241
MARCH8	35	184	45	80	70	119
	144	132	73	50	90	36
MARCH9	25	35	9	13	10	22
	22	23	12	4	10	6
MARCKS	98	501	112	592	283	396
	378	500	255	131	235	80
MARCKSL1	4	30	4	7	6	9
	14	17	3	7	3	3
MARCO	13	0	12	5	7	12
	0	23	9	0	4	2
MARK1	49	65	24	57	53	60
	64	87	63	18	45	13
MARK2	40	145	29	92	61	97
	106	110	66	30	57	23
MARK3	222	1008	238	743	540	816
	730	981	417	383	589	203
MARK4	40	71	16	37	23	41
	54	49	18	18	35	14
MARS	92	448	66	282	156	342
	308	484	194	115	199	72
MARS2	22	52	15	38	28	46
	56	60	34	11	46	9
MARVELD1	16	43	15	22	24	25
	23	40	20	8	15	10
MARVELD2	11	6	2	1	6	3
	0	0	3	2	4	0
MARVELD3	17	2	9	1	4	0
	0	0	7	1	1	0
MAS1	4	0	0	0	1	0
	0	1	2	0	1	1
MAS1L	5	0	5	0	0	4
	0	3	3	1	2	0
MASP1	87	109	64	69	106	129
	107	83	76	28	60	34
MASP2	16	9	8	11	7	14
	2	22	9	2	7	2
MAST1	17	2	8	0	4	0
	0	0	8	0	2	0
MAST2	277	1110	236	771	658	849
	716	587	336	368	396	173
MAST3	31	69	21	33	20	20
	11	20	16	6	11	3
MAST4	137	511	165	253	85	213
	305	319	199	129	202	74
MASTL	36	60	26	55	42	48
	40	77	33	13	43	9
MAT1A	22	0	10	0	6	1
	0	0	8	0	5	0
MAT2A	282	1428	297	919	785	1141
	1432	1789	651	538	900	309
MAT2B	90	480	100	367	256	390
	489	649	277	159	404	99
MATK	12	2	7	1	8	2
	0	4	5	1	9	0

MATN1	19	0	14	0	2	0
	0	0	6	0	4	0
MATN2	75	100	41	196	50	30
	88	71	37	37	40	26
MATN3	18	1	8	0	4	0
	0	1	10	0	2	0
MATN4	8	0	1	2	1	0
	0	0	5	0	0	0
MATR3	635	3298	858	2307	1653	2429
	2458	4232	1511	1222	2169	746
MAU2	117	461	112	294	174	309
	426	508	254	173	280	87
MAVS	299	1207	220	665	521	660
	742	1521	521	409	718	313
MAX	58	267	75	160	134	269
	331	341	142	108	189	60
MAZ	66	170	38	130	76	150
	138	189	75	55	82	34
MB	12416	46160	5931	31474	20074	26399
	45827	45547	20305	10693	27353	6505
MB21D1	9	5	4	2	3	8
	6	8	5	1	10	3
MB21D2	18	25	17	39	16	26
	23	51	34	14	18	7
MBD1	76	245	70	167	102	166
	163	316	115	87	153	63
MBD2	98	535	111	403	180	419
	414	430	186	165	239	82
MBD3	34	169	27	99	79	80
	73	147	64	45	65	26
MBD3L1	1	0	2	0	0	0
	0	0	1	0	2	0
MBD3L2	2	0	2	0	3	0
	0	0	1	0	0	0
MBD3L3	2	0	1	0	1	0
	0	0	0	0	1	0
MBD3L4	2	0	0	0	0	0
	0	0	0	0	0	0
MBD3L5	0	0	0	0	1	0
	0	0	4	0	0	0
MBD4	64	231	50	145	127	211
	194	248	129	106	143	52
MBD5	83	269	72	189	133	158
	187	180	113	53	133	42
MBD6	27	90	12	51	37	26
	47	63	19	19	36	18
MBIP	34	75	30	69	49	36
	72	69	53	19	55	13
MBL2	9	0	5	0	3	0
	0	0	3	0	3	0
MBLAC1	1	6	1	1	3	2
	3	10	6	3	8	1
MBLAC2	33	101	41	81	65	88
	80	78	41	18	41	9
MBNL1	1755	7669	1799	6860	4568	6143
	7652	13080	4417	2889	6211	1686
MBNL2	405	1901	343	1325	976	1311
	1261	2314	909	473	1094	375

MBNL3	66	166	28	94	68	109
	41	99	36	14	49	19
MBOAT1	34	37	23	26	26	13
	21	52	25	11	24	13
MBOAT2	29	35	20	28	15	33
	32	26	22	6	19	8
MBOAT4	4	0	1	0	2	0
	0	0	2	1	0	0
MBOAT7	10	21	11	11	11	14
	24	23	13	4	7	5
MBP	225	951	176	508	414	669
	928	1220	543	297	679	193
MBTD1	108	363	83	275	153	213
	324	520	221	129	298	79
MBTPS1	103	352	88	305	163	202
	284	486	200	127	254	90
MBTPS2	51	263	46	203	109	105
	196	248	91	67	114	48
MC1R	9	3	2	0	2	3
	1	2	5	2	4	0
MC2R	16	0	8	0	4	0
	0	0	9	0	0	0
MC3R	1	0	0	0	2	0
	0	0	1	0	1	0
MC4R	11	0	1	0	0	0
	0	0	6	0	4	0
MC5R	6	0	2	0	0	0
	0	0	1	0	0	0
MCAM	37	219	66	123	77	91
	125	185	72	55	82	93
MCAT	17	32	10	29	15	32
	16	49	17	14	17	9
MCC	129	474	125	659	331	316
	291	289	169	125	158	46
MCCC1	93	403	76	300	169	282
	360	566	216	118	299	86
MCCC2	131	689	126	328	259	265
	558	676	239	150	306	116
MCCD1	3	0	2	2	2	3
	0	0	2	1	1	0
MCEE	56	201	40	145	65	118
	187	337	88	68	135	32
MCF2	43	2	13	6	20	2
	1	1	15	0	8	0
MCF2L	75	185	37	96	81	77
	181	236	100	62	79	31
MCF2L2	56	27	14	19	19	31
	13	14	30	16	36	4
MCFD2	206	780	189	668	454	668
	776	1068	359	242	502	193
MCHR1	6	0	9	0	4	2
	0	2	4	0	3	0
MCHR2	19	0	8	0	1	0
	0	0	9	0	6	0
MCIN	9	0	6	0	2	0
	0	0	2	0	3	0
MCL1	537	2290	385	1580	1189	1389
	2067	3744	1314	802	1644	466

MCM10	44	4	21	7	17	5
	2	9	12	2	16	1
MCM2	36	57	38	58	28	35
	61	82	51	24	56	6
MCM3	54	153	55	129	78	110
	135	192	86	44	106	28
MCM3AP	182	681	174	414	301	429
	553	632	308	198	340	118
MCM4	79	243	78	219	128	131
	235	265	127	102	204	47
MCM5	24	56	18	47	20	27
	38	83	44	13	40	8
MCM6	52	146	44	112	79	96
	93	124	68	34	70	19
MCM7	78	177	60	147	111	151
	149	291	98	75	135	37
MCM8	64	177	58	137	127	175
	177	230	135	96	166	44
MCM9	60	144	46	117	77	79
	75	142	75	28	89	31
MCMBP	87	292	73	225	172	235
	248	343	192	96	181	54
MCMDC2	41	72	30	30	27	46
	39	74	28	9	36	14
MCOLN1	16	29	13	22	14	9
	16	28	12	12	19	8
MCOLN2	25	1	16	1	4	1
	0	6	14	3	8	1
MCOLN3	33	19	23	10	4	3
	2	15	13	1	12	4
MCPH1	77	255	75	188	176	250
	251	356	172	112	213	53
MCRS1	51	199	41	156	93	151
	216	243	119	61	148	39
MCTP1	55	120	45	84	52	57
	73	87	63	29	66	38
MCTP2	74	14	54	5	22	6
	14	8	45	4	29	5
MCTS1	96	333	82	264	160	250
	236	428	182	88	218	66
MCU	238	1006	253	570	484	655
	666	1527	432	375	585	176
MDC1	68	232	65	123	94	123
	153	166	79	46	102	47
MDFI	11	5	2	3	2	0
	2	2	0	0	1	1
MDFIC	198	967	135	859	468	828
	703	827	474	150	550	139
MDGA1	44	18	28	12	10	11
	12	15	17	1	35	3
MDGA2	34	0	33	0	10	0
	0	0	22	0	15	0
MDH1	499	2686	330	1546	1024	1331
	2446	3725	1318	641	1629	479
MDH1B	13	7	10	16	4	15
	15	17	12	1	14	3
MDH2	296	1712	208	1340	709	1199
	2509	1807	961	564	1145	322

MDK	6	8	6	5	4	10
	5	5	4	0	9	1
MDM1	48	110	39	65	45	68
	88	125	72	41	66	21
MDM2	292	1907	755	1579	673	1283
	1020	1307	680	371	807	378
MDM4	279	1167	328	812	539	907
	823	1508	632	453	787	264
MDN1	563	2014	646	1351	1013	1434
	1419	2136	1086	683	1207	427
MDP1	3	25	3	32	4	14
	16	27	12	4	9	6
ME1	167	675	144	723	314	610
	594	542	240	139	229	75
ME2	137	857	167	439	284	367
	465	575	269	157	361	87
ME3	46	99	33	74	37	60
	84	112	67	41	50	23
MEA1	19	86	20	41	36	58
	81	112	27	27	32	22
MEAF6	88	293	83	228	166	299
	228	355	201	94	187	51
MECOM	93	187	72	119	86	112
	152	176	128	47	108	22
MECP2	107	433	89	302	223	307
	338	436	184	136	258	90
MECR	19	89	17	79	39	60
	81	118	64	29	60	20
MED1	168	565	150	319	275	322
	472	529	185	170	285	126
MED10	7	55	9	31	19	39
	43	43	22	21	34	12
MED11	16	62	10	44	31	52
	47	103	38	14	28	10
MED12	141	388	104	307	222	291
	298	448	177	143	280	77
MED12L	88	47	67	82	39	181
	7	21	68	4	45	1
MED13	596	2576	588	2029	1349	1801
	2374	3118	1206	888	1834	622
MED13L	441	1913	482	1532	1016	1446
	1620	2107	1054	726	1271	422
MED14	306	1416	317	1263	687	1329
	1244	1429	849	370	1016	216
MED15	56	107	27	59	59	90
	83	121	52	42	69	25
MED16	16	60	16	40	32	36
	54	69	28	22	21	13
MED17	52	168	53	100	84	98
	111	173	84	59	100	28
MED18	8	23	9	16	26	27
	28	45	31	9	34	7
MED19	19	38	10	31	23	31
	29	41	15	16	25	8
MED20	39	119	16	58	59	83
	95	123	56	33	80	19
MED21	59	194	57	193	132	174
	185	375	141	95	139	43

MED22	24	71	24	48	36	70
	41	92	39	14	35	25
MED23	131	440	128	397	251	419
	364	636	289	161	376	94
MED24	105	406	77	239	173	312
	339	505	213	128	244	85
MED25	26	78	17	41	33	57
	77	72	47	34	38	16
MED26	14	29	10	17	16	22
	40	18	21	11	22	4
MED27	12	51	5	40	29	31
	32	60	34	14	31	11
MED28	32	161	34	151	90	130
	179	186	94	53	104	31
MED29	38	133	21	92	44	85
	86	145	71	49	80	20
MED30	9	23	7	37	21	25
	33	70	25	11	33	8
MED31	18	52	14	37	28	31
	29	94	41	16	34	10
MED4	64	282	48	257	109	223
	230	279	140	61	169	36
MED6	62	318	102	221	187	315
	333	477	169	152	267	51
MED7	34	123	23	87	41	109
	107	122	50	36	66	25
MED8	28	126	30	104	46	85
	89	145	91	35	69	15
MED9	32	152	29	85	60	79
	118	194	46	40	85	16
MEDAG	29	61	27	92	47	59
	39	117	40	14	29	19
MEF2A	345	2342	519	2195	1116	1908
	1951	1962	815	615	1164	398
MEF2B	0	0	0	0	0	0
	2	0	1	0	1	0
MEF2BNB	0	3	0	8	2	4
	5	14	9	8	6	2
MEF2BNB-MEF2B		10	6	7	10	2
	5	0	2	5	1	3
	1					
MEF2C	1942	11378	1974	7541	4894	8959
	9115	12205	4829	3447	6060	1706
MEF2D	158	1283	208	761	421	656
	1159	951	357	300	620	199
MEFV	22	8	15	2	5	1
	3	2	11	1	5	0
MEGF10	127	387	185	277	202	197
	126	202	238	124	246	73
MEGF11	31	3	33	1	6	5
	1	8	23	0	9	0
MEGF6	26	62	36	45	32	43
	31	68	35	19	48	22
MEGF8	79	202	59	89	77	97
	114	171	61	39	103	36
MEGF9	72	259	57	203	140	136
	166	218	97	55	117	66

MEI1	31	3	17	1	5	1
	1	3	31	1	12	0
MEIG1	5	0	1	1	0	3
	0	0	2	0	0	0
MEIOB	13	0	4	0	4	0
	0	0	7	0	7	0
MEIS1	47	165	59	119	79	140
	128	207	127	58	125	25
MEIS2	30	21	19	15	20	14
	8	27	20	4	18	3
MEIS3	10	4	1	4	4	2
	2	0	1	1	1	0
MELK	35	4	19	0	2	1
	0	0	17	1	12	0
MEMO1	108	576	126	443	202	367
	498	682	273	118	288	82
MEN1	28	40	14	18	14	27
	25	26	14	8	11	3
MEOX1	21	82	17	35	25	34
	58	43	46	22	36	13
MEOX2	40	245	72	253	62	170
	161	172	123	80	78	38
MEP1A	20	0	11	0	3	0
	0	0	13	0	10	0
MEP1B	26	0	11	1	3	0
	0	0	10	0	5	1
MEPCE	38	113	33	114	68	129
	145	163	51	66	94	27
MEPE	12	0	15	0	9	1
	0	0	4	0	6	0
MERTK	50	34	23	11	29	23
	27	65	35	11	33	12
MESDC1	8	12	4	18	12	7
	6	20	9	3	4	6
MESDC2	66	202	51	143	89	120
	166	212	101	59	99	38
MESP1	3	4	1	11	2	4
	3	23	0	2	7	1
MESP2	4	0	2	0	0	0
	0	0	0	0	1	0
MEST	26	50	27	57	60	48
	30	45	50	11	30	14
MET	287	1038	242	796	602	663
	961	964	503	274	597	148
METAP1	53	349	59	223	148	200
	293	316	153	86	173	46
METAP1D	24	79	37	41	52	63
	97	146	48	32	58	17
METAP2	207	1152	270	726	527	896
	841	1049	438	357	644	173
METRN	4	15	0	7	3	4
	11	18	8	1	4	0
METRNL	3	7	2	9	9	10
	8	15	5	6	8	2
METTL1	19	26	11	7	12	12
	31	24	13	7	14	3
METTL10	46	267	57	138	104	146
	161	276	112	73	133	39

METTL11B	8	1	3	1	3	2
	1	0	4	1	2	0
METTL12	3	7	3	0	5	7
	6	14	6	0	1	2
METTL13	41	131	29	89	73	106
	99	118	82	41	83	25
METTL14	51	229	51	129	122	171
	182	243	120	81	140	46
METTL15	75	266	84	178	162	189
	215	359	172	65	196	45
METTL16	69	260	48	203	127	129
	142	223	118	71	142	44
METTL17	75	247	46	124	81	157
	157	289	126	68	121	33
METTL18	20	28	7	39	19	30
	25	66	23	10	32	13
METTL20	27	57	22	52	29	42
	53	102	39	26	50	7
METTL21A	35	42	16	25	16	30
	30	44	29	9	22	9
METTL21B	13	17	12	21	4	4
	11	12	7	3	9	2
METTL21C	17	6	9	2	9	14
	9	17	12	2	15	20
METTL21D	27	129	33	86	57	66
	66	119	51	33	58	20
METTL22	31	96	24	83	36	107
	156	135	59	34	78	24
METTL23	19	103	29	81	57	75
	144	199	62	34	78	19
METTL24	2	2	4	3	4	1
	0	5	4	1	5	1
METTL25	62	171	57	156	87	182
	172	189	101	54	123	27
METTL2A	37	129	31	57	65	80
	100	127	53	35	86	20
METTL2B	39	178	38	88	74	116
	109	181	52	43	98	22
METTL3	86	273	76	230	147	255
	235	453	169	94	211	58
METTL4	46	74	39	58	62	73
	67	89	67	17	65	18
METTL5	62	510	61	249	159	253
	461	441	169	117	221	66
METTL6	32	79	32	57	37	50
	63	107	55	23	46	18
METTL7A	190	1151	317	794	607	633
	465	2415	523	231	479	182
METTL7B	6	17	5	3	6	7
	10	18	10	4	7	2
METTL8	94	192	55	134	112	138
	147	168	103	60	125	25
METTL9	166	723	133	554	385	510
	668	925	406	248	391	109
MEX3A	25	4	14	2	3	1
	2	0	10	0	6	0
MEX3B	13	20	6	23	12	13
	29	19	10	5	7	6

MEX3C	71	268	58	165	110	193
	223	257	93	58	132	30
MEX3D	6	7	3	8	2	7
	1	6	8	0	3	2
MFAP1	52	155	51	62	99	159
	115	161	66	79	96	40
MFAP2	8	2	5	9	3	0
	3	6	3	1	1	3
MFAP3	98	383	70	335	200	308
	342	505	215	118	250	74
MFAP3L	69	206	56	36	14	305
	13	176	27	59	65	58
MFAP4	40	194	52	183	117	200
	107	232	67	34	108	68
MFAP5	135	387	144	745	359	638
	204	791	188	58	318	139
MFF	109	612	112	504	261	525
	547	706	304	186	400	83
MFGE8	63	284	55	168	154	224
	234	476	134	71	140	51
MFHAS1	32	37	22	11	29	27
	9	43	20	3	17	7
MFI2	20	7	17	3	5	10
	5	11	5	1	5	1
MFN1	146	680	126	462	295	437
	523	717	390	184	380	120
MFN2	520	2591	390	1561	1119	1379
	2484	2801	1018	876	1612	444
MFNG	9	19	6	6	1	8
	14	25	8	2	10	8
MFRP	6	9	4	4	4	11
	3	11	13	1	14	4
MFSD1	46	239	49	232	96	181
	194	323	130	64	160	38
MFSD10	13	15	7	21	7	22
	12	31	16	8	8	2
MFSD11	116	360	81	307	171	310
	276	318	191	79	218	66
MFSD12	11	22	7	10	4	14
	15	23	15	8	7	3
MFSD2A	10	0	12	1	2	0
	0	0	5	0	5	0
MFSD2B	2	1	4	1	1	1
	2	0	0	0	3	0
MFSD3	5	20	3	8	3	8
	10	14	10	5	10	3
MFSD4	18	13	10	4	8	7
	2	8	11	2	6	0
MFSD5	15	32	8	29	21	25
	31	55	19	13	16	3
MFSD6	45	110	40	95	78	84
	117	127	83	49	82	24
MFSD6L	13	3	5	1	1	0
	1	1	4	0	0	0
MFSD7	3	8	6	2	2	9
	6	7	8	0	4	1
MFSD8	77	255	58	198	127	176
	225	307	126	69	161	53

MFSD9	13	29	16	13	17	23
	8	39	22	7	15	7
MGA	276	1246	309	827	671	931
	900	1237	520	416	698	233
MGAM	68	6	68	2	19	1
	2	6	38	1	23	0
MGARP	6	3	8	3	5	6
	0	8	10	2	3	1
MGAT1	50	149	44	140	84	145
	149	185	96	63	120	25
MGAT2	13	86	16	52	39	46
	65	79	40	17	50	10
MGAT3	21	30	11	13	17	12
	11	17	5	4	16	2
MGAT4A	72	258	60	208	115	179
	206	296	132	60	135	34
MGAT4B	23	126	30	85	50	72
	101	114	49	25	39	23
MGAT4C	4	1	7	0	10	0
	0	0	6	0	8	1
MGAT5	72	89	50	80	57	71
	45	112	74	16	76	25
MGAT5B	16	1	12	0	5	0
	0	2	2	0	4	0
MGEA5	305	1613	283	934	728	971
	1074	1577	748	463	868	377
MGLL	107	386	65	261	205	290
	297	367	221	129	233	65
MGMT	21	72	11	44	16	52
	68	135	47	22	45	7
MGP	61	417	85	458	235	458
	320	860	188	106	228	181
MGRN1	53	171	40	107	61	110
	144	102	60	40	68	25
MGST1	25	25	12	15	30	21
	17	48	13	6	13	32
MGST2	39	190	33	105	56	118
	189	372	109	39	137	24
MGST3	313	1056	163	983	535	848
	1646	2396	799	361	801	227
MIA	2	0	8	0	1	0
	0	0	4	0	1	2
MIA2	12	1	5	1	7	0
	1	0	6	0	5	0
MIA3	179	974	239	559	466	613
	579	897	354	320	523	186
MIB1	204	847	263	717	453	707
	621	1048	481	243	534	177
MIB2	96	331	53	306	145	315
	331	393	179	123	238	61
MICA	19	64	13	16	28	28
	57	65	23	22	42	23
MICAL1	38	53	29	42	18	32
	26	39	27	18	21	5
MICAL2	57	131	55	65	59	89
	38	56	43	51	41	28
MICAL3	236	1016	222	505	444	642
	579	1129	455	233	523	170

MICALCL	31	7	7	1	3	2
	4	1	7	0	9	0
MICALL1	28	72	25	61	24	47
	70	68	30	19	44	14
MICALL2	6	5	9	9	1	10
	1	6	7	1	3	0
MICB	9	10	4	5	4	4
	9	13	1	1	6	1
MICU1	161	649	159	442	330	425
	526	881	365	273	416	152
MID1	50	38	24	61	26	22
	34	58	38	11	25	6
MID1IP1	150	442	71	308	359	371
	615	647	310	242	363	137
MID2	103	378	61	279	232	321
	300	362	144	109	151	59
MIDN	25	69	20	48	50	15
	114	94	25	31	41	21
MIEN1	6	18	6	31	14	40
	62	53	21	7	14	6
MIER1	72	432	105	339	222	322
	337	447	216	134	254	69
MIER2	13	54	8	24	20	22
	63	32	20	18	16	6
MIER3	102	353	101	324	194	334
	263	419	181	103	236	71
MIF	5	32	7	20	16	11
	17	32	15	3	16	6
MIF4GD	16	105	30	108	53	82
	73	121	44	28	45	14
MIIP	9	45	9	17	15	34
	9	69	37	11	19	12
MILR1	5	0	5	4	1	1
	5	3	4	0	1	1
MINA	86	371	88	265	188	323
	315	568	223	115	248	66
MINK1	54	168	40	106	61	87
	108	162	49	44	64	31
MINOS1	21	235	29	185	96	202
	342	333	114	49	135	29
MINPP1	30	69	21	80	47	60
	87	125	43	30	60	8
MIOS	99	631	131	428	309	392
	630	796	302	234	508	144
MIOX	1	0	1	0	0	0
	0	1	0	0	0	0
MIP	14	1	16	0	3	2
	0	0	9	0	3	0
MIPEP	37	111	23	66	55	55
	97	148	72	19	74	16
MIPOL1	78	181	80	144	81	131
	114	167	112	56	120	40
MIR205HG	8	1	0	0	2	2
	0	0	6	0	1	1
MIS12	33	116	33	117	77	114
	99	138	69	39	102	18
MIS18A	14	20	11	15	10	15
	16	18	10	6	10	3

MIS18BP1	33	122	48	78	42	87
	84	113	74	44	76	32
MITD1	23	82	17	60	46	58
	79	137	53	39	53	14
MITF	253	915	281	838	552	894
	867	1196	540	322	814	261
MIXL1	3	0	1	0	0	0
	0	0	0	0	0	0
MKI67	141	8	67	4	32	7
	9	11	77	1	38	1
MKI67IP	52	203	49	111	104	144
	178	281	114	62	124	39
MKKS	76	422	50	347	155	304
	390	354	206	106	215	64
MKL1	45	144	37	116	59	83
	71	125	59	23	54	22
MKL2	208	948	193	767	441	567
	795	737	423	186	479	171
MKLN1	337	1457	384	1162	742	1155
	1005	1945	788	479	903	319
MKNK1	48	146	40	78	90	118
	157	170	78	55	95	45
MKNK2	278	1347	138	933	616	947
	1007	1296	559	423	665	259
MKRN1	165	930	206	573	313	652
	571	811	365	249	478	143
MKRN2	71	351	68	294	279	248
	320	503	195	99	231	90
MKRN3	15	1	9	15	6	13
	7	0	6	1	2	2
MKS1	27	46	22	40	28	37
	41	39	31	20	35	5
MKX	13	3	15	2	5	0
	1	1	10	0	10	7
MLANA	9	2	4	0	3	0
	0	0	3	0	1	0
MLC1	9	0	10	0	1	0
	0	0	4	0	7	0
MLEC	296	1788	337	1755	776	1574
	1361	1601	1014	379	905	280
MLF1	272	1280	250	1474	876	1253
	806	725	665	255	841	38
MLF1IP	22	18	19	17	8	24
	23	22	17	7	11	3
MLF2	43	161	40	140	76	124
	143	143	78	58	101	34
MLH1	136	686	165	446	317	422
	490	755	322	193	450	109
MLH3	134	524	183	422	329	426
	410	591	248	181	336	97
MLIP	149	912	217	565	419	546
	811	1561	485	314	605	164
MLKL	26	36	19	11	9	19
	15	25	14	14	17	3
MLL	343	1284	367	900	636	982
	1075	1356	624	476	696	281
MLL2	209	1193	219	628	390	591
	673	724	272	277	380	185

MLL3	831	4509	1193	3314	2263	3232
	2941	4064	1674	1335	1946	849
MLL4	51	124	33	62	58	82
	99	85	52	46	59	33
MLL5	203	1324	315	841	578	1007
	870	1272	450	434	655	272
MLLT1	31	139	26	78	56	88
	98	115	53	44	51	34
MLLT10	135	629	169	409	241	403
	481	768	289	198	347	91
MLLT11	21	50	13	72	14	56
	20	20	25	14	16	9
MLLT3	62	189	47	115	84	133
	150	129	97	33	78	27
MLLT4	213	1192	201	786	356	523
	857	873	561	308	563	168
MLLT6	237	899	188	752	389	636
	774	867	416	268	540	191
MLN	4	0	2	0	0	0
	0	0	4	0	1	0
MLNR	6	1	3	0	0	0
	0	0	0	0	0	1
MLPH	38	31	19	10	21	11
	30	8	19	1	10	11
MLST8	36	157	23	106	67	110
	164	153	75	56	91	27
MLX	55	329	40	160	122	178
	341	321	135	96	148	63
MLXIP (NC_000012 122516651..122517172)				2	15	2
	5	6	2	16	15	10
	12	2	3			
MLXIP (NC_000012 122611799..122628989)				161	993	148
	725	331	487	801	527	243
	228	312	139			
MLXIPL	29	74	9	13	31	41
	50	115	28	33	36	19
MLYCD	81	354	64	189	218	208
	286	591	230	115	209	56
MMAA	92	196	80	152	99	129
	183	176	102	52	142	35
MMAB	50	216	40	112	87	145
	180	317	102	82	126	17
MMACHC	35	74	28	26	28	29
	64	66	44	25	52	15
MMADHC	285	1772	350	2060	697	1626
	1651	2370	1125	395	1258	225
MMD	31	64	28	42	52	27
	51	113	34	20	33	24
MMD2	6	0	1	0	0	0
	0	0	8	0	0	0
MME	203	872	199	708	337	750
	706	957	504	250	591	173
MMEL1	14	0	9	2	4	0
	1	1	11	2	5	0
MMGT1	48	291	38	222	131	191
	239	305	108	71	127	42
MMP1	13	1	11	0	7	3
	0	1	5	0	3	0

MMP10	19	0	10	0	2	0
	0	0	10	0	5	0
MMP11	3	2	2	3	1	2
	0	3	7	2	5	1
MMP12	25	0	6	0	3	0
	0	0	11	0	1	0
MMP13	29	0	12	0	6	0
	0	0	14	0	5	0
MMP14	28	62	31	74	25	49
	29	72	45	18	36	11
MMP15	20	118	19	76	28	74
	46	58	29	17	30	10
MMP16	26	23	28	22	36	22
	13	12	23	12	19	3
MMP17	5	3	6	4	1	0
	1	2	1	0	3	1
MMP19	23	10	13	6	8	8
	2	7	13	0	7	2
MMP2	62	375	81	514	130	195
	215	320	157	57	116	59
MMP20	19	0	6	0	1	0
	0	1	10	0	2	0
MMP21	8	2	6	2	3	0
	1	1	8	1	1	0
MMP23B	1	1	2	1	0	0
	0	0	0	0	2	0
MMP24	15	2	4	1	2	1
	0	1	12	0	5	1
MMP25	12	6	4	1	1	0
	3	2	5	0	1	0
MMP26	14	0	3	0	6	0
	0	0	3	0	2	0
MMP27	17	9	8	7	9	6
	7	10	22	2	14	1
MMP28	6	20	9	14	9	7
	20	17	17	3	5	4
MMP3	20	0	13	5	3	1
	1	2	13	0	3	0
MMP7	9	0	3	0	0	0
	0	0	8	0	2	0
MMP8	21	0	15	0	10	0
	0	0	13	0	4	0
MMP9	11	5	9	0	2	0
	0	1	7	2	1	0
MMRN1	31	30	36	41	27	17
	8	82	31	5	20	32
MMRN2	59	249	46	145	115	124
	239	266	138	108	154	45
MMS19	107	256	69	241	103	260
	248	373	131	79	174	64
MMS22L	46	60	28	36	50	42
	39	61	46	19	51	10
MN1	164	964	103	782	414	571
	964	723	219	241	372	114
MNAT1	185	698	163	479	390	467
	740	1266	422	231	459	145
MND1	10	10	4	1	7	2
	12	15	7	2	5	0

MNDA	10	48	12	22	8	27
	34	16	31	18	10	8
MNF1	69	321	32	118	115	189
	321	505	139	63	142	54
MNS1	31	12	13	7	13	16
	15	21	29	11	17	3
MNT	17	76	16	29	28	32
	33	44	19	19	18	6
MNX1	7	0	1	0	0	0
	0	0	4	0	0	0
MOAP1	85	358	79	278	160	317
	353	485	226	97	232	67
MOB1A	52	174	47	170	121	144
	117	197	81	47	137	27
MOB1B	115	629	116	522	273	354
	358	340	187	94	215	64
MOB2	40	125	28	108	66	99
	148	156	64	42	82	16
MOB3A	25	45	15	36	36	31
	36	57	21	13	34	11
MOB3B	37	53	31	59	32	39
	54	126	37	26	69	24
MOB3C	68	147	37	78	160	139
	186	288	70	71	125	70
MOB4	19	424	39	334	173	351
	404	519	175	109	295	73
MOBP	20	1	13	0	4	0
	0	0	4	0	3	0
MOCOS	12	6	8	3	5	5
	1	11	10	3	7	3
MOCS1	55	168	43	116	102	158
	176	275	122	71	110	48
MOCS2	95	485	96	365	192	372
	501	434	227	146	256	70
MOCS3	18	69	7	53	51	35
	58	77	39	12	31	13
MOG	21	2	3	0	2	0
	2	0	9	0	3	0
MOGAT1	8	6	6	7	7	9
	24	20	10	3	8	2
MOGAT2	7	0	5	0	2	0
	0	0	3	0	2	0
MOGAT3	7	0	5	0	0	0
	0	0	1	0	1	0
MOGS	28	63	20	48	36	43
	53	118	50	17	62	16
MOK	33	17	18	9	10	10
	15	19	12	10	14	4
MON1A	18	48	14	46	33	70
	39	80	37	15	28	9
MON1B	24	46	11	27	20	35
	31	45	30	12	24	7
MON2	199	832	219	702	435	598
	595	1022	443	301	520	178
MORC1	24	0	26	0	6	0
	0	0	34	0	13	0
MORC2	88	266	78	169	141	157
	178	231	93	57	121	37

MORC3	186	912	250	778	494	779
	760	1019	423	297	523	158
MORC4	56	273	89	195	109	168
	174	188	108	44	103	35
MORF4L1	261	1796	241	1189	581	960
	1352	1245	543	334	706	203
MORF4L2	247	1141	239	792	614	936
	853	1570	579	354	749	232
MORN1	12	26	7	14	5	12
	18	31	11	6	11	2
MORN2	10	58	4	18	17	43
	45	45	23	15	26	9
MORN3	5	0	5	0	2	0
	3	0	5	0	1	0
MORN4	17	8	10	9	3	8
	11	5	10	3	8	2
MORN5	6	0	4	0	1	0
	0	0	0	0	2	0
MOS	2	0	2	0	0	0
	0	0	2	0	1	0
MOSPD1	47	193	46	173	107	175
	184	242	120	68	147	40
MOSPD2	30	58	28	42	45	51
	44	53	42	31	31	8
MOSPD3	3	6	2	9	0	5
	5	10	7	1	9	1
MOV10	18	47	18	33	22	25
	38	55	38	14	36	3
MOV10L1	27	0	23	1	9	0
	1	1	19	1	12	1
MOXD1	29	1	11	16	10	4
	1	4	22	1	10	1
MPC1	117	767	119	490	277	444
	1116	1510	557	258	641	142
MPC2	185	776	125	473	296	375
	691	1095	300	225	435	141
MPDU1	14	30	9	21	20	29
	29	50	11	9	14	8
MPDZ	271	1319	266	819	751	973
	1024	1685	579	429	789	231
MPEG1	52	84	23	90	91	121
	70	198	81	46	92	29
MPG	19	51	9	30	20	40
	47	61	25	11	22	10
MPHOSPH10	87	345	73	201	191	264
	327	342	138	122	155	70
MPHOSPH6	20	122	19	68	30	61
	51	103	43	13	61	12
MPHOSPH8	89	365	85	241	161	275
	257	409	158	152	192	78
MPHOSPH9	116	436	104	239	232	264
	281	441	154	116	215	45
MPI	43	159	25	117	54	79
	136	170	73	35	87	30
MPL	21	2	7	0	4	0
	0	0	4	0	8	0
MPLKIP	9	46	9	25	15	26
	33	44	19	18	23	8

MPND	15	30	6	24	9	14
	29	48	12	7	16	6
MPO	19	3	16	5	3	7
	2	4	12	2	7	0
MPP1	46	182	29	106	55	132
	141	156	78	43	105	19
MPP2	12	4	17	3	3	4
	6	1	8	0	9	1
MPP3	20	9	15	5	10	4
	7	5	17	0	11	1
MPP4	20	2	11	5	5	3
	2	8	9	2	7	0
MPP5	147	805	163	659	384	507
	481	613	284	201	360	132
MPP6	69	226	70	190	128	151
	91	212	90	72	133	42
MPP7	100	417	66	192	170	213
	446	728	279	153	287	86
MPPE1	63	154	41	104	77	131
	94	183	90	52	100	51
MPPED1	12	0	8	0	1	0
	0	0	2	0	4	0
MPPED2	64	68	54	84	50	19
	32	140	44	29	47	20
MPRIP	273	1589	261	1060	695	869
	999	1130	520	516	590	313
MPST	12	75	19	36	32	37
	39	141	44	24	52	16
MPV17	34	74	24	30	28	46
	46	120	42	16	52	22
MPV17L	29	86	28	54	23	65
	63	85	23	26	53	13
MPV17L2	6	13	0	10	3	16
	10	13	9	3	6	2
MPZ	16	2	11	17	7	6
	6	9	7	7	3	37
MPZL1	47	211	64	211	99	202
	176	307	121	57	124	60
MPZL2	24	17	15	14	12	9
	11	22	22	5	8	2
MPZL3	9	4	5	1	1	3
	0	3	9	1	2	0
MR1	45	43	27	40	19	32
	27	47	41	10	25	9
MRAP	10	1	6	2	7	5
	3	3	6	1	1	1
MRAP2	20	2	8	9	7	10
	7	6	10	4	10	0
MRAS	158	718	180	578	274	593
	452	477	166	138	238	97
MRC1	24	44	12	79	21	58
	88	148	82	37	25	18
MRC2	109	293	59	315	166	218
	133	228	109	48	124	51
MRE11A	91	264	77	239	155	205
	267	290	145	105	205	74
MREG	18	18	13	21	5	7
	14	9	22	6	14	5

MRFAP1	147	941	133	589	458	763
	1193	643	371	268	464	134
MRFAP1L1	85	509	86	252	203	302
	329	495	168	117	234	69
MRGPRD	5	0	1	0	0	1
	0	0	3	0	1	0
MRGPRE	0	0	3	0	1	0
	0	0	1	0	0	0
MRGPRF	4	5	3	11	5	8
	2	6	5	0	2	0
MRGPRX1	1	0	5	0	0	0
	0	0	2	0	1	0
MRGPRX2	6	4	6	1	0	0
	0	3	1	0	2	0
MRGPRX3	18	1	13	0	6	5
	0	0	8	0	5	0
MRGPRX4	6	0	4	0	0	0
	0	0	3	0	0	0
MRI1	50	176	36	97	78	109
	111	92	54	58	76	22
MRM1	8	16	6	7	8	11
	11	19	11	6	13	2
MRO	44	20	26	27	30	26
	18	35	33	10	38	2
MRP63	53	207	40	140	72	166
	150	264	99	52	112	20
MRPL1	76	398	70	218	177	277
	489	590	191	134	255	65
MRPL10	45	140	23	99	61	126
	119	171	77	38	104	26
MRPL11	52	216	33	134	80	179
	258	366	107	37	130	36
MRPL12	5	110	8	55	25	40
	165	64	39	12	44	13
MRPL13	71	282	51	206	104	213
	284	483	187	101	205	37
MRPL14	32	135	24	146	77	107
	198	276	73	53	97	33
MRPL15	118	558	87	395	211	388
	726	811	339	200	423	100
MRPL16	62	333	52	223	158	243
	372	402	174	104	212	57
MRPL17	50	190	32	151	69	131
	168	289	103	58	103	23
MRPL18	34	313	39	201	135	210
	336	358	145	76	125	45
MRPL19	300	1039	246	906	759	918
	980	1735	568	407	730	270
MRPL2	35	157	24	81	58	113
	163	176	86	36	113	23
MRPL20	136	961	112	689	384	673
	1103	1563	552	226	531	117
MRPL21	36	193	24	162	68	127
	260	446	134	45	139	36
MRPL22	39	128	22	81	58	77
	123	152	100	43	64	23
MRPL23	47	226	57	219	78	196
	243	377	155	52	154	40

MRPL24	89	446	79	275	171	321
	473	653	202	134	222	56
MRPL27	38	210	40	129	83	112
	252	310	119	47	90	25
MRPL28	25	139	27	100	54	113
	155	175	67	34	74	15
MRPL3	168	775	177	519	334	547
	679	957	416	245	506	115
MRPL30	146	598	109	371	323	378
	470	727	239	228	354	114
MRPL32	55	238	31	151	101	164
	288	382	144	81	150	37
MRPL33	130	796	125	687	334	378
	1040	1338	414	209	475	125
MRPL34	58	232	23	128	128	164
	345	281	86	67	122	21
MRPL35	218	1034	171	633	458	691
	1066	1212	469	275	613	168
MRPL36	27	155	20	99	31	76
	156	161	68	37	74	15
MRPL37	35	297	45	198	108	178
	245	310	152	69	184	51
MRPL38	35	151	21	117	62	86
	139	162	58	55	57	27
MRPL39	57	368	68	253	184	251
	433	538	197	101	238	66
MRPL4	8	46	7	38	26	26
	51	39	22	13	39	11
MRPL40	80	331	51	186	109	203
	282	443	154	72	170	65
MRPL41	33	193	11	139	63	93
	206	358	82	40	107	24
MRPL42	100	389	85	289	206	363
	371	488	214	161	278	89
MRPL43	48	188	41	164	82	169
	240	249	115	65	111	29
MRPL44	82	314	50	213	123	222
	333	377	171	84	208	47
MRPL45	76	397	99	279	191	358
	447	562	205	141	262	82
MRPL46	58	341	47	201	122	258
	324	410	150	68	151	33
MRPL47	40	273	40	178	87	155
	244	320	137	62	124	34
MRPL48	40	121	12	73	92	96
	120	175	67	33	82	17
MRPL49	43	135	29	147	49	127
	128	162	101	44	76	30
MRPL50	50	180	38	132	108	126
	236	309	92	60	147	36
MRPL51	167	862	91	545	321	624
	948	1368	391	222	456	121
MRPL52	26	89	15	65	36	52
	115	156	74	18	49	18
MRPL53	30	187	21	103	65	77
	247	250	107	53	90	34
MRPL54	16	42	11	34	27	28
	61	114	32	22	40	7

MRPL55	34	71	8	51	24	43
	69	133	40	25	41	15
MRPL9	59	446	55	267	175	305
	400	550	190	127	235	67
MRPS10	177	954	175	726	294	743
	745	849	325	191	402	120
MRPS11	47	200	36	123	99	126
	242	246	95	51	114	27
MRPS12	8	36	5	18	11	18
	28	36	20	9	22	8
MRPS14	54	206	50	131	95	155
	167	238	99	45	105	34
MRPS15	129	957	96	637	324	712
	1197	1181	471	342	598	142
MRPS16	113	587	93	343	211	384
	443	720	286	145	329	89
MRPS17	34	136	23	99	76	105
	209	230	95	51	98	27
MRPS18A	61	222	32	124	91	118
	226	353	108	81	120	30
MRPS18B	88	538	75	321	217	294
	615	772	312	163	340	111
MRPS18C	52	203	33	179	88	161
	194	370	181	60	146	45
MRPS2	22	68	11	34	24	35
	65	95	36	19	50	13
MRPS21	50	199	29	125	83	123
	230	346	109	50	128	28
MRPS22	136	639	98	410	267	371
	667	777	298	175	441	131
MRPS23	49	226	52	174	88	147
	270	423	144	67	153	44
MRPS24	15	210	14	165	65	119
	271	319	95	55	123	22
MRPS25	179	673	172	433	265	463
	721	897	449	231	458	127
MRPS26	20	85	14	75	38	63
	121	114	47	21	40	11
MRPS27	74	450	77	301	193	277
	430	544	202	139	248	94
MRPS28	51	315	32	160	92	188
	321	454	165	86	204	32
MRPS30	75	416	56	269	204	236
	346	421	184	143	271	72
MRPS31	98	404	98	214	197	291
	350	500	186	104	245	61
MRPS33	68	312	32	211	99	189
	399	406	158	89	176	41
MRPS34	53	261	41	129	117	180
	295	376	103	91	128	30
MRPS35	132	775	136	532	287	538
	665	985	352	250	532	135
MRPS36	61	292	45	180	114	228
	314	554	186	88	231	45
MRPS5	100	459	83	215	223	307
	418	517	221	141	245	75
MRPS6	87	406	80	436	270	332
	287	286	79	57	80	22

MRPS7	105	673	72	398	260	349
	705	1004	315	163	409	91
MRPS9	134	687	125	366	243	418
	538	868	269	187	406	89
MRRF	54	296	63	208	110	197
	282	247	143	69	182	49
MRS2	160	681	135	624	334	465
	747	676	363	190	430	100
MRTO4	31	88	20	63	52	41
	88	142	51	22	69	21
MRVI1	49	129	33	85	52	63
	93	141	64	33	66	38
MS4A1	12	24	10	0	10	1
	4	0	15	0	7	1
MS4A10	21	0	4	0	1	0
	0	0	3	0	4	0
MS4A12	12	0	9	0	1	0
	0	0	7	0	2	0
MS4A13	7	0	8	0	2	0
	0	0	3	0	4	0
MS4A14	36	3	26	9	19	10
	6	11	23	3	15	0
MS4A15	5	0	5	0	2	0
	2	2	4	0	1	0
MS4A18	5	0	7	0	4	1
	1	0	6	0	2	0
MS4A2	50	15	20	45	21	49
	13	51	33	12	13	8
MS4A3	13	1	13	0	5	0
	0	0	20	0	5	0
MS4A4A	27	15	14	17	21	23
	16	56	24	2	37	6
MS4A4E	13	9	9	9	17	16
	4	31	18	5	8	5
MS4A5	4	0	0	0	0	0
	0	0	2	0	1	0
MS4A6A	52	43	23	46	61	84
	42	159	67	18	48	29
MS4A6E	3	0	2	0	0	0
	0	0	3	0	2	0
MS4A7	33	33	18	24	24	47
	19	64	32	9	29	15
MS4A8B	10	33	10	3	16	8
	2	30	8	10	10	1
MSANTD1	4	2	2	5	0	7
	4	6	1	1	2	1
MSANTD2	17	45	25	37	25	48
	34	75	22	20	35	10
MSANTD3	14	72	14	36	42	56
	55	76	36	21	48	5
MSANTD3-TMEFF1		12	57	23	33	40
	29	24	51	32	17	12
	17					
MSANTD4	74	243	60	260	106	169
	174	235	104	86	124	45
MSC	12	9	4	5	4	13
	3	7	12	4	9	0

MSGN1	2	0	0	0	0	0
	0	0	0	0	1	0
MSH2	45	171	68	143	87	145
	152	179	112	45	105	36
MSH3	93	319	64	226	108	231
	198	239	128	74	167	46
MSH4	35	41	21	34	33	27
	7	78	28	16	32	5
MSH5	25	27	20	17	17	14
	11	20	18	11	23	5
MSH6	65	278	82	205	167	222
	213	296	124	99	189	67
MSI1	15	0	8	0	2	0
	0	0	8	0	6	0
MSI2	94	371	104	271	198	297
	310	440	206	124	244	69
MSL1	103	435	85	372	218	341
	303	439	187	103	208	81
MSL2	73	494	125	342	227	321
	352	418	131	127	192	64
MSL3	90	253	58	188	131	269
	286	270	114	87	161	56
MSLN	10	0	4	0	2	0
	0	0	4	0	2	0
MSMB	3	0	2	0	1	0
	0	0	1	0	1	0
MSMO1	18	43	17	45	27	58
	44	57	37	13	37	7
MSMP	7	3	5	4	1	0
	2	3	1	1	1	0
MSN	117	511	105	400	245	358
	343	430	231	196	259	80
MSR1	46	31	24	30	40	78
	22	47	63	17	39	8
MSRA	21	61	13	30	17	42
	39	38	27	6	22	2
MSRB1	12	59	11	38	20	48
	41	67	28	17	36	4
MSRB2	61	269	39	155	92	109
	231	264	112	48	136	31
MSRB3	322	2012	306	1393	1053	1438
	1580	1457	593	476	804	289
MSS51	516	3002	312	2478	1390	2514
	2448	1727	1495	587	1116	234
MST1	14	23	29	13	12	7
	28	24	20	5	23	2
MST1R	16	3	11	4	7	2
	1	0	6	1	7	1
MST4	29	32	15	23	16	35
	12	28	13	7	13	1
MSTN	63	280	189	401	220	251
	195	123	122	47	83	32
MSTO1	35	48	20	28	20	44
	44	73	26	21	27	6
MSX1	7	20	5	12	5	14
	19	11	6	1	9	3
MSX2	10	11	11	11	6	8
	11	14	19	5	13	3

MT1A	0	0	0	1	1	1
	2	0	0	0	3	0
MT1B	2	0	0	0	0	0
	0	0	0	0	0	0
MT1E	15	22	2	19	20	9
	46	79	48	17	24	29
MT1F	1	1	3	0	0	2
	0	0	0	1	0	0
MT1G	3	0	0	0	0	0
	0	0	1	0	1	0
MT1H	0	0	1	0	0	0
	0	0	1	0	1	0
MT1M	1	3	0	2	3	5
	7	12	3	2	2	0
MT1X	52	147	36	128	88	180
	488	995	238	181	347	204
MT2A	27	93	14	49	31	54
	160	184	59	90	85	56
MT3	1	0	3	0	0	0
	3	2	0	0	0	0
MT4	2	0	1	0	1	0
	0	0	1	0	1	0
MTA1	48	162	31	82	63	97
	86	117	61	38	65	30
MTA2	47	142	44	107	74	103
	108	135	69	31	89	35
MTA3	51	85	29	76	49	72
	59	95	66	38	48	23
MTAP	77	246	73	156	123	244
	209	360	127	66	156	51
MTBP	41	45	39	43	29	40
	32	60	45	16	50	6
MTCH1	76	259	72	214	89	140
	211	325	152	56	176	48
MTCH2	142	687	96	528	212	510
	681	866	351	247	390	129
MTCP1	0	8	1	0	1	4
	1	1	3	1	0	0
MTDH	307	1747	377	1621	937	1672
	1166	1443	739	548	852	327
MTERF	25	98	19	54	50	42
	66	115	46	30	57	8
MTERFD1	33	196	27	73	96	105
	178	207	83	52	95	34
MTERFD2	54	174	44	102	87	145
	123	223	96	70	125	41
MTERFD3	35	200	56	163	101	182
	133	189	102	58	105	20
MTF1	54	169	55	88	109	107
	132	119	62	59	62	33
MTF2	80	295	103	233	142	211
	253	501	180	113	236	72
MTFMT	44	133	21	74	65	70
	104	166	65	36	95	24
MTFP1	4	13	2	5	2	5
	17	31	14	2	14	2
MTFR1	40	106	33	62	48	59
	74	122	48	40	73	19

MTG1	16	72	26	52	34	38
	43	86	30	25	34	9
MTHFD1	168	769	180	427	370	480
	634	772	311	255	442	136
MTHFD1L	46	12	25	10	12	13
	8	10	22	4	16	5
MTHFD2	44	212	112	251	107	244
	149	207	113	28	103	38
MTHFD2L	28	68	16	46	41	34
	65	116	35	15	52	15
MTHFR	55	285	83	146	103	177
	161	277	114	70	137	49
MTHFS	4	20	4	36	14	28
	34	21	16	6	22	5
MTHFSD	30	68	25	63	43	55
	65	79	47	24	45	11
MTIF2	126	760	123	422	285	420
	667	731	276	226	400	97
MTIF3	123	660	94	434	288	516
	771	784	296	202	390	95
MTL5	17	1	11	1	6	0
	0	1	9	2	17	0
MTM1	54	213	38	142	93	125
	125	218	75	47	108	35
MTMR1	35	177	43	106	85	125
	141	183	74	37	125	19
MTMR10	177	845	189	764	410	640
	721	1059	410	246	514	162
MTMR11	36	22	19	15	13	12
	23	26	16	3	19	4
MTMR12	80	171	54	117	111	93
	143	167	92	54	93	36
MTMR14	59	214	43	183	85	230
	248	348	186	94	176	53
MTMR2	64	201	50	130	81	121
	130	171	101	41	84	39
MTMR3	293	1255	322	942	588	833
	912	1120	627	336	716	227
MTMR4	79	264	69	151	114	92
	215	369	152	66	181	49
MTMR6	83	423	96	299	179	255
	326	448	196	129	279	58
MTMR7	32	3	23	2	17	9
	2	20	29	2	14	1
MTMR8	22	25	23	27	17	24
	27	20	24	5	21	8
MTMR9	96	375	108	264	173	299
	282	405	190	121	241	66
MTNR1A	3	0	1	0	2	0
	0	0	0	0	1	0
MTNR1B	1	0	4	0	1	0
	0	0	2	0	2	0
MTO1	69	272	66	220	156	200
	298	290	135	82	191	48
MTOR	227	972	218	530	472	700
	639	1168	458	311	543	207
MTPAP	123	547	140	366	302	341
	476	660	227	150	323	84

MTPN	54	399	65	386	243	495
	406	489	186	131	211	80
MTR	498	2087	562	1349	1154	1307
	1394	2312	918	650	1174	387
MTRF1	42	175	47	123	83	134
	171	161	96	44	116	32
MTRF1L	128	375	95	253	198	334
	374	342	199	124	240	82
MTRNR2L1	1662	922	1034	7442	993	4704
	2205	937	1712	5467	6810	4380
MTRNR2L10	43	11	37	509	25	104
	27	20	44	103	140	125
MTRNR2L2	2	5	0	2	3	2
	1	1	1	0	0	7
MTRNR2L3	60	41	47	214	56	101
	54	27	66	181	233	231
MTRNR2L4	4	3	2	10	3	6
	3	1	2	17	7	18
MTRNR2L5	4	1	3	0	0	0
	0	0	1	0	1	1
MTRNR2L6	33	58	36	1472	44	705
	308	138	130	531	507	668
MTRNR2L7	4	0	2	0	2	0
	0	0	4	0	2	0
MTRNR2L8	3895	1986	2808	27079	2666	9916
	7976	3195	5533	21262	21093	22013
MTRR	93	450	89	372	227	358
	405	682	203	138	296	92
MTSS1	155	703	196	435	269	465
	499	578	255	146	323	115
MTSS1L	40	214	44	117	84	120
	121	143	62	71	63	42
MTTP	39	9	35	10	10	8
	8	24	18	2	11	3
MTUS1	424	2591	475	1736	1213	1674
	2297	2629	968	787	1211	474
MTUS2	42	3	27	0	9	1
	1	1	23	1	18	0
MTX1	24	28	4	20	14	12
	23	52	18	11	29	8
MTX2	78	478	65	358	185	233
	443	536	210	122	269	48
MTX3	125	478	125	429	289	416
	361	597	278	164	321	90
MUC1	11	3	7	1	1	0
	0	0	4	0	3	0
MUC12	386	2	282	3	95	5
	2	1	190	0	143	0
MUC13	21	2	11	0	4	1
	0	2	12	0	4	0
MUC15	35	0	15	0	11	0
	0	0	11	0	4	0
MUC16	391	0	221	1	84	2
	0	4	199	0	72	0
MUC17	174	1	73	0	28	1
	1	3	61	0	35	0
MUC19	272	5	141	1	68	4
	1	3	130	3	67	3

MUC2	55	0	17	0	7	0
	0	0	15	0	12	0
MUC20	115	257	75	212	31	67
	262	154	68	55	127	32
MUC21	26	0	15	0	4	0
	0	0	8	0	10	0
MUC22	27	2	17	5	6	5
	3	3	16	3	13	0
MUC3A	432	37	275	15	150	5
	30	14	177	31	238	17
MUC4	158	2	83	0	37	4
	1	2	87	1	87	0
MUC5AC	7	0	4	0	0	0
	2	0	6	0	0	0
MUC5B	77	0	40	1	8	0
	0	1	39	0	18	0
MUC6	242	0	108	1	69	1
	0	0	139	0	162	0
MUC7	31	0	19	0	1	0
	0	0	10	1	5	0
MUCL1	4	0	2	0	1	0
	0	0	0	0	0	0
MUL1	53	182	43	137	86	140
	192	224	102	68	150	30
MUM1	37	109	47	82	46	86
	64	124	58	25	46	21
MUM1L1	129	534	101	317	253	328
	362	492	212	161	249	62
MURC	732	2756	1441	2263	1448	2878
	2559	2561	1926	1200	2027	844
MUS81	40	148	36	80	71	94
	85	178	68	54	79	25
MUSK	28	36	69	59	9	28
	26	17	31	6	20	4
MUSTN1	181	968	112	624	335	680
	1258	1030	638	372	595	150
MUT	101	601	107	395	204	316
	436	624	230	133	298	67
MUTYH	20	34	17	25	27	43
	24	44	40	12	25	12
MVD	15	62	13	30	22	45
	40	76	26	13	27	11
MVK	13	21	15	23	14	15
	28	29	13	6	15	6
MVP	37	143	48	111	52	100
	69	139	75	45	69	34
MX1	40	176	31	101	53	52
	53	145	69	184	119	15
MX2	34	42	14	35	19	24
	23	30	24	36	37	1
MXD1	72	220	65	133	67	126
	155	195	76	52	126	28
MXD3	10	6	10	6	2	14
	13	26	8	7	10	6
MXD4	41	114	21	75	75	90
	83	137	69	37	53	27
MXI1	107	297	64	173	171	257
	282	637	151	123	267	95

MXRA5	71	162	40	62	101	66
	60	69	54	16	55	24
MXRA7	75	342	45	185	174	249
	224	404	139	110	158	66
MXRA8	26	77	15	103	35	63
	42	107	37	15	35	28
MYADM	21	92	14	67	36	35
	54	110	31	22	36	16
MYADML2	73	275	35	271	120	266
	153	252	113	68	105	26
MYB	47	39	25	12	22	22
	24	25	34	3	26	5
MYBBP1A	60	250	95	258	122	413
	223	320	141	109	181	66
MYBL1	37	28	24	19	27	22
	29	42	21	9	32	5
MYBL2	15	1	15	1	3	1
	0	0	7	0	4	0
MYBPC1	33246	165683	27519	122123	90123	111005
	134588	148799	67022	46682	125500	30638
MYBPC2	1246	23902	4114	18871	7112	15116
	11811	6641	6991	3241	5649	1377
MYBPC3	18	4	23	1	4	0
	2	0	12	0	6	0
MYBPH	15	68	73	92	12	32
	9	8	18	5	5	7
MYBPHL	11	3	4	0	4	2
	1	0	4	1	1	0
MYC	10	33	11	20	17	29
	37	59	25	20	20	21
MYCBP	29	81	19	76	47	74
	71	120	49	26	63	15
MYCBP2	551	2184	717	1702	1280	1841
	1442	2306	1079	797	1337	461
MYCBPAP	39	11	24	11	5	9
	5	3	18	0	15	2
MYCL1	23	11	18	1	7	12
	4	2	7	1	3	0
MYCN	5	8	3	3	1	1
	0	3	6	2	5	0
MYCT1	47	203	15	94	77	93
	162	192	97	61	108	32
MYD88	14	9	7	19	10	14
	19	10	11	8	14	3
MYEF2	53	55	61	43	25	40
	42	50	73	22	54	12
MYEOV	8	0	4	0	3	0
	0	0	1	0	2	0
MYEOV2	33	112	15	77	44	58
	127	170	60	28	68	8
MYF5	9	24	5	12	14	27
	29	31	38	8	26	2
MYF6	526	1668	487	993	821	875
	2668	4484	1782	689	1614	274
MYH1	9723	228349	49283	225136	110061	153814
	106562	28625	30510	13573	29337	4088
MYH10	107	304	96	171	138	114
	176	205	112	52	145	72

MYH11	161	760	112	337	132	147
	164	559	107	113	192	621
MYH13	66	43	35	10	19	11
	12	1	32	1	15	1
MYH14	164	635	132	372	233	377
	382	667	171	291	290	157
MYH15	73	6	37	28	24	15
	8	14	36	10	34	3
MYH2	37510	314718	39668	271843	191892	374238
	320765	271767	237798	134382	293619	45814
MYH3	123	571	278	308	51	228
	114	87	66	75	79	29
MYH4	102	293	3767	249	121	203
	127	76	101	55	97	12
MYH6	158	361	91	219	234	223
	265	341	122	237	118	97
MYH7	43818	192620	28248	70585	98465	72476
	167547	196729	29102	80597	76542	45148
MYH7B	455	1319	434	467	457	776
	1183	2411	378	668	831	331
MYH8	74	1793	1802	303	174	418
	87	49	151	76	213	44
MYH9	251	1446	255	750	730	653
	919	1242	525	547	690	350
MYL1	13437	99814	11405	59178	37740	65760
	106598	77878	39872	16516	43515	9119
MYL10	8	1	5	0	1	0
	1	0	5	0	4	0
MYL12A	1584	10035	1508	3528	2793	3817
	15351	27271	5754	2680	4677	1487
MYL12B	163	690	102	478	235	481
	627	870	403	185	324	133
MYL2	3152	11914	1387	3529	5241	4233
	12155	16771	2910	3934	5908	2817
MYL3	1669	6055	764	2235	1996	2457
	6488	10895	1347	2146	2503	1730
MYL4	6	16	7	3	0	2
	3	2	5	0	4	0
MYL5	14	95	78	77	2	58
	133	11	14	4	18	6
MYL6	210	1198	179	956	490	1078
	1474	1168	570	317	451	176
MYL6B	277	795	224	514	110	536
	1518	1414	744	122	445	195
MYL7	5	0	1	0	1	0
	0	0	3	0	2	0
MYL9	18	119	9	48	33	28
	79	44	21	19	40	38
MYLIP	163	778	188	553	460	743
	748	1142	451	274	525	131
MYLK	79	327	107	174	114	106
	146	187	94	66	114	118
MYLK2	858	2357	270	2268	1412	2370
	2137	2046	1279	1785	1010	684
MYLK3	363	555	381	433	448	306
	545	1237	154	406	594	338
MYLK4	121	589	223	845	1028	1151
	1397	64	318	96	63	8

MYLPF	2245	16841	2501	20255	6334	13517
	21488	16039	12297	3308	10881	1799
MYNN	61	263	75	269	158	193
	197	301	158	99	217	67
MYO10	129	424	90	188	190	166
	274	223	219	145	187	60
MYO15A	52	4	26	5	10	5
	5	0	26	0	6	0
MYO16	54	3	33	5	19	3
	3	8	42	1	20	2
MYO18A	804	4844	929	1954	1743	3310
	2434	4118	1491	1697	1789	915
MYO18B	1124	5380	1146	3096	2483	4010
	2513	4988	2073	2330	2386	1017
MYO19	46	149	34	96	60	66
	122	201	79	37	114	19
MYO1A	39	6	19	2	7	1
	0	2	24	1	12	0
MYO1B	98	423	101	221	189	210
	322	378	204	125	191	59
MYO1C	93	373	116	238	215	156
	278	383	146	150	222	81
MYO1D	48	94	27	76	28	51
	41	98	57	23	48	16
MYO1E	68	289	71	174	147	199
	192	239	140	124	145	72
MYO1F	16	18	20	13	14	15
	14	19	9	3	15	6
MYO1G	17	11	13	5	3	5
	3	10	8	1	8	2
MYO1H	54	0	18	2	11	0
	1	2	21	0	7	0
MYO3A	49	11	34	10	16	1
	1	5	28	0	20	3
MYO3B	61	8	36	3	10	1
	4	6	40	2	14	0
MYO5A	180	588	205	523	245	510
	363	577	337	197	303	96
MYO5B	166	11	94	24	44	18
	11	23	68	0	47	4
MYO5C	82	140	70	88	58	59
	126	145	98	35	84	34
MYO6	107	268	84	252	120	221
	185	269	155	59	179	60
MYO7A	58	23	38	26	27	31
	24	53	43	9	39	11
MYO7B	45	6	37	7	13	9
	3	7	20	5	16	1
MYO9A	265	1188	366	848	629	822
	773	1313	614	394	650	276
MYO9B	74	162	51	79	61	82
	102	116	62	54	69	33
MYOC	71	152	79	467	101	510
	171	402	186	131	221	92
MYOCD	36	20	24	8	17	6
	2	17	20	4	15	7
MYOD1	15	107	17	70	35	81
	93	80	45	31	52	13

MYOF	119	313	104	241	126	131
	66	225	95	37	107	64
MYOG	41	263	74	219	191	216
	187	208	109	97	132	65
MYOM1	2760	16353	3251	7506	7387	7544
	10197	18348	4692	5848	8648	3679
MYOM2	1260	8491	2028	6512	3234	5944
	4618	6827	3880	1809	4564	1675
MYOM3	872	2755	677	2027	1258	2174
	1154	2876	734	1163	1372	724
MYOT	3882	21608	3030	11766	9257	13357
	17612	25722	7221	8730	13006	3900
MYOZ1	3805	20096	2976	13491	8582	14889
	17526	24461	9541	5327	11097	2535
MYOZ2	955	2831	539	806	1654	1089
	2606	8760	785	1876	1406	962
MYOZ3	216	1229	158	1074	555	900
	868	800	579	291	617	177
MYPN	1429	8439	1547	6612	4439	5846
	6483	9009	3528	3058	4687	1673
MYPOP	9	26	2	19	13	14
	45	17	10	8	12	3
MYRIP	37	43	31	94	56	13
	54	14	39	9	27	8
MYSM1	218	1021	253	780	495	793
	939	1332	464	372	732	199
MYT1	25	1	21	7	7	0
	3	0	14	0	8	0
MYT1L	47	0	18	0	10	5
	0	2	23	0	8	0
MYZAP	7	71	13	59	29	83
	31	22	23	66	21	16
MZB1	2	2	3	2	2	1
	1	4	1	4	5	0
MZF1	14	32	9	28	22	31
	23	44	27	10	34	9
MZT1	37	242	38	215	71	188
	192	242	112	51	137	29
MZT2A	7	17	2	13	2	11
	18	28	18	10	7	2
MZT2B	22	75	18	59	43	53
	134	132	56	20	38	18
N4BP1	66	301	82	236	147	279
	242	287	159	99	152	55
N4BP2	69	295	56	136	111	140
	115	246	134	52	111	67
N4BP2L1	33	78	28	49	34	64
	74	126	58	39	82	36
N4BP2L2	847	4214	959	2710	2286	3026
	3723	6405	2201	1781	3014	952
N4BP3	21	82	26	38	48	53
	54	62	40	18	28	11
N6AMT1	19	44	17	49	21	35
	47	36	36	18	46	10
N6AMT2	8	46	9	19	21	30
	22	45	30	10	22	6
NAA10	55	231	29	132	90	181
	254	249	112	79	121	49

NAA11	7	0	3	0	4	0	0	0	2	0	0	0
NAA15	195	1282	212	669	560	737	643	995	376	325	575	152
NAA16	97	336	133	262	218	233	284	514	222	133	292	83
NAA20	62	356	60	244	128	321	309	477	157	103	209	38
NAA25	195	770	175	560	357	512	636	1000	396	276	512	142
NAA30	172	953	172	828	481	755	817	971	458	245	571	177
NAA35	102	421	102	303	187	250	324	383	194	109	241	74
NAA38	209	536	150	397	288	446	464	754	330	166	346	142
NAA40	40	94	24	61	40	45	64	70	45	28	43	11
NAA50	547	3053	745	2999	1546	2253	2154	3069	1254	892	1946	529
NAA60	18	101	16	38	52	26	63	57	28	22	28	20
NAAA	35	46	23	34	32	31	24	49	31	12	29	6
NAALAD2	54	61	33	39	27	25	31	51	69	15	47	15
NAALADL1	14	6	10	2	3	2	4	2	2	2	5	1
NAALADL2	40	66	29	45	31	50	34	54	51	14	49	18
NAB1	89	275	85	212	144	166	189	250	99	62	132	44
NAB2	19	42	19	29	24	28	20	44	19	11	24	10
NABP1	29	74	33	48	52	40	40	108	48	20	55	11
NABP2	31	86	14	74	42	119	114	133	57	32	94	26
NACA	247	2721	342	1610	863	1519	2091	2150	628	649	910	385
NACA2	6	0	1	0	0	1	0	0	2	0	1	0
NACAD	13	5	13	5	3	1	4	3	8	0	4	1
NACC1	17	111	18	54	22	68	79	62	25	16	32	14
NACC2	36	190	42	172	111	141	105	198	79	48	89	34
NADK	44	106	25	58	51	77	89	143	54	30	69	21
NADKD1	102	450	77	320	258	260	317	486	202	134	255	84
NADSYN1	28	50	19	28	38	25	39	64	40	15	17	14
NAE1	101	375	104	277	185	300	308	545	235	131	290	69
NAF1	22	47	21	47	33	39	39	86	44	10	50	17

NAGA	46	88	13	50	41	79
	54	120	45	31	50	9
NAGK	35	57	21	48	36	51
	59	82	37	15	44	17
NAGLU	8	27	5	18	8	9
	22	27	20	11	7	5
NAGPA	16	21	12	16	18	18
	32	48	26	14	15	6
NAGS	2	0	1	2	2	0
	0	1	6	0	1	0
NAIF1	22	50	8	31	26	40
	49	49	23	14	32	6
NAIP	120	228	76	93	76	130
	70	147	103	36	108	37
NALCN	85	20	37	26	26	32
	16	12	44	3	39	1
NAMPT	157	911	246	933	441	650
	1306	1325	781	250	1148	211
NANOG	7	3	5	4	5	0
	2	1	10	0	6	0
NANOGNB	10	1	1	0	1	0
	0	0	3	0	1	0
NANOS1	42	354	82	490	124	215
	313	88	122	28	47	6
NANOS2	2	0	0	0	1	0
	0	0	0	0	0	0
NANOS3	1	2	1	0	0	0
	0	0	0	0	1	0
NANP	24	46	17	36	24	23
	33	36	28	14	27	10
NANS	18	57	18	29	37	61
	69	84	31	21	37	14
NAP1L1	318	1834	346	1202	797	1322
	1237	1566	642	588	778	299
NAP1L2	24	67	14	41	18	30
	27	25	17	11	32	6
NAP1L3	12	11	6	13	9	14
	6	7	3	2	12	3
NAP1L4	197	1118	216	682	522	668
	823	978	366	297	554	168
NAP1L5	15	18	19	17	7	16
	14	33	5	5	12	4
NAPA	56	360	65	216	138	211
	344	302	132	105	216	85
NAPB	68	255	79	180	104	211
	205	300	129	63	151	42
NAPEPLD	62	210	67	195	116	157
	168	196	82	62	85	31
NAPG	81	340	84	329	161	290
	310	454	199	110	260	66
NAPRT1	9	32	11	25	18	19
	17	48	30	9	16	3
NAPSA	11	2	14	0	1	0
	1	4	4	1	1	1
NARF	58	256	32	148	99	155
	203	196	129	67	144	42
NARFL	32	58	17	39	29	36
	43	102	38	21	44	11

NARG2	158	572	147	332	314	341
	370	824	282	190	382	120
NARR	0	0	0	0	1	0
	0	0	0	0	0	0
NARS	225	1405	185	844	511	1059
	1051	1431	583	424	926	206
NARS2	62	262	73	206	150	195
	219	329	137	83	159	43
NASP	91	309	83	143	165	228
	276	437	137	105	180	79
NAT1	23	9	16	9	8	5
	4	9	10	3	6	0
NAT10	73	249	71	147	113	168
	229	271	123	80	151	35
NAT14	7	3	2	7	3	6
	1	1	5	4	0	1
NAT16	12	0	6	0	0	0
	0	0	9	0	2	0
NAT2	8	0	7	0	1	0
	0	0	6	0	1	0
NAT6	0	1	0	5	3	1
	10	2	0	1	2	3
NAT8	7	0	0	0	1	1
	0	0	0	0	2	0
NAT8B	2	0	1	0	0	0
	0	0	2	0	0	0
NAT8L	17	37	8	17	25	29
	19	30	15	15	21	10
NAT9	21	63	29	54	22	57
	49	80	35	19	31	15
NAV1	171	404	104	277	167	282
	256	323	200	115	190	58
NAV2	106	140	108	74	56	101
	106	149	105	32	84	33
NAV3	87	153	73	196	80	183
	124	82	173	51	124	20
NBAS	357	1399	370	949	750	1004
	896	1641	648	483	932	258
NBEA	212	587	183	468	260	361
	498	694	357	221	361	117
NBEAL1	261	917	276	752	510	620
	707	1043	529	303	524	234
NBEAL2	40	92	42	52	43	44
	61	81	45	19	33	16
NBL1	1	11	6	9	5	9
	14	30	11	2	10	2
NBN	130	530	154	343	258	397
	379	570	228	207	365	125
NBPF1	277	614	273	309	149	549
	226	277	277	84	228	60
NBPF10	427	1078	470	946	537	922
	625	973	657	323	521	219
NBPF11	21	60	51	64	60	42
	71	92	75	63	47	20
NBPF12	32	79	49	41	31	33
	20	69	51	5	36	1
NBPF14	202	504	222	236	129	472
	229	353	208	99	239	73

NBPF15	130	358	146	289	135	239
	225	300	214	97	171	51
NBPF16	17	32	20	19	6	30
	7	21	15	4	6	5
NBPF24	67	111	50	94	58	118
	90	109	51	13	73	22
NBPF3	46	58	21	12	24	46
	13	26	26	5	27	14
NBPF4	32	0	17	0	11	2
	0	0	15	0	10	0
NBPF6	54	0	19	0	7	0
	0	0	9	0	4	0
NBPF7	27	1	9	1	6	2
	0	0	8	0	2	0
NBPF9	63	104	35	63	54	71
	40	94	75	18	53	16
NBR1	345	2018	454	1415	943	1279
	1355	1829	733	478	970	362
NCALD	63	152	62	127	120	144
	148	207	93	80	172	39
NCAM1	65	209	125	99	66	224
	102	106	108	35	49	22
NCAM2	58	53	39	55	36	26
	54	50	46	30	54	8
NCAN	17	0	13	0	9	0
	0	0	10	0	5	0
NCAPD2	77	122	46	121	90	133
	122	133	103	56	98	48
NCAPD3	67	125	68	87	73	123
	123	141	62	42	79	35
NCAPG	44	17	29	17	14	15
	2	22	39	2	19	1
NCAPG2	54	65	47	95	67	77
	71	98	67	45	87	17
NCAPH	29	6	18	2	13	1
	1	0	20	1	10	0
NCAPH2	30	59	26	47	40	47
	53	108	50	25	40	19
NCBP1	125	465	157	363	206	294
	338	567	205	149	256	81
NCBP2	79	322	74	314	168	312
	333	427	210	128	205	67
NCCRP1	10	0	2	0	1	0
	0	0	5	0	1	0
NCDN	25	52	18	26	30	37
	40	59	25	12	24	6
NCEH1	33	97	33	119	70	74
	90	176	138	42	77	27
NCF1	13	18	3	7	4	17
	7	20	4	3	9	0
NCF2	30	41	18	21	25	20
	23	56	30	11	32	8
NCF4	15	8	7	7	7	14
	0	20	11	1	8	6
NCK1	60	296	47	220	132	225
	196	236	128	97	150	32
NCK2	79	373	71	261	189	295
	290	415	136	122	151	69

NCKAP1	526	2476	517	2376	1521	1988
	1969	2507	1065	827	1294	494
NCKAP1L	71	34	29	29	36	32
	26	59	48	9	32	14
NCKAP5	56	21	31	15	23	7
	21	18	27	11	8	3
NCKAP5L	39	82	27	43	26	52
	41	58	37	15	33	14
NCKIPSD	21	34	22	25	18	20
	28	37	27	12	37	6
NCL	253	1671	283	1034	833	1236
	1280	1339	500	707	807	358
NCLN	19	46	15	43	24	43
	59	61	32	22	32	16
NCMAP	22	2	6	7	5	1
	1	1	5	2	8	5
NCOA1	344	1462	408	1016	713	967
	1071	1453	546	609	870	291
NCOA2	121	569	122	438	283	403
	422	513	271	150	280	105
NCOA3	215	1572	374	1322	728	1186
	1579	1435	790	432	1016	294
NCOA4	347	1781	279	1262	810	1204
	1379	2206	940	526	1215	335
NCOA5	49	133	58	121	69	154
	134	167	74	41	106	13
NCOA6	91	421	100	198	151	195
	326	311	123	117	187	76
NCOA7	85	165	56	137	93	104
	125	147	106	49	111	50
NCOR1	454	2106	465	1467	942	1481
	1498	1836	891	645	1035	435
NCOR2	147	900	174	376	320	378
	587	559	214	230	266	208
NCR1	7	0	3	0	2	0
	1	0	1	0	3	0
NCR2	4	0	5	0	2	0
	0	0	2	0	1	0
NCR3	5	0	2	0	0	0
	0	0	3	0	1	0
NCR3LG1	78	10	31	7	21	25
	15	18	29	4	31	3
NCS1	14	12	12	5	4	4
	2	0	10	2	4	5
NCSTN	65	203	68	149	109	165
	180	207	113	45	138	49
NDC80	28	5	9	10	12	1
	8	2	23	4	7	1
NDE1	28	61	22	46	25	30
	39	45	23	21	21	14
NDEL1	75	310	63	188	148	227
	205	345	150	104	192	60
NDFIP1	127	791	116	617	327	407
	788	770	334	151	398	91
NDFIP2	89	508	139	831	253	526
	427	621	289	88	320	73
NDN	17	57	12	100	31	70
	40	67	38	20	30	18

NDNF	53	144	42	106	80	71
	97	166	83	58	69	32
NDNL2	0	0	0	4	0	1
	1	2	0	4	0	1
NDOR1	20	37	10	26	11	5
	10	28	12	10	11	5
NDP	8	0	5	2	3	0
	1	2	3	1	0	0
NDRG1	56	169	38	192	84	109
	157	187	103	52	109	44
NDRG2	857	3534	732	3019	2286	2688
	2673	2129	1028	1063	1590	384
NDRG3	90	348	104	263	156	270
	220	310	142	83	174	43
NDRG4	25	71	13	66	14	32
	80	66	31	14	38	7
NDST1	97	389	68	315	171	243
	305	274	165	88	151	55
NDST2	32	80	24	80	44	73
	65	90	49	30	42	21
NDST3	36	0	28	1	16	0
	0	0	12	0	9	0
NDST4	24	0	22	0	12	1
	0	0	16	1	9	0
NDUFA1	247	1010	97	676	341	430
	1352	2144	542	226	619	106
NDUFA10	271	1240	221	758	671	844
	1402	1987	649	420	751	248
NDUFA11	36	177	25	116	56	104
	195	312	120	45	133	22
NDUFA12	263	1122	156	691	381	660
	1468	1949	591	318	653	156
NDUFA13	242	903	103	629	373	568
	1313	1558	488	245	591	145
NDUFA2	46	194	16	165	88	153
	248	417	120	54	133	23
NDUFA3	47	215	24	129	93	118
	309	369	89	69	113	28
NDUFA4	446	1665	240	1920	859	1548
	2551	4502	1588	539	1525	267
NDUFA4L2	6	27	9	10	10	17
	24	27	20	7	11	2
NDUFA5	601	2969	477	2031	1156	2247
	2931	3676	1333	734	1921	475
NDUFA6	244	1214	216	1196	662	923
	1989	2378	928	381	960	209
NDUFA7	111	476	82	440	219	376
	761	968	364	158	359	87
NDUFA8	86	400	47	368	187	283
	612	569	238	135	294	63
NDUFA9	163	1031	126	697	377	539
	942	1301	493	218	640	148
NDUFAB1	181	1041	117	810	387	542
	1603	1676	541	283	609	150
NDUFAF1	39	309	59	257	57	150
	292	266	171	62	136	59
NDUFAF2	58	237	39	149	81	200
	302	443	133	77	111	36

NDUFAF3	21	94	11	54	42	47
	78	142	46	24	52	12
NDUFAF4	84	383	64	234	192	244
	371	474	172	130	210	59
NDUFAF5	96	496	104	317	175	294
	426	421	191	104	245	52
NDUFAF6	36	221	41	157	78	123
	190	167	95	58	128	42
NDUFAF7	60	261	50	175	141	217
	284	285	143	94	166	47
NDUFB1	147	987	97	872	332	680
	1510	2274	688	231	708	168
NDUFB10	323	1704	239	1368	697	1340
	2960	2995	1026	612	1336	244
NDUFB11	115	542	56	438	190	281
	660	817	265	154	306	65
NDUFB2	201	1036	169	751	449	648
	1539	1906	548	222	617	128
NDUFB3	180	786	79	592	368	468
	725	1576	333	183	475	125
NDUFB4	110	422	67	356	221	304
	671	832	255	158	354	93
NDUFB5	229	1275	155	946	479	849
	1504	1954	623	329	668	165
NDUFB6	39	204	27	193	87	85
	247	309	131	48	132	32
NDUFB7	105	500	71	387	190	288
	738	947	301	132	346	70
NDUFB8	143	744	92	443	267	377
	1049	1145	378	193	477	126
NDUFB9	310	1603	201	1360	553	1063
	2389	2545	938	486	1238	234
NDUFC1	127	788	126	548	269	463
	809	741	353	168	433	103
NDUFC2	63	448	65	490	163	377
	486	731	268	143	350	121
NDUFC2-KCTD14	40	221	33	178	93	
	136	287	252	151	61	152
	20					
NDUFS1	982	6245	1036	4640	2553	3457
	6138	6887	2558	1871	4020	945
NDUFS2	412	2074	271	1545	833	1096
	2783	2752	1088	510	1230	289
NDUFS3	125	638	78	459	215	380
	730	740	311	145	367	84
NDUFS4	282	1306	220	832	545	828
	1627	2319	711	405	950	288
NDUFS5	268	1806	166	921	660	881
	2101	2576	639	438	652	234
NDUFS6	71	432	50	242	140	287
	693	544	183	103	253	66
NDUFS7	78	386	60	314	165	195
	494	600	214	108	306	53
NDUFS8	40	241	29	211	84	170
	260	358	160	79	167	36
NDUFV1	365	1672	235	1203	722	1055
	1794	2247	803	555	1122	284

NDUFV2	566	2470	402	2074	1081	1772
	3360	4042	1412	826	1854	393
NDUFV3	175	656	87	281	260	536
	628	628	310	200	482	136
NEB	118202	886081	162238	609661	367856	540575
	589789	688667	304303	311448	436105	177002
NEBL	67	77	47	54	49	27
	49	35	73	15	56	4
NECAB1	32	1	23	4	12	2
	2	2	19	2	17	2
NECAB2	10	0	8	0	3	0
	0	0	6	0	5	0
NECAB3	50	204	29	105	94	131
	177	248	109	65	99	33
NECAP1	58	164	49	134	78	134
	143	258	94	51	124	19
NECAP2	24	71	17	53	35	62
	54	107	47	16	41	12
NEDD1	200	543	224	576	342	898
	827	957	597	287	853	194
NEDD4	757	3198	592	2275	1385	2374
	2977	3922	2227	1009	2465	642
NEDD4L	234	728	233	444	302	523
	584	672	454	150	422	141
NEDD8	100	426	56	240	189	356
	601	742	210	131	277	70
NEDD8-MDP1	61	268	38	116	91	173
	320	410	77	61	138	29
NEDD9	63	190	41	176	101	117
	149	348	153	93	160	49
NEFH	25	2	13	0	2	1
	0	1	15	0	3	0
NEFL	14	0	10	2	4	0
	0	0	8	0	3	0
NEFM	29	6	9	8	9	4
	1	1	10	1	11	0
NEGR1	58	183	89	232	168	216
	110	358	94	57	97	50
NEIL1	12	22	6	10	9	15
	17	32	15	5	11	1
NEIL2	32	116	24	92	42	91
	101	104	67	25	64	10
NEIL3	17	9	14	1	7	3
	3	0	8	0	4	0
NEK1	137	463	141	375	213	321
	305	375	185	138	247	93
NEK10	136	317	144	355	344	241
	682	719	309	228	384	152
NEK11	37	78	28	49	43	61
	31	58	44	14	31	6
NEK2	26	1	12	0	5	0
	1	0	11	0	15	0
NEK3	34	55	24	45	31	29
	46	68	50	16	38	15
NEK4	66	163	40	121	85	126
	115	227	107	66	133	39
NEK5	40	2	22	1	6	1
	3	2	27	1	10	2

NEK6	28	75	13	41	50	68
	53	65	36	26	29	15
NEK7	326	1456	349	1239	944	1134
	1137	1887	823	530	909	320
NEK8	11	5	10	0	6	3
	1	3	8	2	4	0
NEK9	167	1071	140	779	412	542
	790	952	365	229	415	122
NELF	17	28	11	31	12	33
	26	52	24	11	24	14
NELL1	23	4	23	0	10	2
	4	1	25	0	8	0
NELL2	31	14	26	3	16	4
	13	1	21	1	11	0
NEMF	250	1159	202	815	607	994
	900	1177	421	426	602	240
NENF	57	197	40	183	68	132
	295	492	153	72	124	50
NEO1	258	1102	289	947	531	868
	644	670	392	277	421	160
NES	112	593	234	543	191	336
	360	536	182	194	259	99
NET1	103	374	79	292	179	246
	266	438	199	89	173	72
NETO1	27	0	24	0	12	0
	0	0	28	0	15	1
NETO2	33	55	32	41	27	43
	49	26	39	11	8	4
NEU1	23	74	31	60	41	78
	81	133	53	23	57	10
NEU2	2	0	1	0	1	0
	0	0	2	0	0	0
NEU3	139	247	68	275	190	226
	328	450	208	145	220	55
NEU4	3	3	7	0	1	0
	2	0	0	0	2	0
NEURL	93	320	62	240	152	257
	191	292	169	77	146	46
NEURL1B	72	209	47	180	104	101
	161	260	97	56	81	35
NEURL2	7	23	10	22	8	29
	31	32	19	9	26	4
NEURL4	47	174	46	89	82	126
	98	212	70	45	97	31
NEUROD1	12	0	7	0	3	0
	0	0	8	0	0	0
NEUROD2	7	0	2	0	2	0
	0	0	4	0	1	0
NEUROD4	18	0	22	0	9	0
	0	0	13	0	9	0
NEUROD6	11	0	13	0	0	0
	0	0	3	0	2	0
NEUROG1	6	0	3	0	1	0
	0	0	4	0	0	0
NEUROG2	7	0	0	0	2	0
	0	0	4	0	2	0
NEUROG3	1	0	1	0	0	0
	0	0	1	0	1	0

NEXN	778	4177	598	1996	2688	3568
	4318	6750	1667	2041	3451	1083
NF1	297	1025	285	818	522	730
	720	1151	511	338	631	223
NF2	123	552	107	410	239	366
	394	441	201	183	289	84
NFAM1	34	4	15	2	8	6
	6	6	12	2	5	0
NFASC	72	88	52	57	47	38
	59	77	64	34	68	20
NFAT5	324	1708	414	1350	840	1212
	1214	1300	585	434	641	303
NFATC1	63	208	52	154	107	111
	125	267	67	56	109	43
NFATC2	68	202	53	159	71	139
	151	207	99	72	84	35
NFATC2IP	53	184	37	150	108	164
	193	292	102	64	146	38
NFATC3	271	1087	244	1040	612	979
	889	1019	465	320	489	194
NFATC4	45	26	19	34	38	20
	16	56	28	11	34	7
NFE2	14	8	9	3	5	4
	2	0	4	2	5	0
NFE2L1	1357	7158	1024	5015	3343	5247
	5182	5892	2237	2141	3545	1367
NFE2L2	97	419	92	370	245	315
	337	481	201	129	224	87
NFE2L3	39	27	12	23	14	31
	16	46	26	10	26	7
NFE4	46	1	17	4	10	1
	0	0	17	0	9	0
NFIA	385	2122	351	1926	992	1609
	1943	1899	851	585	1072	384
NFIB	361	2264	375	1589	1087	1339
	1835	2584	1090	623	1235	478
NFIC	118	1047	125	468	339	418
	658	568	154	245	253	198
NFIL3	28	95	11	76	13	43
	57	88	33	17	44	11
NFIX	182	1889	266	922	514	834
	1112	917	333	441	483	274
NFKB1	108	355	110	244	183	262
	246	303	162	112	177	75
NFKB2	19	86	36	63	40	42
	32	42	42	20	31	16
NFKBIA	104	374	130	464	262	453
	571	869	484	116	401	85
NFKBIB	3	21	2	12	9	14
	5	25	9	7	16	6
NFKBID	5	3	3	6	5	2
	0	2	5	3	2	0
NFKBIE	8	16	11	9	7	10
	10	15	5	2	3	2
NFKBIL1	9	25	5	28	7	21
	26	43	17	9	18	6
NFKBIZ	58	119	43	117	55	81
	93	154	74	42	72	23

NFRKB	90	247	88	160	178	198
	199	273	168	99	149	65
NFS1	48	159	36	115	79	112
	159	162	73	30	81	31
NFU1	100	404	54	287	161	401
	474	591	233	110	271	65
NFX1	170	706	159	461	308	485
	650	813	341	258	407	132
NFXL1	53	113	44	79	67	80
	76	96	56	33	64	11
NFYA	115	301	110	194	135	166
	195	323	117	71	158	35
NFYB	85	428	88	357	207	354
	410	473	239	140	315	72
NFYC	91	418	88	271	195	270
	374	421	174	103	237	74
NGB	3	0	2	0	1	0
	0	0	2	0	2	0
NGDN	56	210	46	135	104	143
	205	290	144	66	131	40
NGEF	16	4	14	2	6	1
	4	1	12	1	6	0
NGF	4	0	0	0	1	0
	0	0	0	0	1	0
NGFR	16	9	8	75	6	2
	6	3	4	4	9	4
NGFRAP1	8	50	11	27	26	46
	33	62	20	8	28	4
NGLY1	119	591	121	458	292	505
	557	694	334	191	441	87
NGRN	515	2498	452	1985	1156	2079
	2404	2996	1229	742	1511	437
NHEJ1	38	97	34	74	71	129
	67	100	51	32	69	17
NHLH1	9	2	4	2	5	9
	0	1	3	0	6	1
NHLH2	23	5	13	1	2	2
	0	1	6	0	7	1
NHLRC1	15	29	8	20	13	25
	25	12	15	7	22	0
NHLRC2	176	798	200	629	406	596
	684	1155	415	256	513	168
NHLRC3	79	379	102	236	196	292
	318	461	159	109	211	53
NHLRC4	2	0	2	1	0	0
	0	1	2	0	1	0
NHP2	27	125	26	71	44	62
	156	181	61	28	66	15
NHP2L1	78	417	81	291	177	262
	451	579	218	116	257	70
NHS	62	26	15	56	15	17
	11	14	14	8	16	6
NHSL1	34	36	43	55	30	24
	29	68	36	15	42	18
NHSL2	34	34	11	39	15	15
	21	24	9	7	15	6
NICN1	29	110	20	66	27	68
	73	105	45	25	34	18

NID1	108	352	67	287	192	195
	314	318	130	84	183	60
NID2	77	258	77	186	108	178
	184	142	148	76	106	33
NIF3L1	52	197	45	160	120	162
	267	421	140	76	186	37
NIM1	20	8	12	0	9	9
	10	11	11	4	12	1
NIN	300	1091	358	643	1162	1945
	720	681	368	251	447	204
NINJ1	8	25	14	40	19	21
	27	30	22	13	11	16
NINJ2	9	13	3	7	7	18
	13	11	3	3	4	3
NINL	56	86	23	32	43	52
	41	133	65	23	63	22
NIP7	26	63	16	52	33	39
	63	86	40	26	44	10
NIPA1	45	87	30	60	50	71
	73	89	70	26	46	27
NIPA2	63	288	50	248	107	225
	236	307	170	76	171	50
NIPAL1	27	38	21	26	37	43
	27	36	22	9	14	7
NIPAL2	25	15	12	24	7	35
	32	33	14	4	28	7
NIPAL3	50	112	37	91	43	53
	115	93	71	35	65	13
NIPAL4	15	1	12	0	2	0
	1	0	6	0	5	0
NIPBL	243	1307	308	1001	631	869
	955	1304	523	412	657	269
NIPSNAP1	23	21	7	15	2	13
	21	24	12	3	10	6
NIPSNAP3A	18	114	21	108	25	87
	93	119	78	23	69	10
NIPSNAP3B	77	415	91	288	154	285
	368	574	272	56	282	75
NISCH	74	197	63	100	89	109
	128	244	79	49	108	59
NIT1	39	118	21	99	56	101
	70	155	64	30	51	20
NIT2	48	196	43	153	59	135
	211	265	124	54	147	28
NKAIN1	26	74	22	54	47	39
	42	31	23	13	30	5
NKAIN2	26	43	22	19	35	38
	46	61	34	22	36	12
NKAIN3	14	0	3	0	5	0
	2	0	10	1	7	0
NKAIN4	9	0	3	0	0	0
	0	0	2	0	1	0
NKAP	17	123	15	65	42	43
	79	94	45	23	48	19
NKAPL	18	60	11	38	27	23
	38	35	37	24	28	12
NKD1	37	11	24	9	10	4
	3	7	20	2	9	7

NKD2	4	3	10	0	0	0
	3	0	10	1	3	0
NKG7	3	1	4	1	0	2
	0	6	2	1	1	1
NKIRAS1	71	296	68	195	131	216
	219	296	105	84	123	37
NKIRAS2	45	199	33	117	78	172
	186	178	82	43	124	29
NKPD1	7	0	5	0	3	0
	0	1	2	0	1	2
NKRF	30	79	14	40	45	54
	52	85	39	17	38	11
NKTR	328	1564	442	1269	665	1123
	1303	1896	855	607	1126	383
NKX1-2	0	0	1	0	0	0
	0	0	0	0	0	0
NKX2-1	5	1	6	0	2	0
	0	0	1	0	5	0
NKX2-2	3	0	3	0	0	0
	0	0	1	0	1	0
NKX2-3	7	0	4	0	1	0
	0	0	0	0	0	0
NKX2-4	3	0	2	0	0	0
	0	0	0	0	0	0
NKX2-5	8	0	1	0	0	1
	0	0	0	0	0	0
NKX2-6	1	0	1	0	0	0
	0	0	0	0	0	0
NKX2-8	3	0	3	0	1	0
	0	0	2	0	2	0
NKX3-1	23	33	6	32	21	48
	28	29	24	7	22	3
NKX3-2	9	1	1	0	1	0
	0	1	3	0	0	0
NKX6-1	7	0	1	0	0	0
	0	0	5	0	1	0
NKX6-2	0	0	0	0	1	0
	0	0	0	0	0	0
NKX6-3	1	0	1	0	0	0
	0	0	1	0	0	0
NLE1	21	21	16	20	16	19
	17	41	17	4	14	2
NLGN1	31	11	21	23	16	8
	14	11	16	7	17	10
NLGN2	17	28	14	17	14	12
	23	30	11	5	22	5
NLGN3	26	6	4	4	6	2
	2	2	2	1	5	0
NLGN4X	28	6	9	10	11	16
	10	7	21	4	7	1
NLGN4Y	2	24	7	26	0	14
	33	23	15	0	23	7
NLK	43	195	52	152	104	109
	150	217	102	59	90	33
NLN	149	712	162	376	435	398
	514	563	239	187	261	106
NLRC3	32	34	11	9	15	23
	13	27	29	10	19	6

NLRC4	16	6	7	0	6	6
	2	5	5	1	8	0
NLRC5	61	114	45	69	26	46
	98	56	67	31	57	15
NLRP1	61	160	39	95	57	73
	117	129	70	31	70	30
NLRP10	4	0	3	0	4	0
	0	0	3	0	1	0
NLRP11	39	0	13	0	3	0
	0	0	16	0	7	0
NLRP12	32	12	19	6	7	3
	6	6	15	1	6	2
NLRP13	19	0	12	0	2	0
	0	0	13	0	4	0
NLRP14	30	4	20	0	10	2
	2	0	16	0	15	0
NLRP2	18	0	10	0	3	0
	0	0	10	1	3	0
NLRP3	31	14	13	18	12	11
	6	28	29	6	13	1
NLRP4	33	0	18	0	2	0
	0	0	16	0	9	0
NLRP5	38	0	13	0	3	0
	0	0	12	0	5	0
NLRP6	3	1	3	0	0	0
	0	1	3	0	0	0
NLRP7	18	0	8	0	5	0
	0	0	5	0	6	0
NLRP8	26	0	4	0	5	0
	0	0	7	0	2	0
NLRP9	26	1	10	0	6	0
	0	0	12	0	13	0
NLRX1	18	118	8	55	39	46
	70	91	48	22	43	14
NMB	7	3	0	0	1	3
	2	5	1	0	1	0
NMBR	10	0	2	1	1	0
	0	1	1	0	3	0
NMD3	118	391	129	306	230	391
	385	473	221	158	326	99
NME1	6	57	4	26	23	18
	23	57	14	13	19	11
NME1-NME2	65	136	36	89	44	57
	70	164	77	16	72	4
NME2	28	398	35	383	182	366
	1088	953	350	145	219	65
NME3	8	24	7	14	10	34
	26	49	12	7	14	3
NME4	13	69	7	40	40	39
	73	67	12	26	21	9
NME5	6	5	3	4	3	2
	4	13	2	1	2	2
NME6	19	42	9	27	12	34
	33	51	15	15	23	8
NME7	30	64	18	49	35	47
	59	49	41	16	29	8
NME8	14	1	17	0	5	0
	1	4	9	1	11	0

NME9	27	20	21	26	18	9
	4	5	12	2	12	3
NMI	7	22	9	16	11	27
	21	23	21	9	11	4
NMNAT1	80	413	76	332	209	354
	412	347	188	98	271	64
NMNAT2	49	22	28	21	6	5
	16	11	18	4	12	0
NMNAT3	42	106	32	80	36	59
	149	160	73	50	97	24
NMRAL1	5	7	2	13	4	12
	13	24	15	2	10	2
NMRK1	57	137	41	101	69	108
	160	263	99	59	123	31
NMRK2	60	426	33	156	21	108
	848	348	132	108	162	29
NMS	10	0	6	0	5	0
	0	1	6	0	2	0
NMT1	132	411	118	345	206	389
	391	583	233	147	266	95
NMT2	42	166	44	93	100	116
	121	198	80	81	82	35
NMU	11	0	4	0	1	2
	0	1	2	0	0	0
NMUR1	13	3	5	1	1	0
	0	0	3	0	1	0
NMUR2	7	0	13	2	5	2
	0	1	8	0	5	1
NNAT	7	5	6	4	5	3
	7	6	8	1	1	2
NNMT	12	31	44	44	23	63
	20	369	34	12	21	30
NNT	883	5202	721	4118	2708	4160
	5836	5541	2894	1755	3894	1032
NOA1	35	153	31	116	80	133
	124	197	83	42	80	26
NOB1	22	65	19	56	27	32
	71	62	30	15	37	8
NOBOX	14	0	9	0	1	0
	1	0	5	0	2	0
NOC2L	64	260	66	165	128	223
	171	359	117	99	170	45
NOC3L	110	383	116	277	215	337
	311	482	205	138	259	83
NOC4L	10	12	7	16	9	10
	23	22	20	10	17	6
NOD1	47	63	28	63	48	45
	84	81	52	36	51	10
NOD2	23	3	16	4	6	5
	2	3	15	3	8	0
NODAL	11	0	4	2	2	1
	1	0	7	1	4	0
NOG	5	19	2	18	8	16
	15	13	7	10	5	0
NOL10	85	294	94	192	126	243
	195	274	159	101	191	62
NOL11	102	384	107	304	219	346
	348	472	241	128	276	57

NOL12	16	42	16	25	27	32
	38	53	21	15	20	7
NOL3	59	227	51	135	106	152
	178	239	81	49	133	43
NOL4	32	0	21	1	13	0
	0	0	20	0	9	0
NOL6	44	150	50	81	76	100
	69	158	71	37	75	25
NOL7	32	208	42	112	77	126
	187	240	97	55	93	22
NOL8	88	287	78	195	133	178
	191	281	128	88	172	60
NOL9	85	361	67	220	154	195
	287	391	175	95	251	64
NOLC1	114	475	116	339	217	322
	379	478	181	172	288	93
NOM1	75	240	71	150	129	174
	134	285	121	72	126	45
NOMO1	179	876	161	784	395	845
	539	561	270	168	380	125
NOMO2	132	478	161	291	177	366
	310	369	235	124	192	73
NOMO3	89	282	83	87	173	286
	93	135	89	82	121	24
NONO	180	771	149	563	383	543
	611	853	325	300	435	172
NOP10	50	188	12	121	69	126
	194	354	121	46	127	40
NOP14	78	331	63	174	149	210
	244	279	149	133	159	65
NOP16	22	93	13	50	25	70
	106	81	43	21	38	13
NOP2	37	133	29	71	45	92
	80	124	56	28	56	20
NOP56	59	306	47	173	147	211
	235	362	125	75	170	43
NOP58	93	474	109	269	199	303
	308	461	167	139	278	103
NOP9	40	122	34	60	60	73
	54	89	49	35	69	15
NOS1	256	1241	204	745	550	767
	800	601	274	427	427	145
NOS1AP	39	8	31	7	12	5
	5	8	22	3	9	2
NOS2	32	13	17	7	6	1
	5	11	18	1	7	4
NOS3	42	41	8	19	8	10
	27	29	30	6	11	3
NOSIP	64	271	59	239	136	140
	289	336	176	97	169	53
NOSTRIN	31	76	29	35	59	54
	63	109	52	42	48	33
NOTCH1	32	151	26	53	47	69
	107	70	54	35	59	26
NOTCH2	186	516	167	582	313	457
	325	630	345	160	317	161
NOTCH2NL	130	391	162	342	186	277
	315	397	231	105	241	93

NOTCH3	76	431	50	210	141	130
	243	233	110	82	149	68
NOTCH4	66	204	41	95	61	81
	131	146	80	40	91	24
NOTO	7	0	2	0	0	0
	0	0	1	0	2	0
NOTUM	7	27	9	21	12	24
	21	18	20	9	36	3
NOV	33	61	17	60	47	129
	19	52	47	12	21	8
NOVA1	51	137	64	185	118	105
	68	229	59	28	85	41
NOVA2	4	14	3	5	0	4
	5	1	2	0	1	1
NOX1	20	0	7	1	3	1
	0	0	4	0	5	0
NOX3	19	0	6	0	7	0
	1	0	17	0	4	0
NOX4	48	24	27	26	32	15
	10	77	37	4	34	19
NOX5	23	0	14	3	6	3
	1	1	13	0	1	0
NOXA1	11	17	15	14	8	11
	17	34	21	8	10	6
NOXO1	2	0	1	1	0	1
	0	1	2	0	0	0
NOXRED1	11	5	9	2	3	3
	1	2	5	0	1	1
NPAP1	41	9	19	0	11	3
	2	7	26	3	22	0
NPAS1	10	1	7	0	2	0
	0	0	1	0	2	0
NPAS2	31	46	23	35	27	31
	33	49	28	8	29	3
NPAS3	41	24	23	14	26	16
	24	19	23	8	15	3
NPAS4	23	0	11	0	4	0
	0	0	8	0	4	0
NPAT	58	233	65	159	133	155
	196	287	104	94	137	56
NPB	0	1	0	0	0	0
	2	0	1	0	1	0
NPBWR1	4	0	1	0	1	0
	0	0	2	0	1	0
NPBWR2	1	0	0	0	0	0
	0	0	2	0	0	0
NPC1	52	83	41	76	61	98
	90	136	73	32	96	21
NPC1L1	23	0	14	1	2	0
	2	1	8	1	2	0
NPC2	15	51	11	47	23	61
	64	125	60	17	45	8
NPDC1	15	27	7	17	3	10
	19	21	12	8	14	5
NPEPL1	41	169	31	129	70	117
	182	154	88	39	88	38
NPEPPS	369	2186	377	1897	1058	1538
	1741	2111	759	606	911	317

NPFF	4	3	4	1	0	7
	2	3	2	2	4	0
NPFFR1	3	0	5	0	1	0
	0	0	1	0	0	0
NPFFR2	14	0	4	0	4	0
	0	0	6	0	4	0
NPHP1	201	1883	302	1632	1004	2167
	1038	1249	724	294	905	109
NPHP3	121	323	92	311	204	229
	312	500	188	154	248	107
NPHP4	32	13	27	16	14	16
	4	6	26	3	13	3
NPHS1	21	1	12	0	4	0
	0	0	14	0	6	1
NPHS2	14	0	7	0	5	0
	0	0	5	0	3	0
NPIP	172	657	131	349	275	501
	442	521	247	153	235	82
NPIPL2	38	27	19	0	6	23
	15	4	29	2	29	2
NPIPL3	28	172	43	91	90	114
	130	160	38	66	75	53
NPL	16	26	17	21	15	19
	24	59	38	14	25	12
NPLOC4	175	791	138	748	335	675
	692	653	320	182	410	120
NPM1	273	1717	328	977	761	1388
	1721	2538	902	662	1053	379
NPM2	5	1	5	0	1	0
	1	2	0	0	1	0
NPM3	12	63	12	29	10	29
	42	55	17	11	21	7
NPNT	60	130	74	86	105	50
	22	311	36	30	44	33
NPPA	5	0	1	0	1	0
	0	0	0	0	2	0
NPPB	0	0	2	0	0	0
	0	0	1	0	0	0
NPPC	3	1	0	0	0	2
	0	0	0	0	0	0
NPR1	36	113	29	70	50	32
	117	129	52	32	46	25
NPR2	48	97	33	91	63	58
	83	109	50	19	47	16
NPR3	38	37	47	37	27	51
	97	14	81	4	30	17
NPRL2	47	88	22	72	34	85
	78	145	70	30	76	23
NPRL3	23	81	26	45	30	38
	60	66	36	15	33	9
NPS	4	0	0	0	1	0
	0	0	0	0	0	0
NPSR1	17	0	6	0	3	0
	0	0	16	0	2	0
NPTN	109	442	79	348	187	278
	295	572	206	132	236	75
NPTX1	16	2	14	0	6	1
	15	43	6	7	5	10

NPTX2	16	6	6	4	2	1
	5	11	15	2	10	6
NPTXR	15	7	4	0	5	3
	3	0	9	1	3	1
NPVF	12	0	7	0	1	1
	0	0	5	0	2	0
NPY	8	0	6	0	0	0
	0	0	1	0	4	0
NPY1R	19	41	20	25	26	8
	28	52	21	11	24	30
NPY2R	13	0	15	0	3	0
	0	2	5	0	6	0
NPY5R	11	3	4	3	5	0
	3	3	2	0	8	2
NQO1	41	193	31	86	72	74
	139	135	42	32	33	27
NQO2	31	196	6	60	72	202
	174	301	68	51	129	21
NR0B1	5	0	3	0	1	0
	0	0	1	0	0	0
NR0B2	1	2	1	1	0	1
	0	0	1	0	1	0
NR1D1	46	324	53	177	122	235
	183	113	88	29	75	27
NR1D2	487	2398	524	2048	1395	2001
	2070	2914	1277	776	1745	381
NR1H2	72	303	53	209	145	240
	210	308	134	95	159	53
NR1H3	22	26	10	24	10	14
	20	43	20	12	19	12
NR1H4	18	1	11	0	3	0
	0	0	10	0	5	0
NR1I2	25	0	21	0	9	0
	0	0	13	0	8	0
NR1I3	12	3	2	1	3	1
	0	2	3	0	0	0
NR2C1	149	474	124	497	251	449
	454	709	302	171	353	107
NR2C2	147	559	172	522	309	420
	483	657	326	183	380	146
NR2C2AP	10	12	4	17	4	1
	10	14	4	3	7	1
NR2E1	16	0	10	0	1	0
	0	0	8	0	4	0
NR2E3	20	0	12	0	1	1
	0	1	6	0	1	0
NR2F1	11	3	7	4	1	2
	1	5	2	1	2	1
NR2F2	83	350	47	264	143	149
	212	208	101	69	118	55
NR2F6	23	83	14	73	48	47
	87	126	59	36	50	21
NR3C1	683	3083	752	2739	1885	2583
	2912	3148	1490	1085	1947	538
NR3C2	161	635	115	417	396	423
	474	505	252	249	250	86
NR4A1	21	115	22	114	56	103
	122	44	43	42	68	17

NR4A2	22	22	20	24	10	12
	9	8	14	5	8	1
NR4A3	52	19	30	27	24	26
	109	29	22	23	31	9
NR5A1	4	0	4	0	1	0
	0	0	0	0	1	1
NR5A2	37	39	16	16	22	11
	37	43	31	15	21	6
NR6A1	19	22	18	13	7	19
	23	26	26	7	12	8
NRAP	8494	46727	9070	52610	21475	32967
	29778	30820	17206	14588	25308	6786
NRARP	10	30	5	8	11	7
	14	12	10	7	10	2
NRAS	92	278	93	246	108	219
	145	268	150	89	134	44
NRBF2	33	137	40	100	73	127
	143	269	87	60	126	41
NRBP1	135	835	145	503	423	682
	539	732	281	195	350	103
NRBP2	28	117	35	72	61	74
	89	132	58	19	60	27
NRCAM	64	11	35	16	17	8
	1	6	41	2	15	1
NRD1	541	3838	569	2922	1491	2486
	2414	2777	1127	869	1510	487
NRDE2	60	251	59	153	131	154
	178	281	127	89	182	39
NREP	132	712	122	489	318	535
	748	455	389	94	460	93
NRF1	39	212	47	198	84	107
	176	217	81	65	118	22
NRG1	38	0	26	1	8	1
	0	1	17	0	13	0
NRG2	12	32	23	15	18	31
	6	21	13	7	16	0
NRG3	23	0	22	1	5	11
	0	1	14	0	11	1
NRG4	31	114	20	106	51	86
	60	92	91	44	79	11
NRGN	3	6	3	0	0	1
	2	4	2	0	2	0
NRIP1	230	1218	406	1141	738	1040
	1772	1744	775	595	1102	409
NRIP2	23	8	12	11	10	7
	11	7	8	5	9	5
NRIP3	17	0	9	1	5	5
	0	4	2	0	6	1
NRK	84	40	40	34	26	18
	51	35	39	6	27	5
NRL	4	2	5	4	1	3
	4	4	3	1	6	1
NRM	12	5	4	1	3	1
	7	6	9	2	3	0
NRN1	19	80	14	63	31	49
	98	73	59	18	51	14
NRN1L	1	0	0	0	0	2
	2	1	0	1	0	0

NRP1	259	1594	185	1085	688	1207
	1008	1320	675	459	817	227
NRP2	81	83	64	145	46	37
	57	86	52	15	53	23
NRSN1	14	0	5	0	7	0
	0	0	4	0	2	0
NRSN2	4	6	4	1	6	5
	2	5	10	3	2	1
NRTN	3	0	0	0	1	0
	0	0	0	0	0	0
NRXN1	62	12	39	23	31	5
	2	12	61	10	27	13
NRXN2	30	18	21	14	6	15
	8	15	39	3	14	1
NRXN3	60	7	28	1	24	3
	6	4	25	1	12	1
NSA2	101	582	138	396	241	588
	889	712	307	171	322	98
NSD1	313	1583	412	983	777	1261
	900	1238	631	450	670	273
NSDHL	14	27	6	25	14	18
	34	30	25	12	14	4
NSF	206	589	122	797	371	388
	262	646	304	217	226	81
NSFL1C	123	567	79	410	179	324
	463	524	249	173	254	79
NSG1	11	2	5	0	3	0
	0	5	6	0	2	0
NSL1	174	522	150	407	244	352
	340	517	228	139	331	98
NSMAF	123	336	87	322	185	303
	319	339	217	133	211	58
NSMCE1	26	172	12	101	43	83
	128	164	65	22	61	15
NSMCE2	46	180	39	131	76	156
	177	264	113	47	113	36
NSMCE4A	34	112	30	85	52	117
	90	205	81	48	69	30
NSRP1	61	286	59	117	108	173
	202	242	82	95	121	48
NSUN2	125	622	119	440	252	389
	601	822	303	204	443	113
NSUN3	15	33	10	26	11	22
	37	54	27	6	19	6
NSUN4	43	116	39	95	48	117
	126	107	63	46	87	22
NSUN5	38	106	33	73	55	97
	85	167	88	35	79	14
NSUN6	53	128	44	77	60	86
	75	134	71	29	97	20
NSUN7	24	1	19	2	6	1
	5	4	6	0	6	0
NT5C	15	114	25	72	40	74
	101	164	56	20	67	17
NT5C1A	27	149	21	105	60	127
	79	81	62	45	77	9
NT5C1B	0	0	2	0	2	0
	0	0	2	0	0	0

NT5C1B-RDH14	56	31	36	10	51	
	19	13	16	28	6	66
	9					
NT5C2	453	1782	400	895	1151	1274
	1071	2500	743	684	1100	390
NT5C3	146	526	163	764	256	439
	549	594	311	131	383	117
NT5C3L	29	60	18	35	41	75
	91	99	37	44	43	9
NT5DC1	49	271	56	200	131	170
	203	263	127	94	127	37
NT5DC2	14	25	14	15	11	14
	18	24	11	5	9	9
NT5DC3	116	257	77	196	141	164
	368	383	170	73	202	36
NT5DC4	9	0	4	0	0	0
	0	0	5	0	1	0
NT5E	34	78	32	95	46	76
	30	109	36	22	40	25
NT5M	9	18	5	16	7	14
	16	13	4	5	9	1
NTAN1	36	169	29	108	91	156
	268	180	76	58	77	22
NTF3	6	8	5	4	10	11
	6	21	11	1	12	1
NTF4	5	17	1	6	12	6
	8	14	4	2	1	4
NTHL1	6	14	3	7	12	17
	16	15	9	3	7	2
NTM	25	9	14	9	14	10
	9	12	15	2	18	8
NTMT1	23	99	19	48	36	61
	102	143	72	30	42	17
NTN1	14	23	8	16	14	11
	2	6	11	5	11	6
NTN3	2	0	0	0	0	0
	0	0	0	0	0	0
NTN4	76	298	42	158	118	152
	137	194	98	74	119	44
NTN5	4	1	5	3	1	0
	1	2	2	0	1	0
NTNG1	22	21	20	1	8	0
	1	1	17	0	8	2
NTNG2	10	4	5	10	3	8
	1	2	10	1	7	0
NTPCR	37	226	43	179	101	153
	267	201	88	58	106	16
NTRK1	11	6	14	8	6	5
	0	4	16	1	7	2
NTRK2	115	175	138	142	122	38
	55	100	78	23	102	102
NTRK3	50	9	24	14	10	8
	7	2	22	2	21	5
NTS	1	0	5	0	2	0
	0	0	2	0	4	1
NTSR1	9	0	7	0	2	0
	0	0	4	0	1	0

NTSR2	7	0	5	2	2	3
	2	1	6	1	2	0
NUAK1	107	390	108	346	161	284
	231	361	192	90	156	72
NUAK2	20	7	4	5	7	7
	5	8	9	2	11	3
NUB1	143	567	127	407	274	467
	442	732	329	241	375	127
NUBP1	37	98	28	61	57	68
	127	146	58	37	69	12
NUBP2	13	35	7	45	29	47
	31	84	30	21	38	8
NUBPL	53	135	34	133	82	133
	152	209	104	30	119	22
NUCB1	89	468	90	285	140	405
	376	406	225	128	272	72
NUCB2	63	375	76	231	189	412
	197	213	117	77	142	43
NUCKS1	819	5040	979	3405	2157	3219
	3409	4580	1675	1409	2815	785
NUDC	50	244	27	128	137	182
	206	273	110	60	152	42
NUDCD1	74	235	80	166	148	165
	180	292	167	82	154	38
NUDCD2	25	92	21	77	44	66
	84	90	83	13	50	21
NUDCD3	141	633	136	397	241	375
	379	598	319	158	353	118
NUDT1	14	18	9	15	5	23
	20	23	14	4	15	3
NUDT10	5	0	3	3	3	0
	0	1	1	1	2	0
NUDT11	7	2	3	0	3	3
	1	0	5	1	6	2
NUDT12	107	380	136	426	248	374
	365	558	250	173	318	62
NUDT13	37	80	20	45	36	39
	63	123	42	43	49	11
NUDT14	9	25	9	10	8	15
	15	31	8	6	7	0
NUDT15	15	93	9	56	47	56
	64	88	31	26	37	15
NUDT16	105	369	110	182	160	178
	247	329	124	101	204	63
NUDT16L1	15	44	4	16	5	31
	34	34	16	3	16	7
NUDT17	10	11	7	7	7	14
	8	14	10	2	8	5
NUDT18	5	3	2	8	2	6
	1	6	6	1	1	1
NUDT19	74	304	60	293	128	307
	411	429	139	97	191	79
NUDT2	16	54	14	86	55	86
	107	169	27	33	38	10
NUDT21	184	614	161	483	329	593
	560	899	379	185	504	116
NUDT22	6	38	8	36	8	26
	34	54	24	10	22	8

NUDT3	18	475	31	317	127	236
	362	277	125	149	222	76
NUDT4	279	1473	354	663	928	748
	1248	1639	686	364	755	212
NUDT5	17	35	17	34	22	30
	31	48	21	14	25	10
NUDT6	10	76	15	57	29	75
	79	106	72	8	45	13
NUDT7	35	27	20	26	9	25
	40	45	18	7	21	5
NUDT8	15	50	2	35	18	18
	44	61	31	6	22	2
NUDT9	50	202	45	134	73	102
	202	190	86	55	114	35
NUF2	16	2	12	3	7	2
	3	3	20	0	14	0
NUFIP1	51	114	31	69	70	69
	88	111	58	33	84	34
NUFIP2	343	1615	350	1217	854	1232
	1215	1737	678	438	939	299
NUGGC	34	2	12	2	7	0
	0	5	18	1	10	2
NUMA1	319	1465	356	1025	837	974
	922	1804	692	643	817	434
NUMB	85	261	65	200	106	151
	198	241	102	79	131	38
NUMBL	15	31	6	16	10	19
	16	18	11	4	4	5
NUP107	131	347	95	228	192	272
	214	456	195	127	269	74
NUP133	155	639	147	475	329	491
	516	833	355	210	419	123
NUP153	174	763	178	582	453	570
	527	817	361	251	440	171
NUP155	103	313	90	248	149	239
	209	364	158	81	218	61
NUP160	113	411	109	304	189	246
	291	430	224	141	235	61
NUP188	98	189	47	144	93	130
	126	184	112	75	112	40
NUP205	173	509	166	377	248	370
	330	579	266	167	332	78
NUP210	42	91	42	48	52	75
	48	148	67	32	71	12
NUP210L	65	3	36	5	19	10
	1	14	40	9	20	5
NUP214	132	344	105	243	161	222
	293	390	177	111	235	54
NUP35	37	141	38	142	81	146
	136	212	83	50	123	25
NUP37	28	60	16	68	39	56
	80	132	56	17	68	18
NUP43	80	269	76	231	156	191
	186	352	145	88	211	53
NUP50	89	308	85	199	170	237
	234	373	152	101	194	57
NUP54	75	302	105	241	191	273
	292	490	200	128	240	100

NUP62	3	91	4	54	13	46
	67	111	10	28	19	15
NUP62CL	17	3	6	1	4	3
	0	0	2	0	9	1
NUP85	33	76	24	62	33	51
	52	121	48	24	60	33
NUP88	80	361	70	246	168	254
	245	434	177	128	183	86
NUP93	50	131	48	56	50	66
	97	133	103	38	96	26
NUP98	265	1079	285	893	596	771
	765	1215	519	313	622	206
NUPL1	128	519	147	401	259	391
	370	699	277	139	387	101
NUPL2	29	101	32	67	51	78
	85	124	54	27	72	21
NUPR1	37	92	12	72	29	111
	177	156	27	27	74	20
NUPR1L	3	0	0	0	0	0
	1	0	0	0	0	0
NUS1	58	225	48	175	108	144
	152	199	108	58	125	34
NUSAP1	51	172	60	125	46	123
	116	213	102	43	147	32
NUTF2	61	290	20	155	133	149
	244	343	104	67	140	54
NVL	65	163	70	125	96	134
	125	225	110	59	133	42
NWD1	39	13	29	9	9	7
	1	12	16	1	9	0
NXF1	84	272	66	229	116	182
	312	251	102	72	149	35
NXF2	26	0	6	0	2	0
	0	0	7	0	2	0
NXF2B	12	0	6	0	3	0
	0	0	6	0	3	0
NXF3	22	0	4	0	5	0
	0	0	6	1	6	0
NXF5	26	4	6	1	3	3
	0	5	8	1	3	3
NXN	79	289	45	211	123	177
	236	306	145	156	173	64
NXNL1	1	0	0	0	0	0
	0	0	0	0	0	0
NXNL2	24	0	11	1	3	0
	0	4	19	1	4	0
NXPE1	19	0	7	1	5	0
	0	0	12	0	1	0
NXPE2	15	1	9	5	3	2
	0	0	9	0	3	0
NXPE3	320	1429	209	1461	646	1107
	1410	1237	602	558	931	269
NXPE4	21	0	9	0	1	1
	0	1	4	1	6	1
NXPH1	9	0	8	0	3	0
	0	0	6	0	4	0
NXPH2	9	0	2	0	5	0
	0	0	7	0	6	0

NXPH3	23	10	15	9	10	8
	12	15	14	7	10	4
NXPH4	1	0	2	1	3	1
	0	0	0	0	1	1
NXT1	8	7	1	8	2	7
	8	14	5	2	5	1
NXT2	17	25	9	27	7	29
	18	23	20	6	23	6
NYAP1	19	0	17	1	2	0
	1	0	9	0	2	0
NYAP2	28	9	12	10	12	20
	15	54	11	5	13	5
NYNRIN	46	70	32	49	48	38
	59	61	58	20	48	16
NYX	9	0	2	0	1	0
	0	0	2	0	2	0
O3FAR1	37	0	8	1	3	0
	5	2	5	0	5	0
OAF	26	55	19	95	23	35
	48	55	27	22	29	9
OAS1	26	24	15	12	12	17
	16	33	23	34	16	4
OAS2	46	57	29	24	37	22
	43	48	38	47	37	5
OAS3	39	85	27	51	46	39
	64	63	42	93	51	6
OASL	8	3	3	3	0	0
	2	3	7	0	6	0
OAT	131	509	140	553	343	499
	619	693	338	141	467	91
OAZ1	347	1241	190	1056	564	912
	1483	2058	778	373	782	233
OAZ2	74	463	63	323	133	251
	423	295	130	79	155	53
OAZ3	12	5	6	1	4	4
	3	6	5	0	2	0
OBFC1	92	292	79	160	96	159
	195	154	117	77	139	32
OBP2A	6	0	2	0	1	0
	0	0	1	0	2	0
OBP2B	3	0	3	0	1	0
	0	0	2	0	0	0
OBSCN	5459	30636	5157	16704	11365	18375
	17871	30968	11103	9375	14826	4887
OBSL1	134	500	172	416	195	400
	264	541	265	141	328	83
OC90	20	0	14	0	1	1
	0	0	8	0	3	0
OCA2	42	13	26	6	7	0
	6	2	22	2	17	2
OCEL1	5	3	4	11	5	10
	6	6	2	2	8	1
OCIAD1	174	1087	205	725	400	721
	970	1263	551	306	739	178
OCIAD2	5	9	7	5	4	3
	10	14	8	2	5	0
OCLM	0	0	0	0	0	0
	1	2	0	0	0	0

OCLN	50	58	39	31	30	25
	65	81	46	15	36	9
OCM	7	0	2	0	1	0
	0	0	0	0	5	0
OCM2	7	0	0	0	0	0
	0	0	4	0	2	0
OCRL	116	259	64	205	151	213
	257	258	127	84	183	60
OCSTAMP	6	0	0	0	0	0
	0	0	0	0	1	0
ODAM	19	0	5	0	9	0
	0	0	10	0	7	0
ODC1	151	871	143	561	237	579
	508	959	498	182	460	196
ODF1	7	0	1	0	1	0
	0	0	7	0	5	0
ODF2	62	64	28	48	34	72
	43	55	31	21	38	10
ODF2L	183	218	96	146	119	150
	126	238	197	56	146	40
ODF3	3	0	10	0	2	0
	0	0	2	0	1	0
ODF3B	3	5	2	3	1	9
	8	9	5	9	9	2
ODF3L1	5	0	4	0	0	0
	0	0	3	0	4	0
ODF3L2	1	5	1	6	2	5
	3	2	11	1	5	2
ODF4	3	0	4	0	1	0
	0	0	3	0	3	0
OFCC1	26	0	16	0	4	0
	0	0	11	0	4	0
OFD1	68	229	50	144	103	162
	188	222	111	93	115	49
OGDH	338	2239	328	1532	881	1177
	1662	1854	791	635	1182	310
OGDHL	13	0	8	0	1	5
	1	4	10	0	7	1
OGFOD1	147	572	125	336	252	385
	454	690	245	155	455	95
OGFOD2	4	17	8	11	10	12
	13	25	10	2	8	3
OGFOD3	31	52	21	33	30	45
	46	99	41	31	43	17
OGFR	18	71	13	41	40	28
	21	85	40	15	43	21
OGFRL1	53	144	40	153	72	184
	186	155	99	58	113	40
OGG1	20	42	14	29	30	32
	38	44	28	12	27	5
OGN	22	52	38	77	39	23
	52	83	24	10	37	17
OGT	694	3334	792	2769	1590	2751
	2432	4506	1709	1049	1865	507
OIP5	14	55	18	29	33	63
	41	45	26	13	24	7
OIT3	19	1	9	0	3	0
	0	1	3	0	6	1

OLA1	129	960	146	593	320	573
	764	652	356	215	418	111
OLAH	13	3	8	0	0	0
	0	0	8	0	3	0
OLFM1	25	68	23	106	35	198
	58	62	36	20	34	9
OLFM2	6	5	1	2	0	1
	0	4	0	1	0	0
OLFM3	20	0	14	0	7	0
	0	0	18	0	5	0
OLFM4	22	4	9	24	13	8
	17	23	20	5	17	2
OLFML1	20	80	17	69	38	49
	31	63	35	15	37	12
OLFML2A	24	112	34	109	49	42
	106	106	41	34	63	23
OLFML2B	47	110	24	70	53	60
	54	69	60	28	38	14
OLFML3	27	94	30	108	49	106
	81	110	49	38	66	22
OLIG1	2	0	1	1	0	0
	0	0	0	0	0	0
OLIG2	3	1	5	0	1	0
	0	0	0	0	1	0
OLIG3	6	0	3	0	1	0
	0	0	2	0	1	0
OLR1	22	0	10	0	5	1
	0	0	15	0	3	0
OMA1	99	461	102	297	259	380
	366	654	240	129	274	83
OMD	17	15	13	36	10	17
	14	26	23	9	21	6
OMG	6	1	6	0	5	0
	0	2	4	0	3	0
ONECUT1	10	0	7	0	2	0
	0	0	5	0	1	0
ONECUT2	77	3	47	3	15	2
	7	2	41	1	13	0
OOEP	6	1	3	0	2	0
	0	0	3	0	1	0
OPA1	311	1964	326	1291	874	1226
	1340	1983	779	583	1152	382
OPA3	78	180	51	140	69	111
	109	159	74	47	107	19
OPALIN	34	0	18	0	2	0
	1	0	2	0	8	0
OPCML	45	4	33	0	7	2
	0	2	20	0	8	5
OPHN1	101	331	120	244	167	211
	234	352	144	120	158	56
OPLAH	35	119	20	56	50	57
	93	138	58	38	92	27
OPN1LW	10	0	2	0	0	0
	0	0	4	0	2	0
OPN1MW	13	0	6	1	2	1
	0	0	1	0	2	0
OPN1MW2	0	0	1	1	1	0
	0	0	2	0	0	0

OPN1SW	3	0	4	2	1	0
	0	3	5	0	2	0
OPN3	24	28	12	29	18	22
	34	28	28	12	22	2
OPN4	11	9	9	0	4	4
	9	18	12	3	5	4
OPN5	41	4	16	4	11	2
	6	5	19	1	11	0
OPRD1	4	0	0	0	0	3
	0	0	2	0	0	0
OPRK1	18	0	14	0	5	1
	0	1	9	1	6	0
OPRL1	16	1	11	1	2	1
	1	0	2	0	1	0
OPRM1	56	0	32	1	26	0
	1	1	21	0	20	0
OPTC	8	0	2	0	3	0
	2	0	5	0	2	0
OPTN	1049	6433	749	3221	2784	5788
	5688	7645	2864	2908	3647	1204
OR10A2	2	0	4	0	1	0
	0	0	1	0	0	0
OR10A3	6	0	2	0	4	0
	0	0	1	0	2	0
OR10A4	2	0	2	0	0	0
	0	0	0	0	1	0
OR10A5	3	0	6	0	3	0
	0	0	3	0	0	0
OR10A6	3	0	3	0	1	0
	0	0	2	1	2	0
OR10A7	2	0	2	0	2	0
	0	0	5	0	0	0
OR10AD1	3	1	4	0	1	0
	0	2	4	1	0	0
OR10AG1	8	0	2	0	0	0
	0	0	1	0	0	0
OR10C1	2	0	2	0	0	0
	0	0	3	0	2	0
OR10G2	3	0	1	0	1	0
	0	0	0	0	1	0
OR10G3	0	0	1	0	0	0
	0	0	1	0	0	0
OR10G4	5	0	3	0	2	0
	0	0	1	0	1	0
OR10G7	3	0	4	0	0	0
	0	0	2	0	1	0
OR10G8	6	0	4	0	0	0
	0	0	0	0	0	0
OR10G9	3	0	2	0	0	0
	0	0	0	0	2	0
OR10H1	3	0	0	0	1	0
	0	0	1	0	0	0
OR10H2	5	0	2	0	2	0
	0	0	0	0	1	0
OR10H3	3	0	6	0	0	0
	0	0	1	0	1	0
OR10H4	6	0	4	0	2	0
	0	0	1	0	2	1

OR10H5	2	0	0	0	0	0
	0	0	1	0	0	0
OR10J1	6	0	1	0	0	0
	0	0	0	0	1	0
OR10J3	0	0	4	0	0	0
	0	0	4	0	0	0
OR10J5	3	0	3	0	0	0
	0	0	5	0	2	0
OR10K1	5	0	5	0	2	0
	0	0	1	0	2	0
OR10K2	5	0	2	0	0	0
	0	0	1	0	0	0
OR10P1	4	0	3	0	1	0
	0	0	2	0	3	0
OR10Q1	2	0	3	0	1	0
	0	0	0	0	0	0
OR10R2	12	0	4	0	2	0
	0	0	5	0	1	0
OR10S1	1	0	3	0	0	0
	0	0	0	0	0	0
OR10T2	6	0	1	0	0	0
	0	0	1	0	1	0
OR10V1	6	0	2	1	3	0
	0	0	2	0	0	0
OR10W1	2	0	6	0	2	0
	1	0	1	0	0	0
OR10X1	5	0	2	0	0	1
	0	0	6	0	3	0
OR10Z1	1	0	0	0	0	0
	0	0	2	0	1	0
OR11A1	11	0	7	1	6	0
	0	0	4	0	0	0
OR11G2	5	0	4	0	1	0
	0	0	4	0	2	0
OR11H1	4	0	1	0	2	0
	0	0	6	0	0	0
OR11H12	4	0	0	0	0	0
	0	0	1	0	2	0
OR11H2	15	0	3	0	4	0
	0	0	3	1	4	0
OR11H4	6	0	5	0	1	0
	0	0	1	0	2	0
OR11H6	4	0	0	0	4	0
	0	0	1	0	3	0
OR11L1	6	0	3	0	0	0
	0	0	1	0	0	0
OR12D2	7	0	3	0	5	0
	0	0	1	0	2	0
OR12D3	5	0	4	0	3	0
	0	0	2	0	3	0
OR13A1	10	0	6	1	3	0
	1	0	8	0	4	0
OR13C2	10	0	2	0	0	0
	0	0	1	1	0	0
OR13C3	6	0	2	0	2	0
	1	0	3	0	3	0
OR13C4	8	0	3	0	3	1
	0	0	1	0	3	0

OR13C5	5	0	1	0	5	0
	0	1	1	0	2	0
OR13C8	1	0	0	0	0	0
	0	0	0	0	0	0
OR13C9	1	0	3	0	1	0
	0	0	6	0	1	0
OR13D1	3	0	3	1	4	0
	0	0	1	0	1	0
OR13F1	2	0	3	0	1	0
	0	0	2	0	1	0
OR13G1	5	0	3	0	0	0
	0	0	1	0	2	0
OR13H1	4	0	1	0	2	0
	0	0	0	0	0	0
OR13J1	0	0	0	0	0	0
	0	0	1	0	1	0
OR14A16	2	0	3	0	4	0
	0	0	2	0	1	0
OR14C36	5	0	1	0	0	0
	0	0	4	0	1	0
OR14I1	6	0	5	0	2	0
	0	0	2	0	2	0
OR14J1	5	0	1	0	2	0
	0	0	3	0	2	0
OR1A1	4	0	2	0	0	0
	0	0	0	0	2	0
OR1A2	3	0	4	0	1	0
	0	0	5	0	0	0
OR1B1	3	0	1	0	0	0
	0	0	4	0	0	0
OR1C1	11	0	6	0	1	0
	0	0	0	0	5	0
OR1D2	2	0	1	0	0	0
	0	0	3	0	0	0
OR1D5	3	1	4	0	1	0
	0	0	1	0	3	0
OR1E1	3	0	1	0	1	3
	0	1	3	1	1	0
OR1E2	1	0	4	0	2	2
	0	0	4	6	0	0
OR1F1	7	0	0	0	0	0
	0	0	0	0	1	0
OR1G1	9	0	6	0	1	0
	0	0	1	0	1	0
OR1I1	7	0	4	0	0	0
	0	0	6	0	1	0
OR1J1	2	0	0	0	1	0
	0	0	3	0	1	0
OR1J2	5	0	1	0	0	0
	0	0	0	0	0	0
OR1J4	5	0	1	0	0	0
	0	0	0	0	0	0
OR1K1	2	0	0	0	0	0
	0	0	1	0	0	0
OR1L1	3	0	4	0	2	0
	0	0	2	0	0	0
OR1L3	13	0	4	0	0	0
	0	0	3	0	4	0

OR1L4	11	0	2	0	2	1
	0	0	4	0	4	0
OR1L6	2	0	5	0	2	0
	0	0	0	1	2	0
OR1L8	7	2	5	0	1	0
	0	1	5	0	3	0
OR1M1	5	0	2	0	2	0
	0	0	1	0	1	0
OR1N1	3	1	0	0	1	0
	0	0	1	0	1	0
OR1N2	10	0	6	0	2	1
	0	0	2	0	2	0
OR1Q1	1	0	3	0	0	0
	0	0	0	0	4	0
OR1S1	3	0	8	0	1	0
	0	0	4	0	0	0
OR1S2	9	0	7	0	1	0
	0	0	4	0	5	0
OR2A1	7	1	2	0	2	0
	1	0	2	0	1	0
OR2A12	9	0	2	0	2	0
	0	0	2	0	1	0
OR2A14	7	0	2	0	2	0
	0	0	0	0	1	0
OR2A2	6	0	1	0	5	0
	0	0	1	0	1	0
OR2A25	7	0	3	0	1	0
	0	0	4	0	1	0
OR2A4	1	0	0	0	1	0
	0	0	0	0	2	0
OR2A42	3	0	3	1	1	0
	0	0	3	0	1	0
OR2A5	4	0	1	0	1	0
	0	0	3	0	0	0
OR2A7	12	2	0	1	0	0
	0	2	7	0	2	2
OR2AE1	2	1	0	3	5	1
	0	0	3	1	0	0
OR2AG1	2	0	0	0	3	0
	0	0	3	0	1	0
OR2AG2	4	0	3	0	0	0
	0	0	1	0	0	0
OR2AP1	7	0	0	0	1	0
	0	0	2	0	0	0
OR2AT4	7	0	4	0	1	0
	0	0	3	0	0	0
OR2B11	3	0	0	0	0	0
	0	0	1	0	1	0
OR2B2	5	1	0	0	0	0
	0	0	2	0	0	0
OR2B3	1	0	1	0	1	0
	0	0	2	0	0	0
OR2B6	5	1	3	0	1	0
	0	0	4	0	1	0
OR2C1	3	0	1	0	0	3
	0	0	2	1	0	0
OR2C3	9	0	6	0	2	0
	0	0	4	1	3	0

OR2D2	3	0	2	1	0	2
	0	1	2	0	0	1
OR2D3	1	0	3	0	1	0
	0	0	2	0	0	0
OR2F1	10	0	8	0	0	0
	0	0	0	0	1	0
OR2F2	2	0	3	0	0	0
	0	0	2	0	0	0
OR2G2	3	1	2	0	0	0
	0	0	0	0	1	0
OR2G3	2	0	4	0	1	0
	0	0	4	0	1	0
OR2G6	0	0	0	0	0	0
	0	0	3	0	0	0
OR2H1	14	0	9	0	4	0
	0	0	9	0	7	0
OR2H2	9	0	6	0	2	0
	0	0	2	0	1	0
OR2J2	10	1	5	0	2	0
	0	0	2	0	0	0
OR2J3	13	1	7	0	4	0
	0	0	3	0	2	0
OR2K2	7	0	5	3	2	5
	4	4	0	0	1	0
OR2L13	9	0	2	0	1	0
	0	0	3	0	1	0
OR2M2	7	0	6	0	1	0
	0	0	5	0	3	0
OR2M3	3	0	1	0	2	0
	0	0	2	0	3	0
OR2M4	5	0	1	0	2	0
	0	0	1	0	0	0
OR2M5	5	0	8	0	0	0
	0	0	2	0	2	0
OR2M7	5	0	1	0	1	0
	0	0	3	0	3	0
OR2S2	6	0	2	0	0	0
	0	0	1	0	1	0
OR2T1	10	0	1	0	2	0
	0	0	0	0	0	0
OR2T10	5	0	2	0	2	0
	0	0	0	0	1	0
OR2T11	2	0	2	0	0	0
	0	0	1	0	0	0
OR2T12	2	0	3	0	0	0
	0	0	4	0	0	0
OR2T2	15	0	6	0	2	0
	0	0	4	0	1	0
OR2T27	3	0	9	0	0	0
	0	0	3	0	2	0
OR2T29	2	0	4	0	1	0
	0	0	2	0	0	0
OR2T3	10	0	7	0	0	0
	0	0	1	0	1	0
OR2T33	6	0	5	0	2	0
	0	0	2	0	0	0
OR2T34	3	0	3	0	3	0
	0	0	3	0	2	0

OR2T35	0	0	1	0	2	0
	0	0	2	0	1	0
OR2T4	2	0	4	0	0	0
	0	0	1	0	3	0
OR2T5	4	0	4	0	0	0
	0	0	1	0	1	0
OR2T6	4	0	1	0	1	0
	0	0	3	0	2	0
OR2T8	1	0	4	0	1	0
	0	0	0	0	0	0
OR2V1	5	0	5	0	0	0
	0	0	2	0	1	0
OR2V2	7	0	4	0	1	0
	0	0	1	0	0	0
OR2W1	3	0	3	0	2	0
	0	0	0	0	0	0
OR2W3	0	0	6	0	0	0
	0	0	0	0	1	0
OR2W5	4	0	3	0	3	0
	0	0	4	0	2	0
OR2Y1	1	0	1	0	1	0
	0	0	0	0	0	0
OR2Z1	4	0	0	0	0	0
	0	0	0	0	0	0
OR3A1	2	0	0	0	0	0
	0	0	0	0	0	0
OR3A2	7	0	0	0	0	0
	1	0	8	2	2	0
OR3A3	0	0	3	0	3	0
	0	0	1	0	0	0
OR4A15	5	0	4	0	3	0
	0	0	3	0	0	1
OR4A16	4	0	2	0	2	0
	0	0	4	0	2	0
OR4A47	10	0	4	0	2	0
	0	0	4	0	1	0
OR4A5	7	0	3	0	0	0
	0	0	5	0	0	0
OR4B1	3	0	1	0	0	0
	0	0	3	0	0	0
OR4C11	9	0	0	0	2	0
	0	0	0	0	0	0
OR4C12	4	0	3	0	0	0
	0	0	4	0	2	0
OR4C13	5	0	0	0	0	0
	0	0	1	0	1	0
OR4C15	7	0	1	0	0	0
	0	0	2	0	2	0
OR4C16	3	0	2	0	2	0
	0	0	1	0	0	0
OR4C3	13	0	1	0	5	0
	0	0	3	0	5	0
OR4C46	2	0	1	0	0	0
	0	0	2	0	1	0
OR4C6	3	0	0	0	1	0
	0	0	0	0	1	0
OR4D1	10	0	2	0	3	0
	0	0	0	0	1	0

OR4D10	1	0	1	0	2	0
	0	0	3	0	2	0
OR4D11	4	0	3	0	1	0
	0	0	1	0	0	0
OR4D2	4	0	0	0	0	0
	0	0	1	0	0	0
OR4D5	7	0	3	0	1	0
	0	0	3	0	2	0
OR4D6	6	0	1	0	0	0
	0	0	1	0	1	0
OR4D9	4	0	2	0	1	0
	0	0	2	0	0	0
OR4E2	1	0	0	0	0	0
	0	0	0	0	0	0
OR4F15	1	0	2	0	0	0
	0	0	1	0	2	0
OR4F16	2	0	2	0	3	1
	0	2	1	0	0	0
OR4F17	4	0	5	0	3	0
	0	0	2	0	0	0
OR4F21	3	1	2	0	1	0
	0	2	1	1	0	1
OR4F29	1	0	0	0	0	0
	0	0	3	0	1	0
OR4F3	3	2	2	1	3	2
	0	0	2	0	0	0
OR4F4	8	0	0	0	1	0
	0	0	2	0	2	0
OR4F5	7	0	3	0	2	0
	1	0	2	0	1	0
OR4F6	1	0	1	0	0	0
	0	0	1	0	1	0
OR4K1	9	0	3	0	4	0
	0	0	3	0	2	0
OR4K13	10	0	2	0	1	0
	0	0	1	0	2	0
OR4K14	2	0	5	0	0	0
	0	0	3	0	3	0
OR4K15	5	0	1	0	3	0
	0	0	4	0	1	0
OR4K17	3	0	0	0	1	0
	0	0	2	0	2	0
OR4K2	12	0	4	0	2	1
	0	0	0	0	1	0
OR4K5	8	0	6	0	3	0
	0	0	2	0	2	0
OR4L1	2	0	3	0	0	0
	0	0	0	0	1	0
OR4M1	12	0	2	0	5	0
	0	0	2	0	0	0
OR4M2	1	1	1	0	2	0
	0	0	4	0	2	1
OR4N2	4	0	2	0	0	0
	0	0	2	0	1	0
OR4N4	10	1	1	0	2	1
	0	0	1	0	7	0
OR4N5	6	0	1	0	1	0
	0	0	0	0	1	0

OR4P4	4	0	5	0	0	0
	0	0	1	0	0	0
OR4Q3	16	0	0	0	2	0
	0	0	1	0	1	0
OR4S1	0	0	1	0	1	0
	0	0	2	0	0	0
OR4S2	7	0	4	0	1	0
	0	0	2	0	0	0
OR4X1	1	0	1	0	0	0
	0	0	0	0	1	0
OR4X2	0	0	1	0	0	0
	0	0	3	0	2	0
OR51A2	3	0	4	0	0	0
	0	0	1	0	0	0
OR51A4	6	0	1	1	2	0
	0	0	2	0	0	0
OR51A7	2	0	2	0	0	0
	0	1	1	0	1	0
OR51B2	10	0	12	1	2	0
	0	0	3	0	1	0
OR51B4	5	0	2	0	0	0
	0	1	2	0	0	0
OR51B5	6	0	3	0	5	1
	0	1	6	0	6	0
OR51B6	3	0	2	0	1	0
	0	0	0	0	0	0
OR51D1	6	0	3	0	0	0
	0	0	0	0	1	0
OR51E1	19	43	14	27	14	10
	37	41	22	5	34	7
OR51E2	13	1	2	0	3	2
	1	3	8	0	4	0
OR51F1	1	0	4	0	1	0
	0	0	4	0	1	0
OR51F2	7	0	11	0	0	0
	0	0	1	0	1	0
OR51G1	4	0	2	0	2	0
	0	0	0	0	0	0
OR51G2	12	0	5	0	0	0
	0	0	5	0	0	0
OR51I1	13	0	2	0	2	0
	1	0	0	0	1	0
OR51I2	9	0	1	0	0	0
	0	0	0	0	1	0
OR51L1	2	0	3	0	2	0
	0	0	1	0	3	0
OR51M1	4	0	2	0	0	0
	0	0	2	0	0	0
OR51Q1	3	0	1	0	1	0
	0	0	2	0	1	0
OR51S1	9	0	9	0	2	0
	0	0	2	0	2	0
OR51T1	10	0	8	1	1	0
	0	0	0	0	0	0
OR51V1	7	0	1	0	2	0
	0	0	4	0	2	0
OR52A1	5	1	4	0	3	0
	0	0	1	0	0	0

OR52A5	4	0	4	0	0	0
	0	0	3	0	1	0
OR52B2	4	0	0	0	1	0
	0	0	0	0	0	0
OR52B4	5	0	2	0	2	1
	0	0	2	0	4	0
OR52B6	5	1	1	0	0	0
	0	0	3	0	0	0
OR52D1	10	0	3	0	1	0
	0	0	2	0	4	0
OR52E2	1	0	6	0	0	0
	0	0	3	0	1	0
OR52E4	6	0	3	0	2	0
	0	0	2	0	1	0
OR52E6	7	0	1	0	0	0
	0	0	1	0	0	0
OR52E8	2	0	1	0	0	0
	0	0	4	0	3	0
OR52H1	3	0	2	0	0	0
	0	1	0	0	1	0
OR52I1	5	0	1	1	0	0
	0	0	2	0	1	0
OR52I2	6	0	3	1	1	0
	2	0	3	0	1	0
OR52J3	5	0	3	0	4	0
	0	0	2	0	0	0
OR52K1	7	0	3	0	1	0
	0	0	2	0	2	0
OR52K2	11	0	3	0	3	1
	0	0	4	0	0	0
OR52L1	2	0	3	0	0	0
	0	0	3	0	0	0
OR52M1	2	0	3	0	4	0
	0	0	3	0	0	0
OR52N1	4	0	8	0	0	0
	0	0	1	0	1	0
OR52N2	4	0	0	0	1	0
	0	0	0	0	1	0
OR52N4	6	1	5	0	1	2
	0	0	4	0	2	0
OR52N5	3	0	3	0	0	0
	0	0	1	0	1	0
OR52R1	5	0	1	0	1	0
	0	0	0	0	0	0
OR52W1	2	0	0	0	0	0
	0	0	0	0	1	0
OR56A1	5	0	1	1	2	0
	0	0	4	0	2	0
OR56A3	4	0	6	0	1	0
	0	0	4	0	0	0
OR56A4	4	0	6	0	3	0
	0	0	5	0	2	0
OR56A5	3	0	7	0	2	0
	0	2	7	0	4	0
OR56B1	5	0	2	0	1	0
	1	1	3	1	0	1
OR56B4	4	0	0	0	0	0
	0	0	1	0	0	0

OR5A1	5	0	7	0	2	0
	0	0	2	0	2	0
OR5A2	5	0	2	0	0	0
	0	0	3	0	2	0
OR5AC2	4	0	1	0	0	0
	0	0	0	0	0	0
OR5AK2	6	0	1	0	2	0
	0	0	2	0	3	0
OR5AN1	5	0	2	0	0	0
	0	0	1	0	0	0
OR5AP2	4	0	0	0	0	0
	0	0	2	0	2	0
OR5AR1	4	0	5	0	0	0
	0	0	1	0	2	0
OR5AS1	2	0	4	0	0	0
	0	0	1	0	1	0
OR5AU1	7	0	1	0	0	0
	0	0	2	0	0	0
OR5B12	2	0	6	0	1	2
	0	0	1	0	4	0
OR5B17	6	0	2	0	0	0
	0	0	4	0	0	0
OR5B2	4	0	4	0	0	0
	0	0	3	0	1	0
OR5B21	5	1	5	0	1	2
	0	0	0	0	0	0
OR5B3	2	0	2	0	0	0
	0	0	2	0	0	0
OR5C1	1	0	0	0	1	0
	0	0	0	0	0	0
OR5D13	6	1	1	0	1	0
	0	0	1	0	0	0
OR5D14	2	0	1	0	0	0
	0	1	1	0	0	0
OR5D16	6	0	1	0	0	0
	0	0	2	0	2	0
OR5D18	4	0	1	0	1	0
	0	0	3	0	0	0
OR5F1	1	0	1	0	0	0
	0	0	2	0	1	0
OR5H1	9	0	0	0	4	0
	0	0	2	0	2	0
OR5H14	5	0	1	1	0	0
	0	0	0	0	0	0
OR5H15	5	0	3	0	0	0
	0	0	1	0	0	0
OR5H2	4	0	0	0	2	0
	0	0	0	0	3	0
OR5H6	0	0	1	0	0	0
	0	0	0	0	1	0
OR5I1	1	0	3	0	1	0
	0	0	2	0	1	0
OR5J2	1	0	0	0	0	0
	0	0	1	0	2	0
OR5K1	3	2	2	0	2	1
	0	0	1	0	0	0
OR5K2	4	0	0	0	2	0
	3	0	0	0	0	0

OR5K3	2	2	3	0	2	1
	0	0	4	0	1	0
OR5K4	11	3	9	0	2	0
	0	1	7	0	3	0
OR5L1	7	0	1	0	1	0
	0	0	3	0	3	0
OR5L2	4	0	4	0	4	0
	0	0	2	0	1	0
OR5M1	10	0	7	0	1	0
	0	0	1	0	1	0
OR5M10	7	0	1	0	0	0
	0	0	0	0	1	0
OR5M11	3	0	2	0	0	0
	0	0	2	0	0	0
OR5M3	0	0	3	0	0	0
	0	0	2	0	2	0
OR5M8	5	0	0	0	0	0
	0	0	0	0	0	0
OR5M9	3	0	2	0	4	0
	0	0	3	0	2	0
OR5P2	4	0	0	0	2	0
	0	0	1	0	0	0
OR5P3	4	0	1	0	1	0
	0	0	3	0	1	0
OR5R1	5	0	4	0	0	0
	0	0	1	0	1	0
OR5T1	1	0	2	0	0	0
	0	0	1	0	0	0
OR5T2	4	0	3	0	1	0
	0	0	2	0	0	0
OR5T3	6	0	3	0	0	0
	0	0	6	0	1	0
OR5V1	6	0	1	0	2	0
	0	0	1	0	1	0
OR5W2	5	0	2	0	1	0
	0	0	7	0	2	0
OR6A2	12	0	5	0	4	0
	0	0	3	0	0	0
OR6B1	1	0	6	0	1	0
	0	0	2	0	1	0
OR6B2	1	0	2	0	0	0
	0	0	1	0	0	0
OR6B3	3	0	2	0	0	0
	0	0	0	0	1	0
OR6C1	4	0	0	0	1	0
	0	0	2	0	1	0
OR6C2	3	0	0	0	0	0
	0	0	1	0	0	0
OR6C3	4	0	3	0	2	0
	0	1	4	0	1	0
OR6C4	3	0	2	0	2	0
	0	1	1	0	2	0
OR6C6	4	0	1	0	0	0
	0	0	1	0	1	0
OR6C65	5	0	1	0	2	0
	0	0	3	0	4	0
OR6C68	6	0	2	0	0	0
	0	0	0	0	0	0

OR6C70	4	0	3	0	2	0
	0	0	1	0	0	0
OR6C74	3	0	3	0	0	0
	0	0	2	0	1	0
OR6C75	6	0	2	0	0	0
	0	0	2	0	0	0
OR6C76	0	0	2	0	2	0
	0	0	2	0	1	0
OR6F1	4	0	5	0	0	0
	0	0	2	0	0	0
OR6K2	5	0	1	0	0	0
	0	0	3	0	5	0
OR6K3	12	0	2	0	1	0
	0	0	3	0	4	0
OR6K6	7	0	1	0	1	0
	0	0	2	0	1	0
OR6M1	9	0	2	0	1	0
	0	0	3	0	1	0
OR6N1	4	0	2	0	2	1
	0	0	0	0	1	0
OR6N2	1	0	1	0	1	0
	0	0	3	0	1	0
OR6P1	6	0	4	0	0	0
	0	0	3	0	2	0
OR6S1	4	0	2	0	0	0
	0	0	3	0	1	0
OR6T1	2	0	0	0	1	0
	0	0	3	0	1	0
OR6V1	8	0	2	0	1	0
	0	0	2	0	0	0
OR6X1	7	0	2	0	3	0
	0	0	3	0	0	0
OR6Y1	4	0	3	0	0	0
	0	0	3	0	0	0
OR7A10	10	0	6	0	0	0
	0	0	0	0	2	0
OR7A17	3	0	5	0	0	0
	3	0	0	0	0	0
OR7A5	3	1	2	0	1	0
	0	1	3	0	4	0
OR7C1	3	0	1	0	0	0
	0	0	2	0	0	0
OR7C2	3	0	1	0	2	0
	0	0	1	0	0	0
OR7D2	8	0	6	0	1	1
	3	0	8	0	2	0
OR7D4	2	0	2	0	1	0
	0	0	3	0	1	0
OR7E24	7	0	6	0	1	0
	2	1	2	0	3	0
OR7G1	2	0	6	0	0	0
	0	0	0	0	1	0
OR7G2	5	0	3	0	2	0
	0	0	1	0	3	0
OR7G3	3	0	0	0	1	0
	0	0	2	0	0	0
OR8A1	5	0	1	0	1	0
	0	0	2	0	1	0

OR8B12	2	0	3	0	1	0
	0	0	3	1	0	0
OR8B2	8	0	1	0	2	0
	0	0	2	0	3	0
OR8B3	7	0	4	0	0	0
	0	0	1	0	2	0
OR8B4	4	0	1	0	1	0
	0	0	0	0	0	0
OR8B8	4	0	1	0	0	0
	0	0	0	0	0	0
OR8D1	4	0	3	0	1	0
	0	0	0	0	4	0
OR8D2	9	0	2	0	0	0
	0	0	1	0	2	0
OR8D4	2	0	3	0	2	0
	0	1	3	0	2	0
OR8G2	3	0	7	0	0	0
	0	0	2	0	1	0
OR8G5	8	0	3	0	0	1
	0	1	3	0	2	1
OR8H1	3	0	1	0	1	0
	0	0	3	0	0	0
OR8H2	3	0	3	0	4	0
	0	0	2	0	0	0
OR8H3	9	0	3	0	1	0
	0	0	3	0	0	0
OR8I2	3	0	3	0	0	0
	0	0	2	0	0	0
OR8J1	6	0	1	0	0	0
	0	0	0	0	1	0
OR8J3	10	0	1	0	1	0
	0	0	2	0	0	0
OR8K1	7	0	2	0	1	0
	0	0	2	0	0	0
OR8K3	2	0	1	0	0	0
	0	0	5	0	2	0
OR8K5	3	0	2	0	0	0
	0	0	2	0	2	0
OR8S1	6	0	6	0	3	1
	0	0	2	0	1	0
OR8U1	7	0	6	0	1	0
	0	0	0	0	0	0
OR9A2	2	2	6	2	0	1
	1	4	1	2	0	0
OR9A4	8	0	1	0	0	0
	0	1	0	0	1	0
OR9G1	16	0	15	0	3	0
	0	0	7	0	2	0
OR9G4	8	0	1	0	1	1
	0	0	2	0	2	0
OR9I1	3	0	3	1	2	0
	0	0	2	0	1	0
OR9K2	3	0	2	0	0	0
	0	0	1	0	2	0
OR9Q1	12	1	5	0	2	0
	2	2	6	0	4	0
OR9Q2	5	0	3	1	2	0
	0	0	1	0	1	0

ORAI1	8	31	1	28	16	35
	34	62	21	16	33	5
ORAI2	43	14	24	8	11	8
	7	12	22	1	15	3
ORAI3	9	16	7	26	9	18
	15	25	16	6	22	6
ORAOV1	29	70	16	37	40	32
	51	89	29	18	28	12
ORC1	29	6	13	6	6	5
	3	0	22	0	11	0
ORC2	67	303	80	233	134	188
	278	363	156	107	236	61
ORC3	75	337	105	285	145	225
	262	458	188	110	223	80
ORC4	172	573	177	391	287	498
	458	695	319	194	416	111
ORC5	40	95	31	64	64	74
	70	142	65	22	69	19
ORC6	15	4	10	6	3	4
	0	6	8	0	11	2
ORM1	3	0	1	0	0	0
	1	0	6	0	2	0
ORM2	6	0	5	0	1	0
	0	0	3	0	1	0
ORMDL1	54	202	44	209	99	163
	212	320	130	83	133	49
ORMDL2	24	14	9	12	6	18
	16	42	19	6	11	2
ORMDL3	42	161	32	111	75	120
	110	149	64	47	71	30
OS9	107	362	82	224	182	276
	241	453	172	134	188	89
OSBP	165	1205	244	990	671	865
	1082	947	415	284	598	196
OSBP2	25	37	21	18	15	19
	14	34	19	4	19	5
OSBPL10	36	48	25	25	22	19
	27	39	30	9	20	6
OSBPL11	209	1173	142	762	514	812
	1138	1186	456	358	637	251
OSBPL1A	321	1957	384	1454	825	1470
	1594	1971	863	471	1055	275
OSBPL2	58	182	69	160	98	157
	156	208	103	49	93	29
OSBPL3	46	32	26	44	99	71
	64	320	43	72	24	18
OSBPL5	37	73	20	49	33	22
	29	42	29	20	45	19
OSBPL6	205	1344	451	996	562	893
	946	657	451	281	546	175
OSBPL7	52	271	90	287	70	229
	138	80	114	27	71	18
OSBPL8	161	703	206	495	339	463
	435	826	359	196	357	152
OSBPL9	368	1240	243	907	695	867
	1413	2331	900	651	1290	332
OSCAR	9	0	6	2	1	0
	4	0	3	0	2	1

OSCP1	17	23	6	13	9	8
	25	25	17	8	18	3
OSGEP	28	216	34	119	63	86
	145	139	78	49	92	24
OSGEPL1	37	121	21	81	75	88
	75	154	74	27	67	19
OSGIN1	15	33	9	21	11	34
	51	29	20	12	33	5
OSGIN2	90	269	70	161	139	180
	190	318	162	111	159	66
OSM	4	0	2	2	1	0
	0	0	0	0	1	0
OSMR	52	89	41	74	57	67
	76	99	56	31	61	23
OSR1	14	27	9	27	7	16
	11	28	6	3	7	4
OSR2	16	16	9	12	5	10
	10	11	7	2	6	3
OST4	38	179	19	133	71	107
	161	291	83	35	101	22
OSTC	61	220	51	147	98	143
	226	280	93	48	99	33
OSTCP2	2	0	1	0	1	0
	0	0	1	0	0	0
OSTF1	27	65	24	63	27	49
	45	74	49	23	25	13
OSTM1	47	216	55	175	79	213
	147	230	104	50	115	36
OSTN	4	3	2	1	0	0
	1	5	2	0	1	0
OTC	14	0	4	0	5	0
	2	0	3	0	2	3
OTOA	58	1	25	1	5	2
	5	2	20	0	20	2
OTOF	42	4	20	0	5	7
	5	7	16	2	7	0
OTOG	28	1	34	0	11	0
	0	0	19	0	10	0
OTOGL	67	27	66	12	34	16
	18	54	97	6	69	9
OTOL1	8	0	7	0	7	0
	0	1	5	0	8	0
OTOP1	16	0	1	0	2	0
	0	0	4	1	5	0
OTOP2	7	0	3	0	0	0
	0	0	3	0	0	0
OTOP3	3	0	5	0	0	0
	0	0	0	0	1	0
OTOR	10	0	10	0	3	0
	0	0	4	0	5	0
OTOS	3	0	1	0	0	0
	0	0	0	0	1	0
OTP	2	0	0	0	2	0
	0	0	3	0	0	0
OTUB1	15	82	15	69	32	76
	101	110	43	16	54	20
OTUB2	7	3	10	2	7	1
	2	3	11	2	4	0

OTUD1	68	213	82	154	97	102
	207	976	260	228	562	90
OTUD3	94	239	60	193	81	157
	209	263	132	50	158	46
OTUD4	292	1625	317	1214	791	925
	1185	1151	546	444	707	246
OTUD5	49	177	32	112	82	123
	160	212	79	65	95	49
OTUD6A	7	0	1	0	0	0
	0	1	1	0	2	0
OTUD6B	67	303	76	237	184	266
	273	370	157	107	186	67
OTUD7A	18	7	15	6	7	7
	3	3	14	0	4	2
OTUD7B	200	732	176	516	392	596
	529	760	340	256	368	150
OTX1	10	0	15	0	2	0
	0	0	1	0	2	0
OTX2	17	0	12	0	4	0
	0	0	11	0	3	0
OVCA2	1	4	1	6	2	4
	3	17	5	1	1	2
OVCH1	42	6	15	16	10	4
	11	9	22	3	18	5
OVCH2	32	2	17	1	10	2
	2	6	17	0	14	0
OVGP1	27	12	20	19	13	29
	17	34	28	0	15	5
OVOL1	16	0	7	0	0	0
	0	0	3	0	0	0
OVOL2	5	0	0	0	0	0
	0	0	1	0	0	0
OVOL3	3	0	1	0	2	0
	0	0	3	0	0	0
OVOS	7	3	23	5	5	0
	1	3	11	1	2	0
OVOS2	61	60	21	77	32	56
	12	15	68	0	104	5
OXA1L	186	832	151	668	271	607
	973	1023	513	255	482	138
OXCT1	116	327	43	137	113	124
	148	355	89	74	127	113
OXCT2	0	0	0	0	0	0
	0	0	0	0	1	1
OXER1	3	2	3	1	0	3
	0	5	3	1	1	0
OXGR1	18	4	5	1	1	2
	1	2	4	0	5	0
OXLD1	4	38	4	20	14	18
	26	62	19	9	17	4
OXNAD1	92	439	110	222	151	245
	342	562	232	100	327	69
OXR1	268	1167	256	829	595	706
	1170	1493	596	453	722	248
OXSM	35	86	9	63	44	65
	83	151	53	23	62	17
OXSR1	168	709	180	481	297	429
	582	573	274	204	368	115

OXTR	33	4	14	5	15	2
	1	4	19	0	7	2
P2RX1	9	4	6	3	2	9
	0	3	5	0	2	2
P2RX2	5	0	6	0	1	0
	0	0	1	0	2	0
P2RX3	5	0	5	0	4	0
	0	2	3	0	0	0
P2RX4	7	19	7	13	9	16
	11	15	8	4	14	5
P2RX5	17	79	26	31	23	37
	50	85	40	16	27	15
P2RX6	77	199	70	200	100	209
	122	272	114	59	133	36
P2RX7	42	12	13	6	11	7
	4	9	19	3	13	5
P2RY1	42	149	53	123	154	65
	146	177	73	49	58	15
P2RY10	14	2	2	2	2	0
	0	0	2	1	2	0
P2RY11	2	0	7	0	4	0
	0	4	0	1	1	0
P2RY12	11	5	8	2	6	5
	4	4	13	1	2	4
P2RY13	13	13	9	6	11	10
	10	6	12	2	11	2
P2RY14	26	59	17	37	49	58
	64	133	62	23	53	28
P2RY2	31	189	46	118	87	169
	166	194	76	52	94	27
P2RY4	5	9	6	3	4	8
	9	11	5	8	4	0
P2RY6	13	11	7	6	7	9
	4	22	10	3	10	1
P2RY8 (NC_000023 1581464..1656037)				14	13	4
	6	3	3	12	9	4
	3	5	1			
P2RY8 (NC_000024 1531464..1606037)				4	12	5
	8	3	3	14	10	5
	2	6	1			
P4HA1	321	1028	250	824	731	759
	958	1578	499	415	719	336
P4HA2	45	107	19	82	72	126
	73	99	66	27	58	16
P4HA3	19	7	15	1	3	0
	0	2	12	1	8	0
P4HB	154	927	168	643	375	686
	843	864	486	314	521	187
P4HTM	32	38	8	39	22	38
	45	76	28	11	27	13
PA2G4	110	622	109	327	269	372
	468	640	217	190	295	112
PAAF1	156	135	98	79	93	104
	109	185	97	48	93	24
PABPC1	100	759	122	607	307	519
	650	683	236	185	367	156
PABPC1L	15	8	6	7	6	4
	4	1	4	2	5	1

PABPC1L2A	2	0	0	0	0	0
	0	0	2	0	0	0
PABPC1L2B	2	0	0	0	0	0
	0	0	2	0	3	0
PABPC3	12	0	5	0	1	0
	0	0	7	0	3	0
PABPC4	386	3578	506	2151	1123	2271
	3201	2129	1021	862	1190	548
PABPC4L	9	19	13	17	10	9
	19	19	11	5	9	6
PABPC5	21	14	8	17	5	22
	13	16	11	7	10	4
PABPN1	17	124	7	34	26	103
	119	144	31	43	50	17
PABPN1L	3	0	3	0	1	0
	0	0	0	0	2	0
PACRG	8	9	7	2	8	7
	15	16	4	2	9	2
PACRGL	19	90	20	67	33	33
	73	77	49	15	50	15
PACS1	43	200	54	101	60	83
	132	120	63	31	56	23
PACS2	116	562	118	376	184	389
	373	482	205	192	292	72
PACSIN1	15	2	16	1	4	2
	0	1	12	0	4	1
PACSIN2	74	236	50	165	107	199
	221	284	139	98	147	61
PACSIN3	311	1708	280	925	645	987
	1348	2170	674	410	855	227
PADI1	21	0	18	0	5	0
	0	0	10	0	7	0
PADI2	750	3499	654	1714	2030	2027
	3185	4259	1203	1313	1541	566
PADI3	11	0	8	1	1	0
	0	0	6	0	4	0
PADI4	10	4	16	1	3	2
	2	0	5	0	2	0
PADI6	12	0	8	0	4	0
	0	0	7	0	2	0
PAEP	4	0	2	0	1	0
	0	0	0	0	3	0
PAF1	60	184	45	126	104	143
	125	130	73	53	93	30
PAFAH1B1	376	2307	442	1903	1120	1614
	1500	2119	944	661	1150	397
PAFAH1B2	131	498	106	370	232	356
	372	650	221	162	316	77
PAFAH1B3	8	24	4	7	5	15
	21	18	7	9	13	0
PAFAH2	47	109	32	91	51	90
	92	128	46	23	58	23
PAG1	78	135	41	74	74	104
	126	147	100	52	106	23
PAGE1	5	0	0	0	0	0
	0	0	1	0	2	0
PAGE2	1	0	0	0	2	0
	0	0	5	0	1	0

PAGE2B	3	0	4	0	2	0	0	0	3	0	0	0
PAGE4	9	8	2	5	6	11	7	9	6	2	4	3
PAGE5	15	0	7	0	3	0	0	0	1	0	0	0
PAGR1	47	134	22	96	79	121	146	131	55	37	77	18
PAH	26	0	19	0	2	0	0	0	4	0	7	0
PAICS	62	240	50	137	126	124	177	211	107	68	126	49
PAIP1	262	1569	215	1042	532	1174	1188	1438	620	418	672	222
PAIP2	182	886	108	647	494	701	937	1021	365	219	497	154
PAIP2B	544	1522	195	1056	1065	969	1277	3260	1149	424	1188	439
PAK1	211	1079	220	942	608	659	671	571	261	217	391	101
PAK1IP1	44	157	30	78	86	99	134	182	71	44	66	17
PAK2	162	1078	193	717	455	610	658	864	265	231	369	152
PAK3	33	24	17	30	11	5	10	11	19	2	6	8
PAK4	12	16	5	12	7	17	9	11	5	0	3	4
PAK6	14	13	19	7	4	7	8	11	18	4	8	2
PAK7	31	0	16	0	3	0	0	0	18	0	7	0
PALB2	56	148	34	104	57	82	109	137	68	41	70	24
PALD1	35	56	12	44	27	42	46	78	34	17	49	6
PALLD	493	2598	523	1750	1126	1661	1392	2604	1046	711	1115	457
PALM	9	45	12	26	21	15	21	36	23	10	21	17
PALM2	44	7	12	2	10	7	8	8	35	2	17	3
PALM2-AKAP2	135	469	103	295	249	295	411	527	236	192	253	113
PALM3	8	0	6	0	2	0	0	0	5	0	3	0
PALMD	377	2083	382	1214	982	1206	1039	1278	654	742	748	329
PAM	528	2345	551	1469	1338	1096	1522	3335	921	726	1288	601
PAM16	12	22	4	36	14	34	41	95	14	10	20	5
PAMR1	29	47	35	54	14	42	3	29	21	5	19	16
PAN2	145	474	114	272	185	408	293	558	228	115	266	89

PAN3	87	320	69	239	136	192
	256	503	164	100	186	73
PANK1	78	249	60	95	141	84
	228	437	156	99	233	52
PANK2	52	187	32	130	71	113
	152	229	78	66	84	27
PANK3	119	474	123	356	241	335
	427	550	180	138	305	100
PANK4	49	142	32	95	91	104
	166	192	92	53	133	40
PANX1	62	421	91	202	46	228
	202	133	150	122	129	50
PANX2	5	1	2	1	0	0
	2	0	0	1	1	0
PANX3	11	0	7	0	4	0
	0	0	5	0	0	0
PAOX	12	8	9	10	8	8
	13	25	12	4	11	0
PAPD4	115	457	120	411	239	347
	417	685	274	161	297	103
PAPD5	94	311	72	240	148	196
	230	230	133	104	151	36
PAPD7	60	237	53	170	97	178
	151	187	93	63	112	32
PAPL	13	0	5	0	4	3
	0	1	17	0	5	0
PAPLN	38	34	45	75	22	43
	33	40	19	7	20	16
PAPOLA	285	1256	285	860	675	901
	1007	1669	555	358	784	221
PAPOLB	0	0	0	0	0	1
	0	0	0	1	0	0
PAPOLG	96	242	88	161	130	171
	218	291	144	97	205	48
PAPPA	77	20	42	28	34	19
	27	73	72	23	64	13
PAPPA2	53	4	40	5	17	3
	0	5	38	0	11	0
PAPSS1	38	120	47	104	55	94
	79	67	64	27	70	20
PAPSS2	27	25	33	35	40	42
	66	124	75	6	34	25
PAQR3	33	142	28	134	59	112
	107	192	66	44	116	21
PAQR4	19	54	14	37	32	26
	55	82	19	24	40	17
PAQR5	25	1	15	1	9	3
	0	6	23	1	8	0
PAQR6	7	3	3	2	1	3
	2	10	3	0	8	0
PAQR7	18	19	12	10	7	18
	14	19	15	4	7	2
PAQR8	31	12	22	12	9	15
	4	16	18	3	8	1
PAQR9	8	31	4	4	7	15
	40	24	17	2	20	4
PARD3	178	928	258	595	571	676
	491	990	384	343	411	148

PARD3B	117	228	105	243	146	180
	179	196	132	70	142	63
PARD6A	6	3	2	4	1	7
	3	10	6	1	4	2
PARD6B	25	58	22	53	23	35
	40	29	35	12	38	9
PARD6G	16	6	9	17	10	8
	16	10	16	6	11	3
PARG	146	609	153	370	271	425
	526	579	328	198	431	112
PARK2	124	347	90	433	217	412
	363	426	180	90	286	104
PARK7	383	2911	426	2169	1093	1903
	3984	3747	1317	617	1449	318
PARL	58	260	59	213	93	188
	221	297	148	77	192	50
PARM1	139	600	138	543	359	437
	499	421	195	95	274	93
PARN	62	264	73	186	136	196
	207	285	146	74	131	53
PARP1	211	1149	199	771	538	790
	1011	831	383	355	569	178
PARP10	19	65	18	39	28	37
	47	50	22	19	34	12
PARP11	34	83	40	43	50	47
	35	89	48	26	53	14
PARP12	31	100	32	57	35	79
	71	80	60	44	59	4
PARP14	128	569	137	442	271	404
	468	711	298	330	537	148
PARP15	26	35	24	15	27	24
	32	79	17	37	43	12
PARP16	18	34	12	24	13	24
	32	29	22	9	17	10
PARP2	54	210	54	159	81	137
	189	166	85	63	150	35
PARP3	42	135	34	83	51	86
	159	91	72	31	57	20
PARP4	100	348	100	281	160	263
	180	348	196	97	203	58
PARP6	109	396	84	266	146	277
	360	380	174	113	194	67
PARP8	72	91	62	57	45	78
	55	84	70	23	73	15
PARP9	85	248	63	147	105	189
	199	256	121	129	164	55
PARPBP	29	16	13	10	9	7
	31	8	22	2	30	2
PARS2	11	21	11	23	12	18
	37	28	14	6	17	7
PARVA	90	241	87	248	129	171
	144	245	123	79	123	77
PARVB	139	602	77	268	267	284
	559	643	158	230	327	91
PARVG	17	16	12	4	5	1
	8	13	12	3	7	2
PASD1	28	0	16	1	9	4
	0	0	13	0	6	0

PASK	33	25	23	10	17	9
	14	8	19	6	19	0
PATE1	17	0	17	0	3	0
	0	0	12	0	7	0
PATE2	10	6	12	2	4	3
	3	2	5	1	6	1
PATE3	6	1	3	0	1	0
	0	0	4	0	2	0
PATE4	21	1	6	0	1	5
	0	1	8	0	2	0
PATL1	102	522	84	354	216	335
	451	347	143	144	227	70
PATL2	28	2	11	3	3	1
	1	1	11	0	6	1
PATZ1	28	85	23	55	44	54
	47	155	43	34	67	20
PAWR	8	34	14	12	12	6
	13	16	14	7	18	10
PAX1	24	3	13	2	3	6
	8	0	8	1	12	0
PAX2	11	1	10	0	5	0
	0	0	4	0	4	0
PAX3	51	103	50	83	68	103
	83	53	64	37	48	18
PAX4	9	0	7	1	1	0
	0	0	6	0	3	0
PAX5	14	1	25	0	3	0
	3	1	9	0	14	1
PAX6	34	3	32	0	7	5
	2	3	19	0	10	1
PAX7	36	59	25	33	53	66
	35	80	53	27	40	10
PAX8	19	1	24	5	2	5
	0	5	17	3	8	0
PAX9	13	0	8	0	5	0
	0	1	4	1	4	0
PAXIP1	49	106	35	65	62	73
	85	125	72	38	76	21
PBK	12	0	5	0	4	0
	2	0	11	0	9	0
PBLD	33	28	24	24	19	25
	17	30	30	12	26	5
PBOV1	12	0	6	0	3	0
	0	0	10	0	3	0
PBRM1	199	988	207	712	492	670
	800	918	399	325	473	174
PBX1	266	911	279	762	571	702
	757	1099	388	355	518	222
PBX2	51	234	46	138	118	172
	227	198	90	69	125	39
PBX3	46	86	32	64	62	91
	72	76	49	32	43	11
PBX4	8	1	1	0	1	3
	0	1	4	0	1	0
PBXIP1	190	819	211	631	456	582
	409	883	285	238	359	196
PC	38	65	23	28	25	28
	36	75	33	16	26	22

PCBD1	26	115	20	51	30	59
	82	127	36	18	42	16
PCBD2	65	310	65	191	121	169
	265	309	135	85	163	62
PCBP1	133	1307	116	676	392	499
	1139	660	220	222	286	114
PCBP2	176	1257	155	922	466	677
	995	1017	322	241	446	156
PCBP3	13	0	17	2	3	1
	0	1	9	0	2	0
PCBP4	116	376	102	225	188	239
	305	476	124	117	189	69
PCCA	54	285	72	149	97	145
	261	303	186	67	178	51
PCCB	76	279	65	211	124	172
	307	413	167	77	200	38
PCDH1	37	181	22	83	74	82
	140	162	76	51	78	31
PCDH10	28	3	16	2	4	1
	1	3	15	0	11	2
PCDH11X	77	5	17	0	25	2
	32	15	15	1	11	0
PCDH11Y	7	3	18	2	2	2
	2	3	20	0	10	0
PCDH12	26	62	16	40	37	32
	47	39	27	14	26	6
PCDH15	107	0	58	1	32	2
	0	0	57	0	47	0
PCDH17	41	53	12	40	15	11
	24	17	24	8	16	6
PCDH18	54	162	68	165	96	119
	74	202	71	43	68	57
PCDH19	51	44	18	48	36	20
	22	22	34	1	31	5
PCDH20	34	6	10	2	6	3
	0	1	12	2	6	1
PCDH7	49	56	30	18	37	25
	12	20	40	7	34	10
PCDH8	9	1	5	12	2	1
	12	4	5	2	6	1
PCDH9	55	204	58	140	178	171
	125	191	74	45	91	43
PCDHA1	5	0	3	0	2	1
	0	0	5	0	0	0
PCDHA2	7	0	4	0	1	0
	0	0	4	0	2	0
PCDHA3	6	1	4	0	2	0
	0	6	1	0	0	0
PCDHA4	5	0	1	0	1	0
	0	0	2	0	0	0
PCDHA5	2	1	2	0	0	0
	0	0	2	0	1	0
PCDHA6	0	0	2	0	0	0
	0	0	0	0	0	0
PCDHA7	1	0	1	0	0	0
	0	0	0	0	1	0
PCDHA8	2	0	1	0	0	0
	0	0	1	0	0	0

PCDHA9	3	0	1	0	0	0
	0	1	0	0	1	0
PCDHAC2	0	0	0	0	0	2
	0	0	0	0	0	0
PCDHB1	2	0	1	0	1	0
	0	0	1	0	0	0
PCDHB10	2	4	0	3	1	5
	5	2	0	2	0	2
PCDHB11	1	5	4	2	1	3
	1	1	1	3	6	0
PCDHB12	0	5	3	5	0	2
	2	1	2	0	2	5
PCDHB13	6	5	6	3	3	4
	1	3	2	0	1	2
PCDHB14	0	4	1	9	2	1
	3	1	5	1	4	2
PCDHB15	1	0	1	1	2	4
	1	1	1	0	2	2
PCDHB16	0	4	0	7	1	3
	1	2	1	1	1	2
PCDHB2	1	0	2	5	0	1
	3	2	1	3	5	4
PCDHB3	2	0	0	4	1	1
	5	2	0	3	2	2
PCDHB4	2	1	3	1	1	4
	5	1	3	1	4	2
PCDHB5	1	1	1	2	3	2
	1	6	0	1	0	3
PCDHB6	0	0	0	0	1	1
	0	0	2	1	1	2
PCDHB7	1	0	1	2	2	2
	1	1	2	0	0	1
PCDHB8	2	2	1	2	1	0
	4	3	3	1	1	0
PCDHB9	3	3	2	1	1	0
	3	0	1	3	1	0
PCDHGA1	17	9	18	12	8	5
	7	5	11	10	12	4
PCDHGA10	0	0	0	1	0	0
	0	0	0	0	0	0
PCDHGA11	0	1	0	0	0	0
	0	0	0	0	0	0
PCDHGA12	0	1	0	0	0	0
	0	0	1	0	0	0
PCDHGA2	0	0	0	0	0	0
	1	0	0	1	1	0
PCDHGA3	0	1	0	0	0	0
	0	0	0	2	1	3
PCDHGA4	2	4	1	1	1	2
	3	0	0	0	0	1
PCDHGA5	0	1	0	0	1	0
	1	4	0	1	0	2
PCDHGA6	2	2	0	1	0	0
	1	1	0	0	0	1
PCDHGA7	1	4	1	1	1	3
	0	5	0	0	1	0
PCDHGA9	0	0	0	0	0	1
	0	1	0	0	0	0

PCDHGB1	1	1	0	1	1	3
	0	3	2	0	0	0
PCDHGB2	0	2	0	1	1	1
	3	2	1	1	0	0
PCDHGB3	1	2	0	1	0	2
	5	4	0	1	1	2
PCDHGB5	0	0	1	0	0	0
	0	0	1	0	1	0
PCDHGB6	0	0	0	0	0	0
	0	0	0	0	1	0
PCDHGB7	0	0	0	3	0	0
	2	0	0	0	0	0
PCDHGC3	0	3	1	5	1	2
	1	2	0	0	3	1
PCDP1	25	0	22	2	5	0
	0	3	14	0	7	1
PCED1A	21	35	9	29	22	32
	32	69	35	17	25	5
PCED1B	10	4	6	3	4	3
	0	7	2	2	3	3
PCF11	293	1258	365	943	687	1196
	718	1121	515	397	570	199
PCGF1	25	64	9	43	41	58
	69	76	38	20	51	7
PCGF2	9	45	13	19	12	11
	31	22	15	10	12	8
PCGF3	77	243	66	193	102	146
	163	249	106	52	135	68
PCGF5	151	804	197	455	349	377
	546	704	364	205	326	149
PCGF6	25	73	15	66	44	47
	86	117	45	24	59	16
PCID2	63	203	54	153	109	170
	173	346	132	59	164	42
PCIF1	24	91	15	54	35	30
	62	58	51	20	61	20
PCK1	19	3	9	7	22	3
	2	1	8	0	5	8
PCK2	10	5	13	8	5	5
	4	9	12	3	7	7
PCLO	140	19	118	14	69	69
	8	3	79	3	44	5
PCM1	1236	6944	1517	4265	3398	4454
	4302	6035	2384	2247	3625	1304
PCMT1	183	1072	192	830	414	696
	882	1018	514	257	540	157
PCMTD1	928	4049	693	2696	2514	2384
	3938	5439	1747	1090	2499	745
PCMTD2	221	817	181	710	425	511
	685	1002	395	282	522	163
PCNA	47	147	31	119	75	99
	108	208	86	60	104	26
PCNP	115	698	123	540	319	534
	597	736	321	197	335	113
PCNT	863	4114	876	2206	2047	2266
	3812	6568	1525	2266	2168	1170
PCNX	410	2037	423	1788	998	1541
	1479	2121	929	686	1136	386

PCNXL2	50	48	29	56	32	17
	5	36	42	3	36	7
PCNXL3	37	52	21	37	26	41
	47	77	42	18	43	11
PCNXL4	57	312	78	299	186	315
	265	364	149	104	172	53
PCOLCE	15	42	15	50	20	19
	37	56	23	17	23	4
PCOLCE2	31	27	13	56	77	69
	23	145	25	6	46	30
PCP2	2	1	5	0	1	0
	0	0	1	0	0	0
PCP4	4	0	4	0	1	0
	0	0	3	0	1	0
PCP4L1	10	0	8	3	0	0
	0	0	5	0	2	0
PCSK1	32	0	30	0	10	1
	1	0	20	0	6	0
PCSK1N	0	0	0	0	0	0
	1	0	0	0	1	0
PCSK2	22	1	12	5	4	3
	2	3	12	1	15	1
PCSK4	2	2	2	0	3	1
	0	3	2	1	1	0
PCSK5	86	67	38	69	51	63
	43	57	68	10	53	23
PCSK6	83	64	25	58	79	118
	34	108	51	7	40	21
PCSK7	28	142	32	83	62	97
	95	143	61	40	78	30
PCSK9	10	0	2	0	1	0
	0	0	8	0	4	0
PCTP	35	166	27	110	68	122
	169	174	82	44	83	19
PCYOX1	462	2549	382	1826	1618	2142
	1807	2113	920	591	1061	389
PCYOX1L	19	13	9	12	7	15
	13	15	16	4	9	3
PCYT1A	88	467	82	296	265	325
	351	364	177	115	229	58
PCYT1B	43	2	11	4	4	1
	0	0	11	2	3	1
PCYT2	14	101	16	29	37	35
	62	110	45	16	65	17
PDAP1	119	926	157	476	383	674
	758	707	313	264	435	134
PDC	19	2	8	1	6	0
	0	2	6	0	3	1
PDCD1	8	0	3	0	0	0
	0	1	1	0	3	0
PDCD10	58	234	54	144	112	180
	246	279	108	74	141	50
PDCD11	106	288	129	190	137	203
	207	304	146	89	169	57
PDCD1LG2	36	32	19	42	13	35
	21	28	13	15	21	3
PDCD2	77	288	70	200	137	249
	324	414	191	88	175	54

PDCD2L	20	90	11	75	34	85
	78	128	67	22	57	15
PDCD4	103	414	111	353	147	323
	236	425	196	94	226	76
PDCD5	97	503	44	272	128	302
	452	665	264	134	234	64
PDCD6	29	106	34	83	57	73
	80	154	56	27	56	18
PDCD6IP	163	1048	203	833	428	749
	614	966	462	284	638	212
PDCD7	45	152	17	82	58	101
	99	131	61	39	79	18
PDCL	33	110	30	107	68	117
	83	139	82	34	61	29
PDCL2	7	0	3	0	3	0
	0	0	2	0	1	0
PDCL3	30	185	36	116	69	122
	151	122	53	28	80	21
PDDC1	38	94	31	57	44	84
	68	112	54	37	68	25
PDE10A	56	31	33	32	26	18
	16	26	36	8	34	8
PDE11A	111	336	298	199	438	186
	150	230	81	123	95	128
PDE12	39	163	33	101	75	94
	127	177	54	51	95	28
PDE1A	65	95	64	105	39	90
	51	90	76	14	44	12
PDE1B	26	9	24	6	9	3
	10	6	15	0	4	4
PDE1C	36	23	29	21	26	23
	15	23	44	9	40	11
PDE2A	48	132	48	61	60	51
	68	139	59	31	69	29
PDE3A	84	199	71	105	52	78
	88	188	95	45	38	58
PDE3B	36	21	19	24	41	25
	17	33	26	4	19	46
PDE4A	50	186	31	77	91	95
	165	83	64	53	53	20
PDE4B	155	287	101	288	211	342
	296	619	224	246	181	99
PDE4C	34	19	30	12	28	13
	44	70	14	23	45	7
PDE4D	845	3214	647	2762	2012	2844
	3492	5035	2135	1878	2316	918
PDE4DIP	10433	51356	10870	36742	26833	40144
	37216	59236	23635	20053	32834	11171
PDE5A	188	1008	434	979	456	533
	718	1118	550	205	381	141
PDE6A	109	6	50	3	24	11
	2	10	47	7	17	2
PDE6B	15	6	17	2	6	1
	1	0	16	0	5	0
PDE6C	31	0	14	0	6	1
	3	2	16	0	17	0
PDE6D	16	28	7	27	20	34
	23	28	21	5	24	7

PDE6G	3	0	0	0	0	0
	1	3	0	0	1	0
PDE6H	2	0	5	0	2	0
	0	0	4	0	0	0
PDE7A	262	1052	193	605	628	699
	1247	2106	515	616	843	304
PDE7B	80	253	48	208	112	159
	171	220	165	99	188	63
PDE8A	80	269	67	205	122	171
	208	336	158	96	163	54
PDE8B	26	3	29	6	6	0
	3	5	16	2	8	4
PDE9A	18	38	15	20	8	18
	14	23	17	14	9	2
PDF	0	6	0	5	2	4
	1	13	1	3	2	5
PDGFA	38	129	35	118	50	188
	112	106	58	49	62	17
PDGFB	25	92	18	81	53	68
	77	86	72	28	63	22
PDGFC	23	182	111	119	76	115
	102	172	102	23	56	30
PDGFD	21	81	31	76	52	54
	45	95	36	21	49	18
PDGFRA	195	652	207	927	435	691
	554	1068	404	252	461	223
PDGFRB	136	708	117	403	274	394
	330	572	297	158	310	125
PDGFRL	20	42	7	51	25	46
	18	72	35	8	32	7
PDHA1	371	2260	284	1255	920	1198
	2554	2675	1069	683	1463	394
PDHA2	14	0	8	0	2	0
	0	0	5	0	1	0
PDHB	226	1381	152	896	637	943
	1654	1489	572	361	784	176
PDHX	272	1685	285	1428	697	1032
	1479	1451	669	396	992	255
PDIA2	6	0	2	1	0	0
	0	0	5	0	1	0
PDIA3	92	442	105	287	195	321
	333	393	185	136	244	105
PDIA4	34	132	27	107	62	129
	101	155	82	43	77	24
PDIA5	25	45	15	37	20	25
	21	57	20	12	30	11
PDIA6	87	523	99	443	264	467
	400	573	296	167	324	93
PDIK1L	85	311	75	206	133	220
	207	355	150	77	163	46
PDILT	22	0	11	0	8	0
	0	0	7	0	4	0
PDK1	52	178	61	135	94	184
	223	230	114	61	136	31
PDK2	122	487	112	353	226	479
	680	599	304	214	371	118
PDK3	121	133	41	167	97	178
	117	207	105	46	83	53

PDK4	829	2068	2883	506	1572	1321
	8933	26867	2734	2639	5871	1824
PDLIM1	74	331	54	133	167	136
	305	464	92	131	158	59
PDLIM2	45	87	35	70	33	56
	68	180	48	17	54	7
PDLIM3	5511	22063	4833	17465	12982	19860
	17314	26557	13723	7549	13981	3041
PDLIM4	9	6	1	5	4	5
	2	8	3	3	5	2
PDLIM5	2028	10377	2243	6095	4686	8474
	10272	16892	5323	3614	7308	1534
PDLIM7	95	562	99	382	172	445
	522	301	218	139	211	92
PDP1	402	3227	459	2235	1372	1681
	2933	1915	1050	533	1669	246
PDP2	55	73	26	36	35	27
	59	104	40	23	56	20
PDPK1	126	452	98	374	241	323
	408	463	247	137	250	97
PDPN	47	167	40	146	106	141
	177	142	76	58	70	33
PDPR	216	547	148	530	248	353
	775	907	291	186	252	110
PDRG1	8	43	8	28	14	36
	50	40	18	7	24	6
PDS5A	247	936	254	788	652	888
	785	1151	411	354	583	245
PDS5B	161	732	191	635	358	503
	411	597	286	188	355	121
PDSS1	50	75	23	51	48	42
	100	143	59	22	49	14
PDSS2	41	229	51	149	73	117
	143	213	89	46	111	26
PDX1	2	0	5	0	4	0
	0	0	2	0	3	0
PDXDC1	175	929	190	695	346	806
	592	757	419	253	512	145
PDXK	37	155	27	136	66	74
	139	114	55	22	62	24
PDXP	12	15	4	6	9	11
	13	29	7	6	15	5
PDYN	21	0	16	0	2	0
	0	0	7	0	3	0
PDZD11	31	58	14	60	30	63
	92	103	42	35	53	12
PDZD2	104	192	64	203	143	98
	160	145	118	60	110	60
PDZD3	22	0	10	0	1	0
	1	0	2	1	1	0
PDZD4	13	28	6	13	17	11
	7	8	10	4	25	4
PDZD7	17	1	4	0	3	1
	2	2	9	0	6	0
PDZD8	57	288	73	211	151	226
	206	223	111	83	146	68
PDZD9	3	0	4	1	1	0
	0	2	1	1	1	0

PDZK1	34	3	30	3	13	8
	4	2	17	4	15	0
PDZK1IP1	4	1	4	0	0	2
	0	0	2	0	2	0
PDZRN3	230	1003	181	881	542	921
	932	703	403	349	676	144
PDZRN4	26	9	28	50	12	7
	8	6	14	5	19	6
PEA15	64	249	63	155	130	233
	292	233	110	65	121	37
PEAK1	232	980	209	776	466	602
	642	764	362	256	437	129
PEAR1	40	83	22	71	29	23
	71	67	36	47	53	16
PEBP1	459	1779	269	1484	769	1332
	2083	2920	1071	596	1156	410
PEBP4	203	979	132	1253	421	1037
	1409	1781	860	336	940	184
PECAM1	68	306	41	171	181	147
	234	306	129	82	118	56
PECR	29	33	13	35	31	19
	49	81	24	16	25	9
PEF1	13	40	6	39	21	21
	46	83	35	14	29	10
PEG10	48	145	82	64	56	97
	40	76	43	22	41	14
PEG3	1	3	0	14	2	9
	17	1	1	8	2	4
PELI1	42	167	27	123	76	129
	104	212	69	76	79	32
PELI2	32	101	44	87	49	59
	129	135	70	47	89	26
PELI3	19	45	9	53	26	35
	32	35	19	14	21	7
PELO	13	36	7	29	19	26
	37	33	22	10	25	8
PELP1	52	94	32	58	39	87
	96	104	56	36	63	17
PEMT	9	17	10	20	14	13
	28	34	17	6	15	11
PENK	0	9	6	17	0	9
	8	7	11	0	12	0
PEPD	28	91	24	52	29	60
	68	114	52	26	66	14
PER1	81	468	54	256	235	235
	777	1015	347	145	516	145
PER2	91	261	67	172	198	205
	297	474	212	152	290	108
PER3	202	1120	290	681	614	771
	976	1229	593	353	839	229
PERP	19	33	30	31	14	21
	9	22	16	6	11	6
PES1	32	76	34	61	44	57
	101	160	42	50	45	24
PET100	41	189	23	132	98	139
	287	350	68	39	103	20
PET112	51	273	51	149	101	178
	224	320	128	51	139	41

PET117	9	54	18	24	24	31
	53	43	24	13	31	8
PEX1	155	450	108	322	212	377
	411	606	325	173	344	115
PEX10	27	105	20	69	33	47
	56	75	38	23	37	13
PEX11A	24	78	14	48	26	53
	52	93	39	20	48	13
PEX11B	33	104	28	79	66	96
	108	179	84	28	75	19
PEX11G	1	3	0	4	3	4
	2	6	3	2	6	0
PEX12	29	108	26	69	40	66
	110	116	46	31	75	9
PEX13	139	451	101	375	275	326
	378	711	251	197	326	132
PEX14	31	62	20	39	29	42
	66	54	27	26	37	15
PEX16	14	18	11	23	14	25
	24	49	17	8	25	6
PEX19	133	520	77	341	189	363
	389	558	247	121	282	90
PEX2	119	472	116	463	232	472
	437	600	290	137	309	74
PEX26	46	97	23	79	62	82
	71	133	39	34	48	22
PEX3	55	226	60	164	117	178
	162	283	118	55	173	42
PEX5	92	245	60	239	117	191
	225	259	135	68	161	40
PEX5L	92	32	58	8	18	11
	6	9	33	5	17	7
PEX6	37	130	24	94	42	103
	68	103	43	16	54	35
PEX7	24	71	21	103	31	60
	71	107	51	17	54	15
PF4	1	4	5	2	0	2
	1	2	1	1	0	0
PF4V1	1	1	1	0	0	0
	0	0	1	0	1	0
PFAS	39	63	23	44	30	23
	48	84	37	25	46	11
PFDN1	101	352	61	228	239	260
	276	612	251	149	278	83
PFDN2	41	227	19	103	100	122
	240	218	57	41	92	33
PFDN4	53	232	52	211	108	183
	249	372	111	54	130	40
PFDN5	229	1178	250	1071	495	1055
	1509	2447	870	384	808	167
PFDN6	30	143	27	71	78	107
	111	249	48	38	93	19
PFKFB1	132	919	151	575	383	631
	702	746	368	228	439	116
PFKFB2	371	662	320	326	273	180
	772	2641	432	353	1081	436
PFKFB3	451	222	73	534	233	629
	1415	1206	726	344	440	451

PFKFB4	29	23	11	20	21	17
	26	27	18	5	19	7
PFKL	25	78	24	71	21	37
	61	90	27	23	64	17
PFKM	1793	13284	1891	10593	5212	9417
	10439	9795	6468	3335	7346	1607
PFKP	25	81	25	46	33	25
	31	37	29	16	24	20
PFN1	16	176	24	130	65	112
	125	178	87	36	79	23
PFN2	360	1652	262	1086	867	1184
	1143	2389	632	454	1012	295
PFN3	1	0	0	0	0	0
	0	0	0	0	0	0
PFN4	5	2	3	4	6	2
	4	4	11	1	2	1
PGA3	3	0	3	0	4	1
	0	2	7	0	3	0
PGA4	2	0	0	0	1	0
	0	2	6	0	0	1
PGA5	11	0	2	0	3	0
	0	0	8	0	8	0
PGAM1	34	141	32	96	74	85
	86	154	65	24	68	33
PGAM2	444	2674	267	1824	811	1726
	2906	2433	1433	620	1328	323
PGAM4	6	0	5	1	1	0
	0	0	0	0	2	0
PGAM5	39	152	33	87	57	131
	106	207	70	50	99	26
PGAP1	87	302	109	266	169	241
	150	239	139	53	156	36
PGAP2	31	39	16	27	16	31
	34	41	21	16	26	4
PGAP3	12	30	10	24	9	18
	16	43	21	8	18	3
PGBD1	27	54	20	40	19	34
	41	51	26	14	34	11
PGBD2	16	15	13	10	13	15
	23	39	23	6	8	3
PGBD3	0	0	0	0	0	2
	1	1	1	0	0	0
PGBD4	25	45	15	39	30	27
	16	62	25	15	17	3
PGBD5	25	15	13	8	15	14
	6	16	22	3	19	3
PGC	21	0	8	0	1	0
	0	0	7	1	3	0
PGD	40	137	35	95	49	73
	90	79	68	31	49	33
PGF	11	7	1	10	10	10
	12	12	13	4	4	1
PGGT1B	78	320	84	251	165	239
	274	375	170	101	232	50
PGK1	452	2890	458	3219	1094	2537
	2937	2345	1616	579	1354	282
PGK2	7	0	3	0	1	0
	0	0	6	0	3	0

PGLS	7	18	5	9	5	5
	9	28	15	5	20	3
PGLYRP1	3	0	1	0	0	0
	0	0	0	0	0	0
PGLYRP2	8	0	4	0	2	0
	0	0	2	0	1	0
PGLYRP3	7	0	5	0	2	0
	0	0	6	0	5	0
PGLYRP4	9	0	10	0	1	0
	0	0	7	0	3	0
PGM1	1273	8357	1433	7493	3139	6278
	6094	5941	4143	1721	4104	1038
PGM2	23	34	19	20	21	49
	27	57	23	10	11	13
PGM2L1	132	409	97	320	347	291
	397	394	219	137	219	71
PGM3	123	228	88	159	114	131
	139	219	133	70	132	31
PGM5	125	462	139	179	231	245
	222	591	190	174	322	123
PGP	22	124	17	105	48	96
	123	160	90	34	57	21
PGPEP1	99	257	61	149	128	186
	182	223	166	67	172	60
PGPEP1L	46	165	11	64	55	84
	181	240	111	73	134	32
PGR	65	102	87	68	57	57
	48	44	95	15	65	27
PGRMC1	27	177	26	122	76	128
	132	166	93	38	86	34
PGRMC2	51	407	65	317	136	258
	292	249	100	45	116	35
PGS1	31	19	8	22	23	22
	35	74	36	10	20	11
PHACTR1	23	13	13	11	11	7
	11	16	23	2	15	5
PHACTR2	268	862	166	701	415	740
	779	841	513	306	517	201
PHACTR3	28	1	25	7	6	3
	2	5	17	0	11	2
PHACTR4	95	307	70	143	149	174
	227	215	131	80	125	43
PHAX	82	350	57	208	193	246
	229	329	128	133	207	65
PHB	112	478	77	299	183	250
	437	665	286	155	282	106
PHB2	172	787	154	579	328	751
	992	1199	530	319	542	172
PHC1	84	150	53	146	65	128
	125	136	94	70	120	42
PHC2	65	165	47	159	102	145
	189	266	117	83	131	44
PHC3	348	1451	352	1169	745	1029
	988	1649	688	461	882	302
PHEX	23	1	17	3	3	5
	3	3	11	0	3	0
PHF1	47	100	33	83	46	77
	101	146	62	30	55	19

PHF10	83	466	64	357	230	320
	342	393	194	118	188	68
PHF11	38	132	43	122	65	99
	84	126	69	43	69	23
PHF12	38	127	38	73	41	64
	104	81	43	43	49	17
PHF13	16	62	25	27	20	41
	34	58	16	17	26	5
PHF14	140	417	132	339	184	329
	282	435	252	128	266	92
PHF15	40	99	42	84	71	113
	80	121	46	33	69	23
PHF16	25	36	15	13	22	22
	16	29	21	5	10	4
PHF17	107	498	157	519	284	411
	332	339	224	84	186	58
PHF19	28	84	19	46	38	51
	52	88	33	13	41	11
PHF2	100	330	98	303	184	244
	252	329	159	96	174	65
PHF20	166	870	188	764	570	847
	687	811	367	288	408	127
PHF20L1	225	899	242	606	452	745
	760	1015	498	281	566	151
PHF21A	74	290	71	227	137	178
	176	218	129	60	106	42
PHF21B	17	5	13	0	2	5
	0	0	14	0	2	7
PHF23	27	62	12	30	33	47
	48	69	26	20	31	12
PHF3	344	1759	433	1036	939	1170
	1190	2025	704	650	971	360
PHF5A	36	116	34	106	67	103
	146	277	75	43	96	12
PHF6	49	226	61	169	119	153
	153	237	109	62	111	42
PHF7	31	42	28	39	23	43
	63	75	35	21	32	10
PHF8	89	186	62	125	113	112
	118	238	106	71	115	47
PHGDH	20	24	17	29	17	20
	5	10	18	1	17	11
PHGR1	1	0	5	0	1	0
	0	1	0	0	1	0
PHIP	412	1624	520	1322	827	1398
	1232	2358	892	628	1143	416
PHKA1	373	2468	375	1831	899	1641
	2367	2106	1308	710	1327	424
PHKA2	94	344	65	116	123	151
	208	246	66	52	95	38
PHKB	550	3955	506	3100	1496	2429
	2460	2282	1235	731	1489	405
PHKG1	314	1251	273	1172	644	1381
	1170	1675	812	501	899	356
PHKG2	33	12	16	18	22	14
	14	62	25	9	27	10
PHLDA1	39	47	22	38	10	24
	14	17	14	6	10	7

PHLDA2	0	0	1	0	0	0
	0	0	2	0	0	0
PHLDA3	11	51	14	42	15	24
	26	78	41	18	19	16
PHLDB1	135	597	118	462	248	388
	404	452	208	123	261	139
PHLDB2	119	281	155	446	142	162
	280	266	120	157	200	84
PHLDB3	12	12	10	7	4	5
	7	12	14	2	7	0
PHLPP1	48	138	43	93	55	95
	95	114	84	37	80	27
PHLPP2	87	95	55	54	73	63
	84	105	67	22	71	31
PHOSPHO1	7	8	7	2	4	2
	0	6	12	2	4	1
PHOSPHO2	1	6	3	10	8	8
	12	8	2	1	7	3
PHOSPHO2-KLHL23		71	389	79	184	226
	266	249	432	137	92	205
	65					
PHOX2A	6	0	3	0	0	0
	0	0	2	0	0	0
PHOX2B	21	0	9	0	3	0
	0	0	9	0	0	0
PHPT1	70	256	33	121	99	142
	281	342	137	66	153	36
PHRF1	78	258	83	165	152	197
	206	362	111	118	182	59
PHTF1	38	73	21	35	25	37
	37	54	53	20	41	14
PHTF2	705	3245	528	2828	2064	2278
	2683	3382	1781	934	2246	531
PHYH	234	1428	376	1207	687	1472
	1907	1858	1102	613	1603	319
PHYHD1	7	31	9	15	15	14
	51	34	15	10	11	5
PHYHIP	19	3	12	0	1	2
	0	2	3	0	5	1
PHYHIPL	19	9	15	11	9	10
	1	10	5	0	15	3
PI15	54	28	18	23	22	38
	24	34	25	5	37	8
PI16	23	43	15	64	42	32
	15	80	24	9	29	19
PI3	1	0	2	0	0	0
	3	0	0	0	1	0
PI4K2A	33	41	18	51	34	42
	50	73	33	13	41	16
PI4K2B	37	65	33	74	47	55
	53	64	48	39	35	12
PI4KA	115	372	93	246	172	285
	289	312	193	99	205	68
PI4KB	93	324	87	280	158	281
	282	360	174	105	202	64
PIANP	6	0	4	0	2	0
	0	0	3	1	1	0

PIAS1	72	331	69	197	153	196
	250	373	138	108	212	68
PIAS2	181	842	195	601	427	691
	605	960	456	247	487	141
PIAS3	52	73	29	67	32	61
	61	71	40	19	60	10
PIAS4	15	43	11	34	33	31
	55	47	22	12	27	6
PIBF1	60	300	79	192	125	200
	219	319	161	103	184	46
PICALM	237	1335	289	915	620	875
	1138	1384	619	361	722	262
PICK1	22	89	23	54	34	77
	83	94	45	34	64	9
PID1	28	75	29	96	44	114
	89	123	58	26	57	13
PIDD	7	26	7	20	10	28
	29	28	13	8	20	2
PIEZO1	33	85	27	29	41	30
	36	59	34	22	67	20
PIEZO2	119	28	63	20	36	20
	13	12	75	6	40	3
PIF1	12	1	9	2	1	5
	3	1	4	4	8	3
PIFO	9	1	13	7	2	7
	3	3	7	1	6	0
PIGA	30	61	22	31	33	38
	49	93	32	17	51	8
PIGB	18	34	17	31	16	23
	38	27	30	9	21	9
PIGC	27	69	19	50	33	49
	50	99	42	28	45	14
PIGF	56	124	46	139	76	112
	126	213	83	47	92	31
PIGG	39	101	43	100	46	66
	105	132	68	28	92	17
PIGH	14	78	18	78	36	88
	64	118	57	27	43	17
PIGK	87	365	90	301	167	300
	262	416	190	116	202	51
PIGL	27	55	16	40	19	41
	52	94	42	20	42	9
PIGM	20	45	26	51	19	43
	38	49	35	13	27	5
PIGN	128	407	116	298	186	276
	328	550	241	102	269	92
PIGO	42	57	25	30	30	47
	34	75	39	21	49	21
PIGP	28	113	13	65	46	70
	96	140	46	23	66	23
PIGQ	11	15	7	13	15	11
	5	33	11	8	16	7
PIGR	22	1	11	0	4	0
	0	1	12	0	2	0
PIGS	43	140	35	120	52	103
	105	165	84	36	95	31
PIGT	50	183	41	113	64	163
	126	225	105	68	120	42

PIGU	39	91	28	64	45	104
	89	143	63	53	85	19
PIGV	31	50	18	36	17	46
	33	63	40	18	38	4
PIGW	15	46	15	27	27	30
	64	40	21	8	30	3
PIGX	49	149	22	91	95	101
	160	151	57	46	78	23
PIGY	39	213	44	179	96	202
	299	280	127	43	120	40
PIGZ	12	26	6	19	17	31
	17	27	19	11	11	7
PIH1D1	49	167	29	128	63	118
	155	166	89	58	111	37
PIH1D2	10	9	5	2	3	5
	6	10	10	2	7	0
PIH1D3	17	0	4	0	5	0
	0	0	2	0	2	0
PIK3AP1	42	32	23	17	11	35
	10	37	28	10	27	5
PIK3C2A	169	667	166	486	319	520
	617	842	341	252	464	168
PIK3C2B	170	552	139	465	298	357
	456	330	251	132	226	73
PIK3C2G	51	0	44	0	18	0
	1	2	28	0	12	0
PIK3C3	137	502	113	343	216	321
	368	476	248	142	279	81
PIK3CA	123	511	125	401	276	362
	414	632	269	152	371	131
PIK3CB	127	564	129	598	217	389
	450	446	259	98	229	50
PIK3CD	24	27	12	15	16	21
	16	19	17	10	14	2
PIK3CG	42	25	22	27	19	31
	17	28	23	13	19	3
PIK3IP1	58	138	35	119	63	89
	169	355	156	60	119	45
PIK3R1	369	1062	265	1825	1032	1443
	1146	1978	831	386	725	332
PIK3R2	29	117	43	102	37	82
	64	76	41	40	38	12
PIK3R3	69	283	66	236	76	154
	248	195	105	57	115	27
PIK3R4	91	326	86	254	149	222
	245	406	196	108	234	54
PIK3R5	29	16	15	8	7	10
	6	22	18	1	12	4
PIK3R6	25	6	10	5	8	1
	4	7	14	3	6	1
PIKFYVE	273	1147	283	794	598	746
	710	1094	563	316	659	192
PILRA	15	14	10	4	3	8
	10	20	13	6	22	2
PILRB	74	275	34	92	65	110
	169	258	76	62	155	48
PIM1	21	98	20	97	33	73
	150	62	27	39	31	11

PIM2	12	3	3	11	6	12
	6	21	10	5	9	3
PIM3	22	71	18	51	35	59
	118	158	56	31	55	13
PIN1	43	164	28	129	74	126
	176	288	102	41	103	34
PIN4	60	198	27	102	95	144
	218	396	113	58	109	28
PINK1	335	1732	286	1825	689	1760
	1628	1880	924	458	1049	344
PINLYP	5	2	4	3	2	2
	0	3	0	0	3	2
PINX1	12	63	12	40	26	49
	47	70	31	15	35	9
PION	54	84	69	111	43	82
	85	136	79	33	97	14
PIP	5	0	3	0	1	0
	0	0	2	0	0	0
PIP4K2A	38	167	30	73	42	95
	84	120	44	31	64	28
PIP4K2B	290	1354	252	939	667	986
	1032	1150	497	422	741	200
PIP4K2C	43	75	37	76	42	61
	68	75	50	18	56	11
PIP5K1A	95	432	98	315	206	327
	415	489	254	133	286	99
PIP5K1B	30	54	53	70	29	54
	26	53	25	16	32	4
PIP5K1C	26	89	14	29	31	36
	51	44	31	23	24	20
PIP5KL1	6	0	4	0	0	0
	1	0	4	0	3	0
PIPOX	16	3	8	4	7	2
	6	1	6	1	10	2
PIR	34	77	15	77	61	70
	53	144	42	21	58	23
PIRT	17	0	5	0	3	0
	0	1	5	0	1	0
PISD	34	89	18	48	34	65
	70	134	47	33	46	19
PITHD1	74	321	73	267	179	253
	344	405	155	101	189	59
PITPNA	49	169	26	88	76	87
	119	106	43	55	75	32
PITPNB	52	248	51	159	101	150
	164	218	85	58	109	37
PITPNC1	44	45	19	30	29	36
	82	107	44	28	56	17
PITPNM1	20	14	6	10	9	8
	14	9	14	5	13	7
PITPNM2	33	56	23	26	21	36
	51	62	28	10	37	10
PITPNM3	21	10	11	7	13	6
	2	4	13	3	16	2
PITRM1	153	757	155	431	394	568
	529	652	281	212	357	125
PITX1	8	20	3	4	15	7
	3	4	2	0	1	1

PITX2	48	180	61	166	102	170
	278	205	131	78	157	58
PITX3	8	8	2	6	6	7
	24	20	12	8	8	7
PIWIL1	33	0	18	0	11	0
	0	0	21	0	8	0
PIWIL2	36	52	23	17	16	91
	53	44	27	1	30	15
PIWIL3	46	0	17	0	8	0
	0	0	18	0	10	0
PIWIL4	41	20	29	13	10	16
	9	8	23	8	23	4
PJA1	23	114	20	69	51	82
	60	74	42	23	37	24
PJA2	318	1968	438	1495	895	1363
	1408	2067	770	562	1017	351
PKD1	287	1248	248	643	457	796
	652	1197	498	309	493	240
PKD1L1	123	170	72	171	115	105
	211	179	194	98	145	41
PKD1L2	47	30	19	24	11	8
	11	23	26	6	24	3
PKD1L3	52	1	39	2	10	7
	1	5	20	3	13	1
PKD2	59	195	35	156	89	167
	146	209	76	61	102	55
PKD2L1	13	1	8	0	5	1
	0	0	6	0	7	0
PKD2L2	33	25	17	21	20	39
	10	41	28	11	21	8
PKDCC	91	412	99	463	150	417
	214	288	153	103	125	65
PKDREJ	25	2	26	9	12	0
	7	2	15	0	12	1
PKHD1	143	7	109	3	50	12
	7	18	70	2	57	1
PKHD1L1	124	7	77	18	66	7
	5	39	71	4	53	25
PKIA	2273	11637	1945	10388	6294	8637
	10509	13140	5350	2860	8282	1857
PKIB	11	12	14	1	12	2
	7	10	9	4	12	2
PKIG	43	136	26	95	63	92
	173	202	103	48	112	24
PKLR	17	0	5	0	0	0
	0	0	7	0	2	0
PKM	1309	9711	1693	8986	3836	7688
	10111	5995	4549	2410	4890	1119
PKMYT1	3	1	7	1	0	1
	0	1	5	1	3	0
PKN1	55	188	22	111	83	118
	135	167	76	51	123	40
PKN2	158	735	183	580	373	452
	553	793	321	208	367	108
PKN3	18	20	9	6	9	5
	29	27	17	8	17	3
PKNOX1	53	139	31	88	93	96
	115	168	67	37	82	25

PKNOX2	36	86	28	48	51	56
	76	116	56	40	61	26
PKP1	19	34	27	18	17	12
	30	18	25	6	22	3
PKP2	49	123	53	196	23	100
	144	109	66	14	59	18
PKP3	6	0	5	1	2	0
	0	0	0	0	0	0
PKP4	110	337	77	205	167	202
	222	312	144	105	141	61
PLA1A	24	9	19	4	7	8
	7	17	16	12	13	3
PLA2G10	14	3	5	0	5	3
	0	0	7	0	0	1
PLA2G12A	123	622	124	403	211	347
	519	706	235	148	338	114
PLA2G12B	4	1	6	0	0	0
	0	0	4	0	4	0
PLA2G15	28	79	24	53	51	71
	54	103	54	29	40	20
PLA2G16	53	225	41	286	150	238
	314	291	150	63	162	63
PLA2G1B	3	0	3	0	0	0
	0	0	1	0	0	0
PLA2G2A	14	2	8	1	5	0
	1	42	2	1	2	5
PLA2G2C	3	0	2	0	0	0
	0	0	2	0	0	0
PLA2G2D	11	0	3	0	1	0
	0	1	6	0	1	0
PLA2G2E	1	0	3	0	2	1
	0	0	5	0	2	0
PLA2G2F	8	0	0	1	1	0
	0	0	4	0	0	0
PLA2G3	9	0	8	0	1	0
	0	0	3	0	3	0
PLA2G4A	35	23	21	18	18	16
	5	27	37	3	22	5
PLA2G4B	7	8	4	2	6	2
	3	10	6	3	5	1
PLA2G4C	174	839	219	501	370	510
	625	819	451	316	624	136
PLA2G4D	24	0	7	0	6	0
	0	0	8	0	9	0
PLA2G4E	36	9	12	0	8	2
	2	7	18	8	13	0
PLA2G4F	25	38	20	31	10	28
	36	39	35	6	40	11
PLA2G5	14	0	7	1	4	1
	0	5	5	0	3	1
PLA2G6	29	32	24	15	24	13
	34	45	33	15	32	7
PLA2G7	7	2	9	0	1	0
	0	1	5	0	9	0
PLA2R1	88	52	64	126	76	77
	54	67	78	35	52	15
PLAA	142	654	135	514	311	392
	472	673	309	217	399	121

PLAC1	3	0	1	0	2	0
	0	0	2	0	0	0
PLAC1L	5	0	4	0	3	0
	0	0	6	0	3	0
PLAC4	121	8	54	6	31	10
	0	17	51	2	29	1
PLAC8	9	2	6	1	1	4
	5	5	8	1	7	0
PLAC8L1	4	5	5	4	0	8
	1	1	9	3	7	0
PLAC9	1	19	3	22	9	23
	4	34	9	1	6	1
PLAG1	42	49	31	24	24	60
	39	157	46	12	76	18
PLAGL1	25	54	21	45	40	54
	54	77	32	14	39	14
PLAGL2	59	217	51	132	69	121
	108	100	73	23	95	21
PLAT	22	80	16	59	35	44
	75	94	31	16	46	23
PLAU	27	66	17	44	36	64
	40	80	33	23	34	5
PLAUR	9	6	5	5	5	3
	5	3	10	3	7	2
PLB1	66	20	37	35	24	18
	6	32	37	11	47	3
PLBD1	28	94	26	60	52	60
	71	132	72	43	69	19
PLBD2	34	117	20	82	34	81
	72	106	44	16	50	14
PLCB1	171	667	156	480	389	437
	633	595	223	272	391	105
PLCB2	31	28	25	14	13	16
	12	24	15	4	15	2
PLCB3	16	42	16	44	35	29
	28	53	37	23	29	5
PLCB4	110	180	53	209	104	124
	251	181	133	88	191	60
PLCD1	19	22	15	20	15	19
	15	26	21	4	31	3
PLCD3	67	360	61	225	108	207
	239	191	115	107	181	60
PLCD4	320	1416	242	1042	724	1232
	916	1192	576	251	543	146
PLCE1	107	347	234	216	75	260
	167	132	101	76	121	60
PLCG1	93	309	85	203	143	212
	203	298	150	73	169	49
PLCG2	55	57	21	28	24	12
	67	54	44	18	20	4
PLCH1	44	13	37	22	15	21
	77	8	38	1	39	1
PLCH2	6	1	13	0	4	0
	0	2	11	0	2	0
PLCL1	265	1069	246	1065	683	842
	874	929	423	444	485	157
PLCL2	207	1049	249	786	604	756
	1007	1064	474	335	711	214

PLCXD1 (NC_000023 192990..220023)	13	26	6	13	1	6	7	34	21	3	17	5
PLCXD1 (NC_000024 142990..170023)	9	12	9	16	10	3	3	14	10	6	31	0
PLCXD2	11	4	9	7	4	2	6	3	10	5	8	0
PLCXD3	37	11	21	22	18	13	8	6	25	5	18	9
PLCZ1	10	0	13	0	8	0	0	0	13	0	11	0
PLD1	71	130	50	76	94	81	100	181	103	52	84	44
PLD2	22	48	23	18	15	28	35	39	38	21	33	6
PLD3	35	127	15	73	61	77	93	127	62	43	69	18
PLD4	5	0	3	1	1	0	0	0	0	0	0	0
PLD5	35	5	19	10	6	8	10	2	19	4	17	2
PLD6	19	38	7	11	22	32	15	36	13	10	17	6
PLEC	946	6041	885	3324	1776	2539	3220	4855	1746	1723	2727	1121
PLEK	28	32	26	33	24	24	32	49	15	7	21	7
PLEK2	8	2	7	1	2	1	0	0	9	0	4	0
PLEKHA1	80	318	70	248	170	224	216	423	164	109	162	75
PLEKHA2	53	148	85	141	85	93	77	180	78	54	87	44
PLEKHA3	46	120	46	123	88	126	150	175	71	39	100	21
PLEKHA4	35	26	14	21	11	17	16	23	14	5	17	11
PLEKHA5	112	135	60	106	73	77	63	98	81	43	74	25
PLEKHA6	49	118	43	45	59	87	34	113	55	33	60	16
PLEKHA7	31	20	23	14	19	15	19	27	20	9	26	6
PLEKHA8	63	182	64	134	67	116	122	128	88	38	92	31
PLEKHB1	24	21	8	18	23	13	17	20	14	10	24	5
PLEKHB2	77	222	64	229	146	191	243	253	164	75	163	48
PLEKHD1	28	0	6	0	1	0	0	2	6	0	1	0
PLEKHF1	36	82	11	58	76	59	95	188	61	26	68	36
PLEKHF2	55	208	45	250	69	136	126	165	93	45	108	47
PLEKHG1	57	102	44	58	44	46	111	141	83	42	70	27

PLEKHG2	44	31	17	23	15	22
	20	19	29	10	19	2
PLEKHG3	29	65	19	32	42	40
	38	55	33	27	31	22
PLEKHG4	22	6	9	15	4	4
	6	14	16	5	11	1
PLEKHG4B	64	1	26	1	9	1
	0	1	25	0	13	0
PLEKHG5	25	69	25	32	35	46
	37	59	31	18	38	14
PLEKHG6	21	2	9	0	3	1
	0	0	5	0	4	1
PLEKHG7	35	0	17	1	7	1
	0	0	17	3	4	0
PLEKHH1	37	53	31	15	25	22
	30	16	29	10	36	10
PLEKHH2	69	95	58	84	54	58
	51	75	57	23	71	23
PLEKHH3	50	151	59	188	84	131
	62	64	53	26	50	27
PLEKHJ1	15	83	12	39	33	42
	62	96	39	23	42	14
PLEKHM1	44	119	23	70	44	55
	77	114	62	40	73	32
PLEKHM2	109	682	129	420	266	546
	404	611	260	192	299	101
PLEKHM3	88	262	67	236	126	176
	226	339	180	70	176	62
PLEKHN1	5	0	9	0	4	1
	0	2	5	0	3	0
PLEKHO1	23	129	23	65	39	102
	122	117	48	29	68	18
PLEKHO2	31	32	20	36	22	38
	31	44	30	9	22	11
PLEKHS1	15	0	15	0	8	0
	0	1	11	0	9	0
PLG	46	1	22	3	11	4
	2	2	27	0	12	0
PLGLB1	63	60	20	87	21	75
	43	120	66	25	64	20
PLGLB2	9	57	35	11	25	58
	45	4	11	10	33	0
PLGRKT	37	195	32	143	69	100
	202	227	92	40	106	24
PLIN1	14	102	33	51	143	34
	76	119	11	18	26	180
PLIN2	193	743	88	539	229	423
	734	1257	360	262	604	162
PLIN3	61	117	39	87	45	88
	105	115	75	27	69	22
PLIN4	619	1892	339	1285	1354	1531
	1227	2780	631	752	792	654
PLIN5	132	557	22	123	275	258
	219	943	169	88	198	59
PLK1	14	9	10	6	12	14
	4	9	5	3	4	6
PLK1S1	83	264	68	174	111	218
	213	322	143	103	219	50

PLK2	45	204	58	198	68	95
	118	114	63	28	69	20
PLK3	15	23	9	11	15	18
	4	12	12	6	12	5
PLK4	33	7	21	6	10	5
	3	7	16	0	10	0
PLK5	11	0	4	0	0	0
	0	0	0	0	1	0
PLLP	8	4	2	3	3	5
	8	3	7	2	5	1
PLN	2674	10608	1576	4975	5625	7630
	8860	16132	3114	3741	7791	2062
PLOD1	61	164	40	171	123	179
	172	193	110	72	97	41
PLOD2	74	94	31	75	65	81
	106	133	88	45	53	36
PLOD3	29	36	11	22	18	18
	22	34	15	14	13	9
PLP1	22	15	15	32	11	18
	19	11	13	10	15	17
PLP2	17	41	17	18	30	44
	47	56	13	22	24	5
PLRG1	105	392	104	295	180	290
	335	469	198	123	291	63
PLS1	33	14	23	13	14	17
	9	17	33	10	11	1
PLS3	124	610	113	487	271	361
	342	685	314	199	289	122
PLSCR1	21	104	19	78	45	74
	85	158	80	52	77	25
PLSCR2	23	0	7	0	11	0
	0	1	12	0	7	0
PLSCR3	2	35	4	16	18	10
	25	13	9	2	11	4
PLSCR4	48	160	57	194	59	146
	131	226	105	43	116	46
PLSCR5	19	0	10	0	7	0
	0	0	8	0	3	0
PLTP	26	36	35	46	44	48
	22	88	35	6	28	10
PLVAP	14	99	20	36	22	35
	70	107	26	25	26	19
PLXDC1	68	185	36	104	94	127
	99	137	92	35	78	24
PLXDC2	60	365	83	364	183	273
	297	480	205	81	220	65
PLXNA1	94	298	87	250	159	211
	213	261	115	86	127	40
PLXNA2	107	343	101	167	149	230
	231	257	162	95	182	46
PLXNA3	29	51	15	14	13	22
	17	24	14	6	15	10
PLXNA4	104	77	72	20	110	20
	57	61	58	26	57	36
PLXNB1	39	31	29	25	10	13
	13	28	20	3	22	15
PLXNB2	23	95	54	66	34	47
	37	49	15	11	37	26

PLXNB3	12	0	6	4	6	0
	2	0	4	0	0	0
PLXNC1	130	129	77	145	130	176
	88	117	82	73	145	42
PLXND1	68	334	51	217	117	172
	218	296	134	79	131	79
PM20D1	18	4	5	3	6	4
	8	5	17	0	3	2
PM20D2	206	1008	327	709	446	814
	568	1397	513	235	567	188
PMAIP1	5	10	9	12	1	12
	12	6	17	3	2	1
PMCH	6	0	5	0	1	0
	0	0	4	0	0	0
PMEL	15	2	13	0	3	1
	0	0	9	0	5	0
PMEPA1	38	200	27	240	147	154
	181	72	65	40	46	46
PMF1	21	153	34	153	101	166
	228	273	127	70	133	46
PMF1-BGLAP	34	142	27	136	58	136
	158	218	123	56	119	29
PMFBP1	52	0	26	0	9	0
	3	2	23	0	9	0
PML	40	75	22	49	36	45
	55	42	31	19	35	16
PMM1	6	8	4	11	4	8
	13	11	14	5	8	1
PMM2	38	88	18	50	33	61
	65	112	44	24	57	18
PMP2	31	4	10	22	6	0
	1	6	18	4	7	30
PMP22	55	241	72	301	156	272
	342	392	173	170	205	108
PMPCA	33	285	47	158	145	173
	254	309	151	96	185	49
PMPCB	150	695	127	507	285	513
	581	747	324	233	470	110
PMS1	63	187	59	187	91	136
	149	226	87	59	154	31
PMS2	69	193	67	139	100	152
	180	217	101	60	116	24
PMVK	12	46	8	54	23	24
	44	48	26	12	22	5
PNCK	6	1	4	3	0	1
	2	2	4	0	3	4
PNISR	433	2102	512	1354	1070	1782
	1674	2310	913	858	1241	435
PNKD	26	73	21	81	47	85
	107	132	72	28	65	22
PNKP	13	29	14	27	17	22
	24	22	17	15	18	6
PNLDC1	18	7	12	6	15	7
	6	6	11	2	6	0
PNLIP	16	0	10	0	6	0
	0	0	8	0	4	0
PNLIPRP1	12	0	6	0	2	0
	0	0	7	0	4	0

PNLIPRP2	13	0	6	0	1	0
	0	0	7	0	8	0
PNLIPRP3	19	0	7	0	6	0
	0	0	9	0	5	1
PNMA1	14	51	17	37	22	37
	42	47	16	9	22	16
PNMA2	26	4	16	10	7	1
	1	4	9	1	3	2
PNMA3	16	2	1	0	1	0
	0	1	2	0	1	1
PNMA5	10	0	7	0	3	0
	0	0	5	0	1	0
PNMA6A	0	0	0	1	0	0
	0	0	1	0	0	0
PNMA6B	0	0	0	1	0	0
	0	0	1	0	0	0
PNMA6C	3	0	2	0	0	1
	0	0	0	0	0	0
PNMA6D	2	0	1	0	1	0
	0	0	2	0	0	0
PNMAL1	25	51	22	45	15	38
	9	15	40	9	38	4
PNMAL2	1	0	0	0	0	0
	0	0	0	0	0	0
PNMT	4	10	1	7	5	7
	6	17	9	1	4	1
PNN	127	601	124	311	269	427
	420	609	205	214	262	107
PNO1	33	120	22	86	46	69
	89	125	69	43	53	20
PNOC	0	1	6	0	3	0
	0	0	1	0	5	0
PNP	20	30	15	14	12	9
	22	27	16	2	16	0
PNPLA1	14	0	13	0	4	1
	0	0	6	1	5	0
PNPLA2	50	215	26	96	83	99
	153	196	78	46	83	44
PNPLA3	15	13	18	2	5	7
	0	2	10	0	3	3
PNPLA4	58	324	33	185	94	136
	269	444	182	77	165	45
PNPLA5	11	0	10	0	0	0
	0	0	3	0	1	0
PNPLA6	47	149	31	102	56	96
	87	117	91	23	76	25
PNPLA7	63	146	21	89	69	106
	130	204	69	53	101	37
PNPLA8	148	513	115	372	298	314
	404	688	232	149	314	117
PNPO	89	316	42	191	153	263
	236	275	142	72	161	62
PNPT1	186	523	126	388	276	447
	448	716	291	207	369	86
PNRC1	222	1907	345	1399	535	1097
	1332	1230	701	459	739	293
PNRC2	184	1007	191	652	492	649
	531	1166	381	255	604	142

POC1A	17	2	12	0	5	2
	2	4	5	1	5	0
POC1B	33	79	26	69	39	53
	55	96	49	12	46	7
POC1B-GALNT4		0	0	0	0	0
	1	0	0	0	0	0
	0					
POC5	22	69	21	54	26	47
	38	67	47	15	43	12
PODN	36	170	51	156	62	94
	110	207	75	62	78	40
PODNL1	10	2	4	2	1	1
	4	1	5	2	2	1
PODXL	91	634	95	315	180	218
	526	414	197	174	276	78
PODXL2	10	25	10	6	5	15
	14	9	24	5	12	11
POF1B	39	10	8	4	10	1
	0	4	9	0	7	0
POFUT1	64	270	71	198	143	200
	214	292	166	74	133	55
POFUT2	20	66	25	39	25	40
	42	87	32	17	41	13
POGK	55	150	54	141	85	121
	134	183	57	39	77	20
POGLUT1	36	56	29	40	30	44
	37	51	39	16	31	13
POGZ	158	615	138	377	260	354
	506	564	239	185	332	130
POLA1	128	374	101	228	203	222
	208	438	172	121	240	66
POLA2	26	35	24	26	26	18
	39	43	29	19	34	11
POLB	93	643	147	534	199	569
	490	517	278	93	305	55
POLD1	19	9	11	14	7	8
	8	29	12	9	9	3
POLD2	42	154	31	75	77	99
	125	192	82	39	70	33
POLD3	43	78	33	54	45	64
	60	92	44	36	58	21
POLD4	24	70	11	58	25	51
	90	135	42	26	56	13
POLDIP2	142	804	123	670	370	542
	836	995	415	206	538	129
POLDIP3	64	202	51	142	86	180
	146	234	99	60	143	26
POLE	108	351	127	200	159	213
	241	423	250	105	219	87
POLE2	12	9	7	2	5	5
	6	6	9	3	9	2
POLE3	77	372	70	204	178	335
	359	495	186	116	218	76
POLE4	12	52	9	43	37	67
	61	104	39	29	38	7
POLG	71	235	47	167	132	175
	176	294	138	103	153	43

POLG2	21	69	17	33	36	30
	49	50	32	21	43	12
POLH	77	125	47	94	73	98
	107	129	64	57	83	22
POLI	52	241	54	163	113	169
	140	211	110	56	103	38
POLK	129	561	150	421	304	458
	448	637	261	197	358	90
POLL	33	83	26	74	40	75
	90	101	62	26	65	20
POLM	19	38	17	38	22	39
	46	63	27	15	33	13
POLN	46	16	37	36	20	19
	21	46	29	12	21	5
POLQ	72	9	40	19	29	26
	11	26	54	10	33	5
POLR1A	120	286	114	228	141	223
	226	278	190	88	170	68
POLR1B	69	174	49	146	92	153
	162	247	117	65	141	26
POLR1C	13	47	10	30	19	38
	69	70	29	17	43	13
POLR1D	52	218	37	136	94	141
	307	232	87	68	103	47
POLR1E	49	148	27	84	51	98
	75	142	76	41	78	35
POLR2A	115	581	152	458	221	319
	430	363	188	143	251	100
POLR2B	285	1452	345	1156	780	1108
	1130	1674	749	491	1018	297
POLR2C	97	312	50	226	125	278
	262	470	167	85	194	49
POLR2D	37	257	41	191	111	168
	172	191	96	72	113	46
POLR2E	47	205	31	144	45	112
	123	182	91	40	109	34
POLR2F	45	200	24	119	94	114
	291	223	85	47	86	23
POLR2G	18	50	17	46	22	50
	74	105	54	17	49	18
POLR2H	21	78	20	66	39	59
	66	131	34	21	46	13
POLR2I	70	385	70	325	140	288
	516	566	250	110	270	58
POLR2J	76	547	84	324	223	351
	731	556	253	130	221	82
POLR2J2	52	89	6	32	92	81
	74	103	53	57	87	11
POLR2J3	75	134	23	45	77	152
	126	205	96	63	111	44
POLR2K	55	298	57	159	124	205
	310	462	142	86	178	37
POLR2L	38	151	9	68	52	78
	195	180	56	36	86	10
POLR2M	27	479	34	491	198	332
	519	887	215	206	305	108
POLR3A	110	327	108	247	162	208
	298	363	177	99	220	70

POLR3B	71	240	69	147	121	158
	172	169	113	49	103	40
POLR3C	50	126	44	84	56	98
	111	141	73	44	76	26
POLR3D	54	187	50	109	105	132
	148	244	98	84	117	41
POLR3E	96	354	68	255	172	305
	244	474	187	150	219	96
POLR3F	59	168	33	110	80	125
	161	211	93	53	103	29
POLR3G	24	31	22	39	45	34
	23	40	25	6	37	4
POLR3GL	53	288	42	167	117	211
	264	262	97	66	105	38
POLR3H	52	173	46	91	73	149
	111	164	59	53	82	25
POLR3K	22	99	5	41	28	56
	118	92	41	19	47	6
POLRMT	51	141	35	79	68	100
	96	242	85	54	109	34
POM121	138	311	89	149	160	187
	210	244	122	117	128	71
POM121C	57	196	54	114	75	111
	164	148	83	61	80	39
POM121L12	3	0	0	0	1	0
	0	0	1	0	0	0
POM121L2	18	0	8	0	4	1
	0	1	11	0	1	0
POMC	7	2	6	4	3	1
	15	9	8	0	1	0
POMGNT1	66	270	40	177	118	203
	208	248	154	77	144	39
POMP	146	639	84	548	277	454
	720	948	362	198	465	98
POMT1	63	206	63	159	109	159
	166	302	120	90	137	46
POMT2	40	70	31	45	22	50
	53	62	44	23	43	14
POMZP3	25	87	12	51	19	70
	41	47	20	16	34	6
PON1	18	0	8	0	3	0
	0	0	8	1	4	0
PON2	43	126	27	125	71	135
	115	134	83	49	63	14
PON3	11	3	12	13	5	3
	8	5	8	3	14	0
POP1	31	76	33	35	41	48
	47	88	52	13	38	10
POP4	37	109	27	96	62	102
	104	230	86	34	74	25
POP5	7	31	5	37	14	28
	42	56	11	6	18	6
POP7	9	98	11	83	29	45
	100	125	51	20	47	15
POPDC2	56	243	49	124	113	218
	228	247	98	102	122	40
POPDC3	206	1043	146	714	505	972
	905	1330	482	340	585	137

POR	14	56	10	34	30	46
	42	88	26	18	31	10
PORCN	15	10	7	9	12	10
	10	27	13	5	15	4
POSTN	41	36	39	36	38	14
	20	48	34	7	25	33
POT1	62	113	72	130	73	105
	142	179	102	47	143	22
POTEA	30	0	10	0	5	0
	0	1	27	0	14	0
POTEC	40	0	14	0	10	2
	0	0	12	0	11	0
POTED	13	0	12	0	7	0
	0	0	13	0	12	0
POTEE	27	0	20	0	2	0
	1	0	5	0	7	0
POTEF	67	7	41	3	25	2
	2	7	54	1	181	1
POTEG	8	0	15	0	6	0
	0	0	14	0	6	0
POTEH	16	0	23	0	9	0
	0	0	21	0	9	0
POTEI	15	0	12	0	3	0
	1	0	12	0	12	0
POTEJ	29	0	9	0	7	0
	1	0	11	0	11	0
POTEM	431	68	267	32	102	14
	50	64	236	14	184	15
POU1F1	7	0	5	0	3	0
	0	0	9	0	1	0
POU2AF1	12	1	6	1	0	2
	1	0	5	1	3	0
POU2F1	145	490	177	434	251	382
	325	551	306	137	358	97
POU2F2	26	13	22	8	9	10
	2	8	12	3	6	2
POU2F3	27	1	16	2	7	0
	0	1	7	0	4	1
POU3F1	3	1	1	1	5	0
	4	1	2	0	0	0
POU3F2	15	0	13	1	2	1
	0	0	7	0	6	1
POU3F3	0	1	0	0	0	0
	0	0	1	0	0	0
POU3F4	1	0	2	0	0	0
	0	0	2	0	1	0
POU4F1	19	3	16	3	11	4
	2	3	7	3	12	0
POU4F2	3	1	8	0	1	0
	0	1	7	0	4	0
POU4F3	3	0	1	0	0	0
	0	0	4	0	0	0
POU5F1	10	10	9	2	4	1
	1	6	5	1	3	0
POU5F1B	5	3	1	1	2	0
	1	0	4	0	2	0
POU6F1	31	91	25	79	49	58
	82	77	66	35	64	17

POU6F2	19	0	27	0	8	0
	0	1	6	0	5	0
PP2D1	10	1	4	1	1	0
	0	0	7	0	6	0
PPA1	112	445	58	282	208	331
	298	518	218	139	197	76
PPA2	100	463	60	403	159	297
	499	657	264	117	268	100
PPAN	4	22	5	3	6	9
	11	16	8	4	18	2
PPAN-P2RY11		10	35	8	23	11
	26	43	35	20	9	34
	5					
PPAP2A	73	414	74	355	189	359
	246	423	153	89	203	42
PPAP2B	65	318	119	353	142	263
	246	587	231	90	185	60
PPAP2C	2	1	1	0	0	0
	3	0	3	0	1	0
PPAPDC1A	7	0	4	0	1	0
	0	0	3	0	2	0
PPAPDC1B	30	38	22	35	20	30
	18	41	26	12	23	4
PPAPDC2	17	28	8	28	13	18
	11	36	27	5	24	5
PPAPDC3	115	471	71	398	232	394
	425	663	272	169	320	100
PPARA	241	1084	201	631	562	513
	943	1204	478	333	586	251
PPARD	25	118	18	66	39	67
	79	115	33	31	45	30
PPARG	16	49	16	27	41	15
	40	67	40	12	23	28
PPARGC1A	297	1441	237	945	712	896
	2160	2422	736	630	1063	294
PPARGC1B	165	579	140	404	219	470
	219	328	164	130	145	55
PPAT	41	70	25	59	63	47
	56	82	59	14	49	7
PPBP	12	32	2	8	4	24
	11	8	6	1	2	0
PPCDC	18	29	17	29	16	25
	27	53	25	6	35	11
PPCS	30	244	37	124	79	145
	202	255	115	47	109	37
PPDPF	9	86	12	27	28	44
	71	68	24	25	34	39
PPEF1	29	0	9	2	5	0
	0	0	10	0	3	0
PPEF2	22	3	11	0	3	3
	0	4	9	0	12	0
PPFIA1	147	717	183	516	279	567
	593	665	302	224	435	149
PPFIA2	74	39	61	39	20	23
	13	22	42	8	28	3
PPFIA3	26	1	19	5	8	9
	7	2	17	1	5	0

PPFIA4	89	223	56	106	141	147
	139	212	82	62	87	25
PPFIBP1	158	778	142	572	398	427
	620	686	304	265	409	149
PPFIBP2	100	345	138	197	190	278
	322	490	182	133	245	87
PPHLN1	95	323	92	255	157	247
	197	356	149	101	201	50
PPIA	91	590	160	499	258	382
	601	790	276	204	294	133
PPIAL4A	1	0	0	0	0	0
	0	0	0	0	0	0
PPIAL4B	1	1	1	0	0	1
	0	0	1	0	0	0
PPIAL4C	2	0	1	1	0	0
	0	0	1	0	0	0
PPIAL4D	3	0	2	0	0	0
	0	0	0	0	0	0
PPIAL4E	2	1	2	0	0	0
	0	0	0	0	0	0
PPIAL4F	1	1	0	0	0	0
	0	0	3	0	0	0
PPIAL4G	7	0	0	0	2	0
	0	0	0	0	2	0
PPIB	54	207	37	183	90	156
	171	305	100	65	104	36
PPIC	17	173	33	146	89	123
	181	215	89	44	81	25
PPID	108	585	74	352	308	445
	451	754	224	180	327	94
PPIE	76	229	64	171	101	142
	214	304	157	56	163	30
PPIF	45	215	23	116	67	74
	198	168	64	37	123	20
PPIG	101	495	134	339	288	393
	419	499	159	188	262	103
PPIH	20	59	17	31	20	30
	46	70	33	15	35	14
PPIL1	26	138	19	54	40	65
	56	98	48	17	66	10
PPIL2	67	232	50	119	111	172
	176	262	84	64	132	49
PPIL3	31	124	32	81	45	78
	52	162	67	40	57	24
PPIL4	102	431	112	243	228	324
	318	535	175	126	254	94
PPIL6	27	29	10	14	13	20
	12	20	15	8	20	9
PPIP5K1	179	399	158	227	141	211
	228	295	162	97	222	81
PPIP5K2	96	331	110	278	155	228
	213	357	184	99	205	60
PPL	52	218	84	232	82	214
	67	302	105	39	67	48
PPM1A	711	3481	657	2784	1978	2333
	2328	3552	1454	1081	1744	527
PPM1B	289	1739	206	906	736	1006
	1387	2866	608	546	1138	348

PPM1D	33	107	38	71	50	79
	105	91	51	36	72	20
PPM1E	25	1	22	2	12	3
	0	0	17	0	9	0
PPM1F	50	163	47	95	75	107
	73	146	68	34	68	17
PPM1G	56	407	59	277	193	428
	371	452	190	139	245	48
PPM1H	28	94	24	42	30	27
	58	48	39	14	40	13
PPM1J	46	203	27	183	76	159
	242	163	88	59	132	21
PPM1K	76	724	152	253	219	151
	235	732	326	120	383	114
PPM1L	67	312	79	290	208	199
	190	174	104	77	134	30
PPM1M	24	38	10	20	14	23
	9	36	18	6	18	5
PPM1N	9	0	2	2	2	0
	1	1	3	0	2	0
PPME1	56	193	58	129	98	110
	161	208	101	53	120	28
PPOX	15	60	10	38	11	25
	44	41	24	10	16	5
PPP1CA	59	222	50	134	93	204
	232	256	122	50	135	21
PPP1CB	1597	9387	1392	8450	4446	7153
	10114	12019	4524	2403	6320	1385
PPP1CC	223	1513	257	1194	603	1148
	1276	1308	704	403	727	247
PPP1R10	73	232	90	156	143	212
	209	189	94	80	132	42
PPP1R11	35	131	35	87	52	93
	149	151	66	45	76	19
PPP1R12A	228	1252	316	860	598	802
	821	1214	472	387	630	248
PPP1R12B	1403	6886	1516	4932	3567	4368
	4637	5945	2325	2197	3788	1399
PPP1R12C	16	70	14	52	32	43
	55	46	16	21	42	21
PPP1R13B	48	113	37	74	50	69
	65	46	48	32	44	10
PPP1R13L	13	15	6	13	3	2
	8	1	5	2	6	1
PPP1R14A	4	8	2	5	0	1
	4	1	2	0	0	1
PPP1R14B	90	570	102	296	185	299
	729	737	200	101	266	55
PPP1R14C	78	596	72	267	192	255
	291	313	105	98	105	41
PPP1R14D	8	0	3	0	1	0
	0	0	5	0	0	0
PPP1R15A	90	350	73	219	133	368
	254	465	150	147	205	64
PPP1R15B	82	382	77	304	194	328
	411	521	190	119	324	68
PPP1R16A	16	76	7	36	40	16
	49	92	36	8	38	12

PPP1R16B	32	98	18	47	50	61
	56	76	48	32	57	27
PPP1R17	8	1	3	0	0	0
	0	0	4	0	1	0
PPP1R18	31	112	43	61	38	69
	58	70	33	28	32	17
PPP1R1A	487	3026	396	1796	1111	2088
	2400	3782	903	702	1641	388
PPP1R1B	13	3	13	0	4	2
	0	0	4	0	5	2
PPP1R1C	44	148	40	32	49	72
	66	164	36	28	69	18
PPP1R2	331	1232	200	1231	829	1203
	1116	1764	751	486	796	231
PPP1R21	33	143	38	101	58	73
	94	113	84	35	83	31
PPP1R26	18	81	16	18	39	24
	47	57	21	9	16	14
PPP1R27	52	228	28	129	118	177
	210	585	199	59	189	46
PPP1R32	5	10	9	3	3	4
	7	12	7	1	9	2
PPP1R35	10	7	2	2	3	4
	6	21	9	2	3	1
PPP1R36	18	7	11	14	11	7
	6	5	15	4	6	1
PPP1R37	16	69	16	36	44	36
	37	68	28	20	36	16
PPP1R3A	1844	12145	2672	10256	6512	10391
	9309	9983	5381	3842	6828	1795
PPP1R3B	714	2897	788	2881	1493	3771
	2658	1325	1247	599	1446	474
PPP1R3C	1464	9325	1609	9202	3886	10963
	7343	3763	4163	2627	3658	875
PPP1R3D	25	147	35	187	59	94
	95	125	68	22	77	12
PPP1R3F	25	110	15	92	56	80
	75	137	56	29	67	22
PPP1R3G	3	1	3	1	2	4
	0	2	1	1	1	0
PPP1R42	0	0	5	0	1	1
	0	2	6	0	1	1
PPP1R7	65	241	49	150	146	233
	194	233	108	97	141	42
PPP1R8	44	143	44	126	100	125
	143	220	95	45	123	37
PPP1R9A	218	918	193	582	430	644
	504	819	386	291	467	161
PPP1R9B	17	87	21	74	49	86
	53	77	38	25	45	12
PPP2CA	194	1027	137	657	463	560
	737	1169	388	247	590	188
PPP2CB	77	387	69	396	262	311
	366	500	209	96	256	82
PPP2R1A	107	479	98	401	235	321
	391	486	316	138	336	93
PPP2R1B	67	191	60	114	98	130
	203	274	108	98	116	68

PPP2R2A	148	877	187	720	392	654
	641	716	246	211	334	113
PPP2R2B	39	5	26	4	8	1
	0	1	6	0	20	1
PPP2R2C	17	9	5	4	1	1
	2	2	7	0	6	2
PPP2R2D	47	268	55	155	88	126
	250	259	97	60	137	70
PPP2R3A	1042	5116	1107	3994	2796	4410
	4285	6046	2585	1576	3905	930
PPP2R3B (NC_000023 294667..347690)				46	183	44
	101	95	122	141	262	57
	41	113	39			
PPP2R3B (NC_000024 244667..297690)				57	185	34
	126	82	136	155	207	98
	67	128	37			
PPP2R3C	53	212	26	162	107	174
	162	245	134	66	137	32
PPP2R4	98	429	83	288	143	294
	317	395	198	95	200	64
PPP2R5A	109	535	88	451	305	398
	438	663	270	181	304	112
PPP2R5B	39	108	35	76	40	81
	64	126	65	33	51	16
PPP2R5C	213	748	179	559	424	622
	617	716	369	252	496	145
PPP2R5D	79	295	61	167	144	256
	186	291	121	92	157	58
PPP2R5E	75	493	84	373	238	283
	279	373	133	77	207	58
PPP3CA	253	1755	365	1368	757	1078
	1047	1236	511	468	595	205
PPP3CB	532	4354	479	2938	1850	2807
	2824	3648	1442	827	1773	407
PPP3CC	167	713	147	567	365	468
	781	1173	422	237	575	199
PPP3R1	126	1338	188	1201	500	695
	1012	658	400	159	451	104
PPP3R2	0	0	0	0	0	0
	0	0	1	0	0	0
PPP4C	20	47	11	26	28	54
	37	52	26	10	30	10
PPP4R1	97	378	111	328	168	296
	278	424	145	101	235	83
PPP4R2	67	382	75	278	186	244
	250	289	121	98	208	59
PPP4R4	31	2	26	3	15	1
	1	3	32	0	21	1
PPP5C	57	282	41	184	124	156
	229	286	100	98	148	50
PPP5D1	9	1	5	5	2	3
	0	5	6	1	2	1
PPP6C	239	1308	171	1068	623	1161
	1220	1584	627	415	837	218
PPP6R1	39	196	21	90	88	109
	117	118	54	40	60	30
PPP6R2	95	497	114	402	160	300
	323	462	240	138	264	79

PPP6R3	430	1774	448	1230	1004	1439
	1379	2151	926	597	1233	343
PPRC1	44	93	28	52	48	77
	98	96	57	42	66	26
PPT1	48	163	30	119	62	130
	114	197	93	37	95	32
PPT2	46	99	26	67	41	48
	76	98	35	15	48	12
PPTC7	181	1178	156	682	484	610
	1007	766	279	280	373	131
PPWD1	80	372	88	251	205	304
	294	552	191	130	310	73
PPY	1	0	2	0	0	0
	0	0	0	0	1	0
PPYR1	14	0	6	1	0	0
	0	0	11	0	3	0
PQBP1	34	158	22	92	58	90
	163	145	60	56	77	15
PQLC1	25	78	21	61	25	75
	77	99	56	30	46	22
PQLC2	15	19	7	8	7	5
	10	17	14	12	10	4
PQLC3	14	39	17	68	40	47
	41	80	38	18	41	6
PRAC	3	0	1	0	0	0
	0	0	0	0	0	0
PRADC1	38	188	23	145	50	127
	217	231	93	43	107	23
PRAF2	9	7	0	8	1	5
	9	9	3	5	2	2
PRAM1	7	0	2	0	1	0
	1	0	1	0	2	0
PRAME	14	0	8	0	0	0
	0	1	5	0	5	0
PRAMEF1	12	0	8	0	2	0
	0	0	11	0	3	0
PRAMEF10	25	0	7	0	3	0
	0	0	0	0	5	0
PRAMEF11	21	0	11	0	6	0
	0	0	8	0	5	0
PRAMEF12	11	0	5	0	2	0
	0	0	1	0	2	0
PRAMEF13	27	0	15	0	4	0
	0	0	13	0	8	0
PRAMEF14	3	0	1	0	1	0
	0	0	0	0	2	0
PRAMEF15	4	0	1	0	2	1
	0	0	4	0	2	0
PRAMEF16	5	0	0	0	0	0
	0	0	0	0	0	0
PRAMEF17	2	0	2	0	2	1
	0	0	2	0	0	0
PRAMEF18	3	0	2	1	0	0
	0	0	0	0	2	0
PRAMEF19	0	0	1	0	1	0
	0	0	2	0	5	0
PRAMEF2	27	0	8	0	2	0
	0	0	5	0	2	0

PRAMEF20	11	0	0	0	0	0
	0	0	3	0	2	0
PRAMEF21	0	0	1	0	0	0
	0	0	0	0	0	0
PRAMEF22	2	0	0	0	1	1
	0	0	1	0	0	1
PRAMEF23	35	0	13	0	2	0
	0	0	5	0	12	0
PRAMEF25	8	0	2	0	0	0
	0	0	1	0	2	0
PRAMEF3	5	0	2	0	3	0
	0	0	3	0	2	0
PRAMEF4	8	0	3	0	3	0
	0	0	4	0	5	0
PRAMEF5	6	0	8	0	4	0
	0	0	5	0	2	0
PRAMEF6	2	0	4	0	0	0
	0	0	2	0	1	0
PRAMEF7	3	0	0	0	1	0
	0	0	0	0	1	0
PRAMEF8	17	0	7	0	6	0
	0	0	4	0	5	0
PRAMEF9	2	0	3	0	0	0
	0	0	6	0	0	0
PRAP1	3	0	3	0	0	1
	0	1	2	0	0	0
PRB1	10	0	8	0	0	0
	0	0	4	0	1	0
PRB2	13	0	8	0	3	1
	0	0	2	0	2	0
PRB3	8	0	5	0	3	0
	0	0	4	0	3	0
PRB4	2	1	2	0	0	0
	0	0	3	0	2	0
PRC1	39	23	20	17	19	23
	21	24	24	2	16	5
PRCC	38	137	20	91	73	86
	136	141	55	39	95	27
PRCD	6	15	6	1	3	2
	5	10	5	3	4	2
PRCP	32	147	28	132	69	86
	85	172	72	32	54	23
PRDM1	33	26	23	18	14	14
	22	22	19	9	27	2
PRDM10	114	197	111	220	146	277
	250	444	180	132	249	49
PRDM11	57	27	22	30	20	21
	45	33	24	7	20	3
PRDM12	6	0	5	0	1	0
	0	0	2	0	2	0
PRDM13	8	0	5	0	3	0
	0	0	5	0	2	0
PRDM14	18	0	9	1	3	0
	0	0	7	0	3	2
PRDM15	66	108	53	79	42	93
	84	128	72	35	54	16
PRDM16	24	14	17	6	6	8
	5	9	16	1	20	3

PRDM2	183	862	172	617	339	515
	667	695	238	221	411	162
PRDM4	77	235	78	144	108	149
	153	214	107	39	120	40
PRDM5	26	76	23	61	40	52
	55	71	65	35	59	17
PRDM6	17	3	6	2	7	2
	7	4	10	3	3	3
PRDM7	54	0	28	0	12	1
	1	1	20	2	12	0
PRDM8	18	14	23	11	6	12
	23	25	19	6	28	2
PRDM9	37	0	19	0	6	0
	0	0	12	0	3	0
PRDX1	289	1534	321	1234	941	1211
	1315	2424	799	440	945	289
PRDX2	168	775	128	692	363	473
	1146	942	488	269	559	141
PRDX3	390	1983	269	1492	698	2005
	2308	2862	1070	496	1193	300
PRDX4	29	134	20	107	51	110
	113	185	74	30	79	22
PRDX5	98	570	78	396	206	345
	810	731	309	137	298	73
PRDX6	470	2356	358	1306	1105	1206
	2395	3316	1086	770	1242	512
PREB	140	590	74	326	223	490
	460	729	183	169	248	81
PRELID1	21	48	13	45	21	26
	38	89	15	17	33	12
PRELID2	22	29	19	16	15	15
	24	22	21	9	14	2
PRELP	49	81	28	111	52	69
	47	69	29	16	26	49
PREP	116	1072	138	642	454	487
	693	805	275	201	357	101
PREPL	204	1043	440	828	643	683
	704	1181	474	374	556	212
PREX1	55	102	59	69	49	72
	94	191	67	120	100	45
PREX2	128	403	83	262	208	169
	307	433	237	140	224	98
PRF1	9	6	3	2	2	2
	8	6	13	4	10	0
PRG2	6	2	3	1	2	0
	0	1	5	0	9	0
PRG3	3	0	3	0	0	0
	0	0	9	0	1	0
PRG4	64	48	24	178	43	107
	25	78	53	5	44	77
PRH1	0	4	1	2	0	9
	1	1	4	0	2	0
PRH2	18	5	15	3	6	7
	2	5	11	0	7	2
PRHOXNB	2	0	0	0	0	0
	0	0	0	0	0	0
PRICKLE1	40	75	27	54	30	34
	48	33	39	12	28	11

PRICKLE2	73	164	66	120	70	88
	112	134	105	35	91	22
PRICKLE3	12	18	2	10	9	11
	32	27	13	6	9	8
PRICKLE4	20	19	7	11	8	6
	18	19	4	6	3	3
PRIM1	14	67	29	41	32	32
	44	57	28	13	47	8
PRIMA1	14	1	6	6	4	1
	2	0	8	0	4	1
PRKAA1	153	593	181	497	255	502
	549	784	395	200	433	138
PRKAA2	1122	5045	1353	4802	3264	4596
	4930	9262	3914	1848	5272	1259
PRKAB1	21	49	17	58	38	33
	39	119	46	23	52	12
PRKAB2	304	1740	323	1233	694	1054
	1453	1543	773	429	852	291
PRKACA	80	615	99	359	137	249
	528	307	105	114	174	67
PRKACB	76	219	78	156	128	117
	159	212	117	50	111	32
PRKACG	1	0	2	0	1	1
	0	0	2	0	0	0
PRKAG1	158	716	104	464	262	635
	641	935	415	179	488	141
PRKAG2	18	70	24	55	28	26
	52	74	47	10	43	8
PRKAG3	89	421	114	481	308	632
	213	199	168	62	146	42
PRKAR1A	650	3307	981	3534	1863	2611
	2596	3598	1514	865	1850	542
PRKAR1B	14	22	12	12	4	10
	12	15	14	2	18	5
PRKAR2A	146	737	164	553	429	506
	484	530	244	205	393	129
PRKAR2B	17	62	13	33	48	30
	34	73	23	16	38	41
PRKCA	107	234	33	118	131	159
	204	195	77	76	72	34
PRKCB	57	29	36	15	16	17
	9	18	29	7	17	8
PRKCD	14	14	11	6	10	8
	18	19	10	2	8	7
PRKCDBP	10	23	6	26	9	34
	19	14	12	2	6	4
PRKCE	63	167	48	112	65	106
	107	121	82	36	50	20
PRKCG	21	0	10	0	1	0
	0	0	12	0	2	0
PRKCH	96	222	46	134	85	115
	203	288	122	104	135	38
PRKCI	75	278	94	269	155	234
	286	282	174	109	187	47
PRKCQ	377	1933	364	1253	828	1342
	2191	2722	1320	651	1878	403
PRKCSH	44	180	53	108	72	123
	149	175	99	67	99	38

PRKCZ	15	6	12	0	1	0
	1	3	5	1	8	1
PRKD1	43	84	38	35	49	34
	53	38	44	11	54	12
PRKD2	22	51	20	33	23	27
	49	31	33	13	21	5
PRKD3	152	556	94	435	332	392
	470	507	293	219	339	144
PRKDC	552	2351	646	1662	1166	1681
	1694	2764	1201	836	1526	567
PRKG1	473	1564	355	1206	1152	1325
	1543	2362	680	657	1184	386
PRKG2	50	2	29	5	13	1
	1	4	22	0	23	0
PRKRA	108	515	112	423	250	409
	518	715	323	185	390	122
PRKRIP1	6	87	26	48	25	38
	60	119	48	14	48	11
PRKRIR	140	734	187	721	343	609
	659	956	329	221	470	152
PRKX	46	78	20	40	30	53
	54	81	54	22	48	8
PRL	9	0	7	0	2	0
	0	0	4	0	3	0
PRLH	1	0	0	0	0	0
	0	0	0	0	0	0
PRLHR	2	1	0	0	0	0
	0	0	3	0	0	0
PRLR	64	12	38	7	13	4
	3	7	31	2	14	0
PRM1	0	0	1	0	0	0
	0	0	2	0	0	0
PRM2	5	0	2	0	0	1
	0	0	1	0	1	0
PRM3	0	0	1	0	0	0
	0	0	0	0	0	0
PRMT1	127	611	166	498	341	504
	599	919	389	214	386	106
PRMT10	42	118	41	105	54	96
	97	141	101	38	92	22
PRMT2	89	313	132	211	159	288
	269	278	156	86	156	59
PRMT3	43	160	59	101	94	106
	110	178	94	50	105	35
PRMT5	54	220	62	152	110	163
	167	246	106	49	127	44
PRMT6	4	14	2	11	4	8
	18	11	9	3	11	1
PRMT7	66	180	48	100	90	156
	160	230	124	63	145	34
PRMT8	13	0	9	0	4	0
	0	0	10	0	5	0
PRND	8	2	8	0	4	0
	2	0	3	0	1	0
PRNP	90	393	132	347	180	262
	373	459	223	88	224	112
PROB1	45	186	35	119	79	143
	145	147	84	62	101	28

PROC	5	0	5	0	3	2
	0	0	4	0	2	0
PROCA1	8	2	6	2	1	1
	0	5	1	0	1	0
PROCR	14	34	5	21	32	18
	17	70	20	11	16	10
PRODH	12	24	13	3	8	10
	30	128	27	17	32	19
PRODH2	6	0	4	0	0	0
	0	0	4	0	3	0
PROK1	6	0	4	0	1	0
	0	0	6	0	2	0
PROK2	8	1	2	2	5	1
	0	0	3	0	3	0
PROKR1	5	0	2	0	0	0
	0	0	0	0	0	0
PROKR2	10	0	4	0	2	0
	0	0	4	0	0	0
PROL1	13	0	8	0	7	0
	0	1	6	0	2	0
PROM1	42	16	22	11	16	30
	2	32	32	13	31	5
PROM2	26	5	10	7	7	11
	6	9	18	3	5	1
PROP1	2	0	3	0	4	0
	0	0	2	0	3	0
PROS1	52	142	35	181	83	82
	110	211	78	45	103	34
PROSAPIP1	18	30	12	31	22	8
	31	58	24	14	20	10
PROSC	51	377	65	208	158	266
	238	260	127	90	120	38
PROSER1	74	223	67	182	104	116
	160	251	107	53	114	44
PROX1	172	568	167	248	221	224
	389	665	188	104	172	79
PROX2	16	3	8	4	0	0
	1	2	13	0	5	0
PROZ	8	1	5	0	4	0
	3	1	6	0	6	0
PRPF18	70	366	105	194	163	241
	299	429	199	133	221	64
PRPF19	50	188	41	132	93	170
	131	179	99	45	97	43
PRPF3	102	528	138	274	193	288
	305	533	188	118	268	86
PRPF31	52	228	49	145	118	179
	192	263	111	74	121	44
PRPF38A	65	212	57	164	109	126
	151	204	81	74	117	31
PRPF38B	64	246	70	152	140	185
	235	258	131	113	142	50
PRPF39	111	502	176	422	234	462
	442	612	281	165	323	108
PRPF4	66	162	53	106	76	121
	142	180	75	51	108	35
PRPF40A	199	997	258	709	510	809
	643	1060	428	336	556	210

PRPF40B	52	182	41	93	72	98
	108	216	63	54	88	28
PRPF4B	244	1307	289	901	586	1027
	1019	1548	526	456	869	271
PRPF6	119	711	164	390	311	469
	452	635	216	200	349	134
PRPF8	504	2955	542	1936	1177	1614
	2192	2147	829	820	1169	447
PRPH	5	3	2	2	0	0
	2	2	4	0	3	0
PRPH2	26	103	16	78	50	119
	102	35	40	12	45	13
PRPS1	64	199	32	199	93	172
	166	240	97	56	120	43
PRPS1L1	7	0	4	0	1	0
	0	1	0	0	1	0
PRPS2	30	17	15	16	7	7
	13	14	13	10	10	3
PRPSAP1	29	119	43	90	36	68
	87	93	47	20	41	9
PRPSAP2	31	77	39	55	36	57
	45	94	61	20	64	11
PRR11	36	11	26	8	7	6
	9	6	33	5	16	0
PRR12	31	112	35	56	63	54
	64	71	27	30	56	28
PRR13	15	86	26	59	42	59
	82	110	44	23	49	17
PRR14	18	30	7	24	12	33
	47	40	16	21	17	7
PRR14L	162	517	126	320	262	362
	353	462	228	183	266	88
PRR15	4	0	3	1	1	0
	0	2	6	0	1	0
PRR15L	10	0	7	0	1	2
	0	0	2	0	2	0
PRR16	36	182	55	95	68	99
	181	102	66	35	107	13
PRR18	7	0	2	0	0	0
	0	0	2	0	0	1
PRR19	4	0	0	0	0	0
	0	0	2	0	1	0
PRR20A	16	0	6	1	1	0
	0	0	8	0	2	0
PRR20B	30	0	9	0	14	0
	0	0	11	0	5	0
PRR20C	14	0	7	0	4	0
	0	0	4	0	1	0
PRR20D	11	0	4	0	2	0
	0	0	3	0	7	0
PRR20E	11	0	5	0	1	0
	0	0	4	0	2	0
PRR21	0	0	4	0	0	0
	0	0	0	0	0	0
PRR22	6	5	1	0	1	0
	3	5	3	0	3	0
PRR23A	12	0	2	0	3	0
	0	0	1	0	2	0

PRR23B	8	0	2	0	1	0
	0	0	0	0	0	0
PRR23C	10	0	5	0	1	0
	0	0	9	0	3	0
PRR24	0	6	2	5	1	1
	5	7	3	1	2	0
PRR25	0	0	0	0	0	0
	0	0	1	0	0	0
PRR3	26	14	12	14	19	16
	11	17	18	7	16	5
PRR4	0	3	0	5	5	5
	1	3	3	2	7	1
PRR5	5	1	5	5	0	1
	0	2	5	0	1	1
PRR5-ARHGAP8		14	0	5	3	1
	0	0	0	6	0	2
	0					
PRR5L	27	13	16	2	9	7
	8	3	22	6	6	4
PRR7	2	0	1	1	0	0
	5	6	1	0	1	0
PRR9	2	0	2	0	1	0
	0	0	2	0	0	0
PRRC1	97	318	132	346	179	273
	254	394	206	98	202	67
PRRC2A	119	618	123	292	229	328
	330	439	192	137	247	96
PRRC2B	258	1181	207	566	444	676
	728	863	341	304	412	222
PRRC2C	364	2088	487	1499	910	1270
	1359	1635	605	634	796	348
PRRG1	256	905	230	684	516	673
	622	692	321	215	418	100
PRRG2	4	2	4	1	3	1
	1	3	5	0	1	0
PRRG3	4	6	2	3	3	2
	2	0	3	3	3	0
PRRG4	16	2	12	9	10	7
	1	9	13	3	7	4
PRRT1	5	1	1	0	0	0
	0	0	2	1	1	0
PRRT2	13	23	14	14	9	12
	12	26	7	4	13	8
PRRT3	7	3	4	2	2	4
	2	3	4	0	2	0
PRRT4	4	8	9	6	6	12
	9	2	9	1	3	4
PRRX1	326	1130	354	741	684	607
	771	2041	379	410	751	288
PRRX2	3	0	0	3	1	0
	0	2	3	0	0	0
PRSS1	2	0	2	0	0	0
	0	0	0	0	0	0
PRSS12	23	4	23	16	14	7
	3	4	21	1	22	1
PRSS16	24	0	11	0	2	1
	0	0	11	0	8	0

PRSS21	4	0	4	0	1	0
	0	0	1	0	3	0
PRSS22	5	0	2	0	0	0
	0	0	3	0	2	0
PRSS23	138	600	108	579	144	372
	597	347	486	132	478	120
PRSS27	3	3	2	1	0	0
	1	1	1	1	1	0
PRSS3	18	0	2	0	1	0
	0	0	4	0	3	0
PRSS33	2	0	3	0	0	0
	0	0	2	0	2	0
PRSS35	13	5	9	1	4	4
	3	2	9	1	2	0
PRSS36	5	0	10	0	5	6
	4	2	6	2	6	0
PRSS37	19	0	12	0	1	2
	0	2	7	0	3	0
PRSS38	1	0	0	0	1	0
	0	0	0	0	1	0
PRSS41	4	0	2	0	0	0
	0	0	0	0	0	0
PRSS42	116	345	79	49	153	115
	142	863	69	201	189	116
PRSS44	23	31	16	9	30	24
	23	123	25	18	23	15
PRSS45	17	21	10	4	16	7
	10	62	9	10	16	5
PRSS46	7	4	5	3	4	5
	2	21	4	3	2	0
PRSS48	10	1	7	0	2	0
	0	2	9	0	4	1
PRSS50	13	7	14	3	12	2
	8	53	3	9	4	8
PRSS53	7	4	6	3	4	4
	7	11	4	5	4	0
PRSS54	12	1	7	0	1	2
	1	1	4	0	2	0
PRSS55	1	0	3	0	1	0
	0	0	6	0	3	0
PRSS56	10	14	8	19	15	18
	16	11	13	4	20	5
PRSS57	2	0	1	0	0	0
	0	0	1	0	0	0
PRSS58	9	0	3	0	0	0
	0	0	2	0	3	0
PRSS8	6	0	6	0	2	0
	0	0	6	0	4	0
PRTFDC1	18	18	14	16	8	19
	20	33	20	2	18	0
PRTG	106	62	82	78	48	50
	69	58	107	23	86	16
PRTN3	1	0	1	0	0	0
	0	0	1	0	0	0
PRUNE	69	250	50	163	125	191
	206	255	149	56	152	29
PRUNE2	93	324	256	128	33	91
	61	66	106	60	118	50

PRX	22	20	20	30	16	27
	14	22	16	5	14	9
PRY	0	0	3	1	0	0
	0	0	1	0	0	0
PRY2	0	0	10	2	0	0
	0	0	4	0	1	0
PSAP	899	4021	929	3480	1953	3890
	3539	4290	1970	1213	2531	776
PSAPL1	18	0	3	0	1	0
	0	0	5	0	5	0
PSAT1	16	6	6	3	4	3
	2	1	19	0	11	0
PSCA	5	4	1	2	0	2
	0	1	3	0	0	0
PSD	21	1	4	1	3	3
	0	1	6	0	7	0
PSD2	22	7	26	1	12	8
	0	4	14	1	13	1
PSD3	110	118	83	122	84	108
	64	122	120	40	92	42
PSD4	34	15	16	5	7	3
	7	12	24	9	10	4
PSEN1	93	306	80	309	214	292
	355	376	226	120	215	87
PSEN2	55	292	45	209	96	198
	189	227	126	71	148	38
PSENEN	18	32	9	23	15	16
	30	39	27	6	16	4
PSG1	21	0	16	0	3	0
	0	0	10	0	6	0
PSG11	10	0	4	0	1	0
	0	0	5	0	2	0
PSG2	12	0	5	0	6	0
	0	0	3	0	0	0
PSG3	14	0	6	0	3	1
	0	0	3	0	2	0
PSG4	16	0	15	0	4	0
	0	1	6	0	6	0
PSG5	8	0	6	0	1	0
	0	0	8	0	1	0
PSG6	10	0	3	0	1	0
	0	0	1	0	4	0
PSG7	15	0	4	1	1	0
	0	0	5	0	2	0
PSG8	19	0	8	0	6	1
	0	0	9	1	3	0
PSG9	15	0	4	0	0	0
	0	0	3	0	1	0
PSIP1	110	387	103	256	222	345
	336	509	181	166	254	77
PSKH1	18	75	16	41	27	33
	53	55	26	17	23	11
PSKH2	3	1	10	0	0	0
	0	0	1	0	4	0
PSMA1	128	548	75	487	234	385
	491	794	317	166	319	102
PSMA2	133	537	85	530	264	408
	656	1048	361	155	374	81

PSMA3	144	817	139	678	398	607
	783	919	387	218	451	126
PSMA4	160	892	139	711	422	730
	862	1302	530	304	547	171
PSMA5	112	441	98	360	257	391
	450	786	353	144	311	101
PSMA6	301	1272	259	1388	543	1106
	1882	2033	895	434	870	270
PSMA7	186	847	135	813	323	723
	1039	1320	583	273	545	137
PSMA8	13	1	5	1	6	2
	8	17	10	4	5	0
PSMB1	173	705	163	638	345	594
	773	1416	499	199	566	148
PSMB10	11	56	7	34	31	43
	70	87	28	18	31	5
PSMB11	7	1	0	0	2	0
	0	0	1	0	1	0
PSMB2	73	221	42	188	125	189
	202	295	128	78	178	46
PSMB3	44	196	34	166	89	101
	182	313	126	60	135	31
PSMB4	85	340	69	336	135	284
	372	561	252	101	243	68
PSMB5	152	676	103	749	304	683
	894	1027	568	216	503	164
PSMB6	100	469	64	478	223	356
	474	625	290	147	264	77
PSMB7	122	457	103	370	244	369
	582	713	321	133	351	81
PSMB8	12	47	8	42	20	30
	57	76	42	20	45	11
PSMB9	7	31	6	15	7	14
	32	29	22	3	16	5
PSMC1	288	1505	262	1188	676	1234
	1303	1590	704	460	945	231
PSMC2	359	1528	276	1163	762	1344
	1346	2123	766	564	1071	358
PSMC3	181	933	159	616	420	698
	633	939	416	276	512	117
PSMC3IP	12	13	6	14	11	6
	14	19	7	9	8	0
PSMC4	80	427	67	374	162	337
	384	496	192	120	231	69
PSMC5	52	347	44	236	92	182
	341	212	90	66	109	29
PSMC6	222	1169	199	928	506	761
	976	1285	581	328	778	191
PSMD1	336	1977	348	1598	877	1356
	1212	1739	813	576	1045	333
PSMD10	40	168	31	155	95	130
	146	235	101	54	110	21
PSMD11	171	772	149	510	358	594
	651	799	374	206	460	164
PSMD12	83	705	95	474	257	379
	447	361	210	134	294	76
PSMD13	76	412	76	322	162	229
	365	503	258	91	230	77

PSMD14	112	586	127	492	231	425
	419	685	320	153	337	105
PSMD2	325	1611	284	1545	690	1272
	1153	1321	666	419	812	205
PSMD3	159	519	98	486	241	430
	425	620	296	169	340	97
PSMD4	240	1238	218	1051	548	1016
	1081	1558	627	323	730	190
PSMD5	36	127	28	110	53	99
	123	202	78	34	122	32
PSMD6	190	1241	224	793	507	1011
	1077	1324	591	333	685	164
PSMD7	248	1303	176	954	599	1005
	1254	1551	678	429	874	219
PSMD8	117	590	72	383	229	493
	598	687	338	146	399	91
PSMD9	27	109	19	95	34	78
	100	139	55	30	94	15
PSME1	30	210	35	101	77	116
	186	234	94	63	111	39
PSME2	28	114	37	75	48	68
	113	162	54	35	86	24
PSME3	58	219	34	132	94	124
	182	210	94	46	114	30
PSME4	456	2156	449	1873	998	1420
	1790	2315	888	605	1514	512
PSMF1	120	663	91	349	230	379
	511	549	230	117	281	99
PSMG1	20	97	33	113	53	76
	147	162	63	29	55	19
PSMG2	31	87	12	72	48	64
	83	214	60	26	64	25
PSMG3	9	23	7	31	17	27
	27	40	16	10	15	7
PSMG4	8	92	21	42	24	65
	120	136	44	23	50	9
PSORS1C1	3	0	7	0	0	0
	0	0	6	0	2	0
PSORS1C2	4	0	0	0	0	0
	0	0	2	0	0	0
PSPC1	79	371	118	294	174	326
	312	466	216	174	310	98
PSPH	22	57	21	34	9	32
	25	31	9	7	20	3
PSRC1	16	1	6	7	7	7
	5	5	4	1	2	0
PSTK	13	17	13	21	13	29
	19	25	18	6	15	4
PSTPIP1	11	7	5	10	6	4
	4	8	7	1	10	1
PSTPIP2	111	502	67	671	222	690
	530	241	162	133	233	51
PTAFR	18	8	9	10	5	13
	8	13	8	1	10	1
PTAR1	328	1254	343	1029	766	1246
	1107	1768	639	432	965	231
PTBP1	38	118	24	83	64	74
	115	189	71	44	69	27

PTBP2	111	519	169	506	334	419
	556	627	366	179	363	93
PTBP3	114	363	89	255	158	222
	247	416	224	117	178	88
PTCD1	0	5	0	9	4	4
	19	5	11	1	3	1
PTCD2	38	122	32	70	53	64
	116	93	51	29	85	13
PTCD3	381	1639	331	1025	932	1052
	1344	2258	801	557	983	355
PTCH1	67	61	48	91	32	37
	57	64	65	31	47	24
PTCH2	25	19	24	21	10	8
	16	10	8	1	2	1
PTCHD1	31	10	12	21	10	3
	8	10	9	0	11	2
PTCHD2	18	1	14	1	3	0
	0	1	15	0	9	0
PTCHD3	23	0	14	0	2	0
	0	0	11	0	0	0
PTCHD4	30	13	20	15	11	4
	15	14	17	2	13	0
PTCRA	7	0	2	0	1	0
	1	0	1	0	3	0
PTDSS1	77	259	61	213	111	206
	189	352	189	67	155	35
PTDSS2	12	45	15	19	19	21
	41	57	37	16	30	7
PTEN	169	1020	261	763	516	671
	648	1066	370	259	485	218
PTER	88	333	95	212	244	251
	254	378	155	83	177	47
PTF1A	4	0	1	0	0	0
	0	0	2	0	0	0
PTGDR	15	15	7	11	10	5
	11	8	10	7	18	3
PTGDR2	18	13	6	8	12	4
	33	20	8	5	16	5
PTGDS	25	91	15	81	35	69
	88	58	37	25	46	18
PTGER1	3	0	1	0	0	0
	0	1	1	0	0	0
PTGER2	6	4	1	3	1	0
	2	8	3	0	1	1
PTGER3	55	22	23	14	24	5
	14	19	33	3	29	8
PTGER4	20	68	14	51	29	44
	52	60	30	23	40	12
PTGES	11	5	3	14	3	5
	5	9	6	0	3	2
PTGES2	74	499	57	275	138	253
	513	474	176	124	214	51
PTGES3	205	1355	193	1079	683	862
	1128	1356	475	278	639	198
PTGES3L	42	280	46	154	99	104
	215	308	120	48	178	23

PTGES3L-AARSD1	50	270	33	115	75	108	239	248	97	58	133	27
PTGFR	26	47	57	92	25	67	16	98	40	8	34	16
PTGFRN	55	144	51	105	72	106	59	130	94	24	63	16
PTGIR	2	2	4	0	2	3	1	6	3	0	2	1
PTGIS	40	111	17	179	79	136	48	177	84	29	88	48
PTGR1	32	98	33	55	54	69	129	126	50	26	68	20
PTGR2	83	405	89	274	178	267	344	402	174	158	268	59
PTGS1	36	44	19	38	47	47	17	52	42	10	27	18
PTGS2	14	5	11	6	7	3	0	8	19	2	7	1
PTH	3	0	1	0	2	0	0	0	1	0	6	0
PTH1R	11	10	6	7	6	6	16	29	7	3	9	0
PTH2	0	0	3	0	0	0	0	0	1	0	0	0
PTH2R	19	0	15	1	4	0	0	0	11	0	8	0
PTHLH	14	0	13	13	5	0	0	4	10	1	6	0
PTK2	141	864	210	569	331	473	649	847	342	234	397	133
PTK2B	50	82	50	45	32	40	24	46	47	8	42	14
PTK6	9	1	3	0	3	1	1	0	4	1	1	0
PTK7	19	1	18	4	5	6	4	1	12	1	7	3
PTMA	120	962	162	568	451	678	894	1166	390	328	489	241
PTMS	14	86	19	57	27	50	50	51	27	17	14	7
PTN	11	16	9	17	6	2	11	6	18	5	14	2
PTOV1	34	256	22	111	89	120	179	202	102	75	97	35
PTP4A1	921	7034	839	4609	2058	3538	5235	5176	1576	1324	2618	737
PTP4A2	466	3151	443	2418	1254	1806	2019	2362	953	812	1116	422
PTP4A3	121	773	152	629	273	587	873	1538	577	220	680	159
PTPDC1	49	160	43	107	78	148	135	235	97	59	142	40
PTPLA	31	251	48	208	109	173	210	361	219	43	172	44
PTPLAD1	51	184	61	161	96	152	148	222	76	68	81	41

PTPLAD2	12	11	7	15	7	7
	3	14	11	5	8	1
PTPLB	26	50	22	45	34	45
	45	51	18	14	22	19
PTPMT1	51	205	33	164	84	145
	210	315	97	56	113	38
PTPN1	65	238	61	121	121	211
	219	257	110	73	148	51
PTPN11	396	2496	313	1513	960	1615
	1620	2191	822	728	1057	431
PTPN12	99	458	91	297	211	284
	440	389	185	148	209	82
PTPN13	124	120	93	165	84	71
	78	134	101	39	82	46
PTPN14	308	1337	348	1141	668	736
	862	1257	577	428	628	364
PTPN18	36	114	35	41	59	59
	76	101	36	32	53	19
PTPN2	45	216	46	123	77	156
	147	203	83	54	109	48
PTPN20A	10	26	13	30	8	42
	38	14	16	3	57	7
PTPN20B	27	58	24	46	23	64
	80	36	61	8	36	22
PTPN21	94	340	122	283	184	260
	262	311	153	94	188	65
PTPN22	24	3	14	4	5	5
	2	7	17	3	18	4
PTPN23	31	120	39	66	55	78
	81	98	39	14	50	23
PTPN3	228	907	212	559	387	533
	969	2002	541	330	785	227
PTPN4	356	1749	407	1541	874	1420
	1135	1588	843	389	967	202
PTPN5	15	4	17	3	4	0
	5	1	5	0	11	0
PTPN6	18	21	14	12	9	18
	12	28	12	7	13	7
PTPN7	18	4	13	5	7	2
	6	5	15	0	15	1
PTPN9	33	61	21	65	36	55
	67	97	57	26	67	15
PTPRA	124	452	140	401	189	371
	374	585	253	128	297	77
PTPRB	217	1004	117	688	468	412
	1147	1052	599	381	645	263
PTPRC	96	173	43	89	49	104
	56	153	104	35	83	21
PTPRCAP	3	5	2	3	1	6
	1	6	2	2	5	1
PTPRD	95	214	118	95	96	125
	50	153	96	21	82	24
PTPRE	43	75	27	41	27	68
	28	77	70	18	41	18
PTPRF	43	41	34	41	23	21
	16	26	15	5	21	39
PTPRG	132	461	116	329	205	291
	288	378	200	119	298	87

PTPRH	17	0	10	2	3	0
	1	1	4	0	6	0
PTPRJ	90	81	52	81	47	42
	56	66	72	22	55	8
PTPRK	137	289	116	240	143	220
	260	335	190	105	193	50
PTPRM	351	1818	276	939	787	1075
	1347	2236	878	613	1103	373
PTPRN	22	0	11	0	4	0
	0	0	10	0	4	0
PTPRN2	27	10	12	14	11	12
	11	22	12	5	13	5
PTPRO	56	11	51	9	15	19
	8	10	42	2	17	3
PTPRQ	97	53	72	30	57	49
	45	123	72	9	85	11
PTPRR	23	9	25	15	5	10
	10	4	24	8	11	2
PTPRS	96	306	103	228	184	177
	234	365	107	76	115	92
PTPRT	78	1	49	0	17	2
	0	3	41	0	19	0
PTPRU	38	113	35	71	54	40
	35	147	30	45	60	11
PTPRZ1	48	3	46	15	27	0
	10	1	47	2	22	1
PTRF	187	1201	233	622	591	762
	689	975	420	399	468	258
PTRH1	1	5	0	3	1	8
	3	11	2	2	0	0
PTRH2	11	28	12	22	22	29
	51	29	30	7	28	4
PTRHD1	15	55	14	45	36	93
	79	104	66	25	43	10
PTS	47	280	33	145	120	171
	246	322	109	55	130	42
PTTG1	18	7	4	7	6	2
	1	6	9	2	2	1
PTTG1IP	125	470	83	328	224	317
	334	492	165	153	251	100
PTTG2	0	0	2	0	0	0
	0	0	0	0	0	1
PTX3	8	5	3	6	8	4
	1	4	5	4	6	0
PTX4	1	0	0	0	0	0
	0	0	0	0	0	0
PUF60	55	249	44	143	98	155
	185	280	97	60	119	27
PUM1	201	1020	200	738	519	731
	777	1104	472	331	635	201
PUM2	213	1119	296	822	530	882
	884	1085	406	330	574	205
PURA	133	669	174	684	330	590
	544	661	301	179	351	123
PURB	181	726	174	686	380	577
	528	772	310	187	438	165
PURG	7	4	9	10	4	4
	2	7	5	1	6	1

PUS1	9	17	6	10	9	23
	26	25	10	6	10	3
PUS10	65	141	45	77	67	71
	99	214	57	56	99	36
PUS3	24	76	23	66	45	70
	86	77	46	42	59	21
PUS7	54	159	50	127	107	172
	127	184	82	51	104	32
PUS7L	64	144	59	107	83	133
	128	191	109	56	144	35
PUSL1	9	19	1	17	3	17
	10	18	6	3	6	3
PVALB	1	4	12	16	0	140
	9	1	1	1	3	0
PVR	85	429	84	241	161	411
	222	330	170	115	181	52
PVRIG	13	12	10	4	7	16
	13	30	5	13	21	2
PVRL1	19	8	19	18	4	7
	8	5	12	2	7	0
PVRL2	22	36	13	8	21	13
	30	33	21	5	26	3
PVRL3	67	141	67	90	78	101
	86	123	77	36	76	33
PVRL4	22	2	10	0	2	3
	0	0	3	0	4	0
PWP1	50	245	65	169	130	184
	210	275	135	96	178	55
PWP2	30	91	34	42	46	66
	81	141	58	31	59	18
PWWP2A	74	260	78	235	113	253
	201	288	129	88	179	50
PWWP2B	12	56	10	45	38	58
	31	64	35	13	24	10
PXDC1	18	38	17	28	29	47
	30	51	16	24	33	14
PXDN	71	176	34	106	120	83
	162	142	102	58	79	48
PXDNL	73	202	27	27	112	139
	41	280	40	43	244	12
PXK	57	97	39	61	62	72
	90	130	58	47	75	34
PXMP2	15	54	8	34	26	35
	98	116	41	16	60	6
PXMP4	54	102	36	86	49	82
	63	128	67	30	77	25
PXN	92	463	73	300	146	249
	297	482	200	103	205	53
PXT1	19	4	9	0	6	0
	2	1	9	0	4	0
PYCARD	1	3	2	4	3	4
	4	10	2	0	2	0
PYCR1	12	20	7	15	7	20
	2	22	19	6	9	4
PYCR2	35	195	26	97	52	110
	124	248	71	50	73	25
PYCRL	4	4	4	7	2	2
	1	3	11	0	4	2

PYGB	34	105	32	86	67	99
	62	153	63	27	45	29
PYGL	38	61	27	28	63	43
	31	46	21	17	24	22
PYGM	6129	37997	6737	30147	18215	30052
	33616	37138	22101	13296	20129	5773
PYGO1	83	261	78	227	157	319
	302	468	212	128	274	67
PYGO2	32	108	25	61	60	78
	66	102	48	22	46	23
PYHIN1	18	8	17	5	13	4
	12	4	12	0	16	0
PYROXD1	67	251	48	188	126	150
	197	310	104	75	163	66
PYROXD2	19	119	30	24	29	64
	55	54	21	6	23	10
PYURF	39	230	31	181	91	129
	252	250	110	67	107	32
PYY	5	0	1	0	1	2
	2	0	1	0	1	0
PZP	79	27	48	11	27	21
	17	19	44	12	32	3
QARS	146	787	129	621	235	559
	733	766	395	232	502	114
QDPR	24	309	35	263	85	159
	305	219	75	31	67	16
QKI	1501	7352	1275	5032	3609	5062
	4865	9270	3553	2383	4813	1559
QPCT	9	7	9	6	5	0
	10	4	18	0	5	1
QPCTL	9	5	5	1	4	1
	0	6	8	1	9	1
QPRT	4	14	3	6	1	7
	8	13	11	4	3	6
QRFP	1	0	0	0	0	0
	0	0	0	0	0	0
QRFPR	13	0	2	0	2	0
	0	3	5	0	1	0
QRICH1	103	500	82	329	236	358
	439	489	192	169	266	82
QRICH2	48	90	39	36	28	73
	39	67	34	15	39	9
QRSL1	110	477	88	205	181	254
	310	643	220	153	275	88
QSER1	203	910	236	720	453	678
	656	674	295	271	469	123
QSOX1	33	75	20	74	42	63
	80	114	43	32	58	29
QSOX2	25	48	22	40	22	25
	36	42	31	11	33	9
QTRT1	21	69	7	44	29	48
	42	89	29	18	41	12
QTRTD1	49	82	31	51	43	63
	52	99	32	34	68	17
R3HCC1	41	116	30	106	53	111
	136	212	96	76	102	39
R3HCC1L	49	157	54	124	82	143
	110	165	86	50	94	42

R3HDM1	130	629	109	400	254	363
	419	553	211	164	199	85
R3HDM2	158	730	151	474	218	437
	524	474	232	153	309	98
R3HDM4	16	73	10	55	18	61
	43	53	38	13	47	20
R3HDML	3	0	3	1	2	0
	0	0	9	0	1	0
RAB10	616	3589	716	4137	1648	3615
	3702	4253	1792	926	3335	602
RAB11A	140	820	208	536	327	457
	542	913	344	199	474	110
RAB11B	23	93	6	41	42	26
	60	65	22	16	28	12
RAB11FIP1	89	182	58	166	93	132
	150	203	97	67	104	39
RAB11FIP2	75	328	81	251	170	246
	228	357	162	77	195	61
RAB11FIP3	66	203	64	160	84	130
	96	134	69	48	92	38
RAB11FIP4	47	6	21	3	10	6
	2	5	22	0	11	2
RAB11FIP5	29	70	23	70	49	52
	66	101	60	24	44	20
RAB12	275	1538	247	1371	625	1225
	1640	2263	800	521	1078	335
RAB13	25	77	26	38	36	24
	53	77	20	17	19	7
RAB14	110	741	136	561	313	424
	592	737	264	137	350	102
RAB15	32	29	20	32	18	26
	22	51	36	7	15	4
RAB17	14	8	6	7	8	11
	4	10	12	3	13	1
RAB18	124	651	147	504	294	533
	531	770	365	200	446	117
RAB19	2	0	4	0	2	0
	0	0	4	0	1	0
RAB1A	124	596	121	460	288	413
	544	790	329	169	328	111
RAB1B	123	466	70	343	226	384
	477	615	288	146	298	78
RAB20	5	5	1	2	3	3
	5	9	2	0	3	1
RAB21	188	1017	171	803	574	671
	888	1336	472	256	619	231
RAB22A	73	379	92	293	166	273
	267	397	168	84	206	81
RAB23	68	227	66	169	114	202
	144	248	112	64	111	51
RAB24	20	61	6	46	20	68
	40	69	25	23	25	5
RAB25	2	0	1	1	1	0
	0	1	2	0	1	0
RAB26	1	1	2	2	3	0
	0	3	5	0	1	0
RAB27A	23	31	17	20	15	16
	16	21	22	8	22	8

RAB27B	44	10	20	22	20	17
	3	13	33	2	20	3
RAB28	24	76	20	77	30	58
	69	148	50	25	58	20
RAB2A	227	945	239	859	372	571
	682	1083	597	276	665	151
RAB2B	46	103	18	73	59	106
	93	137	42	31	57	35
RAB30	52	177	53	175	132	136
	197	224	132	51	108	29
RAB31	19	66	26	89	39	68
	47	104	50	26	43	22
RAB32	14	16	5	11	15	25
	12	28	9	6	10	2
RAB33A	1	0	0	0	0	0
	0	0	0	0	1	0
RAB33B	41	138	50	126	80	120
	156	192	107	49	105	27
RAB34	9	19	9	30	5	11
	12	34	24	9	18	3
RAB35	13	60	13	41	26	47
	58	74	29	17	31	17
RAB36	20	1	7	1	1	0
	3	0	8	0	1	1
RAB37	18	6	6	5	4	2
	4	8	15	0	7	1
RAB38	10	8	6	2	5	5
	3	19	3	2	14	1
RAB39A	9	6	10	5	2	2
	8	4	2	0	4	1
RAB39B	13	3	6	1	4	0
	1	1	3	0	4	0
RAB3A	11	8	4	6	3	6
	8	3	3	0	4	0
RAB3B	58	1	24	0	11	1
	0	0	23	0	13	0
RAB3C	10	4	4	0	2	0
	0	0	6	0	3	0
RAB3D	22	66	10	30	15	28
	34	53	24	13	19	7
RAB3GAP1	286	1186	303	809	629	928
	862	1389	579	388	724	261
RAB3GAP2	175	618	188	502	283	483
	501	658	351	205	362	147
RAB3IL1	13	27	9	25	11	8
	11	25	7	4	11	1
RAB3IP	64	91	59	40	50	47
	69	116	76	35	83	25
RAB40A	12	6	4	0	8	4
	3	2	2	1	5	1
RAB40AL	3	2	1	1	1	1
	1	2	1	0	1	1
RAB40B	47	236	65	255	85	276
	252	282	139	55	166	50
RAB40C	14	53	15	33	23	41
	72	49	31	16	31	7
RAB41	9	0	2	0	0	0
	1	0	2	0	2	0

RAB42	6	0	1	0	2	0
	0	1	2	0	2	1
RAB43	1	1	0	6	1	0
	0	4	1	0	0	1
RAB44	15	2	10	3	2	0
	0	1	11	0	7	0
RAB4A	80	418	98	320	162	380
	527	476	201	120	240	77
RAB4B	6	9	3	7	6	8
	8	3	5	0	10	0
RAB5A	92	602	98	373	253	404
	414	501	167	109	264	72
RAB5B	188	1268	217	817	549	813
	1091	1026	439	293	565	178
RAB5C	36	93	36	70	50	68
	73	90	49	43	51	22
RAB6A	226	1176	204	909	553	800
	936	1135	483	348	616	169
RAB6B	46	40	20	21	21	29
	19	23	19	2	14	5
RAB6C	12	6	11	3	12	4
	6	6	6	1	4	0
RAB7A	126	1241	111	660	481	582
	1128	808	224	243	408	130
RAB7L1	30	40	15	47	36	82
	49	39	38	12	35	6
RAB8A	38	114	30	59	46	71
	87	109	37	35	59	15
RAB8B	39	135	54	139	82	141
	68	132	93	47	94	27
RAB9A	32	101	20	95	50	109
	104	222	74	46	71	29
RAB9B	61	156	88	198	142	294
	222	388	196	98	229	65
RABAC1	8	43	4	62	25	60
	51	84	43	20	44	8
RABEP1	480	2443	388	1980	1118	2212
	1559	1705	914	604	1255	372
RABEP2	8	18	0	15	10	5
	8	10	16	7	11	2
RABEPK	25	62	11	41	19	45
	51	72	30	21	47	11
RABGAP1	153	804	163	580	405	565
	594	666	257	240	368	144
RABGAP1L	134	341	132	282	154	203
	227	306	220	90	214	56
RABGEF1	103	350	100	268	208	291
	305	458	195	120	239	62
RABGGTA	20	48	15	34	19	44
	42	49	31	6	45	9
RABGGTB	136	692	132	612	326	544
	641	1114	398	230	459	131
RABIF	15	18	10	25	3	34
	17	27	18	7	17	5
RABL2A	26	20	6	9	13	3
	10	24	13	3	9	2
RABL2B	21	21	14	13	15	10
	12	20	18	2	11	6

RABL3	78	248	52	166	119	199
	205	324	126	59	124	39
RABL5	33	85	17	61	25	66
	79	109	51	25	61	6
RABL6	111	536	99	218	189	304
	353	539	207	137	273	83
RAC1	113	877	106	558	350	526
	740	633	322	244	385	120
RAC2	9	9	4	3	5	5
	5	9	6	1	10	2
RAC3	5	0	3	0	4	0
	0	1	2	0	0	0
RACGAP1	30	23	18	37	31	27
	32	35	29	3	31	6
RAD1	71	268	77	233	134	279
	301	306	149	106	185	54
RAD17	72	313	75	212	160	244
	259	290	144	106	201	68
RAD18	46	66	44	60	53	47
	62	63	54	15	61	17
RAD21	197	1186	208	938	571	759
	1027	1440	541	400	718	252
RAD21L1	19	0	14	1	3	2
	0	3	13	0	6	0
RAD23A	271	1786	193	826	633	1087
	1538	1259	539	528	900	258
RAD23B	489	2363	480	1725	1365	1919
	1586	2836	1096	831	1433	535
RAD50	156	972	192	632	379	607
	529	833	309	274	412	147
RAD51	30	3	12	2	7	11
	2	4	19	0	8	3
RAD51AP1	19	12	17	14	15	23
	23	27	27	9	31	5
RAD51AP2	11	5	8	3	11	7
	1	3	11	0	7	2
RAD51B	38	32	21	24	17	29
	14	16	27	11	16	5
RAD51C	24	122	29	94	47	83
	115	121	72	38	70	16
RAD51D	5	15	13	18	6	11
	7	22	18	5	13	3
RAD52	70	170	72	109	123	124
	132	308	104	76	94	44
RAD54B	81	275	61	170	143	152
	111	205	134	71	148	48
RAD54L	26	6	13	2	6	6
	0	3	11	0	6	0
RAD54L2	79	217	69	135	79	103
	152	183	102	80	94	43
RAD9A	7	16	4	11	8	9
	13	23	19	9	7	6
RAD9B	16	11	17	9	6	20
	10	19	10	2	12	2
RADIL	9	6	4	3	4	3
	6	13	7	3	12	2
RAE1	28	62	25	59	30	53
	53	98	29	32	48	17

RAET1E	16	3	8	3	7	0
	2	0	5	2	1	1
RAET1G	8	1	4	0	1	1
	1	0	2	0	1	1
RAET1L	3	0	1	0	0	0
	0	0	1	0	1	0
RAF1	300	1331	270	1280	856	1097
	1014	1557	672	467	879	206
RAG1	34	11	14	4	11	5
	8	13	30	2	9	6
RAG2	22	0	13	0	5	0
	1	0	12	0	6	0
RAI1	22	53	28	49	26	38
	38	33	15	14	21	12
RAI14	75	80	52	124	40	52
	49	35	40	23	30	22
RAI2	19	45	2	32	14	30
	32	30	13	6	23	6
RALA	45	278	56	173	90	94
	153	162	81	49	91	23
RALB	42	214	23	115	86	116
	153	161	66	52	86	43
RALBP1	163	810	172	554	367	618
	676	843	304	280	428	137
RALGAPA1	409	1507	490	1593	705	1125
	1217	1745	733	475	903	316
RALGAPA2	528	2374	455	1603	1284	1578
	1719	2466	1156	741	1177	417
RALGAPB	200	756	202	623	384	550
	659	848	411	243	488	145
RALGDS	54	148	31	102	85	130
	124	179	104	60	80	37
RALGPS1	79	148	72	110	61	120
	124	138	94	48	122	14
RALGPS2	55	129	42	80	73	81
	78	112	90	43	59	22
RALY	71	469	111	371	248	365
	399	489	229	153	265	92
RALYL	21	3	23	3	12	4
	0	1	13	2	5	0
RAMP1	55	163	28	101	74	83
	238	253	88	105	101	36
RAMP2	19	37	7	28	12	11
	39	38	19	11	19	9
RAMP3	7	20	7	24	17	22
	19	41	16	14	31	4
RAN	123	707	108	495	215	512
	691	966	415	182	417	107
RANBP1	40	146	33	103	55	104
	134	247	76	43	86	32
RANBP10	59	180	36	110	60	115
	127	149	72	53	83	28
RANBP17	48	26	38	28	23	10
	15	21	45	6	33	6
RANBP2	762	4412	1003	3448	2375	3246
	2777	4024	1713	1398	2407	768
RANBP3	45	143	32	88	68	96
	110	140	75	51	89	27

RANBP3L	26	7	18	13	11	8
	15	13	18	3	19	5
RANBP6	175	863	159	589	445	556
	617	880	362	241	536	135
RANBP9	153	702	156	631	352	581
	723	882	317	242	509	153
RANGAP1	28	89	27	69	53	50
	79	127	47	21	51	11
RANGRF	11	14	2	1	5	4
	26	17	10	4	13	3
RAP1A	61	446	57	340	136	181
	361	334	107	82	165	45
RAP1B	99	383	104	298	176	261
	358	572	252	144	236	86
RAP1GAP	27	1	16	1	9	1
	1	0	5	0	7	0
RAP1GAP2	40	15	23	14	7	17
	11	32	25	8	24	3
RAP1GDS1	33	99	32	67	54	80
	49	53	58	24	39	15
RAP2A	75	397	68	387	231	282
	246	378	127	116	192	62
RAP2B	56	94	40	95	43	74
	80	95	74	25	72	21
RAP2C	65	354	88	336	176	232
	319	473	155	99	227	56
RAPGEF1	180	1366	221	754	464	714
	1002	789	369	285	379	155
RAPGEF2	229	858	174	616	440	614
	879	1130	477	349	512	187
RAPGEF3	55	125	31	57	48	46
	108	104	75	30	81	27
RAPGEF4	54	65	34	37	40	42
	69	77	48	20	51	19
RAPGEF5	83	237	78	185	108	113
	270	233	143	71	141	44
RAPGEF6	192	533	217	396	258	415
	400	570	376	214	372	111
RAPGEFL1	25	30	32	23	21	33
	12	37	35	4	35	3
RAPH1	145	1004	388	710	337	621
	568	640	355	132	267	100
RAPSN	49	200	44	169	93	208
	184	271	117	91	135	37
RARA	21	77	23	41	30	66
	47	42	31	24	40	14
RARB	34	116	29	79	56	41
	51	109	59	27	72	12
RARG	23	58	19	34	35	26
	30	46	28	9	22	4
RARRES1	5	1	1	3	5	0
	2	11	8	1	6	0
RARRES2	8	15	4	14	10	9
	11	13	6	7	5	9
RARRES3	13	34	10	22	15	8
	12	31	10	6	10	2
RARS	106	355	84	274	208	348
	332	644	248	192	319	99

RARS2	58	268	65	211	119	243
	202	317	146	82	195	46
RASA1	128	488	125	381	261	387
	331	507	289	117	313	84
RASA2	54	168	91	130	90	155
	159	176	93	60	121	33
RASA3	56	200	66	116	98	109
	164	197	94	38	108	34
RASA4	309	770	160	417	611	787
	518	930	688	355	596	164
RASA4B	170	555	98	273	261	411
	490	522	314	325	357	102
RASAL1	23	0	7	0	3	0
	0	0	7	0	3	0
RASAL2	206	658	148	498	423	436
	550	717	425	267	480	159
RASAL3	13	3	11	5	1	1
	3	6	11	1	3	1
RASD1	2	12	5	9	8	8
	13	10	9	5	6	4
RASD2	19	25	12	13	15	23
	24	18	14	2	9	2
RASEF	27	0	11	0	7	1
	1	2	15	0	15	0
RASGEF1A	17	4	15	2	5	5
	6	6	9	2	10	1
RASGEF1B	32	61	22	29	19	19
	49	87	65	23	58	7
RASGEF1C	7	0	4	0	1	0
	0	0	1	0	2	0
RASGRF1	43	1	21	0	7	2
	0	0	21	0	13	1
RASGRF2	112	223	65	97	92	97
	218	241	150	97	119	51
RASGRP1	29	31	16	7	17	9
	6	11	34	3	12	3
RASGRP2	29	31	14	21	18	20
	23	50	20	8	22	8
RASGRP3	218	970	212	680	498	670
	868	1162	382	271	560	128
RASGRP4	14	5	5	7	3	9
	6	12	11	2	10	0
RASIP1	17	30	4	10	7	10
	16	19	22	9	21	4
RASL10A	0	1	0	1	2	0
	2	3	3	0	2	1
RASL10B	26	49	20	23	31	46
	89	114	29	20	43	16
RASL11A	12	13	4	3	12	1
	8	10	7	5	1	6
RASL11B	13	1	9	5	8	3
	4	0	8	0	2	2
RASL12	22	86	8	79	19	62
	71	79	23	19	55	15
RASSF1	12	27	12	38	18	35
	34	65	13	9	24	5
RASSF10	7	12	6	5	4	3
	5	4	7	3	8	2

RASSF2	66	96	42	144	34	54
	52	81	44	24	38	18
RASSF3	48	238	47	437	185	128
	248	154	60	32	100	44
RASSF4	40	160	29	63	77	106
	142	205	104	55	65	18
RASSF5	15	26	17	23	12	23
	28	24	23	4	25	1
RASSF6	20	2	27	0	9	0
	4	4	27	0	12	0
RASSF7	1	1	0	0	0	2
	0	3	2	1	2	0
RASSF8	157	808	154	726	499	590
	540	758	303	226	434	97
RASSF9	15	41	18	25	17	17
	25	45	28	10	21	6
RAVER1	9	24	8	10	9	8
	21	17	9	12	12	7
RAVER2	110	570	118	416	269	463
	498	552	292	164	276	86
RAX	6	0	12	0	0	0
	0	0	3	0	3	0
RAX2	3	0	4	0	0	0
	0	0	2	0	0	0
RB1	152	888	197	686	419	637
	612	744	370	195	415	134
RB1CC1	408	1633	321	1410	969	1343
	1497	2110	937	763	1376	511
RBAK	5	111	3	72	33	142
	88	104	24	36	44	44
RBAK-LOC389458		3	5	3	8	3
	4	4	4	5	5	6
	0					
RBBP4	221	799	191	698	453	590
	659	939	366	289	511	135
RBBP5	62	265	70	176	138	186
	224	323	140	82	148	54
RBBP6	129	700	145	437	306	499
	581	625	266	210	357	134
RBBP7	159	661	143	496	351	508
	670	785	325	236	403	114
RBBP8	33	23	34	29	22	23
	26	31	36	13	26	11
RBBP9	47	75	17	48	36	57
	41	47	27	13	28	9
RBCK1	41	112	21	69	43	76
	127	137	63	51	48	37
RBFA	50	213	51	90	78	90
	134	197	94	59	91	27
RBFOX1	254	1541	334	979	621	1208
	1123	1646	731	527	810	246
RBFOX2	694	2867	792	2163	1473	2328
	2072	3353	1434	763	1889	489
RBFOX3	5	0	9	1	3	0
	0	1	1	0	3	0
RBKS	24	44	13	19	17	23
	36	38	22	16	26	8

RBL1	158	445	126	273	231	338
	384	606	190	160	358	102
RBL2	219	1030	200	767	477	770
	745	1248	532	342	612	229
RBM10	46	191	25	94	76	127
	124	211	67	63	96	29
RBM11	11	2	6	1	1	0
	0	1	7	1	2	0
RBM12	4	18	3	12	16	18
	20	35	29	6	41	2
RBM12B	101	516	136	298	240	289
	317	589	216	156	292	109
RBM14	3	9	5	7	8	6
	11	34	14	10	13	4
RBM14-RBM4	19	46	13	37	48	65
	45	78	33	16	31	20
RBM15	67	333	88	224	188	264
	290	377	154	116	215	81
RBM15B	46	137	35	89	55	91
	104	147	57	35	83	30
RBM17	161	722	131	451	340	553
	607	701	291	249	390	117
RBM18	108	589	117	526	249	428
	521	646	305	165	331	97
RBM19	74	281	66	147	112	200
	170	271	128	103	136	49
RBM20	137	576	159	423	350	558
	404	344	164	109	177	60
RBM22	99	316	78	220	180	238
	340	420	169	101	235	68
RBM23	64	279	63	154	119	189
	241	342	122	74	187	61
RBM24	918	5063	1060	4026	2208	4841
	4978	6882	2803	1642	3604	906
RBM25	200	917	205	687	430	633
	729	1082	426	358	622	156
RBM26	133	684	208	458	374	548
	592	827	319	234	381	113
RBM27	163	670	159	436	354	472
	478	641	358	181	380	127
RBM28	101	398	99	213	149	261
	326	532	206	140	224	76
RBM3	88	684	317	1046	137	784
	836	1138	694	200	728	106
RBM33	340	1565	364	1022	605	1143
	1296	2010	773	490	923	294
RBM34	58	285	62	189	134	204
	224	302	149	90	139	42
RBM38	113	309	54	313	231	345
	417	514	263	181	223	91
RBM39	449	2269	574	1598	1069	1757
	2035	3348	1369	842	1648	475
RBM4	58	414	85	247	178	303
	278	475	212	138	207	77
RBM41	114	435	111	371	255	342
	358	326	183	147	224	97
RBM42	29	84	20	63	44	64
	73	78	47	29	50	27

RBM43	28	71	24	67	28	56
	44	31	45	16	38	13
RBM44	21	31	28	22	22	25
	26	50	30	4	12	3
RBM45	27	79	24	65	35	52
	61	141	53	13	49	20
RBM46	14	0	8	0	3	0
	0	0	13	1	7	0
RBM47	20	13	6	14	13	10
	11	28	15	2	8	3
RBM48	44	167	55	112	79	114
	160	201	67	60	118	32
RBM4B	25	71	21	67	41	64
	68	118	55	22	46	18
RBM5	350	1497	370	973	699	1048
	1370	1957	763	571	1000	298
RBM6	203	966	235	630	357	719
	766	1280	425	344	522	201
RBM7	24	107	26	86	51	90
	101	164	81	42	69	13
RBM8A	143	750	146	551	325	406
	738	908	309	212	487	129
RBMS1	92	408	94	288	214	269
	280	390	172	113	214	88
RBMS2	88	315	52	242	152	182
	389	350	196	126	203	81
RBMS3	201	831	258	694	414	536
	624	868	366	274	519	192
RBMX	250	1089	301	762	499	775
	1067	1564	633	458	855	288
RBMX2	33	81	25	60	53	83
	70	122	38	29	67	16
RBMXL1	0	1	0	0	1	1
	3	3	1	2	0	1
RBMXL2	3	0	1	0	1	0
	1	0	1	0	2	0
RBMXL3	4	0	1	0	0	0
	0	0	0	0	0	0
RBMY1A1	0	0	6	0	0	0
	0	0	9	0	9	0
RBMY1B	0	0	6	0	0	0
	0	0	4	1	8	0
RBMY1D	0	0	0	0	0	0
	0	0	8	0	5	0
RBMY1E	0	0	20	0	0	0
	0	0	18	0	1	0
RBMY1F	0	0	10	0	0	0
	0	0	7	0	6	0
RBMY1J	0	0	1	0	0	0
	0	0	10	0	3	0
RBP1	12	24	6	5	10	15
	24	13	13	1	7	1
RBP2	3	0	1	0	0	0
	1	1	2	0	0	0
RBP3	7	0	1	0	1	0
	0	0	4	0	1	0
RBP4	6	9	2	0	5	3
	2	2	0	0	2	4

RBP5	13	2	4	1	0	1
	6	3	6	3	9	0
RBP7	2	15	3	11	3	8
	44	13	7	3	17	3
RBPJ	214	789	237	776	428	720
	659	1126	536	258	526	177
RBPJL	17	0	6	0	2	0
	0	0	2	0	1	0
RBPMS	26	52	15	43	19	32
	24	55	40	17	35	9
RBPMS2	15	6	6	4	3	2
	1	5	5	1	6	2
RBX1	42	195	32	160	79	116
	174	347	164	59	141	31
RC3H1	315	1162	277	891	569	983
	1739	1779	689	448	992	337
RC3H2	117	424	107	324	264	273
	361	452	216	139	256	109
RCAN1	35	126	25	104	75	106
	126	196	82	58	41	34
RCAN2	111	556	101	525	94	318
	480	819	305	109	384	85
RCAN3	25	23	12	17	14	14
	28	26	19	5	18	4
RCBTB1	47	168	53	145	62	114
	131	136	131	45	107	21
RCBTB2	47	135	51	106	94	96
	87	86	66	37	73	23
RCC1	20	68	21	42	35	56
	42	72	34	21	37	10
RCC2	48	68	45	51	38	61
	46	81	45	30	41	23
RCCD1	17	58	7	36	33	62
	34	57	41	21	25	8
RCE1	18	11	14	7	10	9
	8	26	9	5	10	1
RCHY1	94	468	106	362	175	348
	352	554	212	128	256	62
RCL1	93	380	95	257	187	255
	253	403	188	143	198	53
RCN1	56	369	65	272	167	333
	278	318	214	88	182	36
RCN2	91	431	89	406	163	348
	405	492	292	106	281	74
RCN3	11	29	13	29	16	30
	17	29	15	7	11	5
RCOR1	85	344	88	300	162	272
	266	293	133	93	155	67
RCOR2	10	15	7	4	10	10
	10	5	11	1	6	4
RCOR3	209	1220	228	983	519	840
	914	1239	581	346	664	199
RCSD1	179	1084	212	897	468	834
	791	827	491	303	548	157
RCVRN	9	0	2	0	3	1
	0	0	7	0	1	0
RD3	13	1	8	1	1	0
	0	0	8	0	4	0

RD3L	6	4	1	2	6	0
	4	0	6	0	6	0
RDBP	30	115	13	63	34	70
	86	132	58	26	71	13
RDH10	42	95	15	91	71	90
	55	95	51	23	43	16
RDH11	31	99	30	78	65	107
	80	116	59	30	62	20
RDH12	12	0	13	1	3	2
	0	0	4	0	3	0
RDH13	17	34	13	25	21	22
	45	71	27	17	25	5
RDH14	15	270	55	229	120	182
	349	419	140	97	162	29
RDH16	15	15	8	2	7	4
	9	8	10	6	6	3
RDH5	21	111	9	32	62	56
	79	116	46	29	35	24
RDH8	5	0	3	1	0	0
	0	0	1	0	0	0
RDM1	9	4	7	1	1	2
	0	5	3	1	8	1
RDX	394	2538	468	1670	1087	1643
	1507	2311	805	688	1134	422
REC8	14	5	9	7	4	4
	7	10	9	4	7	1
RECK	39	69	26	89	63	87
	78	156	55	41	69	31
RECQL	91	247	74	224	162	213
	164	332	158	115	189	65
RECQL4	12	7	4	1	0	2
	2	0	6	2	2	0
RECQL5	30	82	28	35	44	47
	70	81	40	35	53	16
REEP1	102	431	113	328	221	358
	324	356	205	113	241	56
REEP2	17	25	7	13	14	18
	45	18	39	13	26	6
REEP3	81	319	78	286	191	272
	282	437	166	133	175	75
REEP4	11	5	7	3	3	3
	15	10	3	2	9	0
REEP5	350	1968	379	1678	718	1548
	1557	1532	833	429	782	278
REEP6	3	3	3	0	1	4
	4	2	1	0	2	0
REG1A	9	0	6	0	3	0
	0	0	1	0	2	0
REG1B	3	0	7	0	2	0
	0	0	3	0	2	0
REG3A	6	0	4	0	2	0
	0	0	1	0	1	0
REG3G	7	0	7	0	1	0
	0	0	3	0	1	0
REG4	18	0	12	0	2	1
	0	0	8	0	7	0
REL	41	126	27	83	48	76
	115	129	42	27	65	18

RELA	38	113	35	90	49	78
	97	102	46	45	73	22
RELB	12	16	8	16	9	15
	10	12	10	3	11	3
RELL1	68	252	46	191	121	228
	270	517	164	111	230	88
RELL2	7	1	5	2	0	0
	0	2	7	0	0	0
RELN	108	17	73	42	41	11
	16	24	71	17	43	20
RELT	21	38	10	29	30	57
	49	73	29	13	25	19
REM1	10	8	7	3	7	8
	16	7	3	3	8	2
REM2	8	0	8	1	1	1
	0	1	4	1	2	0
REN	8	0	4	0	1	0
	0	0	5	0	4	0
RENBP	8	6	2	5	6	5
	11	16	6	1	1	3
REP15	3	12	0	9	4	6
	10	12	7	3	7	3
REPIN1	102	391	63	278	255	350
	346	387	225	108	249	62
REPS1	86	264	78	177	124	193
	206	362	176	104	181	46
REPS2	66	16	19	23	21	19
	21	27	23	7	14	4
RER1	78	279	54	220	175	296
	251	427	178	114	171	50
RERE	200	1078	192	693	457	625
	834	1188	337	276	444	184
RERG	25	35	14	46	32	50
	32	64	28	16	46	23
RERGL	14	20	12	18	7	9
	12	29	14	7	8	8
RESP18	8	0	5	1	1	0
	0	0	3	0	2	0
REST	136	661	152	546	319	508
	461	447	228	137	303	104
RET	54	151	25	66	99	128
	56	118	69	28	49	25
RETN	2	0	0	0	0	1
	0	0	1	0	0	0
RETNLB	3	0	2	0	1	0
	0	0	0	0	0	0
RETSAT	85	305	57	200	155	217
	344	501	132	98	223	90
REV1	142	704	173	530	323	440
	760	850	375	262	491	134
REV3L	314	1503	447	1109	950	1325
	998	1662	663	557	724	343
REXO1	21	48	14	27	21	24
	30	38	21	20	14	18
REXO1L1	801	4	485	3	188	0
	5	3	356	2	313	1
REXO2	70	332	62	221	130	242
	339	522	157	86	164	80

REXO4	43	212	31	121	98	171
	165	164	72	65	83	28
RFC1	125	420	127	327	218	327
	317	397	213	135	250	89
RFC2	29	59	18	61	30	24
	65	88	42	18	31	8
RFC3	41	70	39	56	44	58
	43	97	63	24	74	19
RFC4	16	64	13	38	34	46
	57	76	40	16	27	9
RFC5	31	53	31	39	37	42
	30	90	55	33	39	12
RFESD	26	55	17	21	29	37
	34	53	43	16	45	4
RFFL	46	73	22	61	35	70
	80	104	62	23	74	27
RFK	42	130	29	179	94	148
	157	188	106	51	105	32
RFNG	9	40	5	21	24	39
	43	44	23	12	24	7
RFPL1	4	0	3	0	0	0
	0	0	4	0	0	0
RFPL2	19	0	9	0	2	0
	0	0	4	0	3	0
RFPL3	3	0	4	0	0	0
	0	0	3	0	2	0
RFPL4A	9	0	5	0	2	0
	0	0	8	0	3	0
RFPL4B	15	0	11	1	5	1
	0	0	8	0	0	0
RFT1	45	71	40	60	69	77
	56	118	53	27	64	15
RFTN1	46	154	27	94	71	97
	208	163	82	60	108	47
RFTN2	32	54	24	44	27	46
	50	56	35	17	37	23
RFWD2	80	315	61	213	142	208
	197	307	155	80	151	67
RFWD3	33	42	19	36	30	29
	42	79	44	18	56	14
RFX1	12	18	6	13	9	7
	16	12	6	6	11	2
RFX2	16	11	14	9	13	11
	6	9	17	10	14	5
RFX3	48	112	45	102	46	69
	113	121	88	49	75	32
RFX4	36	0	11	0	5	2
	1	0	9	0	11	0
RFX5	64	243	60	223	97	141
	127	227	81	70	115	33
RFX6	22	0	18	0	5	1
	0	0	22	0	8	0
RFX7	155	750	229	730	404	552
	561	671	274	213	392	105
RFX8	30	1	7	6	10	5
	3	2	10	0	6	0
RFXANK	15	49	12	38	23	40
	32	77	35	12	26	7

RFXAP	29	100	41	59	37	64
	63	69	52	31	62	16
RGAG1	22	2	16	2	2	0
	0	0	8	0	5	2
RGAG4	25	25	7	6	10	15
	9	10	10	4	8	1
RGCC	17	84	10	46	21	49
	149	131	70	30	48	25
RGL1	78	108	65	168	108	123
	90	189	88	35	69	38
RGL2	52	212	38	121	78	122
	183	255	109	71	131	51
RGL3	21	3	7	6	3	6
	3	10	6	3	7	3
RGL4	20	3	19	5	12	8
	1	6	12	0	3	0
RGMA	84	323	40	265	171	217
	332	271	118	95	177	56
RGMB	118	468	92	436	256	411
	395	400	251	151	280	91
RGN	31	61	21	113	40	94
	111	67	64	20	55	18
RGP1	29	134	37	59	51	75
	82	109	58	23	53	25
RGPD1	34	7	39	7	16	30
	2	9	13	21	21	6
RGPD2	175	326	115	171	162	237
	117	182	185	224	282	57
RGPD3	62	30	36	43	47	28
	57	34	78	9	61	7
RGPD4	46	2	23	14	26	22
	18	9	53	2	28	0
RGPD5	26	92	34	92	34	74
	89	55	46	61	84	29
RGPD6	18	97	33	91	41	73
	76	125	96	30	99	25
RGPD8	264	1020	331	1178	420	925
	965	1516	715	417	1021	230
RGR	20	0	10	5	9	1
	0	4	10	0	9	0
RGS1	10	4	9	6	5	5
	2	3	11	0	5	1
RGS10	12	42	19	36	24	22
	62	53	44	10	19	7
RGS11	9	8	4	10	7	18
	6	16	6	1	6	6
RGS12	52	113	34	61	56	61
	88	79	50	37	53	18
RGS13	8	4	8	1	6	1
	0	5	9	0	9	0
RGS14	12	17	20	5	18	30
	6	10	13	0	14	1
RGS16	11	11	5	7	6	10
	7	5	7	0	11	3
RGS17	10	2	8	1	5	1
	0	2	3	0	5	0
RGS18	6	15	3	11	2	7
	10	11	7	5	4	3

RGS19	2	2	2	2	4	1
	7	4	2	2	5	0
RGS2	11	50	17	40	21	32
	27	15	10	3	14	6
RGS20	13	0	6	0	1	1
	0	0	2	0	7	0
RGS21	9	0	8	0	1	0
	0	0	7	0	1	0
RGS22	36	6	28	9	13	5
	5	10	40	2	22	2
RGS3	89	265	51	145	167	152
	266	347	106	51	107	57
RGS4	35	4	15	5	14	2
	4	4	23	0	13	0
RGS5	487	3751	335	2853	1433	2336
	3704	3419	1873	1196	2364	563
RGS6	50	32	38	21	16	12
	21	19	24	7	18	7
RGS7	33	0	7	0	8	0
	0	0	17	0	12	0
RGS7BP	27	11	21	9	17	4
	4	4	20	3	12	4
RGS8	7	0	5	0	1	0
	0	0	5	0	1	0
RGS9	19	4	13	1	10	8
	0	1	8	1	7	4
RGS9BP	30	117	57	102	49	85
	133	150	36	43	73	42
RGSL1	32	1	23	0	7	0
	0	0	11	0	14	0
RHAG	11	1	3	1	2	2
	0	0	7	0	7	0
RHBDD1	59	228	72	160	99	148
	157	208	100	53	102	38
RHBDD2	9	19	5	14	7	10
	12	25	16	4	12	6
RHBDD3	12	23	7	15	13	18
	28	34	14	5	11	3
RHBDF1	34	86	20	57	30	81
	33	78	37	14	47	10
RHBDF2	20	35	19	14	15	30
	14	48	19	8	17	9
RHBDL1	7	1	4	0	0	1
	0	0	4	0	1	0
RHBDL2	14	2	7	2	3	2
	1	5	11	2	6	1
RHBDL3	20	0	14	0	9	0
	1	0	8	0	4	1
RHBG	9	0	6	0	0	0
	0	0	2	0	4	0
RHCE	21	8	9	4	2	4
	2	12	12	1	5	0
RHCG	9	0	3	0	2	0
	0	0	1	0	1	1
RHD	17	3	5	1	2	0
	1	3	5	2	9	0
RHEB	119	518	108	336	246	331
	518	787	250	147	315	94

RHEBL1	9	4	7	1	4	0
	0	0	4	2	3	0
RHNO1	15	44	17	37	23	38
	36	46	21	13	24	4
RHO	11	0	2	0	1	0
	0	0	6	0	3	0
RHOA	228	1707	309	1161	576	1077
	1103	1431	614	351	739	232
RHOB	70	270	74	239	209	255
	351	509	265	112	163	188
RHOBTB1	698	3143	592	2557	1902	2566
	3555	4260	2562	1371	2517	403
RHOBTB2	54	157	41	73	63	119
	117	97	59	51	70	22
RHOBTB3	271	1120	372	1046	595	695
	1021	1847	655	376	730	258
RHOC	16	150	24	90	58	91
	126	233	72	33	63	26
RHOD	3	6	2	1	3	9
	12	17	9	4	1	0
RHOF	8	6	5	9	2	7
	12	5	5	2	4	2
RHOG	9	10	7	9	6	15
	9	11	8	5	5	6
RHOH	6	4	5	0	2	2
	3	1	3	0	3	0
RHOJ	47	120	23	75	39	56
	82	58	42	21	54	11
RHOQ	520	2961	399	2328	1211	1943
	2282	2684	998	830	1411	507
RHOT1	200	767	193	620	375	662
	738	838	411	285	486	135
RHOT2	22	74	11	52	36	46
	74	113	66	19	64	22
RHOU	57	201	50	123	181	145
	167	302	84	39	96	30
RHOV	7	0	6	0	1	0
	0	0	4	0	4	0
RHOXF1	4	0	0	1	1	0
	0	0	1	0	0	1
RHOXF2	5	0	6	1	3	0
	0	1	1	0	0	0
RHOXF2B	18	1	2	1	1	0
	0	0	1	0	1	0
RHPN1	6	4	4	2	2	2
	0	1	1	0	1	0
RHPN2	41	1	17	1	2	1
	1	9	14	1	10	0
RIBC1	25	10	5	2	5	0
	0	5	12	1	1	0
RIBC2	8	1	3	0	0	0
	0	0	7	0	3	0
RIC3	61	130	38	91	54	110
	94	159	109	32	101	29
RIC8A	33	117	33	69	52	94
	79	98	49	27	55	10
RIC8B	85	315	57	183	150	195
	232	283	102	73	158	27

RICTOR	328	1245	309	1022	696	1038
	1471	2051	928	501	996	342
RIF1	374	1247	363	996	763	1010
	1095	1886	720	543	895	284
RIIAD1	2	2	2	0	2	0
	3	1	1	1	2	0
RILP	20	123	21	75	41	63
	152	135	79	44	88	25
RILPL1	56	336	46	158	124	214
	272	263	97	112	152	46
RILPL2	5	22	1	11	11	14
	15	26	7	5	15	4
RIMBP2	36	2	14	2	6	8
	1	2	13	0	4	0
RIMBP3	29	0	8	0	3	0
	0	0	6	0	1	0
RIMBP3B	4	0	2	0	1	1
	0	1	0	0	3	0
RIMBP3C	2	0	2	0	0	0
	0	0	2	0	0	0
RIMKLA	68	17	42	22	18	33
	20	22	41	3	25	8
RIMKLB	87	336	138	239	108	291
	229	403	182	98	205	69
RIMS1	74	11	32	41	35	27
	55	102	67	0	72	3
RIMS2	60	2	52	0	15	13
	0	2	52	0	21	0
RIMS3	40	6	14	7	8	6
	6	1	10	1	8	0
RIMS4	25	6	15	20	6	14
	7	7	7	0	9	2
RIN1	2	12	5	2	4	15
	4	7	3	1	2	1
RIN2	85	355	68	265	195	205
	277	314	198	101	194	72
RIN3	15	11	10	5	7	8
	12	7	6	3	7	5
RING1	28	73	17	35	44	45
	60	79	34	35	30	14
RINL	8	13	11	12	5	11
	8	11	14	4	9	2
RINT1	55	263	51	197	132	205
	226	287	139	88	159	53
RIOK1	62	162	43	95	59	124
	122	140	75	54	74	22
RIOK2	69	215	63	230	134	209
	249	314	131	85	187	45
RIOK3	178	1024	160	710	397	706
	758	1048	445	255	597	174
RIPK1	36	157	48	116	80	104
	96	127	75	54	62	34
RIPK2	15	19	10	15	6	19
	19	16	20	6	14	3
RIPK3	9	5	5	4	3	6
	11	14	11	0	8	3
RIPK4	9	1	7	0	3	0
	0	0	6	0	0	0

RIPPLY1	6	0	3	1	2	1	1	5	4	0	1	0
RIPPLY2	2	0	0	2	0	0	0	1	0	0	0	0
RIT1	62	195	49	134	81	145	158	216	103	52	101	43
RIT2	10	0	6	0	5	0	0	0	4	0	2	0
RLBP1	12	0	12	0	4	1	1	0	4	0	3	0
RLF	128	435	119	358	296	351	409	490	212	148	314	108
RLIM	193	827	180	596	428	578	630	963	389	263	555	182
RLN1	7	2	2	0	1	0	0	0	1	0	1	1
RLN2	4	0	1	0	0	0	0	0	1	1	0	0
RLN3	2	0	0	0	0	0	0	0	0	0	2	0
RLTPR	18	1	10	0	1	0	1	1	10	0	8	0
RMI1	17	77	15	40	31	40	40	68	43	24	42	16
RMI2	6	0	4	0	3	7	5	5	3	0	11	0
RMND1	43	116	35	80	58	89	129	144	86	34	70	22
RMND5A	620	3236	596	2916	1716	2332	2489	3597	1388	878	1791	588
RMND5B	46	100	17	56	40	63	65	98	46	26	54	20
RNASE1	47	234	35	157	175	160	197	487	131	85	143	43
RNASE10	3	0	4	0	1	0	0	0	1	0	1	0
RNASE11	7	0	14	0	2	0	0	0	6	0	2	0
RNASE12	3	0	1	0	0	0	0	0	2	0	0	0
RNASE13	12	2	2	0	1	2	1	0	8	0	5	0
RNASE2	3	0	3	1	0	0	2	0	2	0	1	0
RNASE3	4	0	0	0	1	0	0	0	0	0	2	0
RNASE4	26	64	19	73	44	69	49	124	36	16	45	22
RNASE6	4	4	3	2	4	4	7	11	7	2	4	1
RNASE7	7	1	7	0	2	2	5	4	1	0	1	0
RNASE8	2	0	0	0	0	0	0	0	0	0	0	0
RNASE9	1	0	4	0	2	2	0	0	1	0	2	0
RNASEH1	40	168	36	116	66	119	124	174	86	56	114	38

RNASEH2A	14	15	9	12	10	15
	17	34	12	3	12	2
RNASEH2B	36	80	34	45	33	55
	127	152	77	34	92	24
RNASEH2C	15	47	16	51	35	39
	78	52	35	20	20	5
RNASEK	17	88	8	56	35	60
	121	100	43	27	46	14
RNASEL	33	85	33	59	58	45
	51	78	64	24	47	12
RNASET2	17	55	14	38	23	41
	43	74	18	13	33	14
RND1	12	0	2	0	1	0
	0	0	11	0	3	0
RND2	20	3	11	11	8	12
	5	40	15	0	24	2
RND3	39	110	43	163	40	150
	107	85	103	47	60	18
RNF10	249	1817	228	1306	594	1149
	1342	1293	554	395	786	239
RNF103	11	122	12	123	34	117
	220	214	34	77	60	34
RNF103-CHMP3		232	500	214	516	430
	392	348	502	476	237	546
	148					
RNF11	219	1536	282	1462	658	1165
	1151	1572	660	321	791	241
RNF111	116	260	78	240	150	212
	231	294	140	88	142	61
RNF112	30	21	27	17	12	20
	2	25	8	5	11	2
RNF113A	7	35	14	34	21	30
	58	45	20	16	29	6
RNF113B	3	0	1	0	3	1
	0	0	5	0	4	0
RNF114	122	530	74	385	273	364
	397	658	283	170	298	88
RNF115	216	801	190	525	435	661
	661	1349	453	369	584	217
RNF121	65	198	50	151	75	171
	148	176	107	42	105	35
RNF122	22	94	29	77	34	62
	61	47	28	19	22	4
RNF123	176	943	127	666	370	580
	574	723	291	217	441	110
RNF125	44	100	26	69	28	50
	59	113	57	31	45	15
RNF126	17	83	9	34	18	60
	59	47	25	22	29	7
RNF128	53	172	12	121	119	141
	140	191	76	61	114	24
RNF13	117	545	121	393	324	507
	456	699	363	162	359	109
RNF130	77	325	47	231	141	320
	321	378	217	107	186	62
RNF135	29	61	16	67	19	52
	50	42	43	8	30	2

RNF138	31	135	26	98	52	78
	95	117	47	30	73	24
RNF139	82	320	62	316	167	250
	327	405	217	103	215	63
RNF14	182	623	117	488	290	437
	606	782	277	171	388	114
RNF141	179	915	163	675	374	555
	621	993	413	241	458	145
RNF144A	106	211	51	226	125	188
	205	193	162	56	126	48
RNF144B	406	1826	255	1323	1026	1627
	1895	2606	1286	467	1227	291
RNF145	100	229	73	197	113	168
	218	324	181	70	173	45
RNF146	221	945	193	751	428	677
	901	1424	517	308	767	195
RNF149	54	183	54	154	94	112
	154	272	116	71	123	48
RNF150	106	264	78	152	226	230
	327	535	264	143	261	123
RNF151	2	0	3	0	1	0
	0	0	0	0	1	0
RNF152	9	14	4	8	7	9
	18	9	10	5	13	3
RNF157	112	631	94	392	339	479
	485	618	271	188	260	100
RNF165	34	14	26	10	15	13
	9	25	24	9	18	3
RNF166	26	62	13	45	22	45
	59	86	34	18	49	3
RNF167	34	166	20	92	61	124
	118	126	71	42	70	16
RNF168	115	453	74	421	247	403
	359	410	176	150	232	70
RNF169	191	768	232	532	412	610
	572	888	342	266	516	137
RNF17	46	0	25	0	6	1
	0	0	33	0	22	0
RNF170	90	303	80	238	142	208
	227	299	154	84	158	49
RNF175	19	0	4	2	6	1
	0	0	3	0	6	0
RNF180	81	171	51	185	102	172
	145	136	125	48	125	28
RNF181	38	147	23	110	57	102
	129	259	80	41	79	14
RNF182	21	11	19	3	7	4
	4	5	8	0	5	0
RNF183	1	0	2	0	0	0
	0	0	2	0	0	0
RNF185	37	130	36	113	83	97
	132	187	63	36	84	30
RNF186	2	0	0	0	1	0
	0	0	0	0	0	0
RNF187	86	372	66	217	116	347
	259	325	128	90	163	41
RNF19A	182	908	247	631	466	625
	708	1017	402	320	507	180

RNF19B	33	100	34	87	44	59
	66	85	58	29	63	21
RNF2	48	248	42	178	96	154
	172	196	125	66	110	31
RNF20	116	422	108	321	209	319
	284	429	188	170	259	99
RNF207	28	35	10	33	24	8
	19	41	15	14	7	8
RNF208	1	0	2	1	0	0
	3	0	2	2	1	0
RNF212	18	2	7	4	12	1
	3	6	11	3	8	3
RNF213	517	1444	507	1264	894	1035
	1045	1805	968	619	985	313
RNF214	47	203	59	126	101	141
	129	168	92	63	87	49
RNF215	9	18	7	6	4	12
	8	19	7	5	12	4
RNF216	119	554	115	389	277	373
	399	583	268	167	307	95
RNF217	29	166	44	105	66	89
	59	112	47	31	44	14
RNF219	42	134	28	102	89	83
	113	168	90	42	107	27
RNF220	54	211	34	112	99	132
	153	189	90	65	92	32
RNF222	8	2	4	2	2	0
	0	0	2	0	1	0
RNF224	7	0	0	0	0	0
	0	0	2	0	1	0
RNF24	25	17	14	18	11	6
	5	17	12	2	11	1
RNF25	34	85	21	41	34	62
	33	82	49	28	39	18
RNF26	16	16	6	16	13	4
	9	20	7	2	9	2
RNF31	61	174	49	138	105	140
	161	260	106	86	164	55
RNF32	7	7	9	1	5	5
	0	0	8	5	7	0
RNF34	112	727	86	369	355	445
	506	703	371	168	457	114
RNF38	140	320	83	241	182	224
	233	413	163	110	158	56
RNF39	4	0	4	1	1	0
	0	0	3	0	1	0
RNF4	50	156	40	101	63	102
	132	168	95	61	103	38
RNF40	68	237	63	162	126	142
	187	257	117	89	150	62
RNF41	96	266	59	245	140	266
	219	236	121	103	158	55
RNF43	31	8	18	7	4	5
	20	10	34	1	15	0
RNF44	16	53	11	31	27	33
	57	63	22	9	33	8
RNF5	48	140	23	108	47	109
	141	172	93	29	105	17

RNF6	113	546	98	430	291	472
	405	606	292	158	324	139
RNF7	75	406	51	320	151	301
	424	546	220	99	224	91
RNF8	44	122	33	81	86	96
	107	142	76	45	81	21
RNFT1	47	110	36	101	65	117
	99	117	78	25	76	22
RNFT2	22	0	7	0	5	0
	0	1	11	1	7	0
RNGTT	60	236	68	142	110	170
	152	254	117	56	141	28
RNH1	43	254	47	177	103	167
	139	285	146	72	129	48
RNLS	32	85	33	64	46	54
	71	55	46	21	30	11
RNMT	94	399	105	288	210	247
	320	491	186	154	248	94
RNMTL1	31	112	21	85	53	94
	117	134	59	38	83	18
RNPC3	118	543	132	406	256	508
	519	607	297	235	571	107
RNPEP	33	94	31	65	53	62
	113	101	58	39	56	23
RNPEPL1	17	82	11	52	27	42
	74	63	31	5	27	13
RNPS1	111	437	80	308	226	362
	424	439	256	173	298	86
ROBO1	96	353	110	341	181	289
	383	169	180	78	127	46
ROBO2	96	7	59	15	36	3
	2	5	47	1	31	1
ROBO3	23	13	21	17	11	25
	9	9	25	2	15	5
ROBO4	35	106	9	44	33	49
	92	118	47	29	58	10
ROCK1	233	1112	271	772	456	943
	767	938	417	373	547	192
ROCK2	466	3098	673	2464	2010	2353
	2097	2890	1099	1045	1268	583
ROGDI	43	192	37	105	65	141
	208	167	62	61	114	33
ROM1	4	2	1	5	4	3
	1	3	6	1	4	0
ROMO1	27	124	20	124	43	76
	210	255	97	25	91	15
ROPN1	11	1	9	0	4	0
	0	0	8	0	6	0
ROPN1B	18	2	9	0	4	1
	3	6	8	2	4	0
ROPN1L	7	0	4	0	1	1
	3	0	3	0	0	0
ROR1	53	31	33	53	31	41
	12	49	36	24	38	8
ROR2	28	1	15	2	3	3
	0	5	13	1	6	1
RORA	532	1897	739	2117	1395	1933
	2274	3064	1344	1025	1657	585

RORB	23	2	21	4	5	0
	5	4	7	0	3	2
RORC	130	373	108	417	176	461
	650	538	329	273	375	137
ROS1	68	1	59	2	18	9
	4	1	77	1	26	0
RP1	41	0	25	1	6	0
	0	4	18	0	8	0
RP1L1	31	2	12	5	3	0
	2	1	12	2	5	0
RP2	16	40	10	28	28	39
	39	50	17	8	18	11
RP9	20	110	16	67	31	64
	109	123	49	43	54	15
RPA1	89	512	106	406	216	312
	388	371	184	106	202	75
RPA2	79	311	101	250	146	253
	309	449	172	107	242	74
RPA3	9	41	12	38	21	27
	25	63	21	6	34	5
RPAIN	83	286	63	171	110	192
	288	365	168	83	178	64
RPAP1	50	169	32	72	78	94
	136	127	67	46	81	27
RPAP2	97	447	116	280	208	300
	326	476	211	144	254	61
RPAP3	96	437	89	320	238	330
	300	388	186	126	267	68
RPE	57	240	65	196	126	161
	187	298	137	69	156	36
RPE65	13	0	5	0	8	0
	0	0	16	0	2	0
RPF1	56	245	46	175	105	156
	318	372	141	84	168	54
RPF2	52	327	69	134	148	264
	238	413	124	103	162	37
RPGR	49	78	21	40	38	42
	55	134	55	35	70	20
RPGRIP1	32	2	27	7	8	2
	3	4	31	1	8	3
RPGRIP1L	26	40	35	40	35	33
	39	39	38	24	50	12
RPH3A	35	0	24	0	11	0
	0	0	19	0	8	0
RPH3AL	15	58	9	32	12	18
	49	39	22	7	13	10
RPIA	40	110	19	72	43	75
	89	103	22	26	65	25
RPL10	519	1928	434	1927	961	2071
	2578	4569	1649	668	1452	407
RPL10A	289	1674	373	1347	690	1737
	2324	3424	1220	692	1306	378
RPL10L	1	0	2	0	2	0
	0	0	2	0	1	0
RPL11	294	1544	312	1288	635	1620
	2291	3114	1153	547	1185	332
RPL12	590	3544	988	2316	1230	4438
	5904	6483	2137	1153	2670	605

RPL13	360	1831	347	1743	852	2061
	2525	3516	1653	646	1356	453
RPL13A	945	5149	1015	4005	1979	4654
	7055	8460	2986	1689	3327	899
RPL14	473	2169	365	1539	932	1635
	2645	4200	1446	779	1615	505
RPL15	377	2309	435	1803	1005	1929
	2839	3726	1556	762	1442	475
RPL17	461	2485	440	1782	988	2598
	3217	5081	1695	960	1873	508
RPL17-C18ORF32		68	363	58	241	121
	257	377	539	190	100	220
	45					
RPL18	106	513	116	605	213	530
	685	930	434	184	387	123
RPL18A	92	622	137	638	243	789
	1116	1511	641	272	575	179
RPL19	986	5208	1049	4041	1717	4311
	6307	8407	3239	1648	2845	880
RPL21	376	2051	348	2434	906	2148
	2658	5186	1775	829	1559	535
RPL22	447	2737	508	2659	1156	2909
	3720	6070	1955	1074	1973	653
RPL22L1	18	23	10	34	14	37
	33	45	37	5	26	2
RPL23	414	2341	494	2172	829	2214
	3585	5141	1870	673	1633	424
RPL23A	542	3157	662	1877	1368	2976
	4045	5657	1527	1060	2085	502
RPL24	713	3367	860	3065	1560	3087
	5004	8141	2382	1368	2413	715
RPL26	654	3637	653	2341	1430	3166
	6262	8132	2261	1232	2496	756
RPL26L1	26	73	14	62	27	55
	145	158	55	30	62	13
RPL27	814	4025	1197	4290	1929	5652
	7848	9961	3946	1458	3548	657
RPL27A	351	1678	355	1572	850	1861
	3220	3061	1269	671	1316	295
RPL28	31	186	38	128	62	163
	229	154	96	64	113	45
RPL29	238	1723	202	1389	633	1661
	2873	3634	1370	784	1411	459
RPL3	109	668	196	608	183	395
	523	780	405	138	322	116
RPL30	684	2968	616	2614	1272	3139
	5165	7244	1976	1237	2344	524
RPL31	650	3684	810	3321	1562	3917
	4810	9514	3020	1108	2604	673
RPL32	795	4121	689	2783	1750	3047
	5707	8279	2275	1184	2670	619
RPL34	468	2560	500	2336	1045	1870
	3336	6109	2040	832	1807	535
RPL35	400	1848	324	1411	707	1500
	3327	3998	1266	722	1218	347
RPL35A	262	1121	270	1162	547	1135
	1827	3281	967	388	958	207

RPL36	106	758	118	397	311	458
	989	1398	384	219	382	114
RPL36A	1	38	0	36	0	12
	41	61	12	5	8	8
RPL36A-HNRNPH2		157	242	106	97	214
	94	30	72	182	76	233
	26					
RPL36AL	143	690	142	672	342	601
	1088	1351	504	242	491	152
RPL37	635	3386	689	2296	1367	3035
	5295	7114	2404	1270	2500	558
RPL37A	904	3955	821	2906	1747	3452
	5617	9035	2493	1440	2809	576
RPL38	866	3954	840	3075	2006	3643
	8177	10374	3038	1808	3311	777
RPL39	318	1500	357	1247	728	1620
	3337	4880	1428	697	1635	340
RPL39L	10	0	2	1	6	3
	3	2	3	1	2	2
RPL3L	639	3133	594	2548	1317	2923
	3439	4370	2151	1351	2481	549
RPL4	1302	6952	1584	4612	2873	7240
	6765	9679	4742	2317	4783	1205
RPL41	645	2854	548	2876	1150	3639
	4363	6769	2049	842	2202	442
RPL5	677	3370	739	2831	1344	3348
	4233	4822	2299	1242	2218	754
RPL6	821	4286	829	2881	1711	4341
	4929	5908	2753	1544	2677	859
RPL7	1008	6005	1573	5056	2675	7265
	7331	10647	4142	2140	4065	1082
RPL7A	380	2115	473	1823	833	2406
	2645	3457	1706	731	1416	384
RPL7L1	48	210	46	146	119	124
	141	247	85	81	129	34
RPL8	196	1097	166	849	424	784
	1857	1488	781	368	808	189
RPL9	70	715	365	767	225	3579
	4310	7422	773	216	639	169
RPLP0	922	3933	1298	5013	1848	6206
	5846	7669	3739	1742	3437	1050
RPLP1	733	4324	772	3189	1871	5289
	7399	5686	3043	1527	3068	859
RPLP2	430	3311	500	1979	1001	2840
	4548	4338	1792	910	1757	581
RPN1	113	506	100	396	237	396
	355	534	242	134	271	95
RPN2	142	559	116	489	235	519
	400	682	320	191	286	108
RPP14	140	918	143	657	420	531
	817	849	306	183	417	136
RPP21	1	7	2	6	5	5
	5	12	3	0	10	0
RPP25	3	5	3	5	1	0
	2	5	5	0	5	0
RPP25L	47	147	24	147	65	109
	219	320	97	42	116	31

RPP30	97	264	74	241	126	218	195	391	173	89	214	64
RPP38	9	37	2	24	17	8	26	34	18	9	17	9
RPP40	12	32	11	28	15	23	38	38	26	11	13	1
RPRD1A	185	1027	198	838	396	802	816	821	486	256	485	150
RPRD1B	82	424	94	232	204	265	349	483	191	103	253	63
RPRD2	148	655	160	446	333	460	606	748	240	224	335	148
RPRM	5	0	5	0	0	0	0	0	0	0	2	0
RPRML	0	3	1	0	2	2	2	0	0	0	0	0
RPS10	196	1032	284	1265	476	1480	2209	2975	1032	397	957	201
RPS10-NUDT3	220	895	233	780	443	871	1071	1444	525	181	563	111
RPS11	906	3859	1038	3702	1874	4320	6566	9903	3382	1790	3051	664
RPS12	434	2150	452	2059	827	2421	3055	5076	1702	907	1545	407
RPS13	390	1499	316	1764	813	1744	2357	4611	1812	717	1583	374
RPS14	307	1077	247	1075	494	1155	1658	3172	1213	400	1021	214
RPS15	212	1156	203	817	569	1210	1290	1781	638	585	772	307
RPS15A	950	4212	1097	5808	2476	5452	10147	10849	4488	2057	3951	939
RPS16	46	223	43	167	121	226	674	229	179	119	226	49
RPS17	287	1379	237	1123	513	1524	1900	3365	1107	393	967	220
RPS17L	284	1275	258	1127	554	1616	1990	3310	1112	423	975	202
RPS18	442	2091	342	2231	740	2006	3456	5379	1989	740	1347	390
RPS19	201	1022	276	952	415	993	1793	2529	818	437	823	225
RPS19BP1	41	156	23	120	80	120	164	220	67	50	83	28
RPS2	230	1420	246	1361	545	1456	2362	1871	952	373	908	262
RPS20	852	3903	761	4427	1496	3572	6032	7403	3484	1142	2863	592
RPS21	134	522	99	593	244	550	1069	1242	548	215	458	89
RPS23	678	3232	904	3841	1395	3353	4987	7394	2920	1190	2730	792
RPS24	925	4392	918	3343	1990	3590	6460	9981	2793	1658	2693	800
RPS25	1048	5157	945	4568	2116	5161	7612	11873	3886	1822	3837	1074

RPS26	72	356	30	396	115	211
	496	723	224	135	253	32
RPS27	421	1890	565	2767	1097	3336
	3559	6032	2662	1126	2189	651
RPS27A	545	2632	623	2459	1175	3617
	3898	6063	1903	1004	2029	461
RPS27L	149	583	125	458	332	517
	523	1187	331	161	318	95
RPS28	19	341	57	167	68	318
	21	540	203	8	272	39
RPS29	90	221	66	272	116	257
	308	651	220	76	198	46
RPS3	267	1203	180	1001	503	1358
	2051	1585	970	408	851	221
RPS3A	1471	8369	1657	6592	3764	10011
	11210	17942	5931	3212	6485	1538
RPS4X	818	4509	1271	3864	1968	5251
	6197	7305	3717	2004	3724	830
RPS4Y1	1	816	182	837	1	731
	1111	1680	674	8	635	159
RPS4Y2	0	0	5	0	0	0
	0	0	2	0	4	0
RPS5	334	1214	351	1019	612	1171
	2104	3041	1176	525	1048	298
RPS6	840	4416	906	3400	1638	4858
	5733	8504	3109	1463	2977	715
RPS6KA1	21	13	18	9	4	9
	7	2	9	0	10	3
RPS6KA2	116	393	100	225	180	183
	331	416	242	87	297	84
RPS6KA3	586	4214	752	2835	2013	3011
	2759	3697	1447	992	1611	537
RPS6KA4	16	55	13	37	32	34
	37	55	24	17	32	11
RPS6KA5	67	119	71	146	105	142
	133	123	76	49	98	49
RPS6KA6	38	20	22	12	17	16
	8	24	33	5	15	8
RPS6KB1	152	677	161	563	391	555
	527	888	386	277	492	156
RPS6KB2	50	157	39	92	39	102
	140	163	74	36	84	27
RPS6KC1	114	359	109	293	165	277
	248	372	177	120	218	68
RPS6KL1	52	57	27	61	33	55
	36	93	51	11	48	13
RPS7	287	1412	249	1324	585	1422
	2392	3302	1179	524	1135	285
RPS8	595	3576	578	2451	1279	2600
	4991	6035	2100	929	1959	542
RPS9	185	1005	191	1005	422	943
	1329	1824	749	313	756	160
RPSA	456	1986	486	2253	920	1948
	3158	4148	2171	928	2217	568
RPTN	22	9	27	0	7	0
	0	0	10	0	3	0
RPTOR	105	353	71	239	155	192
	262	312	138	105	175	79

RPUSD1	10	9	4	7	7	7
	8	10	7	4	6	6
RPUSD2	20	29	7	14	18	14
	31	42	23	9	28	2
RPUSD3	20	59	11	41	37	41
	45	72	42	20	49	14
RPUSD4	218	1101	167	897	573	891
	820	1137	484	359	628	135
RQCD1	28	138	20	87	59	95
	124	192	71	52	96	25
RRAD	130	541	163	454	302	181
	224	219	94	104	145	114
RRAGA	50	344	47	246	154	194
	344	308	136	100	166	54
RRAGB	57	169	45	125	67	117
	138	238	103	46	137	41
RRAGC	26	60	21	66	30	60
	63	64	45	18	44	11
RRAGD	517	2918	500	2493	1075	2217
	1830	2160	1127	548	1374	317
RRAS	10	26	4	12	22	21
	20	26	11	10	14	5
RRAS2	78	355	67	201	183	300
	354	575	245	119	200	63
RRBP1	57	235	64	202	121	174
	112	209	98	78	81	46
RREB1	128	729	150	529	313	562
	445	491	225	126	244	107
RRH	17	11	8	5	6	6
	8	6	3	6	16	1
RRM1	144	617	147	357	341	464
	567	678	296	218	373	106
RRM2	16	0	9	1	1	1
	1	1	17	1	9	0
RRM2B	258	946	246	1070	488	904
	888	955	582	282	650	151
RRN3	167	803	210	641	338	840
	624	790	383	186	464	99
RRNAD1	20	47	12	28	27	37
	41	69	26	12	20	7
RRP1	26	74	27	49	36	56
	57	79	33	39	39	13
RRP12	54	93	42	37	73	67
	71	155	60	46	55	50
RRP15	161	765	149	499	321	553
	643	826	303	245	458	116
RRP1B	70	170	59	145	86	145
	146	227	80	71	107	43
RRP36	56	272	46	255	140	241
	256	335	167	81	156	64
RRP7A	49	172	64	89	78	142
	104	287	129	91	76	50
RRP8	21	80	31	45	42	60
	82	107	31	42	42	10
RRP9	12	23	8	18	20	28
	36	76	15	12	29	6
RRS1	16	70	15	22	40	41
	42	61	22	24	36	6

RS1	18	1	6	0	1	0
	0	0	3	0	1	0
RSAD1	56	303	66	245	134	194
	282	182	98	69	115	29
RSAD2	33	128	34	110	56	80
	88	136	83	134	104	18
RSBN1	97	368	105	305	201	362
	370	481	215	128	241	75
RSBN1L	47	295	70	194	142	196
	204	303	122	89	160	58
RSC1A1	38	149	42	173	86	102
	146	211	102	42	128	56
RSF1	144	596	188	389	288	433
	376	482	205	207	301	129
RSG1	6	1	2	3	1	0
	1	2	3	0	1	0
RSL1D1	216	1115	219	603	518	806
	791	976	465	484	480	244
RSL24D1	156	555	128	452	301	480
	594	1014	364	241	444	114
RSPH1	19	2	6	7	4	0
	2	0	5	0	2	0
RSPH10B	41	12	18	2	13	4
	4	1	19	1	14	3
RSPH10B2	18	1	12	2	6	10
	0	7	10	1	9	1
RSPH3	22	25	12	17	21	12
	24	39	15	15	26	9
RSPH4A	25	17	20	12	13	26
	7	10	22	5	24	3
RSPH6A	11	0	6	0	1	0
	0	0	3	0	0	0
RSPH9	3	1	2	0	1	1
	1	0	2	0	0	0
RSPO1	37	4	12	1	10	1
	2	0	20	1	10	0
RSPO2	16	0	4	0	3	0
	0	0	5	0	4	0
RSPO3	106	557	37	185	254	166
	325	469	68	106	191	83
RSPO4	10	0	5	0	1	0
	0	0	6	0	2	0
RSPRY1	45	95	42	66	49	73
	105	91	42	21	66	21
RSRC1	29	149	39	103	88	125
	112	198	87	76	98	38
RSRC2	141	945	148	520	446	687
	679	839	289	296	469	147
RSU1	106	419	94	236	168	231
	342	416	169	102	176	76
RTBDN	16	0	3	0	2	0
	0	1	4	0	3	0
RTCA	93	423	79	266	193	299
	403	521	228	143	224	72
RTDR1	5	0	3	0	0	0
	0	0	1	0	2	0
RTEL1	39	67	30	54	37	79
	60	110	51	31	59	19

RTF1	145	770	132	560	315	520
	606	591	229	195	383	120
RTKN	23	59	13	35	25	40
	34	42	46	18	33	10
RTKN2	37	4	16	3	15	1
	1	4	14	0	12	0
RTL1	14	1	5	1	0	0
	2	1	5	0	5	0
RTN1	25	15	19	14	10	22
	21	15	11	4	13	6
RTN2	77	403	57	276	193	301
	420	285	151	106	244	47
RTN3	142	699	111	505	327	545
	399	494	229	153	225	145
RTN4	2057	13529	2235	10014	6072	10019
	11202	15618	5951	3010	8275	1721
RTN4IP1	59	201	39	114	80	132
	131	233	112	58	123	17
RTN4R	2	0	2	1	2	1
	0	0	1	0	0	0
RTN4RL1	9	26	10	19	18	31
	5	8	8	3	6	2
RTN4RL2	3	1	2	0	0	0
	1	3	1	0	1	0
RTP1	9	0	4	0	0	0
	0	0	4	0	2	0
RTP2	4	1	1	0	2	0
	3	0	1	0	1	1
RTP3	4	0	1	0	0	0
	0	0	1	0	2	0
RTP4	1	7	3	5	3	2
	4	2	2	3	2	0
RTTN	189	466	218	399	220	386
	346	618	315	197	374	98
RUFY1	101	454	80	234	233	325
	254	513	178	147	196	70
RUFY2	101	355	101	248	179	271
	272	453	160	129	217	59
RUFY3	178	971	257	646	446	582
	640	765	421	250	473	141
RUFY4	16	2	9	0	5	0
	0	0	13	0	9	0
RUNDC1	31	100	22	65	50	61
	71	70	35	30	40	14
RUNDC3A	9	2	9	0	0	0
	0	0	3	0	2	0
RUNDC3B	27	19	22	4	17	29
	8	26	21	4	25	4
RUNX1	34	142	86	103	25	95
	31	40	50	10	18	16
RUNX1T1	106	375	100	346	188	229
	284	323	164	94	189	66
RUNX2	32	18	32	27	17	26
	12	32	32	12	24	2
RUNX3	10	5	10	5	4	2
	0	6	9	2	5	2
RUSC1	21	36	17	24	16	21
	17	34	23	9	16	5

RUSC1-AS1	8	3	7	5	5	3
	2	2	0	0	0	2
RUSC2	109	299	76	249	164	256
	255	335	156	98	280	59
RUVBL1	27	165	31	66	69	91
	88	213	67	48	74	27
RUVBL2	41	234	39	160	92	153
	179	312	137	64	136	43
RWDD1	150	886	104	373	306	720
	759	990	361	212	407	118
RWDD2A	11	40	18	24	15	15
	37	48	24	12	27	6
RWDD2B	77	455	84	365	176	392
	447	454	238	123	258	66
RWDD3	27	160	24	111	63	95
	126	187	66	44	52	18
RWDD4	145	649	152	566	298	754
	818	817	347	253	514	113
RXFP1	28	1	18	0	3	0
	2	0	16	0	3	0
RXFP2	18	0	13	0	5	0
	0	0	18	0	9	0
RXFP3	6	0	3	0	0	0
	0	0	3	1	1	0
RXFP4	0	1	1	0	0	0
	0	0	1	0	0	0
RXRA	132	695	91	539	279	358
	752	556	233	250	394	115
RXRB	56	161	39	86	105	112
	139	206	93	54	123	36
RXRG	86	220	49	252	159	361
	227	239	162	51	91	37
RYBP	29	117	36	109	46	89
	93	108	55	40	83	19
RYK	100	496	128	408	230	433
	417	561	234	151	283	77
RYR1	3788	27801	3805	19782	8876	16455
	17802	19488	8196	7502	12111	5207
RYR2	172	22	106	20	58	15
	4	28	105	6	74	9
RYR3	775	4492	384	388	1137	931
	1930	3839	349	1212	2092	372
S100A1	357	2287	95	788	708	953
	2421	1371	406	479	932	75
S100A10	18	139	22	145	77	99
	109	174	72	38	46	30
S100A11	4	11	7	19	8	19
	14	12	10	3	10	5
S100A12	2	2	4	1	4	0
	1	1	1	0	1	0
S100A13	64	283	42	178	103	224
	427	263	128	62	141	25
S100A14	9	1	5	0	3	1
	0	0	3	0	1	0
S100A16	8	32	12	49	18	43
	34	27	29	4	12	2
S100A2	7	1	5	1	2	4
	0	1	2	0	2	0

S100A3	4	1	5	0	2	0
	0	1	1	0	4	0
S100A4	30	189	32	224	55	218
	54	204	88	33	64	37
S100A5	3	0	1	0	1	1
	0	0	4	0	2	0
S100A6	92	487	137	751	145	498
	433	620	320	145	242	95
S100A7	2	1	2	0	3	0
	0	0	3	0	0	0
S100A7A	37	0	34	0	3	0
	0	0	13	0	9	0
S100A7L2	2	0	1	0	1	0
	0	0	0	0	0	0
S100A8	3	8	1	2	1	7
	23	14	3	4	7	0
S100A9	10	26	6	8	2	14
	23	40	2	4	6	0
S100B	9	10	4	12	7	2
	12	23	1	4	0	14
S100G	3	0	0	0	1	0
	0	0	1	0	1	0
S100P	3	4	1	1	1	2
	1	1	0	0	0	1
S100PBP	64	145	76	164	110	138
	127	213	76	48	106	30
S100Z	11	0	2	0	1	0
	0	0	7	0	0	0
S1PR1	32	202	36	98	82	80
	222	231	110	64	141	47
S1PR2	12	10	9	6	6	4
	3	10	8	1	2	3
S1PR3	22	32	8	23	17	19
	27	34	24	6	14	2
S1PR4	2	2	1	0	0	1
	0	3	0	1	2	0
S1PR5	8	2	2	0	3	0
	0	1	2	0	2	0
SAA1	6	33	31	32	3	47
	5	5	5	1	1	3
SAA2	4	5	9	10	1	21
	1	3	10	1	1	2
SAA2-SAA4	9	2	4	1	1	1
	0	0	2	0	2	0
SAA4	0	0	1	1	1	4
	0	0	0	0	1	1
SAAL1	31	82	33	52	47	60
	72	133	57	30	68	21
SAC3D1	6	19	6	19	16	11
	16	19	13	6	9	5
SACM1L	65	175	61	181	113	153
	185	284	128	52	138	52
SACS	1015	3613	1288	2685	2851	3151
	2086	3399	1333	1147	1439	680
SAE1	48	265	73	203	110	240
	247	274	158	76	156	48
SAFB	106	514	99	309	227	265
	404	402	131	156	257	97

SAFB2	92	448	104	247	245	352
	411	527	170	184	297	94
SAG	12	0	20	0	4	0
	0	0	10	0	4	0
SAGE1	34	0	10	0	5	0
	0	0	4	0	0	0
SALL1	27	1	15	0	4	0
	0	1	12	0	4	0
SALL2	22	13	15	11	13	19
	9	3	15	5	6	1
SALL3	11	0	4	0	1	0
	0	0	6	0	0	0
SALL4	6	2	4	0	6	0
	0	0	6	0	1	1
SAMD1	6	20	2	9	20	15
	16	23	4	3	5	5
SAMD10	4	5	1	0	2	0
	4	1	0	0	0	0
SAMD11	5	5	5	2	6	1
	3	2	8	0	0	1
SAMD12	51	49	44	13	12	25
	22	35	55	7	30	8
SAMD13	29	18	10	29	20	17
	29	112	15	14	33	15
SAMD14	12	15	6	3	5	1
	2	0	6	2	10	0
SAMD15	15	1	10	1	5	2
	0	2	8	1	4	0
SAMD3	23	11	14	1	5	3
	6	4	15	0	14	1
SAMD4A	505	2068	340	1996	1179	2001
	1610	2090	997	681	1372	429
SAMD4B	32	119	52	79	68	66
	125	120	70	45	75	21
SAMD5	39	18	20	18	12	8
	7	19	29	1	11	5
SAMD7	24	0	8	1	2	0
	1	4	10	0	8	0
SAMD8	289	1365	329	1370	755	1248
	1095	1285	552	361	803	209
SAMD9	50	165	51	109	67	90
	86	135	71	87	82	22
SAMD9L	130	348	80	274	170	185
	205	305	201	176	272	66
SAMHD1	56	278	59	158	134	155
	168	359	108	123	167	67
SAMM50	161	1004	147	938	341	789
	1139	847	495	238	447	112
SAMSN1	30	5	19	7	5	11
	3	8	15	4	7	1
SAP130	66	149	40	79	77	73
	94	106	61	48	79	29
SAP18	260	1513	256	778	621	1115
	1433	1987	693	460	893	258
SAP25	5	7	1	0	2	3
	0	2	2	0	1	1
SAP30	9	28	5	24	24	21
	12	59	16	7	9	7

SAP30BP	46	228	49	161	114	175	199	269	122	93	109	37
SAP30L	85	231	50	241	121	155	186	252	131	69	146	43
SAPCD1	4	3	3	2	3	2	0	2	7	0	5	0
SAPCD2	14	2	8	1	5	0	0	0	4	0	4	0
SAR1A	200	995	208	827	483	745	913	1200	554	322	593	223
SAR1B	745	4776	841	4204	2270	3680	3927	5213	2279	1278	2826	765
SARDH	9	2	15	4	1	0	2	9	3	1	6	0
SARM1	31	34	28	22	15	22	26	27	19	15	16	9
SARNP	85	442	68	260	150	294	364	581	210	87	247	56
SARS	129	637	132	461	284	531	527	813	316	218	398	142
SARS2	22	36	13	30	24	27	33	49	29	19	13	8
SART1	57	250	39	117	95	163	141	204	84	73	113	46
SART3	127	506	141	314	232	410	377	501	215	153	317	109
SASH1	138	571	106	428	269	319	512	659	320	209	321	139
SASH3	11	5	2	2	4	7	3	17	4	4	4	1
SASS6	37	72	29	62	44	46	53	91	36	21	36	9
SAT1	53	173	56	137	88	173	188	304	96	59	113	47
SAT2	36	153	23	122	61	121	180	259	113	58	102	30
SATB1	306	1563	256	904	812	1008	1184	1628	703	516	929	300
SATB2	52	54	43	109	46	34	50	49	27	16	48	9
SATL1	12	0	6	2	4	2	0	2	3	0	1	0
SAV1	49	141	61	146	84	150	152	196	93	62	104	30
SAYSD1	20	74	38	67	52	117	119	139	72	20	51	10
SBDS	406	2162	331	1467	807	1712	2251	2927	1355	815	1468	433
SBF1	93	324	92	215	131	188	208	389	164	128	190	87
SBF2	156	466	178	355	247	412	403	582	344	163	333	129
SBK1	67	262	65	106	87	168	151	146	69	46	102	19
SBK2	0	0	0	0	0	0	0	0	1	0	1	0
SBNO1	256	1221	268	819	527	855	725	1271	440	354	654	208

SBNO2	17	47	20	40	19	45
	21	54	24	28	26	10
SBSN	7	0	9	0	3	0
	0	0	4	0	3	0
SBSPON	23	9	11	38	3	3
	4	3	12	5	14	13
SC5DL	91	281	57	206	98	189
	205	191	114	47	121	42
SCAF1	26	135	23	60	46	65
	69	89	37	33	52	26
SCAF11	327	1657	375	1268	783	1036
	1191	1592	634	573	874	354
SCAF4	61	219	68	135	114	143
	220	167	65	74	118	45
SCAF8	263	1311	384	923	594	939
	974	1275	573	446	767	242
SCAI	109	301	136	264	181	232
	196	403	209	102	195	65
SCAMP1	118	505	100	491	265	379
	385	699	282	149	328	104
SCAMP2	12	46	11	53	35	50
	52	56	35	22	36	8
SCAMP3	49	162	26	128	61	147
	142	174	70	45	91	39
SCAMP4	10	21	3	15	8	15
	26	20	9	10	11	6
SCAMP5	27	1	12	3	2	4
	2	0	6	0	8	0
SCAND1	11	36	7	37	16	19
	25	53	19	7	12	2
SCAND3	33	11	14	4	8	2
	0	2	25	2	16	0
SCAP	44	116	39	74	45	84
	77	133	64	39	79	30
SCAPER	168	562	184	405	259	380
	423	510	272	227	295	100
SCARA3	28	41	13	66	8	16
	18	28	25	8	19	17
SCARA5	66	271	75	353	202	261
	118	559	123	59	201	89
SCARB1	23	44	14	28	28	51
	38	54	26	13	26	12
SCARB2	528	2102	591	1792	1164	1505
	1840	2925	1145	604	1433	545
SCARF1	29	62	14	29	29	36
	36	80	36	19	35	12
SCARF2	8	3	2	3	2	2
	4	4	4	0	2	3
SCCPDH	107	451	84	493	230	340
	490	601	242	160	287	92
SCD	60	211	33	109	195	120
	84	205	30	37	52	675
SCD5	30	16	14	36	10	15
	13	20	21	3	8	7
SCEL	48	0	29	0	7	0
	0	0	20	0	12	0
SCFD1	123	548	138	426	226	311
	448	635	263	170	385	109

SCFD2	50	167	51	93	69	106
	91	131	71	48	99	26
SCG2	10	2	10	3	2	10
	1	1	9	1	2	2
SCG3	30	4	21	2	2	6
	1	1	20	1	12	3
SCG5	15	1	8	0	1	1
	0	2	8	0	2	0
SCGB1A1	6	0	1	0	0	0
	0	0	5	0	1	0
SCGB1C1	1	0	1	0	1	0
	0	0	1	0	1	0
SCGB1D1	2	0	0	0	0	0
	0	0	1	0	0	0
SCGB1D2	3	2	2	1	1	2
	3	17	5	3	12	2
SCGB1D4	5	0	4	0	1	0
	0	0	1	0	0	0
SCGB2A1	5	0	0	0	0	0
	0	0	4	0	0	0
SCGB2A2	1	0	0	0	1	0
	0	0	1	0	0	0
SCGB2B2	4	1	2	2	0	0
	1	2	2	0	1	0
SCGB3A1	1	3	0	0	1	0
	0	0	0	0	0	0
SCGB3A2	4	0	1	0	1	0
	0	0	1	0	1	0
SCGN	12	0	9	0	3	0
	0	0	14	0	5	0
SCHIP1	1	180	30	58	55	55
	148	650	18	125	61	46
SCIMP	8	1	3	0	3	1
	2	1	3	2	0	0
SCIN	39	10	16	10	8	0
	8	6	15	0	11	0
SCLT1	33	96	34	48	40	48
	87	112	68	30	81	16
SCLY	10	27	7	14	10	27
	21	45	10	5	21	7
SCMH1	68	297	94	216	150	259
	303	384	168	116	223	48
SCML1	66	298	82	268	117	242
	268	517	151	103	224	50
SCML2	26	32	6	24	24	32
	25	14	21	7	15	0
SCML4	38	5	22	3	9	5
	1	8	12	1	11	2
SCN10A	41	0	30	0	13	0
	0	0	27	0	17	1
SCN11A	50	5	26	2	17	0
	2	6	28	1	12	1
SCN1A	65	0	47	1	13	1
	2	5	57	2	61	0
SCN1B	172	629	113	627	378	571
	614	813	321	237	418	152
SCN2A	69	1	46	6	24	0
	2	3	49	0	22	3

SCN2B	56	89	29	26	50	68
	61	194	18	43	57	15
SCN3A	72	15	47	25	32	15
	19	59	46	9	57	11
SCN3B	56	127	53	66	35	99
	62	99	59	34	46	24
SCN4A	426	1831	400	1667	852	1800
	1683	1763	903	541	1058	335
SCN4B	46	170	33	198	87	139
	163	167	55	42	138	21
SCN5A	49	13	47	15	8	12
	0	1	12	8	17	0
SCN7A	175	493	81	609	260	373
	788	960	521	241	464	101
SCN8A	99	12	45	9	19	7
	9	1	69	5	25	0
SCN9A	69	64	47	54	84	83
	36	99	88	23	70	15
SCNM1	6	100	23	100	53	85
	156	191	27	51	56	24
SCNN1A	27	3	13	5	2	5
	0	2	13	0	3	0
SCNN1B	9	1	5	0	0	2
	1	0	3	0	3	0
SCNN1D	6	10	10	8	3	2
	7	11	13	3	10	3
SCNN1G	14	0	14	0	4	0
	4	0	7	0	5	0
SCO1	60	238	54	152	89	206
	172	325	107	89	157	44
SCO2	3	6	3	5	6	1
	7	10	7	2	3	0
SCOC	86	460	101	307	193	366
	391	594	226	110	235	84
SCP2	254	1187	267	925	529	939
	1267	1267	613	338	814	216
SCPEP1	69	237	34	325	79	186
	87	116	73	36	63	33
SCRG1	5	0	2	1	5	0
	0	0	1	1	4	4
SCRIB	58	187	41	110	64	112
	102	138	57	43	73	40
SCRN1	31	57	21	55	45	62
	41	55	34	15	29	26
SCRN2	27	78	36	70	48	61
	98	112	59	12	51	12
SCRN3	110	568	96	332	229	367
	409	422	191	109	232	83
SCRT1	4	0	5	0	0	0
	0	0	2	0	0	0
SCRT2	11	0	8	0	3	0
	0	0	3	0	6	0
SCT	3	2	1	5	0	10
	10	0	15	1	5	0
SCTR	13	0	7	0	3	1
	0	2	5	0	2	0
SCUBE1	24	2	7	14	1	0
	2	3	3	1	5	1

SCUBE2	42	52	47	95	32	68
	43	56	46	8	55	5
SCUBE3	38	19	34	25	10	3
	9	12	26	3	11	5
SCXA	1	7	0	0	2	0
	0	2	1	1	1	0
SCXB	1	0	0	0	1	1
	0	0	2	0	1	0
SCYL1	78	333	66	217	147	267
	284	345	165	133	154	82
SCYL2	91	522	85	275	210	304
	337	383	200	123	209	74
SCYL3	72	159	63	143	104	97
	137	222	116	68	125	25
SDAD1	90	399	71	226	174	251
	294	350	170	135	230	74
SDC1	15	4	4	2	4	1
	1	3	1	0	9	1
SDC2	95	369	139	313	294	397
	219	589	186	118	177	99
SDC3	34	74	26	53	29	44
	63	59	40	21	31	17
SDC4	110	244	67	171	122	209
	374	409	111	118	208	66
SDCBP	64	320	62	239	156	187
	248	371	137	76	144	77
SDCBP2	1	0	0	0	0	0
	0	0	0	1	0	0
SDCCAG3	43	119	36	65	56	132
	164	190	84	45	76	19
SDCCAG8	112	472	135	281	200	312
	326	455	204	165	241	68
SDE2	72	261	67	195	174	193
	227	344	147	97	164	60
SDF2	29	78	15	74	35	77
	73	118	76	31	58	16
SDF2L1	4	6	1	4	3	8
	8	14	6	0	4	2
SDF4	52	279	49	184	112	194
	227	299	130	98	159	50
SDHA	527	2919	434	2012	1206	1309
	2992	3088	1144	798	1835	432
SDHAF1	10	20	11	32	11	20
	34	33	21	7	28	11
SDHAF2	31	150	27	83	74	106
	107	155	69	40	67	23
SDHB	282	1570	205	1300	581	961
	2058	2367	1010	370	1059	245
SDHC	54	480	68	299	161	158
	352	267	72	75	196	66
SDHD	230	960	166	986	467	729
	1163	1901	837	331	765	208
SDK1	67	49	44	29	40	27
	27	30	48	8	32	14
SDK2	48	5	29	4	10	4
	0	2	20	1	11	2
SDPR	73	380	32	217	188	135
	369	564	199	164	272	83

SDR16C5	21	0	8	1	5	2
	1	4	7	0	4	0
SDR39U1	16	64	15	46	19	35
	56	54	32	15	29	8
SDR42E1	16	0	5	1	2	0
	1	0	6	1	2	0
SDR9C7	18	3	9	7	9	2
	0	9	11	2	5	3
SDS	7	0	4	0	2	0
	0	0	2	0	3	0
SDSL	7	2	6	1	1	1
	0	0	4	0	1	1
SEBOX	1	1	1	0	0	0
	0	0	1	0	0	0
SEC11A	116	662	111	430	263	438
	699	909	321	163	267	124
SEC11C	18	54	16	42	16	31
	43	74	39	15	33	12
SEC13	66	394	63	303	186	284
	360	422	231	145	227	79
SEC14L1	175	691	132	515	326	496
	763	600	364	231	413	148
SEC14L2	20	3	13	3	9	5
	2	12	16	2	6	1
SEC14L3	14	0	2	0	1	0
	0	0	11	0	3	0
SEC14L4	22	0	11	0	2	0
	0	0	9	0	3	0
SEC14L5	49	112	50	68	75	65
	118	48	58	18	43	29
SEC14L6	17	0	6	1	1	0
	0	0	2	0	2	0
SEC16A	95	366	85	249	165	210
	280	296	142	101	202	82
SEC16B	96	238	54	251	103	170
	191	309	97	42	152	38
SEC22A	41	106	53	117	58	97
	109	173	86	43	87	33
SEC22C	70	211	88	169	107	161
	194	303	137	73	124	71
SEC23A	245	1192	235	998	641	852
	991	1244	601	321	753	217
SEC23B	33	104	33	74	50	61
	55	97	81	23	52	14
SEC23IP	150	464	122	323	246	340
	411	466	257	145	293	82
SEC24A	100	263	57	258	121	227
	217	272	146	88	179	66
SEC24B	164	803	180	518	352	601
	706	702	334	248	449	170
SEC24C	120	452	98	346	220	361
	312	556	261	123	272	69
SEC24D	73	113	45	117	41	90
	74	160	77	27	81	32
SEC31A	218	1056	228	842	478	667
	763	1028	492	319	576	215
SEC31B	62	131	40	65	71	156
	169	317	107	69	141	68

SEC61A1	116	357	82	362	177	290
	255	437	166	94	172	79
SEC61A2	35	49	15	42	30	54
	42	76	35	23	38	9
SEC61B	11	48	12	40	29	40
	67	58	34	14	30	14
SEC61G	58	289	55	315	107	158
	368	562	228	76	185	47
SEC62	157	811	155	567	441	606
	582	789	360	247	452	151
SEC63	92	442	110	386	226	308
	344	504	199	135	216	121
SECISBP2	143	570	125	428	323	501
	385	756	285	201	332	126
SECISBP2L	200	828	203	710	470	622
	672	1004	372	290	595	218
SECTM1	9	5	4	2	6	2
	4	4	1	2	2	0
SEH1L	45	196	43	114	95	100
	208	216	74	62	115	36
SEL1L	257	983	236	950	503	929
	842	1224	600	285	660	193
SEL1L2	18	5	12	0	3	2
	2	5	8	2	5	1
SEL1L3	34	70	40	56	44	40
	67	73	61	26	48	8
SELE	33	0	11	2	4	0
	0	1	20	0	8	0
SELENBP1	63	209	48	153	171	114
	142	235	79	72	121	67
SELK	95	420	112	406	171	391
	445	866	346	134	295	72
SELL	22	27	13	11	6	10
	12	14	12	6	4	2
SELM	3	7	6	13	9	8
	17	26	9	6	6	3
SELO	17	38	9	39	21	48
	50	72	35	21	21	12
SELP	48	21	13	17	10	3
	7	25	16	5	16	4
SELPLG	11	19	5	6	11	18
	7	39	18	7	11	3
SELRC1	24	146	25	112	80	88
	145	144	65	49	67	23
SELT	125	469	139	494	231	368
	467	830	296	139	363	117
SELV	6	0	5	0	0	0
	0	0	2	0	1	0
SEMA3A	39	13	36	6	31	8
	8	8	25	3	10	4
SEMA3B	13	19	7	21	18	16
	29	42	28	10	25	9
SEMA3C	293	1104	162	568	810	867
	530	1123	403	341	520	188
SEMA3D	51	8	28	25	17	4
	9	7	20	7	15	6
SEMA3E	59	4	35	9	18	3
	3	15	25	2	14	3

SEMA3F	27	51	26	16	25	19
	29	28	25	10	13	3
SEMA3G	49	163	26	56	55	49
	129	170	60	37	86	45
SEMA4A	21	14	14	11	11	5
	24	22	17	3	15	3
SEMA4B	18	7	16	3	8	9
	22	39	19	11	20	2
SEMA4C	24	62	20	35	33	43
	27	38	19	13	31	12
SEMA4D	91	498	123	395	205	346
	253	280	148	89	129	71
SEMA4F	41	9	33	16	7	20
	12	14	22	4	18	2
SEMA4G	20	8	13	6	9	4
	2	2	11	1	7	0
SEMA5A	107	248	66	206	136	191
	156	212	187	71	149	54
SEMA5B	31	15	12	6	8	3
	2	18	11	3	7	3
SEMA6A	78	216	50	167	105	111
	144	107	103	36	95	31
SEMA6B	10	16	5	7	10	3
	8	20	14	3	7	5
SEMA6C	186	1248	212	669	457	784
	879	1077	457	301	550	175
SEMA6D	174	1029	291	758	402	568
	572	539	431	145	379	65
SEMA7A	22	5	11	9	9	2
	6	12	7	2	4	0
SEMG1	13	0	16	0	6	0
	0	0	5	0	4	0
SEMG2	18	0	17	0	9	0
	0	0	11	0	5	0
SENP1	56	119	40	109	68	84
	125	205	94	52	100	32
SENP2	77	433	80	328	176	235
	308	335	175	82	233	59
SENP3	38	77	26	42	44	62
	77	124	60	41	54	21
SENP5	120	436	113	399	215	388
	344	546	244	141	332	97
SENP6	324	1380	368	1088	681	1164
	1110	1632	780	477	895	297
SENP7	149	475	159	374	214	395
	357	718	294	179	390	113
SENP8	29	57	21	46	22	46
	57	81	32	15	39	13
sep-15	56	283	62	269	115	195
	243	402	161	68	134	41
SEPHS1	99	284	84	268	149	232
	226	304	182	107	191	62
SEPHS2	16	155	7	110	52	73
	108	69	29	23	29	14
SEPN1	36	118	24	80	48	61
	93	77	48	32	50	24
SEPP1	390	1286	265	1134	816	1102
	1282	2761	1394	537	1236	423

SEPSECS	63	220	56	164	92	157
	163	326	174	74	203	40
sep-01	13	8	5	4	4	2
	7	5	8	2	8	0
sep-10	92	467	92	315	158	330
	312	407	183	123	220	72
sep-11	108	614	149	420	222	400
	363	513	288	115	268	84
sep-12	9	0	5	0	0	0
	0	0	3	0	1	0
sep-14	24	0	17	0	7	3
	0	0	10	0	5	0
sep-02	302	1425	266	1393	873	1262
	1116	1697	685	445	767	378
sep-03	15	0	15	0	4	0
	1	2	8	1	6	0
sep-04	23	54	22	18	24	21
	34	62	36	13	25	8
sep-05	18	30	21	33	20	23
	18	15	15	8	13	3
sep-06	46	82	26	60	44	49
	38	59	38	20	36	15
sep-07	264	1277	229	909	752	1196
	851	1109	455	403	591	202
sep-08	83	178	38	114	90	116
	88	161	81	46	69	24
sep-09	94	346	85	224	141	227
	278	282	137	126	138	70
SEPW1	226	976	72	887	528	1058
	1420	1608	728	263	546	142
SERAC1	41	171	39	87	87	108
	109	112	76	42	83	27
SERBP1	629	3705	610	2380	1953	2793
	2781	3685	1502	1264	2184	708
SERF1A	38	101	19	30	23	113
	74	88	33	29	59	2
SERF1B	45	164	25	52	99	129
	133	144	67	66	93	21
SERF2	130	749	85	329	248	349
	775	887	286	120	265	74
SERGEF	38	62	26	42	37	60
	63	91	43	25	47	13
SERHL2	19	11	14	5	12	2
	7	16	13	4	4	1
SERINC1	520	2744	537	2610	1387	2508
	2093	3287	1415	832	1698	550
SERINC2	20	19	23	40	17	26
	62	42	34	9	23	11
SERINC3	136	688	129	554	318	485
	519	853	347	199	429	140
SERINC4	21	12	15	11	14	20
	9	24	12	3	19	1
SERINC5	138	206	123	105	108	88
	119	112	94	29	71	23
SERP1	37	159	34	101	79	102
	118	174	88	37	75	35
SERP2	3	4	5	0	2	1
	6	8	4	0	3	0

SERPINA1	25	9	14	3	9	7
	7	8	7	1	9	1
SERPINA10	12	0	14	0	3	0
	0	0	3	0	3	0
SERPINA11	13	0	6	0	2	0
	0	0	5	0	0	0
SERPINA12	8	1	9	0	3	0
	0	0	4	0	3	0
SERPINA3	17	6	13	26	4	5
	0	14	10	2	3	3
SERPINA4	9	0	10	0	1	0
	0	0	2	0	2	0
SERPINA5	71	105	58	779	85	294
	14	22	91	72	13	5
SERPINA6	7	0	3	0	2	0
	0	0	5	0	0	0
SERPINA7	10	0	5	0	5	0
	0	0	3	1	3	0
SERPINA9	10	0	15	0	1	0
	0	0	8	0	1	0
SERPINB1	48	200	37	123	129	140
	181	245	89	109	119	43
SERPINB10	13	0	13	0	4	0
	0	0	4	0	2	0
SERPINB11	15	0	11	0	5	0
	0	0	15	0	4	0
SERPINB12	16	0	8	0	8	1
	0	0	5	0	2	0
SERPINB13	15	0	9	0	2	0
	0	0	21	0	8	0
SERPINB2	12	0	8	0	2	0
	0	0	9	0	3	0
SERPINB3	12	0	5	0	2	0
	0	0	8	0	9	0
SERPINB4	16	0	10	0	8	0
	1	0	4	0	4	0
SERPINB5	17	0	12	0	4	1
	1	0	6	0	6	0
SERPINB6	133	687	93	391	225	543
	891	829	368	227	501	126
SERPINB7	14	0	10	0	5	1
	0	0	10	0	5	0
SERPINB8	40	17	19	21	14	24
	5	15	26	4	12	1
SERPINB9	30	49	21	37	31	26
	30	56	26	22	29	8
SERPINC1	12	2	10	0	4	0
	1	0	10	1	6	0
SERPIND1	14	1	11	1	4	0
	1	4	4	0	4	0
SERPINE1	28	5	8	4	6	14
	15	15	19	3	9	2
SERPINE2	31	33	23	53	20	21
	48	33	30	9	22	4
SERPINE3	6	0	4	0	2	0
	1	1	7	1	0	0
SERPINF1	330	1830	251	1087	859	1341
	1335	1540	698	562	644	320

SERPINF2	4	2	6	1	1	2
	0	5	4	0	3	2
SERPING1	74	270	59	287	147	161
	156	308	102	81	136	57
SERPINH1	25	104	23	81	68	61
	55	67	39	20	31	18
SERPINI1	22	41	22	16	14	17
	21	31	24	12	24	6
SERPINI2	15	2	4	11	4	2
	1	5	22	0	12	0
SERTAD1	9	13	6	15	10	9
	22	11	3	7	11	1
SERTAD2	72	253	54	194	146	128
	184	189	79	56	94	28
SERTAD3	19	59	9	43	29	37
	68	58	24	16	43	8
SERTAD4	15	19	8	7	10	11
	14	11	18	4	6	8
SERTM1	16	0	16	0	8	0
	0	0	5	0	7	0
SESN1	474	2094	389	1360	1313	1388
	3234	6617	2108	1122	3096	754
SESN2	12	33	17	21	19	24
	18	27	10	9	17	7
SESN3	42	224	69	164	61	87
	77	107	52	22	41	26
SESTD1	149	362	125	338	192	277
	243	387	197	112	200	99
SET	323	1757	375	1175	823	1360
	1509	2123	787	672	997	343
SETBP1	247	1054	222	876	513	944
	1098	1036	582	430	855	231
SETD1A	35	136	36	68	60	64
	88	77	38	21	53	18
SETD1B	38	134	37	60	48	56
	87	93	29	19	32	10
SETD2	286	1653	304	989	750	983
	1143	1377	563	499	820	293
SETD3	436	2156	420	1790	907	1685
	1749	2036	898	607	1213	299
SETD4	30	85	23	60	34	60
	59	93	62	20	55	16
SETD5	186	928	219	618	400	518
	716	745	331	269	449	171
SETD6	39	133	32	78	68	97
	115	151	57	51	95	20
SETD7	638	4396	535	2886	2065	2881
	3882	2634	1288	1152	1964	649
SETD8	67	278	68	173	132	168
	189	203	78	63	121	38
SETD9	23	52	19	43	26	15
	61	68	34	13	28	11
SETDB1	91	287	66	191	128	184
	208	312	151	103	160	60
SETDB2	66	127	48	118	62	124
	126	111	85	36	95	18
SETMAR	303	193	140	163	149	120
	152	188	175	66	156	23

SETX	316	1344	340	965	862	1045
	910	1710	612	574	871	401
SEZ6	27	0	15	0	2	0
	0	0	9	0	4	0
SEZ6L	37	2	24	2	2	2
	1	2	12	0	15	0
SEZ6L2	17	2	7	0	5	0
	3	0	13	1	11	0
SF1	130	666	124	462	254	397
	627	564	202	171	330	99
SF3A1	83	303	58	204	117	181
	202	215	94	82	151	47
SF3A2	6	63	15	35	19	22
	47	47	21	16	27	10
SF3A3	115	691	101	498	284	442
	373	663	359	220	547	153
SF3B1	697	3704	853	2787	1749	2738
	2654	3873	1645	1202	2290	706
SF3B14	61	208	31	136	102	137
	216	402	151	70	139	52
SF3B2	81	489	106	276	238	337
	293	442	182	148	233	78
SF3B3	186	668	228	439	321	529
	570	940	344	251	490	150
SF3B4	17	29	16	21	23	8
	24	33	18	7	15	12
SF3B5	23	103	15	65	44	77
	126	159	45	30	47	12
SFI1	43	42	24	26	19	27
	22	41	32	17	21	13
SFMBT1	67	255	90	217	125	257
	186	262	164	74	177	51
SFMBT2	99	244	72	142	93	148
	114	232	137	54	133	15
SFN	0	2	1	1	0	0
	0	0	1	0	1	0
SFPQ	279	1454	378	933	619	1145
	1076	1944	804	599	1119	359
SFR1	15	42	21	36	17	34
	28	75	34	11	34	7
SFRP1	21	33	22	41	58	31
	23	45	12	11	21	32
SFRP2	13	7	22	31	2	20
	0	10	5	1	5	19
SFRP4	91	100	41	438	92	148
	100	52	43	38	63	47
SFRP5	6	1	11	21	0	0
	10	0	3	3	4	3
SFSWAP	76	340	96	269	163	280
	281	393	221	163	248	99
SFT2D1	25	57	29	55	31	70
	70	159	55	21	54	15
SFT2D2	22	35	13	48	10	25
	21	55	32	8	29	9
SFT2D3	21	63	17	48	31	39
	39	86	52	10	43	4
SFTA2	2	1	0	0	2	0
	0	0	3	0	0	0

SFTA3	8	0	3	0	3	0
	0	0	0	0	2	0
SFTPA1	18	0	7	0	1	0
	0	0	7	0	2	0
SFTPA2	8	2	8	1	4	0
	0	0	2	0	2	0
SFTPB	31	0	14	1	5	0
	0	0	7	0	5	1
SFTPC	6	0	2	0	1	0
	0	0	2	0	1	0
SFTPD	8	0	10	4	2	0
	2	4	4	0	4	0
SFXN1	38	153	38	94	85	124
	142	203	98	38	103	30
SFXN2	24	30	11	8	8	11
	18	35	6	7	19	6
SFXN3	36	42	25	28	21	34
	26	48	35	12	23	16
SFXN4	58	280	47	200	82	258
	252	368	156	75	204	42
SFXN5	33	125	34	93	56	114
	85	121	69	34	74	17
SGCA	175	750	119	654	315	649
	1008	1335	567	339	688	154
SGCB	449	2454	592	2465	1238	2054
	2418	2073	896	641	1173	330
SGCD	595	3446	850	2979	1466	3328
	3050	3228	1266	865	1716	601
SGCE	30	93	21	66	62	71
	61	138	46	40	50	22
SGCG	257	874	169	631	485	745
	971	1660	723	349	755	204
SGCZ	17	1	10	1	5	1
	0	1	7	0	5	0
SGIP1	51	70	28	45	47	29
	56	87	53	25	54	10
SGK1	161	1440	207	1462	482	1072
	651	370	775	224	283	65
SGK110	2	0	2	0	1	0
	0	1	1	0	1	0
SGK196	30	73	34	55	34	64
	55	94	55	14	61	9
SGK2	14	7	9	3	1	5
	1	1	3	0	6	0
SGK223	22	68	8	41	19	65
	49	47	43	18	39	19
SGK3	0	0	1	3	0	1
	1	2	1	0	2	1
SGK494	65	112	32	61	43	70
	57	150	74	35	60	13
SGMS1	76	292	104	239	173	231
	240	369	202	88	138	61
SGMS2	77	195	71	180	93	119
	91	120	99	37	66	21
SGOL1	21	1	12	0	6	2
	3	1	12	0	13	0
SGOL2	32	18	30	8	12	15
	14	18	33	4	18	3

SGPL1	80	171	48	147	98	126
	197	250	143	56	174	32
SGPP1	19	90	16	69	33	67
	67	111	31	20	45	15
SGPP2	5	0	2	0	3	0
	0	0	6	0	4	0
SGSH	14	23	7	22	13	10
	9	28	9	3	11	8
SGSM1	62	6	27	5	12	12
	5	7	36	0	18	0
SGSM2	59	164	75	199	71	128
	98	161	97	49	95	47
SGSM3	73	203	59	139	125	184
	136	221	107	52	115	42
SGTA	40	143	48	96	62	112
	167	163	93	48	100	44
SGTB	53	110	51	106	84	85
	82	97	52	40	85	22
SH2B1	71	242	46	127	89	164
	186	213	84	69	103	41
SH2B2	9	17	1	15	14	33
	16	23	18	13	18	4
SH2B3	60	211	48	124	84	128
	205	194	102	71	96	31
SH2D1A	12	8	6	6	11	4
	1	14	4	0	6	1
SH2D1B	20	55	13	91	29	150
	85	59	94	26	133	7
SH2D2A	8	2	4	2	1	0
	4	0	3	0	4	0
SH2D3A	15	1	8	0	4	0
	1	0	9	0	3	0
SH2D3C	20	80	14	24	27	16
	44	34	27	16	24	11
SH2D4A	34	15	9	14	9	15
	3	4	12	5	12	3
SH2D4B	23	0	14	2	6	0
	1	0	5	1	10	0
SH2D5	20	0	8	0	2	1
	0	0	6	0	1	0
SH2D6	3	0	4	0	1	0
	0	0	0	0	0	0
SH2D7	11	0	8	0	2	0
	1	0	2	0	2	0
SH3BGR	809	3462	395	1938	1959	2204
	4521	4100	2208	1075	2315	558
SH3BGRL	27	105	27	91	60	70
	106	153	68	48	68	40
SH3BGRL2	42	113	53	135	100	90
	101	238	93	42	67	29
SH3BGRL3	7	38	11	25	24	30
	31	55	20	21	35	7
SH3BP1	7	2	7	4	0	1
	3	1	9	1	4	1
SH3BP2	77	189	38	113	99	180
	129	160	134	69	104	30
SH3BP4	27	41	19	40	26	33
	28	34	29	17	25	16

SH3BP5	48	193	78	121	118	134
	98	219	180	52	140	51
SH3BP5L	47	215	61	150	92	175
	176	263	152	55	139	41
SH3D19	153	601	167	507	267	430
	478	664	290	198	297	154
SH3D21	9	14	9	5	9	7
	5	9	12	1	9	0
SH3GL1	19	34	5	20	11	17
	21	22	14	11	12	10
SH3GL2	25	1	7	1	8	0
	5	4	16	1	9	0
SH3GL3	14	1	5	0	3	0
	1	0	10	0	4	0
SH3GLB1	788	3621	778	2532	2462	3019
	3163	3863	1400	1014	1927	652
SH3GLB2	23	93	12	49	39	70
	59	97	47	33	46	10
SH3KBP1	50	165	27	71	75	77
	139	121	43	34	67	34
SH3PXD2A	145	526	172	279	212	264
	339	564	196	158	230	132
SH3PXD2B	42	82	41	80	42	64
	48	91	48	14	57	24
SH3RF1	54	170	45	125	109	102
	160	185	126	62	123	33
SH3RF2	53	303	91	331	136	292
	182	185	188	37	154	61
SH3RF3 (NC_000002 109745996..110107395)				14	28	10
	13	6	12	25	20	22
	5	10	4			
SH3RF3 (NC_000002 110259066..110262207)				25	62	16
	21	14	38	46	61	36
	13	28	6			
SH3TC1	11	14	8	12	3	10
	14	7	20	7	8	3
SH3TC2	138	82	110	55	54	51
	63	38	97	20	63	15
SH3YL1	41	147	50	186	58	131
	153	271	109	62	86	27
SHANK1	17	1	13	3	4	2
	2	0	5	0	6	0
SHANK2	57	5	28	7	8	0
	0	0	18	0	12	1
SHANK3	28	186	25	73	61	79
	137	148	74	63	80	30
SHARPIN	49	153	31	100	62	120
	142	197	100	58	100	23
SHB	16	48	6	29	9	25
	30	17	22	13	13	4
SHBG	17	1	5	1	0	0
	0	2	5	2	5	1
SHC1	43	151	44	99	74	85
	86	122	76	34	74	42
SHC2	10	17	7	12	3	9
	13	6	4	4	14	11
SHC3	45	29	33	36	15	29
	12	35	35	3	20	9

SHC4	30	9	21	2	8	2
	8	19	27	2	16	0
SHCBP1	44	40	24	24	20	40
	29	64	31	17	34	9
SHCBP1L	9	0	5	0	5	0
	0	0	7	0	2	0
SHD	6	0	6	0	0	0
	0	2	4	1	1	0
SHE	65	227	42	133	113	119
	153	231	105	70	118	48
SHF	12	19	4	10	6	10
	15	16	10	4	11	5
SHFM1	96	575	71	361	217	376
	675	657	253	101	303	57
SHH	3	0	2	0	1	0
	0	0	0	0	2	0
SHISA2	50	790	217	774	243	411
	195	228	237	30	117	19
SHISA3	6	3	4	7	0	2
	3	8	5	3	6	2
SHISA4	287	1136	179	929	475	959
	890	999	500	265	666	153
SHISA5	15	48	13	44	19	47
	39	74	40	24	42	10
SHISA6	39	11	24	10	15	12
	4	13	20	3	10	3
SHISA7	11	1	5	0	4	0
	0	0	6	0	4	0
SHISA8	2	0	0	0	0	0
	0	0	0	0	0	0
SHISA9	41	0	33	1	15	2
	3	3	39	1	26	2
SHKBP1	17	22	5	21	4	12
	13	27	13	8	12	5
SHMT1	37	133	26	88	60	109
	130	158	84	26	72	24
SHMT2	38	75	31	73	59	78
	122	159	69	23	52	16
SHOC2	124	773	162	711	355	676
	598	840	282	205	441	122
SHOX (NC_000023 585078..620146)			8	1	4	1
	2	0	0	2	8	2
	6	5				
SHOX (NC_000024 535078..570146)			6	1	5	1
	1	1	1	2	3	0
	1	3				
SHOX2	17	24	11	25	8	23
	17	27	17	2	19	5
SHPK	26	62	29	47	39	47
	56	116	43	30	54	21
SHPRH	218	827	275	599	362	655
	659	917	476	259	523	151
SHQ1	38	110	42	63	51	62
	77	117	40	23	52	16
SHROOM1	15	7	5	4	6	0
	9	12	15	1	8	1
SHROOM2	25	7	6	2	10	2
	3	8	12	1	10	1

SHROOM3	53	10	24	7	10	1
	2	7	30	1	18	8
SHROOM4	76	218	35	125	144	146
	181	267	157	113	163	64
SI	50	0	35	0	20	0
	0	0	46	0	36	0
SIAE	65	281	52	188	115	182
	231	296	154	85	160	33
SIAH1	38	213	43	211	107	199
	284	288	153	77	159	44
SIAH2	16	82	18	53	38	70
	69	76	38	24	44	7
SIAH3	14	1	5	0	2	0
	1	1	4	0	4	0
SIDT1	36	7	21	3	13	5
	13	9	26	0	9	2
SIDT2	34	113	36	89	52	70
	75	109	42	18	52	21
SIGIRR	6	5	2	2	2	9
	8	3	2	2	5	2
SIGLEC1	24	33	14	24	29	26
	11	62	34	11	27	14
SIGLEC10	16	10	11	1	5	2
	0	2	3	0	4	1
SIGLEC11	19	3	8	3	3	4
	2	3	7	1	7	1
SIGLEC12	17	0	14	0	1	0
	0	0	5	0	3	0
SIGLEC14	5	0	3	1	3	0
	1	0	3	0	1	0
SIGLEC15	5	1	4	0	1	0
	0	0	4	0	1	0
SIGLEC5	9	2	9	1	3	4
	1	3	11	0	4	0
SIGLEC6	8	0	8	5	2	1
	0	1	9	0	2	0
SIGLEC7	18	2	8	0	3	0
	1	1	6	1	1	0
SIGLEC8	17	1	16	3	3	5
	1	4	10	1	7	0
SIGLEC9	13	0	10	3	3	0
	0	1	9	1	8	0
SIGLECL1	16	0	5	0	4	0
	0	0	2	0	3	0
SIGMAR1	11	42	6	34	19	34
	48	35	17	11	25	12
SIK1	17	32	15	28	12	23
	7	15	18	5	14	5
SIK2	104	270	77	171	190	199
	233	347	118	221	163	65
SIK3	120	535	110	251	185	258
	273	312	151	130	179	54
SIKE1	130	522	143	398	279	411
	503	554	224	176	316	94
SIL1	49	154	31	101	75	129
	152	259	78	62	116	39
SIM1	65	157	48	109	138	154
	151	138	106	76	104	37

SIM2	31	124	31	66	72	88
	91	72	51	47	19	17
SIN3A	103	430	111	319	195	218
	281	316	123	104	168	72
SIN3B	46	162	44	106	68	127
	120	164	69	46	84	32
SIPA1	12	25	2	17	8	8
	20	29	15	8	13	8
SIPA1L1	67	121	55	124	63	91
	70	94	78	36	55	38
SIPA1L2	82	222	104	163	93	118
	136	123	100	57	101	32
SIPA1L3	48	100	41	68	28	60
	74	69	60	29	39	19
SIRPA	37	106	27	90	56	62
	60	82	41	39	53	10
SIRPB1	10	3	8	1	3	2
	0	1	7	0	3	1
SIRPB2	14	7	6	5	6	4
	1	15	9	1	5	1
SIRPD	3	0	2	1	3	0
	0	0	4	0	1	0
SIRPG	17	2	3	1	2	1
	0	0	6	0	1	1
SIRT1	96	379	82	306	183	251
	335	468	160	114	227	77
SIRT2	173	602	115	313	276	548
	609	982	320	307	418	167
SIRT3	29	118	27	48	51	58
	99	90	50	18	62	17
SIRT4	7	29	11	10	6	12
	33	46	13	4	8	3
SIRT5	130	567	104	299	222	286
	562	525	196	132	353	83
SIRT6	10	9	2	5	6	6
	12	18	3	1	7	6
SIRT7	6	10	5	7	8	9
	13	30	10	5	7	3
SIT1	7	0	3	0	0	0
	0	1	1	0	1	0
SIVA1	26	76	8	100	35	58
	71	164	60	12	45	10
SIX1	434	1766	283	1211	818	1064
	1626	2346	693	550	1037	290
SIX2	11	15	3	8	19	21
	7	53	8	17	10	1
SIX3	9	0	4	0	0	0
	0	0	3	0	1	0
SIX4	201	872	212	644	529	619
	924	1088	444	304	556	189
SIX5	10	17	13	13	11	18
	12	22	9	4	13	2
SIX6	6	5	2	3	3	1
	1	1	0	0	0	0
SKA1	17	1	9	1	4	0
	2	5	9	0	7	0
SKA2	47	153	44	137	75	127
	118	143	68	33	97	20

SKA3	17	5	18	7	3	2
	0	1	7	2	14	1
SKAP1	11	7	7	2	2	2
	1	0	9	1	7	1
SKAP2	60	244	38	130	130	200
	143	246	126	62	116	36
SKI	34	213	44	135	101	84
	127	100	48	35	62	44
SKIDA1	27	10	16	7	9	4
	6	1	26	1	10	0
SKIL	198	848	184	1209	675	1156
	1218	807	450	285	566	188
SKIV2L	59	127	50	82	57	91
	114	143	76	37	79	25
SKIV2L2	227	1136	298	800	565	841
	802	1292	519	361	748	237
SKOR1	15	1	5	3	4	6
	0	2	3	1	3	2
SKOR2	5	1	10	0	0	0
	0	0	3	1	2	0
SKP1	391	2201	368	1522	1047	1595
	2213	2321	849	574	1127	311
SKP2	37	88	30	47	56	55
	56	71	44	20	45	12
SLA	27	19	18	8	11	10
	18	28	29	5	21	5
SLA2	14	8	9	4	2	13
	6	5	9	1	4	3
SLAIN1	27	30	21	26	26	29
	23	49	35	12	36	3
SLAIN2	206	1088	192	726	477	661
	788	873	410	320	540	182
SLAMF1	24	4	8	1	1	0
	1	0	13	0	5	0
SLAMF6	20	7	12	2	3	4
	1	6	10	0	8	2
SLAMF7	18	12	8	4	7	1
	5	6	7	1	5	2
SLAMF8	14	6	16	2	3	5
	2	6	7	4	8	0
SLAMF9	8	0	3	1	2	0
	0	1	3	0	0	1
SLBP	38	82	18	75	43	57
	69	114	40	37	71	8
SLC10A1	17	1	9	2	4	0
	2	5	6	0	5	0
SLC10A2	22	0	16	0	11	0
	0	0	10	0	6	0
SLC10A3	9	21	4	22	16	25
	14	27	10	12	11	6
SLC10A4	14	2	7	2	4	0
	2	2	2	0	1	0
SLC10A5	9	4	2	1	6	8
	1	4	1	5	3	0
SLC10A6	11	11	9	12	6	4
	9	18	13	5	18	5
SLC10A7	59	67	36	74	50	67
	91	130	71	40	66	10

SLC11A1	15	13	9	2	4	1
	3	6	15	1	6	0
SLC11A2	138	320	87	237	173	291
	333	445	181	99	194	63
SLC12A1	53	2	24	0	12	0
	0	1	20	1	16	1
SLC12A2	339	2164	418	1317	766	974
	1469	2281	1435	355	1266	242
SLC12A3	26	0	16	0	7	1
	0	0	18	0	5	0
SLC12A4	43	162	51	96	85	106
	143	126	67	35	72	23
SLC12A5	37	0	28	1	9	1
	0	1	28	0	17	0
SLC12A6	133	414	114	329	191	219
	350	499	259	129	254	93
SLC12A7	32	48	8	43	32	38
	17	38	22	5	26	9
SLC12A8	18	3	8	2	3	0
	1	4	14	2	3	0
SLC12A9	15	24	23	10	6	21
	19	19	22	4	24	3
SLC13A1	34	0	22	1	12	5
	1	1	16	0	9	0
SLC13A2	13	0	8	0	4	0
	0	0	5	0	6	0
SLC13A3	43	19	35	36	14	35
	11	15	25	9	45	3
SLC13A4	21	3	9	2	6	6
	3	5	10	1	7	0
SLC13A5	20	1	5	0	5	0
	0	1	7	0	5	0
SLC14A1	53	63	17	36	34	40
	40	119	44	35	51	12
SLC14A2	43	2	23	3	10	9
	2	6	14	1	18	2
SLC15A1	29	2	13	0	4	0
	0	0	13	0	10	0
SLC15A2	69	42	42	21	19	34
	26	53	47	13	38	5
SLC15A3	11	6	4	11	4	6
	14	6	10	7	18	0
SLC15A4	80	234	48	273	139	313
	282	470	217	110	245	75
SLC15A5	26	0	7	0	1	0
	2	0	10	0	8	0
SLC16A1	401	2096	179	996	895	1103
	1547	2129	1046	428	1166	242
SLC16A10	135	712	267	791	314	622
	339	473	268	123	208	62
SLC16A11	3	0	4	1	1	0
	0	1	1	0	3	0
SLC16A12	31	36	13	33	38	8
	67	41	59	23	45	16
SLC16A13	3	5	3	0	2	0
	0	0	3	1	0	1
SLC16A14	27	33	14	16	19	15
	9	32	25	6	10	2

SLC16A2	26	36	17	29	31	40
	21	28	16	17	16	8
SLC16A3	41	290	21	148	92	339
	238	69	138	71	105	18
SLC16A4	47	42	14	29	22	53
	29	67	18	13	29	13
SLC16A5	4	26	5	21	11	15
	28	18	16	7	16	3
SLC16A6	30	15	24	7	10	11
	8	18	19	4	16	6
SLC16A7	131	231	89	250	220	171
	171	499	128	106	129	147
SLC16A8	2	0	1	0	0	1
	0	0	3	0	1	0
SLC16A9	24	41	28	16	16	19
	13	29	14	7	24	14
SLC17A1	14	0	8	1	7	0
	0	0	15	0	3	0
SLC17A2	13	1	12	1	7	0
	0	0	8	0	7	1
SLC17A3	19	0	13	0	2	0
	0	0	4	0	3	0
SLC17A4	26	0	16	0	12	0
	0	0	29	0	11	0
SLC17A5	25	72	21	57	40	72
	49	79	48	25	32	12
SLC17A6	26	0	20	0	11	0
	1	1	10	0	12	0
SLC17A7	27	3	12	3	4	1
	0	1	9	1	6	2
SLC17A8	26	0	16	0	4	0
	0	0	24	0	5	0
SLC17A9	6	2	5	2	3	1
	3	1	5	1	1	0
SLC18A1	21	0	16	0	9	0
	0	0	13	0	7	0
SLC18A2	10	13	16	35	9	14
	3	21	22	4	19	2
SLC18B1	46	65	31	100	51	59
	69	119	68	16	73	8
SLC19A1	3	8	4	8	1	9
	6	6	7	2	2	0
SLC19A2	216	655	107	453	336	572
	1822	2462	575	505	972	283
SLC19A3	26	21	15	4	26	15
	17	13	17	24	13	22
SLC1A1	25	20	9	10	17	3
	9	47	40	13	19	6
SLC1A2	61	21	49	45	29	17
	13	16	48	11	31	11
SLC1A3	105	258	69	115	219	130
	236	517	135	109	183	97
SLC1A4	77	545	85	445	291	801
	295	330	376	133	164	52
SLC1A5	13	21	16	8	15	11
	19	24	7	5	10	10
SLC1A6	16	0	7	0	4	0
	0	0	11	0	3	0

SLC1A7	17	22	5	9	27	22
	20	21	16	7	16	5
SLC20A1	74	198	45	117	98	179
	229	263	113	77	142	39
SLC20A2	475	1951	339	1045	1107	1274
	1632	2404	845	475	1056	329
SLC22A1	17	7	17	12	10	14
	12	15	24	6	20	5
SLC22A10	25	0	5	0	6	0
	0	0	13	0	9	0
SLC22A11	5	0	6	0	2	0
	0	0	4	0	1	0
SLC22A12	15	1	6	0	1	0
	0	0	0	0	0	0
SLC22A13	8	2	7	3	0	2
	2	3	4	2	1	0
SLC22A14	19	1	6	0	2	2
	1	0	6	1	5	0
SLC22A15	32	6	26	13	21	10
	8	21	23	1	23	4
SLC22A16	15	0	15	2	7	0
	0	0	13	0	2	0
SLC22A17	12	7	11	6	5	2
	6	5	6	2	5	1
SLC22A18	5	1	2	2	2	0
	0	0	4	0	2	0
SLC22A18AS	12	0	4	0	0	0
	0	0	2	0	0	0
SLC22A2	27	0	10	1	1	0
	1	2	14	1	6	0
SLC22A20	6	0	2	1	1	0
	0	1	2	1	2	0
SLC22A23	39	74	21	55	40	50
	90	117	32	16	53	28
SLC22A24	25	0	14	0	1	0
	0	0	11	0	8	0
SLC22A25	12	0	9	0	5	0
	0	0	6	0	1	0
SLC22A3	54	313	34	194	56	207
	242	282	340	190	122	84
SLC22A31	6	0	3	0	1	0
	0	0	2	0	1	0
SLC22A4	24	16	7	9	7	14
	17	21	21	8	16	5
SLC22A5	53	61	25	40	42	74
	113	162	68	32	59	29
SLC22A6	12	0	4	0	3	0
	0	0	5	0	1	0
SLC22A7	18	0	1	0	2	0
	0	0	9	1	2	0
SLC22A8	14	0	4	0	4	0
	0	0	6	0	5	0
SLC22A9	18	2	8	1	4	0
	0	0	8	0	4	0
SLC23A1	15	0	5	0	3	0
	0	0	7	0	4	0
SLC23A2	58	124	56	109	47	75
	86	82	70	30	67	26

SLC23A3	14	6	2	0	4	0
	1	4	8	0	1	0
SLC24A1	54	19	23	19	21	17
	26	38	28	4	27	8
SLC24A2	60	4	39	17	19	5
	3	1	26	0	28	0
SLC24A3	70	226	95	175	112	191
	149	203	106	119	169	63
SLC24A4	64	18	25	8	14	7
	6	7	33	0	13	1
SLC24A5	21	1	13	3	7	0
	0	1	8	0	6	0
SLC24A6	17	28	7	41	20	43
	31	32	26	9	15	5
SLC25A1	18	30	10	14	10	19
	16	23	11	9	6	5
SLC25A10	4	8	1	4	1	0
	3	9	2	1	6	2
SLC25A11	86	734	83	520	197	410
	677	872	358	112	443	75
SLC25A12	622	3123	485	2419	1381	2064
	2674	3163	1709	1085	2102	450
SLC25A13	52	168	66	134	81	158
	145	205	115	62	88	32
SLC25A14	19	22	10	25	14	32
	20	51	25	4	21	3
SLC25A15	71	84	38	49	35	47
	64	78	65	19	63	9
SLC25A16	48	175	47	161	87	149
	156	196	80	46	95	31
SLC25A17	23	48	17	40	31	46
	37	81	40	16	40	9
SLC25A18	13	1	9	2	7	7
	9	32	10	4	10	4
SLC25A19	15	26	9	16	7	24
	26	30	12	9	22	3
SLC25A2	11	5	5	3	1	7
	2	9	9	0	3	0
SLC25A20	98	338	62	215	130	197
	237	347	122	66	151	41
SLC25A21	25	10	11	9	13	16
	12	9	15	1	15	0
SLC25A22	9	4	7	2	4	2
	0	4	3	0	7	0
SLC25A23	46	194	26	106	61	103
	208	117	72	50	89	35
SLC25A24	57	288	78	263	124	233
	171	230	174	85	155	49
SLC25A25	65	257	33	185	182	305
	450	244	152	74	163	61
SLC25A26	(NC_000003	66119284..66119661)		1	0	0
	0	0	0	0	0	2
	0	1	0			
SLC25A26	(NC_000003	66271148..66429351)		64	251	67
	199	116	243	278	379	131
	58	136	26			
SLC25A27	81	241	88	211	131	177
	203	450	155	93	219	64

SLC25A28	23	94	12	70	51	75
	92	170	45	30	50	21
SLC25A29	9	15	7	11	10	13
	15	42	9	4	8	4
SLC25A3	815	5025	789	4377	1857	3566
	6101	6665	3384	1525	3467	806
SLC25A30	150	660	116	526	445	680
	1012	829	262	185	394	88
SLC25A31	7	0	9	0	1	0
	0	0	8	0	3	0
SLC25A32	48	193	42	108	71	124
	152	214	97	51	124	29
SLC25A33	15	57	19	44	43	35
	260	355	68	57	149	49
SLC25A34	156	489	155	303	287	427
	270	416	193	88	110	42
SLC25A35	13	5	10	10	6	2
	1	5	6	2	4	1
SLC25A36	182	629	171	735	360	531
	802	987	367	297	575	195
SLC25A37	64	334	41	186	143	166
	156	230	89	61	114	37
SLC25A38	64	206	39	162	110	183
	158	315	130	63	116	33
SLC25A39	14	81	14	44	26	49
	48	87	27	24	29	9
SLC25A4	1756	12418	1370	8319	3886	8051
	19030	17158	6281	3718	8166	1810
SLC25A40	43	109	19	72	66	55
	76	147	77	31	58	24
SLC25A41	3	1	2	1	2	1
	0	0	3	0	1	0
SLC25A42	57	209	46	168	117	183
	154	325	123	76	112	23
SLC25A43	25	28	5	19	18	29
	25	35	20	2	12	3
SLC25A44	30	114	18	88	38	88
	99	90	44	30	63	12
SLC25A45	10	27	16	27	20	14
	42	64	34	13	33	6
SLC25A46	189	866	144	840	406	664
	807	954	449	292	581	139
SLC25A47	5	0	1	0	0	0
	0	0	0	0	0	0
SLC25A48	3	1	1	0	4	0
	0	2	1	0	2	0
SLC25A5	24	116	24	94	39	65
	149	169	49	41	76	28
SLC25A51	30	83	23	60	32	63
	89	105	43	21	54	21
SLC25A52	2	1	3	0	1	0
	1	1	0	0	2	0
SLC25A53	46	5	13	4	11	5
	4	4	15	1	15	0
SLC25A6 (NC_000023 1505044..1511039)				9	50	9
	35	21	42	75	76	36
	22	40	21			

SLC25A6 (NC_000024 1455044..1461039)			7	96	8	
	48	34	58	74	130	33
	31	35	14			
SLC26A1	7	4	2	1	2	0
	0	2	3	0	1	1
SLC26A10	18	7	15	10	10	4
	5	5	12	1	8	3
SLC26A11	12	27	12	27	12	12
	10	21	13	5	11	3
SLC26A2	60	157	76	156	85	151
	144	243	128	58	129	31
SLC26A3	30	0	10	3	8	8
	6	2	15	0	15	0
SLC26A4	32	8	12	7	9	6
	7	9	19	4	18	1
SLC26A5	41	2	23	3	8	1
	0	1	21	0	10	0
SLC26A6	35	78	34	48	30	108
	44	90	52	25	47	13
SLC26A7	45	15	34	21	21	33
	19	26	35	6	35	0
SLC26A8	23	0	11	0	4	0
	0	0	14	0	11	0
SLC26A9	38	3	21	2	10	15
	9	2	24	2	8	9
SLC27A1	37	116	22	73	63	71
	117	225	74	59	102	19
SLC27A2	14	0	5	0	1	0
	0	0	8	0	1	0
SLC27A3	13	27	16	15	14	13
	17	24	20	3	13	4
SLC27A4	16	39	8	26	15	27
	19	33	22	9	20	7
SLC27A5	6	2	7	0	5	5
	2	8	8	0	7	0
SLC27A6	14	7	11	6	15	6
	4	20	23	8	16	1
SLC28A1	37	4	7	6	5	8
	4	3	6	0	5	1
SLC28A2	29	0	9	1	7	0
	2	4	17	0	10	0
SLC28A3	34	0	12	2	10	2
	0	0	17	0	10	0
SLC29A1	116	325	50	227	170	300
	414	415	172	149	262	102
SLC29A2	71	295	64	139	209	254
	299	377	153	104	166	57
SLC29A3	7	6	3	5	4	1
	0	8	5	0	8	1
SLC29A4	10	4	5	0	8	1
	0	16	3	2	4	3
SLC2A1	27	4	14	44	5	5
	8	2	5	1	11	10
SLC2A10	22	37	15	28	18	35
	19	33	17	8	18	6
SLC2A11	29	83	26	62	31	82
	71	93	53	24	47	12

SLC2A12	117	208	83	92	185	125
	302	431	171	56	196	61
SLC2A13	45	46	39	50	37	37
	45	79	54	14	40	15
SLC2A14	22	1	10	0	6	0
	0	1	18	0	0	0
SLC2A2	27	0	14	0	5	0
	0	0	8	0	6	0
SLC2A3	40	57	29	62	27	37
	61	62	36	18	30	15
SLC2A4	251	1592	183	789	458	878
	1852	2005	858	472	1001	279
SLC2A4RG	46	202	37	110	84	124
	148	136	67	46	108	38
SLC2A5	68	262	28	196	178	299
	356	377	240	145	228	98
SLC2A6	8	6	5	4	0	4
	3	11	7	2	2	0
SLC2A7	9	0	1	0	0	0
	0	0	5	0	1	0
SLC2A8	18	44	5	26	15	31
	28	76	35	17	22	8
SLC2A9	20	5	17	4	6	12
	11	11	10	5	2	2
SLC30A1	10	56	15	34	20	25
	39	54	23	17	18	11
SLC30A10	15	0	5	0	1	0
	0	0	3	0	2	1
SLC30A2	12	4	11	5	1	7
	0	5	10	1	2	0
SLC30A3	10	1	5	0	1	0
	0	0	4	0	2	0
SLC30A4	26	51	29	40	31	36
	36	44	28	20	29	9
SLC30A5	73	262	63	210	110	200
	204	343	150	81	158	58
SLC30A6	89	312	80	286	149	264
	292	469	210	95	224	73
SLC30A7	94	188	55	142	104	152
	160	304	146	67	129	66
SLC30A8	36	0	21	0	11	1
	0	0	20	0	12	0
SLC30A9	202	900	210	743	396	716
	816	1049	460	277	706	180
SLC31A1	54	85	26	70	36	39
	51	81	59	26	52	15
SLC31A2	13	43	15	62	26	86
	47	84	38	29	44	13
SLC32A1	9	0	5	0	0	0
	0	0	0	0	3	0
SLC33A1	38	172	51	124	63	136
	124	221	101	49	107	26
SLC34A1	21	0	10	0	2	0
	0	0	4	0	1	0
SLC34A2	37	0	13	0	3	3
	0	0	16	0	9	0
SLC34A3	2	0	3	0	0	0
	0	0	2	0	2	0

SLC35A1	36	157	37	118	62	113
	125	191	65	41	94	34
SLC35A2	13	25	6	15	9	8
	15	36	10	5	16	3
SLC35A3	19	110	20	96	44	68
	104	155	83	31	72	22
SLC35A4	62	250	35	160	86	136
	193	191	98	53	116	33
SLC35A5	36	141	34	121	63	119
	80	173	77	45	93	35
SLC35B1	58	296	45	248	81	225
	277	338	154	66	174	31
SLC35B2	14	35	9	43	38	47
	38	63	36	20	35	9
SLC35B3	48	98	28	70	53	97
	71	156	59	23	66	25
SLC35B4	61	129	37	151	81	133
	104	118	75	48	62	15
SLC35C1	8	21	10	14	19	10
	10	20	10	3	4	6
SLC35C2	26	112	33	73	50	85
	51	116	51	22	72	20
SLC35D1	116	494	115	297	216	292
	298	552	228	130	331	75
SLC35D2	10	32	14	16	14	17
	33	42	22	7	20	12
SLC35D3	4	0	2	0	1	0
	0	0	4	0	0	0
SLC35E1	91	330	81	281	181	226
	261	261	153	104	185	60
SLC35E2	68	317	45	146	106	136
	123	292	91	68	86	37
SLC35E2B	103	228	32	191	164	138
	276	355	116	169	161	54
SLC35E3	38	98	30	73	29	67
	113	92	54	17	71	21
SLC35E4	18	92	21	87	54	112
	67	73	43	27	36	11
SLC35F1	38	7	20	4	17	3
	2	7	19	4	15	4
SLC35F2	18	16	7	16	4	14
	4	14	21	2	11	2
SLC35F3	21	11	6	6	4	4
	3	4	10	2	6	0
SLC35F4	21	0	14	0	4	0
	0	0	12	0	14	1
SLC35F5	155	638	162	548	305	466
	553	976	363	206	512	124
SLC35G1	14	8	9	8	3	10
	8	13	10	9	11	2
SLC35G2	6	22	4	18	6	5
	7	20	14	2	15	4
SLC35G3	9	0	7	2	1	2
	3	0	10	0	2	0
SLC35G4	6	0	3	0	0	0
	0	0	1	0	2	0
SLC35G5	3	1	3	2	1	1
	0	2	1	0	2	0

SLC35G6	2	0	3	0	0	1
	1	0	1	0	0	0
SLC36A1	57	85	41	78	53	118
	56	181	64	28	49	20
SLC36A2	198	781	212	516	493	1342
	821	1526	464	381	414	141
SLC36A3	19	0	4	0	3	0
	0	0	6	0	6	0
SLC36A4	23	28	27	25	22	23
	25	39	31	5	25	11
SLC37A1	29	55	32	42	20	46
	39	38	47	28	36	14
SLC37A2	28	10	14	7	3	8
	0	19	18	0	24	3
SLC37A3	50	112	33	98	57	88
	89	144	78	32	72	31
SLC37A4	70	337	66	341	136	253
	287	255	199	64	170	38
SLC38A1	63	202	54	154	64	227
	189	838	181	56	91	32
SLC38A10	39	90	29	60	44	80
	56	111	46	25	42	19
SLC38A11	8	16	4	27	12	10
	14	25	22	5	12	4
SLC38A2	793	2751	614	2435	2316	2866
	3850	8209	2200	1091	3207	866
SLC38A3	35	88	14	44	25	120
	231	239	69	73	157	46
SLC38A4	113	335	126	162	319	537
	200	744	207	106	166	69
SLC38A5	13	1	4	4	1	3
	2	0	4	0	5	0
SLC38A6	46	118	43	71	73	92
	84	204	70	33	92	34
SLC38A7	26	55	12	35	21	35
	49	69	22	13	37	11
SLC38A8	9	0	2	0	1	0
	0	0	5	0	1	0
SLC38A9	50	132	40	72	46	125
	88	143	78	34	75	19
SLC39A1	72	244	62	189	134	275
	218	375	116	63	149	47
SLC39A10	61	115	56	97	70	114
	86	172	90	42	63	19
SLC39A11	24	31	13	36	26	27
	21	23	32	4	21	5
SLC39A12	15	0	13	0	7	3
	0	1	15	0	5	0
SLC39A13	20	77	25	75	33	60
	64	67	55	14	38	17
SLC39A14	33	111	26	85	46	74
	81	189	71	29	60	30
SLC39A2	5	1	6	0	0	0
	0	0	3	0	0	0
SLC39A3	23	32	11	37	20	46
	36	50	34	12	30	7
SLC39A4	2	4	4	1	1	0
	5	4	2	0	1	1

SLC39A5	5	0	7	1	0	0
	0	0	7	0	2	0
SLC39A6	71	267	87	227	102	274
	182	270	150	83	138	56
SLC39A7	35	131	43	113	64	137
	92	142	63	37	94	22
SLC39A8	35	23	32	20	20	13
	24	31	29	6	22	11
SLC39A9	67	262	71	245	120	175
	185	352	174	58	165	43
SLC3A1	14	4	9	12	4	10
	9	10	8	0	6	1
SLC3A2	64	163	38	142	84	155
	155	184	88	50	98	28
SLC40A1	307	1524	388	1334	707	766
	855	1522	638	457	717	175
SLC41A1	207	808	136	444	361	662
	831	685	294	148	366	162
SLC41A2	30	33	15	32	18	18
	4	28	29	2	20	5
SLC41A3	50	146	36	147	81	190
	178	288	164	69	148	38
SLC43A1	33	66	27	34	39	38
	79	195	66	42	113	29
SLC43A2	18	27	22	32	30	28
	38	98	32	19	50	23
SLC43A3	40	91	28	86	56	93
	72	89	73	38	36	26
SLC44A1	67	267	90	288	115	220
	219	348	170	75	153	42
SLC44A2	130	740	206	510	279	571
	445	643	289	223	351	147
SLC44A3	16	1	12	3	5	1
	1	6	17	0	2	3
SLC44A4	16	0	11	1	5	0
	2	0	14	0	5	0
SLC44A5	96	16	58	137	104	84
	4	354	21	2	129	3
SLC45A1	2	6	2	2	1	2
	2	1	6	1	2	1
SLC45A2	19	7	7	4	4	5
	6	2	7	1	2	0
SLC45A3	31	44	23	19	7	25
	35	20	3	6	39	7
SLC45A4	18	19	16	5	2	8
	5	22	12	3	8	3
SLC46A1	33	65	29	51	31	58
	35	60	43	17	43	8
SLC46A2	9	1	1	0	0	1
	0	0	6	0	2	0
SLC46A3	60	165	42	137	106	132
	162	187	101	55	117	36
SLC47A1	112	343	44	137	214	185
	292	465	176	169	252	99
SLC47A2	9	8	22	12	5	10
	2	2	4	1	4	0
SLC48A1	45	96	19	63	60	69
	86	158	59	36	61	26

SLC4A1	21	43	10	6	10	17
	3	2	15	2	6	2
SLC4A10	47	2	19	0	18	1
	0	0	47	0	18	1
SLC4A11	19	32	12	7	5	6
	8	16	14	4	12	6
SLC4A1AP	86	417	92	262	190	322
	278	438	201	125	262	88
SLC4A2	37	96	30	57	48	82
	70	107	60	23	54	16
SLC4A3	20	8	31	6	2	5
	6	12	12	1	9	2
SLC4A4	77	152	85	154	83	177
	207	130	111	53	97	23
SLC4A5	62	66	50	62	28	72
	48	100	52	15	81	17
SLC4A7	197	621	182	443	306	243
	624	837	348	162	451	210
SLC4A8	98	4	70	7	22	5
	1	7	53	0	26	2
SLC4A9	21	2	15	1	8	2
	0	2	10	1	3	0
SLC50A1	17	20	6	10	7	13
	13	27	17	4	10	2
SLC51A	11	1	2	0	2	0
	0	3	0	0	1	0
SLC51B	7	1	1	0	1	0
	0	0	1	0	1	0
SLC52A1	5	0	1	0	1	0
	0	1	0	0	0	0
SLC52A2	10	5	5	5	8	7
	7	14	7	2	4	3
SLC52A3	10	2	5	3	4	0
	3	2	8	1	2	2
SLC5A1	47	7	28	7	17	7
	2	0	15	5	13	0
SLC5A10	11	0	5	0	1	0
	0	0	3	0	2	0
SLC5A11	18	1	13	0	2	0
	0	1	6	0	2	0
SLC5A12	52	2	38	2	11	7
	0	0	32	1	16	1
SLC5A2	3	1	6	2	0	0
	0	0	3	1	4	0
SLC5A3	0	0	0	0	3	0
	0	1	0	0	0	0
SLC5A4	20	0	7	0	7	0
	0	2	10	0	9	0
SLC5A5	14	0	4	0	1	0
	0	0	4	0	2	0
SLC5A6	54	60	31	39	54	69
	54	106	41	17	49	21
SLC5A7	37	4	21	2	11	0
	2	4	16	0	11	1
SLC5A8	24	0	24	0	12	0
	0	0	21	0	8	0
SLC5A9	22	0	14	1	6	0
	0	2	6	0	3	0

SLC6A1	47	39	19	40	20	41
	27	46	41	9	29	8
SLC6A11	16	0	18	0	3	1
	0	0	7	0	3	0
SLC6A12	26	0	9	2	11	2
	0	1	18	0	8	0
SLC6A13	30	17	11	15	16	52
	5	11	18	4	14	1
SLC6A14	21	1	6	0	10	0
	1	0	12	1	6	0
SLC6A15	36	0	25	3	7	3
	1	4	37	0	12	0
SLC6A16	23	6	12	5	11	4
	13	12	13	6	10	1
SLC6A17	33	1	13	0	4	1
	0	0	10	0	6	0
SLC6A18	11	0	2	0	0	0
	0	0	3	0	0	0
SLC6A19	14	0	8	0	4	0
	0	0	8	0	0	0
SLC6A2	23	0	26	1	6	3
	0	4	16	0	8	2
SLC6A20	38	10	19	6	5	3
	10	7	14	3	15	1
SLC6A3	29	0	11	0	2	0
	0	0	11	0	4	0
SLC6A4	23	1	8	2	4	3
	0	1	20	0	11	2
SLC6A5	24	0	8	0	2	0
	0	0	7	0	8	0
SLC6A6	101	286	143	226	180	263
	217	391	156	106	182	61
SLC6A7	17	0	12	0	6	1
	0	2	5	0	2	0
SLC6A8	78	473	53	273	146	333
	374	267	111	92	159	35
SLC6A9	17	5	11	4	4	7
	3	8	6	3	6	0
SLC7A1	45	34	23	24	14	25
	29	28	27	5	22	8
SLC7A10	7	0	4	0	5	0
	0	0	1	0	1	0
SLC7A11	46	5	38	27	16	21
	1	6	25	3	10	1
SLC7A13	14	0	10	0	3	5
	0	2	14	0	6	0
SLC7A14	48	0	25	0	8	0
	1	1	33	0	20	1
SLC7A2	1054	4756	1020	4298	3372	4388
	5413	8839	3181	2217	3538	1200
SLC7A3	17	0	3	0	3	0
	0	0	7	0	3	0
SLC7A4	7	2	3	6	0	1
	2	1	0	0	2	0
SLC7A5	16	27	9	20	18	24
	17	28	13	10	16	10
SLC7A6	131	601	276	546	150	424
	521	676	290	164	318	151

SLC7A6OS	20	102	26	61	50	78
	86	109	43	41	57	12
SLC7A7	26	9	11	7	11	11
	11	10	21	4	11	0
SLC7A8	36	101	32	73	45	47
	88	115	64	26	45	12
SLC7A9	20	1	14	0	4	4
	0	0	4	0	1	0
SLC8A1	50	113	42	84	43	65
	69	126	73	28	65	28
SLC8A2	13	1	14	0	4	1
	0	0	4	1	6	0
SLC8A3	132	470	132	310	181	395
	332	661	283	188	322	103
SLC9A1	27	55	28	29	27	42
	50	53	23	15	26	12
SLC9A2	67	200	38	184	130	176
	223	137	63	37	113	42
SLC9A3	14	0	10	0	1	0
	0	0	3	0	1	0
SLC9A3R1	9	7	9	5	7	7
	14	13	7	4	3	3
SLC9A3R2	10	172	6	67	58	65
	150	115	66	32	79	14
SLC9A4	33	0	24	1	5	0
	0	0	17	0	8	0
SLC9A5	22	14	8	4	7	8
	5	18	12	5	15	5
SLC9A6	70	210	57	173	106	195
	150	261	111	66	133	40
SLC9A7	115	476	121	284	194	257
	291	363	170	107	171	65
SLC9A8	83	239	59	194	107	158
	147	431	148	96	153	56
SLC9A9	46	71	36	60	50	87
	46	129	85	25	57	21
SLC9B1	25	8	8	6	13	11
	8	21	30	7	14	5
SLC9B2	30	24	18	20	21	18
	13	43	22	5	25	7
SLC9C1	38	21	25	32	33	15
	33	21	25	8	12	1
SLC9C2	38	1	21	1	11	0
	1	1	16	0	14	0
SLCO1A2	39	0	37	1	16	0
	2	0	39	0	14	0
SLCO1B1	18	0	17	0	6	0
	0	0	15	0	7	1
SLCO1B3	19	0	14	1	12	0
	0	3	17	0	8	0
SLCO1B7	9	0	10	1	6	0
	0	2	9	0	4	0
SLCO1C1	35	7	9	10	4	0
	7	2	16	1	12	0
SLCO2A1	29	3	17	1	3	0
	3	4	13	2	12	7
SLCO2B1	34	55	20	42	34	26
	46	83	40	17	31	8

SLCO3A1	46	57	35	48	19	37
	29	47	29	9	38	21
SLCO4A1	12	13	8	2	9	1
	6	12	3	1	10	1
SLCO4C1	45	7	32	6	10	4
	6	4	28	3	14	1
SLCO5A1	108	365	124	234	201	273
	372	446	250	121	321	81
SLCO6A1	4	0	5	0	4	0
	0	2	11	0	5	0
SLFN11	63	256	67	185	106	147
	167	235	105	66	128	35
SLFN12	38	42	17	23	18	21
	34	34	29	9	19	5
SLFN12L	8	10	7	5	4	7
	2	9	14	3	5	2
SLFN13	54	23	36	24	15	9
	13	17	30	8	28	5
SLFN14	23	1	10	0	1	1
	1	1	5	0	3	0
SLFN5	80	360	117	358	211	248
	164	367	188	115	254	137
SLFNL1	8	6	7	0	3	1
	0	1	1	0	1	0
SLIRP	86	509	49	370	175	266
	533	726	219	98	266	62
SLIT1	40	2	24	4	16	5
	1	3	15	5	16	0
SLIT2	61	235	71	134	161	86
	151	115	85	57	88	26
SLIT3	41	104	32	83	41	41
	32	73	44	11	30	25
SLITRK1	34	1	16	0	3	0
	0	1	14	0	4	1
SLITRK2	19	1	3	0	9	0
	0	0	8	0	4	3
SLITRK3	29	2	15	5	9	0
	2	3	8	0	9	0
SLITRK4	87	276	31	166	50	112
	390	424	46	26	95	25
SLITRK5	24	9	10	11	9	1
	7	3	11	2	7	1
SLITRK6	19	5	15	3	10	3
	0	2	9	1	16	1
SLK	257	1313	305	924	760	991
	965	1315	529	469	773	266
SLMAP	432	1931	257	1147	1051	1422
	1383	2080	858	552	1014	343
SLMO1	9	4	3	2	3	3
	1	5	0	1	5	1
SLMO2	73	234	54	194	95	209
	193	309	108	77	148	58
SLN	4682	26042	3295	20679	10797	15360
	28299	31565	17321	5215	16571	3366
SLPI	7	12	8	9	3	4
	1	25	5	2	9	3
SLTM	237	1185	246	730	498	798
	814	1282	491	381	676	209

SLU7	126	535	110	434	300	409
	410	580	265	189	342	114
SLURP1	2	0	0	0	0	0
	0	0	0	0	0	0
SLX1A	10	23	5	24	14	27
	31	48	27	8	11	5
SLX1B	3	18	5	26	5	21
	22	30	8	4	20	3
SLX4	58	81	35	45	46	55
	47	80	30	30	50	8
SMAD1	46	191	52	139	87	134
	162	199	125	62	127	47
SMAD2	230	1127	291	934	521	948
	912	1242	601	357	694	172
SMAD3	236	917	174	748	469	845
	1324	1098	503	427	612	219
SMAD4	291	1094	300	1169	681	902
	1124	1410	771	390	892	273
SMAD5	219	844	193	713	463	745
	794	1080	491	254	581	168
SMAD6	6	16	8	7	3	6
	6	7	7	2	9	6
SMAD7	24	88	10	81	34	63
	68	60	37	20	45	23
SMAD9	37	131	47	102	44	83
	100	224	132	37	125	38
SMAGP	10	7	1	6	4	0
	13	3	6	4	4	1
SMAP1	90	420	61	246	164	382
	342	337	170	119	198	50
SMAP2	46	88	28	52	42	53
	56	86	40	19	42	12
SMARCA1	172	661	183	442	474	421
	430	526	216	200	312	106
SMARCA2	402	2094	410	1168	1130	1357
	1124	1761	734	645	1025	451
SMARCA4	80	456	98	228	149	287
	252	318	144	116	181	66
SMARCA5	237	1093	240	799	577	729
	900	1235	520	393	689	223
SMARCAD1	128	446	128	309	202	363
	322	520	228	132	299	93
SMARCAL1	39	126	34	85	42	63
	102	63	64	26	53	20
SMARCB1	26	121	20	68	58	103
	138	102	55	54	65	28
SMARCC1	106	438	148	328	197	271
	262	305	150	113	222	81
SMARCC2	167	866	166	578	463	646
	564	727	296	278	396	155
SMARCD1	36	121	37	78	40	88
	89	84	59	33	65	18
SMARCD2	63	157	35	107	103	120
	152	302	99	66	103	40
SMARCD3	125	501	140	335	199	421
	524	601	278	179	289	82
SMARCE1	107	578	111	352	298	484
	470	713	256	245	354	136

SMC1A	215	612	154	367	385	438
	469	596	205	264	356	125
SMC1B	59	0	26	1	5	0
	0	1	40	0	14	0
SMC2	66	142	83	112	100	77
	102	129	84	38	102	30
SMC3	156	795	198	501	403	655
	546	770	294	302	390	135
SMC4	71	336	93	196	197	214
	226	323	147	119	186	56
SMC5	188	781	219	519	359	588
	577	960	389	324	492	171
SMC6	152	538	148	367	343	455
	438	630	236	224	333	114
SMCHD1	307	1390	423	1029	787	1075
	1110	1594	807	515	849	343
SMCP	1	0	1	0	1	0
	0	0	2	0	0	0
SMCR7	49	164	33	128	98	165
	160	218	116	46	108	21
SMCR7L	64	158	31	109	88	122
	150	178	101	47	115	35
SMCR8	95	266	75	200	160	199
	214	347	133	111	187	47
SMCR9	3	0	0	0	1	0
	0	0	2	0	0	0
SMEK1	105	678	147	492	315	484
	536	666	307	197	404	136
SMEK2	153	661	175	487	391	578
	506	788	286	232	459	175
SMG1	1469	7142	2233	5182	3483	5318
	5713	9718	2985	2449	4805	2064
SMG5	189	560	107	383	306	509
	408	750	361	219	349	130
SMG6	102	362	94	262	170	295
	215	331	153	112	182	73
SMG7	112	546	133	293	207	279
	313	418	165	104	200	65
SMG8	45	112	27	92	93	87
	96	150	62	45	60	28
SMG9	18	48	21	46	16	41
	45	37	40	12	27	10
SMIM1	0	1	1	0	0	0
	0	0	0	1	2	0
SMIM3	15	20	6	17	16	31
	12	65	23	5	23	11
SMIM4	27	133	27	100	56	106
	165	195	48	35	56	19
SMIM5	2	0	1	0	0	0
	1	0	3	0	0	0
SMIM6	2	0	1	0	0	0
	0	0	1	0	1	0
SMN1	66	183	45	98	108	136
	168	209	90	86	142	17
SMN2	36	242	25	7	103	172
	80	153	127	81	167	31
SMNDC1	30	164	28	127	83	104
	125	149	47	51	77	24

SMO	28	33	19	30	13	20
	16	17	13	2	12	4
SMOC1	56	113	57	72	56	49
	99	100	69	57	81	17
SMOC2	134	682	116	603	333	608
	487	585	438	207	435	97
SMOX	10	8	14	7	5	11
	5	11	9	4	10	2
SMPD1	28	95	20	88	44	112
	78	129	49	28	59	21
SMPD2	9	7	8	7	7	7
	10	5	7	0	6	1
SMPD3	17	4	15	4	2	8
	3	6	10	1	4	1
SMPD4	50	148	43	87	61	85
	109	152	69	38	75	31
SMPD5	1	1	0	0	0	0
	0	0	0	0	0	0
SMPDL3A	90	344	71	265	209	263
	507	484	228	143	285	69
SMPDL3B	15	2	2	0	2	0
	0	0	7	0	7	0
SMPX	650	3444	448	2196	1294	1770
	3499	5545	2227	1139	2506	520
SMR3A	4	0	1	0	1	0
	0	0	4	0	1	0
SMR3B	8	0	4	0	0	0
	0	0	2	0	1	0
SMS	63	379	46	170	135	178
	315	357	129	105	170	64
SMTN	85	347	98	251	150	287
	381	273	176	78	214	63
SMTNL1	494	1848	279	792	1431	1764
	983	3771	934	1216	2247	725
SMTNL2	189	961	258	898	605	823
	711	1041	364	260	405	147
SMU1	189	761	209	560	392	510
	582	880	417	286	532	148
SMUG1	25	87	14	64	46	67
	44	109	41	20	50	18
SMURF1	65	154	44	129	75	95
	99	184	83	63	96	35
SMURF2	83	253	49	209	139	187
	198	252	135	79	169	73
SMYD1	903	5978	1044	3538	2268	3959
	4400	4948	2072	1323	3107	1002
SMYD2	125	559	114	401	261	456
	634	698	294	124	310	97
SMYD3	35	77	19	84	35	58
	89	61	47	35	69	16
SMYD4	69	164	56	146	111	118
	219	199	121	75	116	37
SMYD5	33	68	27	53	26	53
	49	77	38	20	49	13
SNAI1	3	1	2	0	1	1
	0	0	2	0	6	3
SNAI2	17	42	24	29	26	26
	38	42	22	6	17	10

SNAI3	6	53	18	38	16	21
	53	86	40	3	68	7
SNAP23	89	417	83	205	199	227
	272	572	212	109	212	87
SNAP25	15	1	7	0	6	0
	0	1	7	0	5	0
SNAP29	62	320	49	181	125	182
	253	285	127	65	158	61
SNAP47	34	88	27	98	72	106
	88	152	71	32	64	30
SNAP91	36	3	41	2	15	8
	7	4	40	1	23	2
SNAPC1	53	195	37	117	115	128
	116	241	54	51	84	23
SNAPC2	6	14	4	2	8	4
	2	9	3	0	5	0
SNAPC3	81	401	102	292	195	325
	281	380	182	125	250	79
SNAPC4	26	55	16	38	20	37
	39	68	27	14	29	14
SNAPC5	33	132	20	111	43	100
	85	158	90	38	67	17
SNAPIN	53	200	30	117	72	163
	172	270	110	60	125	20
SNCA	23	123	11	16	17	61
	28	22	15	10	11	4
SNCAIP	37	25	26	29	14	11
	18	11	27	6	17	12
SNCB	9	0	1	0	1	0
	1	0	8	1	2	0
SNCG	6	31	8	20	11	8
	16	32	6	3	9	11
SND1	92	279	78	221	102	187
	180	325	124	81	160	55
SNED1	58	128	38	92	60	97
	182	242	137	91	134	88
SNF8	29	173	27	117	63	104
	181	184	100	38	91	27
SNIP1	50	114	37	75	78	95
	117	186	79	39	105	24
SNN	48	134	33	166	54	125
	144	201	82	78	95	41
SNPH	12	43	13	31	27	37
	16	25	12	7	19	4
SNRK	96	529	98	380	228	292
	470	608	259	159	294	122
SNRNP200	272	1382	298	1082	580	775
	904	1115	473	385	600	286
SNRNP25	54	179	20	117	53	149
	220	323	114	63	149	16
SNRNP27	96	612	128	315	331	435
	698	690	211	169	303	96
SNRNP35	34	138	27	53	63	70
	95	140	51	34	81	17
SNRNP40	40	225	50	143	101	190
	164	306	133	74	147	34
SNRNP48	83	264	76	223	195	274
	214	360	148	117	198	64

SNRNP70	161	542	114	303	274	504
	523	907	343	207	346	149
SNRPA	23	89	11	47	42	52
	39	89	36	24	47	11
SNRPA1	18	78	20	82	37	50
	86	82	53	23	66	24
SNRPB	45	97	34	103	52	84
	145	204	91	50	101	26
SNRPB2	86	352	65	221	183	270
	334	469	188	107	208	47
SNRPC	79	293	53	166	114	207
	261	385	149	93	170	52
SNRPD1	41	186	30	91	96	145
	169	240	106	62	147	32
SNRPD2	61	266	29	159	100	133
	371	451	132	109	152	36
SNRPD3	87	420	65	285	186	289
	379	528	221	101	252	66
SNRPE	66	293	56	250	121	230
	343	622	216	89	223	34
SNRPF	22	155	19	114	49	68
	157	148	61	39	78	12
SNRPG	35	198	34	108	102	123
	177	334	81	43	91	36
SNRPN	165	626	215	775	320	745
	619	627	591	155	505	103
SNTA1	154	842	101	749	284	602
	688	965	472	290	536	156
SNTB1	103	524	214	493	258	476
	410	340	265	163	213	73
SNTB2	126	465	116	401	283	384
	270	463	214	138	214	116
SNTG1	29	0	21	0	11	0
	0	0	15	0	6	0
SNTG2	11	10	11	5	9	2
	5	8	16	3	12	5
SNTN	13	0	9	0	2	0
	0	0	4	0	1	0
SNUPN	55	171	29	119	70	94
	152	194	72	59	77	34
SNURF	8	438	1	65	23	232
	264	188	11	31	23	56
SNW1	114	439	108	269	240	340
	319	509	228	164	302	102
SNX1	208	644	163	441	289	525
	451	875	313	242	405	195
SNX10	17	17	7	5	11	11
	21	20	12	1	12	3
SNX11	13	30	13	32	12	28
	30	38	20	10	16	6
SNX12	58	162	30	132	64	163
	130	202	91	48	110	23
SNX13	250	1374	307	1152	693	1163
	989	1404	718	419	911	278
SNX14	101	405	118	394	199	345
	310	335	218	122	242	77
SNX15	21	42	20	45	27	57
	26	32	45	16	36	15

SNX16	43	107	39	81	57	88
	101	120	65	39	89	18
SNX17	33	145	40	114	75	92
	138	183	87	44	90	31
SNX18	85	317	69	239	147	222
	206	297	144	90	148	59
SNX19	198	998	145	704	447	567
	603	911	328	264	431	162
SNX2	78	328	90	276	213	355
	296	400	183	113	262	60
SNX20	23	16	6	7	10	7
	14	23	11	5	22	4
SNX21	57	205	52	192	95	155
	145	189	65	44	92	33
SNX22	10	5	9	2	3	3
	1	5	10	1	4	2
SNX24	32	96	33	96	45	72
	101	118	65	33	70	24
SNX25	56	170	56	126	86	141
	158	175	91	60	101	41
SNX27	195	738	164	640	470	568
	537	636	294	193	393	133
SNX29	84	225	60	162	94	158
	138	202	111	64	108	43
SNX3	175	1155	151	592	418	593
	1036	1117	392	249	513	146
SNX30	68	166	52	114	82	111
	102	175	77	34	78	27
SNX31	26	4	12	2	9	10
	2	26	15	0	16	4
SNX32	10	0	3	0	1	0
	0	1	5	0	1	0
SNX33	22	65	14	53	25	61
	32	53	34	15	26	21
SNX4	92	366	113	297	173	288
	292	398	220	122	290	51
SNX5	107	708	135	481	314	586
	609	782	305	225	355	115
SNX6	141	662	129	498	301	606
	513	630	300	202	346	85
SNX7	14	103	85	52	15	28
	31	30	29	11	34	8
SNX8	15	36	17	27	16	20
	28	40	28	22	30	5
SNX9	92	380	70	335	224	324
	344	413	202	114	254	72
SOAT1	59	105	37	66	59	58
	74	129	67	26	70	25
SOAT2	17	0	8	0	2	0
	0	2	4	1	1	0
SOBP	88	429	62	179	175	209
	211	227	102	81	131	51
SOCS1	2	3	0	0	2	1
	1	4	3	0	1	0
SOCS2	19	20	12	17	10	11
	23	56	23	1	8	3
SOCS3	10	23	13	20	14	24
	8	51	16	9	11	5

SOCS4	68	209	64	194	141	218
	214	277	146	87	168	63
SOCS5	105	422	106	349	206	311
	254	445	202	121	215	81
SOCS6	188	803	196	700	380	488
	591	780	371	211	459	132
SOCS7	100	306	100	222	172	251
	199	330	145	90	130	66
SOD1	327	1508	260	1139	700	1133
	1678	2084	651	323	766	193
SOD2	451	2814	477	2230	1081	1622
	2893	3434	1311	709	1708	559
SOD3	4	7	2	6	4	2
	5	9	10	1	1	6
SOGA1	55	141	29	82	65	63
	81	105	58	33	42	22
SOGA2	36	67	35	58	38	62
	51	39	43	14	39	8
SOGA3	395	557	320	322	633	349
	310	518	551	246	712	76
SOHLH1	13	0	5	0	2	0
	0	0	4	0	0	0
SOHLH2	0	0	0	0	0	2
	0	0	0	0	0	0
SOLH	10	31	11	21	20	28
	19	26	15	9	15	6
SON	537	3339	616	2418	1420	2007
	2376	3506	1209	927	1661	565
SORBS1	955	4410	848	2779	1879	2562
	3430	6229	1460	1552	2796	1238
SORBS2	77	112	70	61	41	34
	61	96	69	29	78	31
SORBS3	33	128	26	79	41	78
	122	178	68	45	104	30
SORCS1	68	47	46	15	40	19
	28	72	48	12	33	9
SORCS2	24	20	26	25	15	13
	4	7	17	2	11	3
SORCS3	52	3	31	0	18	0
	0	2	25	0	22	0
SORD	24	33	10	11	14	13
	38	16	13	7	13	2
SORL1	144	212	72	281	94	176
	102	150	140	53	123	25
SORT1	265	2351	371	1327	786	1129
	1399	1572	732	401	832	288
SOS1	276	1369	341	848	768	812
	1129	1262	440	332	580	293
SOS2	360	2714	568	2249	1423	2153
	1899	1729	1100	749	1264	457
SOST	6	0	5	0	0	0
	0	0	3	0	0	0
SOSTDC1	9	2	10	1	1	1
	1	4	2	0	3	0
SOWAHA	3	0	2	0	0	0
	0	1	4	0	2	1
SOWAHB	8	0	6	1	0	0
	1	0	6	0	3	0

SOWAHC	11	37	14	27	10	24
	19	27	18	9	26	4
SOWAHD	0	0	0	1	0	0
	0	0	0	0	0	0
SOX1	17	0	5	0	2	0
	0	0	5	0	0	0
SOX10	10	1	6	1	0	1
	0	0	4	0	4	4
SOX11	38	17	23	5	7	3
	1	3	34	1	11	2
SOX12	2	9	8	3	1	5
	6	2	7	3	5	0
SOX13	16	66	20	51	40	47
	91	116	49	24	49	37
SOX14	5	0	1	0	0	0
	0	0	2	0	2	0
SOX15	3	1	6	0	5	2
	4	1	4	1	5	2
SOX17	12	33	9	10	10	8
	16	15	11	5	13	5
SOX18	3	11	2	2	5	4
	8	13	3	3	0	3
SOX2	1	2	1	1	0	2
	0	2	1	1	0	1
SOX21	5	1	1	0	1	1
	0	0	1	0	2	0
SOX3	5	0	1	0	0	0
	0	0	0	0	0	0
SOX30	17	0	10	0	3	1
	0	0	5	0	4	0
SOX4	30	116	33	104	45	71
	38	53	55	27	28	15
SOX5	50	42	38	52	44	31
	24	49	43	13	42	11
SOX6	203	1205	263	787	475	732
	992	1169	614	389	750	273
SOX7	14	82	11	30	24	19
	44	39	26	22	17	20
SOX8	10	3	11	9	3	15
	2	5	9	2	4	0
SOX9	17	19	23	50	29	25
	51	52	40	15	29	13
SP1	253	1006	228	873	560	792
	831	1321	508	332	628	241
SP100	184	372	129	273	179	258
	281	344	257	185	226	90
SP110	53	65	29	40	49	48
	50	61	49	40	54	14
SP140	51	10	30	4	7	2
	5	7	38	2	25	1
SP140L	43	93	32	52	48	31
	52	57	57	34	53	17
SP2	27	53	14	26	41	22
	36	22	19	23	31	14
SP3	168	925	205	763	538	865
	733	1028	484	284	526	191
SP4	66	244	97	218	157	207
	180	343	156	100	213	76

SP5	4	3	3	0	1	0
	0	0	3	1	2	0
SP6	15	0	17	1	1	0
	0	0	8	1	3	0
SP7	15	2	4	0	3	0
	0	0	7	0	3	0
SP8	19	0	5	0	5	0
	0	0	8	0	3	0
SP9	0	0	1	0	0	0
	0	0	0	0	0	0
SPA17	15	4	6	7	4	5
	4	3	9	1	7	3
SPACA1	7	0	16	0	9	0
	0	0	9	0	2	0
SPACA3	2	0	0	0	1	0
	0	0	1	0	5	0
SPACA4	2	0	0	0	0	0
	0	0	2	0	0	0
SPACA5	8	0	2	0	1	0
	0	0	2	0	0	0
SPACA5B	1	0	1	0	0	0
	0	0	0	0	0	0
SPACA7	14	0	2	0	0	0
	0	0	8	0	3	0
SPAG1	25	7	15	11	7	6
	0	10	33	2	14	6
SPAG11A	2	0	1	0	1	0
	0	0	3	0	2	0
SPAG11B	2	0	5	0	1	0
	0	0	4	0	2	0
SPAG16	33	90	46	57	38	56
	86	137	61	19	67	26
SPAG17	84	38	55	35	72	52
	25	70	79	18	80	24
SPAG4	2	2	6	0	1	0
	4	0	4	0	2	2
SPAG5	38	15	22	17	5	12
	4	23	12	3	20	3
SPAG6	22	0	14	1	4	2
	3	1	17	0	12	0
SPAG7	129	879	145	502	332	483
	812	1008	340	181	478	143
SPAG8	18	15	12	7	11	13
	7	12	13	5	15	1
SPAG9	665	2459	694	2048	1396	2105
	1904	2991	1420	953	1765	613
SPAM1	27	36	23	60	21	35
	5	40	15	3	20	1
SPANXA1	0	0	1	0	0	0
	0	0	0	0	0	0
SPANXA2	0	0	0	0	0	0
	0	0	1	0	0	0
SPANXB1	3	0	1	0	1	0
	0	0	0	0	0	0
SPANXB2	2	0	3	0	1	0
	0	0	1	0	0	0
SPANXC	3	0	0	0	1	0
	0	0	0	0	0	0

SPANXD	1	0	1	0	0	0
	0	0	1	0	0	0
SPANXN1	3	0	0	0	1	0
	0	0	3	0	0	0
SPANXN2	21	0	9	0	7	0
	0	1	1	0	1	0
SPANXN3	3	0	4	0	1	0
	0	0	1	0	1	0
SPANXN4	15	0	1	0	1	0
	0	0	1	0	1	0
SPANXN5	2	0	0	0	1	0
	0	0	2	0	0	0
SPARC	303	2388	492	1941	1210	1486
	1799	1709	886	519	940	517
SPARCL1	525	3437	386	1765	1268	1746
	2153	3526	1640	1201	1707	702
SPAST	103	350	113	317	211	330
	357	423	205	113	236	72
SPATA1	17	8	9	5	7	6
	14	11	14	2	11	1
SPATA12	18	0	7	0	5	0
	0	0	7	0	4	1
SPATA13	67	107	24	77	42	53
	66	84	63	47	94	26
SPATA16	28	1	10	0	9	0
	1	0	6	0	16	0
SPATA17	26	5	6	9	15	5
	3	8	6	1	12	0
SPATA18	46	36	19	40	19	30
	15	15	33	4	15	3
SPATA19	7	1	9	0	3	0
	0	0	5	0	3	0
SPATA2	15	65	10	58	39	42
	53	51	23	18	32	2
SPATA20	43	194	52	134	76	125
	64	183	57	27	99	26
SPATA21	19	0	4	0	2	1
	1	0	7	1	1	1
SPATA22	18	6	10	12	18	11
	11	5	21	10	7	0
SPATA24	17	32	16	33	34	41
	61	69	26	15	31	8
SPATA25	7	11	2	5	4	9
	7	18	11	2	11	1
SPATA2L	2	1	1	0	0	0
	0	1	2	0	6	0
SPATA3	12	0	1	0	2	0
	0	0	0	0	0	0
SPATA31A1	6	0	2	0	1	0
	0	0	10	0	4	0
SPATA31A2	12	0	3	0	2	0
	0	0	2	0	6	0
SPATA31A3	31	0	18	1	2	0
	0	0	2	0	1	0
SPATA31A4	8	0	6	0	1	0
	0	0	2	0	3	0
SPATA31A5	3	0	1	0	1	1
	0	0	3	0	3	0

SPATA31A6	39	0	22	0	6	0
	0	0	1	0	3	0
SPATA31A7	7	0	3	1	0	0
	0	0	1	0	4	0
SPATA31C1	27	0	12	0	1	0
	0	0	10	0	4	0
SPATA31C2	18	0	11	0	10	0
	0	0	9	0	7	0
SPATA31D1	29	0	14	1	5	0
	0	0	13	0	9	0
SPATA31D3	35	0	22	0	5	1
	0	0	11	0	5	0
SPATA31D4	21	0	5	1	5	0
	0	0	10	0	2	0
SPATA31E1	13	0	11	0	2	0
	0	0	4	0	3	0
SPATA32	9	0	3	0	2	0
	0	0	8	0	2	0
SPATA4	15	8	3	2	0	7
	18	11	6	1	3	2
SPATA5	99	239	89	156	177	170
	140	289	141	85	164	34
SPATA5L1	38	87	26	64	36	69
	71	119	51	43	65	22
SPATA6	38	73	34	77	40	39
	54	117	36	21	40	16
SPATA6L	23	1	18	7	7	6
	3	7	13	3	13	3
SPATA7	28	106	46	105	59	110
	137	162	52	46	109	9
SPATA8	1	0	2	0	2	0
	0	0	0	0	2	0
SPATA9	7	0	5	0	3	3
	1	3	13	0	2	0
SPATC1	5	0	3	1	2	0
	1	0	3	0	0	0
SPATS1	12	0	11	0	2	0
	0	0	6	1	2	0
SPATS2	74	165	59	135	65	102
	120	196	78	50	106	24
SPATS2L	129	671	124	443	233	514
	547	549	238	160	346	109
SPC24	1	1	0	0	0	0
	0	0	0	0	0	0
SPC25	14	0	6	0	1	2
	4	1	3	0	1	0
SPCS1	55	315	58	214	105	240
	343	368	170	108	204	67
SPCS2	55	268	65	229	114	214
	248	297	118	84	177	38
SPCS3	154	825	164	758	422	712
	737	1131	406	260	535	135
SPDEF	0	0	2	0	0	0
	0	2	2	0	3	0
SPDL1	31	90	47	54	38	56
	56	70	25	20	65	9
SPDYA	9	0	7	2	4	2
	0	1	6	1	3	0

SPDYC	10	3	2	0	4	0
	0	0	3	0	1	0
SPDYE1	26	5	10	0	4	6
	0	6	15	1	14	1
SPDYE2	46	15	34	9	14	17
	8	17	39	9	19	2
SPDYE2L	8	3	3	2	3	3
	2	2	12	1	6	4
SPDYE3	36	1	30	0	5	3
	2	5	22	0	18	0
SPDYE4	4	0	8	0	3	0
	0	0	4	0	4	0
SPDYE5	18	6	16	1	13	4
	0	14	11	3	16	1
SPDYE6	47	2	8	1	11	7
	6	6	12	0	9	1
SPECC1	98	187	69	115	114	125
	134	161	85	87	129	52
SPECC1L	160	688	193	530	349	496
	437	527	231	233	338	152
SPEF1	4	0	7	0	2	0
	0	0	6	0	1	0
SPEF2	52	26	54	36	22	23
	26	38	55	13	41	6
SPEG	486	2565	465	1532	870	1706
	1259	2018	706	663	866	371
SPEM1	2	0	0	0	1	0
	0	0	1	0	1	0
SPEN	201	1143	286	735	587	705
	769	839	292	366	421	257
SPERT	3	0	4	0	1	0
	0	0	1	0	1	0
SPESP1	0	2	0	2	3	3
	2	5	1	0	0	3
SPG11	289	1057	248	708	594	719
	784	1523	578	390	694	247
SPG20	115	398	103	371	253	348
	294	513	242	153	262	93
SPG21	28	104	14	108	52	83
	93	115	81	29	54	17
SPG7	118	671	117	432	261	474
	516	699	303	202	361	95
SPHAR	10	63	20	84	20	62
	74	85	38	21	36	10
SPHK1	5	6	1	12	2	1
	1	7	4	0	4	1
SPHK2	14	15	17	6	10	9
	22	19	12	7	17	8
SPHKAP	30	1	31	0	12	0
	0	0	23	0	9	1
SPI1	5	8	1	6	5	6
	10	16	3	1	4	1
SPIB	13	1	12	2	3	3
	0	2	5	1	1	0
SPIC	9	0	7	0	1	0
	0	0	3	0	0	0
SPICE1	52	169	67	119	101	151
	105	176	106	52	127	42

SPIN1	35	110	53	120	46	79
	99	124	74	38	73	29
SPIN2A	7	5	8	1	4	3
	1	0	2	0	2	1
SPIN2B	5	24	8	14	16	22
	19	13	15	6	8	3
SPIN3	49	125	33	77	71	105
	66	112	62	20	61	15
SPIN4	9	14	10	22	9	17
	12	23	10	1	11	1
SPINK1	6	3	6	4	3	1
	0	0	4	0	1	0
SPINK13	4	0	1	0	1	0
	0	0	1	0	0	0
SPINK14	1	0	1	0	0	0
	0	0	0	0	1	0
SPINK2	4	0	1	2	1	0
	0	3	4	0	1	1
SPINK4	5	0	1	0	0	0
	0	0	0	0	1	0
SPINK5	69	10	49	1	19	3
	8	9	30	12	32	4
SPINK6	8	0	4	0	0	0
	0	0	5	0	1	0
SPINK7	4	0	1	1	0	0
	0	1	2	0	3	0
SPINK8	5	0	5	0	0	0
	0	0	3	0	0	0
SPINK9	2	1	4	0	1	0
	0	1	2	0	0	0
SPINT1	5	2	5	0	2	1
	2	0	3	1	1	0
SPINT2	8	3	6	2	8	5
	4	8	7	2	0	1
SPINT3	0	0	2	0	0	0
	0	0	1	0	1	0
SPINT4	2	0	2	0	0	0
	0	0	1	0	0	0
SPIRE1	118	586	110	466	240	409
	396	382	290	148	235	81
SPIRE2	6	1	3	0	5	0
	0	4	6	0	5	0
SPN	48	18	25	8	7	8
	5	14	14	1	15	2
SPNS1	11	31	8	34	20	20
	25	46	22	13	28	13
SPNS2	15	24	11	12	9	19
	17	12	16	6	15	5
SPNS3	9	0	1	0	1	0
	0	0	1	0	2	0
SPO11	12	0	3	0	1	0
	0	0	5	0	8	0
SPOCD1	15	0	9	0	1	0
	0	0	5	0	5	0
SPOCK1	37	76	42	92	15	38
	52	125	72	12	45	22
SPOCK2	38	44	27	42	52	28
	30	74	37	19	24	5

SPOCK3	25	5	15	0	36	17
	4	2	26	1	12	0
SPON1	34	6	18	1	7	3
	5	3	18	0	12	5
SPON2	28	68	14	53	25	49
	37	60	35	17	33	10
SPOP	137	612	160	431	305	587
	547	667	318	188	390	98
SPOPL	92	309	88	239	157	215
	271	302	152	88	157	52
SPP1	14	2	10	1	5	1
	0	0	7	0	5	0
SPP2	11	0	2	0	4	0
	0	0	6	0	2	0
SPPL2A	293	1223	212	1066	814	716
	763	1847	496	361	892	259
SPPL2B	25	73	20	26	23	35
	30	120	48	21	46	16
SPPL2C	10	0	2	1	0	0
	0	0	1	0	0	0
SPPL3	42	118	35	138	55	91
	85	113	60	19	80	13
SPR	15	74	12	42	42	62
	70	100	41	25	45	11
SPRED1	87	219	55	244	151	248
	138	279	194	98	172	60
SPRED2	39	91	35	88	50	69
	90	96	57	29	52	13
SPRED3	13	1	12	0	1	1
	0	5	6	1	4	0
SPRN	11	3	5	1	0	0
	1	0	0	1	2	0
SPRR1A	2	0	0	0	2	0
	0	0	0	0	0	0
SPRR1B	2	0	1	0	0	0
	0	0	0	0	0	0
SPRR2A	2	0	3	0	2	0
	0	0	0	0	0	0
SPRR2B	5	0	1	0	1	0
	0	0	0	0	0	0
SPRR2D	3	0	4	0	0	0
	0	0	3	0	1	1
SPRR2E	1	0	1	0	0	0
	0	0	1	0	0	0
SPRR2F	5	0	1	0	0	0
	0	0	1	0	1	0
SPRR2G	4	0	3	0	2	0
	0	0	0	0	0	0
SPRR3	7	0	2	0	0	0
	0	0	2	0	1	0
SPRR4	6	0	4	0	1	0
	0	0	1	0	0	0
SPRTN	54	105	35	112	76	98
	135	200	102	57	110	32
SPRY1	59	339	79	281	145	290
	272	312	162	106	154	30
SPRY2	39	153	42	154	85	152
	154	183	79	50	79	36

SPRY3 (NC_000023 154997450..155012117)			18	29	20	
	21	8	30	18	18	21
	10	13	6			
SPRY3 (NC_000024 59100456..59115123)			15	30	19	
	30	25	33	15	38	17
	9	24	2			
SPRY4	31	106	25	70	31	57
	64	62	35	12	32	21
SPRYD3	20	43	16	53	28	36
	41	47	28	17	27	9
SPRYD4	33	79	18	50	23	41
	50	64	30	18	37	10
SPRYD7	53	345	55	185	134	239
	269	348	155	79	157	42
SPSB1	22	27	12	31	48	21
	55	89	32	24	49	33
SPSB2	4	5	3	0	2	4
	0	3	2	1	3	0
SPSB3	21	78	13	57	33	75
	93	174	78	44	72	24
SPSB4	9	3	10	13	2	8
	1	12	10	0	8	1
SPTA1	91	19	81	1	45	7
	1	3	66	0	35	2
SPTAN1	198	1332	355	1143	586	838
	792	1034	507	360	563	292
SPTB	737	4528	1355	3064	1768	3265
	2013	5661	1870	1443	2015	779
SPTBN1	1117	6576	1130	4397	3202	4225
	3851	5352	2337	2189	3084	1597
SPTBN2	39	3	15	4	7	4
	3	2	24	1	9	1
SPTBN4	55	58	61	21	37	35
	62	94	49	11	50	16
SPTBN5	40	16	37	5	14	8
	7	21	25	7	12	4
SPTLC1	52	157	51	155	85	150
	151	245	124	62	141	57
SPTLC2	85	301	66	280	139	208
	199	334	170	81	156	48
SPTLC3	29	23	19	23	17	22
	11	31	36	6	29	7
SPTSSA	20	158	29	121	73	134
	113	163	63	30	82	22
SPTSSB	19	2	10	0	2	3
	2	0	9	0	3	0
SPTY2D1	100	301	114	242	165	189
	233	301	125	99	182	58
SPZ1	13	1	10	0	5	0
	0	0	6	0	3	0
SQLE	33	27	11	24	16	20
	11	17	32	4	26	4
SQRDL	60	366	107	294	147	388
	381	465	239	101	305	53
SQSTM1	688	2685	540	2002	1374	2317
	2559	4895	1873	927	2207	495
SRA1	18	137	23	85	37	70
	77	178	60	22	77	13

SRBD1	60	159	68	111	104	116
	91	147	102	41	89	20
SRC	15	13	11	13	12	9
	5	13	6	1	8	5
SRCAP	138	512	124	324	247	312
	454	443	163	169	252	108
SRCIN1	18	0	19	0	8	0
	1	0	18	2	7	0
SRCRB4D	13	0	9	3	1	3
	3	3	7	0	1	0
SRD5A1	12	18	11	12	11	13
	11	18	10	3	28	1
SRD5A2	16	1	13	3	5	0
	0	12	11	0	3	0
SRD5A3	26	60	18	64	33	32
	26	30	36	11	30	6
SREBF1	45	114	34	84	114	77
	107	252	56	29	65	43
SREBF2	65	212	44	151	124	169
	200	168	103	75	125	30
SREK1	263	1104	263	819	517	903
	892	1515	581	449	661	208
SREK1IP1	67	301	70	241	158	246
	231	283	125	73	177	67
SRF	69	346	43	207	122	242
	227	303	111	91	148	55
SRFBP1	45	187	58	118	109	125
	143	247	105	80	126	29
SRGAP1	76	43	45	52	40	32
	32	54	42	14	51	9
SRGAP2	83	214	107	122	91	98
	82	160	97	62	112	26
SRGAP3	84	92	64	50	49	59
	42	108	84	14	51	16
SRGAP3-AS3	5	0	0	0	2	0
	0	2	3	0	1	0
SRGN	12	92	12	68	25	35
	91	152	54	18	55	16
SRI	43	162	27	164	67	143
	142	238	101	69	90	40
SRL	742	3785	712	3165	1768	2974
	3243	4214	1912	1311	1943	605
SRM	11	83	16	51	20	47
	84	67	41	25	35	15
SRMS	5	0	1	0	0	0
	0	0	3	0	1	0
SRP14	267	1447	186	1094	592	1043
	1798	2108	734	437	883	238
SRP19	58	201	43	139	110	136
	187	282	98	56	115	36
SRP54	97	562	138	351	298	365
	380	619	264	179	374	94
SRP68	92	529	80	306	223	259
	392	351	135	97	172	60
SRP72	115	549	145	371	264	305
	414	499	228	164	266	95
SRP9	335	1948	321	1361	796	1523
	1606	3007	874	485	1170	290

SRPK1	98	354	65	230	164	253
	255	441	211	122	212	84
SRPK2	92	377	77	281	154	321
	284	417	194	130	179	73
SRPK3	195	793	190	537	312	738
	658	754	426	247	500	160
SRPR	81	464	54	427	166	363
	414	361	159	121	178	72
SRPRB	44	156	34	111	57	116
	105	166	75	42	62	29
SRPX	21	35	17	31	18	30
	12	48	33	16	12	6
SRPX2	38	33	10	60	40	48
	26	74	37	20	17	18
SRR	26	35	15	36	27	53
	40	59	26	13	29	12
SRRD	32	147	29	165	45	96
	112	150	99	44	92	15
SRRM1	168	897	175	503	353	506
	712	815	265	264	426	163
SRRM2	989	7958	1072	3328	3222	3522
	5691	4514	1307	2272	2314	1318
SRRM3	8	1	4	0	5	0
	0	0	0	0	5	0
SRRM4	49	5	23	6	7	3
	2	6	19	2	12	0
SRRM5	45	15	26	3	10	6
	6	6	21	4	8	3
SRRT	52	261	57	131	95	137
	172	195	73	69	109	37
SRSF1	428	2174	479	1422	1024	1621
	1628	3167	901	682	1378	467
SRSF10	140	647	141	376	307	417
	384	863	298	189	482	117
SRSF11	272	1095	277	976	595	1126
	955	1876	686	492	875	299
SRSF12	21	8	15	4	6	9
	3	2	15	1	11	2
SRSF2	161	708	181	532	290	685
	619	948	441	228	501	128
SRSF3	147	810	163	596	376	608
	741	1012	359	243	526	142
SRSF4	85	441	63	261	212	303
	385	385	145	170	260	83
SRSF5	299	1498	345	1226	778	1606
	1346	1998	881	554	1145	285
SRSF6	215	1196	255	695	425	847
	922	1262	455	331	672	239
SRSF7	126	538	101	395	254	369
	469	742	231	161	353	80
SRSF8	66	212	57	170	120	188
	149	225	108	66	123	40
SRSF9	23	115	22	97	48	69
	95	180	48	31	44	20
SRXN1	23	116	26	57	65	59
	28	83	43	26	44	22
SRY	0	0	2	0	0	0
	0	0	1	0	0	0

SS18	69	276	69	220	146	173
	216	322	144	86	156	47
SS18L1	86	269	71	217	102	247
	263	253	101	70	140	47
SS18L2	7	29	4	14	9	15
	18	45	23	4	13	6
SSB	106	591	107	344	280	440
	457	564	206	170	313	106
SSBP1	91	414	91	316	132	282
	494	624	248	115	294	75
SSBP2	116	546	135	386	281	295
	285	458	194	122	225	64
SSBP3	34	69	20	51	47	48
	42	67	37	19	43	8
SSBP4	10	24	9	24	19	32
	19	43	24	7	23	10
SSC5D	32	45	23	35	29	27
	12	37	19	12	16	8
SSFA2	274	1156	269	807	631	800
	981	1340	601	433	723	219
SSH1	103	342	108	301	164	456
	284	227	138	87	171	79
SSH2	478	1942	485	1701	1220	1650
	1658	2407	884	732	1306	430
SSH3	10	24	6	16	16	18
	11	36	15	5	9	6
SSNA1	14	51	9	37	22	50
	54	63	29	12	25	12
SSPN	429	2031	425	922	798	1803
	1260	2236	970	660	658	410
SSPO	35	4	39	4	11	3
	2	4	27	1	17	2
SSR1	140	722	162	522	282	477
	520	620	350	187	369	131
SSR2	21	103	24	108	59	115
	95	154	81	42	64	19
SSR3	181	1181	239	931	469	952
	853	1223	574	335	633	164
SSR4	21	46	6	79	12	57
	55	81	37	17	33	7
SSRP1	81	326	73	208	163	268
	271	347	129	145	188	68
SSSCA1	8	28	0	21	8	21
	58	55	16	7	32	5
SST	2	0	0	0	1	0
	0	0	1	0	0	0
SSTR1	7	4	6	3	6	4
	2	1	8	0	2	0
SSTR2	14	3	10	2	9	1
	3	6	3	3	1	1
SSTR3	8	0	4	0	1	0
	0	0	1	0	0	0
SSTR4	1	0	2	0	0	0
	0	0	0	0	0	0
SSTR5	4	0	3	0	0	0
	0	0	2	0	3	0
SSU72	90	505	45	361	216	379
	651	475	234	137	251	89

SSX1	22	0	3	0	2	0
	0	1	5	0	1	0
SSX2	15	0	3	0	3	0
	0	0	1	0	1	0
SSX2B	13	0	10	0	3	0
	0	0	11	0	2	0
SSX2IP	50	142	66	95	73	92
	72	58	53	18	38	16
SSX3	18	0	5	0	1	0
	0	0	2	0	0	0
SSX4	5	0	5	0	6	0
	0	0	2	0	2	0
SSX4B	26	0	2	0	1	0
	0	0	0	0	4	0
SSX5	7	0	6	0	3	0
	0	0	8	0	1	0
SSX7	27	0	7	0	5	0
	0	0	6	0	1	0
ST13	531	3186	512	1899	1532	2542
	2357	3351	1299	976	1666	584
ST14	23	1	10	0	3	0
	0	0	7	0	4	0
ST18	52	3	51	4	19	0
	1	0	34	0	23	0
ST20	5	15	4	16	2	17
	13	16	14	0	10	1
ST20-MTHFS	13	34	10	16	11	21
	18	26	10	7	7	2
ST3GAL1	218	758	134	484	364	556
	787	1005	413	292	673	218
ST3GAL2	60	304	66	271	191	245
	178	220	111	57	120	20
ST3GAL3	49	250	50	153	75	217
	193	276	146	117	173	60
ST3GAL4	11	27	7	19	11	11
	15	19	17	5	10	1
ST3GAL5	35	149	34	195	69	175
	132	153	56	48	73	23
ST3GAL6	55	135	29	42	112	81
	69	354	54	82	72	32
ST5	61	93	44	86	70	82
	81	106	49	41	77	28
ST6GAL1	72	290	46	129	130	266
	200	284	118	61	114	40
ST6GAL2	39	1	20	0	5	1
	0	0	22	0	8	0
ST6GALNAC1	11	8	7	4	4	8
	10	8	10	4	11	3
ST6GALNAC2	41	94	11	55	52	71
	102	165	38	44	71	25
ST6GALNAC3	23	32	14	8	16	19
	26	35	23	10	24	8
ST6GALNAC4	9	23	6	13	7	32
	26	30	17	7	17	7
ST6GALNAC5	12	0	6	1	3	0
	0	1	8	1	5	0
ST6GALNAC6	32	150	26	68	70	75
	95	188	77	40	90	35

ST7	91	161	61	93	76	106
	96	223	98	66	111	29
ST7L	78	251	86	169	121	168
	211	267	134	86	170	49
ST8SIA1	56	29	40	39	22	31
	18	21	49	12	25	3
ST8SIA2	28	8	28	5	7	5
	0	1	10	1	8	1
ST8SIA3	62	0	36	1	11	0
	0	1	36	0	13	0
ST8SIA4	43	108	34	85	55	56
	61	87	81	43	54	20
ST8SIA5	20	19	10	12	4	11
	28	13	18	3	18	3
ST8SIA6	14	8	8	4	3	9
	2	10	6	4	4	5
STAB1	66	138	52	122	121	96
	94	373	73	46	107	73
STAB2	83	17	52	9	15	0
	0	3	53	3	19	0
STAC	18	13	11	13	8	3
	7	16	9	0	10	0
STAC2	14	2	8	4	2	0
	0	0	3	0	4	0
STAC3	1110	6687	1490	4445	3643	4946
	5028	5090	2234	1939	3362	1141
STAG1	213	1005	293	808	563	811
	679	1026	511	327	580	175
STAG2	312	1411	373	1232	764	1150
	1170	1645	670	507	860	314
STAG3	85	86	32	51	40	47
	43	95	67	24	51	21
STAM	79	309	83	227	164	190
	300	385	185	91	216	62
STAM2	122	438	91	274	252	309
	306	579	206	135	234	84
STAMBP	123	379	97	318	223	287
	358	510	225	119	269	77
STAMBPL1	28	39	14	37	21	29
	18	10	23	13	20	4
STAP1	22	1	15	2	8	2
	0	3	9	0	3	0
STAP2	8	7	5	1	3	0
	7	0	2	0	1	0
STAR	11	0	11	1	0	2
	2	3	11	0	4	0
STARD10	43	129	15	122	50	167
	128	149	79	38	112	16
STARD13	87	195	76	222	88	118
	151	212	107	71	119	46
STARD3	7	77	29	32	29	38
	52	67	32	24	32	11
STARD3NL	53	143	23	128	76	163
	119	221	79	39	78	17
STARD4	22	27	13	21	20	19
	13	15	15	4	10	3
STARD5	8	5	1	2	1	2
	6	2	8	1	4	1

STARD6	8	0	3	0	2	0
	0	0	3	0	1	0
STARD7	629	2808	709	2702	1514	2350
	2374	2875	1386	716	1548	521
STARD8	29	80	17	64	48	41
	60	74	56	20	51	15
STARD9	186	590	120	461	268	357
	364	486	263	161	270	146
STAT1	127	582	111	439	289	323
	412	481	246	179	361	79
STAT2	118	359	77	227	146	208
	225	392	158	126	167	72
STAT3	253	1440	206	680	576	499
	828	744	436	379	470	200
STAT4	41	12	18	17	12	16
	11	31	34	17	31	6
STAT5A	41	92	33	81	65	97
	100	168	90	47	97	29
STAT5B	150	668	145	568	420	608
	617	670	364	207	454	131
STAT6	64	129	45	94	74	123
	92	171	67	37	75	17
STATH	7	0	3	1	2	0
	0	0	3	0	0	0
STAU1	144	858	125	491	362	478
	597	602	292	218	328	131
STAU2	237	1240	227	838	547	819
	1080	1230	591	306	696	232
STBD1	9	591	84	496	206	495
	933	530	203	159	252	83
STC1	20	3	20	2	4	4
	1	14	6	1	13	3
STC2	30	21	17	22	8	29
	8	4	18	3	14	2
STEAP1	8	6	4	8	4	8
	1	11	7	4	6	3
STEAP1B	7	26	13	22	15	19
	16	21	12	8	22	2
STEAP2	33	28	35	41	23	68
	31	36	33	12	23	15
STEAP3	86	380	96	268	231	325
	234	273	143	66	136	49
STEAP4	64	505	63	274	165	193
	263	391	129	111	229	59
STIL	33	9	29	15	11	14
	13	12	37	2	23	5
STIM1	170	1026	217	712	494	759
	710	881	390	258	501	155
STIM2	63	264	93	212	122	171
	177	241	138	68	143	48
STIP1	153	868	165	446	486	603
	478	702	283	223	385	123
STK10	48	104	32	57	41	59
	67	69	53	22	44	12
STK11	58	306	47	209	146	181
	162	352	147	101	156	66
STK11IP	26	21	13	24	22	19
	15	23	14	3	17	0

STK16	15	42	18	19	21	23
	26	45	17	11	18	6
STK17A	40	91	32	86	85	95
	81	160	57	57	75	24
STK17B	37	87	38	90	62	108
	112	161	91	36	71	28
STK19	16	18	10	14	8	10
	17	32	17	8	17	4
STK24	70	337	90	253	176	226
	263	431	180	113	181	71
STK25	262	1367	174	967	504	1044
	1002	1046	481	374	532	190
STK3	73	312	87	239	107	201
	238	360	145	92	168	43
STK31	34	5	28	3	13	3
	5	5	37	0	25	1
STK32A	16	3	26	10	9	3
	6	8	10	1	18	2
STK32B	18	32	12	10	16	28
	14	28	13	3	13	1
STK32C	7	8	3	3	2	3
	0	1	4	1	1	0
STK33	32	58	22	35	22	34
	43	78	58	27	50	17
STK35	50	137	23	122	69	72
	166	206	86	39	72	28
STK36	66	68	36	33	49	71
	52	119	64	31	61	17
STK38	65	287	66	191	108	258
	219	236	121	65	109	42
STK38L	62	295	85	270	165	221
	219	341	213	84	153	43
STK39	39	75	36	65	29	50
	78	76	50	20	42	14
STK4	59	147	59	132	102	130
	127	171	90	58	121	36
STK40	77	380	60	244	189	292
	239	286	121	106	126	65
STMN1	36	116	26	58	54	60
	119	151	48	17	46	15
STMN2	17	1	12	1	3	0
	0	0	8	0	3	0
STMN3	9	13	4	10	2	4
	4	10	10	2	7	1
STMN4	8	1	11	0	7	0
	0	0	5	0	7	1
STOM	108	793	96	507	336	379
	615	838	379	252	457	128
STOML1	20	27	16	13	15	17
	26	38	21	9	21	6
STOML2	61	273	42	173	123	200
	292	409	176	78	190	27
STOML3	12	0	4	0	3	0
	0	0	5	0	3	0
STON1	0	3	2	6	1	2
	10	18	5	4	3	4

STON1-GTF2A1L	37	47	28	33	32	35	32	54	38	10	35	8
STON2	293	1325	354	1005	463	875	650	979	515	398	512	200
STOX1	17	11	12	8	9	17	11	20	14	8	14	4
STOX2	24	81	30	51	27	53	62	38	42	17	30	12
STPG1	25	16	12	6	11	10	10	17	16	3	14	2
STPG2	8	5	7	1	8	0	5	1	9	0	6	0
STRA13	3	18	7	16	6	10	16	33	5	6	11	4
STRA6	24	0	18	0	6	0	0	0	12	0	6	0
STRA8	17	0	5	2	2	0	0	0	7	0	3	0
STRADA	22	67	24	57	29	56	75	83	40	29	51	21
STRADB	160	769	139	695	330	618	778	789	382	189	394	109
STRAP	97	706	109	558	277	484	607	694	274	197	324	103
STRBP	55	235	76	160	95	135	138	240	122	62	109	29
STRC	64	4	30	7	8	6	2	3	24	0	19	2
STRN	190	596	164	478	328	461	459	697	315	193	353	134
STRN3	225	1408	320	1082	739	1032	984	1749	569	478	905	304
STRN4	46	160	29	108	65	137	133	201	78	52	105	44
STS	75	118	24	80	64	40	95	82	56	46	64	28
STT3A	97	467	122	366	180	311	300	524	174	111	204	72
STT3B	213	1186	192	884	579	895	868	1499	559	387	644	198
STUB1	105	571	98	507	239	501	576	833	310	169	309	101
STX10	12	35	13	28	20	38	29	48	19	8	21	1
STX11	27	12	12	8	5	8	13	16	16	7	10	3
STX12	47	262	60	211	115	182	166	285	100	72	140	49
STX16	74	481	89	319	213	311	275	434	176	132	215	96
STX17	141	596	161	500	280	430	495	599	279	189	370	125
STX18	43	180	44	145	90	137	139	210	83	57	104	28
STX19	0	1	1	0	3	1	0	0	1	0	2	0

STX1A	8	2	9	1	2	2
	1	2	0	0	5	0
STX1B	22	3	7	2	2	0
	0	2	2	1	4	0
STX2	37	80	16	68	54	72
	58	77	43	25	28	9
STX3	48	147	43	98	59	80
	120	142	85	46	104	26
STX4	26	133	22	89	51	63
	113	99	41	23	50	15
STX5	25	60	22	44	33	31
	35	77	43	22	56	17
STX6	73	217	76	182	104	127
	183	269	141	56	166	51
STX7	133	518	116	401	240	408
	442	663	291	149	303	108
STX8	44	230	31	103	74	152
	189	279	118	86	158	30
STXBP1	40	76	27	44	27	45
	46	50	21	20	33	15
STXBP2	3	6	5	3	6	1
	1	2	2	0	3	0
STXBP3	75	368	103	338	219	362
	292	408	229	116	237	79
STXBP4	53	73	39	48	42	59
	83	80	46	24	46	21
STXBP5	236	1031	325	943	380	979
	610	860	472	256	524	150
STXBP5L	68	0	33	1	20	3
	0	2	42	0	25	0
STXBP6	21	32	9	33	19	15
	23	54	20	16	24	10
STYK1	24	2	14	2	6	3
	0	4	10	1	6	0
STYX	119	684	114	445	270	424
	529	595	238	123	363	145
STYXL1	16	20	14	19	15	15
	12	32	20	5	17	11
SUB1	254	1312	255	1013	640	921
	1290	1840	658	382	875	203
SUCLA2	391	2397	335	1749	941	1551
	2261	2360	1078	744	1418	384
SUCLG1	222	1308	178	980	485	787
	1542	1806	749	356	799	176
SUCLG2	283	1219	227	949	562	1007
	1307	1686	697	402	796	217
SUCNR1	12	2	9	2	6	2
	1	1	8	0	2	0
SUCO	96	287	112	197	150	230
	231	248	172	62	171	72
SUDS3	153	499	155	540	283	469
	420	664	249	207	319	125
SUFU	40	75	26	66	34	59
	55	51	40	32	40	10
SUGP1	31	92	27	50	46	59
	92	101	47	32	62	18
SUGP2	137	767	160	482	295	583
	458	708	338	218	333	126

SUGT1	85	345	63	193	144	221
	210	317	156	94	192	48
SULF1	55	110	54	87	54	26
	74	143	53	41	57	78
SULF2	47	130	44	96	40	94
	90	151	65	33	82	34
SULT1A1	21	45	19	24	10	37
	29	33	32	19	25	10
SULT1A2	6	6	2	5	2	1
	2	6	5	2	2	3
SULT1A3	7	11	10	16	16	15
	10	45	9	6	19	5
SULT1A4	9	21	9	16	11	27
	14	25	10	8	18	6
SULT1B1	12	3	2	1	1	1
	4	1	10	0	2	0
SULT1C2	18	1	11	2	3	0
	0	0	8	0	10	0
SULT1C3	13	0	12	0	3	0
	0	0	8	0	3	0
SULT1C4	24	16	11	7	4	6
	17	25	10	5	13	6
SULT1E1	13	0	5	0	7	0
	0	0	9	0	2	0
SULT2A1	19	0	6	0	2	1
	0	1	11	0	1	0
SULT2B1	7	0	2	0	2	0
	0	0	3	0	1	0
SULT4A1	11	0	7	0	2	0
	0	1	3	1	5	0
SULT6B1	9	0	3	0	2	0
	0	0	5	0	1	0
SUMF1	25	83	32	56	38	63
	66	118	56	25	61	11
SUMF2	39	78	27	79	34	83
	57	107	34	20	44	23
SUMO1	135	781	150	564	343	632
	786	894	408	202	515	123
SUMO2	162	723	132	496	382	597
	811	1121	471	217	479	109
SUMO3	69	306	32	172	142	190
	309	312	117	77	165	39
SUN1	333	1513	309	1251	884	1005
	1328	1791	836	470	988	274
SUN2	82	258	72	214	148	172
	198	542	185	71	186	68
SUN3	10	0	8	1	2	1
	0	0	7	0	4	0
SUN5	9	0	7	0	3	0
	0	0	5	0	2	0
SUOX	73	317	55	165	145	157
	246	281	170	67	162	33
SUPT16H	143	516	123	390	251	380
	428	602	259	204	337	108
SUPT3H	37	78	32	35	28	49
	49	58	34	17	35	10
SUPT4H1	105	397	75	279	182	345
	392	641	228	110	271	52

SUPT5H	90	319	96	214	128	201
	167	267	146	92	148	70
SUPT6H	190	863	188	604	343	491
	611	698	350	233	477	166
SUPT7L	92	402	66	291	195	309
	335	470	239	136	270	72
SUPV3L1	118	459	82	292	181	298
	518	533	214	148	326	76
SURF1	8	37	7	38	13	24
	28	38	20	9	22	3
SURF2	8	31	4	10	10	35
	24	26	10	11	23	4
SURF4	51	372	65	309	167	313
	208	426	180	90	222	74
SURF6	30	140	26	93	41	95
	96	122	57	56	78	25
SUSD1	36	45	21	44	29	34
	33	53	39	17	29	10
SUSD2	18	13	15	13	7	9
	3	20	7	6	8	3
SUSD3	3	2	4	0	2	1
	0	0	0	0	0	0
SUSD4	20	18	29	11	16	15
	10	4	14	0	4	0
SUSD5	28	27	24	22	10	84
	17	30	23	6	22	3
SUV39H1	13	25	10	21	6	10
	16	27	18	8	12	5
SUV39H2	37	70	41	48	41	58
	48	81	48	16	57	21
SUV420H1	105	666	153	507	338	453
	481	597	242	190	319	98
SUV420H2	17	19	0	10	9	8
	15	19	12	2	8	1
SUZ12	86	317	81	208	172	211
	222	356	130	95	182	53
SV2A	24	5	26	3	5	4
	0	5	12	1	4	1
SV2B	88	42	53	53	35	38
	43	70	59	13	70	15
SV2C	16	2	22	1	5	0
	0	0	12	0	7	1
SVEP1	170	388	220	406	265	369
	261	584	246	119	262	156
SVIL	4116	26265	3752	17601	12263	16125
	16234	19837	8216	8605	13081	5159
SVIP	26	74	31	121	63	126
	103	176	75	41	117	28
SVOP	12	3	10	1	7	2
	0	1	4	0	5	0
SVOPL	28	0	6	0	8	0
	0	0	11	0	4	0
SWAP70	64	222	59	162	104	175
	175	270	132	87	143	65
SWI5	7	33	13	23	8	19
	35	33	17	7	27	4
SWSAP1	6	11	3	7	4	6
	6	10	7	4	6	0

SWT1	58	188	58	142	91	160
	175	181	99	62	104	28
SYAP1	89	451	81	310	304	327
	387	467	188	172	244	70
SYBU	22	22	17	15	11	22
	17	24	35	11	14	4
SYCE1	10	1	9	0	4	5
	0	2	5	1	5	1
SYCE1L	4	1	1	3	1	5
	10	4	3	0	4	1
SYCE2	6	0	6	0	1	0
	0	1	1	0	1	0
SYCE3	8	0	3	0	4	0
	0	0	1	0	3	0
SYCP1	24	1	29	0	17	0
	0	0	37	0	14	0
SYCP2	53	57	53	66	46	56
	81	57	66	24	46	11
SYCP2L	55	13	19	28	23	14
	22	20	32	6	10	2
SYCP3	10	8	11	7	3	16
	8	11	8	5	8	1
SYDE1	10	14	4	13	8	9
	8	17	10	7	15	5
SYDE2	32	99	26	80	54	84
	70	65	36	23	43	9
SYF2	76	291	97	260	168	286
	313	398	205	148	240	101
SYK	52	45	21	40	33	42
	32	67	49	11	30	11
SYMPK	53	200	50	98	91	106
	108	198	65	52	77	42
SYN1	20	4	5	0	3	2
	0	0	4	0	1	0
SYN2	46	14	28	18	7	14
	24	12	42	5	19	2
SYN3	30	3	10	0	2	0
	0	2	9	0	5	0
SYNC	250	1687	409	1237	663	1222
	1038	978	541	458	624	243
SYNCRIP	358	2278	419	1634	1029	1503
	1508	2177	780	592	1165	372
SYNDIG1	6	0	4	0	1	0
	0	0	3	0	0	0
SYNDIG1L	10	0	7	0	0	0
	0	0	3	0	3	0
SYNE1	904	4834	1504	2933	2045	3182
	2908	3792	1878	1498	2505	812
SYNE2	1148	5594	1209	3310	3317	3312
	3929	5243	2424	1986	3087	1198
SYNE3	23	84	34	98	45	67
	37	91	50	22	29	28
SYNE4	9	0	4	0	0	0
	0	1	1	0	1	0
SYNGAP1	31	33	19	16	13	14
	12	13	28	2	6	2
SYNGR1	31	64	31	64	40	48
	56	57	22	19	44	14

SYNGR2	93	436	64	387	209	300
	575	575	300	182	309	95
SYNGR3	4	0	0	0	0	0
	0	0	1	0	0	0
SYNGR4	1	0	1	0	0	0
	0	0	0	0	2	0
SYNJ1	80	167	56	143	87	118
	107	146	95	62	110	20
SYNJ2	65	155	49	87	111	174
	98	79	59	58	64	26
SYNJ2BP	8	184	10	94	77	54
	81	136	46	86	120	58
SYNJ2BP-COX16		46	97	37	60	50
	99	98	119	41	31	62
	17					
SYNM	2042	15659	1872	9499	7115	7803
	8407	9246	3753	3543	5685	2155
SYNPO	975	4723	480	2704	1812	3531
	3799	3788	1727	1886	2808	938
SYNPO2	4549	22777	3409	11734	13260	13865
	13568	26927	8528	8446	11234	4441
SYNPO2L	244	2361	351	1396	821	1294
	1322	1204	396	497	659	266
SYNPR	14	0	8	0	8	0
	0	0	1	0	3	0
SYNRG	95	394	110	274	192	245
	294	427	185	118	194	67
SYP	15	10	7	3	4	2
	5	5	6	5	8	2
SYPL1	157	723	116	827	355	580
	709	901	322	218	409	135
SYPL2	297	1999	246	1041	628	1379
	1811	1281	616	393	742	179
SYS1	12	50	5	40	41	76
	61	48	25	12	17	5
SYT1	28	3	26	4	13	0
	5	3	35	0	10	2
SYT10	21	0	18	1	9	0
	0	1	15	0	8	0
SYT11	31	50	20	26	32	42
	35	72	40	13	26	15
SYT12	15	4	9	0	1	0
	2	2	7	1	8	0
SYT13	27	1	26	1	11	1
	1	0	20	0	9	0
SYT14	29	2	18	4	9	3
	3	0	20	1	9	0
SYT15	96	147	39	51	38	43
	59	94	67	24	42	21
SYT16	21	0	13	0	7	0
	0	0	8	0	4	0
SYT17	16	2	7	7	4	6
	5	2	10	2	5	2
SYT2	44	7	21	1	18	3
	0	6	17	0	16	1
SYT3	26	0	9	0	1	1
	0	0	6	0	5	0

SYT4	15	0	12	0	6	0
	0	0	11	0	7	0
SYT5	7	0	4	0	0	0
	0	0	3	0	0	0
SYT6	17	6	15	6	9	3
	12	3	14	1	4	1
SYT7	34	29	11	10	13	11
	13	25	17	6	11	1
SYT8	5	0	0	0	0	0
	0	0	3	0	1	0
SYT9	25	2	15	0	4	3
	1	2	11	0	5	1
SYTL1	10	3	2	1	1	0
	0	1	2	1	3	1
SYTL2	90	289	72	111	79	154
	126	147	71	74	95	39
SYTL3	61	269	75	169	102	231
	182	229	162	70	124	46
SYTL4	44	44	23	52	39	47
	81	102	37	23	26	21
SYTL5	32	0	13	0	13	1
	0	0	18	0	5	0
SYVN1	14	72	13	69	24	51
	60	76	26	28	27	19
SZRD1	70	310	54	263	127	205
	260	148	102	74	117	45
SZT2	190	438	149	237	191	282
	340	441	221	136	265	79
T	6	0	6	0	2	0
	0	1	8	0	4	0
TAAR1	9	0	3	0	0	0
	0	0	3	0	2	0
TAAR2	7	0	2	0	0	0
	0	0	2	0	2	0
TAAR5	8	0	0	0	1	0
	0	0	1	0	0	0
TAAR6	4	0	1	0	0	0
	0	0	3	0	2	0
TAAR8	6	0	4	0	2	0
	0	0	5	0	3	0
TAAR9	8	0	2	0	2	0
	0	0	5	0	0	0
TAB1	43	108	33	77	60	80
	69	90	45	43	59	20
TAB2	206	1123	161	970	416	776
	866	893	347	261	455	167
TAB3	85	408	94	277	148	268
	292	350	146	64	169	67
TAC1	9	0	6	1	3	0
	0	0	7	0	4	0
TAC3	7	0	4	0	1	0
	0	1	1	0	4	0
TAC4	6	1	2	2	1	0
	0	2	1	0	3	1
TACC1	178	814	159	655	449	543
	811	1197	530	320	608	253
TACC2	1082	5291	770	2827	2574	3761
	4579	5078	1934	2162	3011	1126

TACC3	9	12	5	7	11	8
	16	10	10	4	4	4
TACO1	36	212	27	96	62	120
	188	229	85	54	117	38
TACR1	20	6	14	4	8	1
	1	1	10	0	5	0
TACR2	37	8	18	3	6	6
	0	3	10	1	10	0
TACR3	10	3	2	0	7	2
	0	1	7	2	2	0
TACSTD2	4	6	5	1	3	0
	2	0	2	0	2	0
TADA1	31	112	28	88	52	91
	95	133	76	42	81	16
TADA2A	57	141	50	73	69	85
	91	145	50	47	84	25
TADA2B	94	381	75	357	157	236
	335	284	155	95	242	71
TADA3	56	212	38	126	92	139
	142	172	73	51	83	24
TAF1	224	904	260	550	394	495
	579	937	331	256	430	188
TAF10	25	127	19	96	49	110
	141	163	88	34	66	24
TAF11	54	178	22	125	94	133
	155	216	88	67	130	37
TAF12	54	124	24	89	50	117
	74	178	67	46	69	21
TAF13	50	263	36	156	100	118
	165	152	74	43	101	29
TAF15	72	258	54	157	130	97
	153	271	96	80	121	60
TAF1A	24	33	24	19	30	26
	32	21	23	8	22	3
TAF1B	24	58	22	58	29	51
	28	74	31	13	27	15
TAF1C	21	66	27	64	27	46
	77	79	36	21	57	13
TAF1D	30	159	36	109	51	80
	147	139	65	53	77	20
TAF1L	34	1	21	4	6	0
	1	0	20	0	12	0
TAF2	59	268	83	198	131	196
	219	312	160	100	220	59
TAF3	26	86	30	38	50	60
	61	70	30	26	38	19
TAF4	39	160	33	76	62	74
	101	127	46	44	73	17
TAF4B	56	88	36	58	60	75
	67	141	57	28	83	27
TAF5	34	52	27	44	37	42
	33	64	31	22	37	12
TAF5L	72	129	70	108	77	114
	150	147	81	40	84	29
TAF6	45	117	26	73	52	81
	82	94	40	28	56	21
TAF6L	21	48	15	27	26	38
	45	69	47	12	27	9

TAF7	207	1379	242	920	813	929
	1174	1426	526	468	778	223
TAF7L	19	3	9	2	6	0
	2	3	6	0	2	0
TAF8	64	161	52	116	108	143
	146	202	88	69	103	36
TAF9	105	506	101	336	243	407
	565	788	252	163	341	95
TAF9B	43	204	34	187	82	197
	163	231	106	77	124	48
TAGAP	18	22	15	22	6	14
	1	6	13	3	13	4
TAGLN	41	280	41	206	70	116
	86	281	94	31	100	121
TAGLN2	39	188	20	129	93	92
	155	266	84	49	80	32
TAGLN3	11	0	7	0	0	1
	0	0	3	0	1	0
TAL1	16	30	15	27	18	15
	37	42	31	6	18	4
TAL2	7	17	5	14	4	13
	10	10	1	2	2	0
TALDO1	62	291	42	148	121	165
	240	329	143	61	155	43
TAMM41	41	106	22	56	30	49
	42	113	34	20	53	13
TANC1	83	256	83	174	78	97
	157	185	120	71	97	45
TANC2	135	159	81	149	134	144
	107	104	123	25	107	25
TANK	78	359	67	282	205	322
	361	535	215	140	292	75
TAOK1	416	2058	479	1592	1075	1503
	1368	1919	933	578	1095	383
TAOK2	68	237	71	156	118	141
	174	268	131	82	164	40
TAOK3	101	407	79	267	196	329
	270	365	141	138	170	77
TAP1	24	88	36	71	35	48
	82	90	58	42	70	22
TAP2	53	147	50	102	58	110
	111	156	80	64	106	29
TAPBP	58	280	40	174	90	148
	204	216	107	110	130	47
TAPBPL	22	44	9	63	30	37
	62	49	34	14	39	11
TAPT1	141	631	140	670	340	534
	522	711	361	188	424	129
TARBP1	118	502	119	366	200	354
	437	691	381	212	409	118
TARBP2	13	20	14	15	19	20
	30	36	27	9	17	2
TARDBP	150	1033	164	662	391	634
	803	788	318	296	425	177
TARM1	2	0	2	0	0	0
	0	0	2	0	0	0
TARP	4	1	2	1	0	4
	1	1	3	1	5	1

TARS	81	514	106	343	266	410
	432	442	273	97	283	100
TARS2	41	180	53	79	71	99
	115	196	87	34	92	42
TARSL2	173	760	194	593	522	591
	592	981	367	366	556	171
TAS1R1	7	1	3	1	1	0
	3	1	2	0	1	0
TAS1R2	11	0	2	0	2	0
	0	0	7	0	4	0
TAS1R3	3	0	1	0	0	0
	0	0	2	0	1	0
TAS2R1	6	0	3	0	2	0
	1	0	3	0	0	0
TAS2R10	6	1	6	3	6	8
	0	4	3	0	0	1
TAS2R13	2	4	0	1	1	2
	2	1	2	2	1	0
TAS2R14	0	3	2	1	2	4
	0	4	0	1	2	1
TAS2R16	11	0	3	0	0	0
	0	0	2	0	0	0
TAS2R19	3	2	2	2	4	6
	1	2	2	2	0	1
TAS2R20	3	2	3	3	2	2
	2	1	3	0	0	1
TAS2R3	6	20	13	14	11	15
	5	14	13	4	7	4
TAS2R30	2	4	1	1	0	4
	1	1	3	4	1	2
TAS2R31	1	1	2	2	1	0
	1	1	4	0	2	1
TAS2R38	10	0	5	0	4	0
	0	0	6	0	0	0
TAS2R39	7	0	2	0	4	0
	0	0	4	0	4	0
TAS2R4	8	15	8	15	13	19
	9	28	12	1	9	3
TAS2R40	4	0	5	0	2	0
	0	0	0	0	0	0
TAS2R41	0	1	2	0	0	0
	0	0	0	0	0	0
TAS2R42	2	0	3	0	2	0
	0	0	4	0	2	0
TAS2R43	0	1	1	2	0	0
	0	0	2	1	2	1
TAS2R46	0	1	1	1	0	0
	2	2	1	0	3	1
TAS2R5	5	11	2	12	5	12
	4	16	6	2	6	4
TAS2R50	2	0	0	1	2	1
	0	2	1	0	2	0
TAS2R60	2	0	0	0	0	0
	0	0	1	0	0	0
TAS2R7	9	0	2	0	3	4
	0	0	0	0	1	0
TAS2R8	4	0	0	1	2	2
	0	3	3	0	1	2

TAS2R9	5	0	2	0	5	6
	0	0	3	0	1	0
TASP1	34	50	20	38	38	40
	46	70	57	15	54	17
TAT	16	1	7	0	3	1
	0	0	7	0	6	0
TATDN1	65	213	34	148	101	138
	169	273	112	78	166	27
TATDN2	57	186	59	121	92	116
	142	157	82	55	103	39
TATDN3	68	306	67	244	139	198
	327	460	181	95	254	54
TAX1BP1	199	1517	224	930	626	894
	980	1302	491	405	670	222
TAX1BP3	14	42	13	44	15	38
	52	66	27	10	14	10
TAZ	19	71	8	59	27	40
	52	78	36	12	47	17
TBATA	13	0	9	0	1	0
	0	0	1	0	2	0
TBC1D1	208	671	96	1169	296	1173
	1012	436	323	291	397	90
TBC1D10A	8	7	5	6	7	2
	5	12	7	3	9	0
TBC1D10B	22	72	21	53	38	63
	60	87	45	20	37	18
TBC1D10C	9	1	4	2	0	0
	3	4	1	0	5	0
TBC1D12	57	152	55	178	98	161
	117	171	103	67	117	48
TBC1D13	47	105	33	74	58	90
	95	137	68	53	73	27
TBC1D14	137	484	104	325	203	341
	358	616	225	158	302	94
TBC1D15	85	404	86	355	209	276
	341	449	203	119	255	72
TBC1D16	30	61	14	40	30	31
	15	51	28	10	19	16
TBC1D17	24	62	16	65	28	37
	61	72	39	31	60	21
TBC1D19	36	84	26	73	38	50
	57	87	62	25	66	14
TBC1D2	13	22	12	26	9	12
	15	19	15	5	27	2
TBC1D20	69	231	43	183	94	195
	187	221	127	73	132	59
TBC1D21	9	0	1	0	2	0
	0	0	4	0	2	0
TBC1D22A	38	133	29	75	52	126
	98	122	84	35	63	23
TBC1D22B	43	91	39	64	44	64
	85	105	38	32	48	31
TBC1D23	165	788	243	548	428	549
	627	932	386	229	444	143
TBC1D24	30	91	30	96	69	71
	72	94	51	26	45	16
TBC1D25	19	44	18	44	21	33
	28	36	20	11	31	8

TBC1D26	19	0	13	0	4	0
	0	0	7	0	3	0
TBC1D27	7	0	6	0	0	0
	0	1	5	0	2	0
TBC1D28	9	0	10	0	3	0
	0	0	9	0	5	0
TBC1D29	3	1	4	3	1	4
	0	2	2	1	1	0
TBC1D2B	81	164	55	135	79	114
	101	154	88	51	93	43
TBC1D3	43	23	21	18	24	43
	13	59	29	72	44	12
TBC1D30	42	10	31	5	12	4
	4	10	17	5	14	1
TBC1D3B	30	6	14	2	2	7
	3	2	21	3	10	0
TBC1D3C	19	4	4	1	5	11
	4	13	5	5	13	2
TBC1D3F	65	97	82	82	51	60
	57	173	69	27	49	27
TBC1D3G	21	3	13	1	1	8
	6	3	5	0	9	3
TBC1D3H	17	4	8	6	2	3
	5	12	3	6	5	2
TBC1D4	739	3998	840	3590	2147	3238
	3269	5193	2153	946	2470	631
TBC1D5	144	416	133	371	196	372
	367	459	276	168	268	80
TBC1D7	17	50	22	24	21	21
	47	32	39	9	30	6
TBC1D8	195	445	107	334	257	474
	1033	1145	496	484	707	219
TBC1D8B	33	30	21	36	29	29
	22	29	22	10	29	1
TBC1D9	47	110	43	91	65	67
	88	111	68	33	63	28
TBC1D9B	86	310	73	266	164	230
	256	293	154	95	148	74
TBCA	40	196	35	143	95	114
	272	257	102	62	98	26
TBCB	46	235	49	140	85	179
	216	211	100	54	127	31
TBCC	31	135	14	105	79	101
	100	138	57	22	74	28
TBCCD1	30	87	34	61	53	68
	86	76	41	33	54	16
TBCD	70	180	61	134	92	138
	163	190	114	37	115	34
TBCE	57	229	34	126	104	132
	168	203	70	46	119	47
TBCEL	72	201	51	154	69	146
	160	262	123	50	137	45
TBCK	97	305	118	223	182	298
	229	370	177	90	175	52
TBK1	79	328	87	242	200	232
	277	470	200	100	240	80
TBKBP1	31	77	20	65	32	60
	69	73	26	12	38	12

TBL1X	51	84	28	56	42	68
	80	92	36	30	43	24
TBL1XR1	388	1792	389	1470	944	1405
	1319	2056	935	520	1129	319
TBL1Y	0	24	8	23	0	32
	12	10	24	0	2	2
TBL2	29	73	17	49	53	56
	95	92	44	27	64	24
TBL3	32	52	13	43	44	34
	31	73	32	26	40	11
TBP	32	79	21	55	49	64
	82	123	60	40	72	18
TBPL1	14	37	11	28	15	31
	42	52	29	23	18	8
TBPL2	9	0	3	0	3	0
	0	0	7	0	4	0
TBR1	16	0	5	0	1	0
	0	0	3	0	4	0
TBRG1	86	392	70	334	198	269
	347	468	201	106	206	82
TBRG4	60	178	30	91	80	98
	174	259	95	61	133	43
TBX1	23	140	12	35	37	46
	57	83	35	57	37	21
TBX10	2	0	5	1	1	0
	0	0	1	0	1	0
TBX15	253	1103	171	901	595	600
	1018	1012	480	342	569	180
TBX18	22	67	32	63	24	27
	40	28	39	12	42	11
TBX19	17	5	10	5	5	3
	3	2	7	1	10	0
TBX2	17	40	12	38	14	42
	37	42	22	12	17	13
TBX20	15	0	21	0	3	0
	0	0	14	0	11	0
TBX21	13	3	4	0	0	0
	1	3	7	1	3	0
TBX22	13	1	3	2	6	1
	0	1	7	0	2	0
TBX3	28	122	35	88	42	43
	110	92	56	34	60	8
TBX4	7	1	12	0	1	0
	0	0	9	0	4	0
TBX5	23	7	14	3	7	1
	2	9	13	1	10	1
TBX6	6	2	2	1	2	3
	1	4	5	0	2	0
TBXA2R	7	3	1	0	2	4
	1	2	3	1	3	0
TBXAS1	29	13	9	18	12	5
	8	27	18	4	19	2
TC2N	71	163	48	115	51	148
	120	201	82	30	106	25
TCAIM	157	778	154	467	337	463
	598	661	292	201	396	102
TCAP	1929	9191	1165	5052	4503	4934
	6812	11649	3750	2485	5370	1706

TCEA1	84	383	81	363	237	326
	299	437	263	156	270	93
TCEA2	11	26	11	17	14	19
	25	29	20	11	20	2
TCEA3	675	3490	800	2266	1636	3464
	3350	4399	1754	1460	2058	582
TCEAL1	59	211	52	134	107	172
	189	338	92	106	118	38
TCEAL2	9	0	2	1	7	2
	0	6	5	0	0	1
TCEAL3	10	34	20	13	20	20
	18	40	11	13	32	6
TCEAL4	37	211	34	94	78	165
	174	207	76	60	90	37
TCEAL5	6	1	3	1	5	0
	0	1	4	0	2	0
TCEAL6	8	1	6	0	0	0
	0	0	4	0	1	0
TCEAL7	11	24	7	13	9	12
	8	55	9	7	8	4
TCEAL8	29	146	19	77	77	89
	125	157	91	37	54	17
TCEANC	27	47	8	36	25	32
	42	55	22	19	35	11
TCEANC2	40	110	29	72	61	102
	75	110	58	40	52	24
TCEB1	62	312	46	325	125	295
	373	431	186	96	193	44
TCEB2	80	339	52	284	124	232
	434	630	230	83	199	38
TCEB3	140	769	142	575	349	594
	552	556	295	232	391	121
TCEB3B	12	2	6	4	4	3
	0	0	6	3	4	0
TCEB3C	17	0	9	0	6	1
	0	1	2	0	9	2
TCEB3CL	1	0	7	0	0	0
	0	0	10	0	4	0
TCERG1	161	922	229	601	426	685
	662	1009	455	329	584	177
TCERG1L	18	0	10	0	2	1
	0	2	6	0	1	0
TCF12	166	610	183	459	305	477
	408	668	318	166	344	147
TCF15	9	43	2	18	13	17
	62	45	16	2	11	6
TCF19	27	29	11	15	16	28
	27	25	28	11	23	5
TCF20	77	268	68	148	123	176
	191	258	117	88	122	49
TCF21	30	5	9	1	1	0
	2	5	10	3	14	0
TCF23	3	0	0	2	0	0
	0	0	0	1	0	0
TCF24	4	9	5	2	12	7
	4	8	5	1	8	0
TCF25	64	252	53	142	95	168
	197	253	76	100	120	45

TCF3	32	58	23	35	19	47
	49	48	23	20	29	9
TCF4	292	1465	380	1205	705	1069
	1219	1670	869	539	884	298
TCF7	19	13	14	4	5	8
	2	8	11	2	4	2
TCF7L1	13	11	7	6	11	5
	10	7	6	2	11	6
TCF7L2	45	118	39	101	52	70
	72	98	46	32	56	18
TCFL5	17	51	22	39	29	31
	34	33	34	18	39	19
TCHH	49	0	19	1	4	1
	1	3	22	0	11	0
TCHHL1	43	0	18	0	6	0
	0	0	12	0	6	0
TCHP	44	174	36	69	92	109
	120	218	101	52	83	33
TCIRG1	12	32	8	20	15	25
	17	32	16	2	13	7
TCL1A	3	1	2	0	0	0
	0	0	3	0	1	0
TCL1B	3	0	4	0	0	0
	1	0	1	0	3	0
TCN1	16	0	12	0	5	0
	0	0	11	0	3	0
TCN2	16	11	2	17	7	6
	20	30	16	8	18	6
TCOF1	67	106	51	91	88	100
	94	126	64	45	70	37
TCP1	245	1686	289	1014	659	1110
	1247	1723	723	410	874	230
TCP10	11	0	4	0	2	0
	0	0	2	0	1	0
TCP10L	11	9	7	8	5	9
	4	17	12	1	6	3
TCP10L2	24	1	14	0	3	1
	2	0	16	0	7	0
TCP11	15	0	14	0	2	1
	0	0	12	0	4	0
TCP11L1	30	79	32	56	35	67
	65	83	35	33	56	19
TCP11L2	88	427	78	264	237	203
	376	587	171	150	237	94
TCTA	18	76	21	58	38	48
	54	69	35	13	34	19
TCTE1	4	0	4	0	1	0
	0	1	3	0	2	0
TCTE3	4	2	4	2	1	6
	0	5	6	0	5	0
TCTEX1D1	9	10	5	7	6	5
	8	11	6	1	7	4
TCTEX1D2	6	12	7	8	3	3
	2	11	7	3	4	4
TCTEX1D4	5	0	2	0	0	0
	2	2	1	0	1	0
TCTN1	20	35	20	34	14	44
	23	66	34	17	20	5

TCTN2	26	23	13	20	12	6
	20	24	19	9	19	12
TCTN3	15	74	21	43	38	40
	36	93	28	22	46	9
TDG	68	148	50	156	97	101
	144	204	106	36	101	37
TDGF1	18	0	4	2	4	0
	5	0	10	3	8	1
TDO2	8	1	11	15	6	3
	0	0	12	1	4	1
TDP1	22	59	31	23	23	18
	34	34	44	12	30	8
TDP2	44	177	35	124	64	111
	117	188	88	46	102	27
TDRD1	21	5	31	7	15	1
	4	12	34	0	9	0
TDRD10	11	18	6	9	12	7
	8	17	7	2	4	2
TDRD12	11	0	4	0	1	0
	1	1	3	0	6	0
TDRD3	120	545	119	391	284	328
	489	668	270	217	365	95
TDRD5	35	1	20	1	5	1
	2	1	16	0	14	1
TDRD6	76	72	46	51	47	76
	52	109	39	41	60	15
TDRD7	36	84	45	85	56	73
	75	90	47	31	46	26
TDRD9	57	0	29	4	11	0
	2	0	34	0	14	3
TDRKH	37	76	38	46	33	44
	52	58	42	19	48	9
TEAD1	617	3431	512	2257	1716	2375
	2557	4057	1358	729	1789	663
TEAD2	20	30	18	24	22	17
	28	30	15	6	19	9
TEAD3	48	112	28	64	62	77
	141	141	54	49	77	36
TEAD4	52	288	52	144	113	218
	154	156	89	66	79	34
TEC	48	57	29	49	27	45
	37	43	40	16	32	6
TECPR1	16	39	19	24	23	26
	23	58	38	15	33	14
TECPR2	72	215	70	135	101	208
	160	206	95	76	96	53
TECR	122	561	85	486	211	486
	822	628	331	206	332	109
TECRL	279	1012	136	374	412	495
	905	1896	267	358	431	263
TECTA	31	22	33	17	13	13
	17	21	27	6	18	8
TECTB	16	0	4	0	2	1
	0	0	5	0	2	0
TEDDM1	8	0	7	0	2	0
	0	1	3	1	4	0
TEF	113	503	106	351	315	360
	475	704	349	129	306	101

TEFM	27	89	16	51	47	47
	71	121	52	21	45	12
TEK	68	278	62	196	112	117
	227	298	175	124	190	40
TEKT1	6	0	4	0	3	0
	0	0	8	0	2	0
TEKT2	10	0	5	1	1	0
	0	0	2	0	1	0
TEKT3	16	6	9	4	8	1
	3	1	14	0	3	0
TEKT4	5	0	1	0	2	0
	1	0	1	0	0	0
TEKT5	6	1	2	0	2	0
	1	0	3	0	0	0
TELO2	14	28	6	8	8	15
	16	37	19	17	24	12
TEN1	10	16	14	12	2	12
	18	24	12	3	9	4
TENC1	78	322	94	185	146	163
	274	480	139	119	194	84
TENM1	132	111	73	103	85	60
	63	118	79	33	84	19
TENM2	77	17	55	52	12	8
	12	0	48	7	20	12
TENM3	88	177	96	73	89	106
	146	180	156	60	132	72
TENM4	93	76	56	40	43	75
	38	29	72	9	45	3
TEP1	163	446	255	456	233	464
	171	526	256	114	231	75
TEPP	1	0	0	0	2	0
	0	2	3	0	1	0
TERF1	76	178	49	110	73	129
	141	187	74	69	93	39
TERF2	39	153	36	95	85	87
	140	129	47	36	57	26
TERF2IP	65	403	64	265	147	232
	331	405	179	94	210	75
TERT	10	0	12	0	0	1
	0	0	4	0	2	0
TES	18	78	16	47	47	33
	74	92	28	34	67	27
TESC	11	6	2	7	5	7
	24	17	5	4	7	1
TESK1	36	146	23	55	71	91
	154	70	54	44	55	27
TESK2	34	108	35	110	44	82
	83	88	73	20	75	13
TESPA1	43	11	24	22	18	16
	13	7	24	2	21	3
TET1	131	633	121	384	277	414
	272	226	218	103	183	56
TET2	264	924	295	784	439	853
	907	864	543	373	564	238
TET3	57	74	33	66	39	59
	39	52	55	24	61	16
TEX10	48	192	55	131	103	112
	131	269	106	73	169	33

TEX101	11	1	8	1	5	1
	0	2	4	7	4	4
TEX11	37	0	10	0	8	0
	0	0	10	0	3	0
TEX12	8	18	6	15	4	8
	5	35	10	8	18	2
TEX13A	6	0	2	0	1	0
	0	0	1	0	1	0
TEX14	48	6	40	2	12	5
	0	2	31	0	9	0
TEX15	59	0	33	1	9	1
	0	2	33	0	19	0
TEX19	5	0	3	0	0	0
	0	0	3	0	1	0
TEX2	191	1050	195	747	563	787
	791	743	387	310	428	131
TEX22	3	2	4	3	3	3
	0	7	5	0	5	2
TEX26	11	0	10	0	2	0
	0	0	9	1	6	0
TEX261	90	290	77	251	117	256
	279	392	143	75	156	59
TEX264	19	72	13	64	38	80
	84	113	62	28	36	15
TEX28	15	0	2	0	4	0
	0	0	1	0	4	0
TEX29	6	0	1	2	6	1
	2	0	2	0	4	1
TEX30	13	45	17	27	30	29
	61	78	18	23	32	14
TEX33	5	0	8	0	1	0
	0	0	5	0	4	0
TEX35	11	0	3	0	4	0
	0	2	5	0	4	0
TEX36	8	0	6	0	1	0
	0	0	1	0	2	0
TEX37	7	0	5	0	0	0
	0	0	2	0	0	0
TEX38	7	1	2	0	3	0
	0	0	5	1	1	0
TEX9	20	10	14	16	6	8
	9	14	23	5	20	1
TF	22	8	23	9	11	3
	2	8	21	0	16	0
TFAM	78	441	98	283	224	277
	340	435	213	122	230	63
TFAP2A	21	1	10	2	2	0
	1	0	5	0	3	0
TFAP2B	32	0	28	0	8	1
	0	0	14	0	10	0
TFAP2C	16	3	8	1	4	1
	0	0	5	0	3	0
TFAP2D	14	0	7	0	3	0
	0	0	5	0	6	0
TFAP2E	9	0	8	1	1	4
	0	1	8	0	1	2
TFAP4	19	14	12	10	13	12
	15	19	15	6	9	3

TFB1M	40	152	56	131	82	125
	156	202	115	53	85	30
TFB2M	34	148	37	85	85	83
	138	218	77	47	115	27
TFCP2	77	375	90	218	130	207
	259	312	110	96	141	37
TFCP2L1	68	3	29	10	13	10
	20	10	22	0	20	5
TFDP1	49	348	57	284	129	245
	339	355	134	71	190	60
TFDP2	216	779	159	659	448	517
	609	753	362	236	457	188
TFDP3	8	0	6	4	0	0
	2	0	3	0	2	0
TFE3	29	56	8	59	44	35
	29	60	25	22	36	23
TFEB	42	74	28	68	67	65
	114	153	87	55	108	46
TFEC	27	12	15	18	28	25
	12	28	19	10	17	5
TFF1	2	0	2	0	0	0
	0	0	1	0	1	0
TFF2	1	0	2	0	1	0
	0	0	2	0	1	0
TFF3	7	0	1	1	2	0
	1	2	1	1	0	0
TFG	70	308	41	217	138	210
	289	294	127	80	173	55
TFIP11	96	291	81	333	152	236
	357	308	192	92	240	53
TFPI	99	225	50	224	181	254
	227	442	97	119	129	85
TFPI2	18	16	11	19	13	19
	32	24	36	19	12	7
TFPT	11	32	13	18	24	43
	33	26	19	12	21	10
TFR2	18	0	4	0	1	0
	1	2	4	0	0	0
TFRC	400	2619	225	1062	1086	1157
	1030	1337	487	956	1320	195
TG	72	4	52	1	11	3
	6	10	44	0	14	0
TGDS	19	72	17	50	42	46
	60	109	53	20	52	14
TGFA	30	3	12	2	5	1
	2	2	7	1	7	0
TGFB1	11	29	16	6	23	4
	12	15	10	10	10	4
TGFB1I1	9	21	9	12	5	11
	7	15	9	4	11	0
TGFB2	126	456	137	323	216	299
	212	312	152	91	161	43
TGFB3	62	141	55	86	90	117
	88	190	51	49	64	34
TGFBI	36	167	30	138	77	130
	63	340	121	37	135	61
TGFBR1	116	530	121	452	279	343
	389	558	230	134	304	89

TGFBR2	117	597	128	496	296	409
	395	657	253	152	285	137
TGFBR3	135	312	114	354	235	261
	251	823	228	178	331	184
TGFBR3L	2	1	0	0	1	0
	2	1	0	1	0	4
TGFBRAP1	38	98	40	94	63	104
	85	123	71	50	58	30
TGIF1	25	37	19	15	18	20
	33	26	20	7	26	6
TGIF2	0	0	0	2	5	1
	9	3	1	0	0	0
TGIF2-C20ORF24		33	29	9	5	14
	12	7	7	21	2	20
	6					
TGIF2LX	5	0	0	0	0	0
	0	0	2	0	0	0
TGIF2LY	0	0	2	0	0	0
	0	0	0	0	0	0
TGM1	13	6	11	1	7	4
	2	1	8	1	6	0
TGM2	49	109	31	51	36	78
	117	151	51	34	67	23
TGM3	26	0	8	0	3	0
	0	0	8	1	6	0
TGM4	19	0	6	0	4	0
	0	0	6	0	4	0
TGM5	23	0	16	1	1	0
	1	1	8	0	5	0
TGM6	12	0	1	0	3	1
	0	0	4	0	3	0
TGM7	12	0	9	0	0	0
	0	0	9	0	7	0
TGOLN2	166	719	162	526	348	494
	599	730	303	227	349	152
TGS1	64	247	68	207	123	153
	236	266	138	89	192	90
TH	9	0	4	0	1	0
	0	0	3	0	2	0
TH1L	46	288	62	188	121	212
	242	273	136	65	165	40
THADA	137	430	123	336	235	281
	291	486	231	106	273	84
THAP1	46	151	32	93	76	127
	146	182	70	46	80	36
THAP10	11	25	6	12	10	16
	16	29	9	6	13	1
THAP11	38	159	29	153	78	144
	141	215	85	56	115	23
THAP2	52	145	45	120	85	141
	83	138	70	34	50	10
THAP3	19	61	20	52	34	57
	65	84	29	21	33	18
THAP4	49	245	40	151	81	162
	271	305	135	71	172	31
THAP5	96	374	88	338	168	357
	324	489	175	125	244	72

THAP6	46	201	42	167	97	134
	125	226	87	60	113	40
THAP7	7	34	15	50	16	26
	56	84	29	9	42	7
THAP8	7	11	4	8	14	12
	22	11	8	5	8	2
THAP9	89	262	65	225	151	252
	257	321	155	94	209	48
THBD	37	166	27	132	83	113
	87	127	80	38	65	28
THBS1	54	74	68	237	37	30
	38	99	57	18	48	90
THBS2	32	69	24	106	68	62
	22	106	34	7	43	41
THBS3	38	108	46	83	50	98
	43	117	68	26	36	13
THBS4	250	1051	532	765	602	1763
	732	548	300	185	294	144
THEG	12	0	2	0	1	0
	0	0	5	0	3	0
THEG5	2	0	1	0	0	0
	0	0	1	0	1	0
THEGL	5	0	8	0	4	0
	0	1	9	0	3	0
THEM4	82	210	36	145	73	141
	150	205	104	50	101	32
THEM5	8	2	1	0	1	0
	0	2	2	0	4	0
THEM6	6	28	8	17	11	28
	32	42	16	8	11	1
THEMIS	19	8	11	0	9	1
	3	16	7	0	14	0
THEMIS2	22	17	10	24	13	21
	14	47	14	11	16	1
THG1L	18	23	10	19	10	25
	27	34	18	12	16	5
THNSL1	39	105	31	75	86	69
	83	47	37	35	50	14
THNSL2	32	207	24	77	47	78
	130	189	31	43	25	48
THOC1	79	243	90	174	151	223
	260	368	175	97	170	60
THOC2	190	767	188	490	390	595
	559	856	298	250	435	158
THOC3	32	117	24	118	65	83
	105	115	55	32	71	25
THOC5	85	294	54	178	164	182
	232	302	141	101	145	50
THOC6	9	23	9	14	10	15
	27	42	18	3	20	3
THOC7	93	615	94	420	243	503
	577	771	293	192	329	100
THOP1	23	118	27	66	54	95
	77	137	49	32	68	24
THPO	15	2	7	2	6	5
	4	0	11	0	4	0
THRA	111	420	86	328	194	364
	376	389	216	109	190	72

THRAP3	294	1377	225	963	707	901
	1276	1528	540	543	797	364
THRB	217	834	200	573	419	629
	670	659	293	232	353	143
THRSP	28	32	28	48	22	48
	33	16	32	8	13	30
THSD1	25	65	19	62	19	30
	43	47	43	15	23	9
THSD4	63	69	52	105	66	54
	122	118	73	38	126	25
THSD7A	64	54	53	31	44	36
	40	32	82	14	33	14
THSD7B	44	2	27	9	18	0
	3	1	38	2	24	2
THTPA	14	52	8	25	21	24
	32	35	28	12	21	9
THUMPD1	177	617	173	586	392	500
	615	829	364	233	467	150
THUMPD2	53	187	64	133	96	102
	129	207	106	62	103	30
THUMPD3	79	244	51	198	131	177
	219	315	142	69	191	41
THY1	14	38	9	19	9	17
	16	21	19	4	6	6
THYN1	50	163	30	130	73	134
	156	306	81	57	120	24
TIA1	138	724	142	570	359	539
	483	902	369	209	384	126
TIAF1	0	0	0	0	0	0
	1	0	1	0	1	1
TIAL1	113	690	115	547	295	392
	559	795	203	159	316	85
TIAM1	84	30	34	47	31	29
	29	26	61	17	28	17
TIAM2	63	55	52	29	28	21
	17	64	62	27	44	19
TICAM1	10	15	3	12	8	20
	10	13	4	10	4	3
TICAM2	5	27	5	26	3	19
	4	14	10	7	14	2
TICRR	55	1	30	0	5	0
	0	0	19	0	13	0
TIE1	38	110	21	66	53	48
	82	124	62	29	48	26
TIFA	25	43	18	14	33	21
	25	58	29	7	28	11
TIFAB	5	0	0	0	1	1
	0	2	0	0	1	0
TIGD1	552	210	222	144	187	153
	116	214	275	43	185	29
TIGD2	16	36	9	26	12	26
	30	38	22	11	16	11
TIGD3	7	1	5	2	1	0
	0	0	1	0	1	0
TIGD4	16	10	11	8	7	6
	8	10	17	2	12	2
TIGD5	7	19	5	17	13	9
	13	29	16	6	19	2

TIGD6	24	32	18	20	13	25
	28	42	31	12	34	8
TIGD7	31	88	30	82	64	75
	93	108	66	38	76	26
TIGIT	19	4	10	1	7	1
	0	2	9	1	12	1
TIMD4	24	0	5	0	7	3
	1	0	15	0	3	0
TIMELESS	64	32	20	22	28	19
	19	23	31	5	22	9
TIMM10	30	129	25	126	34	70
	280	228	77	46	63	11
TIMM13	52	181	31	145	84	163
	303	302	109	56	115	35
TIMM17A	76	424	77	364	168	287
	335	484	234	94	268	49
TIMM17B	21	89	11	67	24	48
	69	134	33	27	66	19
TIMM21	67	455	70	356	195	299
	453	596	187	121	263	77
TIMM22	22	102	22	53	51	35
	76	112	42	26	35	18
TIMM23	92	577	74	408	212	410
	642	801	348	145	395	87
TIMM23B	11	6	3	11	6	10
	14	19	5	3	13	0
TIMM44	57	221	44	172	95	175
	306	369	135	96	189	67
TIMM50	50	175	33	85	66	105
	191	233	90	53	109	26
TIMM8A	16	46	6	16	25	14
	44	38	9	10	18	6
TIMM8B	84	344	54	350	125	231
	425	467	250	77	199	50
TIMM9	22	91	22	76	55	91
	108	120	54	32	64	17
TIMMDC1	170	1125	169	675	432	746
	989	1353	488	242	578	146
TIMP1	11	24	6	17	10	13
	12	14	13	7	9	4
TIMP2	79	394	76	482	170	236
	206	392	129	69	160	72
TIMP3	318	2146	273	1322	835	1229
	1735	2323	905	603	1210	437
TIMP4	18	20	9	11	16	17
	48	83	21	9	23	19
TINAG	17	6	8	3	11	3
	4	7	9	1	9	2
TINAGL1	28	145	18	86	53	65
	109	189	75	50	90	26
TINF2	41	139	32	114	78	82
	127	160	85	52	101	30
TIPARP	58	116	42	103	86	118
	124	242	89	47	109	36
TIPIN	15	42	17	25	22	19
	45	55	26	11	39	3
TIPRL	100	438	123	407	194	411
	410	588	242	121	266	62

TIRAP	12	27	9	27	14	20
	24	28	12	13	17	7
TJAP1	42	144	30	64	61	101
	118	124	71	47	63	27
TJP1	248	1396	174	848	554	725
	1017	964	560	367	629	282
TJP2	98	269	87	147	91	132
	163	416	101	58	162	46
TJP3	12	5	8	3	2	0
	0	0	8	0	0	0
TK1	16	5	11	4	12	0
	13	11	7	1	5	0
TK2	44	165	58	127	76	137
	132	214	98	36	116	44
TKT	27	39	20	21	35	36
	33	51	22	11	23	12
TKTL1	12	0	4	0	5	0
	3	1	5	0	4	0
TKTL2	8	0	4	0	4	0
	0	0	2	0	3	0
TLCD1	5	0	1	3	0	0
	1	0	1	0	1	0
TLCD2	47	24	14	23	22	6
	7	22	21	9	9	7
TLE1	112	404	87	311	183	232
	347	436	310	124	277	78
TLE2	46	132	27	86	63	132
	127	123	45	41	72	18
TLE3	38	87	31	46	35	62
	41	55	65	13	35	22
TLE4	105	458	115	222	215	309
	435	557	198	191	275	96
TLE6	9	1	5	4	2	1
	0	2	10	0	7	0
TLK1	115	506	115	394	211	364
	334	414	178	122	189	90
TLK2	191	773	174	514	370	598
	667	1184	373	309	504	248
TLL1	53	56	37	44	45	17
	23	36	57	3	36	9
TLL2	41	41	37	24	35	29
	21	14	36	8	18	7
TLN1	281	1403	284	1012	617	761
	851	1105	487	341	615	286
TLN2	303	1030	275	710	627	862
	783	1250	670	323	658	305
TLR1	10	20	22	25	22	32
	23	53	25	11	21	7
TLR10	21	3	11	6	5	0
	2	5	13	0	10	0
TLR2	14	11	14	29	12	19
	8	20	17	5	10	5
TLR3	24	39	9	37	18	19
	12	38	32	20	24	10
TLR4	54	171	54	141	122	156
	144	295	92	61	133	48
TLR5	27	23	11	22	17	19
	13	23	20	2	16	4

TLR6	43	15	19	16	18	22
	8	29	25	5	22	1
TLR7	40	5	2	6	9	13
	8	5	16	2	6	4
TLR8	25	6	12	7	8	9
	7	12	14	2	12	1
TLR9	13	3	10	6	16	9
	3	11	5	3	5	4
TLX1	6	0	6	0	0	0
	0	0	2	0	4	0
TLX1NB	13	1	6	0	4	0
	0	0	2	0	2	0
TLX2	2	0	3	0	4	0
	0	0	0	0	0	0
TLX3	0	0	2	0	0	0
	0	0	1	0	0	0
TM2D1	53	210	35	155	88	142
	199	342	116	71	126	47
TM2D2	30	128	26	155	62	116
	129	189	89	37	84	36
TM2D3	70	278	52	250	111	242
	280	357	156	82	206	34
TM4SF1	60	324	41	184	115	128
	320	393	218	107	156	64
TM4SF18	22	82	18	44	31	37
	74	96	74	42	58	13
TM4SF19	5	8	1	2	1	9
	0	2	0	0	1	3
TM4SF20	21	0	7	0	3	2
	0	1	12	0	5	1
TM4SF4	6	0	3	0	2	0
	0	0	4	0	3	0
TM4SF5	3	0	1	0	1	0
	0	0	2	0	0	0
TM6SF1	90	455	99	353	195	381
	467	593	203	129	264	63
TM6SF2	12	0	3	0	1	0
	0	0	2	0	0	0
TM7SF2	13	32	17	20	9	17
	35	36	29	11	17	10
TM7SF3	66	251	63	170	163	185
	158	292	154	83	112	49
TM9SF1	27	70	26	78	48	89
	78	118	53	27	58	21
TM9SF2	149	763	131	584	329	510
	560	944	395	221	428	120
TM9SF3	200	1116	216	932	568	878
	966	1244	524	361	710	220
TM9SF4	70	307	52	172	122	191
	221	265	128	68	144	56
TMA16	22	84	23	69	40	91
	87	131	68	26	76	18
TMA7	112	567	42	248	170	296
	502	951	127	88	185	33
TMBIM1	165	730	139	499	349	579
	541	811	369	255	406	127
TMBIM4	80	425	73	421	156	378
	503	660	270	113	298	84

TMBIM6	474	2031	374	1676	1139	1885
	1532	2622	1101	541	1206	325
TMC1	24	4	26	4	16	7
	2	12	22	2	20	2
TMC2	31	0	14	3	2	0
	1	0	12	0	3	1
TMC3	39	0	14	0	8	0
	1	1	20	0	6	0
TMC4	16	5	15	7	3	2
	4	8	5	2	3	0
TMC5	45	0	18	0	5	1
	0	0	33	1	13	0
TMC6	9	17	11	17	9	10
	9	22	7	6	6	5
TMC7	26	23	11	4	11	7
	17	10	22	3	14	1
TMC8	14	10	14	6	3	2
	5	7	13	3	8	1
TMCC1	226	767	186	578	461	587
	868	952	445	260	591	136
TMCC2	26	63	33	78	36	99
	60	66	37	14	33	5
TMCC3	97	372	84	305	241	393
	240	251	185	76	157	45
TMCO1	102	463	82	413	251	459
	424	765	310	139	321	91
TMCO2	6	0	3	0	1	1
	0	0	0	0	2	0
TMCO3	53	111	36	98	80	106
	151	162	87	50	90	24
TMCO4	21	22	16	10	13	11
	15	33	23	4	22	6
TMCO5A	23	0	16	0	6	0
	0	0	11	0	12	0
TMCO6	22	24	13	10	11	9
	16	36	21	7	20	2
TMCO7	94	238	75	165	146	205
	154	283	136	81	155	47
TMED1	19	62	19	90	35	79
	82	132	43	28	53	14
TMED10	334	1791	274	1407	886	1300
	1310	1792	809	461	986	321
TMED2	155	1006	150	965	434	896
	825	1131	524	244	483	160
TMED3	5	13	7	15	5	9
	7	6	9	3	10	4
TMED4	102	411	84	298	177	331
	344	484	261	111	318	64
TMED5	238	1118	250	865	776	1035
	968	1767	547	464	608	253
TMED6	6	10	5	3	4	0
	2	6	5	5	4	0
TMED7	10	81	14	100	13	59
	100	112	75	40	56	19
TMED7-TICAM2		43	221	62	180	124
	197	129	253	135	69	124
	30					

TMED8	7	13	8	9	3	14
	6	16	12	3	11	0
TMED9	28	168	26	80	85	101
	119	121	49	25	74	16
TMEFF1	1	0	1	0	0	0
	0	0	0	0	1	0
TMEFF2	15	13	7	4	10	7
	12	2	5	4	7	2
TMEM100	13	15	17	13	15	12
	29	35	17	6	13	11
TMEM101	23	82	13	76	39	87
	98	153	53	33	51	24
TMEM102	5	3	3	1	1	2
	5	3	2	1	3	1
TMEM104	18	24	26	24	12	24
	13	44	18	11	31	13
TMEM105	12	0	2	0	2	0
	2	1	2	0	3	0
TMEM106A	28	28	14	28	12	24
	25	23	15	15	28	6
TMEM106B	269	1215	273	1098	555	983
	1040	1381	596	348	771	202
TMEM106C	41	103	39	94	50	103
	67	122	87	23	89	24
TMEM107	12	12	8	5	7	2
	1	10	11	1	10	1
TMEM108	40	89	27	35	52	66
	91	73	57	27	51	15
TMEM109	56	256	57	217	114	249
	249	276	108	89	139	34
TMEM11	79	456	45	277	228	454
	570	548	228	153	216	69
TMEM110	7	31	9	30	23	15
	37	82	28	10	29	6
TMEM110-MUSTN1		27	95	32	59	29
	85	85	103	48	31	58
	17					
TMEM114	0	0	1	0	0	0
	0	0	0	0	0	0
TMEM115	28	48	17	46	29	40
	69	76	35	25	43	13
TMEM116	43	109	34	91	47	83
	114	129	57	25	77	17
TMEM117	36	91	21	64	52	71
	70	116	69	25	54	12
TMEM119	13	15	9	7	3	1
	8	8	5	3	5	1
TMEM120A	26	81	10	26	32	31
	48	78	29	18	31	16
TMEM120B	81	269	50	161	144	165
	128	301	112	82	131	35
TMEM121	1	0	0	0	0	0
	0	2	2	0	0	0
TMEM123	121	394	105	309	281	356
	354	725	305	182	268	123
TMEM125	2	0	3	0	1	0
	0	0	0	0	1	0

TMEM126A	26	150	24	101	49	125
	190	326	132	51	118	21
TMEM126B	69	324	70	215	117	318
	264	449	215	72	183	46
TMEM127	45	170	43	124	96	167
	150	195	93	45	95	43
TMEM128	33	67	13	53	20	45
	61	97	49	24	47	8
TMEM129	22	50	13	43	36	48
	39	104	42	18	38	13
TMEM130	17	3	10	0	4	0
	0	1	5	0	4	0
TMEM131	258	1433	281	1011	510	845
	1092	1713	633	419	894	330
TMEM132A	13	5	6	7	3	3
	13	17	6	2	4	0
TMEM132B	48	4	28	5	12	2
	4	24	44	5	19	4
TMEM132C	18	5	12	3	14	2
	8	10	11	1	4	6
TMEM132D	25	0	13	0	7	1
	1	0	12	0	5	0
TMEM132E	8	0	6	0	0	0
	0	0	3	0	3	0
TMEM133	18	27	6	30	19	17
	18	26	16	10	12	3
TMEM134	8	15	2	9	5	7
	23	21	8	4	11	3
TMEM135	137	250	106	176	188	261
	177	430	146	73	177	67
TMEM136	34	36	14	24	16	12
	22	20	22	6	19	9
TMEM138	14	36	17	47	16	41
	30	51	26	16	32	11
TMEM139	13	1	18	1	5	2
	0	0	5	0	4	0
TMEM140	92	221	56	142	102	182
	177	217	105	43	75	36
TMEM141	13	54	15	34	20	15
	62	70	25	17	22	3
TMEM143	51	190	23	127	61	117
	188	137	45	50	78	22
TMEM144	30	100	29	72	18	48
	50	41	40	16	58	12
TMEM145	15	0	8	0	1	0
	0	3	4	0	1	0
TMEM147	43	129	18	104	33	137
	94	144	78	20	69	19
TMEM14A	16	20	10	24	7	16
	16	29	13	3	9	8
TMEM14B	96	471	63	377	193	341
	602	737	247	145	289	95
TMEM14C	105	449	84	400	236	328
	626	682	213	142	265	70
TMEM14E	14	30	9	12	27	40
	25	93	25	11	27	9
TMEM150A	21	65	10	64	23	36
	74	67	57	18	48	17

TMEM150B	8	0	4	0	0	0
	0	0	1	0	1	1
TMEM150C	16	43	12	44	24	21
	34	60	28	12	29	12
TMEM151A	3	0	0	0	1	0
	0	0	0	0	1	0
TMEM151B	16	0	8	0	0	0
	0	0	9	0	1	0
TMEM154	28	21	13	5	6	11
	11	11	20	2	9	3
TMEM155	22	0	9	0	2	0
	0	0	9	0	8	0
TMEM156	13	2	8	2	3	0
	0	0	11	0	10	0
TMEM158	5	5	0	5	6	0
	0	4	7	1	2	2
TMEM159	175	500	170	449	212	330
	497	753	343	228	584	166
TMEM160	3	14	3	15	4	7
	6	8	4	2	10	1
TMEM161A	23	60	12	51	22	48
	48	130	50	27	56	19
TMEM161B	36	176	63	149	100	150
	189	291	113	54	132	39
TMEM163	11	1	12	5	2	1
	2	1	2	0	3	0
TMEM164	133	474	91	297	219	314
	300	426	202	94	201	68
TMEM165	22	187	31	172	86	133
	136	219	103	40	113	29
TMEM167A	126	537	100	463	286	425
	369	648	259	137	314	116
TMEM167B	61	210	60	234	127	204
	205	294	149	51	121	44
TMEM168	78	333	85	231	129	283
	267	426	174	112	262	55
TMEM169	24	1	6	0	7	1
	4	3	11	0	10	0
TMEM17	10	6	5	6	6	5
	9	9	8	1	4	3
TMEM170A	6	13	7	19	7	5
	17	12	10	4	5	2
TMEM170B	122	544	167	631	279	505
	487	552	333	153	341	92
TMEM171	3	7	0	2	2	5
	0	0	6	2	1	0
TMEM173	10	49	14	33	20	20
	22	36	29	6	46	6
TMEM174	7	0	5	0	2	0
	0	0	5	0	5	0
TMEM175	13	34	6	28	15	21
	33	40	17	10	21	11
TMEM176A	7	11	7	1	9	8
	0	12	5	2	7	1
TMEM176B	13	39	27	28	43	36
	15	57	17	5	19	7
TMEM177	11	29	10	16	11	10
	35	40	16	11	16	4

TMEM178A	9	20	7	7	7	8
	6	11	9	1	8	0
TMEM178B	75	203	121	250	55	194
	164	93	169	57	186	54
TMEM179	4	0	5	0	3	0
	0	0	0	0	0	0
TMEM179B	14	26	8	24	19	22
	13	36	14	14	15	7
TMEM18	60	177	56	178	67	125
	188	291	125	43	123	25
TMEM180	12	5	4	1	4	3
	3	5	4	2	8	2
TMEM181	62	290	85	238	131	258
	227	307	182	90	141	45
TMEM182	382	2485	600	2304	1034	1909
	2305	2389	1400	939	1691	288
TMEM183A	52	141	45	104	78	116
	101	214	87	41	86	28
TMEM183B	4	0	2	0	0	1
	0	1	2	1	0	1
TMEM184A	18	14	18	13	12	19
	12	22	11	6	12	2
TMEM184B	34	174	24	95	61	106
	141	114	65	40	91	18
TMEM184C	71	289	53	187	169	194
	233	264	132	88	162	39
TMEM185A	39	84	32	71	43	75
	45	66	38	25	45	14
TMEM185B	64	224	53	175	137	166
	175	275	140	84	173	42
TMEM186	9	34	11	32	21	13
	56	57	21	8	24	6
TMEM187	8	10	0	13	10	13
	15	20	15	6	7	3
TMEM189	0	30	4	16	8	23
	45	29	28	9	7	7
TMEM189-UBE2V1		83	250	65	235	98
	255	127	210	105	74	139
	26					
TMEM19	124	450	125	401	278	433
	426	525	228	164	256	111
TMEM190	2	0	5	0	0	0
	0	0	2	0	1	0
TMEM191B	1	2	0	0	0	0
	0	1	0	0	1	0
TMEM191C	2	0	1	1	1	0
	0	0	1	0	0	0
TMEM192	53	198	34	158	76	142
	125	236	138	60	165	48
TMEM194A	36	138	46	88	67	83
	82	167	62	31	85	26
TMEM194B	28	24	24	25	6	24
	19	30	26	13	21	6
TMEM196	11	0	16	0	2	0
	0	0	4	0	1	0
TMEM198	12	0	13	1	1	1
	0	0	3	0	5	1

TMEM199	23	42	16	28	24	32
	37	86	40	20	51	8
TMEM2	96	249	121	182	155	221
	181	265	128	125	178	63
TMEM200A	28	23	23	6	10	12
	3	5	29	2	10	0
TMEM200B	10	11	1	11	9	7
	8	17	10	3	7	4
TMEM200C	3	1	0	0	1	0
	0	0	0	0	1	0
TMEM201	38	107	21	55	44	87
	96	122	55	27	55	16
TMEM202	13	0	3	0	1	0
	0	0	6	0	2	0
TMEM203	18	149	24	100	62	94
	114	152	55	31	71	19
TMEM204	7	26	2	20	13	18
	17	36	23	6	24	13
TMEM205	34	134	27	108	46	112
	145	211	60	38	79	27
TMEM206	12	6	11	5	4	13
	10	6	9	1	10	2
TMEM207	10	0	3	0	0	0
	0	0	6	0	2	0
TMEM208	20	64	12	28	38	47
	56	117	16	14	43	5
TMEM209	46	113	41	100	72	90
	112	208	81	35	98	23
TMEM210	1	0	3	0	0	0
	0	0	2	0	1	0
TMEM211	1	0	0	0	0	0
	0	0	0	0	0	0
TMEM212	95	12	44	7	28	7
	8	13	40	1	24	0
TMEM213	19	2	5	0	2	1
	1	0	10	0	9	0
TMEM214	37	86	33	74	49	71
	73	88	44	35	41	21
TMEM215	27	18	16	10	14	7
	6	7	19	2	7	1
TMEM216	6	27	6	26	17	18
	21	28	17	3	12	5
TMEM217	14	1	10	0	2	3
	0	1	12	0	6	0
TMEM218	51	98	30	57	58	67
	61	114	69	16	50	14
TMEM219	22	85	19	63	32	81
	80	123	63	33	50	15
TMEM220	30	86	22	51	41	55
	83	103	41	35	50	19
TMEM221	5	0	0	1	0	0
	0	1	2	0	1	0
TMEM222	17	50	9	24	17	42
	39	66	21	16	34	11
TMEM223	15	60	5	53	17	45
	71	133	33	25	42	5
TMEM225	10	0	7	0	4	0
	0	0	1	0	1	0

TMEM229A	1	0	3	3	0	0
	0	0	6	0	0	0
TMEM229B	12	4	9	4	6	2
	1	4	7	0	5	2
TMEM230	134	675	86	377	241	382
	482	750	275	160	307	96
TMEM231	22	14	16	16	11	22
	20	18	17	5	30	4
TMEM232	43	6	19	10	12	13
	9	17	29	3	19	1
TMEM233	38	132	34	146	86	185
	192	154	86	80	99	35
TMEM234	11	24	5	14	4	20
	18	26	14	8	14	7
TMEM235	8	0	3	0	0	0
	0	0	1	0	1	0
TMEM236	30	9	10	2	5	7
	4	12	22	0	21	1
TMEM237	105	297	123	241	159	265
	215	434	170	109	233	73
TMEM238	2	3	2	1	0	0
	1	3	0	1	0	0
TMEM239	0	0	2	0	0	0
	0	0	0	0	0	0
TMEM240	0	1	1	2	0	2
	4	2	1	2	3	1
TMEM241	29	18	17	12	10	18
	6	14	19	3	14	4
TMEM242	67	282	69	172	120	150
	214	306	136	79	117	49
TMEM244	1	1	3	0	1	1
	0	1	1	0	0	0
TMEM245	340	1523	456	1402	676	1287
	1680	2317	870	433	1193	387
TMEM246	13	17	4	25	10	22
	16	21	14	10	7	6
TMEM247	6	0	2	0	0	0
	0	0	4	0	0	0
TMEM248	80	404	82	347	199	350
	325	568	216	136	273	119
TMEM249	0	0	0	0	1	0
	0	0	2	0	0	0
TMEM25	30	71	25	50	22	52
	44	45	37	16	31	6
TMEM251	17	64	12	52	23	56
	77	93	26	20	38	3
TMEM252	8	1	2	1	2	1
	0	2	1	0	1	0
TMEM253	9	3	9	1	0	1
	1	0	3	0	8	0
TMEM26	28	10	18	6	15	6
	16	7	24	8	24	1
TMEM27	9	0	1	0	2	0
	1	0	4	0	1	0
TMEM30A	187	920	199	692	404	638
	661	939	405	227	407	142
TMEM30B	14	17	12	21	11	10
	15	21	19	8	19	5

TMEM31	3	1	1	0	0	3
	2	1	1	1	4	0
TMEM33	118	486	105	365	215	332
	328	677	285	137	342	117
TMEM35	11	3	1	5	2	4
	3	9	5	2	2	3
TMEM37	5	7	5	8	4	10
	10	10	4	3	10	8
TMEM38A	269	1120	278	1314	678	1300
	1262	2063	991	571	1215	360
TMEM38B	245	995	271	1191	550	986
	882	1571	628	327	777	193
TMEM39A	51	115	30	114	75	80
	92	160	86	41	87	29
TMEM39B	11	27	10	14	8	24
	24	12	15	14	17	6
TMEM40	11	4	9	0	0	0
	1	2	5	1	1	0
TMEM41A	39	94	21	73	52	65
	97	120	69	30	65	26
TMEM41B	62	246	50	191	127	177
	188	342	144	66	166	59
TMEM42	17	58	5	47	19	44
	58	75	49	8	34	7
TMEM43	100	318	101	282	146	270
	225	354	214	95	179	74
TMEM44	25	30	13	9	13	10
	15	12	12	5	13	5
TMEM45A	17	3	5	9	1	13
	1	15	6	3	4	0
TMEM45B	14	2	6	0	0	1
	1	1	12	0	0	1
TMEM47	181	795	204	464	426	501
	498	911	354	200	434	161
TMEM48	57	121	57	144	93	139
	157	200	101	60	120	21
TMEM5	30	96	28	76	43	109
	92	112	74	27	71	20
TMEM50A	51	216	46	150	97	142
	196	283	123	71	111	38
TMEM50B	99	326	56	253	184	264
	282	620	204	118	233	74
TMEM51	21	13	7	5	6	7
	3	10	8	4	6	2
TMEM52	65	246	83	421	231	465
	501	433	240	144	245	64
TMEM52B	19	0	9	1	3	0
	0	0	12	0	2	0
TMEM53	7	8	6	4	1	6
	6	2	5	0	2	1
TMEM54	8	7	2	3	2	4
	0	2	4	1	4	2
TMEM55A	51	128	31	107	70	124
	96	138	53	34	64	23
TMEM55B	22	78	13	58	47	47
	59	88	24	19	45	11
TMEM56	10	112	13	159	53	71
	156	84	40	19	32	10

TMEM56-RWDD3	22	82	30	55	52	43	56	57	50	9	46	6
TMEM57	163	785	150	441	446	565	739	921	302	228	426	132
TMEM59	129	707	131	705	336	519	730	1100	419	174	450	110
TMEM59L	7	2	3	0	0	1	3	2	7	1	5	0
TMEM60	9	44	8	74	25	48	65	74	22	18	44	7
TMEM61	6	0	2	0	0	0	0	0	2	0	0	0
TMEM62	37	90	32	68	45	72	76	145	68	32	59	19
TMEM63A	57	213	65	135	71	130	145	190	80	58	103	33
TMEM63B	77	180	60	128	106	153	136	178	70	92	109	38
TMEM63C	20	9	17	9	6	8	4	0	15	0	9	0
TMEM64	84	224	96	262	143	198	183	297	125	98	206	59
TMEM65	162	711	189	524	434	442	639	873	365	274	633	139
TMEM66	209	1048	193	829	427	728	936	1144	477	227	569	137
TMEM67	45	29	26	21	24	12	21	42	19	12	48	7
TMEM68	28	69	18	41	43	63	78	99	32	23	49	12
TMEM69	42	97	17	70	58	81	115	138	44	58	68	21
TMEM70	120	420	48	690	150	879	2074	363	397	408	356	118
TMEM71	15	5	4	10	4	20	6	28	18	6	16	0
TMEM72	2	0	4	1	0	0	0	0	0	0	3	0
TMEM74	9	4	3	1	11	6	5	5	13	14	12	2
TMEM74B	10	4	3	4	6	3	6	12	3	2	4	1
TMEM79	10	11	5	1	2	4	8	3	2	1	1	0
TMEM80	8	28	9	51	12	18	13	50	29	12	21	9
TMEM81	11	19	7	10	13	22	5	15	8	3	17	1
TMEM82	3	0	0	0	2	1	0	0	0	0	0	0
TMEM86A	37	18	19	11	10	17	25	51	18	11	30	3
TMEM86B	1	10	4	0	1	6	0	7	3	1	3	2
TMEM87A	95	337	89	280	174	263	244	400	194	109	197	84

TMEM87B	47	90	31	82	38	95
	98	94	74	20	38	23
TMEM88	9	20	4	7	18	22
	25	28	7	7	15	3
TMEM88B	1	0	0	0	0	0
	0	0	0	0	1	0
TMEM89	4	0	2	0	0	0
	0	0	0	0	0	0
TMEM8A	28	59	13	39	36	38
	44	61	27	15	30	8
TMEM8B	24	80	22	40	29	30
	31	55	26	11	30	11
TMEM8C	3	0	2	0	0	0
	0	0	2	0	1	0
TMEM9	27	63	16	90	24	99
	85	123	85	37	60	16
TMEM91	3	2	6	5	5	3
	9	8	5	0	7	1
TMEM92	19	0	6	0	1	0
	0	0	7	0	3	0
TMEM95	7	0	2	0	0	0
	0	0	1	0	0	0
TMEM97	28	58	19	47	26	73
	74	88	40	27	49	15
TMEM98	18	10	8	21	3	7
	18	14	13	5	13	2
TMEM99	19	40	10	16	19	21
	29	36	24	2	21	6
TMEM9B	56	205	32	181	86	181
	221	250	135	48	115	33
TMF1	139	599	154	475	347	504
	431	618	280	272	351	145
TMIE	5	6	7	3	5	7
	6	10	5	0	9	2
TMIGD1	19	0	7	0	0	0
	0	0	6	0	5	0
TMIGD2	0	0	1	0	0	0
	0	0	1	0	1	0
TMLHE	165	561	126	384	278	344
	622	575	214	175	308	87
TMOD1	219	2601	432	2271	727	1508
	1733	1474	563	435	760	235
TMOD2	104	253	108	267	130	278
	158	230	149	87	128	58
TMOD3	67	272	59	199	122	159
	214	338	149	80	181	79
TMOD4	606	3341	507	2313	1669	2425
	2531	2900	1165	970	1413	472
TMPO	106	380	121	211	230	252
	200	614	211	126	226	74
TMPPE	0	0	0	0	0	2
	0	2	1	0	0	0
TMPRSS11A	18	0	8	0	3	0
	0	0	5	0	5	0
TMPRSS11B	13	0	7	1	4	1
	0	1	9	0	3	0

TMPRSS11BNL	25	0	9	0	4	0	0	4	8	0	0	0
TMPRSS11D	20	2	14	0	3	1	4	2	9	0	6	0
TMPRSS11E	9	0	10	0	5	0	0	0	11	0	6	0
TMPRSS11F	23	0	10	2	9	0	0	0	11	0	2	1
TMPRSS12	12	0	3	1	3	1	0	0	6	0	1	0
TMPRSS13	24	0	22	1	7	0	0	0	15	0	3	0
TMPRSS15	28	0	17	0	13	0	0	0	22	0	4	0
TMPRSS2	23	0	12	1	4	0	0	0	17	0	3	0
TMPRSS3	21	3	8	1	3	0	0	0	12	0	8	0
TMPRSS4	18	1	13	0	3	3	0	0	7	0	10	0
TMPRSS5	15	25	13	20	16	29	18	49	34	5	25	5
TMPRSS6	14	0	8	0	3	0	0	0	5	0	2	1
TMPRSS7	27	0	18	0	5	0	0	0	17	0	8	0
TMPRSS9	6	1	6	0	1	5	0	0	5	0	4	0
TMSB10	120	1011	141	648	387	546	1163	1498	527	324	442	170
TMSB15A	4	0	2	0	1	1	0	0	2	0	1	0
TMSB15B	2	0	3	0	2	0	0	1	3	0	0	0
TMSB4X	275	1615	241	812	619	1104	1353	2738	724	457	811	266
TMSB4Y	0	6	6	17	0	13	2	15	8	0	6	0
TMTC1	259	965	249	586	542	752	776	1713	477	361	586	302
TMTC2	41	29	27	30	34	13	42	43	41	19	38	7
TMTC3	102	215	70	205	143	146	187	287	108	68	140	41
TMTC4	75	145	40	146	95	140	104	179	83	29	70	20
TMUB1	16	42	11	46	29	30	42	47	34	13	24	10
TMUB2	11	64	18	71	38	53	62	78	33	31	54	13
TMX1	71	239	52	232	131	199	269	371	179	63	165	45
TMX2	69	283	50	204	116	207	278	371	150	64	168	44
TMX3	101	511	136	485	220	409	367	519	264	141	311	109

TMX4	111	478	119	471	262	374
	437	667	251	254	379	130
TNC	70	83	70	28	21	36
	16	42	102	11	41	49
TNF	15	0	4	0	1	0
	1	1	5	0	5	0
TNFAIP1	48	171	35	132	94	140
	160	222	114	64	112	42
TNFAIP2	53	164	68	192	113	165
	97	261	92	48	129	30
TNFAIP3	38	112	52	89	56	102
	50	67	47	23	39	14
TNFAIP6	11	17	10	4	8	3
	15	26	13	4	13	0
TNFAIP8	15	21	9	20	7	12
	14	30	18	2	5	9
TNFAIP8L1	13	33	11	22	30	24
	35	33	22	14	22	8
TNFAIP8L2	0	0	0	0	0	0
	1	2	3	0	0	0
TNFAIP8L2-SCNM1		41	73	20	42	23
	67	43	60	65	14	42
	5					
TNFAIP8L3	18	11	11	11	5	2
	3	4	11	1	14	0
TNFRSF10A	13	11	9	9	5	9
	12	11	13	7	13	3
TNFRSF10B	34	73	34	66	32	41
	40	66	49	24	40	12
TNFRSF10C	8	9	4	5	5	4
	2	2	10	3	1	0
TNFRSF10D	25	30	18	22	12	16
	19	21	16	6	9	1
TNFRSF11A	34	11	15	11	11	17
	13	18	17	10	12	3
TNFRSF11B	12	3	15	15	8	2
	10	0	11	0	8	1
TNFRSF12A	8	24	13	12	15	27
	10	61	30	12	10	7
TNFRSF13B	7	0	6	1	3	3
	0	0	3	2	6	1
TNFRSF13C	2	1	0	0	0	0
	0	0	3	0	0	0
TNFRSF14	11	29	11	21	8	20
	23	31	13	11	10	3
TNFRSF17	12	0	6	0	0	0
	0	0	2	0	2	0
TNFRSF18	2	0	2	0	1	0
	0	0	1	0	0	0
TNFRSF19	62	118	26	90	63	118
	77	47	70	23	52	10
TNFRSF1A	28	126	26	142	48	108
	114	122	65	38	57	21
TNFRSF1B	30	70	11	36	42	30
	38	64	31	16	29	19
TNFRSF21	28	49	22	46	25	26
	39	63	37	18	35	11

TNFRSF25	6	7	6	11	7	15
	9	15	5	3	5	0
TNFRSF4	2	3	1	2	1	1
	3	5	1	0	2	0
TNFRSF6B	3	4	4	3	7	3
	4	10	5	0	2	2
TNFRSF8	11	9	13	5	5	3
	5	5	7	2	9	1
TNFRSF9	35	1	20	2	8	1
	0	2	10	0	11	0
TNFSF10	63	321	32	136	137	183
	249	349	175	224	220	75
TNFSF11	24	0	10	0	3	0
	0	1	11	0	9	0
TNFSF12	6	26	5	5	6	13
	17	16	7	2	11	0
TNFSF12-TNFSF13		17	17	10	16	13
	22	18	28	12	3	16
	1					
TNFSF13	11	4	2	2	4	5
	3	2	1	4	0	1
TNFSF13B	13	12	12	6	10	11
	4	13	13	8	10	1
TNFSF14	22	3	3	2	4	2
	1	2	6	1	3	0
TNFSF15	46	3	22	10	10	0
	1	1	22	1	10	0
TNFSF18	4	0	1	0	4	0
	0	0	5	0	0	0
TNFSF4	15	15	19	8	11	10
	8	25	26	8	19	3
TNFSF8	35	3	17	7	11	11
	1	9	25	0	8	1
TNFSF9	4	3	3	0	1	2
	1	2	6	0	1	0
TNIK	198	698	232	387	352	669
	1008	1048	416	351	655	196
TNIP1	156	924	138	514	406	556
	635	719	264	319	366	118
TNIP2	25	105	27	108	46	130
	86	110	68	48	54	29
TNIP3	24	1	18	0	13	2
	1	0	7	0	4	0
TNK1	17	1	2	0	1	0
	0	1	7	0	2	0
TNK2	79	284	68	153	130	246
	164	268	126	107	130	54
TNKS	223	960	282	933	471	703
	818	1038	532	340	705	226
TNKS1BP1	71	218	73	174	149	200
	148	184	101	61	117	61
TNKS2	460	2349	445	2283	1172	1966
	2055	2712	1444	642	1590	419
TNMD	17	7	5	4	3	1
	3	3	2	1	3	3
TNN	44	2	21	1	11	1
	8	1	27	0	11	0

TNNC1	9659	23767	4902	11249	10984	11285
	30960	70115	7568	11075	15157	5763
TNNC2	9013	58780	11721	60620	35670	87183
	85794	73828	62937	29504	61040	15825
TNNI1	5138	13569	2843	5094	6595	5442
	13571	28896	3890	5648	8378	4088
TNNI2	2401	21535	2625	24877	11083	19089
	28897	20588	15544	4506	15532	2273
TNNI3	14	12	5	0	14	3
	24	40	10	4	10	2
TNNI3K	0	4	0	0	1	0
	2	1	0	0	0	0
TNNT1	10291	37102	6994	11390	16808	16157
	45268	86492	10321	23975	21093	11412
TNNT2	13	55	26	5	10	3
	10	29	13	6	15	4
TNNT3	5223	50674	6215	35744	21452	47777
	55869	29714	30105	11893	31371	4259
TNP1	2	1	6	0	1	0
	0	1	2	0	3	0
TNP2	5	0	2	0	1	0
	0	0	2	0	1	0
TNPO1	407	2344	540	1941	1315	1933
	1603	2206	1108	709	1204	371
TNPO2	68	161	64	124	76	138
	124	151	85	59	99	50
TNPO3	186	685	127	449	373	554
	388	648	269	198	367	125
TNR	46	1	25	0	10	0
	1	0	27	0	18	0
TNRC18	63	261	56	156	76	151
	129	205	91	65	79	57
TNRC6A	299	1438	336	856	656	998
	1161	1623	605	523	834	235
TNRC6B	555	2391	549	1836	1129	1711
	1807	2677	1043	708	1408	470
TNRC6C	180	845	213	517	306	504
	537	569	327	212	302	104
TNS1	487	2974	553	2249	1163	1903
	1964	2097	837	594	1102	563
TNS3	126	227	90	232	127	218
	210	358	196	130	218	86
TNS4	19	1	13	0	5	0
	0	0	11	0	1	0
TNXB	262	998	189	1224	715	865
	352	1440	437	253	541	330
TOB1	179	1438	179	848	543	686
	1171	1605	335	398	613	232
TOB2	223	1140	158	566	606	701
	1481	1185	417	294	667	251
TOE1	19	28	6	22	20	30
	32	49	30	11	30	6
TOLLIP	38	135	29	87	60	73
	97	120	56	34	67	16
TOM1	105	431	79	336	229	285
	375	589	205	149	309	100
TOM1L1	22	21	26	19	16	18
	31	52	25	3	15	7

TOM1L2	83	530	110	401	257	348
	384	419	197	146	304	92
TOMM20	186	861	212	768	402	677
	804	936	458	244	516	161
TOMM20L	1	2	4	1	3	0
	1	5	12	2	1	1
TOMM22	84	436	55	260	137	208
	398	507	208	87	176	52
TOMM34	22	56	13	36	27	31
	30	50	27	11	26	12
TOMM40	23	125	35	71	45	69
	101	170	76	36	65	25
TOMM40L	47	274	43	157	100	135
	185	204	87	45	122	25
TOMM5	123	585	65	347	279	485
	724	972	236	161	280	103
TOMM6	49	270	26	146	102	200
	246	543	106	65	149	26
TOMM7	237	1057	170	1030	438	852
	1330	2440	589	308	644	160
TOMM70A	342	1839	332	1230	913	1103
	1132	1975	690	490	881	259
TONSL	11	3	10	4	1	1
	3	5	5	2	8	0
TOP1	78	406	101	256	146	205
	298	356	151	75	210	66
TOP1MT	11	17	11	11	12	22
	12	17	19	4	5	2
TOP2A	56	8	21	5	12	9
	11	6	38	3	16	0
TOP2B	381	1925	415	1596	954	1357
	1522	2237	911	672	1294	413
TOP3A	37	87	35	60	42	70
	70	81	55	34	65	18
TOP3B	31	68	25	46	22	37
	48	75	28	27	30	12
TOPAZ1	30	0	14	0	9	0
	0	0	19	0	17	0
TOPBP1	102	320	109	227	154	240
	230	334	166	94	260	46
TOPORS	72	269	61	187	163	289
	250	366	140	107	211	68
TOR1A	22	65	16	59	30	57
	65	102	67	25	37	17
TOR1AIP1	185	848	175	827	490	924
	885	991	488	304	714	213
TOR1AIP2	272	974	264	796	563	722
	842	1313	508	269	629	246
TOR1B	30	90	32	86	54	97
	60	118	59	30	58	25
TOR2A	9	20	2	12	6	17
	9	20	8	4	11	3
TOR3A	20	33	11	32	23	46
	46	47	26	12	29	6
TOR4A	12	7	7	6	7	2
	10	8	9	2	6	2
TOX	21	4	9	6	12	2
	2	3	17	0	11	0

TOX2	14	5	16	0	5	2
	2	1	9	0	5	1
TOX3	17	0	12	4	5	0
	0	0	10	2	11	1
TOX4	150	762	124	454	262	514
	502	579	282	183	341	102
TP53	25	29	18	37	14	20
	19	30	18	6	24	8
TP53AIP1	14	0	4	0	0	2
	1	1	5	0	1	0
TP53BP1	127	474	127	320	223	355
	292	367	180	133	197	87
TP53BP2	86	343	77	245	199	288
	215	287	171	120	153	54
TP53I11	25	50	16	29	19	27
	26	55	23	15	29	7
TP53I13	9	21	9	15	18	20
	23	35	14	11	13	2
TP53I3	19	32	15	29	8	12
	21	27	16	3	13	10
TP53INP1	83	353	88	275	135	148
	223	403	154	95	173	93
TP53INP2	39	326	30	120	140	137
	402	215	67	85	149	45
TP53RK	32	79	39	94	55	108
	89	82	96	23	65	11
TP53TG3	15	0	7	0	1	0
	0	0	6	0	7	0
TP53TG3B	19	0	5	0	2	0
	0	0	0	0	7	0
TP53TG3C	3	0	0	0	0	0
	0	0	2	0	0	0
TP53TG5	8	0	2	0	0	0
	1	4	4	0	2	0
TP63	117	312	219	234	150	221
	290	504	229	199	273	118
TP73	29	1	14	0	2	0
	1	2	9	0	4	0
TPBG	15	14	10	35	9	11
	16	22	19	6	22	1
TPBGL	1	0	6	0	3	0
	0	0	2	0	2	0
TPCN1	107	409	81	259	232	361
	291	326	202	118	233	78
TPCN2	33	49	25	31	24	36
	26	45	25	10	28	14
TPD52	48	206	55	158	99	143
	167	180	74	33	81	22
TPD52L1	101	465	91	220	218	172
	371	786	209	144	297	104
TPD52L2	81	319	63	213	124	156
	239	344	102	72	142	40
TPD52L3	31	0	15	0	8	0
	0	0	13	0	8	0
TPGS1	0	1	2	2	0	4
	6	2	3	0	3	1
TPGS2	36	37	27	33	25	31
	28	44	18	15	32	12

TPH1	13	5	13	8	7	2
	1	13	10	5	16	0
TPH2	19	0	9	0	1	1
	0	0	6	0	4	0
TPI1	1281	8500	1082	7258	2366	6611
	7150	7422	3919	1503	3853	732
TPK1	22	49	16	29	10	25
	18	41	29	6	36	10
TPM1	3729	43711	6720	35785	16641	45105
	43978	35611	24074	10753	26754	3093
TPM2	15090	84357	14220	46955	41692	81668
	75088	79004	36727	31054	52578	12735
TPM3	13762	48540	9430	17586	24653	24785
	56068	74021	12731	21121	25853	11618
TPM4	69	310	70	209	150	179
	180	327	150	110	157	67
TPMT	54	192	39	129	92	150
	183	248	118	55	118	49
TPO	33	25	15	16	18	11
	36	42	24	22	23	6
TPP1	32	106	32	57	52	74
	88	67	33	34	41	15
TPP2	314	1459	306	963	669	1026
	1073	1678	747	437	978	287
TPPP	35	116	55	90	56	80
	70	110	47	32	43	20
TPPP2	8	6	6	2	4	8
	0	1	5	5	7	1
TPPP3	22	107	31	40	81	85
	53	209	35	40	64	26
TPR	373	1786	476	1246	1105	1189
	1202	1744	669	717	1176	438
TPRA1	34	127	39	67	55	114
	109	173	78	32	79	19
TPRG1	30	39	31	16	43	28
	35	59	27	153	22	7
TPRG1L	38	252	33	182	81	141
	241	241	78	48	106	31
TPRKB	50	178	22	152	77	134
	247	396	86	67	121	36
TPRN	14	16	6	11	8	15
	5	17	4	8	10	3
TPRX1	4	0	1	0	0	0
	0	0	1	0	2	0
TPSAB1	14	2	5	14	8	5
	3	12	8	4	0	5
TPSB2	0	7	2	7	2	10
	3	40	8	0	3	1
TPSD1	0	0	0	0	0	3
	0	0	4	0	0	2
TPSG1	3	2	5	0	1	4
	0	0	1	0	3	0
TPST1	17	69	8	43	32	35
	39	47	33	10	19	9
TPST2	35	122	33	95	89	112
	117	178	67	29	77	21
TPT1	6830	36548	7145	32339	15084	28476
	43508	60651	21245	10345	21074	5799

TPTE	45	0	21	4	12	2
	2	3	25	1	24	1
TPTE2	26	13	17	3	7	2
	9	4	27	3	7	1
TPX2	33	14	14	13	20	14
	12	20	25	13	17	2
TRA2A	32	236	39	132	79	124
	150	198	58	65	84	46
TRA2B	102	470	113	352	243	368
	400	626	316	163	353	109
TRABD	11	46	11	23	13	38
	41	39	35	15	38	11
TRABD2A	12	0	3	0	4	0
	0	0	8	1	6	0
TRABD2B	42	10	19	7	8	2
	9	10	24	0	8	3
TRADD	5	5	2	9	4	11
	5	9	5	3	7	1
TRAF1	26	42	18	28	15	24
	11	40	26	12	23	7
TRAF2	14	47	14	20	13	24
	13	27	14	6	15	8
TRAF3	35	105	33	91	60	101
	57	88	37	20	57	15
TRAF3IP1	61	244	46	153	85	221
	126	230	111	66	139	47
TRAF3IP2	55	50	32	26	30	46
	42	68	40	23	28	16
TRAF3IP3	25	14	9	6	6	6
	0	10	18	1	16	2
TRAF4	12	5	11	4	1	4
	3	2	4	1	2	0
TRAF5	38	62	28	74	36	54
	45	93	48	16	35	15
TRAF6	71	260	78	155	155	163
	173	338	133	84	147	69
TRAF7	24	58	17	43	27	62
	48	80	36	30	34	17
TRAFD1	34	126	42	98	82	126
	112	145	64	35	82	24
TRAIP	22	19	9	11	13	12
	11	25	14	4	13	3
TRAK1	441	2304	379	1402	989	1584
	1661	1825	861	678	1175	353
TRAK2	139	660	168	429	229	464
	539	978	301	190	390	111
TRAM1	89	418	93	368	207	351
	307	556	220	122	238	70
TRAM1L1	8	6	11	6	3	3
	2	3	13	1	6	2
TRAM2	55	114	34	78	57	68
	72	94	64	23	50	15
TRANK1	70	99	65	87	62	60
	35	74	58	26	47	10
TRAP1	174	958	169	638	408	597
	733	904	469	351	579	181
TRAPPC1	7	35	3	22	11	22
	42	28	16	13	21	4

TRAPPC10	104	343	75	251	180	235
	268	369	188	81	187	64
TRAPPC11	161	715	182	466	346	576
	612	913	350	233	469	135
TRAPPC12	51	282	40	133	104	182
	128	251	101	68	124	45
TRAPPC2	40	77	20	51	34	53
	61	86	31	17	44	8
TRAPPC2L	22	131	21	105	38	93
	159	207	81	32	64	18
TRAPPC3	45	155	27	119	88	115
	190	274	72	48	91	44
TRAPPC4	29	115	23	115	48	127
	102	206	88	28	50	25
TRAPPC5	43	180	14	215	73	110
	164	294	111	47	86	22
TRAPPC6A	6	15	6	10	4	5
	13	13	7	1	3	2
TRAPPC6B	84	480	94	369	217	406
	373	567	228	136	265	72
TRAPPC8	202	775	190	563	452	505
	578	846	370	236	523	142
TRAPPC9	94	411	83	259	216	304
	249	324	167	96	199	70
TRAT1	13	5	7	5	5	3
	2	3	13	1	4	1
TRDMT1	64	116	68	78	59	68
	92	125	88	26	64	21
TRDN	5587	39530	6570	28141	15156	26971
	33820	35333	13916	10396	17861	5567
TREH	4	0	7	0	2	0
	0	0	4	0	0	0
TREM1	16	1	16	3	4	3
	0	6	11	1	8	0
TREM2	5	0	2	0	0	1
	0	0	0	0	2	0
TREML1	9	2	4	0	1	2
	1	0	4	0	2	0
TREML2	53	60	25	21	26	28
	26	46	17	18	34	9
TREML4	17	0	8	0	3	0
	0	0	7	0	4	0
TRERF1	65	48	31	35	35	48
	27	43	32	10	28	4
TREX1	11	11	10	10	14	8
	14	13	12	4	17	4
TREX2	2	2	0	0	1	0
	0	0	1	0	0	0
TRH	8	0	4	0	0	0
	0	0	3	0	4	1
TRHDE	44	16	27	13	29	26
	31	52	45	25	48	10
TRHR	12	1	7	1	1	0
	0	0	3	0	0	2
TRIAP1	67	315	53	225	135	257
	372	407	174	75	166	39
TRIB1	119	658	87	485	369	450
	725	676	293	224	302	112

TRIB2	45	172	35	126	72	95
	131	190	106	42	66	35
TRIB3	5	4	1	4	3	15
	1	0	3	2	5	1
TRIL	31	69	18	62	57	72
	58	53	42	20	32	5
TRIM10	24	0	10	0	1	0
	1	0	10	0	2	0
TRIM11	5	19	10	10	5	18
	5	9	11	4	14	2
TRIM13	80	292	77	260	174	232
	303	352	191	110	217	61
TRIM14	32	17	12	3	13	10
	14	19	9	7	9	6
TRIM15	13	0	5	0	3	0
	0	0	4	0	2	0
TRIM16	68	149	39	65	91	78
	110	136	62	43	66	20
TRIM16L	27	153	27	43	81	85
	102	86	56	29	29	11
TRIM17	6	3	5	1	2	0
	0	1	3	0	3	0
TRIM2	73	190	70	195	112	183
	156	231	145	70	137	44
TRIM21	19	42	20	21	24	31
	30	39	25	25	37	5
TRIM22	60	239	60	205	135	143
	139	353	128	166	214	64
TRIM23	130	456	117	375	225	299
	422	574	303	130	350	88
TRIM24	80	269	67	200	151	137
	210	240	110	78	136	47
TRIM25	75	228	54	134	111	158
	172	192	81	66	119	37
TRIM26	37	91	24	56	37	41
	64	89	37	33	45	13
TRIM27	29	101	39	74	29	88
	72	117	34	32	69	27
TRIM28	64	208	45	119	93	172
	181	235	117	63	141	33
TRIM29	10	1	4	1	1	0
	0	0	5	0	4	0
TRIM3	20	28	19	27	25	27
	29	34	20	23	31	6
TRIM31	13	0	5	0	1	0
	0	0	3	0	4	0
TRIM32	52	291	90	230	142	223
	194	221	123	62	158	37
TRIM33	168	760	208	678	391	624
	687	860	396	252	421	152
TRIM34	1	5	5	5	0	5
	18	12	2	5	1	3
TRIM35	36	79	37	62	37	86
	73	94	48	26	33	26
TRIM36	31	6	18	1	6	3
	0	6	11	0	10	3
TRIM37	87	371	85	259	173	248
	288	355	177	105	179	67

TRIM38	69	164	81	158	73	84
	120	223	89	71	98	39
TRIM39	26	83	27	61	36	50
	68	89	68	27	47	2
TRIM39-RPP21		15	17	11	10	14
	13	1	22	7	5	5
	3					
TRIM4	52	87	22	67	45	73
	78	136	61	27	82	21
TRIM40	11	0	3	0	1	0
	0	0	3	0	2	0
TRIM41	36	107	34	75	52	95
	101	122	51	30	53	21
TRIM42	16	0	10	0	6	0
	0	0	14	0	4	1
TRIM43	30	0	19	1	7	1
	0	0	10	0	13	0
TRIM43B	25	0	15	0	4	0
	0	0	10	0	11	0
TRIM44	66	358	80	242	159	220
	269	370	160	112	161	86
TRIM45	121	318	88	246	191	356
	198	257	175	99	190	38
TRIM46	8	3	9	1	2	0
	0	0	7	0	1	0
TRIM47	10	20	9	18	9	10
	8	18	11	9	16	5
TRIM48	9	0	2	0	2	1
	0	0	8	0	2	0
TRIM49	21	0	5	0	6	0
	0	0	12	0	9	0
TRIM49B	13	0	9	0	2	0
	0	0	5	0	3	0
TRIM49C	17	0	11	0	4	0
	0	0	9	0	9	0
TRIM49DP	0	0	2	0	3	0
	0	0	3	0	5	0
TRIM49L1	22	1	14	0	3	0
	0	0	9	0	4	0
TRIM5	43	50	24	41	18	31
	41	49	33	15	34	16
TRIM50	8	5	3	3	3	1
	0	0	7	2	3	0
TRIM51	19	0	10	0	4	0
	0	0	6	0	4	0
TRIM51GP	7	0	7	0	3	0
	0	0	7	0	1	0
TRIM52	27	119	20	57	55	90
	100	128	57	46	59	22
TRIM54	683	3250	580	1747	1492	2156
	2548	3194	1106	955	1697	461
TRIM55	119	493	179	334	228	294
	354	450	264	210	345	122
TRIM56	25	60	16	59	41	44
	25	81	35	21	40	14
TRIM58	27	71	13	5	7	20
	24	7	10	2	13	1

TRIM59	20	9	7	6	5	4
	1	7	6	4	4	1
TRIM6	1	4	3	1	1	3
	1	8	0	2	2	1
TRIM6-TRIM34		28	21	16	8	8
	8	6	7	21	7	13
	4					
TRIM60	10	1	3	0	2	1
	0	2	1	0	1	0
TRIM61	15	3	7	1	5	0
	3	1	9	1	3	0
TRIM62	9	14	6	8	3	13
	10	10	2	2	8	7
TRIM63	288	1078	189	856	820	1099
	1910	1548	840	611	1263	387
TRIM64	1	0	0	0	0	0
	0	0	0	0	1	0
TRIM64B	23	0	18	0	13	0
	0	0	7	0	6	0
TRIM64C	10	0	6	0	2	0
	0	0	3	0	8	0
TRIM65	21	68	18	48	34	74
	38	87	42	25	55	17
TRIM66	78	186	33	84	69	84
	79	99	68	32	43	25
TRIM67	29	0	17	1	8	0
	0	0	15	3	8	0
TRIM68	30	52	22	60	39	63
	65	48	36	15	47	9
TRIM69	27	52	29	47	18	37
	11	28	13	9	43	5
TRIM7	205	1008	171	760	468	748
	925	1012	439	302	545	157
TRIM71	10	1	6	2	0	0
	0	0	6	0	1	0
TRIM72	112	492	92	296	236	329
	320	608	248	195	332	86
TRIM73	2	2	3	3	5	2
	0	0	4	1	8	0
TRIM74	8	6	11	9	1	4
	2	2	6	2	0	0
TRIM77P	17	0	11	0	10	1
	0	0	13	0	7	0
TRIM8	91	336	68	249	187	246
	305	334	136	112	160	74
TRIM9	41	4	27	4	8	8
	6	8	17	2	14	1
TRIML1	10	0	8	0	0	0
	0	0	4	0	2	0
TRIML2	14	0	3	0	0	0
	0	0	6	0	3	0
TRIO	208	1033	315	658	535	798
	803	1280	588	316	716	259
TRIOBP	106	207	63	144	68	149
	126	203	89	53	114	54
TRIP10	266	1233	201	851	640	1188
	1301	1320	634	546	852	254

TRIP11	315	1684	346	1139	888	1215
	1114	1537	631	587	803	362
TRIP12	461	2909	616	1947	1222	1869
	1847	2259	1053	728	1185	414
TRIP13	23	2	8	5	3	2
	3	5	11	1	5	0
TRIP4	42	127	12	65	34	29
	94	90	41	28	45	15
TRIP6	13	29	11	22	15	32
	19	44	21	11	18	6
TRIQK	32	57	28	73	40	52
	38	52	40	21	48	7
TRIT1	34	111	35	77	57	93
	127	161	85	39	98	20
TRMT1	24	81	8	45	29	39
	47	94	39	24	35	12
TRMT10A	39	66	31	63	42	52
	48	81	50	22	49	17
TRMT10B	25	38	27	36	21	26
	48	40	28	12	22	6
TRMT10C	61	333	56	238	156	215
	402	568	131	101	236	51
TRMT11	55	209	67	169	97	157
	169	268	160	69	151	30
TRMT112	53	295	59	217	123	222
	282	569	181	86	148	43
TRMT12	29	115	19	92	58	98
	110	120	64	50	77	18
TRMT13	97	341	80	183	199	395
	269	533	162	110	248	70
TRMT1L	131	634	119	447	358	429
	367	602	243	178	343	117
TRMT2A	34	93	17	42	45	58
	57	105	41	17	54	17
TRMT2B	44	201	31	141	107	164
	150	180	101	59	126	23
TRMT44	30	85	23	68	38	73
	52	102	44	18	51	16
TRMT5	56	167	53	112	107	139
	132	155	71	53	126	32
TRMT6	37	112	23	90	54	126
	82	145	50	32	50	22
TRMT61A	4	10	4	9	6	10
	11	20	8	3	12	1
TRMT61B	25	135	23	71	48	74
	99	133	57	26	73	30
TRMU	40	105	17	63	38	85
	75	106	59	31	55	13
TRNAU1AP	32	78	32	50	42	64
	73	124	51	32	53	27
TRNP1	3	3	2	2	0	3
	3	3	2	2	1	3
TRNT1	38	105	29	55	55	74
	64	151	55	34	53	24
TRO	38	53	20	25	8	30
	15	35	24	7	19	8
TROAP	13	0	10	0	3	0
	1	0	4	0	3	0

TROVE2	104	531	124	455	309	392
	440	627	236	140	345	112
TRPA1	32	1	43	1	18	1
	0	5	43	1	17	0
TRPC1	36	74	39	93	46	54
	54	78	61	12	65	9
TRPC3	25	4	18	5	7	4
	5	17	17	0	15	2
TRPC4	23	5	15	2	12	1
	4	4	14	2	9	0
TRPC4AP	121	418	112	378	210	359
	305	431	243	161	292	100
TRPC5	29	0	12	0	6	0
	0	0	2	0	6	0
TRPC5OS	11	0	9	0	4	0
	0	1	4	0	5	0
TRPC6	40	18	31	13	16	4
	22	9	27	4	18	6
TRPC7	18	0	18	0	2	0
	0	0	13	0	5	0
TRPM1	53	0	36	0	10	0
	1	0	27	0	11	0
TRPM2	33	13	17	9	8	9
	14	12	31	2	12	3
TRPM3	54	4	32	3	12	1
	2	6	43	1	19	4
TRPM4	33	122	29	46	39	53
	96	97	47	36	43	18
TRPM5	14	0	15	0	3	0
	0	1	2	0	2	0
TRPM6	67	9	50	6	26	9
	4	8	46	2	31	1
TRPM7	248	875	274	658	393	573
	671	1124	431	284	521	201
TRPM8	54	1	36	0	11	1
	0	1	32	0	15	0
TRPS1	174	556	144	541	278	506
	399	564	314	189	347	106
TRPT1	51	230	42	336	75	265
	487	404	195	94	118	30
TRPV1	53	98	28	65	39	52
	68	109	68	29	47	29
TRPV2	25	18	9	11	21	9
	8	15	14	8	14	4
TRPV3	52	63	32	13	24	12
	39	101	48	15	32	4
TRPV4	17	1	11	1	4	1
	0	1	12	0	4	0
TRPV5	26	0	13	1	4	3
	4	2	13	3	10	0
TRPV6	23	0	16	1	1	3
	1	1	5	0	6	0
TRRAP	200	798	210	490	354	487
	618	662	360	249	438	180
TRUB1	56	247	68	244	113	198
	273	238	194	77	140	29
TRUB2	29	79	22	58	44	69
	76	118	42	28	70	12

TSACC	6	0	3	0	2	1
	3	1	3	0	2	0
TSC1	236	841	196	610	397	580
	760	1142	477	325	664	190
TSC2	77	273	58	161	96	173
	227	275	133	102	161	47
TSC22D1	428	1815	401	1155	1202	1398
	3107	3674	1265	896	2147	763
TSC22D2	41	208	49	165	102	183
	140	199	70	55	116	42
TSC22D3	169	330	77	398	241	417
	515	776	363	148	370	174
TSC22D4	10	32	5	10	13	17
	24	18	10	11	9	8
TSEN15	65	268	50	158	96	176
	224	331	141	55	127	39
TSEN2	48	121	38	63	54	94
	84	145	64	38	72	18
TSEN34	24	26	15	41	24	41
	47	65	28	6	32	8
TSEN54	17	11	9	14	5	14
	8	21	9	3	7	3
TSFM	75	308	51	207	158	177
	360	407	178	85	191	81
TSG101	58	356	62	259	129	257
	408	422	190	122	234	64
TSGA10	38	8	34	23	18	29
	16	13	43	3	11	2
TSGA10IP	7	0	4	1	1	0
	0	0	1	0	0	1
TSGA13	20	0	10	0	1	1
	0	0	11	0	2	0
TSHB	3	0	5	0	2	0
	0	0	0	0	3	0
TSHR	41	11	23	4	10	3
	3	19	22	1	23	2
TSHZ1	64	209	62	175	103	171
	152	220	107	45	102	41
TSHZ2	74	127	59	80	69	67
	72	133	80	23	72	18
TSHZ3	47	49	26	48	26	26
	32	40	25	15	24	13
TSKS	11	1	4	2	1	1
	1	1	8	1	1	0
TSKU	7	8	4	12	5	4
	3	5	10	3	4	4
TSLP	17	8	11	17	16	11
	16	12	24	7	13	5
TSN	131	731	116	616	302	518
	560	822	345	182	481	127
TSNARE1	9	26	8	15	15	23
	15	45	16	14	14	5
TSNAX	120	557	118	451	270	447
	497	935	307	225	499	151
TSNAXIP1	23	6	7	2	6	0
	1	0	15	2	1	0
TSPAN1	12	2	3	1	6	0
	0	0	8	2	3	0

TSPAN10	2	0	0	0	0	0
	0	0	1	0	0	0
TSPAN11	26	4	19	7	7	1
	3	4	17	1	9	4
TSPAN12	22	47	9	40	20	38
	25	34	28	12	28	3
TSPAN13	24	105	40	40	37	44
	79	76	53	30	52	14
TSPAN14	37	134	24	81	62	45
	97	109	44	51	60	17
TSPAN15	38	65	25	65	35	87
	40	56	44	10	19	13
TSPAN16	4	0	3	0	6	0
	0	0	2	0	0	0
TSPAN17	31	124	29	88	47	92
	93	71	42	41	46	20
TSPAN18	37	85	21	54	30	41
	65	54	39	24	39	22
TSPAN19	9	0	6	0	4	2
	1	4	5	0	11	0
TSPAN2	25	43	16	30	22	17
	52	36	30	27	30	9
TSPAN3	396	2823	584	2759	1122	2476
	2225	2572	1367	649	1329	370
TSPAN31	32	111	22	92	58	97
	87	148	75	33	64	20
TSPAN32	9	0	4	1	1	1
	0	2	1	1	0	0
TSPAN33	30	82	26	59	19	59
	76	82	38	27	33	8
TSPAN4	6	31	3	24	18	26
	19	36	26	7	14	3
TSPAN5	22	25	12	36	3	26
	18	27	49	3	40	2
TSPAN6	26	66	28	61	23	53
	100	79	56	21	51	15
TSPAN7	158	556	114	457	222	430
	452	848	345	225	476	109
TSPAN8	57	121	17	130	83	76
	208	365	191	41	250	60
TSPAN9	56	156	24	102	66	117
	113	75	89	30	69	9
TSPEAR	16	1	16	0	4	1
	0	0	3	0	5	0
TSPO	7	24	7	15	15	8
	23	39	19	9	15	7
TSPO2	4	0	0	0	1	0
	0	0	3	0	1	0
TSPY1	0	0	17	0	0	0
	0	0	7	0	1	0
TSPY10	0	0	13	0	0	0
	0	0	7	0	4	0
TSPY2	0	0	6	1	0	0
	0	0	4	0	1	0
TSPY3	0	0	6	0	0	0
	0	0	2	0	6	0
TSPY4	0	0	11	0	0	0
	0	0	4	0	2	0

TSPY8	0	0	6	0	0	0
	0	0	7	0	3	0
TSPYL1	354	2319	261	1910	801	1370
	1452	1994	771	503	1118	302
TSPYL2	63	197	78	217	132	290
	183	286	76	93	150	77
TSPYL4	23	66	29	53	40	53
	53	56	44	17	36	6
TSPYL5	48	278	58	115	153	151
	144	167	104	70	158	50
TSPYL6	14	4	10	4	7	4
	1	9	14	2	3	0
TSR1	188	779	154	633	293	576
	459	635	308	233	445	134
TSR2	39	187	45	122	60	123
	166	174	56	60	89	16
TSR3	42	237	37	155	73	180
	240	268	132	90	110	34
TSSC1	29	84	14	57	24	38
	46	60	32	13	36	13
TSSC4	13	69	14	52	27	47
	52	99	31	23	30	12
TSSK2	5	2	3	0	1	0
	0	1	1	0	2	1
TSSK3	1	4	1	2	1	3
	2	2	3	0	6	0
TSSK4	6	2	5	1	4	1
	1	0	4	0	2	0
TSSK6	2	3	0	0	2	0
	3	0	2	0	0	0
TST	8	17	4	8	11	8
	34	62	17	4	12	7
TSTA3	22	39	9	28	18	36
	37	99	19	18	34	6
TSTD1	8	121	21	38	27	39
	94	68	35	14	17	11
TSTD2	104	497	122	353	209	387
	326	427	205	93	212	68
TSTD3	34	83	29	89	32	46
	54	86	59	30	63	18
TTBK1	30	0	17	0	2	0
	1	0	8	1	4	0
TTBK2	140	525	129	387	255	410
	337	521	244	164	290	97
TTC1	89	493	108	376	256	441
	397	593	258	131	258	77
TTC12	43	72	41	59	27	50
	58	134	45	34	63	15
TTC13	64	179	66	118	70	162
	94	175	99	51	129	29
TTC14	108	436	100	325	226	326
	266	512	238	143	252	100
TTC16	12	1	12	0	4	0
	0	2	5	0	3	0
TTC17	189	725	203	526	424	560
	629	1014	427	284	505	142
TTC18	67	92	49	62	49	109
	61	102	58	28	70	26

TTC19	103	472	95	383	207	367
	444	506	211	123	274	76
TTC21A	26	10	35	11	6	4
	12	14	22	2	20	1
TTC21B	185	617	179	483	372	471
	530	734	433	281	473	140
TTC22	13	1	7	0	3	0
	0	4	1	1	4	0
TTC23	67	130	34	80	84	95
	57	165	81	36	70	32
TTC23L	17	5	11	3	7	0
	0	1	10	0	4	0
TTC24	9	2	1	1	1	2
	0	2	7	0	3	0
TTC25	16	8	17	3	7	17
	28	9	11	11	11	0
TTC26	19	12	8	18	12	6
	13	20	13	7	33	1
TTC27	46	195	56	138	115	149
	216	257	124	67	156	35
TTC28	127	333	106	305	204	278
	279	319	206	124	201	65
TTC29	34	0	11	0	7	0
	0	1	21	0	6	0
TTC3	600	3384	805	2469	1618	2402
	1827	2248	1157	936	1162	589
TTC30A	47	80	29	69	39	60
	47	86	46	34	57	15
TTC30B	11	27	8	25	13	8
	6	19	16	1	22	4
TTC31	34	67	25	54	60	52
	56	89	47	22	45	20
TTC32	12	71	13	56	23	66
	69	111	53	18	47	24
TTC33	134	690	154	527	331	479
	592	711	328	185	408	118
TTC34	3	0	1	0	2	0
	0	0	0	0	0	0
TTC36	3	5	2	0	1	2
	2	3	0	1	0	0
TTC37	488	2428	536	1670	1240	1845
	1374	2118	1071	758	1351	450
TTC38	49	110	19	69	71	105
	99	188	70	36	73	23
TTC39A	26	5	9	7	4	6
	17	8	11	2	12	2
TTC39B	32	80	37	52	62	65
	18	54	51	27	39	8
TTC39C	33	13	16	10	10	6
	1	14	16	4	11	6
TTC4	81	268	60	144	128	159
	196	304	158	69	186	59
TTC40	68	74	33	60	24	80
	66	101	73	27	74	10
TTC5	34	89	31	74	37	77
	79	132	52	26	65	15
TTC6	6	0	3	0	2	1
	0	0	11	0	2	0

TTC7A	31	49	30	32	28	72
	23	66	54	12	36	15
TTC7B	89	282	93	288	180	245
	374	189	247	98	147	53
TTC8	28	44	23	40	23	43
	37	30	32	9	39	6
TTC9	21	101	35	62	73	71
	48	63	38	26	40	14
TTC9B	2	0	0	0	0	0
	0	0	1	0	0	0
TTC9C	15	46	18	25	11	36
	42	68	29	19	30	12
TTF1	56	128	37	94	65	103
	92	121	67	47	68	34
TTF2	49	61	37	35	34	55
	39	60	61	19	54	9
TTI1	62	199	52	116	109	163
	178	274	111	66	140	43
TTI2	30	72	25	68	45	72
	78	93	55	22	48	14
TTK	28	2	28	0	12	2
	3	0	29	0	23	0
TTL	162	760	124	608	288	576
	569	632	250	199	336	95
TTLL1	31	15	15	11	11	11
	14	24	19	3	17	3
TTLL10	5	2	4	0	1	0
	1	1	2	1	7	0
TTLL11	31	200	38	96	109	138
	88	121	115	46	77	19
TTLL12	23	39	13	19	17	27
	26	37	21	10	29	10
TTLL13	22	0	3	2	5	3
	0	1	7	0	2	0
TTLL2	16	0	5	0	2	0
	0	0	4	0	1	0
TTLL3	5	26	4	31	1	31
	45	28	15	20	16	13
TTLL4	41	41	35	36	23	51
	45	35	40	16	29	5
TTLL5	74	157	62	100	87	111
	116	159	79	73	114	30
TTLL6	25	0	20	0	8	0
	0	2	13	0	9	0
TTLL7	73	186	53	151	114	186
	124	155	119	70	123	46
TTLL8	7	0	2	0	1	0
	0	0	2	0	4	0
TTLL9	14	0	5	0	4	0
	0	0	5	0	3	0
TTN	718865	4990408	809344	3789528	2580470	3313500
	3359775	4080411	1801097	1717975	2813117	973933
TTPA	10	1	13	4	6	2
	6	3	10	0	9	1
TTPAL	33	54	20	48	20	46
	38	77	47	24	35	17
TTR	9	0	1	0	0	0
	1	0	4	0	2	1

TTYH1	4	3	4	2	0	0
	0	0	1	0	6	0
TTYH2	55	141	52	89	84	106
	114	162	67	47	71	30
TTYH3	11	11	13	16	6	9
	3	13	10	4	6	5
TUB	44	46	45	31	25	43
	26	47	34	13	39	16
TUBA1A	37	138	34	105	53	95
	111	155	85	44	68	25
TUBA1B	60	353	73	257	196	240
	350	679	219	141	186	81
TUBA1C	22	89	17	32	18	53
	46	59	45	15	21	13
TUBA3C	14	0	2	0	1	0
	0	0	3	0	1	0
TUBA3D	10	0	6	1	0	0
	0	0	2	1	5	0
TUBA3E	13	0	3	0	2	0
	0	1	2	0	1	0
TUBA4A	184	1128	148	548	638	680
	1175	998	334	349	379	164
TUBA8	227	1216	167	792	480	1065
	1059	910	441	310	441	100
TUBAL3	17	0	3	0	2	0
	3	4	5	0	5	0
TUBB	140	960	213	587	489	687
	705	986	381	295	371	172
TUBB1	20	13	8	3	3	6
	0	1	5	1	5	1
TUBB2A	15	45	13	26	22	29
	14	32	28	7	25	15
TUBB2B	12	30	5	11	9	19
	31	30	25	17	18	8
TUBB3	6	0	1	0	0	0
	0	1	1	0	3	1
TUBB4A	11	2	5	5	4	1
	1	4	4	0	3	1
TUBB4B	54	437	54	305	244	259
	311	217	111	64	96	44
TUBB6	18	97	7	46	33	39
	48	70	34	25	35	22
TUBB8	3	0	4	0	1	0
	0	0	1	0	0	0
TUBD1	29	82	25	41	44	69
	59	106	56	26	56	21
TUBE1	40	122	27	103	65	105
	141	200	77	46	107	24
TUBG1	26	151	36	99	59	106
	142	133	43	42	55	23
TUBG2	20	37	27	28	17	27
	29	32	28	8	33	11
TUBGCP2	59	287	55	160	108	175
	165	288	127	100	173	52
TUBGCP3	95	314	94	204	118	168
	181	260	130	67	161	48
TUBGCP4	38	112	47	49	44	48
	100	121	39	46	46	25

TUBGCP5	98	322	60	201	147	185
	225	375	169	117	229	66
TUBGCP6	70	250	67	175	96	228
	230	333	146	74	154	44
TUFM	174	994	157	652	390	689
	1097	1234	554	277	609	216
TUFT1	38	48	18	30	29	48
	18	32	14	13	19	6
TULP1	18	1	11	0	5	2
	0	0	6	0	3	0
TULP2	14	0	5	0	2	0
	0	0	6	0	5	0
TULP3	52	110	44	49	44	112
	73	148	54	47	73	39
TULP4	200	1141	452	1774	388	1189
	713	661	524	251	487	181
TUSC1	9	12	11	23	9	16
	19	25	8	7	8	1
TUSC2	28	79	22	98	48	77
	102	100	53	27	44	22
TUSC3	19	84	16	38	36	29
	71	87	34	15	35	16
TUSC5	20	10	11	8	10	4
	9	8	1	4	8	20
TUT1	28	69	17	36	20	39
	63	56	26	22	38	10
TWF1	53	166	63	164	85	137
	151	175	125	50	114	41
TWF2	112	401	41	203	185	224
	259	428	134	105	207	76
TWIST1	7	2	1	4	0	0
	0	3	1	1	1	1
TWIST2	4	2	2	1	3	0
	1	0	7	0	0	0
TWISTNB	64	259	63	207	122	187
	240	312	163	78	198	52
TWSG1	21	102	26	138	72	92
	77	126	48	30	75	15
TXK	19	17	21	6	8	15
	8	8	21	4	15	1
TXLNA	102	388	94	271	166	309
	303	330	147	124	189	60
TXLNB	1743	7056	1705	5768	4664	6481
	6631	10474	3951	3514	6506	1974
TXLNG	67	101	40	81	101	70
	117	111	50	57	68	25
TXN	41	184	44	171	61	139
	143	241	114	46	94	23
TXN2	53	238	26	154	92	126
	290	232	92	71	134	32
TXNDC11	41	183	33	146	76	124
	163	201	91	67	101	46
TXNDC12	40	154	31	125	74	111
	150	211	81	54	88	15
TXNDC15	123	489	125	468	236	393
	338	497	279	196	304	77
TXNDC16	57	154	73	99	90	112
	161	304	103	47	150	34

TXNDC17	21	55	30	31	19	44
	64	56	34	22	36	8
TXNDC2	11	0	10	0	5	0
	0	0	4	0	2	1
TXNDC5	44	189	48	146	134	180
	148	281	119	79	107	54
TXNDC8	1	0	2	0	0	0
	0	0	5	0	1	0
TXNDC9	41	177	51	138	84	150
	148	259	95	55	104	22
TXNIP	3270	7691	5345	5886	6819	10569
	16926	31236	9754	6495	10754	4812
TXNL1	176	1112	175	640	475	915
	938	1206	412	281	497	93
TXNL4A	47	180	32	166	75	158
	211	224	87	54	92	26
TXNL4B	18	58	28	42	36	52
	56	80	37	19	44	20
TXNRD1	289	1239	246	1058	669	885
	1030	1329	562	374	630	208
TXNRD2	19	52	19	50	28	30
	39	76	30	22	36	11
TXNRD3	51	169	42	136	76	93
	127	124	77	45	98	16
TXNRD3NB	10	18	7	10	4	6
	3	9	11	3	8	2
TYK2	33	97	27	55	44	77
	61	103	58	33	75	22
TYMP	9	26	7	17	11	20
	9	33	15	21	19	8
TYMS	11	6	5	8	2	1
	0	4	6	3	8	0
TYR	10	0	8	0	2	0
	0	0	12	0	2	0
TYRO3	39	15	22	18	20	8
	12	19	14	6	17	10
TYROBP	8	11	5	11	10	16
	15	15	11	1	5	3
TYRP1	32	30	9	12	34	9
	19	90	13	13	28	4
TYSND1	14	33	8	11	23	16
	28	43	9	11	16	4
TYW1	67	269	71	206	114	236
	156	290	144	66	142	57
TYW1B	45	102	44	44	44	110
	45	78	67	26	52	15
TYW3	36	207	54	175	112	169
	145	247	108	74	123	32
TYW5	37	125	29	94	50	78
	96	151	63	27	75	32
U2AF1	19	160	36	69	55	83
	185	195	63	38	81	21
U2AF1L4	9	41	6	16	21	15
	30	44	16	12	23	4
U2AF2	66	240	47	139	105	176
	182	251	94	62	137	47
U2SURP	329	1432	404	991	868	1033
	964	1584	673	525	1001	254

UACA	1127	6889	1445	5060	2868	4343
	5322	5887	2972	2336	3817	1038
UAP1	67	367	62	285	238	338
	405	650	236	121	311	89
UAP1L1	19	11	8	9	6	10
	13	18	14	2	3	6
UBA1	116	618	101	369	331	368
	360	481	220	189	262	105
UBA2	266	1035	213	896	495	827
	861	1262	592	386	704	239
UBA3	117	501	148	442	264	547
	501	826	293	218	329	100
UBA5	85	408	95	329	179	263
	363	421	180	111	256	78
UBA52	305	1352	253	1152	600	1056
	1657	2631	827	519	795	282
UBA6	172	532	139	409	302	416
	419	644	326	173	306	103
UBA7	52	112	24	82	54	70
	67	116	73	36	69	22
UBAC1	160	923	136	653	317	659
	837	1081	423	266	524	161
UBAC2	108	429	63	296	226	254
	335	521	172	125	245	59
UBAP1	90	385	81	308	194	332
	313	377	153	112	228	57
UBAP1L	2	19	5	14	7	5
	5	22	14	3	7	5
UBAP2	153	678	198	421	342	495
	547	778	298	217	473	147
UBAP2L	141	631	155	360	263	434
	476	638	244	220	360	139
UBASH3A	28	1	12	1	2	1
	0	0	9	0	5	0
UBASH3B	45	32	33	33	30	83
	17	17	42	5	24	4
UBB	1174	5028	564	3832	2778	4015
	5755	9411	3592	1220	3742	677
UBC	3363	12975	3445	12518	9099	14896
	15439	14204	7927	6136	10276	3233
UBD	6	2	6	1	3	0
	3	2	5	0	6	1
UBE2A	87	441	63	291	186	343
	380	524	190	109	232	72
UBE2B	186	1437	130	1048	489	720
	1083	1547	475	274	676	160
UBE2C	10	1	6	1	6	2
	4	0	3	0	2	0
UBE2D1	388	1572	277	1490	915	1386
	2135	3589	1277	698	1838	520
UBE2D2	112	557	108	415	248	503
	592	560	286	164	313	80
UBE2D3	342	1873	343	1255	960	1504
	1651	2172	844	602	1221	411
UBE2D4	191	960	136	786	379	789
	1002	952	396	298	444	164
UBE2E1	115	449	91	292	236	413
	348	603	229	111	280	64

UBE2E2	20	80	23	57	40	52
	56	88	39	16	49	14
UBE2E3	127	1011	129	1092	294	530
	819	569	294	148	343	90
UBE2F	66	317	47	227	80	223
	301	311	117	76	151	37
UBE2G1	590	3041	639	2296	1668	2660
	3880	4372	2109	1080	2644	631
UBE2G2	173	920	142	624	374	638
	681	989	405	267	452	156
UBE2H	399	1923	388	1479	910	1222
	1671	2239	786	471	983	348
UBE2I	23	140	34	83	61	99
	117	193	63	51	56	30
UBE2J1	73	293	65	218	113	172
	238	272	134	81	154	58
UBE2J2	22	72	15	42	36	54
	78	75	33	22	40	16
UBE2K	168	891	162	674	429	569
	608	774	289	240	428	123
UBE2L3	114	540	64	316	245	389
	462	593	261	158	239	74
UBE2L6	12	34	7	24	17	25
	36	38	24	25	29	7
UBE2M	21	97	20	90	53	75
	99	129	52	30	50	16
UBE2N	103	504	85	358	234	365
	423	765	257	134	311	87
UBE2NL	6	0	0	0	0	0
	0	0	2	0	1	0
UBE2O	65	257	46	155	82	139
	176	183	85	54	113	36
UBE2Q1	66	198	46	173	125	178
	232	240	102	97	140	40
UBE2Q2	72	348	72	340	150	243
	284	197	188	71	125	54
UBE2QL1	79	276	55	160	160	233
	217	253	125	104	180	42
UBE2R2	152	1106	199	718	438	578
	922	772	288	198	404	99
UBE2S	2	19	6	12	3	27
	13	13	8	4	11	1
UBE2T	30	79	19	58	55	62
	63	129	43	28	61	16
UBE2U	11	0	2	0	4	1
	0	0	8	0	3	0
UBE2V1	28	434	14	231	155	209
	421	300	107	102	148	71
UBE2V2	73	453	76	249	189	259
	283	455	161	127	221	74
UBE2W	146	579	193	493	314	452
	443	634	344	179	269	108
UBE2Z	51	167	37	141	81	108
	149	198	83	48	104	33
UBE3A	567	3087	650	2261	1532	2120
	2268	3478	1294	915	1786	591
UBE3B	178	642	137	406	347	403
	485	550	233	198	313	139

UBE3C	412	2042	297	1435	986	1376
	1387	1747	839	505	917	314
UBE3D	21	32	12	41	22	43
	37	64	42	11	40	6
UBE4A	221	804	197	718	415	625
	875	1157	502	338	667	241
UBE4B	184	795	156	574	374	585
	638	769	329	223	471	175
UBFD1	218	967	193	940	581	853
	997	1198	563	313	612	212
UBIAD1	39	75	14	49	35	42
	77	72	46	23	58	16
UBL3	117	507	129	406	238	384
	470	534	232	151	275	106
UBL4A	52	210	34	186	106	157
	143	234	106	72	141	40
UBL4B	4	0	2	0	0	0
	0	1	2	0	0	0
UBL5	85	285	33	260	133	148
	389	586	218	73	230	61
UBL7	44	112	24	95	35	90
	95	158	96	34	83	20
UBLCP1	27	181	38	135	80	125
	179	192	77	53	105	21
UBN1	88	351	101	258	186	251
	290	344	146	124	165	100
UBN2	322	1319	350	930	618	803
	875	1314	483	358	721	211
UBOX5	47	83	20	37	34	51
	71	51	42	21	44	16
UBP1	83	366	70	293	158	210
	291	258	158	95	176	69
UBQLN1	243	1373	265	1150	590	1110
	1104	1316	663	375	888	243
UBQLN2	51	403	69	301	149	215
	312	262	81	69	152	37
UBQLN3	13	0	6	1	1	0
	1	0	5	1	4	0
UBQLN4	74	323	60	169	128	204
	218	267	104	77	114	51
UBQLNL	14	2	8	1	3	1
	1	0	8	4	4	1
UBR1	320	1141	245	979	628	985
	828	1435	702	388	732	202
UBR2	447	2310	467	1709	1075	1521
	1823	2405	1015	657	1304	457
UBR3	1224	7318	1289	6741	3412	5208
	5829	6573	3064	1825	3904	1184
UBR4	943	3460	815	2953	2045	2621
	2739	3729	1642	1154	2108	708
UBR5	624	2496	634	2153	1438	2156
	1817	2798	1171	791	1416	462
UBR7	39	192	63	153	98	146
	177	235	137	49	132	42
UBTD1	10	23	7	20	15	33
	15	17	5	6	5	7
UBTD2	46	187	43	154	111	142
	125	166	78	37	84	20

UBTF	76	526	116	323	256	385
	344	386	153	148	225	80
UBTFL1	11	0	6	0	0	0
	0	0	7	0	2	0
UBXN1	80	460	73	297	161	442
	492	514	235	130	244	65
UBXN10	13	1	7	1	0	0
	0	2	7	0	2	0
UBXN11	20	22	12	16	9	11
	6	20	19	1	10	3
UBXN2A	78	230	66	210	121	189
	164	262	134	62	146	45
UBXN2B	73	277	59	175	124	175
	162	183	115	61	156	48
UBXN4	214	1270	220	898	565	838
	888	1234	491	341	683	226
UBXN6	64	416	62	218	145	387
	335	391	174	126	202	80
UBXN7	87	348	114	239	146	202
	287	391	163	114	206	67
UBXN8	15	38	11	28	9	34
	27	54	14	8	28	1
UCHL1	22	100	12	29	21	29
	32	18	21	10	13	9
UCHL3	43	287	49	165	101	202
	340	310	157	56	143	29
UCHL5	128	1007	189	819	406	609
	651	817	319	225	499	130
UCK1	27	93	29	90	36	88
	98	85	61	29	58	17
UCK2	44	92	36	90	33	87
	54	73	64	26	39	28
UCKL1	78	205	30	85	93	102
	262	377	140	136	162	66
UCMA	6	0	0	0	0	0
	0	0	2	0	0	0
UCN	1	3	0	0	0	0
	1	3	0	0	1	0
UCN2	4	0	3	0	2	1
	2	2	0	2	0	1
UCN3	2	0	1	0	0	0
	0	0	0	0	2	0
UCP1	6	0	5	0	2	0
	0	0	6	0	1	0
UCP2	11	48	12	17	21	21
	41	56	16	13	24	4
UCP3	390	1475	380	884	603	1459
	2248	1540	1170	1093	1164	314
UEVLD	54	215	49	193	108	212
	195	222	106	71	124	52
UFC1	34	147	29	119	75	153
	153	165	95	51	106	24
UFD1L	43	135	37	189	62	218
	198	150	118	58	99	32
UFL1	198	889	231	619	425	725
	672	930	448	321	647	184
UFM1	115	362	82	277	232	306
	314	504	195	115	228	74

UFSP1	1	4	1	5	3	6
	4	8	1	2	1	4
UFSP2	99	508	110	513	254	321
	399	532	319	145	353	99
UGCG	29	58	28	41	25	25
	32	49	31	23	33	10
UGDH	22	58	29	66	37	48
	44	77	38	22	44	14
UGGT1	329	1081	256	741	601	953
	640	1062	458	342	588	220
UGGT2	86	234	101	234	107	215
	211	330	171	95	188	54
UGP2	536	4064	702	2631	1292	3066
	3692	4945	1993	1066	2373	556
UGT1A1	1	0	0	0	0	0
	0	0	0	0	0	0
UGT1A10	1	0	2	0	2	0
	0	0	1	0	1	0
UGT1A3	2	0	0	0	0	0
	0	0	1	0	0	0
UGT1A4	2	0	0	0	0	0
	0	0	0	0	0	0
UGT1A5	3	0	0	0	0	0
	0	0	0	0	0	0
UGT1A6	0	0	2	0	1	0
	0	0	2	0	0	0
UGT1A7	3	0	2	0	1	0
	0	0	1	0	0	0
UGT1A8	1	0	3	0	0	0
	0	0	3	0	0	0
UGT1A9	2	0	0	0	0	0
	0	0	2	0	1	0
UGT2A1	24	0	17	0	5	0
	0	0	18	0	4	0
UGT2A2	1	0	0	0	0	0
	0	0	1	0	0	1
UGT2A3	24	0	15	0	5	0
	0	0	9	0	7	0
UGT2B10	29	0	7	0	7	0
	0	0	12	0	6	0
UGT2B11	9	0	7	0	4	0
	0	0	5	0	4	0
UGT2B15	8	0	2	0	7	0
	0	1	6	0	3	0
UGT2B17	5	0	4	0	2	0
	0	0	5	0	3	0
UGT2B28	12	0	6	0	5	0
	0	0	3	0	1	0
UGT2B4	13	0	7	0	6	0
	0	1	6	0	6	0
UGT2B7	7	0	5	0	3	0
	0	0	7	0	11	0
UGT3A1	124	60	80	28	46	78
	68	147	91	27	63	19
UGT3A2	8	0	7	0	4	0
	0	0	6	0	4	0
UGT8	25	0	12	2	5	0
	1	0	17	1	11	1

UHMK1	297	1576	328	1548	866	1369
	1354	1793	873	406	975	232
UHRF1	14	4	10	0	7	2
	8	2	9	3	9	0
UHRF1BP1	170	702	179	500	193	472
	551	506	317	191	318	117
UHRF1BP1L	135	474	145	538	191	447
	402	402	328	153	338	98
UHRF2	82	291	87	228	191	260
	334	478	201	149	217	72
UIMC1	77	269	80	175	117	196
	254	319	115	76	126	46
ULBP1	24	2	18	1	6	1
	1	4	17	0	8	1
ULBP2	6	0	4	2	2	0
	0	0	5	0	1	1
ULBP3	5	0	1	0	2	1
	1	1	2	0	4	0
ULK1	47	82	29	87	43	70
	102	129	53	35	82	41
ULK2	130	371	91	213	185	216
	303	443	158	156	283	83
ULK3	62	191	40	149	74	156
	140	202	92	64	103	38
ULK4	72	31	31	31	12	17
	24	49	40	17	31	12
UMOD	8	1	7	0	0	0
	0	0	4	0	2	0
UMODL1	16	1	15	0	10	0
	0	0	12	0	2	0
UMPS	40	118	62	85	65	115
	97	140	59	47	105	34
UNC119	13	25	6	25	10	14
	20	21	14	12	10	1
UNC119B	34	102	28	98	45	83
	96	114	55	17	57	18
UNC13A	72	1	30	0	5	0
	0	0	34	0	27	0
UNC13B	400	1545	634	1593	814	2213
	1149	1453	1248	382	894	226
UNC13C	462	3508	988	3220	1136	2089
	1187	2705	1477	160	2050	280
UNC13D	12	11	19	13	9	9
	10	36	18	9	23	5
UNC45A	36	77	26	66	51	61
	65	80	50	25	58	23
UNC45B	601	2942	702	2133	1500	2812
	2518	3072	1405	834	1687	451
UNC50	48	177	38	174	62	109
	203	259	106	55	128	35
UNC5A	11	0	3	0	1	0
	0	1	4	0	2	0
UNC5B	33	39	18	16	12	18
	32	14	18	5	15	1
UNC5C	69	17	45	18	24	14
	16	2	39	5	27	2
UNC5CL	22	7	14	4	1	4
	2	9	15	1	8	1

UNC5D	33	0	37	2	6	0
	0	0	14	0	19	0
UNC79	81	59	69	47	42	32
	34	35	66	15	69	10
UNC80	116	0	75	1	35	1
	0	0	68	0	38	0
UNC93A	15	0	7	0	1	0
	0	0	7	0	3	0
UNC93B1	8	14	3	7	11	13
	7	20	15	6	11	8
UNCX	0	0	1	0	0	0
	0	0	0	0	0	0
UNG	60	215	36	150	104	142
	218	241	87	46	107	25
UNK	36	99	30	62	26	37
	58	76	39	27	42	21
UNKL	41	97	41	74	44	58
	89	165	81	38	64	37
UPB1	11	8	13	8	3	2
	7	10	6	1	9	0
UPF1	93	267	83	190	124	158
	152	289	148	86	158	66
UPF2	131	738	133	422	361	479
	435	623	291	194	373	115
UPF3A	113	361	108	231	161	325
	279	464	163	140	215	67
UPF3B	45	114	27	70	54	68
	87	104	31	34	41	18
UPK1A	1	0	1	0	0	0
	0	0	4	0	1	0
UPK1B	14	0	8	1	2	1
	0	0	9	0	7	0
UPK2	4	0	1	1	0	0
	1	0	3	0	1	0
UPK3A	8	12	12	11	9	5
	24	25	25	16	22	6
UPK3B	11	6	9	1	2	1
	0	3	4	2	0	3
UPK3BL	0	2	0	2	9	5
	11	15	10	7	8	2
UPP1	18	21	6	16	7	9
	14	20	18	5	12	3
UPP2	46	4	21	0	13	2
	0	2	27	0	23	0
UPRT	45	153	25	141	95	120
	175	176	81	50	107	20
UQCC	137	724	124	532	336	501
	666	746	307	171	482	87
UQCR10	150	682	122	530	280	477
	859	1548	400	203	489	101
UQCR11	131	572	63	525	241	348
	988	1167	398	199	437	119
UQCRB	1647	10072	1401	7840	3437	6821
	14621	18742	5806	2805	6269	1209
UQCRC1	389	2088	291	1772	864	1326
	2435	2510	1110	705	1455	357
UQCRC2	593	3255	401	2194	1233	1750
	3603	4284	1575	886	2110	460

UQCRFS1	269	1731	180	1385	498	935
	2009	2332	797	419	1008	222
UQCRH	380	1867	178	841	700	844
	2311	3332	749	610	1063	241
UQCRHL	3	7	4	4	3	2
	2	5	0	2	3	0
UQCRQ	349	1809	193	1090	582	886
	2504	2706	843	354	922	190
URB1	118	274	133	182	145	206
	205	299	181	85	168	54
URB2	25	123	32	78	57	62
	98	106	59	33	73	18
URGCP	44	207	48	205	81	139
	155	237	92	54	127	44
URGCP-MRPS24		33	64	26	15	27
	48	25	49	35	14	21
	6					
URI1	200	1014	196	627	413	773
	716	1004	425	315	490	180
URM1	27	51	25	37	24	35
	58	84	31	19	47	11
UROC1	9	0	14	1	8	0
	0	1	8	0	5	0
UROD	84	346	62	246	185	240
	393	522	194	92	213	46
UROS	74	484	78	316	147	396
	390	359	214	83	221	43
USB1	21	12	11	8	11	12
	16	23	14	9	7	6
USE1	14	65	8	38	22	42
	59	49	24	23	27	7
USF1	41	193	44	134	76	147
	153	225	134	66	151	40
USF2	75	492	48	228	193	328
	533	353	154	127	185	82
USH1C	28	0	13	0	5	0
	0	0	6	0	7	0
USH1G	14	0	7	0	1	0
	0	0	6	0	1	0
USH2A	127	3	100	1	47	5
	0	4	75	2	52	0
USHBP1	9	36	6	16	11	3
	30	16	17	12	17	3
USMG5	318	1553	161	982	542	876
	2177	2515	754	335	858	208
USO1	1131	5642	1404	4438	2795	4708
	4010	6988	2548	1974	3043	1114
USP1	82	432	112	286	199	280
	305	400	176	101	237	67
USP10	81	520	101	294	176	269
	385	493	177	156	229	107
USP11	55	208	42	135	84	143
	118	191	73	59	102	29
USP12	72	389	87	239	160	314
	340	354	167	95	188	71
USP13	1476	7842	1485	5561	3545	5385
	6849	9887	2630	2459	4509	1718

USP14	122	889	135	708	353	537
	724	885	297	161	370	115
USP15	479	2907	507	1873	1303	2051
	2140	2683	1216	863	1556	407
USP16	127	807	124	571	306	673
	534	844	365	281	455	148
USP17L10	0	0	1	0	1	0
	0	0	0	0	0	0
USP17L11	1	0	4	0	1	0
	0	0	2	0	1	0
USP17L12	0	0	0	0	1	0
	0	0	0	0	2	0
USP17L13	0	0	5	0	0	0
	0	0	5	0	1	0
USP17L15	187	0	162	1	38	1
	0	0	139	0	52	0
USP17L17	3	0	4	0	2	0
	0	0	4	0	0	0
USP17L18	0	0	0	0	0	0
	0	0	1	0	1	0
USP17L19	0	0	0	0	0	0
	0	0	1	0	0	0
USP17L1P	7	0	3	0	0	0
	0	0	2	0	0	0
USP17L2	2	0	2	0	1	0
	0	0	0	0	0	0
USP17L20	1	0	0	0	0	0
	0	0	0	0	1	0
USP17L22	11	0	0	0	2	0
	0	0	3	0	2	0
USP17L24	1	0	1	0	0	0
	0	0	0	0	0	0
USP17L25	0	0	2	0	0	0
	0	0	1	0	1	0
USP17L26	0	0	0	0	1	0
	0	0	0	0	0	0
USP17L28	0	0	1	0	1	0
	0	0	0	0	0	0
USP17L29	0	0	0	0	0	0
	0	0	1	0	1	0
USP17L3	3	0	0	0	0	0
	0	0	0	1	0	0
USP17L30	0	0	0	0	0	0
	0	0	0	0	1	0
USP17L4	5	0	1	0	0	0
	0	0	0	0	0	0
USP17L5	0	0	0	0	1	0
	0	0	0	0	0	0
USP17L7	9	0	3	0	2	0
	0	0	5	0	2	0
USP17L8	1	0	1	0	0	1
	0	0	1	0	0	0
USP18	39	25	25	23	13	17
	20	34	35	13	22	11
USP19	124	542	93	428	278	399
	489	614	307	155	345	110
USP2	75	579	72	316	195	242
	504	346	139	160	221	101

USP20	22	43	23	35	23	47
	29	55	36	14	44	17
USP21	27	73	31	51	34	68
	66	92	56	17	49	17
USP22	348	1500	335	1242	724	997
	1191	1692	768	446	964	324
USP24	1077	5011	1129	4034	2858	4419
	3478	5735	2368	1607	3166	855
USP25	489	2373	442	1501	1498	1810
	2254	3419	1433	1105	1936	517
USP26	23	0	4	0	8	0
	0	0	2	0	2	0
USP27X	24	14	9	15	19	19
	17	14	8	4	7	3
USP28	283	1234	288	800	727	932
	892	1426	580	370	865	237
USP29	34	0	12	0	0	0
	0	0	6	0	4	0
USP3	54	127	46	142	77	113
	154	243	102	39	121	32
USP30	73	177	45	128	72	152
	170	154	86	59	114	34
USP31	107	380	108	396	147	371
	352	381	292	121	288	79
USP32	241	955	213	698	448	679
	709	836	371	254	497	193
USP33	140	515	136	404	299	373
	383	640	278	193	341	131
USP34	699	3285	884	2734	1621	2544
	2213	2992	1575	1090	1747	585
USP35	20	27	19	23	15	26
	23	30	23	10	22	4
USP36	62	200	80	106	91	144
	167	203	113	86	118	35
USP37	137	321	138	315	230	285
	276	440	206	132	254	77
USP38	241	815	114	701	599	637
	739	936	399	310	541	158
USP39	80	317	62	272	136	232
	268	293	118	75	244	53
USP4	85	353	76	224	126	153
	229	207	116	94	134	61
USP40	172	678	199	419	330	445
	489	836	343	206	364	131
USP41	11	0	5	0	1	0
	2	0	9	0	3	0
USP42	51	80	39	48	51	71
	78	80	47	37	67	17
USP43	16	0	13	0	6	0
	0	0	8	0	6	0
USP44	20	10	10	5	6	3
	3	3	21	1	14	5
USP45	84	248	79	196	107	234
	193	311	162	111	161	67
USP46	102	442	149	383	164	388
	319	407	227	122	244	70
USP47	478	2714	520	1924	1292	2044
	1568	2555	1076	859	1557	515

USP48	147	720	186	524	340	473
	509	638	292	215	350	105
USP49	12	26	8	12	8	17
	20	21	8	5	15	0
USP5	92	323	121	281	146	217
	213	304	144	103	216	78
USP50	11	1	9	1	2	0
	5	5	9	0	9	2
USP51	20	20	13	15	9	4
	8	15	8	1	12	3
USP53	413	2102	510	1695	1140	1314
	2155	2344	1323	729	1634	526
USP54	576	1320	695	1050	842	1252
	1118	3728	821	910	1263	573
USP6	423	684	443	821	631	944
	848	1642	947	665	1124	511
USP6NL	29	28	22	28	22	19
	23	33	31	8	28	11
USP7	305	1725	353	1235	756	1213
	1355	1598	630	582	985	344
USP8	139	682	150	492	333	518
	558	743	285	225	341	143
USP9X	1122	5302	952	4182	3233	3754
	3518	4544	1917	1833	2576	903
USP9Y	1	995	220	791	1	699
	775	1033	538	6	620	195
USPL1	54	224	62	161	96	157
	193	242	104	63	171	47
UST	40	36	19	49	24	37
	17	55	27	14	35	8
UTP11L	302	1670	321	1228	782	1332
	2195	2209	698	517	1331	307
UTP14A	37	110	14	44	48	54
	47	107	44	51	48	18
UTP14C	70	343	68	211	156	209
	218	282	120	100	184	75
UTP15	80	251	88	200	129	216
	189	256	149	81	200	45
UTP18	42	139	57	125	74	122
	110	197	106	44	86	25
UTP20	134	527	155	280	255	308
	366	556	302	160	326	115
UTP23	62	181	38	169	105	123
	154	287	120	75	157	44
UTP3	21	89	17	69	60	62
	63	87	30	31	57	15
UTP6	77	263	79	194	168	242
	209	422	203	118	217	59
UTRN	552	2993	674	2087	1339	1680
	2180	2802	1341	966	1486	603
UTS2	2	0	2	0	1	0
	0	0	2	0	1	0
UTS2D	42	5	23	7	16	1
	1	4	19	2	14	0
UTY	0	837	270	771	2	775
	759	1275	677	3	685	227
UVRAG	90	255	73	182	148	177
	166	322	137	79	198	51

UVSSA	30	105	29	76	76	69
	68	103	52	27	66	27
UXS1	52	217	51	157	82	98
	180	182	60	36	116	35
UXT	41	212	32	120	64	113
	184	317	112	61	96	31
VAC14	28	90	34	48	48	40
	63	55	51	23	48	15
VAMP1	26	64	18	36	31	26
	43	37	21	19	15	5
VAMP2	23	146	21	82	53	58
	95	96	27	30	39	11
VAMP3	133	544	100	390	236	356
	378	543	234	138	315	87
VAMP4	60	217	76	225	132	175
	204	265	150	71	124	45
VAMP5	10	33	6	21	13	16
	34	26	12	2	25	3
VAMP7 (NC_000023 155110942..155173433)				33	98	21
	84	54	109	98	155	74
	48	60	16			
VAMP7 (NC_000024 59213948..59276439)				31	132	25
	98	65	98	115	119	51
	24	98	39			
VAMP8	10	23	11	22	16	28
	40	56	14	10	4	5
VANGL1	52	51	22	29	25	24
	36	50	30	9	32	7
VANGL2	22	27	14	9	15	7
	25	14	12	7	10	1
VAPA	351	1854	347	1501	950	1404
	1380	2002	856	446	1068	284
VAPB	224	1585	279	1182	660	922
	1023	1148	398	342	573	243
VARS	42	155	35	118	57	100
	132	138	69	47	77	35
VARS2	34	111	33	101	58	95
	142	220	92	58	118	24
VASH1	56	83	36	61	28	55
	65	118	53	29	57	18
VASH2	36	16	7	16	20	20
	6	14	13	5	19	4
VASN	6	10	11	18	10	6
	4	17	10	3	2	3
VASP	22	52	12	44	27	40
	28	50	21	6	19	7
VAT1	33	76	28	101	56	83
	70	103	43	13	54	17
VAT1L	23	4	10	2	4	0
	1	2	8	0	1	1
VAV1	16	9	20	10	11	11
	12	17	13	3	16	4
VAV2	20	23	19	14	12	13
	11	27	17	6	13	5
VAV3	63	33	41	22	27	27
	20	45	52	12	32	6
VAX1	37	0	11	0	3	0
	0	0	6	0	7	2

VAX2	3	0	2	0	3	0
	0	0	3	0	2	1
VBP1	191	937	140	810	399	774
	950	1281	561	270	641	123
VCAM1	32	49	49	50	23	17
	39	61	28	13	25	6
VCAN	232	711	396	763	387	876
	398	1022	337	178	424	218
VCL	438	2806	683	2099	1498	2157
	1778	2356	1030	623	1274	481
VCP	630	3037	591	2450	1518	2262
	2251	3830	1597	885	2040	535
VCPIP1	180	820	177	667	462	490
	663	889	356	258	514	174
VCX	1	0	2	0	0	0
	1	0	1	0	0	0
VCX2	3	0	0	0	1	0
	1	0	0	0	0	0
VCX3A	1	0	0	0	0	1
	0	0	1	0	1	0
VCX3B	8	0	6	0	0	0
	0	0	2	0	1	0
VCY	0	0	0	0	0	0
	0	0	1	0	0	0
VCY1B	0	0	0	0	0	0
	0	0	1	0	0	0
VDAC1	1250	5933	1034	4625	2814	4661
	5168	7546	3199	2010	3737	1142
VDAC2	329	2730	336	1742	847	1529
	2541	2913	1079	591	1308	336
VDAC3	747	3631	474	2694	1713	2309
	4155	6103	2211	1107	2739	597
VDR	29	6	17	5	9	7
	1	3	10	0	13	2
VEGFA	282	1178	114	535	566	787
	1378	1366	465	440	643	257
VEGFB	58	326	39	200	112	200
	528	296	123	84	131	46
VEGFC	9	43	18	26	14	28
	20	39	25	7	21	2
VENTX	5	6	3	6	3	3
	2	3	2	0	2	0
VEPH1	50	2	31	5	10	1
	5	5	25	1	8	0
VEZF1	242	1007	187	754	583	748
	1013	1180	481	355	629	190
VEZT	311	1598	374	1178	705	1575
	1170	1646	745	582	1029	261
VGF	2	0	2	0	0	0
	0	0	0	0	0	0
VGLL1	5	0	1	0	0	0
	0	0	4	0	0	0
VGLL2	72	339	34	304	191	326
	479	356	159	110	181	55
VGLL3	67	234	79	200	111	171
	155	277	95	61	149	52
VGLL4	85	230	60	178	85	175
	148	278	169	41	130	26

VHL	45	158	49	134	89	143
	120	219	92	63	129	41
VHLL	5	0	5	0	3	2
	0	1	0	0	4	1
VIL1	22	1	13	0	9	0
	0	0	9	0	11	0
VILL	13	15	8	15	9	7
	7	16	15	3	13	0
VIM	202	1178	297	1170	515	789
	715	1493	582	300	565	472
VIMP	60	268	36	216	111	219
	285	335	145	66	130	29
VIP	9	7	10	7	10	1
	7	4	6	0	7	2
VIPAS39	64	126	44	80	68	90
	100	170	92	45	100	27
VIPR1	23	5	20	10	10	14
	12	19	17	2	12	5
VIPR2	36	40	15	35	16	34
	60	43	31	19	36	12
VIT	66	125	61	158	87	121
	88	192	86	36	104	44
VKORC1	15	40	7	24	22	31
	51	45	22	6	17	12
VKORC1L1	13	54	17	48	34	52
	42	53	29	11	43	9
VLDLR	152	780	170	582	502	664
	744	775	350	161	416	107
VMA21	62	265	70	274	126	212
	254	350	174	87	175	73
VMAC	8	20	1	6	8	6
	14	15	13	6	8	1
VMO1	3	0	3	19	2	0
	16	0	1	0	1	0
VMP1	70	302	70	220	112	160
	234	294	121	68	153	42
VN1R1	21	22	12	19	27	15
	29	38	31	22	54	9
VN1R2	13	2	5	0	1	0
	0	0	4	0	2	0
VN1R4	11	0	1	0	0	0
	0	0	0	0	1	0
VN1R5	3	0	2	0	4	0
	0	0	3	0	1	0
VNN1	30	3	12	4	16	4
	2	10	19	0	11	0
VNN2	12	13	7	1	3	7
	6	8	13	2	4	3
VOPP1	87	434	68	417	198	369
	367	508	215	98	238	66
VPRBP	146	343	101	224	224	210
	276	418	158	112	158	57
VPREB1	0	1	0	0	0	0
	0	0	0	0	0	0
VPREB3	2	0	0	0	0	0
	0	1	0	0	1	0
VPS11	57	235	41	134	86	105
	131	267	124	69	106	49

VPS13A	887	3441	868	2679	1524	2511
	2308	4130	1827	1023	2470	860
VPS13B	326	1220	401	874	579	832
	744	1288	587	340	740	274
VPS13C	932	3934	1074	3099	1790	2898
	2935	5520	2189	1491	3065	833
VPS13D	674	3076	688	2376	1386	2241
	2227	3305	1481	931	1856	677
VPS16	25	102	40	71	37	56
	74	109	56	22	58	17
VPS18	30	82	29	51	69	59
	69	79	51	30	49	19
VPS25	32	100	18	66	54	59
	93	183	60	29	71	16
VPS26A	72	437	82	392	197	321
	345	431	163	93	227	72
VPS26B	46	139	35	132	70	131
	113	154	86	36	89	19
VPS28	39	98	11	49	72	45
	91	119	67	42	54	19
VPS29	81	645	101	449	207	397
	579	676	242	146	304	110
VPS33A	56	179	27	128	71	123
	108	179	82	57	107	50
VPS33B	33	39	23	27	28	31
	35	45	36	18	27	10
VPS35	147	634	187	481	338	450
	468	641	322	226	374	125
VPS36	157	747	162	521	410	525
	649	983	420	272	465	140
VPS37A	140	584	120	472	285	426
	467	707	294	198	353	120
VPS37B	19	39	3	22	18	17
	21	25	14	12	17	3
VPS37C	19	73	12	29	20	52
	65	41	24	13	34	10
VPS37D	1	0	1	2	0	0
	2	2	4	0	1	0
VPS39	114	526	122	375	222	405
	410	545	262	140	298	111
VPS41	91	234	90	193	102	175
	175	313	165	86	160	67
VPS45	99	405	118	266	213	336
	265	395	152	108	217	75
VPS4A	116	628	92	412	278	488
	568	628	292	185	338	108
VPS4B	46	199	38	161	85	120
	132	249	108	64	118	52
VPS51	57	225	56	163	69	188
	108	273	157	69	114	49
VPS52	93	348	81	208	152	221
	269	394	167	105	207	70
VPS53	168	328	137	208	199	219
	215	266	164	117	175	69
VPS54	126	484	123	360	217	354
	362	653	273	164	304	98
VPS72	68	264	70	206	151	274
	303	490	164	94	175	38

VPS8	296	1068	316	835	570	1088
	1017	2254	948	432	870	224
VPS9D1	14	38	12	13	14	10
	25	46	19	11	15	2
VRK1	32	66	27	42	33	32
	45	75	42	23	59	9
VRK2	79	284	64	169	134	206
	220	253	107	80	146	53
VRK3	26	59	20	40	28	35
	32	52	34	16	36	7
VRTN	8	0	6	0	5	0
	0	0	3	0	1	0
VSIG1	30	1	9	0	4	1
	0	3	6	0	3	0
VSIG10	111	338	59	294	186	268
	424	622	234	147	302	82
VSIG10L	17	2	10	0	1	0
	4	0	5	0	8	0
VSIG2	10	8	7	5	6	5
	7	0	6	2	2	1
VSIG4	34	17	9	27	18	41
	14	72	27	7	20	12
VSIG8	8	1	1	0	0	0
	0	0	3	0	1	0
VSNL1	15	26	18	32	17	47
	33	30	17	11	20	8
VSTM1	7	0	1	0	0	0
	0	0	3	0	0	0
VSTM2A	6	0	2	5	1	0
	0	0	5	0	1	0
VSTM2B	1	1	1	0	0	0
	0	0	0	0	0	0
VSTM2L	8	1	4	0	1	0
	0	0	0	0	1	0
VSTM4	77	172	57	147	57	124
	79	116	85	32	71	17
VSTM5	3	0	3	1	0	0
	0	0	1	0	0	0
VSX1	21	1	10	1	5	2
	1	13	10	2	10	1
VSX2	4	0	3	0	1	0
	0	0	2	0	2	0
VTA1	165	853	141	557	334	560
	581	830	338	203	465	107
VTCN1	37	0	16	2	5	1
	0	2	13	0	12	0
VTI1A	69	339	71	262	147	254
	278	348	170	96	204	50
VTI1B	89	708	75	350	230	422
	598	540	185	152	264	79
VTN	10	2	5	0	3	1
	2	0	0	0	2	0
VWA1	10	25	3	20	18	24
	21	17	16	6	11	6
VWA2	11	1	4	0	4	0
	0	4	11	0	8	1
VWA3A	37	1	18	1	2	4
	0	1	17	1	11	1

VWA3B	43	10	26	4	15	2
	7	14	24	2	18	1
VWA5A	41	36	34	55	39	65
	24	71	56	20	39	19
VWA5B1	23	1	22	0	12	1
	0	3	16	0	10	0
VWA5B2	3	0	7	0	2	0
	0	0	7	0	4	0
VWA7	27	34	13	15	22	31
	15	44	28	15	34	13
VWA8	404	2109	489	1586	1051	1240
	1835	2182	969	637	1253	348
VWA9	40	170	45	115	66	104
	174	240	111	50	126	30
VWC2	8	0	3	2	0	2
	0	1	4	0	2	0
VWC2L	4	0	2	0	1	0
	1	0	2	0	2	0
VWCE	15	4	8	3	5	2
	3	0	6	0	8	6
VWDE	39	2	29	6	13	0
	1	0	27	3	20	0
VWF	369	1997	229	889	886	878
	1566	1990	1279	609	1337	539
WAC	255	1039	233	662	635	864
	908	1153	455	398	611	243
WAPAL	254	1093	220	731	596	870
	825	1164	418	363	651	188
WARS	101	559	101	319	241	350
	509	555	347	248	435	110
WARS2	32	115	36	65	71	69
	64	112	61	47	62	21
WAS	12	1	8	5	5	4
	3	9	3	1	5	2
WASF1	24	10	8	8	7	7
	3	12	12	1	11	1
WASF2	64	234	58	162	138	125
	178	225	106	83	127	70
WASF3	39	110	42	134	72	112
	81	121	61	44	74	30
WASH1	71	253	54	182	104	220
	168	277	115	42	141	51
WASL	125	749	148	537	357	459
	620	689	251	205	375	151
WBP1	18	56	6	40	23	70
	81	83	30	20	47	18
WBP11	74	383	87	265	180	294
	336	391	164	142	208	92
WBP1L	128	290	43	236	228	211
	501	368	163	109	236	83
WBP2	74	182	28	120	100	120
	196	292	90	68	158	43
WBP2NL	16	9	12	15	11	1
	5	10	11	5	12	3
WBP4	103	601	119	353	292	466
	431	890	251	190	343	111
WBP5	17	63	12	32	26	65
	46	69	27	23	34	11

WBSCR16	58	377	54	224	149	187
	308	463	175	96	236	55
WBSCR17	14	13	22	6	13	10
	11	7	11	2	10	9
WBSCR22	50	303	39	218	102	206
	310	288	145	73	175	47
WBSCR27	3	0	2	0	0	1
	0	2	2	0	1	0
WBSCR28	2	0	0	0	1	0
	0	0	0	0	0	0
WDFY1	121	410	107	367	234	317
	312	452	255	157	278	87
WDFY2	26	38	27	39	24	39
	21	41	42	11	39	10
WDFY3	401	1344	421	1202	703	1039
	860	1494	691	404	762	307
WDFY4	79	41	46	41	36	51
	16	43	62	9	43	1
WDHD1	47	33	42	29	21	26
	33	50	48	13	42	8
WDPCP	45	88	42	82	56	93
	86	140	68	24	84	29
WDR1	118	602	123	371	240	397
	364	584	222	118	296	105
WDR11	107	326	135	262	175	261
	307	535	280	125	326	89
WDR12	65	288	78	191	117	192
	223	334	110	86	160	41
WDR13	33	143	20	120	55	112
	126	212	108	44	95	26
WDR16	17	1	6	0	5	0
	0	2	9	0	7	0
WDR17	56	3	34	8	18	13
	7	25	53	2	26	3
WDR18	16	22	5	19	11	20
	27	36	23	4	30	2
WDR19	56	107	34	71	52	67
	95	147	91	42	89	36
WDR20	84	223	78	170	121	155
	251	350	158	96	175	52
WDR24	7	15	7	14	6	13
	13	22	13	7	7	7
WDR25	29	56	10	48	34	60
	54	86	35	19	57	5
WDR26	426	2456	372	1933	981	1565
	1857	1927	769	552	1132	359
WDR27	55	137	46	84	33	78
	77	106	65	39	60	11
WDR3	97	262	95	204	155	250
	228	391	183	96	189	68
WDR31	16	7	12	1	4	2
	11	7	19	3	3	1
WDR33	209	917	266	635	408	737
	650	1106	529	285	604	176
WDR34	12	8	5	13	9	13
	4	10	6	5	6	0
WDR35	91	157	75	96	107	138
	128	200	110	42	136	28

WDR36	166	542	186	509	352	539
	634	854	376	206	461	111
WDR37	83	290	65	213	119	174
	279	342	182	101	209	61
WDR38	8	0	2	0	1	0
	0	0	7	0	1	0
WDR4	7	24	12	11	10	16
	12	26	13	9	11	5
WDR41	94	294	63	226	141	277
	195	331	146	75	158	52
WDR43	99	548	131	379	272	417
	481	676	257	197	387	110
WDR44	68	300	79	214	162	211
	234	253	145	87	172	61
WDR45	38	129	28	106	46	145
	125	214	101	45	132	28
WDR45L	55	186	61	148	78	171
	152	283	120	86	103	22
WDR46	39	146	37	130	64	136
	125	191	77	41	99	28
WDR47	182	578	128	428	347	363
	499	594	266	198	351	97
WDR48	111	405	93	256	185	265
	339	437	171	124	230	76
WDR49	21	3	17	1	7	10
	1	10	23	2	14	1
WDR5	54	162	42	88	70	105
	135	180	75	42	76	30
WDR52	118	233	133	159	130	178
	100	189	153	57	122	43
WDR53	19	55	12	36	22	35
	52	60	26	17	34	8
WDR54	12	5	6	3	4	2
	2	1	9	0	5	1
WDR55	40	135	15	72	37	59
	64	84	45	20	46	17
WDR59	77	236	53	152	102	113
	188	176	128	62	106	28
WDR5B	30	64	26	47	30	52
	44	70	55	15	48	17
WDR6	75	392	60	235	186	294
	333	423	180	147	195	66
WDR60	83	267	63	160	108	181
	247	250	116	104	165	64
WDR61	39	173	49	157	74	140
	164	244	109	51	122	30
WDR62	33	109	24	68	51	86
	143	40	76	27	105	15
WDR63	45	6	23	1	8	3
	2	4	12	3	7	0
WDR64	39	0	27	0	12	2
	0	0	37	1	22	0
WDR65	39	0	21	1	7	0
	3	0	15	0	9	0
WDR66	51	15	25	15	14	13
	13	8	16	3	13	4
WDR67	51	137	44	63	77	83
	102	151	62	42	97	23

WDR69	19	0	11	0	3	0
	0	0	5	0	4	0
WDR7	153	682	153	537	331	486
	472	672	354	188	377	106
WDR70	79	296	80	216	134	234
	234	312	168	86	204	65
WDR72	44	0	34	1	13	1
	0	0	32	0	10	0
WDR73	31	80	21	47	33	45
	31	112	38	28	35	13
WDR74	39	91	20	86	36	96
	84	146	62	46	85	26
WDR75	76	257	89	215	153	235
	246	358	151	122	180	37
WDR76	27	13	19	18	18	14
	15	38	32	4	13	6
WDR77	54	174	43	123	64	127
	112	225	96	37	100	23
WDR78	51	31	46	28	14	11
	16	34	37	4	22	4
WDR81	23	65	27	53	34	55
	77	133	49	34	61	18
WDR82	197	846	172	650	392	599
	718	755	360	230	469	158
WDR83	7	30	4	17	6	8
	11	24	12	6	9	2
WDR83OS	36	110	28	117	64	86
	145	183	69	37	69	34
WDR85	30	64	21	30	39	53
	64	95	43	22	31	15
WDR86	1	0	1	0	3	0
	0	1	1	0	1	1
WDR87	70	0	43	0	12	0
	0	0	27	0	22	0
WDR88	17	0	7	3	4	0
	2	1	3	0	2	0
WDR89	54	153	57	148	81	147
	140	233	100	65	107	23
WDR90	13	17	10	5	3	6
	13	24	10	7	14	3
WDR91	41	93	49	94	50	93
	71	85	62	26	37	20
WDR92	43	163	31	78	101	133
	118	179	86	55	85	29
WDR93	14	0	9	0	1	1
	0	0	5	0	2	0
WDR96	68	3	35	0	17	1
	1	4	40	1	35	0
WDSUB1	33	93	18	94	38	65
	107	151	63	40	66	14
WDTC1	111	431	61	263	178	243
	334	357	191	106	222	68
WDYHV1	18	60	10	18	25	42
	50	63	45	17	41	13
WEE1	54	132	26	74	50	60
	108	135	62	43	75	30
WEE2	24	0	21	0	6	3
	1	0	14	1	8	0

WFDC1	7	3	2	3	3	0
	1	0	8	2	3	3
WFDC10A	2	0	1	0	0	0
	0	0	2	0	1	0
WFDC10B	9	2	5	0	1	0
	0	1	2	0	7	0
WFDC11	9	1	2	1	3	0
	0	0	3	0	2	0
WFDC12	4	0	5	0	2	1
	0	1	0	0	1	0
WFDC13	5	0	1	0	0	0
	0	2	0	0	1	1
WFDC2	4	1	3	0	0	0
	0	0	0	0	5	0
WFDC3	4	3	7	0	0	1
	0	1	3	1	2	1
WFDC5	6	0	3	0	1	0
	0	0	6	0	1	0
WFDC6	6	0	0	0	0	0
	0	0	0	0	1	0
WFDC8	14	0	7	0	4	1
	0	1	5	0	3	0
WFDC9	5	0	3	0	1	0
	0	0	2	0	3	0
WFIKKN1	0	1	1	0	0	0
	0	2	0	0	0	0
WFIKKN2	11	4	9	2	3	5
	6	10	4	3	10	2
WFS1	26	87	29	53	40	40
	48	74	36	20	46	17
WHAMM	60	285	41	207	135	190
	243	254	97	91	121	43
WHSC1	150	515	160	325	197	330
	341	306	199	127	215	74
WHSC1L1	165	534	192	398	254	430
	426	573	252	179	355	110
WHSC2	11	47	18	27	23	44
	52	56	26	13	31	10
WIBG	15	65	16	38	33	66
	50	85	22	21	27	13
WIF1	13	0	13	0	5	0
	0	0	9	0	4	0
WIPF1	56	152	40	149	71	111
	127	159	66	43	70	29
WIPF2	64	99	29	123	74	68
	90	108	63	53	68	26
WIPF3	78	241	63	149	116	120
	205	339	72	74	106	70
WIPI1	114	570	103	455	263	471
	556	842	400	226	585	156
WIPI2	71	225	47	143	82	134
	217	241	104	62	147	48
WISP1	14	12	15	4	8	6
	8	0	8	0	8	2
WISP2	21	25	7	85	42	78
	12	45	18	5	24	19
WISP3	7	0	5	0	2	0
	0	0	1	0	3	0

WIZ	24	65	11	32	26	51
	60	47	29	16	36	13
WLS	47	113	29	68	55	65
	58	89	55	26	51	24
WNK1	669	3765	560	2290	1840	2059
	2312	3050	1153	1106	1701	727
WNK2	78	422	66	277	120	227
	328	264	93	113	178	66
WNK3	64	10	21	5	19	9
	2	7	27	3	14	5
WNK4	23	1	7	0	4	1
	0	0	5	0	3	0
WNT1	2	0	2	0	0	0
	0	0	2	0	1	0
WNT10A	4	1	4	0	2	0
	0	0	3	0	2	0
WNT10B	4	0	10	2	4	4
	1	2	6	1	3	1
WNT11	15	38	9	48	26	42
	52	57	31	10	32	1
WNT16	9	0	11	0	7	0
	0	0	4	0	8	0
WNT2	24	4	9	2	3	0
	2	5	8	1	6	1
WNT2B	14	10	17	12	7	5
	5	4	9	3	12	1
WNT3	6	1	3	3	1	0
	0	0	7	0	3	0
WNT3A	8	0	4	0	0	0
	0	0	0	0	1	0
WNT4	21	25	14	40	19	31
	13	12	15	4	10	0
WNT5A	68	151	71	174	103	157
	84	205	100	47	110	31
WNT5B	13	11	10	7	17	8
	9	17	17	2	12	2
WNT6	0	0	1	0	0	0
	0	0	1	0	2	0
WNT7A	10	0	3	0	0	0
	0	0	0	0	0	0
WNT7B	11	0	0	0	6	2
	0	1	5	0	1	0
WNT8A	16	0	5	0	0	0
	0	0	7	0	3	0
WNT8B	6	0	6	0	2	1
	0	2	10	0	3	0
WNT9A	4	17	3	14	7	8
	25	63	15	3	13	5
WNT9B	8	0	2	0	0	0
	0	0	0	0	0	0
WRAP53	15	18	10	13	3	12
	10	18	13	5	13	2
WRAP73	24	29	19	26	21	37
	35	39	20	13	47	10
WRB	40	147	37	172	65	167
	238	191	121	47	125	31
WRN	98	272	102	180	123	164
	166	271	156	92	187	54

WRNIP1	63	227	52	194	106	184
	230	317	130	75	129	47
WSB1	253	1182	236	779	542	886
	763	1324	582	359	635	218
WSB2	100	539	106	446	249	370
	681	765	285	120	347	102
WSCD1	43	162	59	58	97	159
	146	234	75	63	109	30
WSCD2	22	1	19	17	6	9
	6	8	6	0	8	2
WT1	15	0	4	0	2	0
	0	0	9	0	4	0
WTAP	96	453	66	385	227	331
	487	600	255	143	280	91
WTH3DI	27	2	21	0	11	0
	0	0	24	0	14	0
WTIP	0	18	5	10	12	2
	17	6	6	5	10	7
WWC1	35	1	22	1	9	0
	3	1	12	0	14	2
WWC2	76	307	91	162	125	212
	256	196	142	72	106	61
WWC3	77	249	60	145	95	173
	180	221	93	82	110	35
WWOX	29	37	22	47	13	30
	35	45	26	2	20	2
WWP1	637	4817	668	3741	1746	2587
	3586	2934	1155	707	1730	458
WWP2	53	147	54	102	63	81
	62	136	88	51	96	31
WWTR1	80	304	81	235	99	165
	255	273	122	67	115	64
XAB2	25	78	19	81	35	48
	63	92	46	28	48	18
XAF1	252	377	94	220	194	177
	334	436	207	239	239	70
XAGE1A	6	0	4	0	2	0
	0	0	1	0	1	0
XAGE1B	5	0	1	0	1	0
	0	0	1	0	1	0
XAGE1C	5	0	3	0	0	0
	0	0	0	0	1	0
XAGE1D	4	0	0	0	0	0
	0	0	1	0	0	0
XAGE1E	2	0	2	0	2	0
	0	0	0	0	0	0
XAGE2	12	0	4	0	0	0
	0	0	0	0	1	0
XAGE3	12	0	5	0	3	1
	0	0	4	0	2	0
XAGE5	11	0	0	0	0	0
	0	0	2	0	0	0
XBP1	36	223	22	153	76	194
	176	238	98	51	118	50
XCL1	7	0	6	2	3	0
	0	1	2	0	5	0
XCL2	2	0	1	0	0	0
	0	0	3	0	1	0

XCR1	8	1	7	0	0	0
	0	0	5	2	3	0
XDH	53	4	34	1	12	2
	0	3	33	0	14	0
XG	18	23	27	43	28	24
	16	33	26	8	22	8
XIAP	244	802	208	631	431	528
	477	943	370	214	505	163
XIRP1	516	1775	303	789	1395	1054
	800	3087	620	721	726	327
XIRP2	9123	91567	9191	40441	30478	67549
	124922	83061	29140	33772	45467	15332
XK	82	216	73	193	131	171
	148	159	103	53	90	28
XKR3	8	4	7	4	4	0
	1	2	5	0	11	2
XKR4	29	5	12	0	3	0
	5	1	12	1	4	2
XKR5	15	0	14	0	4	0
	0	0	11	0	6	1
XKR6	13	4	9	3	3	2
	1	2	6	1	4	1
XKR7	8	0	2	0	1	0
	0	0	2	0	1	0
XKR8	9	19	7	5	8	10
	14	22	17	7	8	1
XKR9	15	13	16	12	12	16
	22	0	16	4	14	0
XKRX	13	5	3	5	1	2
	0	4	3	0	2	0
XKRY	0	0	4	0	0	0
	0	0	3	0	1	0
XKRY2	0	0	4	0	0	0
	0	0	3	0	1	0
XPA	44	254	46	147	112	177
	200	234	106	81	160	40
XPC	70	211	75	157	123	195
	142	244	123	74	150	55
XPNPEP1	67	219	59	140	106	160
	155	272	138	75	123	37
XPNPEP2	39	6	9	3	12	4
	3	11	9	2	14	1
XPNPEP3	86	322	79	171	183	150
	184	240	129	77	174	46
XPO1	281	1651	383	886	829	1026
	1006	1792	689	422	732	301
XPO4	1456	4567	1434	1566	1811	2444
	3092	11686	2370	1095	3138	1259
XPO5	94	165	69	105	90	96
	112	199	134	54	113	41
XPO6	73	201	73	170	97	209
	148	255	147	71	146	57
XPO7	149	678	161	486	279	423
	515	717	287	172	369	143
XPOT	129	527	116	413	258	487
	443	568	254	172	269	131
XPR1	106	332	102	256	148	206
	255	275	168	88	171	74

XRCC1	37	87	29	49	41	83
	73	87	29	35	47	17
XRCC2	23	18	20	6	9	9
	8	6	20	4	10	2
XRCC3	14	10	2	6	5	7
	6	9	11	4	10	1
XRCC4	14	19	18	11	14	14
	12	18	21	5	11	0
XRCC5	260	1389	316	1037	637	931
	1006	1520	708	472	922	298
XRCC6	324	1669	348	1185	764	1203
	1229	1934	752	533	936	307
XRCC6BP1	22	95	6	98	36	95
	94	85	82	32	67	22
XRN1	297	1451	384	1168	734	989
	1153	1663	721	493	990	310
XRN2	118	529	122	312	263	387
	443	673	279	200	313	119
XRRA1	129	117	110	83	156	132
	90	166	177	115	101	26
XXYLT1	20	30	13	14	17	17
	19	21	17	4	11	1
XYLB	33	38	18	16	23	34
	20	28	25	7	22	2
XYLT1	64	50	27	42	37	33
	39	125	33	29	26	16
XYLT2	19	78	24	39	49	71
	36	70	43	13	43	11
YAE1D1	15	117	18	52	46	49
	78	140	25	32	58	13
YAF2	127	500	126	344	259	371
	445	807	275	179	347	102
YAP1	250	1843	417	1216	801	1094
	1178	1220	738	331	722	190
YARS	49	256	50	166	123	214
	192	203	109	62	141	58
YARS2	31	88	20	76	39	63
	61	93	49	17	60	20
YBEY	16	32	12	40	22	41
	37	56	16	11	32	7
YBX1	1784	10884	1623	5116	4588	8648
	12091	11523	3847	3576	6000	1829
YBX2	9	7	3	1	1	6
	6	13	2	3	7	5
YDJC	4	3	3	1	3	2
	2	12	5	0	4	3
YEATS2	107	212	91	132	135	166
	117	157	131	35	97	28
YEATS4	11	90	13	58	35	65
	77	103	69	30	67	16
YES1	76	364	70	267	208	290
	297	400	223	119	193	81
YIF1A	14	66	16	55	27	43
	72	102	47	19	38	14
YIF1B	17	20	12	10	8	16
	20	23	17	6	15	2
YIPF1	33	103	16	50	30	104
	58	85	40	33	42	15

YIPF2	6	33	15	22	12	24
	18	41	24	15	20	4
YIPF3	46	138	32	105	57	118
	100	219	80	58	80	27
YIPF4	114	562	99	487	302	456
	482	834	370	210	423	92
YIPF5	40	171	51	154	91	122
	143	234	144	60	115	29
YIPF6	156	755	158	573	361	662
	509	848	363	203	422	145
YIPF7	83	398	73	321	218	332
	372	591	228	100	308	79
YJEFN3	5	7	4	2	0	1
	5	5	2	2	1	1
YKT6	53	226	43	177	107	210
	228	238	93	56	110	36
YLPM1	147	631	166	389	329	411
	436	581	240	231	344	108
YME1L1	313	1745	301	1182	913	1104
	1200	1921	726	502	1086	307
YOD1	51	263	55	155	129	116
	212	352	121	68	212	47
YPEL1	34	106	71	82	37	81
	53	116	91	28	70	18
YPEL2	205	1096	187	1048	443	688
	748	799	454	238	493	123
YPEL3	66	134	26	88	96	128
	272	344	124	64	174	69
YPEL4	7	7	3	1	2	8
	4	0	8	0	5	1
YPEL5	110	467	116	378	228	344
	505	513	266	103	328	93
YRDC	13	22	11	47	13	30
	50	51	21	13	28	2
YTHDC1	154	757	162	432	319	501
	537	901	359	266	561	173
YTHDC2	174	737	180	596	333	583
	689	1038	421	244	528	158
YTHDF1	81	374	65	277	183	274
	334	415	167	105	192	60
YTHDF2	79	370	71	294	178	249
	324	399	145	115	237	82
YTHDF3	176	1063	189	894	527	848
	806	1061	418	280	562	168
YWHAB	123	864	145	771	376	636
	711	775	350	259	429	135
YWHAE	325	2580	374	1468	1010	1748
	2456	1706	776	576	1057	274
YWHAG	595	4878	525	2766	2019	2841
	3310	3761	1185	1112	1926	548
YWHAH	56	266	65	170	109	194
	197	259	100	78	108	31
YWHAQ	74	312	54	244	141	190
	177	331	118	82	136	85
YWHAZ	149	874	191	645	391	554
	693	916	338	246	431	195
YY1	65	316	66	243	173	266
	290	354	142	104	180	68

YY1AP1	125	614	149	357	273	394
	497	652	219	186	306	104
YY2	1	0	1	1	0	0
	0	0	0	0	0	0
ZACN	8	3	2	0	3	2
	1	5	11	0	7	1
ZADH2	143	790	163	634	389	564
	625	804	368	229	363	143
ZAK	1911	10308	1627	7353	6138	7856
	7275	12515	4084	2899	5955	1987
ZAN	48	0	31	0	3	0
	0	0	30	0	13	0
ZAP70	12	9	3	4	3	5
	4	8	7	0	8	5
ZAR1	2	1	1	0	0	0
	0	0	2	0	1	0
ZAR1L	6	0	3	0	5	2
	0	0	4	0	0	0
ZBBX	31	0	16	0	16	0
	0	2	21	0	12	2
ZBED1 (NC_000023 2404454..2419008)				4	52	5
	107	15	34	39	40	36
	23	22	13			
ZBED1 (NC_000024 2354454..2369008)				14	39	8
	72	25	53	32	36	35
	11	29	15			
ZBED2	11	0	7	0	3	0
	0	0	6	0	2	0
ZBED3	3	8	4	5	2	1
	4	3	4	1	1	0
ZBED4	48	142	43	112	62	117
	136	99	63	43	66	21
ZBED5	111	787	190	530	290	567
	555	820	308	216	433	101
ZBED6	0	2	0	0	0	1
	1	1	0	0	0	0
ZBP1	9	5	11	1	2	1
	0	1	10	1	8	1
ZBTB1	158	676	152	519	363	597
	537	677	300	237	302	142
ZBTB10	98	465	113	352	254	309
	405	607	222	159	318	113
ZBTB11	97	398	95	303	243	356
	307	557	202	146	267	72
ZBTB12	8	5	4	5	2	4
	1	3	6	1	2	0
ZBTB16	172	547	131	323	427	500
	1000	1443	403	406	683	377
ZBTB17	36	76	14	44	39	48
	55	92	41	29	54	21
ZBTB2	49	110	15	90	69	73
	89	166	57	36	64	12
ZBTB20	274	1089	299	907	633	865
	941	1225	624	413	760	297
ZBTB22	16	35	13	33	26	44
	37	35	23	13	25	7
ZBTB24	49	146	39	84	74	107
	76	141	69	46	75	23

ZBTB25	20	73	9	39	42	49
	46	104	50	29	37	18
ZBTB26	14	49	6	35	20	39
	36	53	18	21	21	13
ZBTB3	11	22	10	37	8	24
	22	27	20	11	22	6
ZBTB32	13	0	2	1	0	0
	0	2	2	0	3	0
ZBTB33	81	220	75	182	157	193
	284	259	105	74	135	39
ZBTB34	56	227	54	154	99	163
	135	201	104	47	111	44
ZBTB37	46	121	44	62	60	118
	80	99	59	31	75	19
ZBTB38	357	1422	287	1049	767	1216
	1229	1421	703	480	818	266
ZBTB39	43	111	40	83	74	79
	82	130	84	46	81	24
ZBTB4	119	612	126	369	295	437
	510	600	253	199	294	123
ZBTB40	176	512	152	417	296	431
	295	514	252	151	235	74
ZBTB41	110	370	132	378	230	347
	342	491	194	116	281	54
ZBTB42	11	15	9	5	11	15
	15	20	14	5	14	3
ZBTB43	162	818	166	661	410	578
	697	732	369	223	469	145
ZBTB44	644	3125	590	3209	1837	2478
	2979	3085	1476	989	1633	538
ZBTB45	7	11	6	8	7	6
	16	16	8	4	11	2
ZBTB46	29	65	16	40	29	24
	39	37	24	30	34	7
ZBTB47	92	381	89	314	220	293
	296	480	189	168	245	94
ZBTB48	18	35	11	20	19	19
	20	32	23	12	22	1
ZBTB49	27	54	21	39	26	29
	32	69	33	21	48	14
ZBTB5	39	112	32	112	63	89
	130	144	60	37	113	26
ZBTB6	57	169	55	189	101	171
	166	237	119	59	135	25
ZBTB7A	32	193	22	130	77	163
	163	138	43	61	58	39
ZBTB7B	21	79	36	80	45	134
	55	76	32	26	47	13
ZBTB7C	25	10	11	3	11	12
	10	9	8	2	5	1
ZBTB8A	85	118	39	94	73	106
	112	159	77	50	120	40
ZBTB8B	27	1	10	0	3	0
	0	1	6	0	8	0
ZBTB8OS	20	153	25	80	47	99
	144	202	70	43	74	26
ZBTB9	10	21	10	12	12	10
	11	26	21	6	16	4

ZC2HC1A	28	85	27	47	57	70
	82	154	37	36	61	23
ZC2HC1B	9	7	6	5	5	9
	3	3	7	0	2	0
ZC2HC1C	22	33	14	28	17	28
	34	43	29	9	25	3
ZC3H10	23	44	18	38	30	29
	30	57	22	19	23	7
ZC3H11A	572	2482	677	2458	1483	2544
	2109	2638	1183	908	1456	457
ZC3H12A	12	5	8	3	5	5
	6	14	7	5	11	1
ZC3H12B	34	24	9	18	18	8
	5	11	12	1	9	1
ZC3H12C	180	668	191	580	380	590
	506	638	438	190	397	138
ZC3H12D	24	6	11	6	6	4
	0	6	12	0	3	0
ZC3H13	158	797	180	407	357	544
	536	603	244	291	365	134
ZC3H14	98	394	99	274	218	289
	268	413	165	148	214	81
ZC3H15	99	628	122	388	302	406
	430	592	256	221	340	106
ZC3H18	38	122	38	55	51	62
	86	90	38	31	65	22
ZC3H3	18	35	12	27	18	24
	23	37	12	11	20	9
ZC3H4	41	139	40	84	60	93
	120	112	51	48	70	30
ZC3H6	279	1147	336	1148	668	999
	1066	1306	659	410	813	238
ZC3H7A	86	406	106	218	199	193
	366	427	142	137	202	75
ZC3H7B	70	329	57	144	151	162
	197	281	148	95	154	54
ZC3H8	45	247	50	102	104	164
	236	231	81	74	145	50
ZC3HAV1	81	255	90	148	130	145
	142	236	115	84	141	46
ZC3HAV1L	10	14	6	19	4	11
	11	20	13	7	12	11
ZC3HC1	51	153	25	143	79	142
	154	185	80	40	108	26
ZC4H2	17	20	12	18	14	25
	18	21	13	5	14	2
ZCCHC10	46	137	41	91	86	112
	104	205	93	72	123	24
ZCCHC11	289	1679	374	1255	892	1184
	1145	1419	581	551	819	262
ZCCHC12	13	0	4	0	2	0
	0	0	2	0	3	0
ZCCHC13	5	0	1	0	1	0
	0	0	1	0	0	0
ZCCHC14	94	342	82	252	157	234
	242	295	111	99	145	81
ZCCHC16	20	0	4	0	3	0
	0	0	5	0	0	0

ZCCHC17	46	152	43	129	89	141
	138	177	78	44	99	34
ZCCHC18	16	3	6	3	4	0
	1	2	4	0	1	0
ZCCHC2	102	442	93	232	217	246
	366	432	171	170	234	88
ZCCHC24	120	565	115	514	345	421
	337	369	218	121	206	92
ZCCHC3	16	49	17	28	28	43
	79	85	29	23	46	5
ZCCHC4	60	152	39	104	99	111
	118	263	93	48	107	29
ZCCHC5	6	0	2	0	2	0
	0	0	3	0	0	0
ZCCHC6	131	451	154	294	248	347
	307	484	269	131	281	103
ZCCHC7	65	204	63	160	94	112
	156	226	94	67	131	34
ZCCHC8	83	220	77	193	129	219
	310	417	165	127	238	65
ZCCHC9	42	131	40	108	53	139
	196	142	66	32	95	35
ZCRB1	131	673	127	492	246	698
	797	775	349	225	373	118
ZCWPW1	33	59	32	38	18	40
	37	56	36	8	41	7
ZCWPW2	35	82	24	82	43	60
	63	68	42	15	56	18
ZDBF2	236	912	213	515	574	606
	495	798	414	281	419	140
ZDHHC1	9	2	6	4	6	12
	12	17	13	1	14	6
ZDHHC11	11	32	13	8	15	10
	12	19	17	2	8	16
ZDHHC11B	14	48	18	13	21	16
	16	16	16	6	17	22
ZDHHC12	3	7	5	11	2	12
	14	13	13	4	6	0
ZDHHC13	23	24	18	10	13	22
	17	40	28	11	24	3
ZDHHC14	10	37	10	14	36	18
	11	39	14	17	37	7
ZDHHC15	26	18	12	20	24	13
	8	17	23	3	20	5
ZDHHC16	41	145	41	90	51	127
	136	156	65	46	67	32
ZDHHC17	179	679	180	534	356	451
	548	961	394	246	501	144
ZDHHC18	21	37	17	27	18	28
	30	55	26	13	29	8
ZDHHC19	8	0	6	1	1	2
	0	1	2	1	3	0
ZDHHC2	128	642	211	503	355	479
	811	453	317	129	330	128
ZDHHC20	87	269	51	187	122	176
	166	269	152	75	130	48
ZDHHC21	181	725	216	617	380	581
	484	934	400	220	457	128

ZDHHC22	10	0	12	2	5	0
	0	3	6	0	7	2
ZDHHC23	24	13	17	15	14	15
	11	13	18	3	15	1
ZDHHC24	5	5	3	15	1	5
	2	7	9	0	7	1
ZDHHC3	162	443	96	292	208	367
	396	555	203	147	252	99
ZDHHC4	50	210	41	162	107	210
	222	357	137	68	134	31
ZDHHC5	102	441	98	376	223	307
	375	480	203	138	266	81
ZDHHC6	49	145	55	121	87	150
	111	243	98	57	120	34
ZDHHC7	132	675	121	561	310	565
	681	645	290	192	341	99
ZDHHC8	20	57	23	32	28	37
	32	66	32	16	30	13
ZDHHC9	51	70	26	65	56	90
	75	91	52	34	53	17
ZEB1	371	2046	478	1579	1098	1245
	1409	1589	742	593	974	347
ZEB2	157	873	228	619	411	584
	663	712	329	292	444	168
ZER1	66	269	45	143	127	189
	185	280	136	100	154	61
ZFAND1	41	206	50	144	96	187
	178	176	114	71	124	31
ZFAND2A	11	26	4	27	14	34
	10	39	22	7	22	3
ZFAND2B	19	93	14	58	45	90
	59	89	53	19	50	7
ZFAND3	65	419	59	211	170	256
	280	313	117	102	136	54
ZFAND4	33	99	32	77	56	75
	89	147	61	28	66	13
ZFAND5	514	2239	386	1823	1146	1523
	2294	3941	1199	750	1529	570
ZFAND6	150	919	145	681	400	656
	772	1011	417	231	522	129
ZFAT	48	169	47	79	66	88
	86	126	56	50	88	12
ZFC3H1	133	597	152	426	305	397
	447	621	264	183	328	122
ZFHX2	60	20	30	5	12	13
	1	5	21	1	23	3
ZFHX3	191	682	182	526	351	397
	509	756	254	238	308	164
ZFHX4	274	1160	361	941	565	867
	982	1036	467	283	531	189
ZFP1	25	77	31	48	35	87
	94	107	34	32	60	16
ZFP106	4890	30813	5431	19903	14365	17500
	22833	26891	10625	10002	17849	5704
ZFP112	31	62	33	34	32	41
	31	73	58	22	54	16
ZFP14	56	126	54	89	63	73
	62	101	69	31	63	29

ZFP161	36	150	35	168	102	158
	138	138	97	56	98	13
ZFP2	27	17	26	22	12	19
	13	23	12	6	11	9
ZFP28	27	72	23	47	53	62
	47	76	30	18	46	10
ZFP3	58	239	50	162	91	151
	160	199	85	81	137	49
ZFP30	42	94	29	93	68	71
	75	111	67	27	61	22
ZFP36	17	65	25	46	76	62
	111	160	53	44	50	48
ZFP36L1	64	464	112	364	141	291
	342	395	95	92	154	58
ZFP36L2	44	360	87	236	140	214
	212	306	98	74	118	60
ZFP37	12	32	10	24	15	21
	21	25	27	11	27	5
ZFP41	36	76	24	24	22	39
	53	68	42	17	38	11
ZFP42	14	0	6	0	1	0
	1	0	11	0	3	0
ZFP57	12	0	7	0	4	0
	0	0	7	0	1	0
ZFP62	66	255	103	152	119	210
	152	268	112	90	161	52
ZFP64	37	65	20	43	31	41
	37	56	40	25	36	8
ZFP82	23	21	6	9	10	5
	14	12	14	7	17	8
ZFP90	45	195	57	109	63	97
	125	140	77	64	94	43
ZFP91	386	1799	328	1396	902	1408
	1324	1843	808	548	1099	360
ZFP92	7	0	3	0	1	0
	1	2	1	0	1	1
ZFPL1	14	24	9	22	16	28
	33	50	22	19	31	5
ZFPM1	7	14	2	4	6	7
	8	6	4	2	3	0
ZFPM2	86	412	73	300	182	219
	351	260	114	98	160	50
ZFR	216	969	229	700	490	760
	657	966	457	300	598	210
ZFR2	21	1	3	0	0	1
	0	0	6	0	2	2
ZFX	341	1065	219	867	882	869
	940	1088	466	492	692	199
ZFY	1	377	79	266	0	251
	228	391	165	2	208	51
ZFYVE1	53	174	46	96	94	114
	141	147	81	48	93	43
ZFYVE16	182	867	212	618	360	637
	625	963	453	280	531	167
ZFYVE19	18	70	24	58	38	63
	77	67	40	29	27	16
ZFYVE20	121	416	94	331	272	289
	272	447	173	138	231	70

ZFYVE21	22	78	29	50	31	68
	79	118	58	24	49	20
ZFYVE26	173	499	144	333	220	306
	386	505	296	155	308	100
ZFYVE27	19	44	18	38	25	31
	24	52	32	19	30	9
ZFYVE28	18	30	24	25	23	12
	12	37	16	3	30	2
ZFYVE9	202	946	228	863	564	942
	631	889	450	319	485	160
ZG16	13	0	7	0	0	0
	0	0	3	0	0	0
ZG16B	2	0	0	0	0	0
	0	0	1	0	0	0
ZGLP1	7	4	2	3	3	2
	3	7	5	0	3	1
ZGPAT	17	88	14	44	25	48
	32	103	27	19	39	11
ZHX1	5	83	11	78	33	37
	45	69	31	35	26	10
ZHX1-C8ORF76		4	47	12	65	32
	60	33	85	38	14	35
	7					
ZHX2	75	302	46	195	134	198
	278	210	104	80	154	61
ZHX3	175	695	184	731	404	776
	646	778	421	224	476	130
ZIC1	34	47	33	56	59	37
	24	60	52	19	37	4
ZIC2	10	0	3	0	2	0
	0	0	0	0	2	0
ZIC3	14	0	7	0	7	0
	0	0	7	1	4	3
ZIC4	18	4	18	2	3	4
	3	3	13	0	7	1
ZIC5	19	0	3	0	5	0
	0	0	11	1	7	0
ZIK1	21	13	15	10	7	7
	9	23	19	5	18	7
ZIM2	21	6	10	8	6	5
	4	5	16	1	11	0
ZIM3	21	0	6	0	4	0
	0	1	4	0	5	0
ZKSCAN1	107	468	125	367	249	338
	386	566	211	164	248	118
ZKSCAN2	89	202	42	124	110	133
	166	191	87	61	139	37
ZKSCAN3	30	64	13	40	31	31
	31	55	29	27	33	11
ZKSCAN4	28	31	15	24	24	28
	20	39	21	15	20	7
ZKSCAN5	44	158	40	107	87	114
	84	130	78	43	96	26
ZMAT1	61	111	45	59	56	88
	71	147	59	49	67	24
ZMAT2	89	557	51	212	203	310
	455	653	231	138	264	80

ZMAT3	70	310	114	329	135	250
	163	247	138	72	161	45
ZMAT4	14	0	9	4	5	0
	1	0	9	0	1	0
ZMAT5	30	100	11	52	33	55
	93	128	40	21	52	20
ZMIZ1	66	229	60	167	113	134
	122	189	90	39	96	44
ZMIZ2	27	94	31	55	51	56
	70	78	35	23	32	10
ZMPSTE24	64	318	71	266	162	265
	275	337	161	79	182	55
ZMYM1	31	111	36	82	34	65
	71	103	48	24	58	17
ZMYM2	271	1229	286	1112	656	976
	765	1105	636	299	700	243
ZMYM3	80	132	52	91	99	99
	93	143	72	38	77	26
ZMYM4	191	1067	254	833	499	819
	825	1048	527	300	587	200
ZMYM5	74	187	46	128	94	132
	172	204	101	69	114	50
ZMYM6	60	212	57	163	131	145
	173	236	134	61	146	37
ZMYM6NB	11	15	5	10	9	5
	19	37	10	7	11	2
ZMYND10	7	4	11	3	2	3
	1	1	2	2	4	0
ZMYND11	256	1221	241	1015	613	926
	1035	1256	604	433	650	233
ZMYND12	23	27	10	4	5	10
	15	29	27	10	10	1
ZMYND15	8	8	11	6	1	2
	4	2	3	1	4	0
ZMYND19	15	55	15	22	21	21
	34	59	26	8	22	6
ZMYND8	157	390	130	291	202	292
	371	547	182	133	291	77
ZNF10	44	138	31	69	58	56
	97	113	55	37	85	28
ZNF100	55	99	57	105	117	138
	172	244	124	35	96	43
ZNF101	10	10	8	13	10	6
	7	18	8	4	10	6
ZNF107	47	99	50	84	53	76
	78	65	60	26	50	10
ZNF114	16	3	4	1	1	2
	2	0	8	2	4	1
ZNF117	83	225	58	141	131	171
	65	151	76	56	153	67
ZNF12	63	202	44	165	96	155
	161	212	109	61	147	37
ZNF121	44	128	25	65	78	62
	87	141	68	42	99	34
ZNF124	27	60	21	35	26	30
	47	71	27	21	32	17
ZNF131	49	239	63	205	111	178
	186	232	103	84	147	37

ZNF132	30	40	17	24	28	27
	22	51	24	17	34	14
ZNF133	46	160	39	105	62	124
	117	197	97	42	91	28
ZNF134	24	96	43	70	49	55
	74	130	87	43	90	31
ZNF135	50	33	29	15	25	10
	20	37	35	24	31	12
ZNF136	25	68	24	56	42	38
	72	74	53	25	67	21
ZNF138	71	292	90	213	141	211
	121	310	116	77	142	59
ZNF14	14	68	24	37	31	22
	58	68	25	31	41	15
ZNF140	42	86	31	55	48	63
	80	105	48	43	54	13
ZNF141	12	84	20	44	29	59
	56	79	41	33	60	16
ZNF142	59	230	51	135	107	130
	135	164	69	72	117	41
ZNF143	79	234	57	109	137	169
	155	280	111	99	139	25
ZNF146	173	941	239	681	494	643
	718	919	320	271	487	133
ZNF148	251	912	254	964	601	906
	770	1208	564	315	617	206
ZNF154	41	110	12	36	44	18
	85	102	54	25	38	19
ZNF155	33	25	27	41	22	39
	26	59	40	23	45	7
ZNF157	16	2	1	1	1	2
	0	3	4	1	2	0
ZNF16	20	38	8	24	14	27
	48	55	28	12	37	9
ZNF160	64	226	39	172	151	150
	186	241	122	88	184	61
ZNF165	15	4	5	2	4	5
	1	5	14	2	2	3
ZNF167	22	27	24	12	18	22
	32	57	19	9	26	6
ZNF169	23	48	13	39	42	29
	30	47	33	26	33	11
ZNF17	22	120	24	81	48	59
	90	128	56	51	87	27
ZNF174	18	81	14	49	42	69
	64	75	37	21	30	11
ZNF175	29	95	32	60	54	33
	68	115	41	34	55	15
ZNF177	0	1	1	0	0	2
	1	0	0	0	0	0
ZNF18	22	51	21	41	30	55
	34	73	40	16	38	14
ZNF180	30	65	20	40	37	54
	38	75	42	22	46	14
ZNF181	82	292	86	208	150	214
	268	316	144	82	217	58
ZNF182	32	71	13	49	34	50
	30	46	39	28	28	11

ZNF184	46	109	43	73	61	84
	83	98	60	27	59	20
ZNF185	36	21	14	25	18	8
	20	16	24	6	12	6
ZNF187	39	195	39	115	85	136
	104	172	76	42	98	37
ZNF189	48	206	74	198	143	160
	222	259	130	89	158	77
ZNF19	22	20	9	27	10	16
	9	26	16	7	23	3
ZNF192	99	512	156	432	266	360
	378	660	287	190	333	93
ZNF193	35	72	28	50	35	41
	60	70	42	16	47	11
ZNF195	49	165	47	113	102	121
	130	214	69	52	89	36
ZNF197	84	295	84	228	212	273
	205	296	135	126	172	59
ZNF2	31	43	22	28	30	27
	42	48	33	17	30	8
ZNF20	4	10	7	18	5	10
	5	28	7	4	10	4
ZNF200	40	55	25	22	18	26
	44	63	40	16	31	10
ZNF202	56	68	24	49	35	48
	51	74	39	16	43	15
ZNF205	21	12	7	14	11	19
	5	26	14	4	11	7
ZNF207	185	852	182	605	380	682
	710	1086	440	320	600	182
ZNF208	67	65	51	54	49	31
	49	60	57	18	42	18
ZNF211	41	80	29	43	30	47
	52	116	63	31	44	19
ZNF212	27	56	11	38	26	27
	52	56	29	17	35	10
ZNF213	10	14	9	13	5	17
	11	8	15	6	9	4
ZNF214	22	9	24	8	18	11
	9	26	10	8	13	3
ZNF215	22	3	2	8	5	1
	2	15	10	2	4	1
ZNF217	46	203	45	119	62	60
	104	123	64	31	83	17
ZNF219	24	69	21	50	29	48
	39	40	25	14	29	9
ZNF22	37	162	37	92	78	113
	153	219	95	68	88	30
ZNF221	33	36	27	15	19	23
	26	25	24	20	27	21
ZNF222	23	15	14	8	6	10
	9	17	11	6	18	1
ZNF223	23	30	11	13	13	12
	18	37	25	13	28	6
ZNF224	39	95	23	63	42	54
	61	110	63	41	49	30
ZNF225	21	32	17	13	25	43
	14	42	36	18	28	11

ZNF226	57	115	45	85	57	58
	66	166	84	58	90	33
ZNF227	47	101	42	66	55	67
	56	121	66	42	81	29
ZNF229	41	49	27	21	22	13
	40	34	40	16	28	15
ZNF23	39	100	25	102	46	69
	72	140	72	49	78	16
ZNF230	18	29	19	20	17	16
	21	28	20	15	14	5
ZNF232	25	35	12	19	23	19
	44	54	21	23	27	20
ZNF233	20	11	14	3	8	8
	10	15	21	4	12	7
ZNF234	47	106	53	75	70	78
	54	107	55	34	74	22
ZNF235	40	50	25	36	46	37
	44	67	36	27	44	20
ZNF236	117	355	147	246	195	274
	241	344	189	118	240	60
ZNF238	207	1249	228	1128	733	1088
	881	888	530	371	603	167
ZNF239	19	22	17	25	18	23
	23	18	14	6	22	5
ZNF24	293	1357	301	1254	846	1190
	1211	1855	707	430	878	277
ZNF248	134	486	131	274	201	329
	321	467	183	109	248	72
ZNF25	171	800	206	759	440	901
	725	922	456	326	565	161
ZNF250	38	122	33	64	62	57
	65	82	44	30	47	25
ZNF251	52	147	47	91	64	109
	107	234	74	45	102	34
ZNF253	61	293	68	131	136	128
	155	254	93	98	181	48
ZNF254	80	255	39	178	79	114
	147	250	117	76	159	52
ZNF256	9	48	10	28	32	14
	36	77	25	10	29	9
ZNF257	25	11	10	15	12	1
	8	21	7	1	18	2
ZNF259	47	153	22	75	73	109
	81	195	76	30	84	23
ZNF26	29	62	19	33	43	38
	46	74	36	19	48	12
ZNF260	60	169	45	108	82	105
	83	171	90	55	105	29
ZNF263	38	112	33	68	46	76
	59	137	58	40	73	24
ZNF264	111	400	100	312	202	270
	264	445	204	139	228	67
ZNF266	34	110	47	88	51	56
	63	105	108	41	94	36
ZNF267	38	78	22	55	41	66
	59	69	57	35	70	21
ZNF268	72	144	38	67	68	71
	91	119	57	31	99	42

ZNF273	67	214	42	191	110	121
	78	131	41	54	133	21
ZNF274	39	100	31	51	50	62
	75	149	56	42	79	37
ZNF275	40	99	16	47	55	46
	60	92	41	24	52	11
ZNF276	45	116	27	75	48	78
	95	177	69	39	63	35
ZNF277	40	124	33	85	71	107
	137	139	91	49	71	25
ZNF28	40	32	17	25	20	28
	13	20	23	11	29	10
ZNF280A	5	1	4	1	3	0
	0	0	5	0	3	0
ZNF280B	27	11	16	13	2	18
	6	7	14	1	9	1
ZNF280C	46	53	23	59	53	47
	50	52	33	28	47	15
ZNF280D	292	1291	375	1083	744	1119
	890	1660	601	473	694	255
ZNF281	49	213	60	173	133	173
	200	269	97	62	130	49
ZNF282	35	81	22	67	42	54
	94	108	48	23	41	18
ZNF283	23	73	18	31	34	34
	19	45	32	27	30	14
ZNF284	18	28	14	21	17	13
	27	32	21	7	22	7
ZNF285	41	28	20	13	19	17
	27	24	29	16	26	10
ZNF286A	51	57	40	41	43	61
	48	44	37	15	36	7
ZNF286B	32	18	19	8	12	14
	19	27	16	4	24	4
ZNF287	40	60	22	43	21	33
	29	55	42	17	44	11
ZNF292	307	1131	302	907	705	1054
	902	1138	545	383	636	216
ZNF295	54	72	40	59	50	68
	91	98	55	32	80	26
ZNF296	3	0	1	1	0	0
	1	0	2	0	1	0
ZNF3	69	192	45	161	102	148
	165	288	104	77	160	54
ZNF30	23	63	15	32	29	34
	23	39	24	16	29	9
ZNF300	21	30	23	24	10	10
	13	32	23	4	21	6
ZNF302	94	529	101	420	238	346
	366	719	244	194	340	143
ZNF304	50	110	33	79	75	67
	97	151	82	41	62	22
ZNF311	21	7	15	3	4	0
	3	2	16	0	4	1
ZNF317	50	133	44	77	99	104
	97	154	85	48	100	34
ZNF318	107	307	78	200	158	222
	252	271	161	97	150	62

ZNF319	9	45	12	33	16	21
	18	26	14	7	24	7
ZNF32	43	235	51	271	112	253
	298	309	178	83	150	41
ZNF320	73	113	45	115	94	112
	89	138	90	50	85	32
ZNF322	96	252	87	219	145	245
	209	334	150	88	200	47
ZNF323	26	57	31	73	59	50
	35	97	87	29	49	10
ZNF324	19	32	11	23	15	16
	23	32	17	10	24	7
ZNF324B	12	14	6	12	8	12
	14	21	11	3	10	1
ZNF326	59	187	66	122	86	96
	189	211	98	72	170	38
ZNF329	15	25	8	9	13	9
	11	19	13	8	16	4
ZNF330	160	546	111	337	340	397
	636	644	265	225	374	98
ZNF331	61	117	40	90	63	96
	95	120	68	41	66	29
ZNF333	66	136	42	121	76	111
	112	227	89	60	113	39
ZNF334	28	43	12	27	10	9
	21	57	29	14	18	5
ZNF335	51	113	43	86	64	88
	136	186	93	57	79	24
ZNF337	45	171	44	112	78	102
	126	161	75	49	96	38
ZNF33A	198	919	209	611	468	632
	596	845	374	313	505	184
ZNF33B	53	152	56	77	42	95
	104	105	132	66	84	40
ZNF34	20	24	12	21	21	25
	21	24	13	14	21	8
ZNF341	22	22	13	24	19	14
	32	36	24	7	17	8
ZNF343	41	142	32	72	62	117
	106	120	62	35	84	18
ZNF345	22	53	23	39	23	31
	31	46	34	9	37	4
ZNF346	49	108	31	73	39	65
	70	117	43	42	51	19
ZNF347	37	69	26	42	55	39
	51	75	65	26	66	24
ZNF35	25	72	24	44	22	25
	49	63	35	22	39	12
ZNF350	29	50	21	46	34	33
	52	99	49	26	44	16
ZNF354A	20	49	16	30	26	45
	51	35	28	20	32	11
ZNF354B	27	61	27	59	47	72
	57	92	32	25	58	21
ZNF354C	28	81	44	53	47	57
	88	97	57	32	60	18
ZNF358	40	178	20	95	59	163
	187	153	82	38	119	22

ZNF362	33	144	28	83	53	104
	97	111	61	30	73	24
ZNF365	50	3	30	2	19	3
	0	4	25	0	10	1
ZNF366	28	60	17	20	22	20
	55	42	35	30	35	10
ZNF367	11	22	15	12	8	21
	9	28	23	5	21	6
ZNF37A	115	383	113	235	136	219
	289	408	173	129	205	85
ZNF382	7	16	11	10	7	2
	5	9	14	5	4	5
ZNF383	28	77	22	54	21	29
	70	99	57	25	60	16
ZNF384	51	134	39	87	66	100
	133	138	44	44	67	35
ZNF385A	12	5	15	14	3	3
	6	17	3	1	4	2
ZNF385B	38	92	46	31	33	40
	94	133	49	33	48	24
ZNF385C	19	5	7	1	5	5
	6	3	11	1	9	2
ZNF385D	18	35	17	18	14	11
	29	32	17	14	22	9
ZNF391	11	22	14	9	7	18
	16	6	13	5	15	2
ZNF394	35	109	21	75	51	66
	91	149	72	42	60	23
ZNF395	77	241	56	181	117	154
	184	242	134	53	114	38
ZNF396	28	9	12	8	5	4
	4	9	20	2	8	0
ZNF397	39	162	55	111	84	117
	137	198	57	35	99	30
ZNF398	70	233	60	143	100	146
	159	246	98	61	122	55
ZNF404	14	29	10	7	21	13
	10	58	21	15	28	10
ZNF407	113	571	124	366	247	379
	316	571	259	172	329	88
ZNF408	4	19	8	14	6	17
	22	25	19	10	19	4
ZNF41	92	362	62	269	168	226
	262	280	123	102	164	52
ZNF410	65	150	41	109	92	122
	136	216	103	43	98	27
ZNF414	4	3	0	2	1	3
	9	6	3	3	5	1
ZNF415	19	26	14	16	24	14
	28	40	35	20	29	3
ZNF416	25	39	17	28	16	20
	30	73	32	20	23	15
ZNF417	41	131	39	102	85	74
	87	189	87	54	128	45
ZNF418	23	54	14	36	22	24
	50	51	39	19	38	16
ZNF419	15	82	18	49	26	28
	52	82	52	25	44	17

ZNF420	19	59	21	52	27	28
	32	62	34	19	41	13
ZNF423	56	360	65	212	141	214
	245	183	86	78	127	48
ZNF425	15	20	13	19	14	11
	3	15	12	7	13	4
ZNF426	24	60	21	40	43	19
	49	83	33	23	66	12
ZNF428	19	106	16	39	31	60
	116	112	43	19	38	10
ZNF429	31	61	26	57	44	65
	28	50	32	31	38	10
ZNF43	73	148	52	100	52	86
	98	117	86	47	101	34
ZNF430	40	76	25	56	27	42
	48	53	46	20	41	7
ZNF431	56	202	75	151	97	189
	130	238	104	75	143	55
ZNF432	22	68	23	48	36	43
	31	63	39	21	45	13
ZNF433	23	49	20	32	32	23
	41	60	31	20	48	19
ZNF434	35	46	23	31	31	29
	35	45	33	24	29	9
ZNF436	125	576	78	444	153	343
	246	315	154	94	164	62
ZNF438	82	294	94	177	159	229
	227	247	155	106	165	43
ZNF439	14	25	13	14	12	11
	8	19	20	7	10	9
ZNF44	28	127	42	77	39	70
	78	120	53	58	87	32
ZNF440	35	74	24	60	48	34
	60	82	45	29	60	26
ZNF441	41	223	69	121	91	109
	125	240	142	76	165	50
ZNF442	12	26	15	15	19	9
	9	23	20	5	16	4
ZNF443	14	42	15	40	41	11
	28	40	38	13	29	11
ZNF444	3	19	10	14	8	15
	17	33	10	12	14	7
ZNF445	140	505	133	322	218	351
	251	310	191	97	204	71
ZNF446	3	8	6	9	8	8
	5	15	7	2	7	2
ZNF449	71	165	71	137	84	149
	165	182	74	63	80	34
ZNF45	51	120	48	84	56	67
	82	153	73	27	78	22
ZNF451	199	665	269	647	453	667
	684	1107	512	289	665	183
ZNF454	14	6	9	6	4	1
	0	3	5	0	6	0
ZNF460	140	590	184	520	324	438
	397	691	318	173	398	143
ZNF461	18	42	16	23	24	24
	24	49	27	18	32	8

ZNF462	208	940	260	708	478	777
	713	524	331	253	362	121
ZNF467	3	9	4	4	5	4
	7	3	5	3	2	0
ZNF468	22	29	14	12	16	21
	22	19	25	13	40	13
ZNF469	21	18	15	11	7	18
	23	11	12	8	11	5
ZNF470	53	74	65	56	47	52
	55	75	64	26	46	15
ZNF471	125	156	64	62	68	43
	65	97	93	47	102	28
ZNF473	34	35	21	38	32	23
	40	58	20	10	39	11
ZNF474	11	2	1	1	4	0
	0	1	5	0	2	1
ZNF479	18	0	12	0	9	0
	0	0	16	0	9	0
ZNF48	7	17	8	9	8	14
	10	19	10	8	9	3
ZNF480	121	264	85	126	148	205
	219	353	130	91	221	47
ZNF483	53	24	27	29	26	19
	18	29	43	11	30	11
ZNF484	68	140	57	116	76	118
	161	161	83	40	100	25
ZNF485	28	34	19	22	24	31
	28	39	25	15	29	10
ZNF486	28	20	13	15	15	17
	10	13	29	9	20	4
ZNF488	11	2	4	0	0	0
	1	1	1	0	6	0
ZNF490	38	100	35	45	53	57
	68	90	54	32	56	23
ZNF491	14	35	11	30	36	11
	20	63	38	19	37	10
ZNF492	28	3	11	4	10	1
	3	7	17	3	12	3
ZNF493	112	334	119	251	152	234
	246	418	220	128	201	75
ZNF496	32	68	21	41	33	43
	41	68	34	18	53	14
ZNF497	10	4	2	2	10	0
	0	1	5	0	1	1
ZNF498	49	119	42	74	68	65
	81	153	75	40	92	15
ZNF500	18	33	8	18	20	25
	34	41	26	10	18	7
ZNF501	28	55	24	28	45	49
	36	79	29	25	36	10
ZNF502	50	80	37	30	70	48
	47	87	47	38	45	13
ZNF503	8	23	9	31	9	18
	6	20	5	6	9	2
ZNF506	66	242	66	121	130	168
	187	283	132	65	166	56
ZNF507	137	624	126	387	238	369
	536	608	246	205	382	114

ZNF510	66	241	80	170	138	138
	159	256	114	69	138	59
ZNF511	33	159	20	111	71	147
	184	229	73	50	123	33
ZNF512	65	146	36	95	74	98
	135	169	93	44	115	33
ZNF512B	52	94	40	67	51	59
	84	98	52	20	69	17
ZNF513	14	21	12	15	7	14
	17	37	13	8	19	5
ZNF514	73	208	77	127	134	139
	143	130	84	71	93	25
ZNF516	103	384	124	235	191	326
	234	479	210	86	195	69
ZNF517	6	6	4	9	11	5
	12	15	11	3	7	3
ZNF518A	153	745	164	547	302	418
	428	574	305	195	396	119
ZNF518B	93	487	81	304	183	374
	334	387	169	99	213	84
ZNF519	53	120	56	74	56	85
	72	116	91	48	103	24
ZNF521	31	179	47	121	61	145
	76	135	69	32	47	33
ZNF524	17	44	3	23	15	19
	29	48	20	11	17	5
ZNF526	22	58	12	33	26	43
	24	53	32	18	39	14
ZNF527	45	98	43	42	55	65
	62	84	64	39	59	32
ZNF528	57	139	44	67	71	85
	57	156	88	60	83	32
ZNF529	45	121	38	60	63	72
	98	154	114	40	129	39
ZNF530	16	16	7	9	4	8
	4	27	9	6	16	2
ZNF532	258	1084	280	902	552	837
	812	1179	596	419	683	225
ZNF534	7	4	11	3	4	3
	1	3	11	0	4	1
ZNF536	13	4	8	3	4	6
	6	4	12	3	11	5
ZNF540	28	38	25	26	34	44
	38	74	28	14	42	15
ZNF541	22	7	11	0	2	3
	1	3	7	0	5	4
ZNF543	45	91	32	94	74	56
	65	107	62	31	64	21
ZNF544	21	72	21	10	36	15
	19	69	26	20	31	24
ZNF546	42	143	28	60	76	81
	65	116	58	37	63	15
ZNF547	17	35	5	14	21	13
	14	35	16	6	26	14
ZNF548	48	134	45	89	68	83
	103	178	67	45	89	27
ZNF549	39	90	39	57	64	54
	52	109	61	44	66	29

ZNF550	13	24	10	7	7	5
	18	17	21	5	18	6
ZNF551	21	73	35	61	31	26
	77	151	72	35	55	19
ZNF552	21	60	19	29	19	33
	52	46	19	19	27	14
ZNF554	24	41	22	27	16	23
	38	54	45	17	41	14
ZNF555	56	118	44	44	67	57
	100	151	68	40	90	41
ZNF556	13	13	12	3	5	8
	12	12	17	0	7	6
ZNF557	30	80	24	37	48	43
	44	94	44	32	50	14
ZNF558	35	74	20	61	47	47
	61	81	47	26	47	17
ZNF559	1	11	1	4	0	13
	22	15	4	5	8	9
ZNF559-ZNF177		33	25	29	7	15
	11	14	22	22	10	23
	9					
ZNF560	23	0	9	0	6	0
	0	0	19	0	6	0
ZNF561	52	151	38	103	80	62
	108	135	75	42	83	29
ZNF562	67	135	38	50	60	56
	61	192	74	34	76	30
ZNF563	15	26	11	16	21	17
	18	22	24	14	12	3
ZNF564	61	171	36	105	75	108
	129	193	101	55	88	39
ZNF565	14	38	16	28	23	31
	19	57	20	10	26	7
ZNF566	54	156	45	124	83	103
	109	165	87	48	104	29
ZNF567	31	49	20	25	26	34
	27	43	29	22	30	20
ZNF568	62	135	98	62	56	64
	76	83	92	40	86	30
ZNF569	27	82	36	48	30	39
	45	67	40	18	34	19
ZNF57	11	1	11	2	4	4
	5	18	8	4	4	3
ZNF570	24	53	19	15	20	25
	32	45	21	12	25	14
ZNF571	27	31	15	29	20	38
	22	22	27	11	21	12
ZNF572	14	17	13	7	12	1
	5	10	18	6	14	2
ZNF573	43	96	38	62	70	50
	55	81	52	37	73	17
ZNF574	16	35	8	32	17	23
	33	39	14	20	21	8
ZNF575	3	4	1	3	0	9
	10	6	1	0	2	1
ZNF576	12	40	8	30	16	21
	30	37	15	10	35	2

ZNF577	53	165	55	80	86	104
	124	236	94	63	110	31
ZNF578	44	26	23	8	12	13
	10	19	29	9	19	4
ZNF579	13	30	13	13	14	29
	30	34	25	9	14	3
ZNF580	11	14	12	5	10	8
	13	19	11	5	9	2
ZNF581	7	11	12	16	14	26
	19	21	5	3	8	4
ZNF582	9	11	9	9	7	10
	6	10	17	5	20	2
ZNF583	24	42	21	34	26	24
	31	52	37	13	33	9
ZNF584	18	50	11	27	21	24
	43	56	22	17	34	12
ZNF585A	34	77	20	42	51	40
	42	63	50	26	50	17
ZNF585B	37	99	33	41	39	60
	61	91	66	23	61	25
ZNF586	30	167	37	64	76	77
	83	189	77	49	91	28
ZNF587	77	315	85	147	146	138
	140	255	120	88	140	61
ZNF587B	52	160	31	74	70	72
	101	202	115	48	94	29
ZNF589	43	102	36	76	41	107
	78	133	46	41	57	30
ZNF592	156	674	128	412	273	416
	534	622	250	220	338	119
ZNF593	18	79	9	41	16	63
	94	131	60	12	25	5
ZNF594	54	228	84	129	119	159
	130	219	111	84	141	52
ZNF595	74	192	77	104	103	125
	135	238	115	89	131	36
ZNF596	15	31	15	41	20	20
	22	31	37	17	35	9
ZNF597	18	14	7	8	12	11
	15	30	15	7	17	4
ZNF598	19	39	20	36	24	38
	41	44	23	22	19	11
ZNF599	14	18	14	18	16	13
	25	28	28	6	15	8
ZNF600	10	24	7	24	9	12
	18	12	25	8	16	8
ZNF605	94	456	127	190	202	282
	225	364	145	109	166	87
ZNF606	53	131	43	73	67	94
	99	158	85	40	89	32
ZNF607	18	40	22	17	22	20
	31	54	35	14	22	12
ZNF608	65	269	91	151	92	157
	148	182	102	37	106	22
ZNF609	105	334	86	252	136	279
	230	247	142	102	182	58
ZNF610	21	8	12	9	12	5
	8	21	25	2	15	2

ZNF611	28	55	30	51	32	39
	45	49	38	23	40	16
ZNF613	24	36	17	28	15	28
	23	30	17	19	20	11
ZNF614	41	124	36	67	61	69
	90	138	63	41	93	36
ZNF615	50	104	33	46	39	57
	61	100	59	33	61	32
ZNF616	29	52	27	47	47	56
	49	57	25	21	28	13
ZNF618	55	114	59	57	49	67
	56	52	50	11	70	16
ZNF619	34	68	18	45	36	55
	47	90	33	25	44	9
ZNF620	20	32	11	18	15	22
	20	32	23	21	28	0
ZNF621	104	295	83	189	148	165
	229	340	143	105	179	59
ZNF622	49	127	30	98	75	109
	106	142	88	47	82	23
ZNF623	51	182	38	152	95	111
	119	180	71	45	101	33
ZNF624	16	42	28	53	32	35
	33	51	42	16	28	8
ZNF625	4	17	5	6	4	4
	7	9	11	7	12	2
ZNF626	78	142	52	142	72	137
	79	158	108	46	105	33
ZNF627	27	60	11	36	39	34
	41	66	31	16	40	22
ZNF628	9	19	6	9	4	3
	8	5	10	5	10	1
ZNF629	55	188	54	87	69	111
	116	222	64	71	112	53
ZNF630	15	26	8	12	9	26
	15	13	6	7	13	6
ZNF638	648	2598	648	2112	1513	1930
	1640	3370	1384	1003	1639	561
ZNF639	75	320	70	301	165	264
	299	387	153	94	173	70
ZNF641	77	376	88	363	120	368
	249	228	167	139	182	48
ZNF642	19	8	10	8	6	4
	3	9	15	4	11	3
ZNF643	13	8	8	2	1	6
	4	2	6	1	6	0
ZNF644	252	1163	314	1049	655	862
	907	1162	547	376	732	231
ZNF645	8	0	1	0	2	1
	0	1	7	0	0	0
ZNF646	38	71	25	61	47	52
	62	100	62	32	50	29
ZNF648	15	1	3	1	3	1
	0	4	5	1	1	0
ZNF649	27	69	27	39	33	52
	42	94	35	36	53	21
ZNF652	215	924	175	976	515	713
	541	783	343	213	462	148

ZNF653	13	4	7	7	5	9
	3	8	5	5	5	1
ZNF654	83	280	73	206	149	180
	191	207	96	64	147	41
ZNF655	141	515	119	334	258	349
	349	553	249	185	282	114
ZNF658	81	191	81	139	85	151
	146	173	98	56	128	42
ZNF660	12	17	6	8	7	9
	7	15	11	6	13	3
ZNF662	80	154	39	106	69	80
	78	159	56	54	93	35
ZNF664	7	111	6	107	19	99
	161	129	12	24	23	39
ZNF664-FAM101A		17	21	5	13	8
	13	17	36	9	6	9
	6					
ZNF665	25	11	11	13	4	3
	2	13	20	4	15	2
ZNF667	23	23	18	9	15	8
	10	18	14	13	21	8
ZNF668	17	13	9	6	16	17
	22	19	21	6	12	3
ZNF669	13	32	13	42	17	16
	30	32	17	13	23	14
ZNF670	2	5	3	14	3	6
	8	23	7	4	4	3
ZNF671	34	106	29	54	65	44
	105	179	76	51	78	20
ZNF672	50	159	29	89	104	120
	140	223	95	35	102	30
ZNF673	26	57	19	51	25	45
	47	59	31	26	40	11
ZNF674	35	55	19	38	22	38
	40	53	28	22	45	14
ZNF675	18	41	9	42	13	28
	26	39	20	9	26	7
ZNF676	19	11	12	3	13	6
	1	16	13	5	8	8
ZNF677	21	18	19	15	14	9
	20	16	17	7	18	3
ZNF678	83	149	63	121	80	130
	117	164	116	54	119	37
ZNF679	16	0	5	0	7	3
	0	1	6	0	7	1
ZNF680	178	742	174	654	444	644
	511	752	349	228	456	142
ZNF681	40	37	20	17	22	29
	4	32	23	6	22	4
ZNF682	16	26	14	21	13	10
	26	7	26	3	15	3
ZNF683	16	0	1	0	3	0
	0	1	4	0	2	0
ZNF684	20	33	11	40	37	42
	28	41	24	15	29	6
ZNF687	36	47	25	29	27	32
	33	56	29	17	36	8

ZNF688	12	40	14	36	24	34
	48	74	33	11	23	8
ZNF689	9	27	5	11	24	29
	30	42	20	14	30	8
ZNF69	12	10	6	14	3	7
	7	16	12	4	5	3
ZNF691	13	40	7	24	17	16
	37	30	18	10	27	10
ZNF692	35	98	34	49	63	83
	53	110	53	33	35	15
ZNF695	5	0	7	3	6	1
	2	2	4	3	1	2
ZNF696	33	38	13	15	21	35
	20	55	29	15	19	10
ZNF697	36	59	34	36	35	68
	50	76	37	17	33	7
ZNF699	12	22	16	20	14	19
	29	17	24	6	23	6
ZNF7	28	140	28	115	52	87
	101	167	54	31	86	23
ZNF70	22	58	17	43	28	53
	57	96	27	34	34	14
ZNF700	36	92	37	70	50	67
	66	135	65	50	68	26
ZNF701	27	63	35	54	36	31
	61	70	42	13	43	19
ZNF703	7	30	4	17	12	10
	28	13	10	14	3	17
ZNF704	168	514	161	490	338	433
	423	559	272	208	384	180
ZNF705A	45	2	24	1	15	4
	1	5	14	1	10	1
ZNF705B	17	0	8	0	6	2
	0	0	10	0	3	0
ZNF705D	52	2	26	3	19	3
	0	0	34	1	27	0
ZNF705E	12	0	3	6	3	3
	1	0	2	2	4	0
ZNF705G	6	0	1	0	3	0
	0	0	3	0	5	0
ZNF706	57	338	96	222	174	238
	288	404	134	89	168	67
ZNF707	5	16	5	7	6	3
	9	13	8	1	8	2
ZNF708	50	118	31	80	61	73
	101	100	50	42	60	39
ZNF709	24	50	19	32	37	33
	40	49	41	16	30	12
ZNF71	26	48	27	24	28	16
	40	43	24	20	29	12
ZNF710	14	37	13	26	17	17
	22	32	12	10	18	5
ZNF711	37	35	33	31	55	35
	27	68	29	21	28	14
ZNF713	30	39	20	16	20	34
	40	14	17	14	35	8
ZNF714	67	152	32	80	79	75
	64	109	94	39	83	31

ZNF716	24	0	8	0	6	1
	0	0	14	0	13	0
ZNF717	124	126	78	71	85	99
	82	119	96	40	82	35
ZNF718	28	56	23	24	22	43
	49	56	47	9	30	9
ZNF720	17	45	26	43	30	53
	43	59	60	10	36	9
ZNF721	94	398	97	246	198	238
	220	338	156	150	255	106
ZNF726	13	5	11	5	7	6
	1	7	10	1	11	0
ZNF727	24	37	31	9	30	35
	16	52	39	18	26	11
ZNF728	14	9	7	4	8	5
	0	9	13	4	6	2
ZNF729	18	1	15	0	6	0
	1	0	11	0	8	1
ZNF730	14	2	4	1	3	1
	1	0	4	1	5	0
ZNF732	14	1	10	2	5	1
	3	0	11	1	8	1
ZNF735	6	0	11	0	5	0
	0	0	3	0	4	0
ZNF736	28	75	62	63	42	58
	46	68	54	21	41	10
ZNF737	82	105	52	77	85	69
	70	77	77	46	78	23
ZNF74	22	37	15	39	17	47
	22	33	27	10	31	6
ZNF740	66	200	56	163	111	126
	180	276	104	74	103	46
ZNF746	26	59	20	58	32	50
	49	69	47	15	39	9
ZNF747	8	34	8	17	17	24
	34	31	13	8	25	7
ZNF749	14	38	15	20	13	10
	20	19	8	9	13	3
ZNF750	15	1	8	0	4	0
	2	1	9	0	2	2
ZNF75A	42	104	28	69	55	63
	87	140	55	37	68	29
ZNF75D	34	87	25	86	50	73
	53	95	39	17	53	17
ZNF76	64	268	65	229	140	213
	234	249	172	74	174	46
ZNF761	31	95	31	38	25	48
	75	79	46	22	57	17
ZNF763	18	20	15	12	9	17
	31	27	22	12	25	8
ZNF764	14	53	12	32	23	31
	39	37	25	17	26	12
ZNF765	29	38	22	38	30	24
	23	66	34	29	33	11
ZNF766	32	85	38	63	44	62
	66	104	56	37	68	28
ZNF768	63	247	41	143	113	155
	191	248	88	80	140	32

ZNF77	14	28	6	24	32	34
	28	25	17	2	25	6
ZNF770	229	1058	201	784	592	708
	842	1076	426	221	606	161
ZNF771	4	9	1	7	7	3
	13	34	11	1	8	4
ZNF772	82	145	51	153	130	103
	247	370	253	81	245	65
ZNF773	16	24	12	11	7	8
	12	35	15	15	13	6
ZNF774	21	31	9	18	13	18
	51	52	21	42	36	13
ZNF775	4	6	4	9	5	10
	8	8	5	5	2	0
ZNF776	87	269	59	162	148	159
	240	355	154	108	195	58
ZNF777	26	68	13	33	22	29
	42	44	34	19	29	11
ZNF778	34	88	30	72	68	74
	71	100	60	29	88	18
ZNF780A	82	214	90	187	116	169
	150	325	152	79	144	67
ZNF780B	149	479	161	328	233	354
	295	555	244	126	317	83
ZNF781	8	18	13	10	17	1
	12	10	9	2	6	2
ZNF782	54	181	64	92	84	122
	155	228	103	75	104	43
ZNF783	32	70	36	63	39	53
	66	100	45	29	48	19
ZNF784	11	70	10	22	34	41
	65	103	22	28	44	10
ZNF785	14	37	10	20	13	23
	21	41	12	8	22	5
ZNF786	22	30	11	23	21	22
	30	26	17	12	21	7
ZNF787	6	72	2	35	21	35
	33	77	20	19	32	6
ZNF789	18	45	9	34	19	11
	21	38	23	7	15	9
ZNF79	16	59	25	30	28	41
	29	55	19	18	29	10
ZNF790	31	87	36	61	47	61
	45	68	44	27	66	15
ZNF791	49	182	60	115	110	130
	155	241	116	84	152	44
ZNF792	27	66	17	39	29	32
	25	51	36	13	26	10
ZNF793	56	72	32	25	27	17
	37	34	31	24	53	16
ZNF799	18	36	13	31	26	28
	25	34	25	7	23	10
ZNF8	16	28	7	12	13	15
	7	16	9	3	10	7
ZNF80	24	1	9	1	4	1
	1	0	11	0	5	0
ZNF800	62	238	68	191	139	140
	223	233	155	138	181	82

ZNF804A	43	0	12	1	14	0
	0	0	12	0	6	0
ZNF804B	35	0	25	0	13	0
	0	2	13	0	9	1
ZNF805	93	296	90	212	160	228
	252	434	161	81	202	67
ZNF806	19	1	15	0	7	0
	0	0	12	0	4	0
ZNF808	52	265	57	121	105	165
	207	237	115	76	153	58
ZNF81	73	108	53	90	53	86
	71	119	69	40	53	21
ZNF812	17	0	8	0	8	2
	0	0	4	0	6	0
ZNF813	17	20	29	15	24	23
	12	22	23	4	18	4
ZNF814	40	138	44	66	68	49
	100	164	86	59	109	40
ZNF816	0	3	1	3	3	10
	11	3	11	6	5	1
ZNF816-ZNF321P		21	7	8	4	4
	5	5	7	6	2	6
	2					
ZNF821	20	22	17	30	9	26
	28	31	17	8	19	4
ZNF823	22	34	16	36	23	22
	26	51	22	15	21	7
ZNF827	61	150	54	74	67	89
	104	106	67	20	65	23
ZNF829	60	48	43	31	41	29
	36	47	38	18	43	5
ZNF83	53	118	23	135	59	72
	91	166	100	46	83	33
ZNF830	30	103	22	63	63	104
	93	139	52	48	71	17
ZNF831	52	6	23	0	10	2
	1	5	15	0	12	1
ZNF835	9	14	3	3	7	7
	17	10	7	2	8	4
ZNF836	1	10	2	8	0	5
	10	9	2	0	1	3
ZNF837	3	2	1	0	0	0
	0	1	1	1	1	0
ZNF839	45	253	52	201	110	159
	171	214	136	85	140	37
ZNF84	109	277	104	206	185	224
	237	337	149	124	188	58
ZNF841	46	134	52	99	58	65
	87	127	78	44	86	36
ZNF843	4	1	1	2	5	3
	3	4	3	0	1	0
ZNF844	43	176	45	58	96	24
	110	196	76	58	101	58
ZNF845	20	26	17	29	20	19
	23	39	36	7	20	7
ZNF846	19	55	19	41	24	27
	44	73	34	15	31	13

ZNF85	26	39	16	17	8	20
	20	33	28	11	24	7
ZNF850	41	32	18	16	12	16
	18	28	31	2	21	3
ZNF852	14	32	14	31	18	30
	34	35	27	8	18	12
ZNF853	41	171	28	81	40	81
	103	107	48	38	73	22
ZNF860	10	8	15	9	6	9
	1	3	9	3	6	1
ZNF862	39	98	36	52	40	57
	78	66	33	28	61	14
ZNF865	10	61	14	32	19	31
	29	28	18	14	23	7
ZNF878	9	0	4	0	2	0
	0	1	6	0	1	0
ZNF879	20	69	28	55	43	60
	32	60	36	16	55	9
ZNF880	36	73	24	37	25	43
	32	69	44	28	54	17
ZNF883	22	29	13	6	13	9
	16	12	11	7	15	7
ZNF888	10	4	8	1	7	6
	4	10	9	2	6	1
ZNF90	29	10	9	12	14	7
	2	13	19	8	11	2
ZNF91	657	3869	665	1948	856	1763
	2577	1930	814	938	1762	677
ZNF92	44	129	20	99	82	94
	63	106	63	24	96	30
ZNF93	37	19	24	22	32	18
	26	39	29	9	23	8
ZNF98	21	4	8	4	6	5
	1	0	13	0	2	1
ZNF99	32	4	24	3	10	7
	1	5	19	2	12	2
ZNFX1	124	520	133	335	236	320
	314	437	240	205	287	103
ZNHIT1	47	188	23	133	79	145
	215	292	101	39	113	35
ZNHIT2	4	9	2	8	2	10
	10	23	5	2	8	0
ZNHIT3	52	224	37	144	86	172
	199	350	108	70	147	36
ZNHIT6	76	224	60	149	135	171
	144	195	92	60	111	35
ZNRD1	11	33	14	27	17	38
	22	44	9	10	25	3
ZNRF1	50	224	41	149	90	134
	130	156	89	57	89	35
ZNRF2	62	266	92	250	91	186
	178	260	159	65	145	43
ZNRF3	43	64	36	42	38	45
	53	79	26	13	32	10
ZNRF4	7	0	0	0	1	0
	0	0	1	0	0	0
ZP1	7	0	8	0	3	0
	1	1	0	0	0	0

ZP2	26	0	15	0	2	0
	0	0	14	0	2	0
ZP3	7	3	8	4	1	6
	2	1	3	1	3	0
ZP4	19	0	14	0	2	0
	0	0	10	0	5	0
ZPBP	11	0	5	0	1	0
	0	0	6	0	5	0
ZPBP2	12	0	8	2	8	0
	0	0	9	0	7	0
ZPLD1	19	0	13	0	7	1
	0	1	10	0	7	0
ZRANB1	260	1094	243	1027	555	961
	1171	1691	788	369	1089	300
ZRANB2	216	962	193	746	414	732
	814	1150	538	329	660	199
ZRANB3	87	172	76	118	88	138
	130	153	105	56	110	32
ZRSR2	45	66	23	24	36	51
	30	33	26	30	39	13
ZSCAN1	1	0	5	0	0	0
	0	0	4	0	2	0
ZSCAN10	4	0	1	0	1	0
	0	0	7	0	0	0
ZSCAN12	38	42	10	21	16	23
	20	27	24	10	17	8
ZSCAN16	11	20	11	14	12	23
	14	33	10	5	10	5
ZSCAN18	27	106	11	48	35	37
	54	115	37	34	60	28
ZSCAN2	32	40	15	25	22	39
	37	41	27	7	24	7
ZSCAN20	29	53	29	25	34	38
	36	69	25	16	40	6
ZSCAN21	21	86	21	49	53	52
	66	95	28	23	31	6
ZSCAN22	27	15	12	11	15	16
	12	19	21	11	12	6
ZSCAN23	17	39	16	21	14	15
	13	25	22	12	21	4
ZSCAN29	61	171	43	144	102	133
	121	238	81	57	113	48
ZSCAN30	104	236	92	154	125	198
	209	348	117	79	180	44
ZSCAN4	9	2	5	0	3	1
	0	1	10	0	5	0
ZSCAN5A	11	7	8	2	3	7
	2	6	8	2	9	1
ZSCAN5B	6	0	4	1	3	0
	0	0	3	0	2	0
ZSCAN5D	8	0	2	0	0	0
	0	0	4	0	3	0
ZSWIM1	26	47	9	36	31	34
	43	39	26	21	29	16
ZSWIM2	17	0	18	0	9	0
	0	1	3	0	9	1
ZSWIM3	20	21	8	11	13	16
	19	19	7	4	11	3

ZSWIM4	18	6	7	3	1	3
	5	4	12	2	9	2
ZSWIM5	69	57	47	56	28	67
	49	39	59	13	38	11
ZSWIM6	57	113	43	89	70	83
	70	105	64	24	60	18
ZSWIM7	49	165	47	133	67	91
	187	233	53	31	94	14
ZUFSP	15	44	27	28	29	42
	38	60	32	10	53	12
ZW10	114	354	105	230	185	296
	263	385	181	151	247	67
ZWILCH	46	51	22	43	28	55
	67	83	36	20	63	15
ZWINT	16	9	15	7	6	8
	7	4	7	2	4	1
ZXDA	64	251	54	219	106	163
	129	225	90	58	92	33
ZXDB	44	122	39	86	81	101
	89	124	62	34	83	20
ZXDC	376	1477	362	1102	776	1041
	1156	1974	748	622	1022	372
ZYG11A	36	1	10	0	5	1
	1	1	17	0	14	0
ZYG11B	1082	5936	933	5814	3732	4688
	4989	6965	2717	1763	3858	1074
ZYX	16	90	18	62	27	65
	73	93	35	7	32	30
ZZEF1	236	1049	247	734	458	598
	717	1031	479	327	566	232
ZZZ3	169	580	190	406	314	423
	453	617	276	201	398	114
